# Light-enabled metal-free carbo- and silyl functionalization of [1.1.1]propellane and [3.1.1]propellane

**DOI:** 10.1039/d6qo00901h

**Published:** 2026-08-11

**Authors:** Yongchen Wang, Martin Pauze, Spencer P. Pitre, Julian G. West

**Affiliations:** a Department of Chemistry, Bioscience Research Collaborative, Rice University Houston TX 77030 USA jgwest@rice.edu; b Department of Chemistry, Oklahoma State University Stillwater OK 74078 USA

## Abstract

1,3-Disubstituted bicyclo[1.1.1]pentanes (BCPs) have emerged as privileged scaffolds in pharmaceutical discovery and functional materials, often employed as bioisosteres of *para*-disubstituted benzenes. Existing synthetic approaches to these motifs typically rely on muti-step sequences or multi-component reactions and frequently require the participation of transition metals, photocatalysts and radical initiators. Herein, we report a modular and transition metal-free strategy for the synthesis of 1,3-disubstituted BCPs directly from [1.1.1]propellane through sequential C–C bond formation. The protocol enables efficient access to dichloro- and trichloro-substituted BCPs, which represent underexplored yet promising scaffolds for drug discovery. The strategy could be further extended to [3.1.1]propellane, affording 1,5-disubstituted bicyclo[3.1.1]heptanes (BCHs). Notably, this approach could also be extended to C–Si bond formation, providing rare silyl-substituted BCPs with potential applications in material science. Overall, this work establishes a general, catalyst-free platform for the modular functionalization of highly strained propellanes.

## Abstract 2

A light-driven, metal- and photocatalyst-free multicomponent strategy converts [1.1.1]- and [3.1.1]propellane with CH_2_Cl_2_, CHCl_3_, or (Me_3_Si)_3_SiH and acrylamides into diverse chlorinated and silylated bis-BCP/BCH scaffolds.

## Graphical abstract

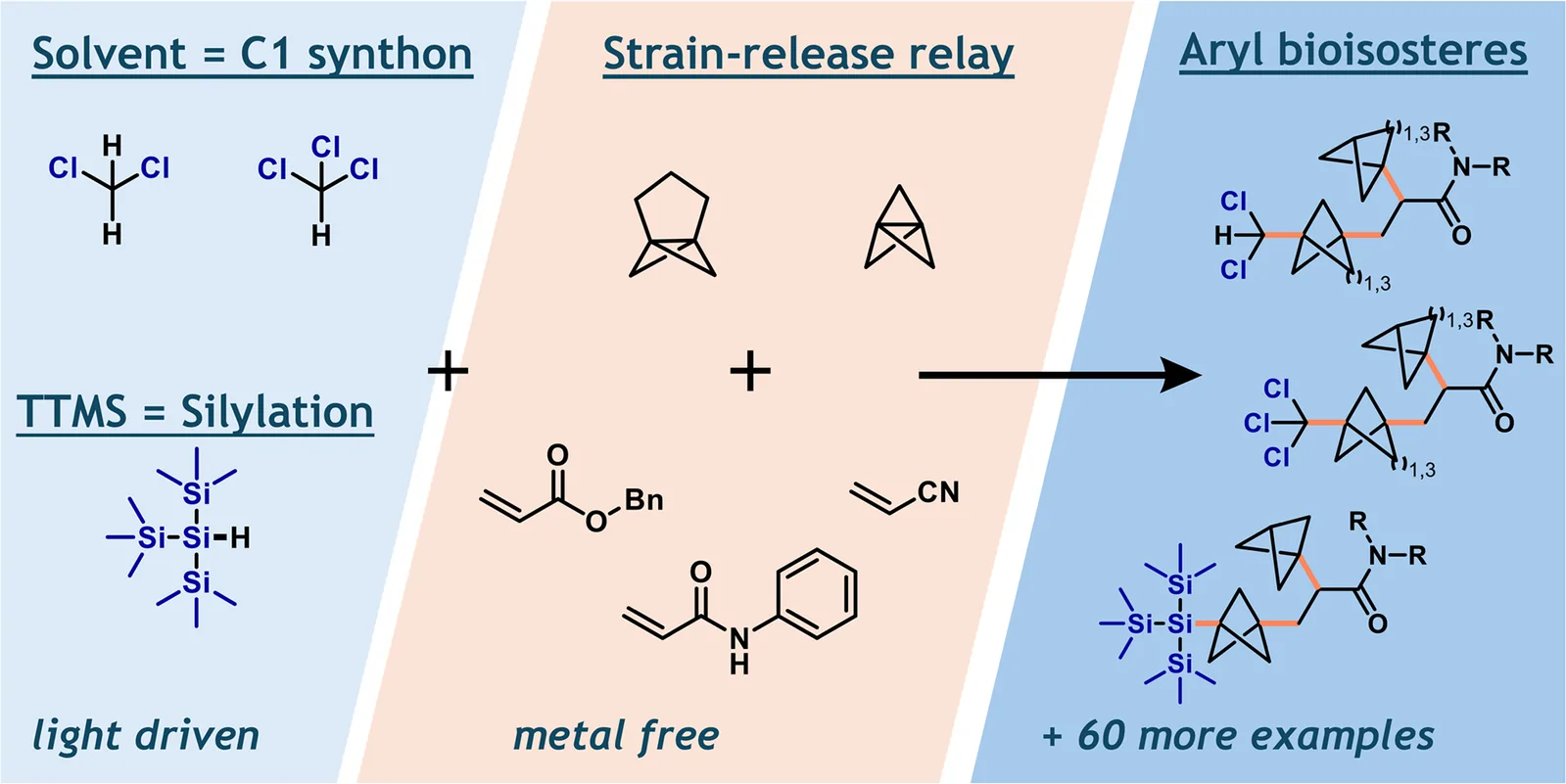

## Introduction

**Fig. 1:**
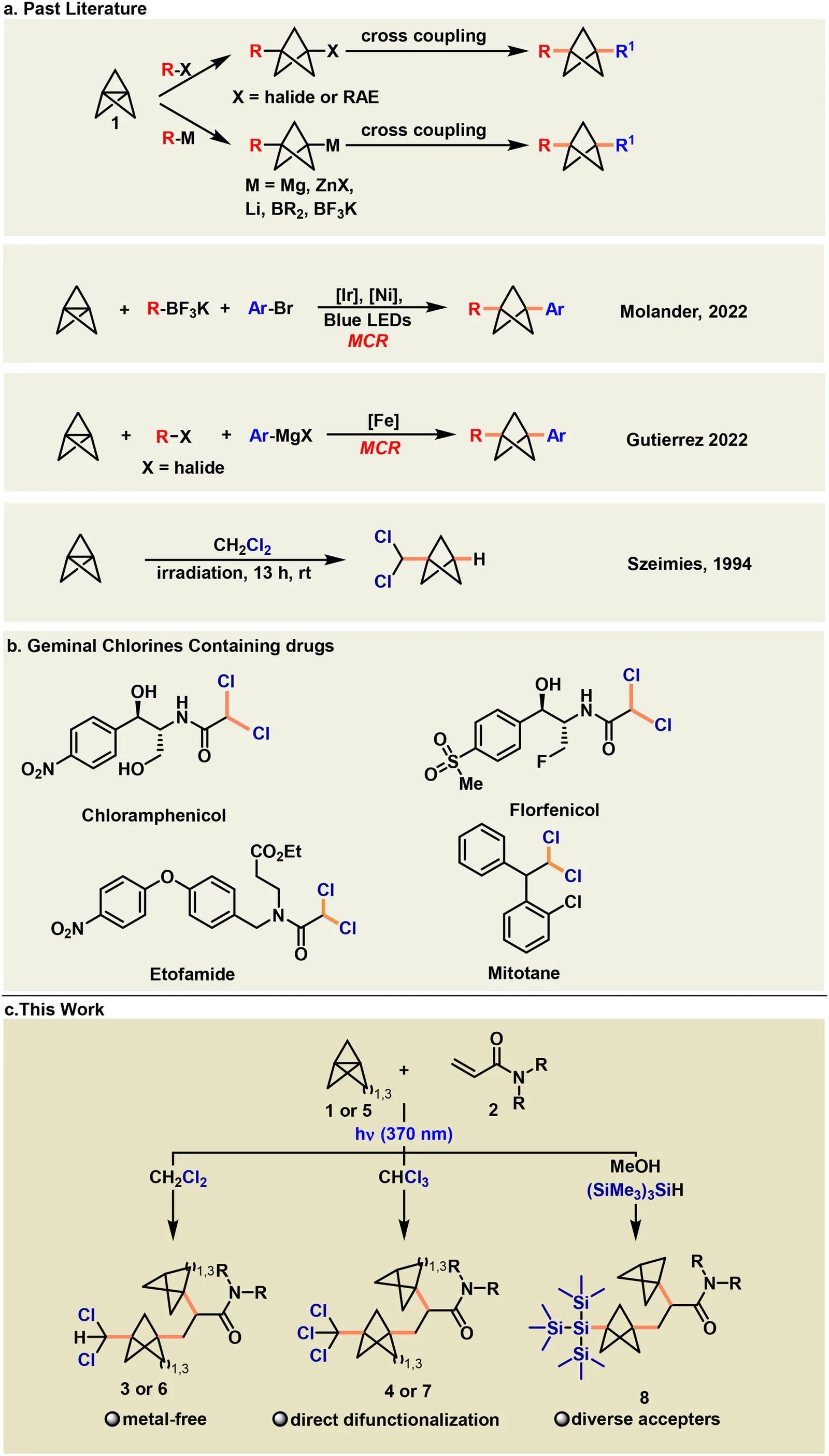
(a) Previous strategies for accessing BCPs *via* dual C–C bond formation, alongside approach to *gem*-dichloro-substituted BCP synthesis. (b) Approved drugs containing gemi-dichloromethyl motifs. (c) This work: light-driven, metal-free construction of BCPs *via* direct C–C and C–Si bonds formation, and of BCHs *via* direct C–C bond formation.

In pharmaceuticals, substituting 1,4-disubstituted arenes with 1,3-disubstituted bicyclopentanes (BCPs) has been shown to greatly improve the biological profiles^1^ of drug molecules including pharmacokinetics, metabolic stability, membrane permeability and solubility.^2,3^ In the past few decades, 1,3-dicarbofunctionalized BCPs have been mainly obtained *via* two different transition metal catalyzed cross-coupling strategies (Fig. 1a). For example, [1.1.1]propellane (1) can be converted to bicyclo[1.1.1]pentylmetals (Mg, Zn, Li, *etc*.) and employed as nucleophiles to couple with (pseudo)halides using palladium, nickel, nickel/iridium dual catalysts and copper.^4–15^ Conversely, electrophilic bicyclo[1.1.1]pentyl derivatives can be generated from 1 and then coupled with organometallic reagents, mainly through iron or copper catalysis.^16–19^ Both strategies require two sequential steps to furnish the dicarbofunctionalized BCP with the involvement of transition metals, greatly reducing atom economy and sustainability. More recently, the Molander group disclosed a single-step approach to 1,3-dicarbofunctionalised BCPs, in which both C–C bonds are forged through a dual iridium/nickel catalytic manifold.^20^ In addition, Gutierrez and coworkers reported a one-pot, multicomponent Fe-catalyzed radical cross-coupling reaction that provides a rapid and pratical method to achieve the direct dicarbofunctionalization of 1.^18^

None of the aforementioned dicarbofunctionalization strategies affords access to chlorine-containing BCPs, despite the considerable potential of such motifs in medicinal chemistry. At present, more than 250 FDA-approved drugs on the market contain at least one chlorine atom, with many additional pharmaceutically important candidates progressing through pre-clinical development.^21^ In particular, *gem*-dichloromethyl motifs are widespread structural features in pharmaceutical agents, appearing across diverse therapeutic classes that include antiamoebic, antibiotic, antiprotozoal and anticancer drugs (Fig. 1b).^22^ Given the established significance of 1,3-substituted BCPs and the prominent role of chlorine atoms in pharmaceuticals, we hypothesized that multichlorinated dicarbofunctionalized BCPs could be synthesized for potential applications as drug candidates. Building on the report by Szeimies and coworkers who demonstrated that irradiation of 1 in dichloromethane (CH_2_Cl_2_) leads to dichloromethylated BCPs,^23^ we discovered that CH_2_Cl_2_ can play a more active role in the reaction manifold. Herein, we reported a facile, transition metal-free multicomponent reaction (MCR) to realize the dicarbofunctionalization of 1 and [3.1.1]propellane (5) with acrylamides (2) using CH_2_Cl_2_ and CHCl_3_ as radical precursors (Fig. 1c). Furthermore, we also demonstrated that we could access silylated BCPs in the presence of (Me_3_Si)_3_SiH (TTMS) and MeOH. Given the importance of these scaffolds in medicinal chemistry and other fields, our methodology has the potential to provide new, unique dual-BCP containing compounds with potentially meaningful biological activities, affording new avenues for future drug development.

## Results and discussion

Our study commenced with exploring the possibility of dicarbofunctionalization of 1 with *N*-phenylacrylamide (2a) and CH_2_Cl_2_ as source of dichloromethyl radical (Table 1).^24,25^ Initial experiments conducted in the absence of light, employing 2.0 equivalents of 1 at a concentration of 0.1 M in CH_2_Cl_2_, afforded product 3a in 21% yield (Table 1, entry 1). Dilution of the reaction mixture to 0.05 M resulted in a diminished yield (17%, Table 1, entry 2), while increasing the concentration to 0.2 M and 0.4 M further reduced the yield to 15% and 10%, respectively (Table 1, entries 3 and 4). Adjusting the stoichiometry of 1 revealed some influence on reaction efficiency. Using 1.0 equivalent of 1 led to a reduced yield of 11% (Table 1, entry 5), whereas increasing the loading to 3.0, 4.0, and 5.0 equivalents provided 3a in yields ranging from 15% to 22% (Table 1, entries 6–8). Extending the reaction time to 48 h with 2.0 equivalents of 1 improved the yield to 27% (Table 1, entry 9), although a similar outcome was observed when 3.0 equivalents of 1 were employed under identical conditions (Table 1, entry 10). Introduction of light proved to be a decisive factor. Upon irradiation with a 370 nm LED at room temperature for 24 h, the yield of 3a increased markedly to 58% when 2 equivalents of 1 was used (Table 1, entry 11). Prolonging the irradiation time to 48 h led to a decrease in yield to 38% (Table 1, entry 12). Similarly, increasing the amount of 1 to 3.0 and 4.0 equivalents under irradiation resulted in lower yields of 40% and 36%, respectively (Table 1, entries 13 and 14). On balance, the optimal conditions were identified as 2.0 equivalents of 1 with respect to 2a in 0.1 M CH_2_Cl_2_ under 370 nm LED irradiation at room temperature for 24 h. Notably, a side product arising from dimerization of BCP was also detected (see SI, section 3.4, staffane 3ae).

**Table 1:** Optimization of dichloromethylation of 1^a^

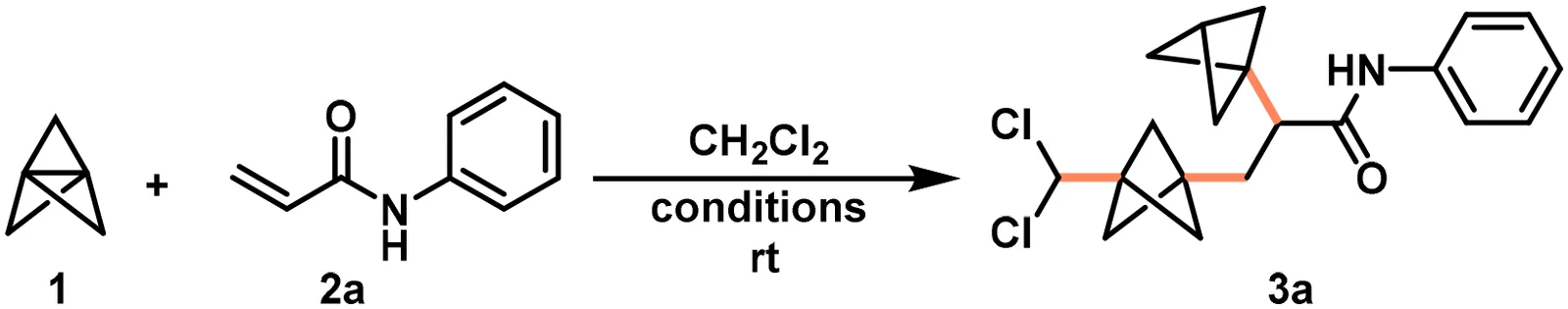					
Entry	CH_2_Cl_2_ conc. (M)	Light^b^	1 (equiv.)	Time (h)	Yield (%)
1	0.1	None	2.0	24	21
2	0.05	None	2.0	24	17
3	0.2	None	2.0	24	15
4	0.4	None	2.0	24	10
5	0.1	None	1.0	24	11
6	0.1	None	3.0	24	22
7	0.1	None	4.0	24	20
8	0.1	None	5.0	24	15
9	0.1	None	2.0	48	27
10	0.1	None	3.0	48	27
11	0.1	370 nm	2.0	24	58
12	0.1	370 nm	2.0	48	38
13	0.1	370 nm	3.0	24	40
14	0.1	370 nm	4.0	24	36

a Reactions were carried out using *N*-phenylacrylamide 2a (0.1 mmol, 1.0 equiv.) and [1.1.1]propellane 1 (0.1–0.5 mmol, 1.0–5.0 equiv.) in CH_2_Cl_2_ at rt without light or irradiation of 370 nm Kessil blue LED (25% intensity, 11 W).

b “None” means in the dark. Isolated yields are reported.

**Fig. 2:**
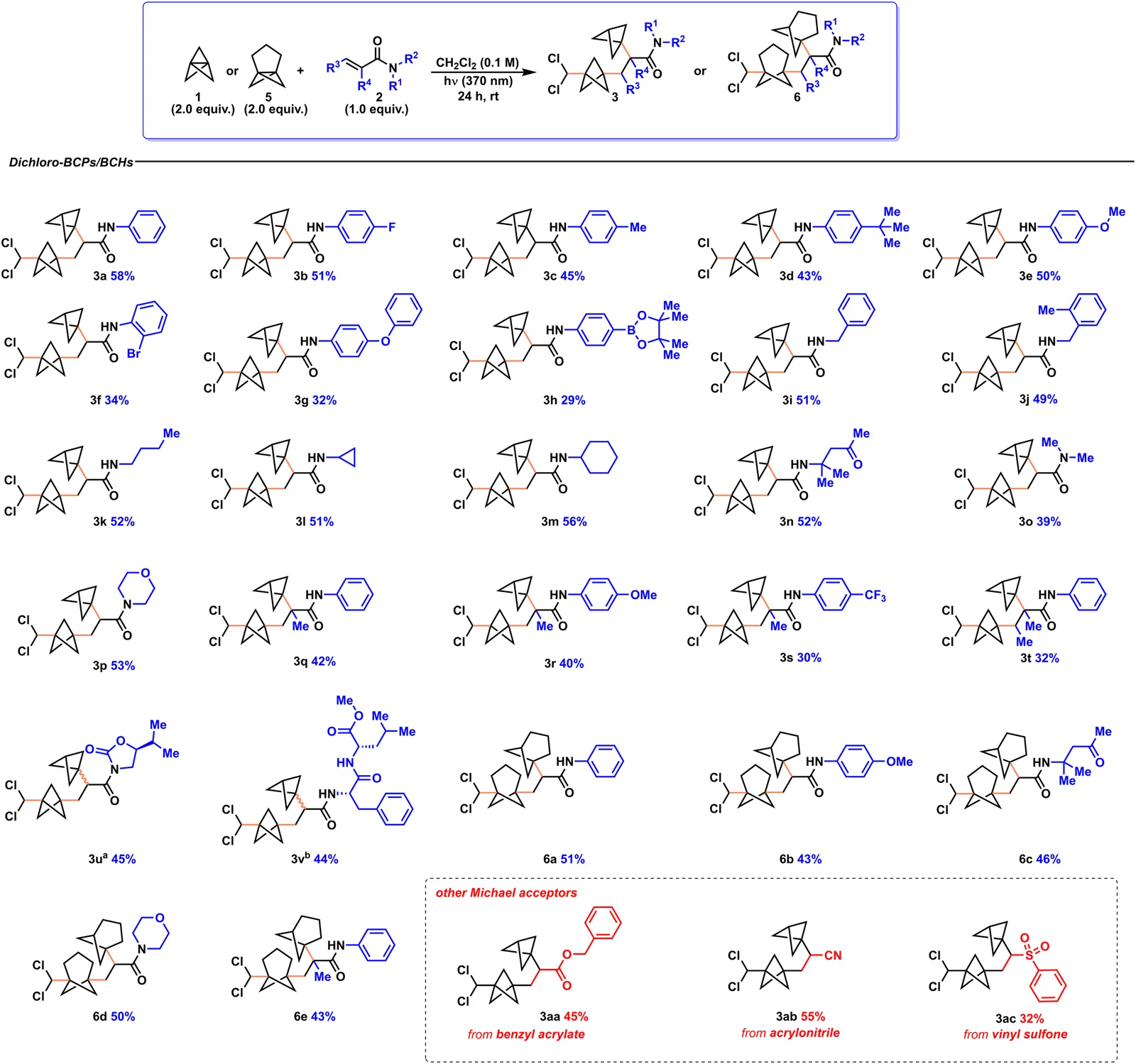
Scope of dichloromethylation of 1 or 5. Reaction conditions: acrylamide 2 (0.1 mmol, 1.0 equiv.), [1.1.1]propellane 1 (0.2 mmol, 2.0 equiv.) and CH_2_Cl_2_ (0.1 M), 24 h, rt, 370 nm Kessil blue LED (25% intensity, 11 W). Yields are isolated yields. ^*a*^ Combined yield of two individually isolated diastereomers. ^*b*^ Isolated as inseparable diastereomers, 1 : 1 dr (see SI for detailed information).

With the optimized conditions in hand, we began exploring the substrate applicability of this MCRs approach. Pleasingly, the synthesis of 1,1-dichloro-BCPS with two BCP cores was found to be applicable to a wide range of acrylamides (Fig. 2). Phenyl acrylamides bearing electron withdrawing (3b, 51%) or electron donating substituents (3c–3e, 43%–50%) both gave us moderate yield of desired product, although acrylamides with an *ortho*-bromophenyl group (3f, 34%) and *para*-phenoxyphenol group (3g, 32%) offered us the desired product in slightly reduced yields. An acrylamide bearing a boronic ester (3h) was also accommodated under the reaction conditions, albeit in low yield (29%) on account of product degradation during isolation. *N*-Benzylacrylamides 3i and 3j were also tolerated, providing the desired product in 51% and 49%, respectively. Aliphatic acrylamides bearing *n*-butyl, cyclopropyl, and cyclohexyl groups all reacted smoothly to provide the desired products in good yields (3k–3m). An acrylamide bearing a dimethylbutanone group also successfully yielded product 3n in 52% yield. Tertiary acrylamides including *N*,*N*-dimethylacrylamide and 4-acryloylmorpholine also yielded the corresponding products 3o and 3p in 39% and 53%, respectively. Notably, phenyl methacrylamides bearing electron-neutral, electron-donating and electron-withdrawing substituents were also accommodated, furnishing reasonable yield of desired products (3q–3s, 30%–42%). The more challenging α,β-dimethylacrylamide also delivered the corresponding product 3t in 32% yield. Furthermore, an acrylamide with a chiral auxiliary was subjected to the reaction conditions and two separable diastereomers of product were successfully obtained in reasonable yield (3u, 45%). Additionally, a dipeptide-bearing acrylamide was also tolerated, giving product 3v as a mixture of inseparable diastereomers in 44% yield. To further explore the substrate applicability of the MCRs, 1 was replaced with [3.1.1]propellane 5 and subjected to our standard reaction conditions. Pleasingly, a variety of 1,1-dichloro-BCHs containing two BCH cores were obtained in good yield (6a–6e, 43%–51%). Finally, several other Michael acceptors were also accommodated for the MCRs, including benzyl acrylate (3aa, 45%), acrylonitrile (3ab, 55%) and phenyl vinyl sulfone (3ac, 32%).

**Fig. 3:**
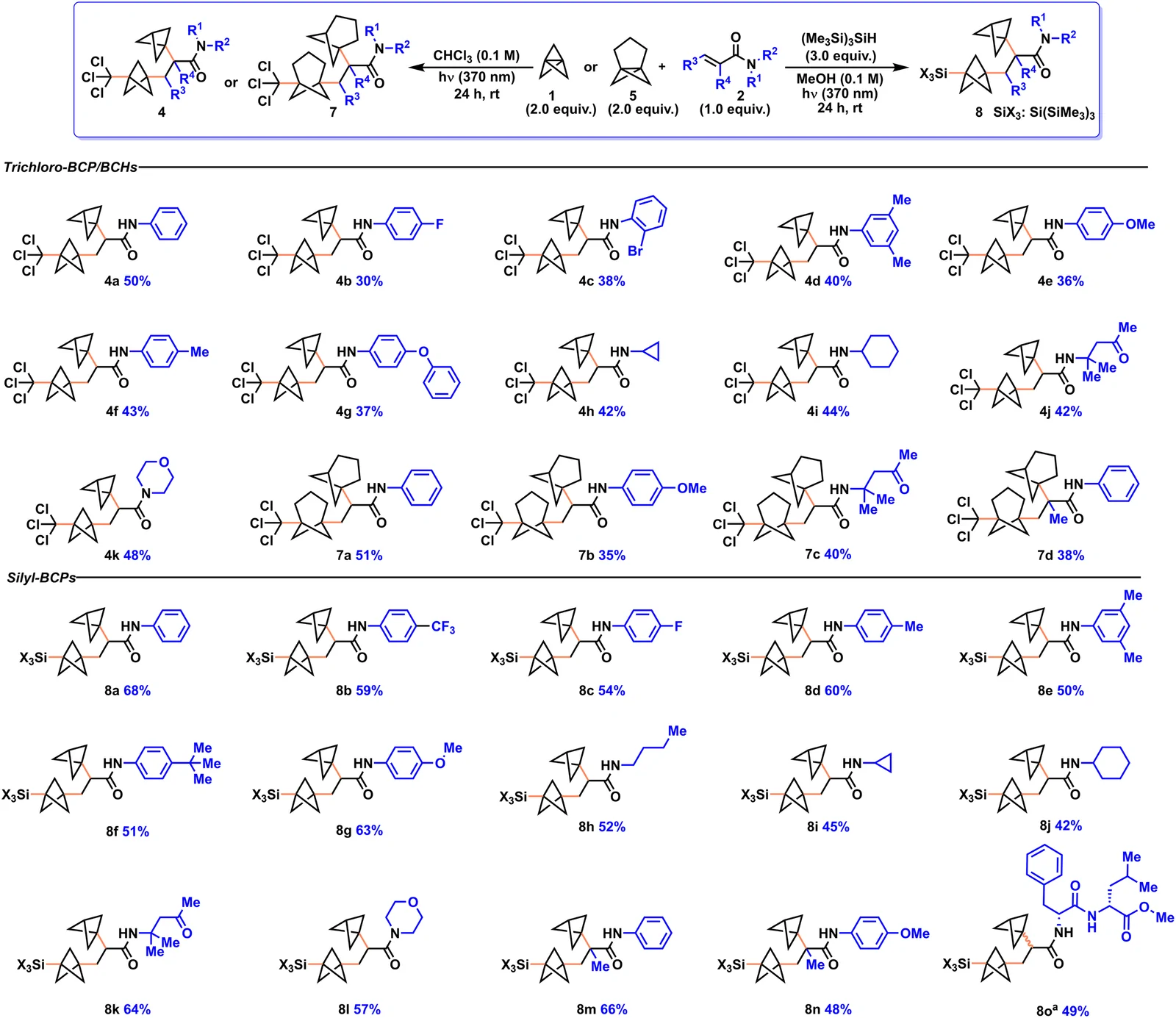
Scope of trichloromethylation of 1 or 5 and silylation of 1. Reaction conditions for trichloromethylation: acrylamide 2 (0.1 mmol, 1.0 equiv.), [1.1.1]propellane 1 or [3.1.1]propellane 5 (0.2 mmol, 2.0 equiv.) and CHCl_3_ (0.1 M), 24 h, rt, 370 nm Kessil blue LED (25% intensity, 11 W). Reaction conditions for silylation: acrylamide 2 (0.1 mmol, 1.0 equiv.), [1.1.1]propellane 1 (0.2 mmol, 2.0 equiv.), TTMS (0.3 mmol, 3.0 equiv.) and MeOH (0.1 M), 24 h, rt, 370 nm Kessil blue LED (25% intensity, 11 W). Yields are isolated yields. ^*a*^ Isolated as inseparable diastereomers, 1 : 1 dr (see SI for detailed information) and reaction was carried out with 0.05 mmol scale.

Replacement of CH_2_Cl_2_ with CHCl_3_ likewise enabled the transformation to proceed successfully. Our investigation also began with phenylacrylamide 2a, which was subjected to similar reaction conditions as the reaction conditions as the CH_2_Cl_2_ reaction and the desired product 4a with a 1,1,1-trichloro-BCP backbone was obtained in 50% yield (Fig. 3). Phenylacrylamides featuring electron-withdrawing groups (4b, 4c) and electron-donating groups (4d–4g) are all well suited to the MCRs, giving reasonable yield of desired product (30–43%).

A wide range of aliphatic acrylamides (4h–4j) were also demonstrated to be applicable to the MCR, offering corresponding products in moderate yield (42%–44%). Tertiary acrylamides like 4-acryloylmorpholine also reacted smoothly, providing 4k in 48% yield. Replacing 1 with 5 also proceeded smoothly under the standard reaction conditions, yielding a range of 1,1,1-trichloro-BCHs containing two BCH backbones (7a–7d, 35–51%) (Fig. 3). We next investigated whether silane reagents could trap the intermediate α-keto radicals *via* hydrogen-atom transfer (HAT), which, if successful, could lead to predominant formation of 1,1-dichloro or 1,1,1-trichloro BCPs containing only a single BCP core. Unfortunately, a number of silane reagents were screened but none of them delivered the desired product in reasonable yields.

To our delight, when TTMS was employed into this reaction, silylated-BCPs were observed as the primary product, providing an attractive alternative for C–Si bond formation (Fig. 3). Addition of TTMS to the mixture of 1, acrylamide 2a and CH_2_Cl_2_ in the absence of light provided silylated BCP 8a in 27% yield (see SI, Table S5). After optimization of the various reaction parameters, the yield of 8a could be increased to 68% under 370 nm irradiation. Next, the scope for the reaction was explored. Phenylacrylamides bearing electron-withdrawing groups (8b, 8c) and electron-donating groups (8d–8g) all reacted smoothly, delivering the corresponding products in good yields (50%–63%). Aliphatic acrylamides also successfully rendered the desired products in good yields (8h–8k, 42%–64%). 4-Acryloylmorpholine gave the product 8i with 57% yield and α-methyl-substituted phenylacrylamides also successfully produced the corresponding products 8m and 8n in 66% and 48% yield, respectively. An acrylamide bearing a dipeptide was also accommodated, furnishing the desired product 8o in 49% yield. Unfortunately, incorporation of TTMS into [3.1.1] propellane (5) was not successful and the direct attack of the silane on the acrylamide was observed (SI section 3 for additional information).

**Fig. 4:**
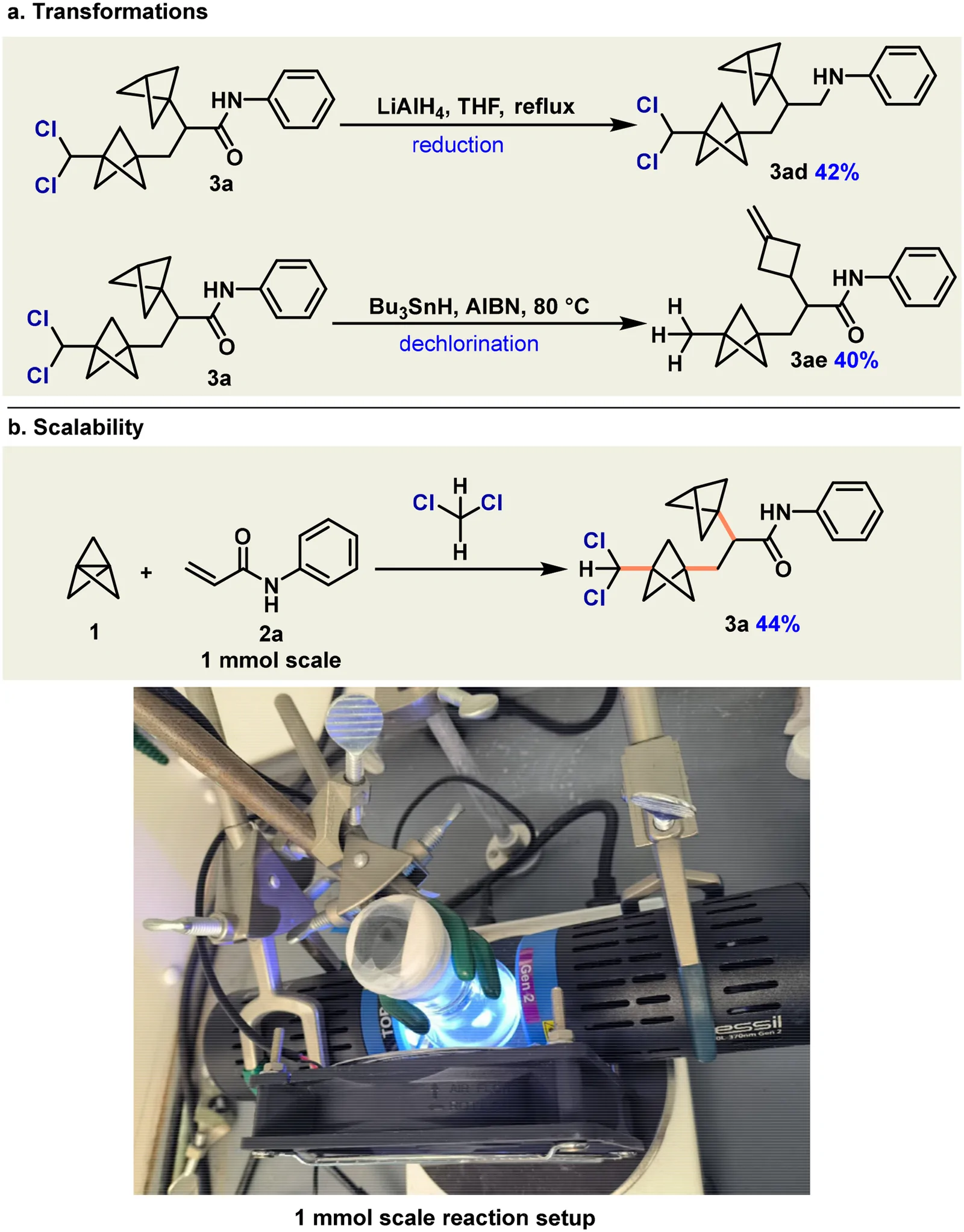
Application of the MCRs. (a) Transformations of 3a. (b) Scaling up the dichloromethylation MCR.

With *gem*-dichloromethyl BCP 3a in hand, we proceeded to examine potential downstream transformations (Fig. 4). Reduction of 3a with LiAlH_4_ proceeded smoothly to afford the corresponding amine 3ad in 42% yield (Fig. 4a). Under radical dehalogenation conditions using Bu_3_SnH and AIBN, 3a underwent rearrangement to deliver product 3ae in 40% yield. Notably, scaling the dichloromethylation multicomponent reaction to the 1 mmol level had no detrimental effect on efficiency, giving 3a reproducibly in a moderate yield of 44% (Fig. 4b).

**Fig. 5:**
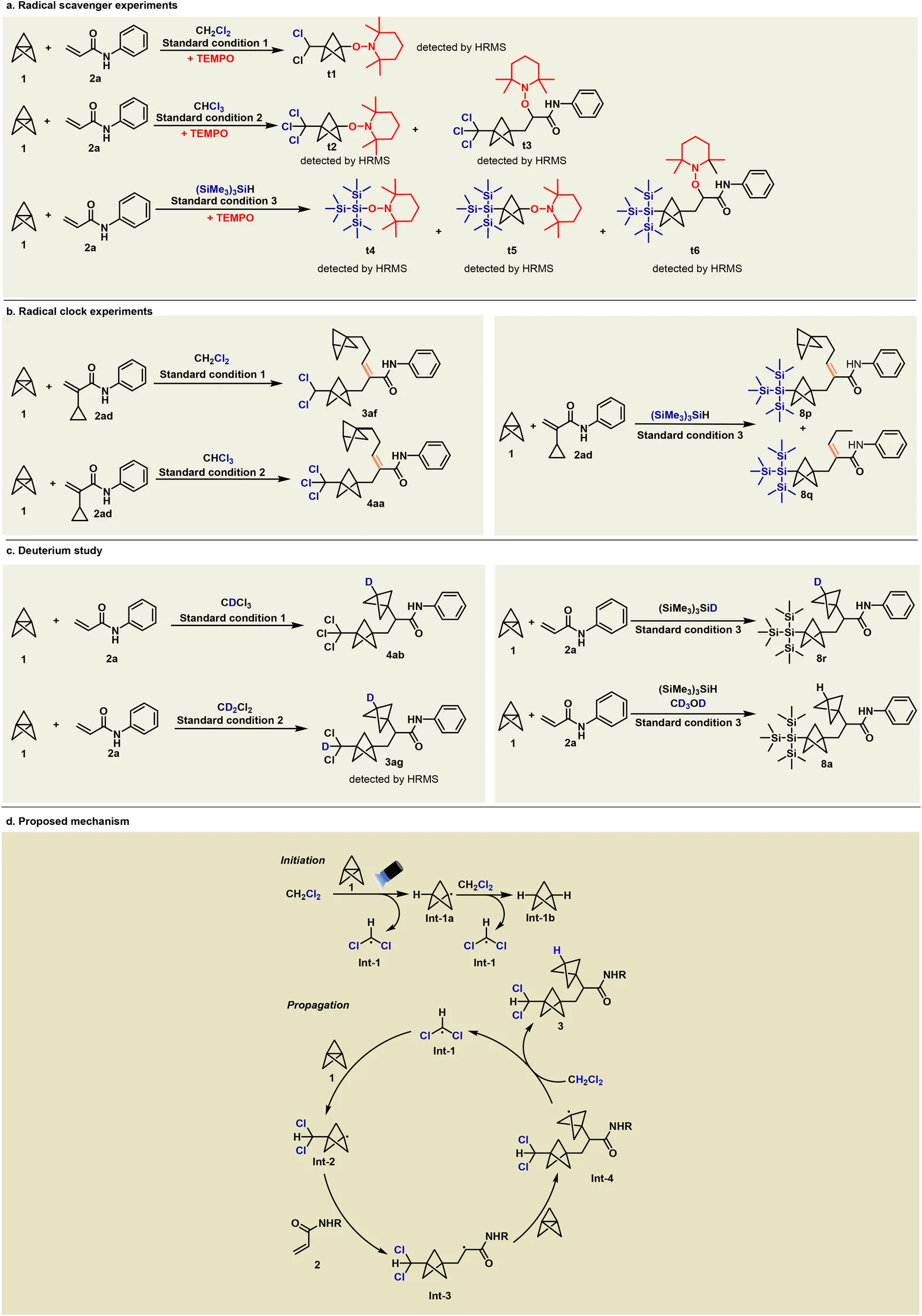
Mechanistic studies of the MCRs. (a) Radical scavenger experiments. Standard condition 1: 1 (2.0 equiv., 0.2 mmol), 2a or 2ad (1.0 equiv., 0.1 mmol), CH_2_Cl_2_ or CD_2_Cl_2_ (0.1 M), 24 h, rt, 370 nm Kessil blue LED (25% intensity, 11 W). Standard condition 2: 1 (2.0 equiv., 0.2 mmol), 2a or 2ad (1.0 equiv., 0.1 mmol), CHCl_3_ or CDCl_3_ (0.1 M), 24 h, rt, 370 nm Kessil blue LED (25% intensity, 11 W). Standard condition 3: 1 (2.0 equiv., 0.2 mmol), 2a or 2ad (1.0 equiv., 0.1 mmol), TTMS or d-TTMS (3.0 equiv.), MeOH or CD_3_OD (0.1 M), 24 h, rt, 370 nm Kessil blue LED (25% intensity, 11 W). 1.0 equivalent of TEMPO was added for each condition. (b) Radical clock experiments. 0.05 mmol of 2-cyclopropyl-*N*-phenylacrylamide 2ad was used for each condition. (c) Deuterium labelling experiment.

The efficiency of the reaction as well as the novelty of the products prompted our exploration of the reaction mechanisms (Fig. 5). First, different equivalents of radical scavenger 2,2,6,6-tetramethyl-1-piperidinyloxy (TEMPO) were added to our standard conditions for each protocol and several TEMPO adducts intermediates (t1 to t6) were detected by HRMS analysis, supporting the existence of radical intermediates (Fig. 5a). To provide further support for a radical pathway, radical clock 2ad was subjected to our standard reaction conditions, furnishing the desired ring opening products 3af (10%), 4aa (6%) and 8p (14%), respectively (Fig. 5b). It is worth noting that subjecting 2ad to our silylation conditions also rendered ring-opened product 8q bearing a single BCP moiety in 38% yield. These results strongly support a radical process. Replacing CHCl_3_ with CDCl_3_ led to complete deuteriation product 4ab, suggesting the hydrogen atom of the second BCP core comes from CHCl_3_ and further supports the intermediacy of trichloromethyl radicals (Fig. 5c). However, replacing CH_2_Cl_2_ with CD_2_Cl_2_ led to only trace deuterium incorporation in the product, as detected by HRMS analysis. This observation indicates that the C–D bonds in CD_2_Cl_2_ are significantly stronger than the corresponding C–H bonds, rendering them less susceptible to radical-mediated processes such as deuterium atom abstraction (see SI section 4 for additional information). The deuteration experiment employing TTMS-*d* proceeded smoothly and furnished the desired deuterated product 8r. In contrast, when CD_3_OD was used under otherwise identical conditions, no deuterium incorporation was observed and only 8a was observed. These results unambiguously indicate that the hydrogen atom incorporated into the second BCP core originates from TTMS rather than from MeOH (see SI section 4 for additional information). We further measured the UV-vis absorption spectra of 1 in CH_2_Cl_2_ and CHCl_3_, as well as mixtures of 1 with 2a in either CH_2_Cl_2_ or CHCl_3_. They all exhibited appreciable absorbance under the conditions employed although the absorption in CHCl_3_ solution is less than that in CH_2_Cl_2_ solution (see SI section 4 for additional information).

The proposed mechanism for the transformation is displayed on Fig. 5d. Based on our investigation, the mechanism is likely to follow a radical chain pathway. Plausible initiation and propagation steps are outlined below. During initiation, dichloromethyl radical Int-1 adds to 1 to generate the BCP-centered radical Int-2 which subsequently reacts with an acrylate 2 to afford Int-3. α-Keto radical Int-3 then couples with a second equivalent of 1 to form Int-4, which undergoes hydrogen atom transfer (HAT) with CH_2_Cl_2_ to deliver the product 3 while propagating the chain. Notably, while the reaction proceeded in the absence of irradiation (21% yield of 3a without light), we propose that irradiation likely increases the probability of chain initiating events, resulting in improved reaction efficiency. Recent studies provide a compelling rationale for the persistence of [1.1.1]propellane reactivity in the absence of light irradiation. Notably, the Dell'Amico group reported the hydrosilylation of [1.1.1]propellane without irradiation and addition of initiators, demonstrating that this strained hydrocarbon is capable of initiating a radical chain process without photochemical activation.^26^ Complementary computational investigations by the Duarte group has revealed the partial biradical character of [1.1.1]propellane, lending further support to its intrinsic propensity for radical engagement.^27^ This electronic structure feature is consistent with our proposed mechanism, in which [1.1.1]propellane could act as a hydrogen atom transfer (HAT) reagent, abstracting hydrogen from dichloromethane and thereby thermally initiating the reaction sequence. In addition, earlier work from the Waddell group documented analogous reactivity patterns, showing that [1.1.1]propellane can undergo transformations with various partners without initiators.^28^ Collectively, these findings reinforce the notion that [1.1.1]propellane possesses an inherent capacity to promote radical processes under purely thermal conditions. Additionally, the reaction still proceeds smoothly when CH_2_Cl_2_ is replaced by CHCl_3_ and TTMS, supporting the potential involvement of 1 in the radical initiating step (see section 4 of the SI for a detailed mechanistic study).

**Fig. 6:**
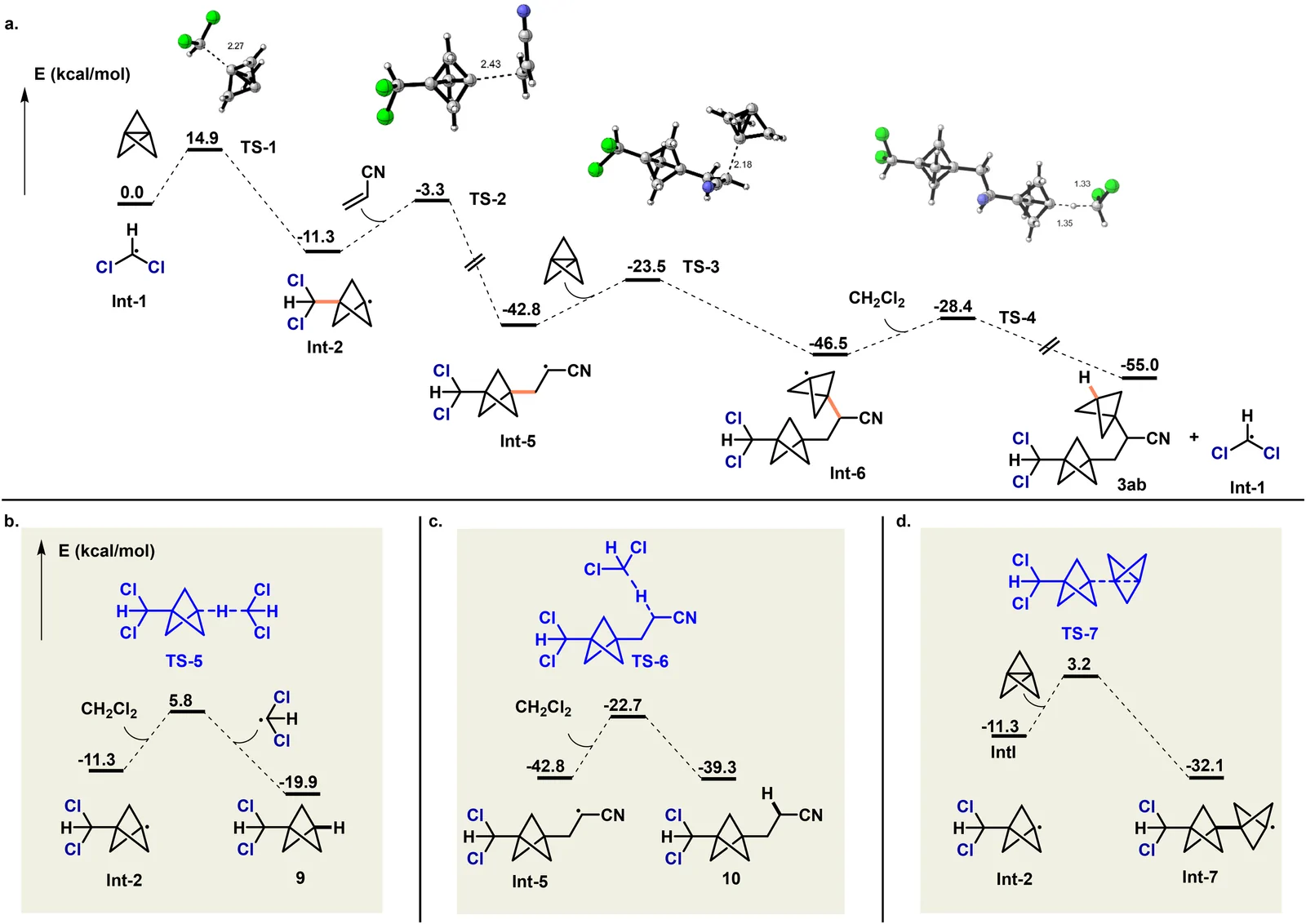
DFT studies of proposed mechanisms and potential side reactions. (a) Main energy pathway. (b) Energy pathway for Int-2 HAT capping. (c) Energy pathway for Int-5 HAT capping. (d) Energy pathway for Int-2 reacting with an additional propellane molecule. Calculations performed using (DLPNO-CCSD(T)^29^/ma-def2-QZVPP^30^//M062X^31^-d3^32^/def2-TZVP^33^ (SMD : CH_2_Cl_2_),^34^ ORCA 6.0^35^//Gaussian 16).^36^

To further explore the mechanism and explain the selectivity of the MCRs, calculations were carried out using acrylonitrile as a radical acceptor (Fig. 6a, see the SI for further details). First, we wanted to provide support that the proposed mechanism is energetically feasible under the standard experimental conditions. Assuming the formation of Int-1, we computed the transition state (TS) associated with radical addition to [1.1.1]propellane 1. We found an energy of activation of 14.9 kcal mol^−1^ for TS-1, with Int-2 having a potential energy of −11.3 kcal mol^−1^, explained by the strain release. Radical addition to acrylonitrile needs 8.0 kcal mol^−1^ for TS-2 and Int-5 lies at −42.8 kcal mol^−1^. Another equivalent of 1 then reacts with an activation energy of 19.3 kcal mol^−1^ and Int-6 is formed at −46.5 kcal mol^−1^. TS-4 corresponds to the HAT between Int-6 and CH_2_Cl_2_, requiring 18.1 kcal mol^−1^, yielding the product (3ab) and regenerating the initial radical Int-1. The reaction was found to be exothermic by −55.0 kcal mol^−1^. Also, all energies (maximum at 14.9 kcal mol^−1^ with TS-1) associated with the different steps are feasible under our experimental conditions. In parallel, we computed different potential side reactions to compare with the proposed main reaction pathway to see which one is favored. The first potential side reaction is the HAT reaction between Int-2 and CH_2_Cl_2_, which might result in chain termination (Fig. 6b). From Int-2, TS-5 was found to have an activation energy of 17.1 kcal mol^−1^, producing product 9 with an energy of −19.9 kcal mol^−1^. While this reaction is feasible, this is still unfavored compared to TS-2 by a difference of 9.1 kcal mol^−1^. A second possible side reaction is the HAT transformation with Int-5 and CH_2_Cl_2_ (TS-6) to yield compound 10 (Fig. 6c). This step is also not favorable, with an activation energy of 20.1 kcal mol^−1^, which is 0.8 kcal mol^−1^ higher than TS-3. While this energy difference is at the limit of the model used for our calculations, these side products were not detected. The side reaction investigated is the radical addition of Int-2 to 1 (TS-7), which had an activation energy of 14.5 kcal mol^−1^, which is unfavored compared to TS-2 by 6.5 kcal mol^−1^ (Fig. 6d). Side products from the polymerization of 1 were also not detected experimentally.

## Conclusions

In summary, we have demonstrated three different MCRs to generate unique chlorinated- and silylated-BCP and BCH scaffolds. The scope could be extended to a broad range of acrylamides and Michael acceptors, leading to great diversity of products and broadening the applicability of the reaction. Mechanistic studies provided support for a radical process and explained the origin of the HAT step, and in-depth computational studies provided support for the proposed mechanism. We anticipate that our methodology will be immediately impactful to both pharmaceutical chemistry and material science by providing efficient access to a variety of unique halogenated and silylated bioisosteres containing multiple BCP/BCH cores.

## Author contributions

Y. W., M. P., and J. G. W. designed the project. Y. W. and M. P. performed the experiments. Y. W., M. P., S. P. P. and J. G. W. wrote the manuscript. J. G. W. and S. P. P. directed the project. All authors interpreted the results in the manuscript.

## Conflicts of interest

There are no conflicts to declare.

## Supplementary Material

QO-013-D6QO00901H-s001

## Data Availability

All data for experimental procedures and compound characterization are available in the paper and its supplementary information (SI). Supplementary information: experimental procedures, details of reaction optimization, copies of ^1^H and ^13^C NMR spectra (pdf). See DOI: https://doi.org/10.1039/d6qo00901h.
