# Supplementary material for: Light-enabled metal-free carbo- and silyl functionalization of [1.1.1]propellane and [3.1.1]propellane
Source: Org Chem Front. 2026 Aug 11;13(19):6593–602. doi: 10.1039/d6qo00901h (PMC13484456; doi:10.1039/d6qo00901h)
Supplement: Supplementary file 1 [file QO-013-D6QO00901H-s001.pdf]

Supplementary Information (SI) for Organic Chemistry Frontiers.
This journal is © the Partner Organisations 2026
Supplementary Information (SI) for Organic Chemistry Frontiers.
This journal is © the Partner Organisations

Light-enabled metal-free carbo- and silyl functionalization of [1.1.1]propellane and [3.1.1]propellane

Yongchen Wang[1†], Martin Pauze[1-2†], Spencer P. Pitre[2], Julian G. West[1*]
[1]Department of Chemistry, Biosciences Research Collaborative, Rice University; Houston, TX 77030, USA.
[2]Department of Chemistry, Oklahoma State University, Stillwater, OK 74078, USA
[†]equal contribution
*Corresponding author. Email: jgwest@rice.edu.

Supplementary Information

Table of contents

1. Experimental. 3

1.1 General Experimental. 3

1.2 Concentration calculation of [1.1.1]propellane and [3.1.1]propellane and Procedures for selective acrylamides synthesis. 5

2. Optimization of MCRs for direct C-C bond and C-Si bond construction. 11

2.1 Optimization of CH_2Cl_2 concentration for dichloromethylation MCR (Table S1). 11

2.2 Optimization of equivalents of 1 for dichloromethylation MCR (Table S2). 12

2.3 Optimization of light wavelength for dichloromethylation MCR (Table S3). 13

2.4 Optimization of equivalents of 1 and reaction time for dichloromethylation MCR under 370 nm irradiation (Table S4). 14

2.5 Optimization of conditions for silylation MCR (Table S5). 15

3. General procedures for the MCRs and characteristic data of product. 16

3.1 General procedure A (dichloromethylation MCR). 16

3.2 General procedure B (trichloromethylation MCR). 16

3.3 General procedure C (silylation MCR). 16

3.4 Characterization of MCR product. 17

4. Mechanistic Study 55

5. NMR spectra of novel compounds. 65

6. References. 137

7. 3D Coordinates of optimized structures, (thermal correction) and energy associated (Hartree) from DLPNO-CCSD(T), charge and multiplicity. 138

1. Experimental.

1.1 General Experimental.

Regents, solvents and reaction conditions

All reactions were performed in dry glassware under a nitrogen atmosphere unless otherwise stated. Solvents and commercially available reagents were dried and purified before use where appropriate using standard procedures. Dichloromethane, diethyl ether (Et_2O), tetrahydrofuran (THF), toluene, acetonitrile (MeCN) were obtained anhydrous from solvent dispenser units having been passed through an activated alumina column under nitrogen. Anhydrous methanol (MeOH) was obtained commercially and used directly without further purification. Dichloromethane (anhydrous, ≥99.8%, contains 40-150 ppm amylene as stabilizer) purchased from sigma-aldrich was used for MCRs. (Note: fresh distilled dichloromethane was also tested for MCRs and gave similar yield of corresponding product) Chloroform (99.9%, extra dry over molecular sieve, stabilized, acroseal®) used for MCRs (**4aa**, **4a**-**4m**) was obtained from thermo scientific. Chloroform-d_1 from sigma-aldrich and Chloroform-d_1 containing 0.05% V/V TMS from Cambridge Isotope Labs Inc were used for obtaining NMR spectra.

Chromatography methods

Reactions were monitored by thin layer chromatography using F_{254} 0.25 mm precoated silica plates purchased from Silicycle, Quebec, Canada. Visualisation was carried out under UV light (λ = 254 nm) or stains such as vanillin, phosphomolybdic acid (PMA) and potassium permanganate ($KMnO_4$). Flash column chromatography was performed on Silicycle P60 silica gel (40-63 μm, 230-400 mesh particle size) as static phase and under increased pressure.

Instrumental analytical methods

NMR spectra were recorded on a Bruker DRX-600 spectrometers (600 MHz for proton nuclei ^1H and 151 MHz for carbon nuclei ^{13}C). The analysis of the NMR spectra was carried out with MestReNova®. Chemical shifts δ are given in parts per million (ppm) to the nearest 0.01 ppm for ^1H and 0.1 ppm for ^{13}C spectra, with the solvent resonance as internal standard: chloroform-d_1: 7.26 (^1H NMR) and 77.16 (^{13}C NMR). Coupling constants J are reported to the nearest 0.1 Hz. Multiplicities are reported as follows: s = singlet, br s = broad singlet, d = doublet, dd = doublet of doublets etc., t = triplet, q = quartet, sept = septet, m = multiplet. Assignments of compounds were based on two-dimensional NMR (COSY, HSQC, HMBC).

High resolution mass spectra (HRMS) were recorded under ESI conditions on a Agilent UHPLC TOF mass spectrometer using electrospray ionization time-of-flight (ESI-TOF), chemical ionization time-of-flight (CI-TOF) or atmospheric pressure chemical ionization (ACPI). Values are calculated to 4 decimal places from the molecular formula. The parent ion $[M]^+$, $[M+H]^+$, $[M-H]^-$or $[M+COOH]^-$ are quoted.

Gas chromatography– mass spectrometry (GC-MS) were recorded on an Agilent 8860 GC using an Agilent J&W HP-5 (5%-diphenyl)-dimethylpolysiloxane column (30 m x 0.32 mm x 0.25 μm film thickness). Data analysis was completed through OpenLab CDS and MassHunter software.

Computational details

To perform calculations, Gaussian 16 was used. All intermediate and transition states were initially optimized with uWB97XD functional and Def2-TZVPP basis set for all atoms. Then conformational search was performed with GOAT algorithm from Orca 6.0. Lowest conformers were optimized with M062X functional, def2-TZVP as basis set, Grimme dispersion 3 and SMD solvent model with CH_2Cl_2 Frequency calculations were performed at the same level and, respectively, zero or one imaginary frequency was found for intermediate and transition states. Single points were calculated from the optimized structures with Orca 6.0 at DLPNO-CCSD(T) level, with ma-def-QZVPP as basis and dispersion model, SMD solvent model with CH_2Cl_2. Energy from single points were added to the thermal corrections to Gibbs free energy from the frequency's calculations and used to calculate the different energy pathways. Radical and non-radical intermediates and transition states were calculated as neutral, and, respectively, with a multiplicity of 2 or 1. The level of theory was adapted from Zhang's work[1] and Anderson's[2].

CYLview was used to generate the 3D drawing.[3]

Header for Gaussian 16 calculation:

opt freq scrf=(smd,solvent=dichloromethane) geom=connectivity def2tzvp
um062x empiricaldispersion=gd3

Header for Orca 6.0 calculation:

! DLPNO-CCSD(T) ma-def2-QZVPP TightSCF TightPNO AutoAux RIJCOSX SMD(CH2Cl2)

1.2 Concentration calculation of [1.1.1]propellane and [3.1.1]propellane and Procedures for selective acrylamides synthesis.

List of acrylamides and other Michael acceptors for the MCRs.

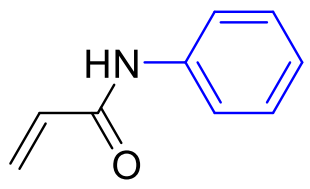

2a

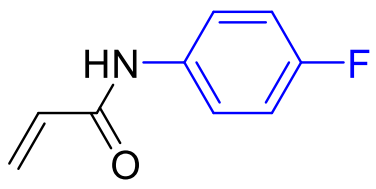

2b

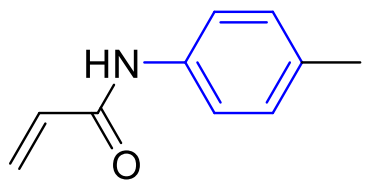

2c

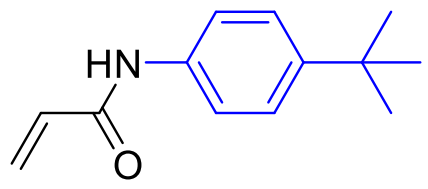

2d

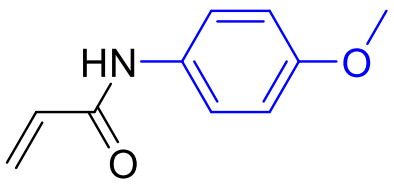

2e

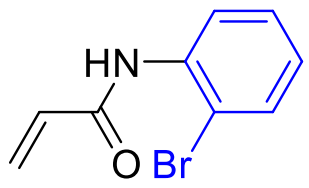

2f

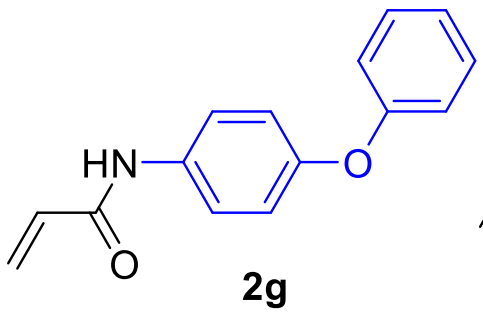

2g

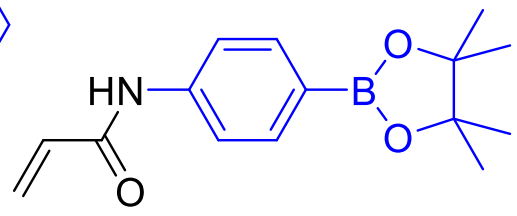

2h

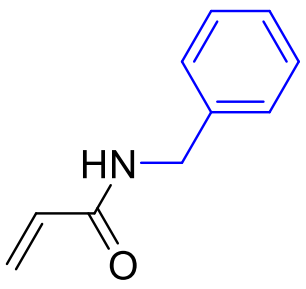

2i

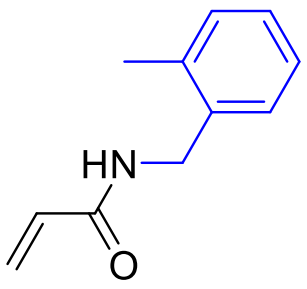

2j

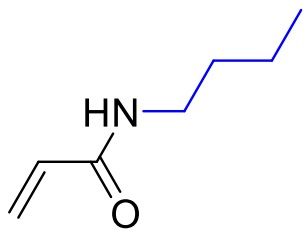

2k

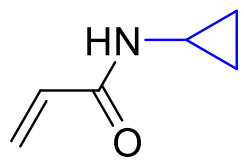

2l

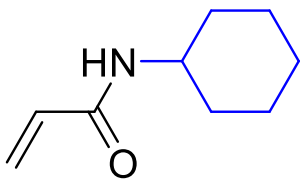

2m

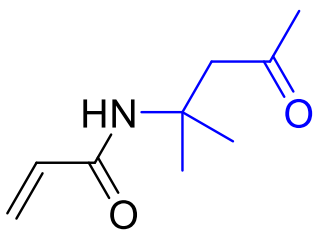

2n

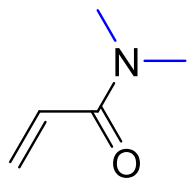

2o

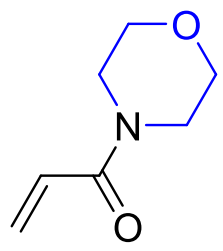

2p

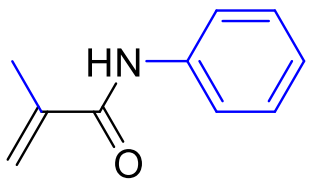

2q

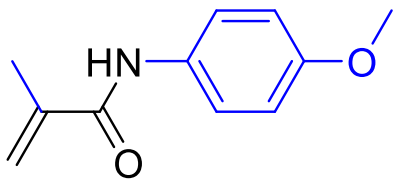

2r

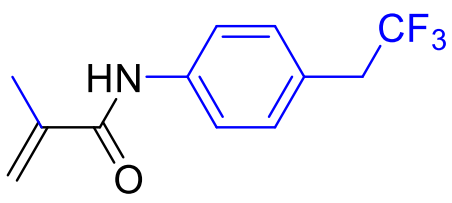

2s

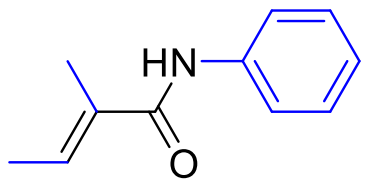

2t

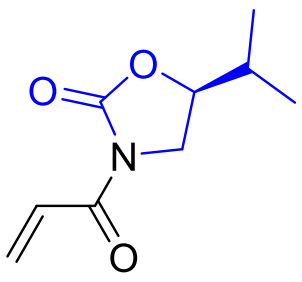

2u

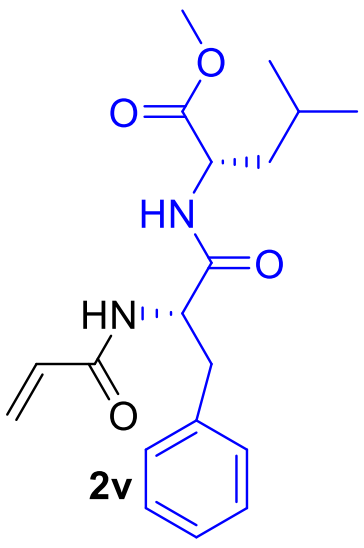

2v

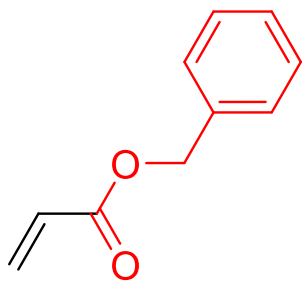

2aa

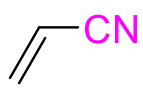

2ab

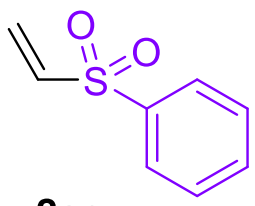

2ac

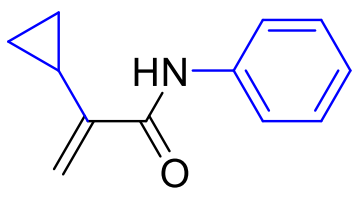

2ad

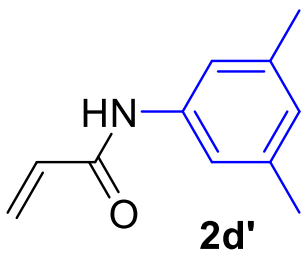

2d'

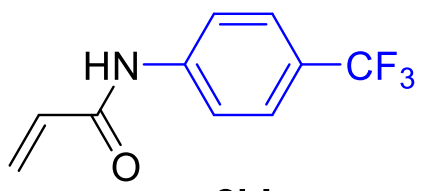

2b'

Less successful and failed substrates

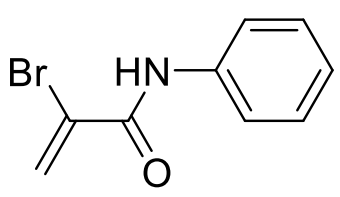

2ae product with inseparable impurities

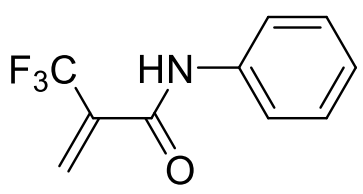

2af trace product (product was seen from gc-mass)

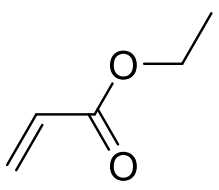

2ag product with inseparable impurities

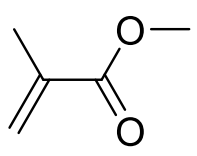

2ah product with inseparable impurities

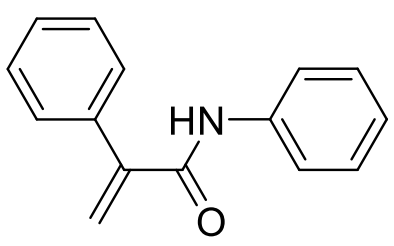

no reaction

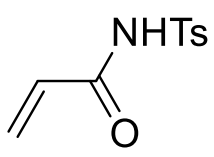

no reaction

Acrylamides **2a**, **2n**, **2o, 2q** and other acceptors **2aa, 2ab, 2ac, 2ag, 2ah** were purchased online and acrylamides (**2b-2m**, **2p, 2r- 2v, 2d', 2b', 2ad, 2ae, 2af**) were synthesized according to reported literature procedures.[4-16] [1.1.1]propellane and [3.1.1]propellane were synthesized according to reported literature procedures.[2,17]

Concentration of [1.1.1]propellane was determined by ^{1}H NMR spectroscopy using a sample of stock solution (200 μL), DCE (50 μL) and $CDCl_3$ in NMR tube.
Calculation of concentration of [1.1.1]propellane batch 1:
2.93 (DCE, 3.65 ppm, 4H) : 1.00 ([1.1.1]propellane, 1.94 ppm, 6H)
(2.93/4) : (1.00/6) = 0.7325 : 0.167
(50 μL x 1.253 g/mL)/98.96 g/mol = 0.63 mmol
(0.63 mmol/(0.7325/0.167))/0.2 mL = 0.72 M

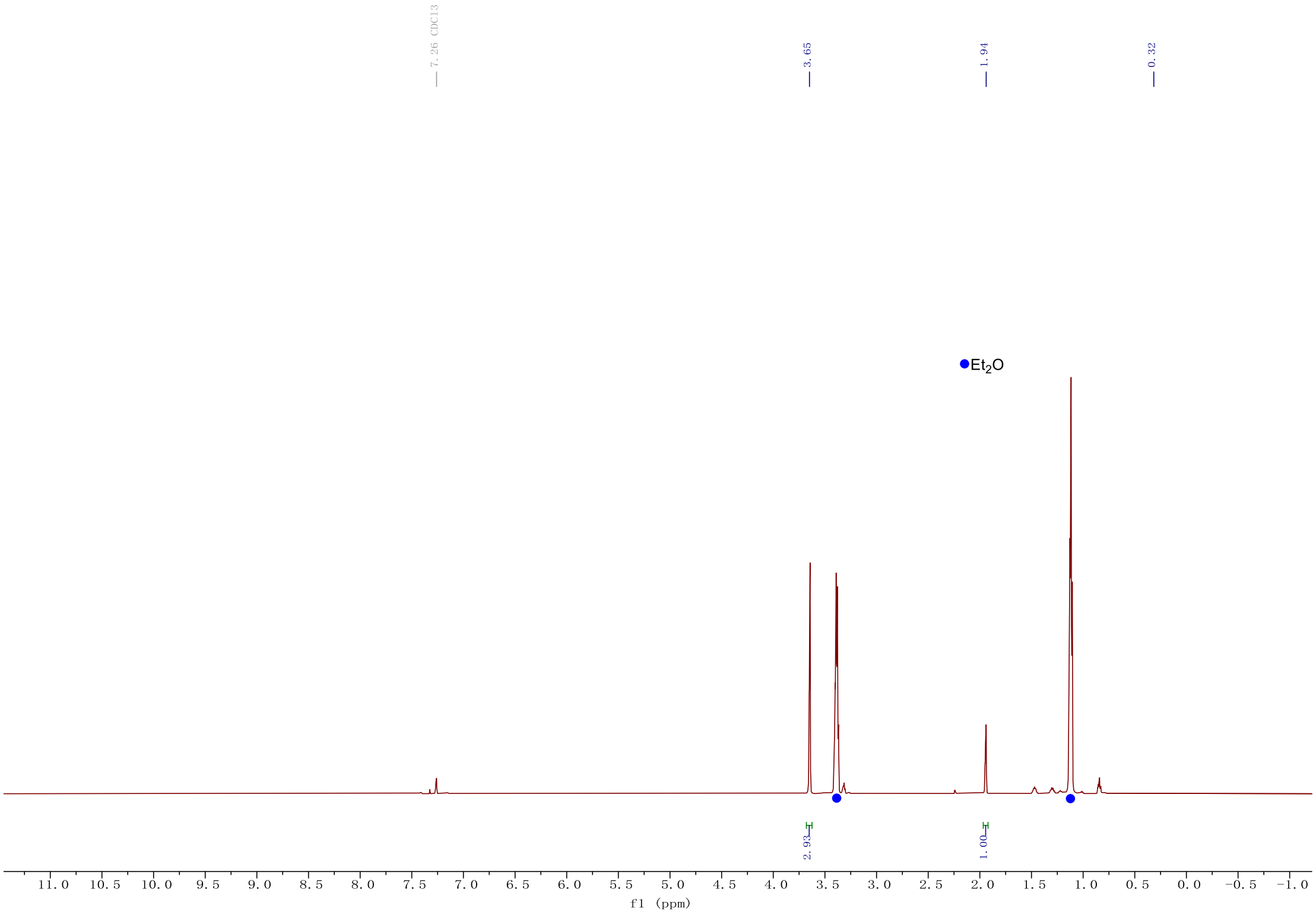

Calculation of concentration of [1.1.1]propellane batch 2:
3.07 (DCE, 3.65 ppm, 4H) : 1.00 ([1.1.1]propellane, 1.94 ppm, 6H)
(3.07/4) : (1.00/6) = 0.7675 : 0.167
(50 μL x 1.253 g/mL)/98.96 g/mol = 0.63 mmol
(0.63 mmol/(0.7675/0.167))/0.2 mL = 0.69 M

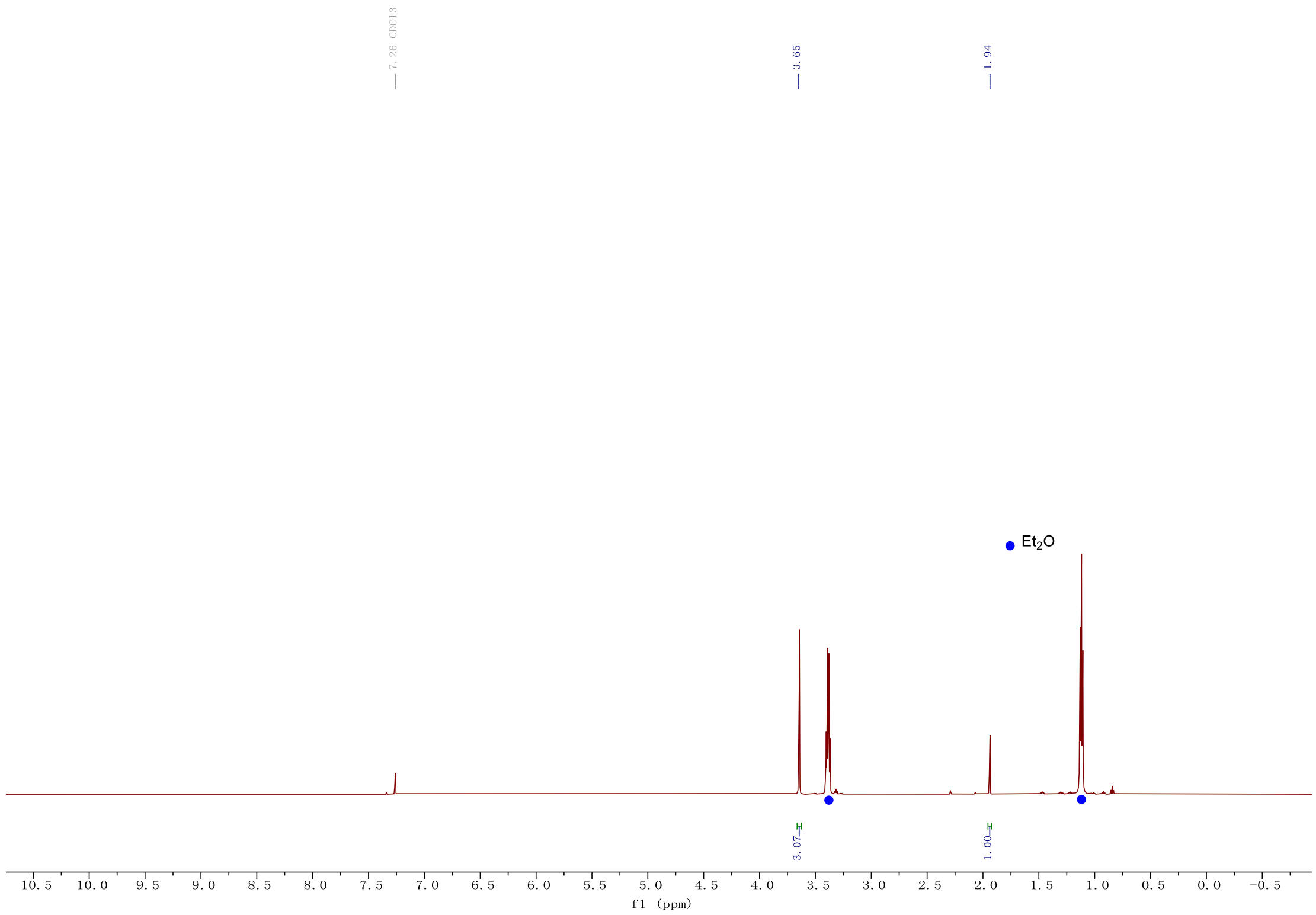

Both [1.1.1]propellane from batch 1 and batch 2 were tested following the general procedure A with acrylamide **2a** and rendered the desired product **3a** in 58% and 54% isolated yield respectively. Et_2O does not affect the reactions.

Concentration of [3.1.1]propellane was determined by ^{1}H NMR spectroscopy using a sample of stock solution (200 μL), DCE (50 μL) and d_6-benzene in NMR tube.
1.00 (DCE, 3.11 ppm, 4H) : 0.14 ([3.1.1]propellane, 2.31-2.21 ppm, 4H) Other signals of [3.1.1]propellane overlap with n-Bu_2O peaks.
(1/4) : (0.14/4) = 0.25 : 0.035
(50 μL x 1.253 g/mL)/98.96 g/mol = 0.63 mmol
(0.63 mmol/(0.25/0.035))/0.2 mL = 0.44 M

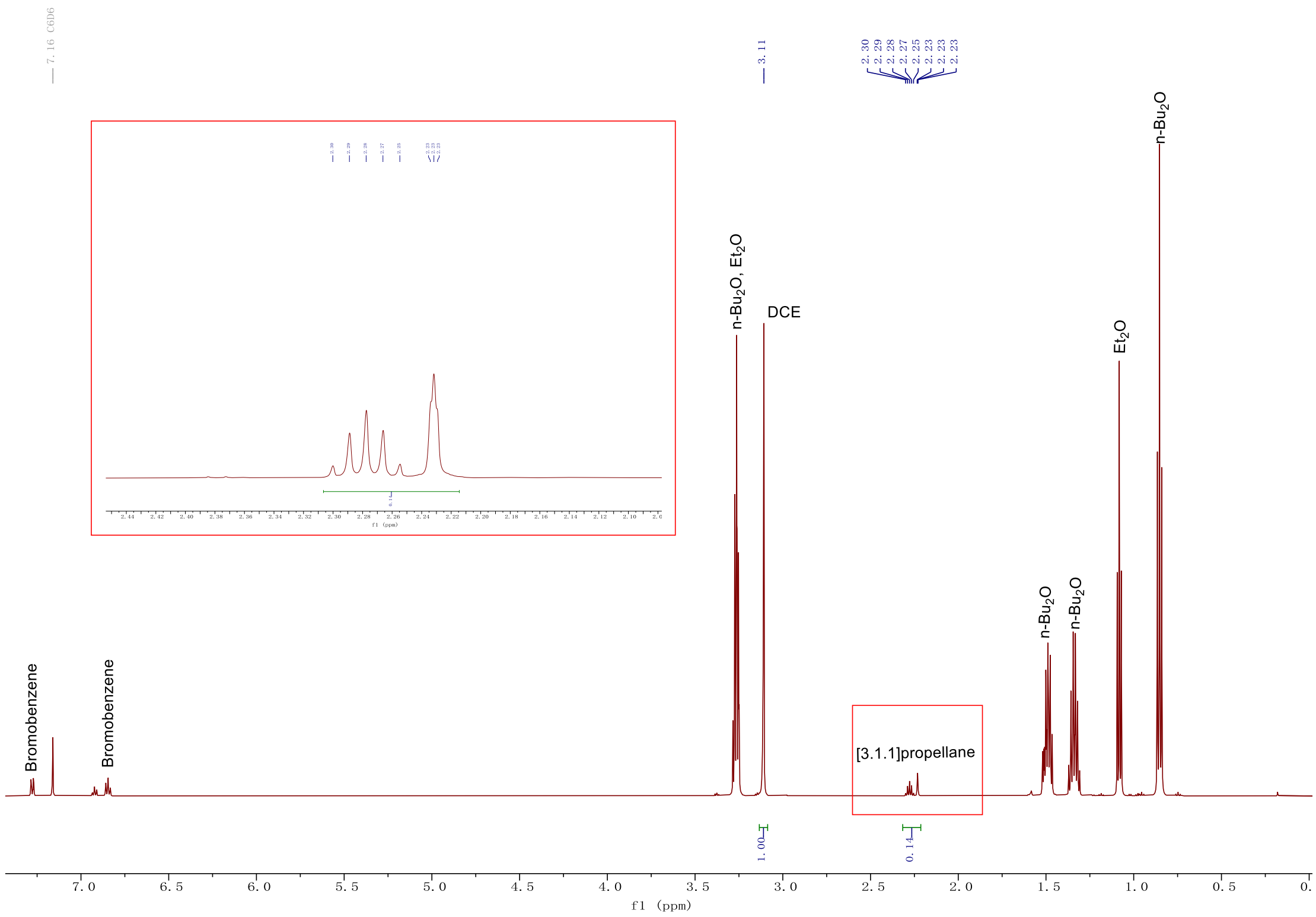

The ^{1}H NMR spectrum matches that previously reported[18]. The resulting [3.1.1]propellane stock solution contains bromobenzene, nBu_2O and Et_2O which do not influence the reactions presented herein.

GC-MS analysis of **2af** reaction mixture (retention time of starting material was at t = 5.89, product was observed at t = 14.60, (EI^+) calc. for $C_{21}H_{22}Cl_2F_3NO$ $[M]^+$ 431.10, found 431.1).

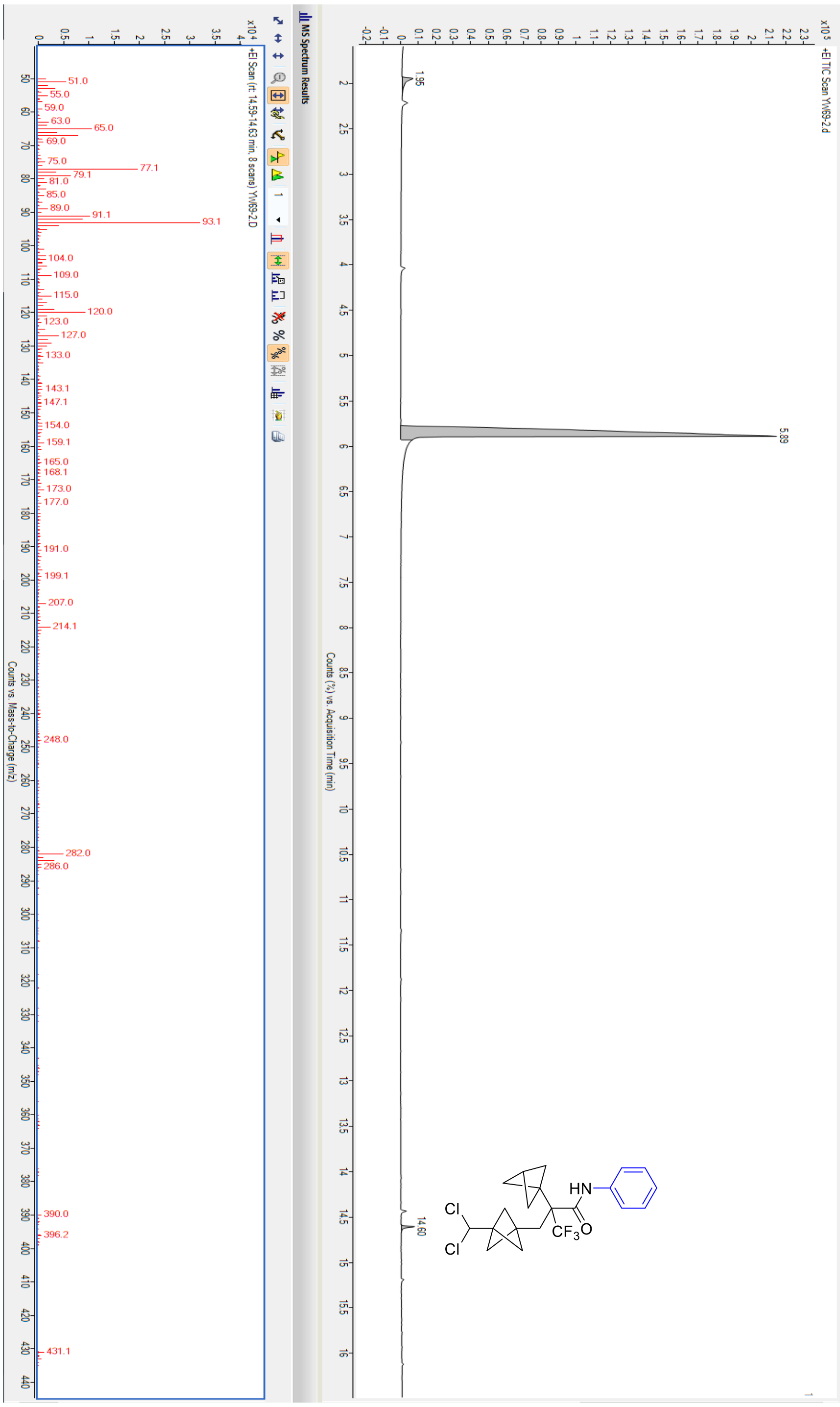

Synthesis of (*S*)-3-acryloyl-5-isopropyloxazolidin-2-one (2u)

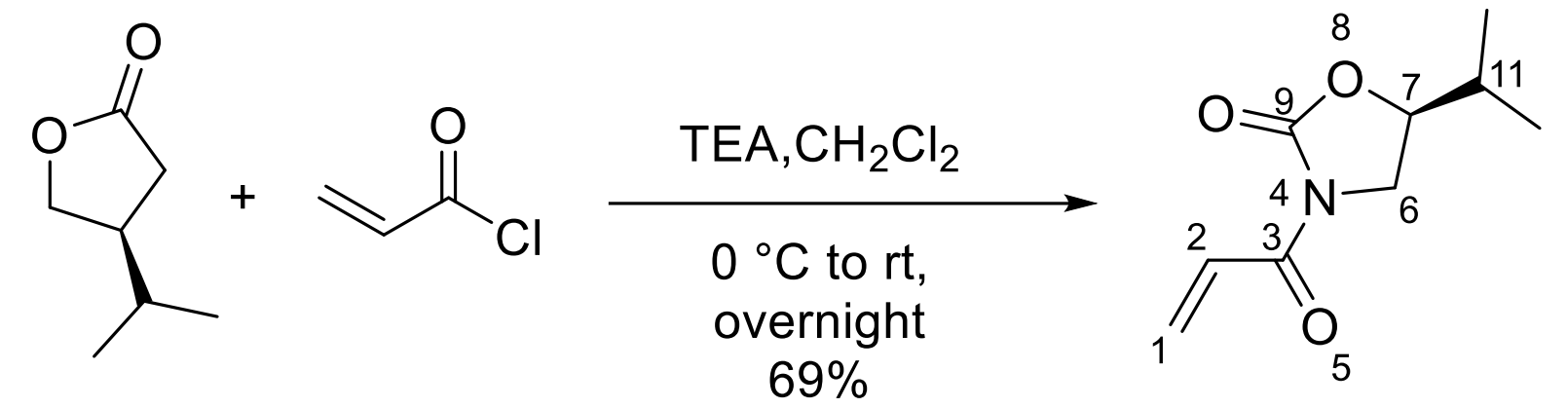

To a solution of (*S*)-4-isopropyldihydrofuran-2(3H)-one (0.5 g, 3.87 mmol, 1.0 equiv.) in CH_2Cl_2 (20 mL) was added triethylamine (0.7 mL, 5.03 mmol, 1.3 equiv.). The reaction mixture was cooled to 0 °C and acyl chloride (0.38 mL, 4.65 mmol, 1.2 equiv.) was added dropwise. After stirring at room temperature overnight, the mixture was concentrated under reduced pressure. The residue was purified by flash column chromatography (10%-30% EtOAc/hexane) to obtain acrylamide **2u** (489 mg, 2.67 mmol, 69%) as a white solid.

R_f 0.25 (20% EtOAc/hexane); **^{1}H NMR** (600 MHz, $CDCl_3$) δ 7.52 (dd, *J* = 17.0, 10.5 Hz, 1H, H2), 6.54 (dd, *J* = 17.0, 1.8 Hz, 1H, H1), 5.89 (dd, *J* = 10.5, 1.8 Hz, 1H, H1'), 4.50 (ddd, *J* = 8.4, 4.0, 3.1 Hz, 1H, H7), 4.30 (dd, *J* = 9.1, 8.3 Hz, 1H, H6), 4.24 (dd, *J* = 9.1, 3.1 Hz, 1H, H6'), 2.43 (heptd, *J* = 7.0, 3.9 Hz, 1H, H11), 0.94 (d, *J* = 7.1 Hz, 3H, CH_3), 0.89 (d, *J* = 6.9 Hz, 3H, CH_3); **^{13}C NMR** (151 MHz, $CDCl_3$) δ 165.1, 154.1, 131.8, 127.6, 63.6, 58.7, 28.5, 18.2, 14.8; **HRMS** (ESI^+) calc. for $C_9H_{13}NO_3$ $[M+H]^+$ 184.0968, found 184.0967. The spectroscopic data corresponded with the literature reported data.[15]

Synthesis of methyl acryloyl-*L*-phenylalanyl-*L*-leucinate (2v)

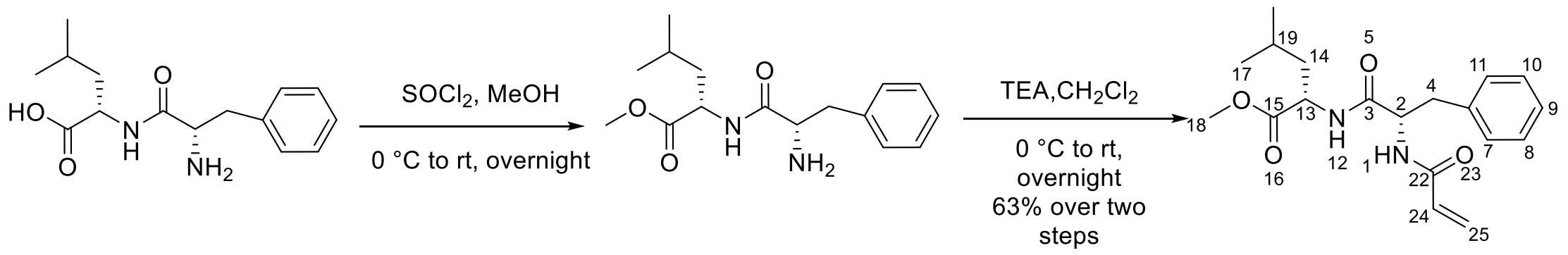

To a solution of *L*-phenylalanyl-*L*-leucine (0.4 g, 1.44 mmol, 1.0 equiv.) in MeOH (8 mL) at 0 °C was added thionyl chloride (0.14 mL, 1.87 mmol, 1.3 equiv.) dropwise. After stirring at room temperature overnight, the mixture was concentrated under reduced pressure. The crude methyl *L*-phenylalanyl-*L*-leucinate (0.41 g, 1.40 mmol) was used directly for next step without further purification

To a solution of methyl *L*-phenylalanyl-*L*-leucinate (0.41 g, 1.40 mmol, 1.0 equiv.) in CH_2Cl_2 (7.0 mL) was added triethylamine (0.25 mL, 1.82 mmol, 1.3 equiv.). The reaction mixture was cooled to 0 °C and a solution of acyl chloride (0.13 mL, 1.54 mmol, 1.1 equiv.) in CH_2Cl_2 (7.0 mL) was added dropwise. After stirring at room temperature overnight, the mixture was concentrated under reduced pressure. The residue was purified by flash column chromatography (10%-40% EtOAc/hexane) to obtain acrylamide **2v** (314 mg, 0.91 mmol, 63%) as a white solid.

R_f 0.25 (35% EtOAc/hexane); **^{1}H NMR** (600 MHz, $CDCl_3$) δ 7.31 – 7.27 (m, 2H, ArH), 7.23 (ddd, J = 7.1, 4.9, 2.8 Hz, 3H, ArH), 6.38 (s, 1H, exchangeable NH), 6.30-6.23 (m, 2H, exchangeable NH and H24), 6.09 (dd, J = 17.0, 10.3 Hz, 1H, H25), 5.66 (dd, J = 10.3, 1.3 Hz, 1H, H25'), 4.82 – 4.72 (m, 1H, H2), 4.51 (ddd, J = 8.9, 7.9, 5.4 Hz, 1H, H13), 3.70 (s, 3H, OCH_3), 3.14 (dd, J = 13.9, 6.1 Hz, 1H, H4), 3.08 (dd, J = 13.9, 7.6 Hz, 1H, H4'), 1.61 – 1.42 (m, 3H, H14 and H19), 0.88-0.86 (m, 6H, CH_3); **^{13}C NMR** (151 MHz, $CDCl_3$) δ 172.8, 170.6, 165.3, 136.5, 130.4, 129.5, 128.8, 127.4, 127.2, 54.4, 52.4, 51.1, 41.5, 38.5, 24.9, 22.8, 22.0; **HRMS** (ESI$^+$) calc. for $C_{19}H_{26}N_2O_4$ $[M+Na]^+$ 369.1785, found 369.1784.

2. Optimization of MCRs for direct C-C bond and C-Si bond construction.

2.1 Optimization of CH_2Cl_2 concentration for dichloromethylation MCR (Table S1).

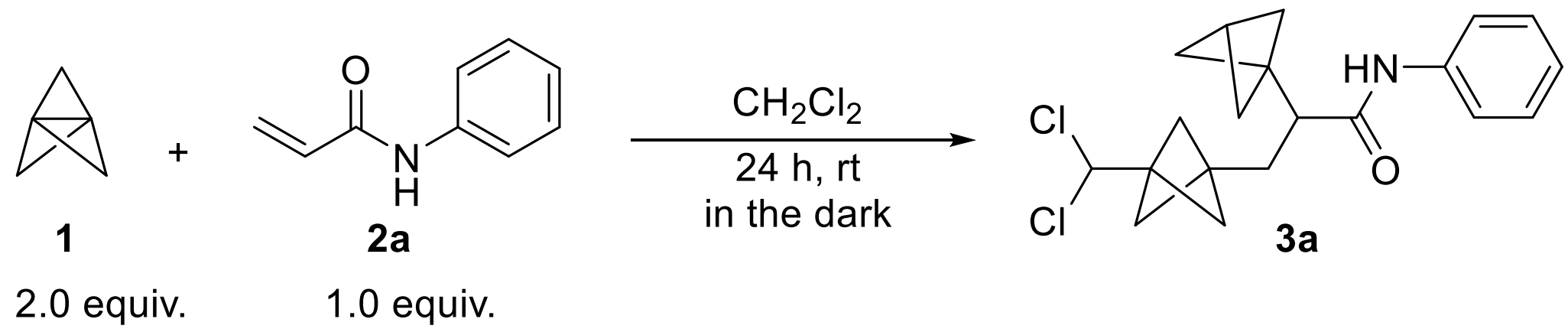

Entry	Concentration of CH_2Cl_2	Yield (%)
1	0.05 M	17
2	0.1 M	21
3	0.2 M	15
4	0.4 M	10

2.2 Optimization of equivalents of 1 for dichloromethylation MCR (Table S2).

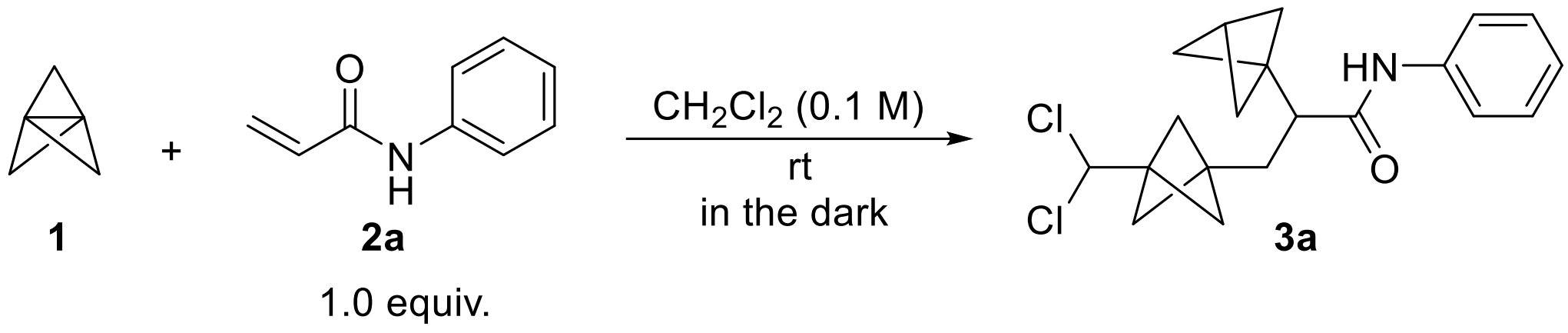

Entry	Equivalence of **1** and reaction time	Yield (%)
1	1.0, 24 h	11
2	2.0, 24 h	21
3	3.0, 24 h	22
4	4.0, 24 h	20
5	5.0, 24 h	15
6	2.0, 48 h	27
7	3.0, 48 h	27

2.3 Optimization of light wavelength for dichloromethylation MCR (Table S3).

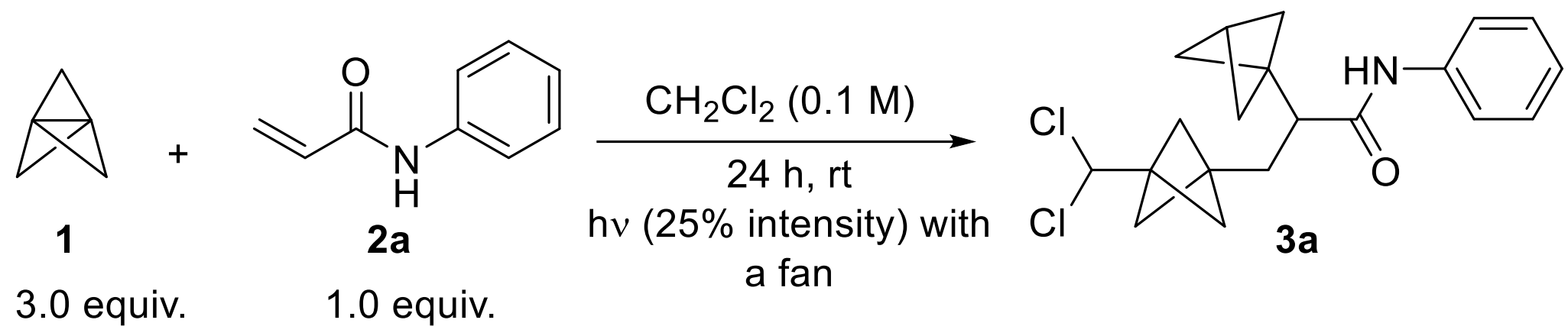

Entry	Wavelength of light	Yield (%)
1	370 nm	40
2	390 nm	25
3	427 nm	23
4	525 nm	22

2.4 Optimization of equivalents of 1 and reaction time for dichloromethylation MCR under 370 nm irradiation (Table S4).

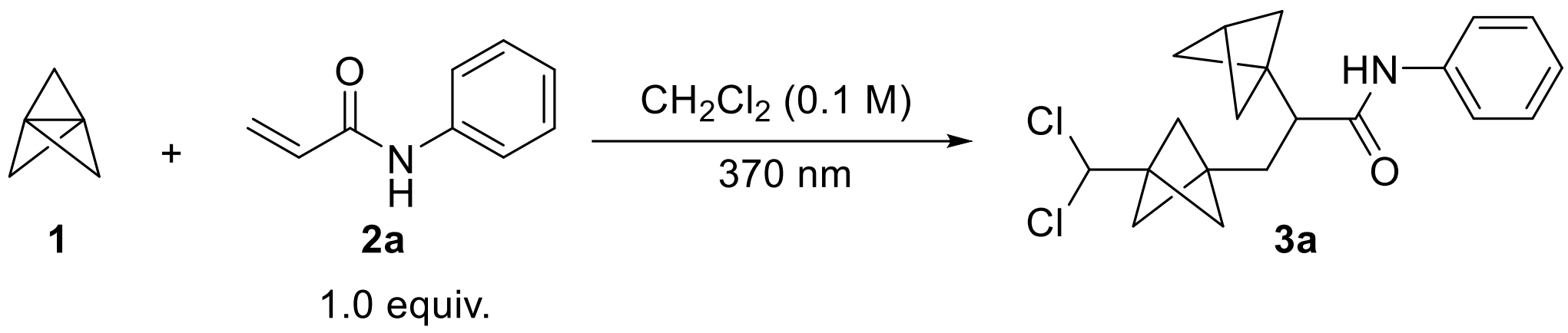

Entry	Equivalence of **1** and reaction conditions	Yield (%)
1	2.0, 24 h, 25% light intensity and a fan	58
2	2.0, 48 h, 25% light intensity and a fan	38
3	3.0, 24 h, 25% light intensity and a fan	40
4	4.0, 24 h, 25% light intensity and a fan	36
5	2.0, 24 h, 100% light intensity and a fan	38
6	2.0, 24h, 100% light intensity without a fan	25

2.5 Optimization of conditions for silylation MCR (Table S5).

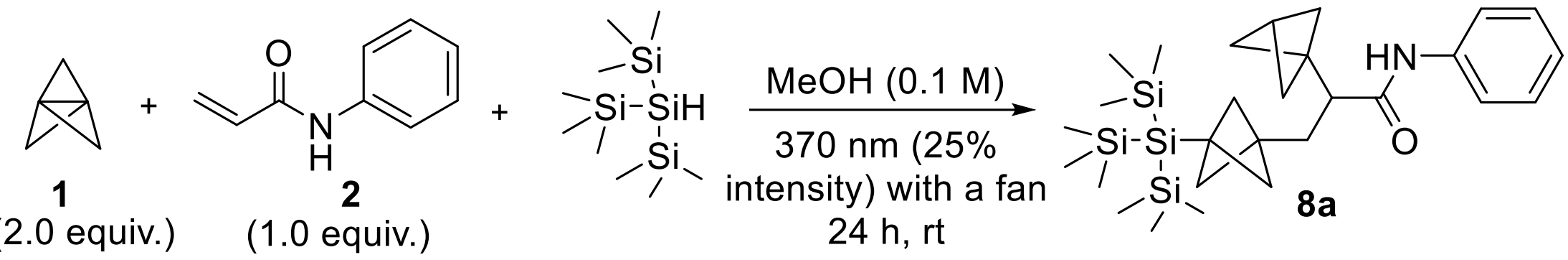

Entry	Equivalence of **TTMS** and reaction conditions	Yield (%)
1	1.0, in the dark	29
2	2.0, in the dark	34
3	2.0, in the dark, CH_2Cl_2 (0.1 M) instead of MeOH	27
4	1.0	42
5	2.0	51
6	3.0	68
7	4.0	56
8	5.0	53

3. General procedures for the MCRs and characteristic data of product.

3.1 General procedure A (dichloromethylation MCR).

To a solution of acrylamides or other acceptors (0.1 mmol, 1.0 equiv.) in CH_2Cl_2 (1.0 mL) under N_2 atmosphere was added [1.1.1]propellane **1** (0.2 mmol, 2.0 equiv.) or [3.1.1]propellane **5** (0.2 mmol, 2.0 equiv.). The reaction mixture was placed under 370 nm Kessil® LED (25% intensity) with a cooling fan after sealing the punctured holes of the vial cap with vacuum grease and electric tape/parafilm for better air-tight protection. After stirring at room temperature for 24 h, the reaction mixture was concentrated under reduced pressure. The crude residue was purified by flash column chromatography (Et_2O/hexane) to give corresponding product of the MCR.

3.2 General procedure B (trichloromethylation MCR).

To a solution of acrylamides (0.1 mmol, 1.0 equiv.) in $CHCl_3$ (1.0 mL) under N_2 atmosphere was added [1.1.1]propellane **1** (0.2 mmol, 2.0 equiv.) or [3.1.1]propellane **5** (0.2 mmol, 2.0 equiv.). The reaction mixture was placed under 370 nm Kessil® LED (25% intensity) with a cooling fan after sealing the punctured holes of the vial cap with vacuum grease and electric tape/parafilm for better air-tight protection. After stirring at room temperature for 24 h, the reaction mixture was concentrated under reduced pressure. The crude residue was purified by flash column chromatography (Et_2O/hexane) to give corresponding product of the MCR.

3.3 General procedure C (silylation MCR).

To a solution of acrylamides (0.1 mmol, 1.0 equiv.) in MeOH (1.0 mL) under N_2 atmosphere was added tris(trimethylsilyl)silane (TTMS) (0.3 mmol, 3.0 equiv.) and [1.1.1]propellane **1** (0.2 mmol, 2.0 equiv.) or [3.1.1]propellane **5** (0.2 mmol, 2.0 equiv.). The reaction mixture was placed under 370 nm Kessil® LED (25% intensity) with a cooling fan after sealing the punctured holes of the vial cap with vacuum grease and electric tape/parafilm for better air-tight protection. After stirring at room temperature for 24 h, the reaction mixture was concentrated under reduced pressure. The crude residue was purified by flash column chromatography (Et_2O/hexane) to give corresponding product of the MCR.

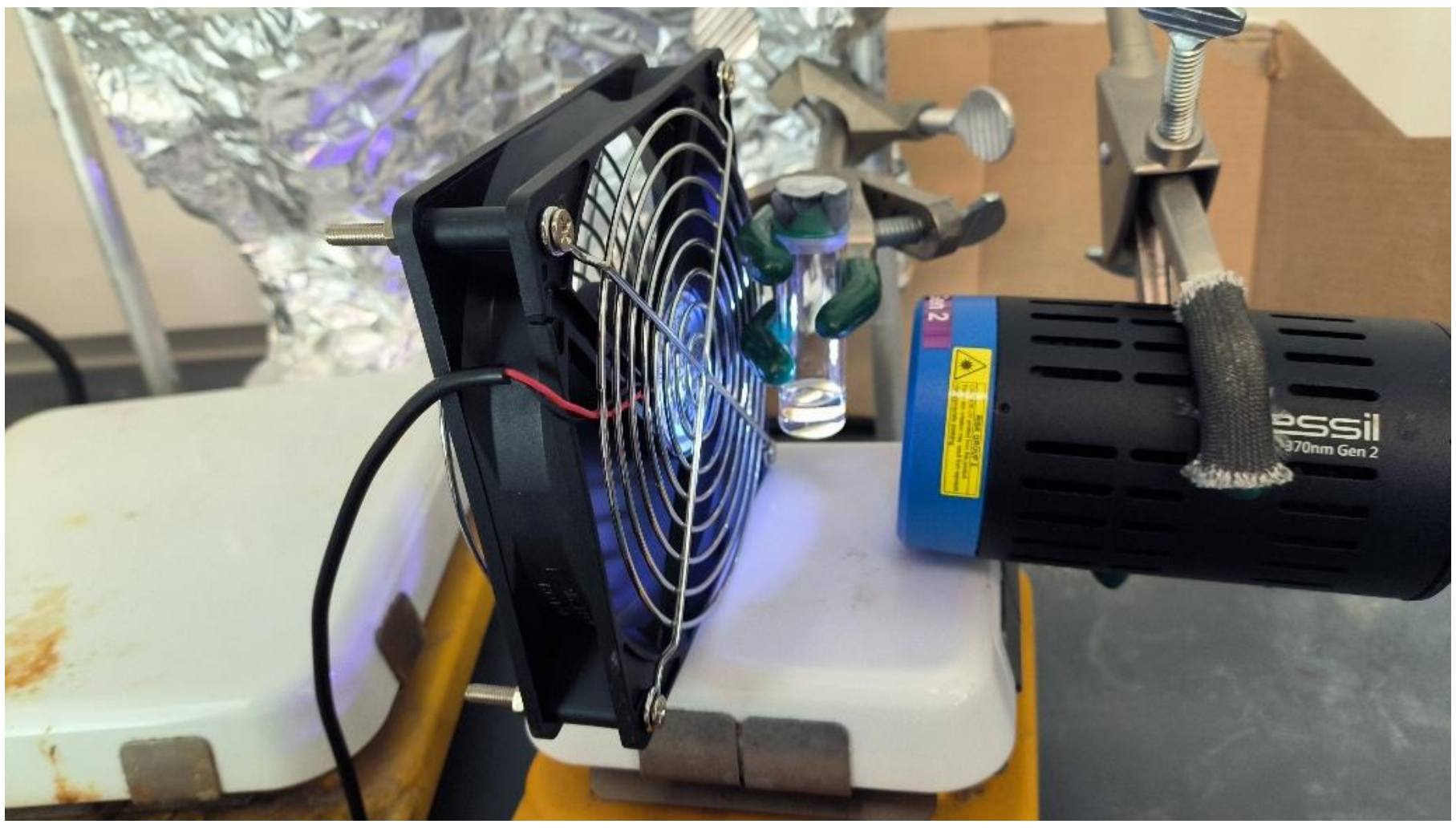

3.4 Characterization of MCR product.

2-(bicyclo[1.1.1]pentan-1-yl)-3-(3-(dichloromethyl)bicyclo[1.1.1]pentan-1-yl)-*N*-phenylpropanamide (3a)

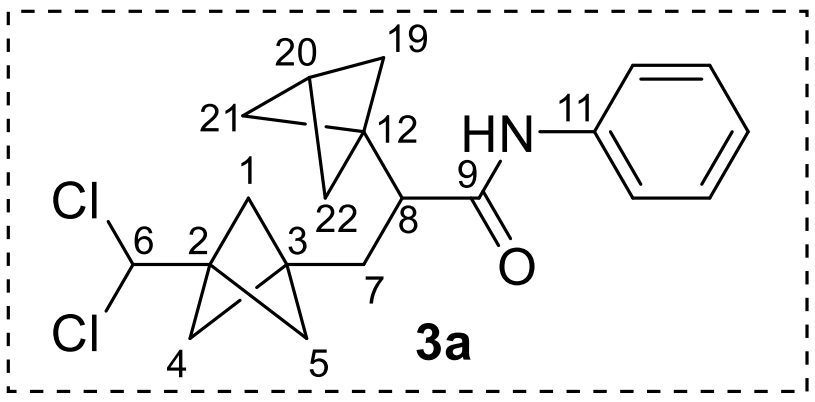

Synthesized according to *General Procedure A* using acrylamide **2a** (14.7 mg, 0.1 mmol, 1.0 equiv.) and **1** (0.28 mL, 0.72 M in Et_2O solution, 0.2 mmol, 2.0 equiv.). Purification is through flash column chromatography (0-20% Et_2O/Hex) to give product **3a** (21.1 mg, 0.058 mmol, 58%) as a white solid.

R*f* 0.28 (20% Et_2O/hexane); **^{1}H NMR** (600 MHz, $CDCl_3$) δ 7.52–7.47 (m, 2H, ArH), 7.35–7.30 (m, 2H, ArH), 7.12 (ddt, *J* = 8.6, 7.4, 1.2 Hz, 1H, ArH), 7.02 (s, 1H, exchangeable NH), 5.70 (s, 1H, H6), 2.51 (s, 1H, H20), 2.33 (dd, *J* = 11.6, 2.9 Hz, 1H, H8), 2.09 (dd, *J* = 14.4, 11.6 Hz, 1H, H7), 1.77–1.67 (m, 12H, H1,H4,H5,H19,H21,H22), 1.61 (dd, *J* = 14.4, 2.9 Hz, 1H, H7'); **^{13}C NMR** (151 MHz, $CDCl_3$) δ 170.6, 137.9, 129.2, 124.5, 120.1, 72.0, 49.5, 49.1, 48.6, 45.6, 44.4, 37.7, 30.2, 26.9; **HRMS** (ESI$^+$) calc. for $C_{20}H_{23}Cl_2NO$ $[M+H]^+$364.1229, found 364.1230.

TLC analysis for reaction mixture of **3a** is as followings (left: PMA stain, right: vanillin stain):

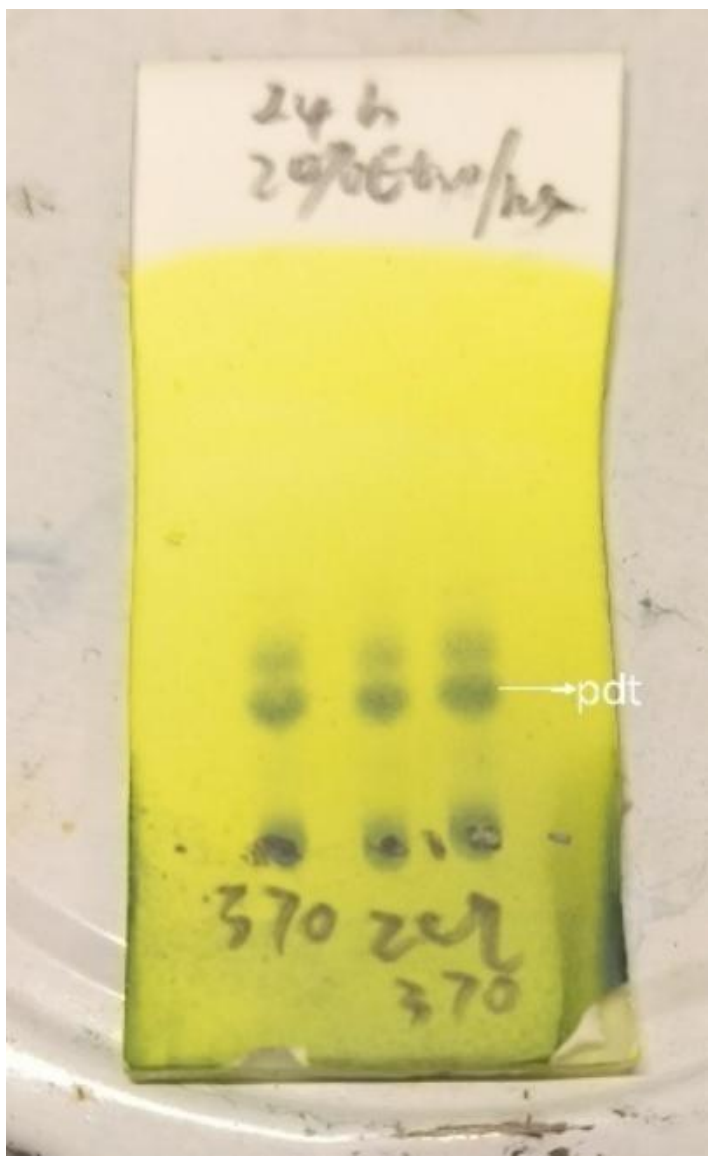

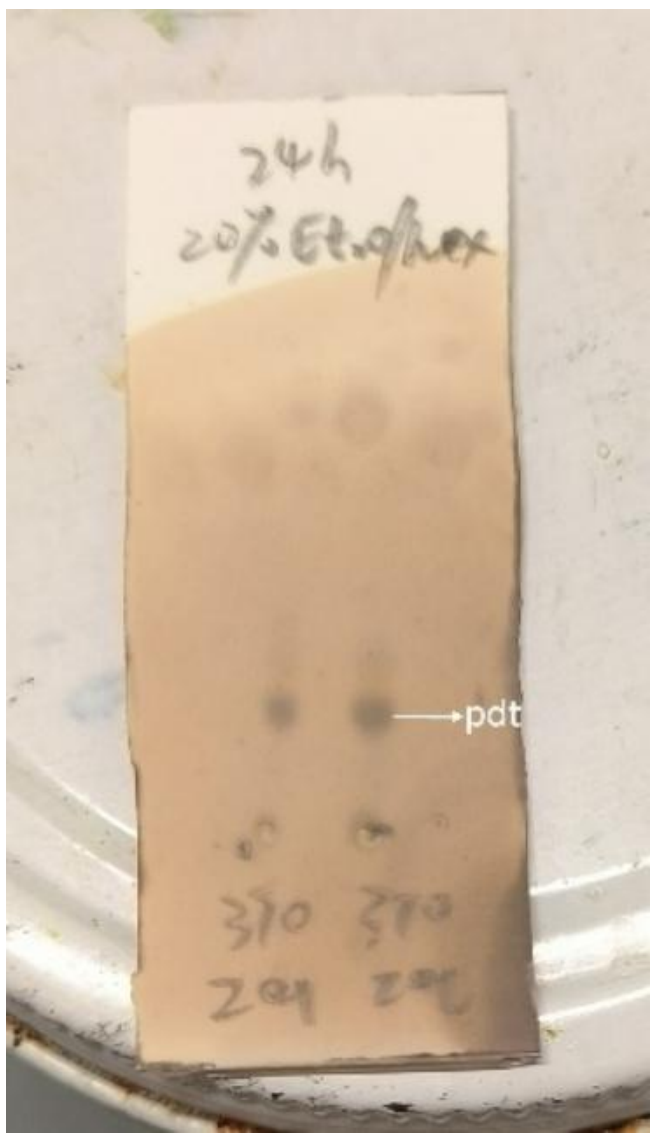

2-([1,1'-bi(bicyclo[1.1.1]pentan)]-3-yl)-3-(3-(dichloromethyl)bicyclo[1.1.1]pentan-1-yl)-*N*-phenylpropanamide (3a', side product) (top spot in TLC).

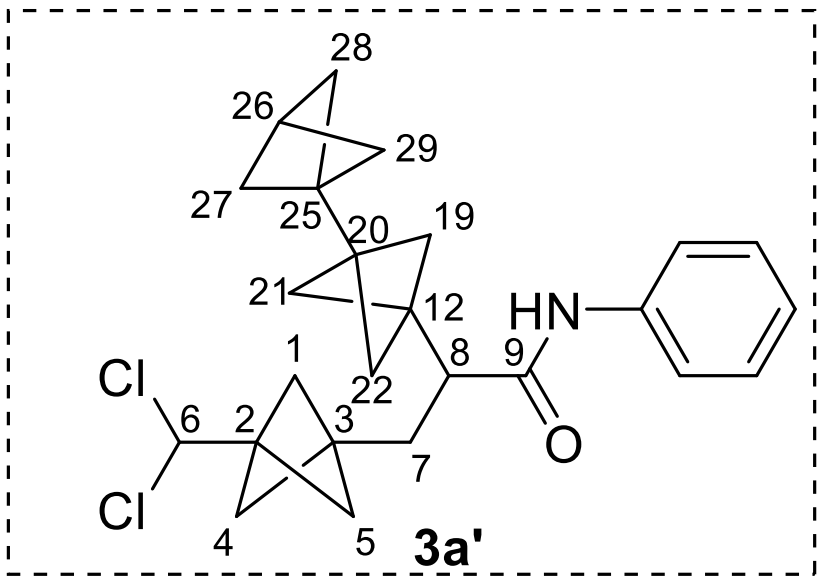

3a'

***R**f* 0.31 (20% Et_2O/hexane); **^{1}H NMR** (600 MHz, $CDCl_3$) δ 7.52-7.48 (m, 2H, ArH), 7.35–7.30 (m, 2H, ArH), 7.12 (tt, *J* = 7.4, 1.2 Hz, 1H, ArH), 6.95 (s, 1H, exchangeable NH), 5.71 (s, 1H, H6), 2.50 (s, 1H, H26), 2.30 (dd, *J* = 11.6, 2.9 Hz, 1H, H8), 1.96 (dd, *J* = 14.4, 11.6 Hz, 1H, H7), 1.76–1.67 (m, 7H, H1,H4,H5 and H7'), 1.61 (s, 6H, H19,H21,H22), 1.53–1.44 (m, 6H, H27,H28,H29); **^{13}C NMR** (151 MHz, $CDCl_3$) δ 170.9, 138.0, 129.2, 124.4, 120.0, 72.5, 49.6, 49.5, 48.9, 47.7, 45.7, 43.6, 38.6, 38.0, 37.8, 31.0, 26.8; **HRMS** (ESI^+) calc. for $C_{25}H_{29}Cl_2NO$ $[M+H]^+$430.1699, found 430.1695.

2-(bicyclo[1.1.1]pentan-1-yl)-3-(3-(dichloromethyl)bicyclo[1.1.1]pentan-1-yl)-*N*-(4-fluorophenyl)propenamide (3b)

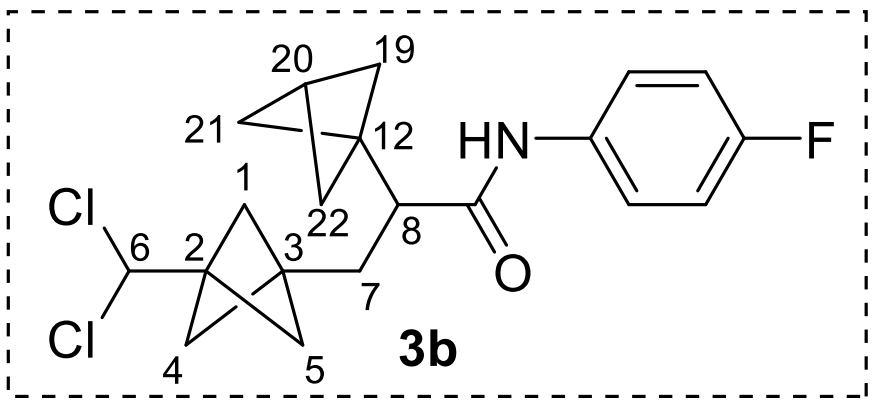

3b

Synthesized according to *General Procedure A* using acrylamide **2b** (16.5 mg, 0.1 mmol, 1.0 equiv.) and **1** (0.28 mL, 0.72 M in Et_2O solution, 0.2 mmol, 2.0 equiv.). Purification is through flash column chromatography (0-25% Et_2O/Hex) to give product **3b** (17.0 mg, 0.045 mmol, 45%) as a white solid.

***R**f* 0.20 (20% Et_2O/hexane);**^{1}H NMR** (600 MHz, $CDCl_3$) δ 7.47–7.39 (m, 2H, ArH), 7.08–6.93 (m, 3H, ArH and exchangeable NH), 5.71 (s, 1H, H6), 2.52 (s, 1H, H20), 2.31 (dd, *J* = 11.6, 2.9 Hz, 1H, H8), 2.08 (dd, *J* = 14.4, 11.6 Hz, 1H, H7), 1.78–1.66 (m, 12H, H1,H4,H5,H19,H21,H22), 1.64–1.60 (m, 1H, H7' overlap with water peak); **^{13}C NMR** (151 MHz, $CDCl_3$) δ 170.9, 159.8 (d, *J* = 243.7 Hz), 134.0 (d, *J* = 2.8 Hz), 122.2 (d, *J* = 7.8 Hz), 116.1 (d, *J* = 22.6 Hz). 72.2, 49.7, 49.3, 48.7, 45.8, 44.6, 37.9, 30.3, 27.1; **HRMS** (ESI^+) calc. for $C_{20}H_{22}Cl_2FNO$ $[M-H]^-$380.0979, found 380.0993; $[M+COOH]^-$426.1034, found 426.1050.

2-(bicyclo[1.1.1]pentan-1-yl)-3-(3-(dichloromethyl)bicyclo[1.1.1]pentan-1-yl)-*N*-(p-tolyl)propenamide (3c)

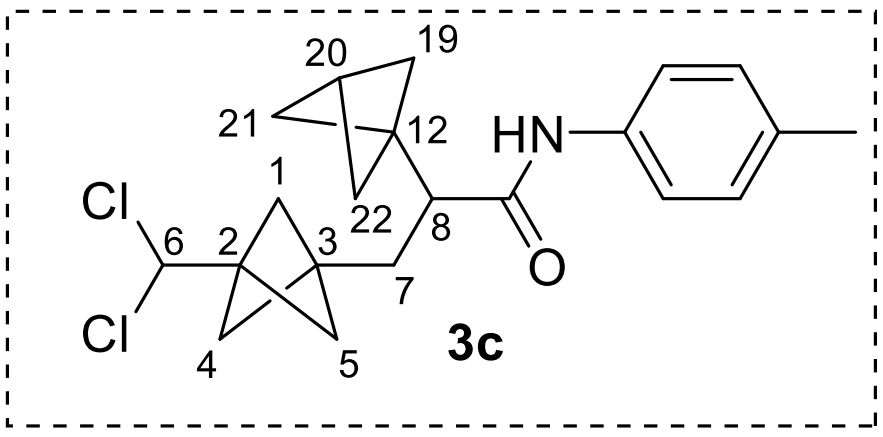

Synthesized according to *General Procedure A* using acrylamide **2c** (16.1 mg, 0.1 mmol, 1.0 equiv.) and **1** (0.2 mmol, 2.0 equiv.). Purification is through flash column chromatography (0-20% Et_2O/Hex) to give product **3c** (17.0 mg, 0.045 mmol, 45%) as a white solid.

R*f* 0.29 (20% Et_2O/hexane);**^{1}H NMR** (600 MHz, $CDCl_3$) δ 7.40–7.34 (m, 2H, ArH), 7.13 (d, *J* = 8.1 Hz, 2H, ArH), 6.97 (s, 1H, exchangeable NH), 5.70 (s, 1H, H6), 2.50 (s, 1H, H20), 2.33-2.28 (m, 4H, CH_3 and H8), 2.08 (dd, *J* = 14.3, 11.6 Hz, 1H, H7), 1.77–1.66 (m, 12H, H1,H4,H5,H19,H21,H22), 1.63–1.56(m, 1H, H7' overlap with water peak); **^{13}C NMR** (151 MHz, $CDCl_3$) δ 170.5, 135.3, 134.2, 129.7, 120.2, 72.0, 49.5, 49.1, 48.5, 45.6, 44.4, 37.7, 30.2, 26.8, 21.0; **HRMS** (ESI$^+$) calc. for $C_{21}H_{25}Cl_2NO$ $[M+H]^+$378.1386, found 378.1386.

2-(bicyclo[1.1.1]pentan-1-yl)-*N*-(4-(tert-butyl)phenyl)-3-(3-(dichloromethyl)bicyclo[1.1.1]pentan-1-yl)propenamide (3d)

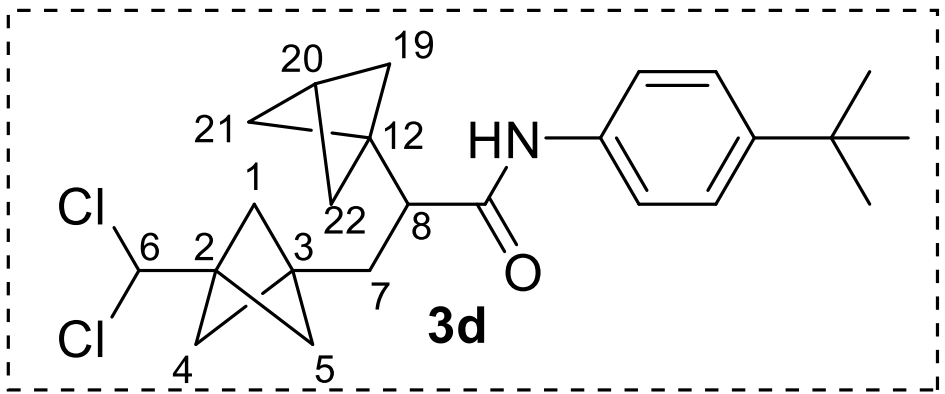

Synthesized according to *General Procedure A* using acrylamide **2d** (20.3 mg, 0.1 mmol, 1.0 equiv.) and **1** (0.2 mmol, 2.0 equiv.). Purification is through flash column chromatography (0-20% Et_2O/Hex) to give product **3d** (18.1 mg, 0.043 mmol, 43%) as a white solid.

R*f* 0.3 (20% Et_2O/hexane);**^{1}H NMR** (600 MHz, $CDCl_3$) δ 7.44–7.40 (m, 2H, ArH), 7.36–7.33 (m, 2H, ArH), 7.03 (s, 1H, exchangeable NH), 5.70 (s, 1H, H6), 2.50 (s, 1H, H20), 2.32 (dd, *J* = 11.6, 2.8 Hz, 1H, H8), 2.09 (dd, *J* = 14.3, 11.6 Hz, 1H, H7), 1.76–1.66 (m, 12H, H1,H4,H5,H19,H21,H22), 1.62–1.56 (m, 1H, H7' overlap with water peak), 1.31 (s, 9H, CH_3); **^{13}C NMR** (151 MHz, $CDCl_3$) δ 170.5, 147.5, 135.3, 126.0, 119.9, 72.0, 49.5, 49.1, 48.5, 45.6, 44.4, 37.7, 34.5, 31.5, 30.2, 26.8; **HRMS** (ESI$^+$) calc. for $C_{24}H_{31}Cl_2NO$ $[M+H]^+$420.1855, found 420.1855.

2-(bicyclo[1.1.1]pentan-1-yl)-3-(3-(dichloromethyl)bicyclo[1.1.1]pentan-1-yl)-*N*-(4-methoxyphenyl)propenamide (3e)

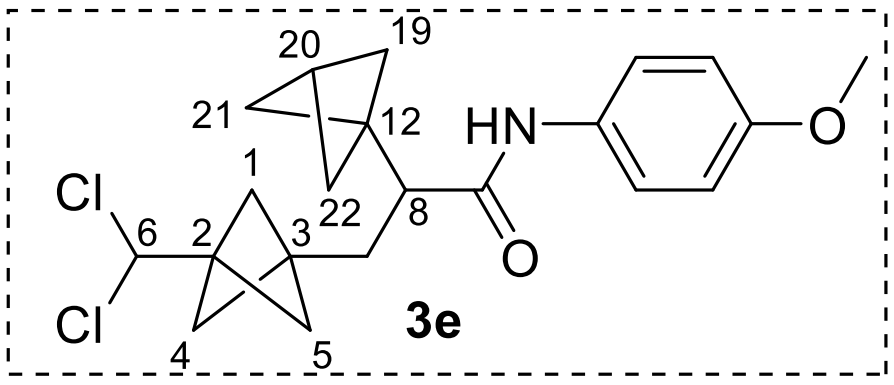

Synthesized according to *General Procedure A* using acrylamide **2e** (17.7 mg, 0.1 mmol, 1.0 equiv.) and **1** (0.2 mmol, 2.0 equiv.). Purification is through flash column chromatography (0-30% Et_2O/Hex) to give product **3e** (19.7 mg, 0.05 mmol, 50%) as a white solid.

R_f 0.3 (30% Et_2O/hexane); **^{1}H NMR** (600 MHz, $CDCl_3$) δ 7.42–7.34 (m, 2H, ArH), 6.90 (s, 1H, exchangeable NH), 6.89–6.85 (m, 2H, ArH), 5.71 (s, 1H, H6), 3.79 (s, 3H, OCH_3), 2.51 (s, 1H, H20), 2.30 (dd, *J* = 11.6, 2.9 Hz, 1H, H8), 2.08 (dd, *J* = 14.3, 11.6 Hz, 1H, H7), 1.78 – 1.65 (m, 12H, H1,H4,H5,H19,H21,H22), 1.61 (dd, *J* = 14.4, 2.8 Hz, 1H, H7'); **^{13}C NMR** (151 MHz, $CDCl_3$) δ 170.4, 156.7, 130.9, 122.1, 114.4, 72.0, 55.7, 49.5, 49.1, 48.4, 45.7, 44.4, 37.7, 30.2, 26.8; **HRMS** (ESI^+) calc. for $C_{21}H_{25}Cl_2NO_2$ $[M+H]^+$ 394.1335, found 394.1337.

2-(bicyclo[1.1.1]pentan-1-yl)-*N*-(2-bromophenyl)-3-(3-(dichloromethyl)bicyclo[1.1.1]pentan-1-yl)propenamide (3f)

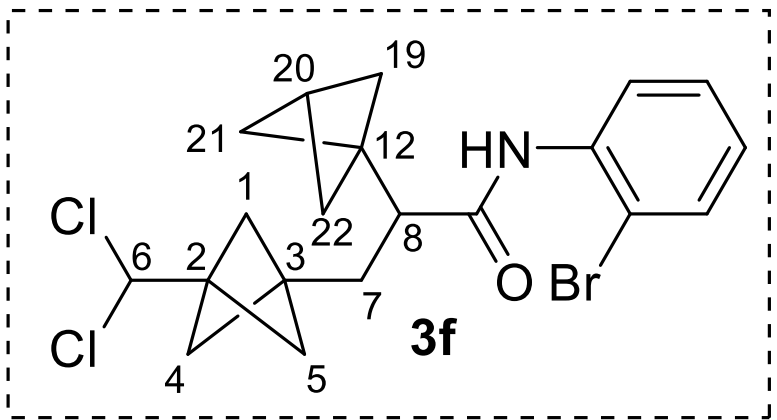

Synthesized according to *General Procedure A* using acrylamide **2f** (22.6 mg, 0.1 mmol, 1.0 equiv.) and **1** (0.2 mmol, 2.0 equiv.). Purification is through flash column chromatography (0-10% Et_2O/Hex) to give product **3f** (15.1 mg, 0.034 mmol, 34%) as a white solid.

R_f 0.7 (20% Et_2O/hexane); **^{1}H NMR** (600 MHz, $CDCl_3$) δ 8.30 (t, *J* = 6.5 Hz, 1H, ArH), 7.58–7.46 (m, 2H, ArH), 7.36–7.17 (m, 1H, ArH overlap with $CDCl_3$), 6.99 (s, 1H, exchangeable NH), 5.71 (s, 1H, H6), 2.53 (t, *J* = 3.0 Hz, 1H, H20), 2.46–2.37 (m, 1H, H8), 2.13-2.03 (m, 1H, H7), 1.83–1.63 (m, 13H, H1,H4,H5,H19,H21,H22 and H7'); **^{13}C NMR** (151 MHz, $CDCl_3$) δ 170.7, 135.7, 132.4, 128.6, 125.3, 122.2, 113.4, 72.0, 49.5, 49.1, 49.1, 45.3, 44.4, 37.6, 30.4, 26.9; **HRMS** (ESI^+) calc. for $C_{20}H_{22}BrCl_2NO$ $[M+H]^+$ 442.0335, found 442.0338.

2-(bicyclo[1.1.1]pentan-1-yl)-3-(3-(dichloromethyl)bicyclo[1.1.1]pentan-1-yl)-*N*-(4-phenoxyphenyl)propenamide (3g)

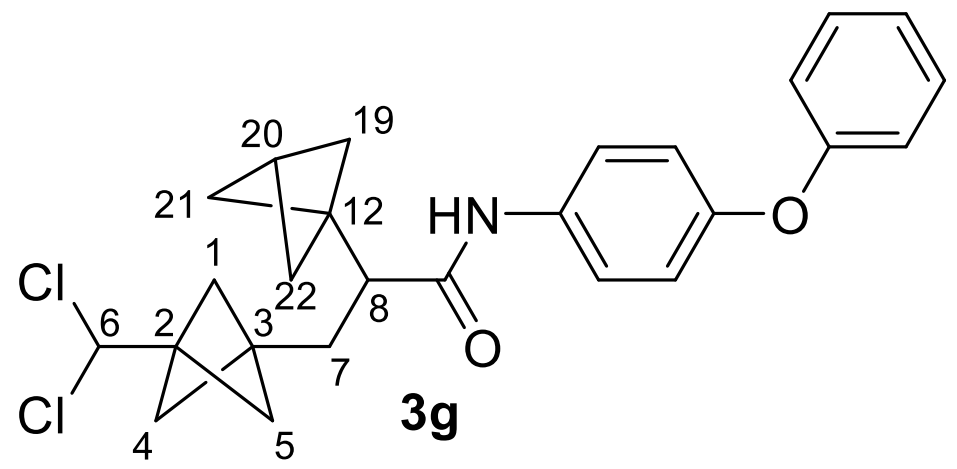

3g

Synthesized according to *General Procedure A* using acrylamide **2g** (23.9 mg, 0.1 mmol, 1.0 equiv.) and **1** (0.2 mmol, 2.0 equiv.). Purification is through flash column chromatography (0-25% Et_2O/Hex) to give product **3g** (14.6 mg, 0.032 mmol, 32%) as a white solid.

R_f 0.3 (25% Et_2O/hexane);**^{1}H NMR** (600 MHz, $CDCl_3$) δ 7.48–7.43 (m, 2H, ArH), 7.34–7.30 (m, 2H, ArH), 7.08 (tt, *J* = 7.4, 1.2 Hz, 1H, ArH), 7.02–6.96 (m, 5H, ArH and exchangeable NH), 5.71 (s, 1H, H6), 2.52 (s, 1H, H20), 2.32 (dd, *J* = 11.6, 2.8 Hz, 1H, H8), 2.09 (dd, *J* = 14.4, 11.6 Hz, 1H, H7), 1.78–1.66 (m, 12H, H1,H4,H5,H19,H21,H22), 1.64–1.56 (m, 1H, H7' overlap with water peak); **^{13}C NMR** (151 MHz, $CDCl_3$) δ 170.6, 157.7, 153.7, 133.3, 129.9, 123.2, 121.9, 119.8, 118.6, 72.0, 49.5, 49.1, 48.5, 45.6, 44.4, 37.7, 30.1, 26.9; **HRMS** (ESI^+) calc. for $C_{26}H_{27}Cl_2NO2$ $[M+H]^+$456.1492, found 456.1492.

2-(bicyclo[1.1.1]pentan-1-yl)-3-(3-(dichloromethyl)bicyclo[1.1.1]pentan-1-yl)-*N*-(4-(4,4,5,5-tetramethyl-1,3,2-dioxaborolan-2-yl)phenyl)propenamide (3h)

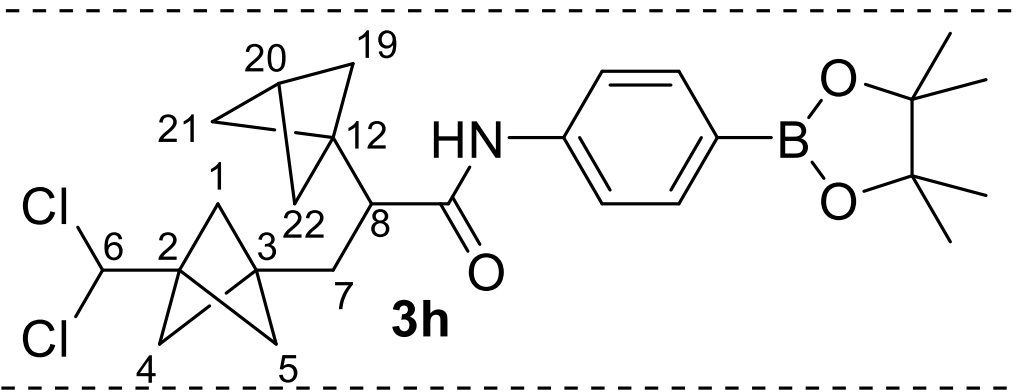

3h

Synthesized according to *General Procedure A* using acrylamide **2h** (27.3 mg, 0.1 mmol, 1.0 equiv.) and **1** (0.2 mmol, 2.0 equiv.). Purification is through flash column chromatography (0-40% Et_2O/Hex) to give product **3h** (14.2 mg, 0.029 mmol, 29%) as a white solid.

R_f 0.28 (30% Et_2O/hexane);**^{1}H NMR** (600 MHz, $CDCl_3$) δ 7.80–7.76 (m, 2H, ArH), 7.54–7.50 (m, 2H, ArH), 7.02 (s, 1H, exchangeable NH), 5.69 (s, 1H, H6), 2.51 (s, 1H, H20), 2.32 (dd, *J* = 11.5, 2.9 Hz, 1H, H8), 2.08 (dd, *J* = 14.4, 11.5 Hz, 1H, H7), 1.75–1.65 (m, 12H, H1,H4,H5,H19,H21,H22), 1.63-1.57 (m, 1H, H7'), 1.33 (s, 12H, CH_3); **^{13}C NMR** (151 MHz, $CDCl_3$) δ 170.6, 140.5, 136.0, 118.8, 83.9, 72.0, 49.5, 49.1, 48.8, 45.5, 44.4, 37.6, 30.2, 29.8, 26.9, 25.0, 25.0; **HRMS** (ESI^+) calc. for $C_{26}H_{34}BCl_2NO_3$ $[M+H]^+$490.2082, found 490.2082.

N-benzyl-2-(bicyclo[1.1.1]pentan-1-yl)-3-(3-(dichloromethyl)bicyclo[1.1.1]pentan-1-yl)propenamide (3i)

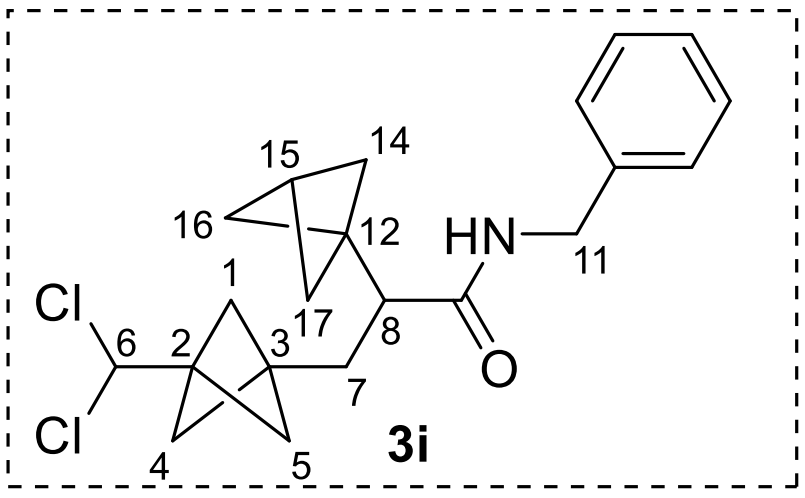

Synthesized according to *General Procedure A* using acrylamide **2i** (16.1 mg, 0.1 mmol, 1.0 equiv.) and **1** (0.2 mmol, 2.0 equiv.). Purification is through flash column chromatography (0-30% Et_2O/Hex) to give product **3i** (19.3 mg, 0.051 mmol, 51%) as a white solid.

R_f 0.25 (25% Et_2O/hexane); **^{1}H NMR** (600 MHz, $CDCl_3$) δ 7.36–7.27 (m, 5H, ArH), 5.71 (s, 1H, H6), 5.60 (t, *J* = 6.4 Hz, 1H, exchangeable NH), 4.47–4.39 (m, 2H, H11), 2.47 (s, 1H, H15), 2.16 (dd, *J* = 11.5, 2.8 Hz, 1H, H8), 2.01 (dd, *J* = 14.2, 11.5 Hz, 1H, H7), 1.70–1.61 (m, 12H, H1,H4,H5,H14,H16,H17), 1.53 (dd, *J* = 14.2, 2.9 Hz, 1H, H7'); **^{13}C NMR** (151 MHz, $CDCl_3$) δ 172.1, 138.5, 128.9, 128.3, 127.8, 72.1, 49.4, 49.1, 47.6, 45.7, 44.3, 43.9, 37.7, 30.0, 26.7; **HRMS** (ESI$^+$) calc. for $C_{21}H_{25}Cl_2NO$ $[M+H]^+$378.1386, found 378.1386.

2-(bicyclo[1.1.1]pentan-1-yl)-3-(3-(dichloromethyl)bicyclo[1.1.1]pentan-1-yl)-N-(2-methylbenzyl)propenamide (3j)

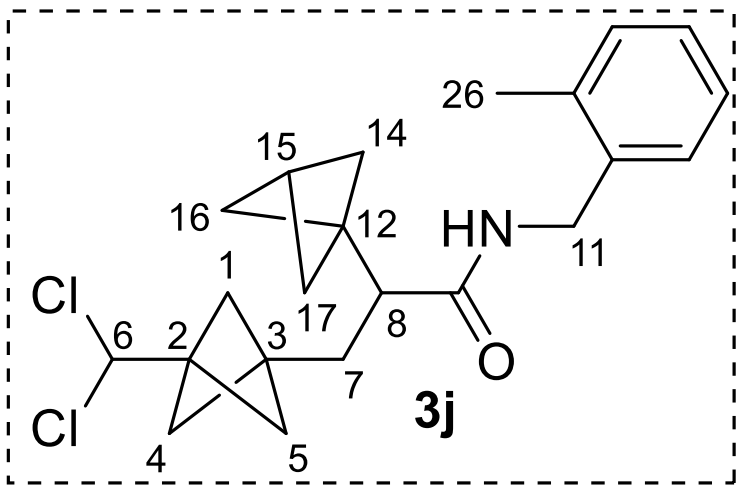

Synthesized according to *General Procedure A* using acrylamide **2j** (17.5 mg, 0.1 mmol, 1.0 equiv.) and **1** (0.2 mmol, 2.0 equiv.). Purification is through flash column chromatography (0-30% Et_2O/Hex) to give product **3j** (19.2 mg, 0.049 mmol, 49%) as a white solid.

R_f 0.27 (25% Et_2O/hexane);**^{1}H NMR** (600 MHz, $CDCl_3$) δ 7.26 (dd, *J* = 7.8, 1.8 Hz, 1H, ArH), 7.23–7.17 (m, 3H, ArH), 5.71 (s, 1H, H6), 5.43 (t, *J* = 5.2 Hz, 1H, exchangeable NH), 4.46–4.39 (m, 2H, H11), 2.47 (s, 1H, H15), 2.36 (s, 3H, CH_3), 2.16 (dd, *J* = 11.5, 2.8 Hz, 1H, H8), 2.01 (dd, *J* = 14.2, 11.5 Hz, 1H, H7), 1.69–1.62 (m, 12H, H1,H4,H5,H14,H16,H17), 1.53 (dd, *J* = 14.2, 2.9 Hz, 1H, H7'); **^{13}C NMR** (151 MHz, $CDCl_3$) δ 172.1, 136.5, 136.0, 130.7, 129.1, 128.0, 126.4, 72.1, 49.4, 49.0, 47.5, 45.7, 44.3, 42.0, 37.6, 30.1, 26.7, 19.2; **HRMS** (ESI$^+$) calc. for $C_{22}H_{27}Cl_2NO$ $[M+H]^+$392.1542, found 392.1543.

2-(bicyclo[1.1.1]pentan-1-yl)-*N*-butyl-3-(3-(dichloromethyl)bicyclo[1.1.1]pentan-1-yl)propenamide (3k)

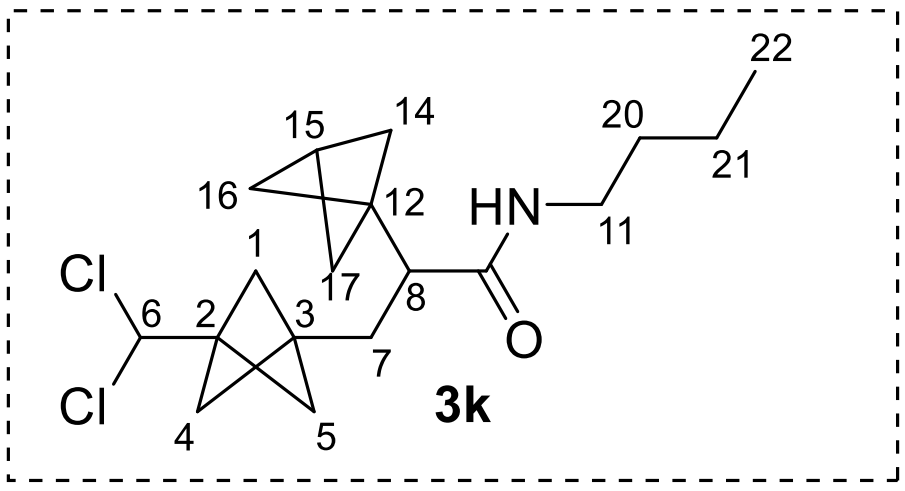

Synthesized according to *General Procedure A* using acrylamide **2k** (12.7 mg, 0.1 mmol, 1.0 equiv.) and **1** (0.2 mmol, 2.0 equiv.). Purification is through flash column chromatography (0-30% Et_2O/Hex) to give product **3k** (17.9 mg, 0.052 mmol, 52%) as a white solid.

R*f* 0.25 (25% Et_2O/hexane); **^{1}H NMR** (600 MHz, $CDCl_3$) δ 5.71 (s, 1H, H6), 5.32 (t, *J* = 5.4 Hz, 1H, exchangeable NH), 3.29–3.20 (m, 2H, H11), 2.47 (s, 1H, H15), 2.12 (dd, *J* = 11.5, 2.9 Hz, 1H, H8), 1.97 (dd, *J* = 14.2, 11.5 Hz, 1H, H7), 1.66–1.54 (m, 12H, H1,H4,H5,H14,H16,H17), 1.47–1.36 (m, 3H, H20 and H7'), 1.33–1.25 (m, 2H, H21), 0.93 (t, *J* = 7.3 Hz, 3H, H22); **^{13}C NMR** (151 MHz, $CDCl_3$) δ 172.1, 72.2, 49.4, 49.0, 47.6, 45.7, 44.3, 39.3, 37.7, 32.0, 30.0, 26.6, 20.3, 13.9; **HRMS** (ESI^+) calc. for $C_{18}H_{27}Cl_2NO$ $[M+H]^+$344.1542, found 344.1541.

2-(bicyclo[1.1.1]pentan-1-yl)-*N*-cyclopropyl-3-(3-(dichloromethyl)bicyclo[1.1.1]pentan-1-yl)propenamide (3l)

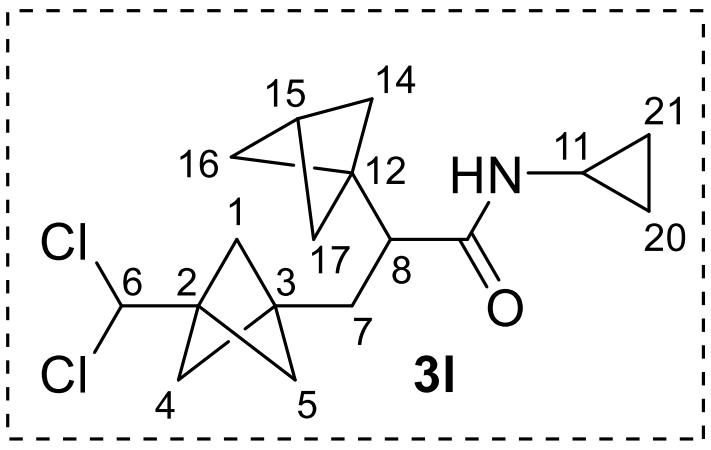

Synthesized according to *General Procedure A* using acrylamide **2l** (11.1 mg, 0.1 mmol, 1.0 equiv.) and **1** (0.2 mmol, 2.0 equiv.). Purification is through flash column chromatography (0-30% Et_2O/Hex) to give product **3l** (16.7 mg, 0.051 mmol, 51%) as a white solid.

R*f* 0.23 (25% Et_2O/hexane); **^{1}H NMR** (600 MHz, $CDCl_3$) δ 5.71 (s, 1H, H6), 5.51 (s, 1H, exchangeable NH), 2.71-2.65 (m, 1H, H11), 2.46 (s, 1H, H15), 2.09 (dd, *J* = 11.6, 2.8 Hz, 1H, H8), 1.96 (dd, *J* = 14.2, 11.5 Hz, 1H, H7), 1.73–1.58 (m, 12H, H1,H4,H5,H14,H16,H17), 1.50 (dd, *J* = 14.2, 2.7 Hz, 1H, H7'), 0.80-0.74 (m, 2H, H21), 0.51–0.43 (m, 2H, H20); **^{13}C NMR** (151 MHz, $CDCl_3$) δ 173.6, 72.1, 49.4, 49.0, 47.3, 45.6, 44.3, 37.7, 29.9, 26.7, 22.6, 7.0, 6.9; **HRMS** (ESI^+) calc. for $C_{17}H_{23}Cl_2NO$ $[M+H]^+$328.1229, found 328.1228.

2-(bicyclo[1.1.1]pentan-1-yl)-*N*-cyclohexyl-3-(3-(dichloromethyl)bicyclo[1.1.1]pentan-1-yl)propenamide (3m)

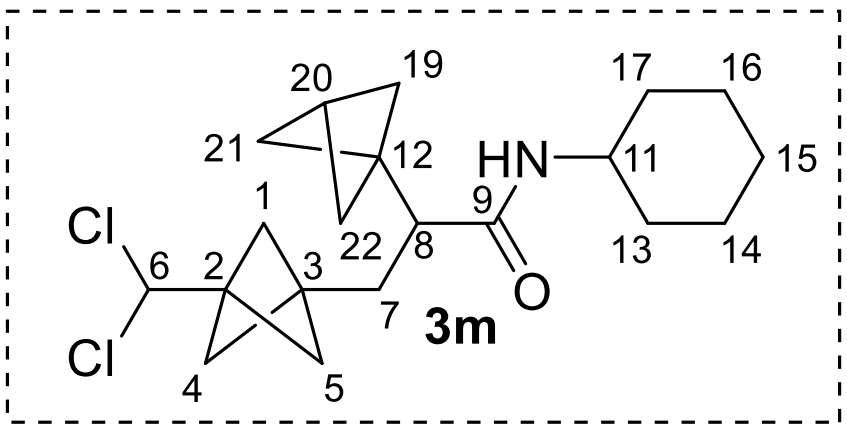

Synthesized according to *General Procedure A* using acrylamide **2m** (15.3 mg, 0.1 mmol, 1.0 equiv.) and **1** (0.2 mmol, 2.0 equiv.). Purification is through flash column chromatography (0-30% Et_2O/Hex) to give product **3m** (20.7 mg, 0.056 mmol, 56%) as a white solid.

R*f* 0.3 (25% Et_2O/hexane);**^{1}H NMR** (600 MHz, $CDCl_3$) δ 5.71 (s, 1H, H6), 5.18 (d, *J* = 8.4 Hz, 1H, exchangeable NH), 3.78 (tdt, *J* = 11.3, 8.1, 4.0 Hz, 1H, H11), 2.46 (s, 1H, H20), 2.09 (dd, *J* = 11.6, 2.9 Hz, 1H, H8), 1.97–1.87 (m, 3H, H7 and cyclohex-CH_2), 1.71–1.61 (m, 14H, H1,H4,H5,H19,H21,H22 and cyclohex-CH_2), 1.52–1.32 (m, 4H, H7' and cyclohex-CH_2), 1.20–1.06 (m, 3H, cyclohex-CH_2); **^{13}C NMR** (151 MHz, $CDCl_3$) δ 171.0, 72.2, 49.3, 49.1, 47.7, 47.7, 45.7, 44.3, 37.7, 33.7, 33.4, 29.9, 26.6, 25.7, 25.0; **HRMS** (ESI^+) calc. for $C_{20}H_{29}Cl_2NO$ $[M+H]^+$370.1699, found 370.1698.

2-(bicyclo[1.1.1]pentan-1-yl)-3-(3-(dichloromethyl)bicyclo[1.1.1]pentan-1-yl)-*N*-(2-methyl-4-oxopentan-2-yl)propenamide (3n)

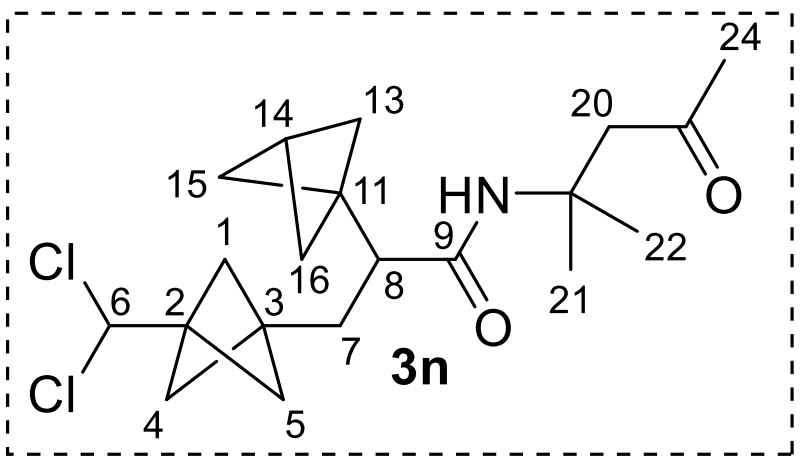

Synthesized according to *General Procedure A* using acrylamide **2n** (16.9 mg, 0.1 mmol, 1.0 equiv.) and **1** (0.2 mmol, 2.0 equiv.). Purification is through flash column chromatography (0-35% Et_2O/Hex) to give product **3n** (20.1 mg, 0.052 mmol, 52%) as a colorless oil.

R*f* 0.24 (28% Et_2O/hexane);**^{1}H NMR** (600 MHz, $CDCl_3$) δ 5.83 (s, 1H, exchangeable NH), 5.71 (s, 1H, H6), 2.87 (d, *J* = 5.8 Hz, 2H, H20), 2.45 (s, 1H. H14), 2.14 (s, 3H, H24), 2.06 (dd, *J* = 11.6, 2.6 Hz, 1H, H8), 1.92 (dd, *J* = 14.2, 11.6 Hz, 1H, H7), 1.69–1.60 (m, 12H, H1,H4,H5,H13,H15,H16), 1.45 (dd, *J* = 14.3, 2.6 Hz, 1H, H7'), 1.42 (s, 3H, H21), 1.40 (s, 3H, H22); **^{13}C NMR** (151 MHz, $CDCl_3$) δ 208.3, 171.7, 72.2, 52.4, 51.7, 49.4, 49.1, 47.9, 45.8, 44.2, 37.7, 32.1, 29.9, 27.5, 27.4, 26.6; **HRMS** (ESI^+) calc. for $C_{20}H_{29}Cl_2NO_2$ $[M+H]^+$386.1648, found 386.1645.

2-(bicyclo[1.1.1]pentan-1-yl)-3-(3-(dichloromethyl)bicyclo[1.1.1]pentan-1-yl)-*N*,*N*-dimethylpropanamide (3o)

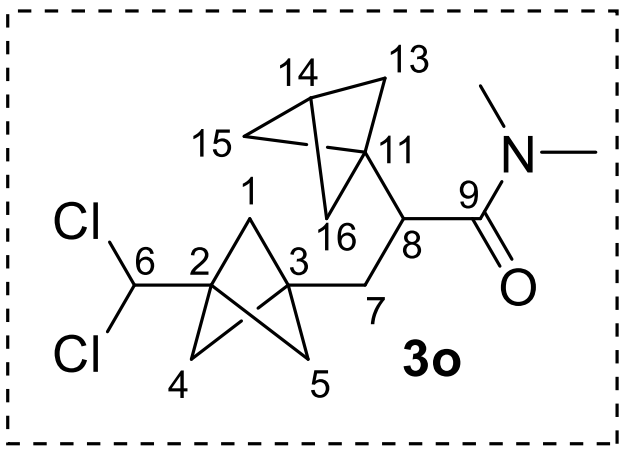

Synthesized according to *General Procedure A* using acrylamide **2o** (9.9 mg, 0.1 mmol, 1.0 equiv.) and **1** (0.2 mmol, 2.0 equiv.). Purification is through flash column chromatography (0-35% Et_2O/Hex) to give product **3o** (12.3 mg, 0.039 mmol, 39%) as a white solid.

$\mathbf{R_f}$ 0.24 (28% Et_2O/hexane); **^{1}H NMR** (600 MHz, $CDCl_3$) δ 5.71 (s, 1H, H6), 3.05 (s, 3H, CH_3), 2.95 (s, 3H, CH_3), 2.77 (dd, *J* = 11.2, 2.8 Hz, 1H, H8), 2.46 (s, 1H, H14), 2.13 (dd, *J* = 13.9, 11.3 Hz, 1H, H7), 1.69-1.54 (m, 12H, H1, H4, H5, H13, H15, H16), 1.49 (dd, *J* = 13.9, 2.8 Hz, 1H, H7'); **^{13}C NMR** (151 MHz, $CDCl_3$) δ 172.5, 72.2, 49.6, 49.1, 47.7, 45.5, 44.2, 41.0, 37.8, 30.5, 27.2; **HRMS** (ESI^+) calc. for $C_{16}H_{23}Cl_2NO$ $[M+H]^+$316.1229, found 316.1229.

2-(bicyclo[1.1.1]pentan-1-yl)-3-(3-(dichloromethyl)bicyclo[1.1.1]pentan-1-yl)-1-morpholinopropan-1-one (3p)

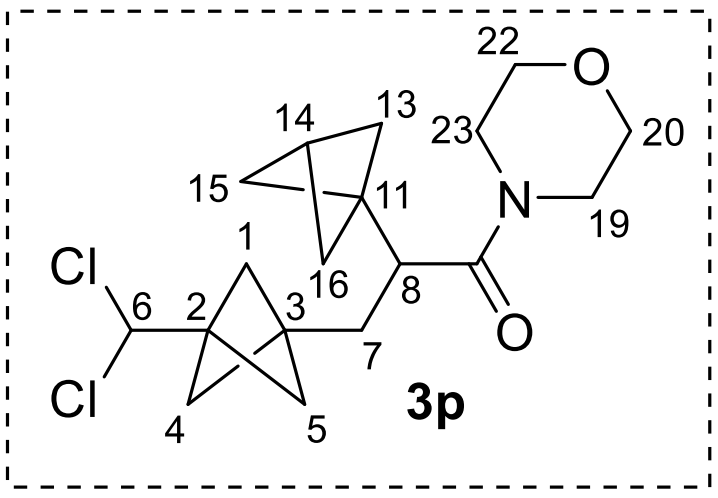

Synthesized according to *General Procedure A* using acrylamide **2p** (14.1 mg, 0.1 mmol, 1.0 equiv.) and **1** (0.2 mmol, 2.0 equiv.). Purification is through flash column chromatography (0-35% Et_2O/Hex) to give product **3p** (19.0 mg, 0.053 mmol, 53%) as a white solid.

$\mathbf{R_f}$ 0.23 (28% Et_2O/hexane); **^{1}H NMR** (600 MHz, $CDCl_3$) δ 5.72 (s, 1H, H6), 3.74–3.50 (m, 8H, H19, H20, H22, H23), 2.72 (dd, *J* = 11.2, 2.7 Hz, 1H, H8), 2.47 (s, 1H, H14), 2.17 (dd, *J* = 13.9, 11.2 Hz, 1H, H7), 1.68–1.56 (m, 12H, H1,H4,H5,H13,H15,H16), 1.51 (dd, *J* = 13.9, 2.7 Hz, 1H, H7'); **^{13}C NMR** (151 MHz, $CDCl_3$) δ 171.0, 72.1, 67.3, 66.9, 49.6, 49.1, 47.7, 46.3, 45.3, 44.2, 42.1, 40.6, 37.8, 30.3, 27.2; **HRMS** (ESI^+) calc. for $C_{18}H_{53}Cl_2NO_2$ $[M+H]^+$358.1335, found 358.1331.

2-(bicyclo[1.1.1]pentan-1-yl)-3-(3-(dichloromethyl)bicyclo[1.1.1]pentan-1-yl)-2-methyl-*N*-phenylpropanamide (3q)

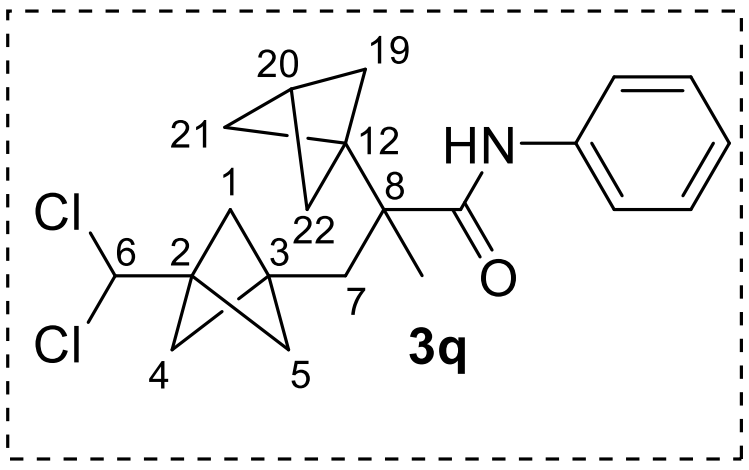

Synthesized according to *General Procedure A* using acrylamide **2q** (16.1 mg, 0.1 mmol, 1.0 equiv.) and **1** (0.2 mmol, 2.0 equiv.). Purification is through flash column chromatography (0-20% Et_2O/Hex) to give product **3q** (15.9 mg, 0.042 mmol, 42%) as a white solid.

R*f* 0.3 (20% Et_2O/hexane);**^{1}H NMR** (600 MHz, $CDCl_3$) δ 7.52–7.43 (m, 2H, ArH), 7.38–7.28 (m, 2H, ArH), 7.17–7.05 (m, 2H, ArH and exchangeable NH), 5.68 (s, 1H, H6), 2.52 (s, 1H, H20), 2.19 (d, *J* = 14.8 Hz, 1H, H7), 1.80–1.68 (m, 12H, H1,H4,H5,H19,H21,H22), 1.55 (d, *J* = 14.8 Hz, 1H, H7'), 1.25 (s, 3H, CH_3); **^{13}C NMR** (151 MHz, $CDCl_3$) δ 172.0, 137.8, 129.2, 124.4, 120.2, 71.8, 50.9, 50.5, 47.5, 46.7, 45.0, 37.2, 36.7, 25.2, 18.3; **HRMS** (ESI^+) calc. for $C_{21}H_{25}Cl_2NO$ $[M+H]^+$378.1386, found 378.1387.

2-(bicyclo[1.1.1]pentan-1-yl)-3-(3-(dichloromethyl)bicyclo[1.1.1]pentan-1-yl)-*N*-(4-methoxyphenyl)-2-methylpropanamide (3r)

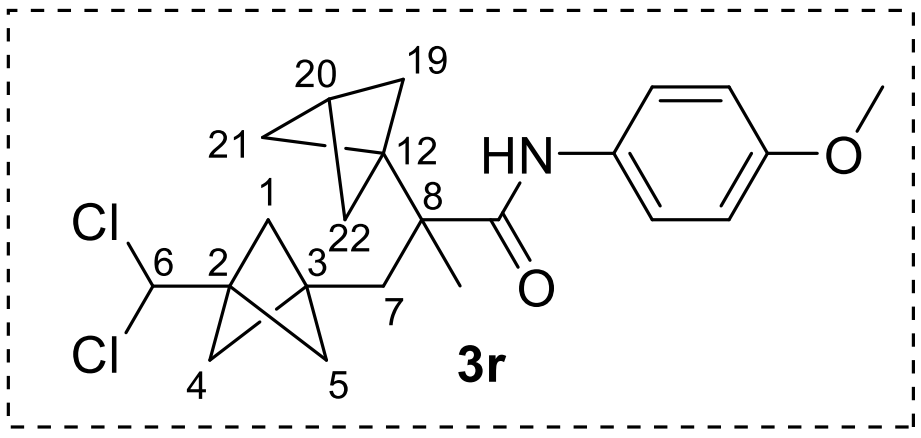

Synthesized according to *General Procedure A* using acrylamide **2r** (19.1 mg, 0.1 mmol, 1.0 equiv.) and **1** (0.2 mmol, 2.0 equiv.). Purification is through flash column chromatography (0-30% Et_2O/Hex) to give product **3r** (16.3 mg, 0.04 mmol, 40%) as a white solid.

R*f* 0.18 (20% Et_2O/hexane);**^{1}H NMR** (600 MHz, $CDCl_3$) δ 7.43–7.35 (m, 2H, ArH), 7.01 (s, 1H, exchangeable NH), 6.89–6.84 (m, 2H, ArH), 5.68 (s, 1H, H6), 3.79 (s, 3H, OCH_3), 2.51 (s, 1H, H20), 2.18 (d, *J* = 14.8 Hz, 1H, H7), 1.81–1.66 (m, 12H, H1,H4,H5,H19,H21,H22), 1.53 (d, *J* = 14.8 Hz, 1H, H7'), 1.24 (s, 3H, CH_3); **^{13}C NMR** (151 MHz, $CDCl_3$) δ 171.8, 156.6, 130.9, 122.1, 114.4, 71.8, 55.7, 50.9, 50.5, 47.5, 46.5, 45.0, 37.1, 36.8, 25.2, 18.3; **HRMS** (ESI^+) calc. for $C_{22}H_{27}Cl_2NO_2$ $[M+H]^+$408.1492, found 408.1490.

2-(bicyclo[1.1.1]pentan-1-yl)-3-(3-(dichloromethyl)bicyclo[1.1.1]pentan-1-yl)-2-methyl-*N*-(4-(trifluoromethyl)phenyl)propenamide (3s)

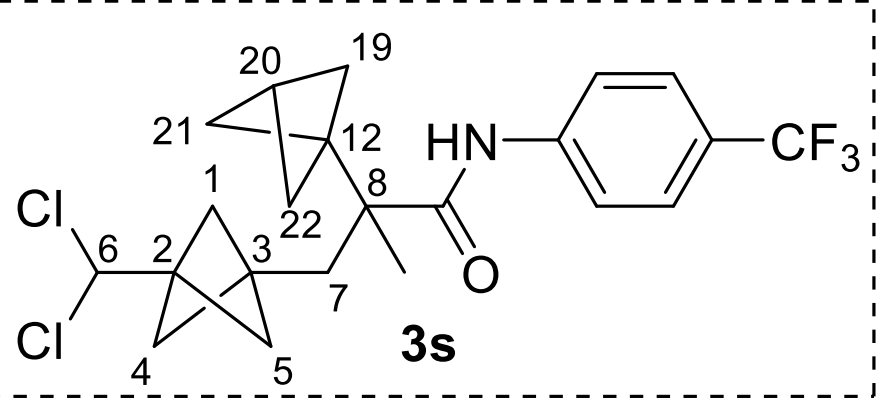

3s

Synthesized according to *General Procedure A* using acrylamide **2s** (24.3 mg, 0.1 mmol, 1.0 equiv.) and **1** (0.2 mmol, 2.0 equiv.). Purification is through flash column chromatography (0-25% Et_2O/Hex) to give product **3s** (13.4 mg, 0.03 mmol, 30%) as a colorless oil.

R*f* 0.25 (20% Et_2O/hexane);**^{1}H NMR** (600 MHz, $CDCl_3$) δ 7.65–7.57 (m, 4H, ArH), 7.22 (s, 1H, exchangeable NH), 5.67 (s, 1H, H6), 2.54 (s, 1H, H20), 2.18 (d, *J* = 14.9 Hz, 1H, H7), 1.78–1.67 (m, 12H, H1,H4,H5,H19,H21,H22), 1.59-1.50 (m, 1H, H7' overlap with water peak), 1.26 (s, 3H, CH_3); **^{13}C NMR** (151 MHz, $CDCl_3$) δ 172.4, 140.8, 126.5 (q, *J* = 3.7 Hz), 126,2 (q, *J* = 30.2 Hz) , 124.2 (d, *J* = 271.8 Hz), 119.6, 71.7, 50.7, 50.5, 47.5, 47.0, 45.0, 37.2, 36.7, 25.3, 18.2; **HRMS** (ESI^+) calc. for $C_{22}H_{24}Cl_2F_3NO$ $[M-H]^-$444.1103, found 444.1119.

2-(bicyclo[1.1.1]pentan-1-yl)-3-(3-(dichloromethyl)bicyclo[1.1.1]pentan-1-yl)-2-methyl-*N*-phenylbutanamide (3t)

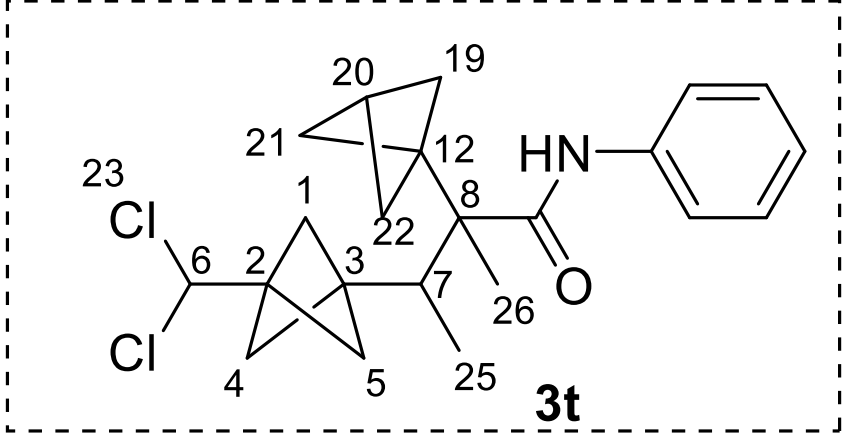

3t

Synthesized according to *General Procedure A* using acrylamide **2t** (17.5 mg, 0.1 mmol, 1.0 equiv.) and **1** (0.2 mmol, 2.0 equiv.). Purification is through flash column chromatography (0-20% Et_2O/Hex) to give product **3t** (12.6 mg, 0.032 mmol, 32%) as a white solid.

R*f* 0.3 (20% Et_2O/hexane);**^{1}H NMR** (600 MHz, $CDCl_3$) δ 7.53–7.43 (m, 2H, ArH), 7.37–7.30 (m, 2H, ArH), 7.16–7.08 (m, 2H, ArH and exchangeable NH), 5.66 (s, 1H, H6), 2.50 (s, 1H, H20), 2.39 (q, *J* = 6.9 Hz, 1H, H7), 1.85–1.70 (m, 12H, H1,H4,H5,H19,H21,H22), 1.13 (s, 3H, H26), 1.00 (d, *J* = 7.0 Hz, 3H, H25); **^{13}C NMR** (151 MHz, $CDCl_3$) δ 172.9, 137.8, 129.2, 124.4, 120.1, 72.1, 50.8, 49.7, 49.3, 48.9, 43.9, 41.8, 37.8, 26.3, 12.8; **HRMS** (ESI^+) calc. for $C_{22}H_{27}Cl_2NO$ $[M+H]^+$392.1542, found 392.1541.

(5*S*)-3-(2-(bicyclo[1.1.1]pentan-1-yl)-3-(3-(dichloromethyl)bicyclo[1.1.1]pentan-1-yl)propanoyl)-5-isopropyloxazolidin-2-on e (3ut and 3ub)

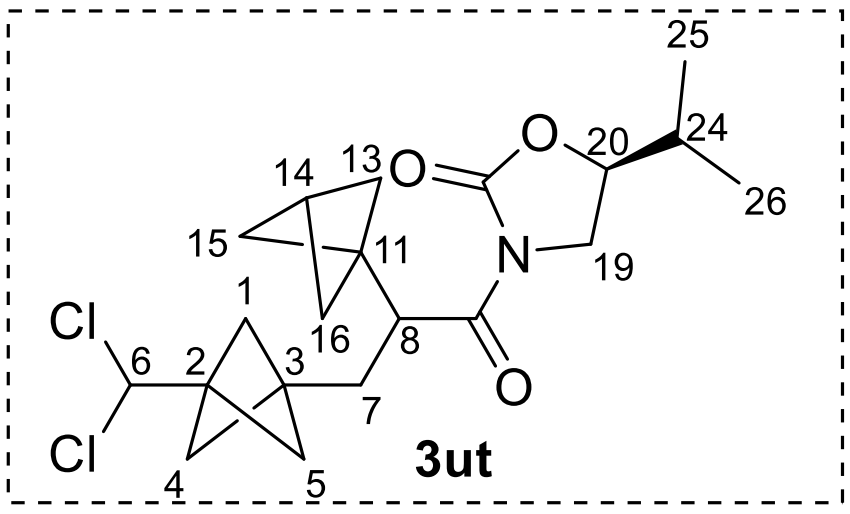

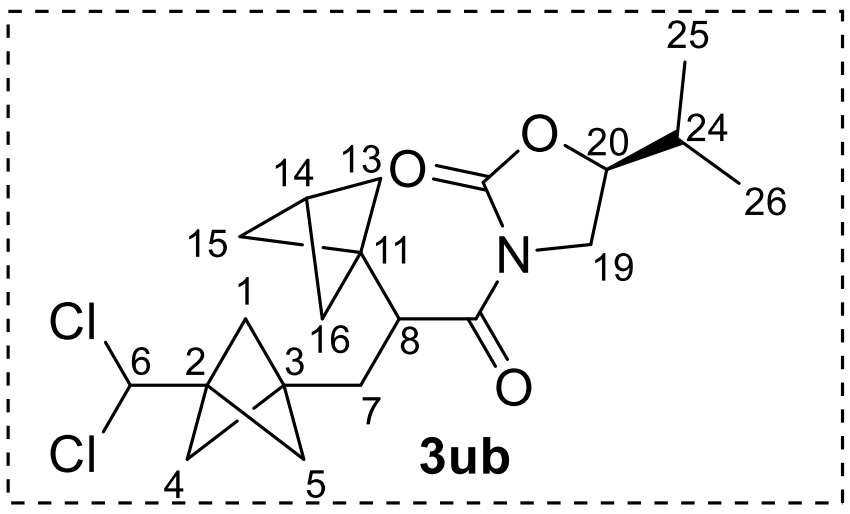

Synthesized according to *General Procedure A* using acrylamide **2u** (18.3 mg, 0.1 mmol, 1.0 equiv.) and **1** (0.2 mmol, 2.0 equiv.). Purification is through flash column chromatography (0-20% Et_2O/Hex) to give product **3ut** (8.8 mg, 0.022 mmol, 22%) as a white solid and **3ub** (9.1 mg, 0.023 mmol, 23%) as a white solid.

3ut (one diastereomer):

R*f* 0.28 (20% Et_2O/hexane); **^{1}H NMR** (600 MHz, $CDCl_3$) δ 5.70 (s, 1H, H6), 4.46 (dt, *J* = 8.2, 3.1 Hz, 1H, H20), 4.29–4.21 (m, 2H, H19), 4.08 (dd, *J* = 11.7, 2.8 Hz, 1H, H8), 2.48 (s, 1H, H14), 2.34 (pd, *J* = 7.0, 3.4 Hz, 1H, H24), 2.12 (dd, *J* = 14.1, 11.7 Hz, 1H, H7), 1.73–1.57 (m, 13H, H1,H4,H5,H13,H15,H16 and H7'), 0.93 (dd, *J* = 12.5, 7.0 Hz, 6H, H25 and H26); **^{13}C NMR** (151 MHz, $CDCl_3$) δ 173.5, 153.9, 72.0, 63.2, 59.0, 49.7, 49.2, 45.3, 44.2, 42.2, 37.7, 30.3, 28.8, 27.2, 18.4, 14.8; **HRMS** (ESI^+) calc. for $C_{20}H_{27}Cl_2NO_3$ $[M+H]^+$400.1441, found 400.1439.

3ub (the other diastereomer):

R*f* 0.26 (20% Et_2O/hexane); **^{1}H NMR** (600 MHz, $CDCl_3$) δ 5.71 (s, 1H, H6), 4.43 (dt, *J* = 7.7, 3.1 Hz, 1H, H20), 4.24–4.19 (m, 2H, H19), 4.17 (dd, *J* = 11.8, 2.6 Hz, 1H, H8), 2.48 (s, 1H, H14), 2.39 (pd, *J* = 7.0, 3.4 Hz, 1H, H24), 2.14 (dd, *J* = 14.1, 11.7 Hz, 1H, H7), 1.71–1.56 (m, 13H, H1, H4,H5,H13,H15,H16 and H7'), 0.92 (dd, *J* = 22.5, 7.0 Hz, 6H, H25 and H26); **^{13}C NMR** (151 MHz, $CDCl_3$) δ 173.1, 154.0, 72.1, 63.1, 59.1, 49.4, 49.2, 45.3, 44.3, 42.4, 37.7, 29.8, 28.6, 26.8, 18.3, 14.6; **HRMS** (ESI^+) calc. for $C_{20}H_{27}Cl_2NO_3$ $[M+H]^+$400.1441, found 400.1442.

methyl (2-(bicyclo[1.1.1]pentan-1-yl)-3-(3-(dichloromethyl)bicyclo[1.1.1]pentan-1-yl)propanoyl)-*L*-phenylalanyl-*L*-leucinate (3v, inseparable mixture of 1:1 diastereomers)

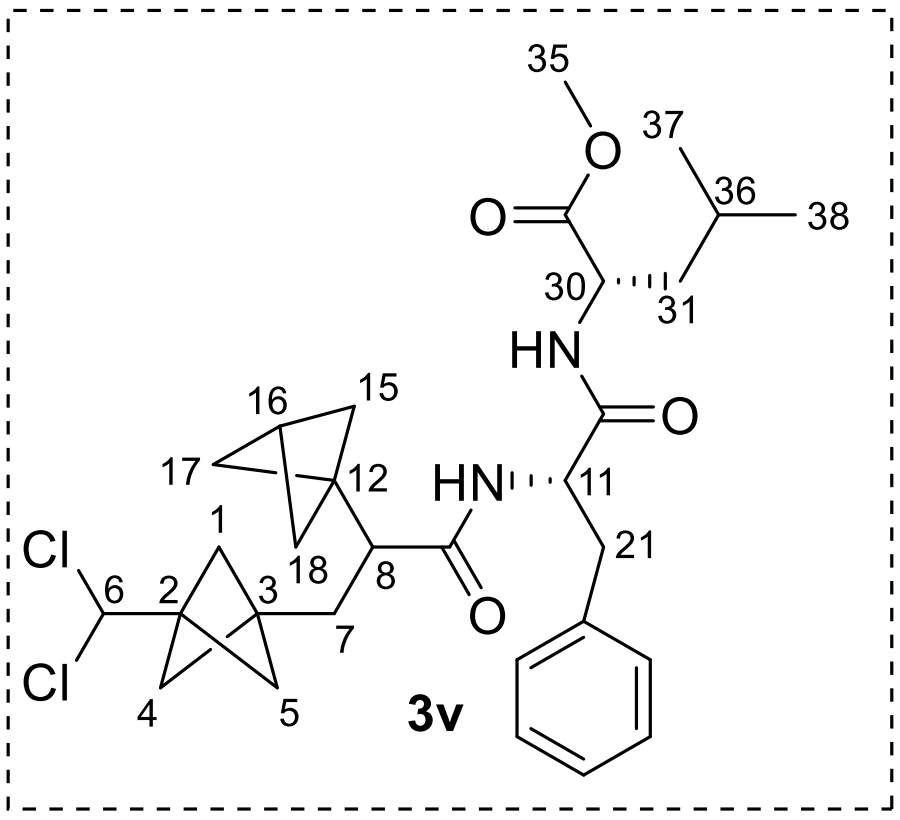

Synthesized according to *General Procedure A* using acrylamide **2v** (34.6 mg, 0.1 mmol, 1.0 equiv.) and **1** (0.2 mmol, 2.0 equiv.). Purification is through flash column chromatography (0-60% Et_2O/Hex) to give product **3ut** (24.8 mg, 0.044 mmol, 44%) as a white solid.

R*f* 0.4 (60% Et_2O/hexane);**^{1}H NMR** (600 MHz, $CDCl_3$) δ 7.33–7.21 (m, 10H, ArH overlap with $CDCl_3$), 6.21 (d, *J* = 7.9 Hz, 1H, exchangeable NH), 6.08 (d, *J* = 7.9 Hz, 1H, exchangeable NH), 5.98 (d, *J* = 7.5 Hz, 1H, exchangeable NH), 5.94 (d, *J* = 7.6 Hz, 1H, exchangeable NH), 5.68 (s, 1H, H6), 5.64 (s, 1H, H6), 4.65 (dq, *J* = 10.2, 7.4 Hz, 2H, H11), 4.54 – 4.48 (m, 2H, H30), 3.70 (s, 3H, OCH_3), 3.69 (s, 3H, OCH_3), 3.13–2.96 (m, 6H, H21 and H36), 2.44 (s, 1H, H16), 2.41 (s, 1H, H16), 2.14 (ddd, *J* = 20.5, 11.6, 2.8 Hz, 2H, H8), 1.92 (ddd, *J* = 14.3, 11.6, 8.0 Hz, 2H, H7), 1.64–1.41 (m, 30H, H1,H4,H5,H15,H17,H18,H31 and H7'), 0.91–0.86 (m, 12H, H37 and H38); **^{13}C NMR** (151 MHz, $CDCl_3$) δ 172.7, 172.7, 172.6, 172.5, 170.9, 170.7, 136.8, 136.6, 129.4, 129.4, 129.0, 128.9, 127.3, 127.3, 72.0, 72.0, 54.5, 54.4, 52.4, 52.4, 51.1, 51.1, 49.3, 49.3, 48.9, 48.9, 47.3, 47.3, 45.5, 45.4, 44.3, 44.2, 41.6, 41.6, 38.8, 38.2, 37.5, 37.4, 30.1, 29.8, 26.6, 26.6, 24.9, 24.8, 22.8, 22.8, 22.2, 22.0; **HRMS** (ESI^+) calc. for $C_{30}H_{40}Cl_2N_2O_4$ $[M+H]^+$563.2438, found 563.2438.

2-(bicyclo[3.1.1]heptan-1-yl)-3-(5-(dichloromethyl)bicyclo[3.1.1]heptan-1-yl)-*N*-phenylpropanamide (6a)

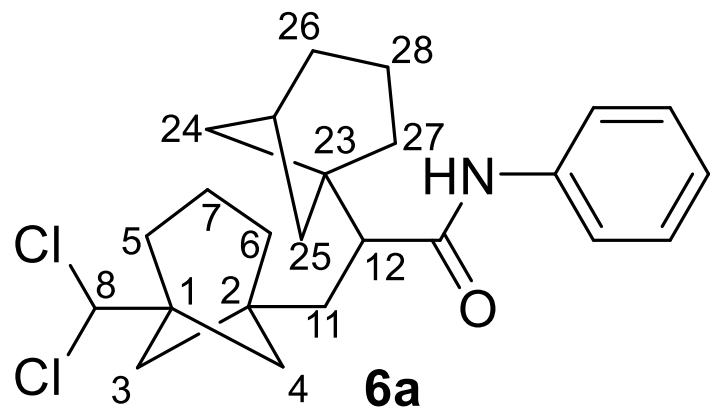

6a

Synthesized according to *General Procedure A* using acrylamide **2a** (14.7 mg, 0.1 mmol, 1.0 equiv.) and **5** (0.2 mmol, 2.0 equiv.). Purification is through flash column chromatography (0-20% Et_2O/Hex) to give product **6a** (21.4 mg, 0.051 mmol, 51%) as a colorless oil.

R_f 0.3 (20% Et_2O/hexane);**^{1}H NMR** (600 MHz, $CDCl_3$) δ 7.50–7.45 (m, 2H, ArH), 7.35–7.30 (m, 2H, ArH), 7.11 (tt, J = 7.3, 1.2 Hz, 1H, ArH), 6.95 (s, 1H, exchangeable NH), 5.59 (s, 1H, H8), 2.29 (tt, J = 6.4, 3.1 Hz, 1H, H22), 2.05–1.97 (m, 2H, H12 and H11), 1.92–1.59 (m, 16H, BCHep-CH_2), 1.54–1.46 (m, 4H, BCHep-CH_2), 1.27-1.23 (m, 1H, H11'); **^{13}C NMR** (151 MHz, $CDCl_3$) δ 171.6, 138.0, 129.2, 124.4, 120.1, 81.0, 53.8, 46.6, 44.9, 40.4, 39.7, 37.2, 37.1, 36.7, 36.3, 33.1, 31.2, 29.9, 28.6, 27.8, 17.1, 16.8; **HRMS** (ESI^+) calc. for $C_{24}H_{31}Cl_2NO$ $[M+H]^+$420.1855, found 420.1854.

2-(bicyclo[3.1.1]heptan-1-yl)-3-(5-(dichloromethyl)bicyclo[3.1.1]heptan-1-yl)-*N*-(4-methoxyphenyl)propenamide (6b)

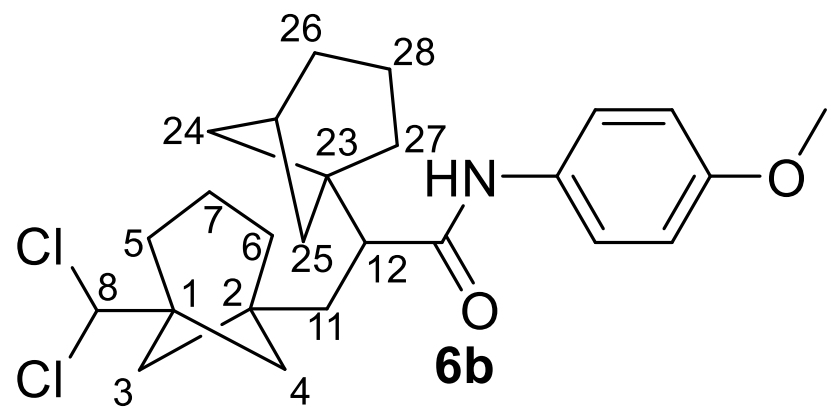

6b

Synthesized according to *General Procedure A* using acrylamide **2e** (17.7mg, 0.1 mmol, 1.0 equiv.) and **5** (0.2 mmol, 2.0 equiv.). Purification is through flash column chromatography (0-30% Et_2O/Hex) to give product **6b** (19.4 mg, 0.043 mmol, 43%) as a colorless oil.

R_f 0.17 (20% Et_2O/hexane);**^{1}H NMR** (600 MHz, $CDCl_3$) δ 7.40–7.35 (m, 2H, ArH), 6.89–6.79 (m, 3H, ArH and exchangeable NH), 5.59 (s, 1H, H8), 3.79 (s, 3H, OCH_3), 2.29 (tt, J = 6.4, 3.4 Hz, 1H, H22), 2.02-1.95 (m, 2H, H12 and H11), 1.91–1.58 (m, 16H, BCHep-CH_2), 1.56–1.40 (m, 4H, BCHep-CH_2), 1.27–1.23 (m, 1H, H11'); **^{13}C NMR** (151 MHz, $CDCl_3$) δ 171.4, 156.6, 131.1, 122.1, 114.3, 81.1, 55.7, 53.6, 46.6, 44.9, 40.4, 39.7, 37.2, 37.1, 36.7, 36.2, 33.1, 31.2, 29.9, 28.7, 27.8, 17.2, 16.8; **HRMS** (ESI^+) calc. for $C_{25}H_{33}Cl_2NO_2$ $[M+H]^+$450.1961, found 420.1961.

2-(bicyclo[3.1.1]heptan-1-yl)-3-(5-(dichloromethyl)bicyclo[3.1.1]heptan-1-yl)-*N*-(2-methyl-4-oxopentan-2-yl)propenamide (6c)

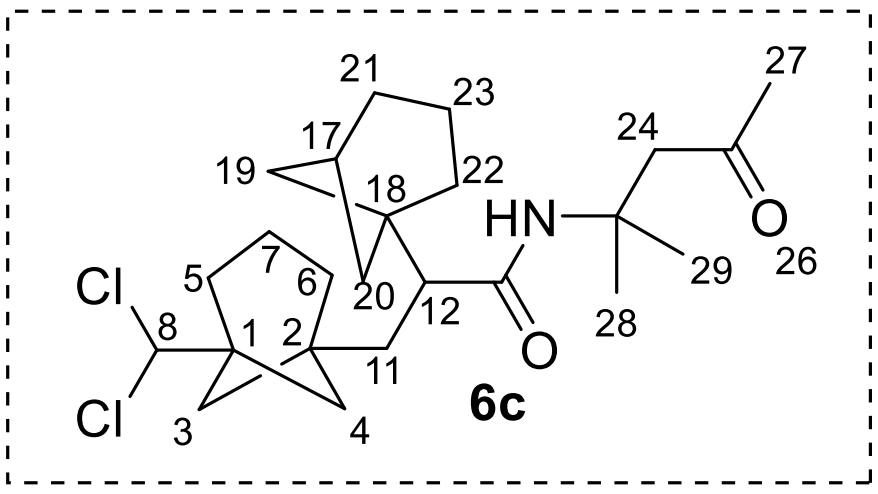

Synthesized according to *General Procedure A* using acrylamide **2n** (16.9 mg, 0.1 mmol, 1.0 equiv.) and **5** (0.2 mmol, 2.0 equiv.). Purification is through flash column chromatography (0-35% Et_2O/Hex) to give product **6c** (20.4 mg, 0.046 mmol, 46%) as a colorless oil.

R*f* 0.25 (30% Et_2O/hexane); **^{1}H NMR** (600 MHz, $CDCl_3$) δ 5.73 (s, 1H, exchangeable NH), 5.61 (s, 1H, H8), 2.98 (d, *J* = 17.0 Hz, 1H, H24), 2.79 (d, *J* = 17.0 Hz, 1H, H24'), 2.24 (tt, *J* = 5.9, 2.8 Hz, 1H, H17), 2.12 (s, 3H, H27), 1.88–1.53 (m,18H, H11, H12 and BCHep-CH_2), 1.48–1.41(m, 4H, BCHep-CH_2), 1.40 (s, 3H, CH_3), 1.38 (s, 3H, CH_3), 1.08 (d, *J* = 12.2 Hz, 1H, H11'); **^{13}C NMR** (151 MHz, $CDCl_3$) δ 208.3, 172.5, 81.3, 53.4, 52.2, 51.5, 46.5, 44.8, 40.4, 39.5, 37.2, 37.1, 36.7, 36.5, 33.2, 32.0, 30.7, 29.9, 29.8, 28.8, 27.6, 27.5, 17.1, 16.9; **HRMS** (ESI^+) calc. for $C_{24}H_{37}Cl_2NO_2$ $[M+H]^+$ 442.2274, found 442.2272.

2-(bicyclo[3.1.1]heptan-1-yl)-3-(5-(dichloromethyl)bicyclo[3.1.1]heptan-1-yl)-1-morpholinopropan-1-one (6d)

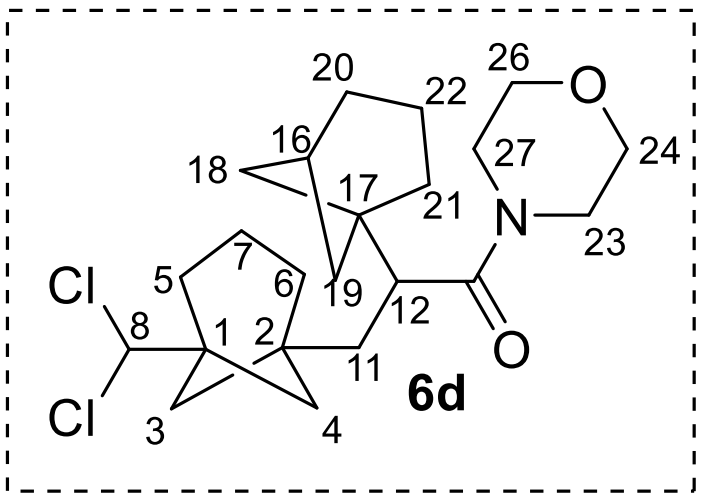

Synthesized according to *General Procedure A* using acrylamide **2p** (14.1 mg, 0.1 mmol, 1.0 equiv.) and **5** (0.2 mmol, 2.0 equiv.). Purification is through flash column chromatography (0-40% Et_2O/Hex) to give product **6d** (20.7 mg, 0.05 mmol, 50%) as a colorless oil.

R*f* 0.24 (35% Et_2O/hexane); **^{1}H NMR** (600 MHz, $CDCl_3$) δ 5.58 (s, 1H, H8), 3.78–3.61 (m, 5H, morpholine CH_2), 3.55–3.46 (m, 3H, morpholine CH_2), 2.45 (dd, *J* = 10.8, 1.7 Hz, 1H, H12), 2.23 (tt, *J* = 6.4, 3.2 Hz, 1H, H16), 2.01 (dd, *J* = 13.7, 10.8 Hz, 1H, H11), 1.90–1.58 (m, 16H, BCHep-CH_2), 1.47–1.30 (m, 4H, BCHep-CH_2), 1.27-1.23 (m, 1H, H11'); **^{13}C NMR** (151 MHz, $CDCl_3$) δ 172.6, 81.0, 67.3, 66.8, 46.6, 46.6, 44.7, 44.1, 42.2, 40.4, 39.7, 38.0, 37.0, 36.7, 33.9, 33.4, 33.1, 30.1, 29.9, 28.6, 27.8, 17.2, 17.0; **HRMS** (ESI^+) calc. for $C_{22}H_{33}Cl_2NO_2$ $[M+H]^+$ 414.1961, found 414.1960.

2-(bicyclo[3.1.1]heptan-1-yl)-3-(5-(dichloromethyl)bicyclo[3.1.1]heptan-1-yl)-2-methyl-*N*-phenylpropanamide (6e)

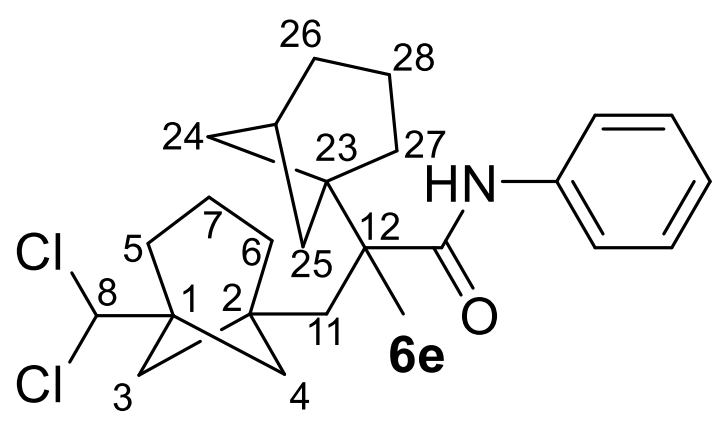

Synthesized according to *General Procedure A* using acrylamide **2q** (16.1 mg, 0.1 mmol, 1.0 equiv.) and **5** (0.2 mmol, 2.0 equiv.). Purification is through flash column chromatography (0-20% Et_2O/Hex) to give product **6e** (18.7 mg, 0.043 mmol, 43%) as a colorless oil.

R*f* 0.32 (20% Et_2O/hexane);**^{1}H NMR** (600 MHz, $CDCl_3$) δ 7.49–7.43 (m, 2H, ArH), 7.33 (dd, *J* = 8.6, 7.4 Hz, 2H, ArH), 7.13–7.09 (m, 1H, ArH), 7.04 (s, 1H, exchangeable NH), 5.60 (s, 1H, H8), 2.25 (tt, *J* = 6.7, 3.0 Hz, 1H, H22), 2.18–2.11 (m, 2H, H11 and BCHep-CH_2), 2.05-2.00 (m,1H, BCHep-CH_2), 1.89–1.60 (m, 14H, BCHep-CH_2), 1.60–1.54 (m, 2H, BCHep-CH_2 overlap with water peak), 1.51 (d, *J* = 14.3 Hz, 1H, H11'), 1.35–1.30 (m, 2H, BCHep-CH_2), 1.06 (s, 3H, CH_3); **^{13}C NMR** (151 MHz, $CDCl_3$) δ 174.6, 138.0, 129.2, 124.3, 120.2, 80.9, 49.8, 48.6, 47.3, 45.4, 43.8, 41.5, 37.0, 33.8, 31.8, 31.0, 30.4, 28.7, 28.7, 28.2, 17.2, 17.2, 16.1; **HRMS** (ESI^+) calc. for $C_{25}H_{33}Cl_2NO$ $[M+H]^+$434.2012, found 434.2011.

benzyl 2-(bicyclo[1.1.1]pentan-1-yl)-3-(3-(dichloromethyl)bicyclo[1.1.1]pentan-1-yl)propanoate (3aa)

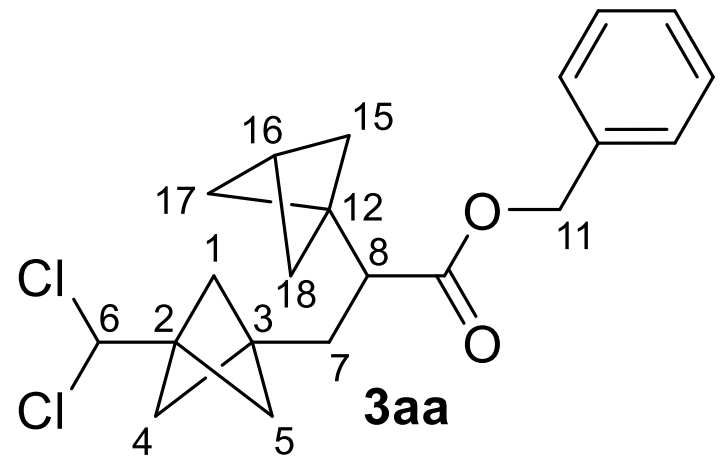

Synthesized according to *General Procedure A* using acrylamide **2aa** (16.2 mg, 0.1 mmol, 1.0 equiv.) and **1** (0.2 mmol, 2.0 equiv.). Purification is through flash column chromatography (0-10% Et_2O/Hex) to give product **6e** (17.1 mg, 0.045 mmol, 45%) as a colorless oil.

R*f* 0.7 (18% Et_2O/hexane);**^{1}H NMR** (600 MHz, $CDCl_3$) δ 7.40–7.30 (m, 5H, ArH), 5.69 (s, 1H, H6), 5.14–5.07 (m, 2H, H11), 2.49 (dd, *J* = 11.9, 2.9 Hz, 1H, H8), 2.45 (s, 1H, H16), 1.96 (dd, *J* = 14.3, 11.9 Hz, 1H, H7), 1.68–1.57 (m, 13H, H1,H4,H5,H15,H17,H18 and H7'); **^{13}C NMR** (151 MHz, $CDCl_3$) δ 173.3, 136.0, 128.8, 128.7, 128.5, 72.0, 66.4, 49.5, 48.9, 45.9, 45.1, 44.3, 37.6, 30.3, 26.8; **HRMS** (ESI^+) calc. for $C_{21}H_{24}Cl_2O_2$ $[M+H]^+$379.1226, found 379.1217.

2-(bicyclo[1.1.1]pentan-1-yl)-3-(3-(dichloromethyl)bicyclo[1.1.1]pentan-1-yl)propanenitrile (3ab)

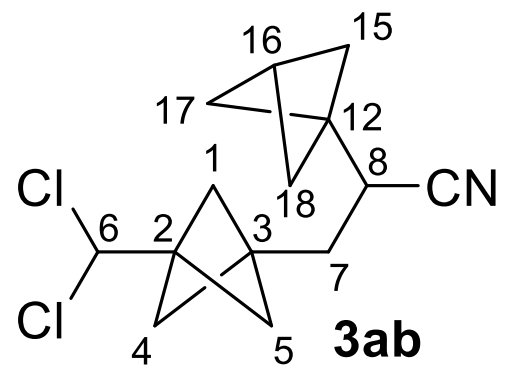

3ab

Synthesized according to *General Procedure A* using acrylamide **2ab** (5.3 mg, 0.1 mmol, 1.0 equiv.) and **1** (0.2 mmol, 2.0 equiv.). Purification is through flash column chromatography (0-10% Et_2O/Hex) to give product **6e** (14.9 mg, 0.055 mmol, 55%) as a white solid.

R_f 0.4 (10% Et_2O/hexane); **^{1}H NMR** (600 MHz, $CDCl_3$) δ 5.76 (s, 1H, H6), 2.59 (s, 1H, H16), 2.54 (dd, *J* = 11.7, 4.0 Hz, 1H, H8), 1.88–1.76 (m, 13H, H1,H4,H5,H15,H17,H18 and H7), 1.72 (dd, *J* = 14.3, 4.0 Hz, 1H, H7'); **^{13}C NMR** (151 MHz, $CDCl_3$) δ 120.2, 71.7, 49.2, 49.0, 44.6, 43.4, 37.1, 32.0, 31.1, 27.1; **HRMS** (ESI^+) calc. for $C_{14}H_{17}Cl_2N[M+H]^+$ 270.0811, found 270.0806.

1-(2-(bicyclo[1.1.1]pentan-1-yl)-2-(phenylsulfonyl)ethyl)-3-(dichloromethyl)bicyclo[1.1.1]pentane (3ac)

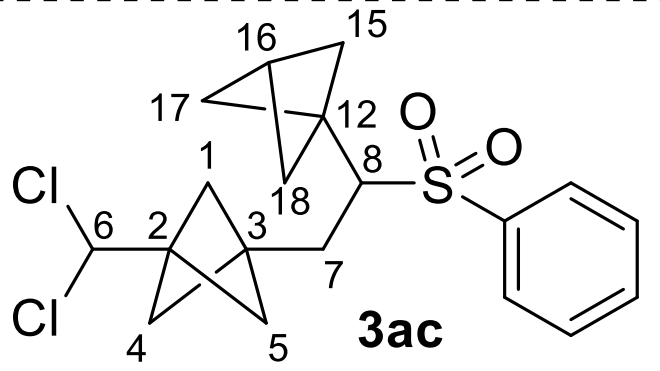

3ac

Synthesized according to *General Procedure A* using acrylamide **2ac** (16.8 mg, 0.1 mmol, 1.0 equiv.) and **1** (0.2 mmol, 2.0 equiv.). Purification is through flash column chromatography (0-20% Et_2O/Hex) to give product **6e** (12.3 mg, 0.032 mmol, 32%) as a colorless oil.

R_f 0.27 (20% Et_2O/hexane); **^{1}H NMR** (600 MHz, $CDCl_3$) δ 7.88 (d, *J* = 7.6 Hz, 2H, ArH), 7.65 (t, *J* = 7.2 Hz, 1H, ArH), 7.57 (t, *J* = 7.7 Hz, 2H, ArH), 5.67 (s, 1H, H6), 3.02 (dd, *J* = 6.2, 2.6 Hz, 1H, H8), 2.49 (s, 1H, H16), 2.07 (dd, *J* = 15.6, 2.7 Hz, 1H, H7), 1.89-1.84 (m, 7H, H15, H17,H18 and H7'), 1.50 (ddd, *J* = 48.2, 9.5, 1.9 Hz, 6H, H1,H4,H5); **^{13}C NMR** (151 MHz, $CDCl_3$) δ 139.3, 133.8, 129.3, 128.7, 71.7, 62.4, 50.9, 48.6, 44.5, 41.9, 37.4, 29.0, 28.2; **HRMS** (ESI^+) calc. for $C_{19}H_{22}Cl_2O_2S[M+H]^+$ 385.0790, found 385.0788.

2-(bicyclo[1.1.1]pentan-1-yl)-N-phenyl-3-(3-(trichloromethyl)bicyclo[1.1.1]pentan-1-yl)propenamide (4a)

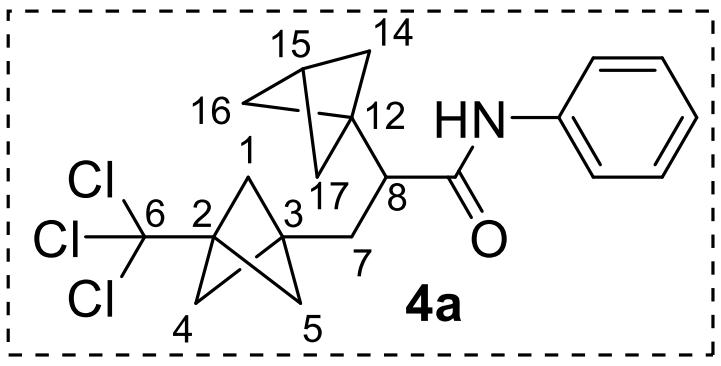

Synthesized according to *General Procedure B* using acrylamide **2a** (14.7 mg, 0.1 mmol, 1.0 equiv.) and **1** (0.2 mmol, 2.0 equiv.). Purification is through flash column chromatography (0-20% Et_2O/Hex) to give product **4a** (19.9 mg, 0.05 mmol, 50%) as a white solid.

R_f 0.3 (20% Et_2O/hexane); **^{1}H NMR** (600 MHz, $CDCl_3$) δ 7.52–7.46 (m, 2H, ArH), 7.37–7.30 (m, 2H, ArH), 7.13 (ddt, *J* = 8.6, 7.4, 1.2 Hz, 1H, ArH), 7.02 (s, 1H, exchangeable NH), 2.52 (s, 1H, H15), 2.34 (dd, *J* = 11.6, 2.8 Hz, 1H, H8), 2.15 (dd, *J* = 14.3, 11.5 Hz, 1H, H7), 1.89–1.79 (m, 6H, BCP-CH_2), 1.78–1.72 (m, 6H, BCP-CH_2), 1.66 (dd, *J* = 14.4, 2.8 Hz, 1H, H7'); **^{13}C NMR** (151 MHz, $CDCl_3$) δ 170.5, 137.8, 129.2, 124.6, 120.2, 98.1, 51.8, 49.5, 48.5, 45.6, 35.7, 29.6, 26.9; **HRMS** (ESI^+) calc. for $C_{20}H_{22}Cl_3NO$ $[M+H]^+$ 398.0840, found 398.0844.

2-(bicyclo[1.1.1]pentan-1-yl)-*N*-(4-fluorophenyl)-3-(3-(trichloromethyl)bicyclo[1.1.1]pentan-1-yl)propenamide (4b)

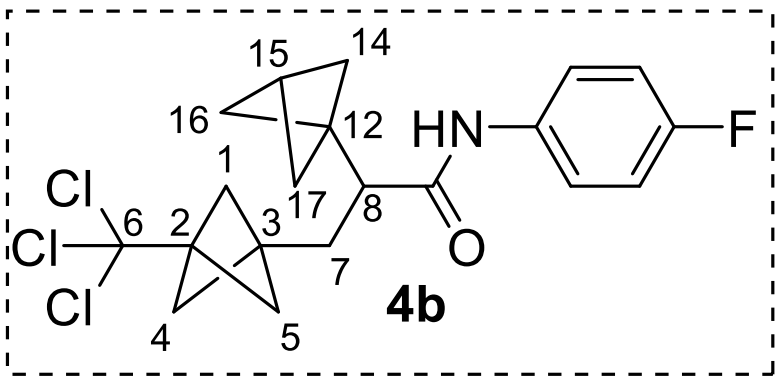

Synthesized according to *General Procedure B* using acrylamide **2b** (16.5 mg, 0.1 mmol, 1.0 equiv.) and **1** (0.2 mmol, 2.0 equiv.). Purification is through flash column chromatography (0-20% Et_2O/Hex) to give product **4b** (12.5 mg, 0.03 mmol, 30%) as a white solid.

R_f 0.28 (20% Et_2O/hexane); **^{1}H NMR** (600 MHz, $CDCl_3$) δ 7.47–7.41 (m, 2H, ArH), 7.06–7.00 (m, 2H, ArH), 6.96 (s, 1H, exchangeable NH), 2.53 (s, 1H, H15), 2.32 (dd, *J* = 11.5, 2.8 Hz, 1H, H8), 2.14 (dd, *J* = 14.4, 11.5 Hz, 1H, H7), 1.89–1.80 (m, 6H, BCP-CH_2), 1.78–1.71 (m, 6H, BCP-CH_2), 1.66 (dd, *J* = 14.4, 2.8 Hz, 1H, H7'); **^{13}C NMR** (151 MHz, $CDCl_3$) δ 170.5, 159.6 (d, *J* = 243.8 Hz), 133.7 (d, *J* = 2.8 Hz), 122.0 (d, *J* = 7.8 Hz), 115.9 (d, *J* = 22.5 Hz), 115.8, 98.0, 51.8, 49.5, 49.5, 48.4, 45.5, 35.7, 29.6, 26.9; **HRMS** (ESI^+) calc. for $C_{20}H_{21}Cl_3FNO$ $[M-H]^-$ 414.0589, found 414.06041; $[M+COOH]^-$ 460.0644, found 460.0661

2-(bicyclo[1.1.1]pentan-1-yl)-*N*-(2-bromophenyl)-3-(3-(trichloromethyl)bicyclo[1.1.1]pentan-1-yl)propenamide (4c)

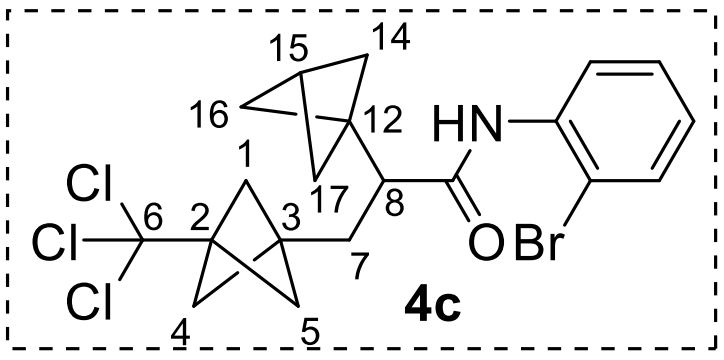

Synthesized according to *General Procedure B* using acrylamide **2f** (22.6 mg, 0.1 mmol, 1.0 equiv.) and **1** (0.2 mmol, 2.0 equiv.). Purification is through flash column chromatography (0-15% Et_2O/Hex) to give product **4c** (18.2 mg, 0.038 mmol, 38%) as a white solid.

R*f* 0.4 (15% Et_2O/hexane); **^{1}H NMR** (600 MHz, $CDCl_3$) δ 7.55 (dd, *J* = 8.0, 1.5 Hz, 1H, ArH), 7.50 (s, 1H, exchangeable NH), 7.33 (ddd, *J* = 8.5, 7.3, 1.5 Hz, 1H, ArH), 7.00 (ddd, *J* = 8.1, 7.4, 1.6 Hz, 1H, ArH), 2.54 (s, 1H, H15), 2.43 (dd, *J* = 11.7, 2.8 Hz, 1H, H8), 2.14 (dd, *J* = 14.5, 11.7 Hz, 1H, H7), 1.91–1.75 (m, 12H, BCP-CH_2), 1.70 (dd, *J* = 14.4, 2.9 Hz, 1H, H7'); **^{13}C NMR** (151 MHz, $CDCl_3$) δ 170.6, 135.6, 132.4, 128.6, 125.4, 122.3, 113.5, 98.0, 51.8, 49.5, 49.5, 49.0, 45.3, 35.7, 29.8, 26.9; **HRMS** (ESI$^+$) calc. for $C_{20}H_{21}BrCl_3NO$ $[M-H]^-$ 473.9788, found 473.9804.

2-(bicyclo[1.1.1]pentan-1-yl)-*N*-(3,5-dimethylphenyl)-3-(3-(trichloromethyl)bicyclo[1.1.1]pentan-1-yl)propenamide (4d)

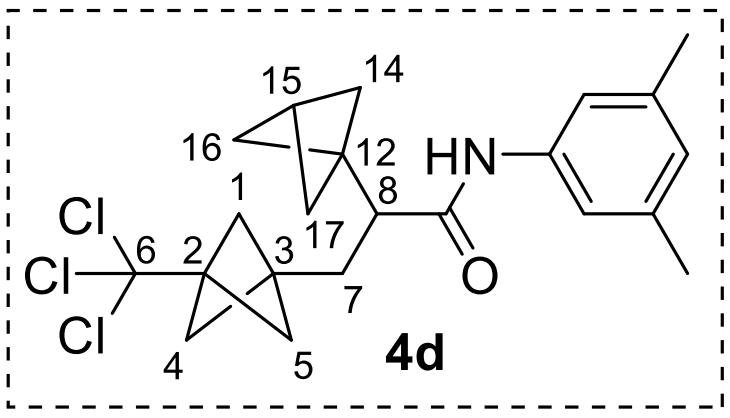

Synthesized according to *General Procedure B* using acrylamide **2d'** (17.5 mg, 0.1 mmol, 1.0 equiv.) and **1** (0.2 mmol, 2.0 equiv.). Purification is through flash column chromatography (0-20% Et_2O/Hex) to give product **4d** (17.1 mg, 0.04 mmol, 40%) as a white solid.

R*f* 0.32 (20% Et_2O/hexane); **^{1}H NMR** (600 MHz, $CDCl_3$) δ 7.14 (s, 2H, ArH), 6.95 (s, 1H, exchangeable NH), 6.77 (s, 1H, ArH), 2.52 (s, 1H, H15), 2.33-2.27 (m, 7H, CH_3 and H8), 2.14 (dd, *J* = 14.3, 11.6 Hz, 1H, H7), 1.89–1.79 (m, 6H, BCP-CH_2), 1.78–1.71 (m, 6H, BCP-CH_2), 1.65 (dd, *J* = 14.3, 2.8 Hz, 1H, H7'); **^{13}C NMR** (151 MHz, $CDCl_3$) δ 170.3, 139.0, 137.7, 126.3, 117.7, 98.1, 51.8, 49.5, 48.5, 45.6, 35.7, 29.6, 26.8, 21.5; **HRMS** (ESI$^+$) calc. for $C_{22}H_{26}Cl_3NO$ $[M+H]^+$ 426.1153, found 426.1152.

2-(bicyclo[1.1.1]pentan-1-yl)-*N*-(4-methoxyphenyl)-3-(3-(trichloromethyl)bicyclo[1.1.1]pentan-1-yl)propenamide (4e)

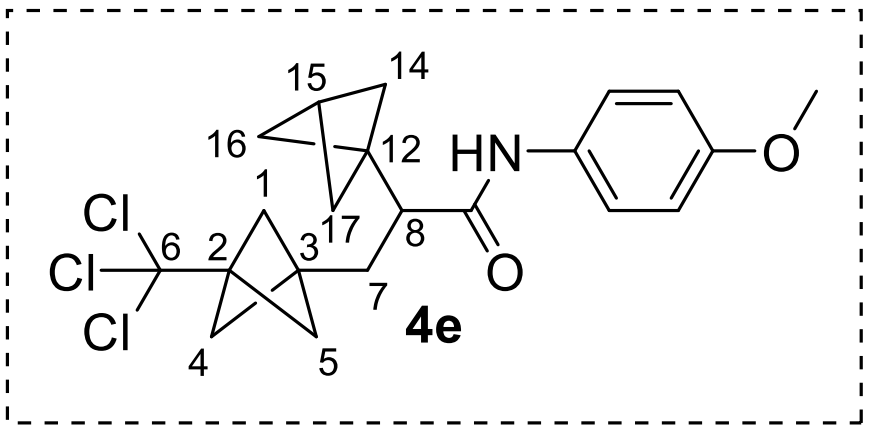

Synthesized according to *General Procedure B* using acrylamide **2e** (17.7 mg, 0.1 mmol, 1.0 equiv.) and **1** (0.2 mmol, 2.0 equiv.). Purification is through flash column chromatography (0-30% Et_2O/Hex) to give product **4d** (15.4 mg, 0.036 mmol, 36%) as a white solid.

R*f* 0.24 (25% Et_2O/hexane);**^{1}H NMR** (600 MHz, $CDCl_3$) δ 7.42–7.37 (m, 2H, ArH), 6.92 (s, 1H, exchangeable NH), 6.90–6.83 (m, 2H, ArH), 3.79 (s, 3H. OCH_3), 2.52 (s, 1H, H15), 2.31 (dd, *J* = 11.6, 2.8 Hz, 1H, H8), 2.14 (dd, *J* = 14.3, 11.6 Hz, 1H, H7), 1.90–1.80 (m, 6H, BCP-CH_2), 1.78–1.71 (m, 6H, BCP-CH_2), 1.65 (dd, *J* = 14.3, 2.8 Hz, 1H, H7'); **^{13}C NMR** (151 MHz, $CDCl_3$) δ 170.3, 156.7, 130.9, 122.1, 114.4, 98.1, 55.7, 51.8, 49.5, 48.3, 45.6, 35.7, 29.6, 26.8; **HRMS** (ESI$^+$) calc. for $C_{21}H_{24}Cl_3NO_2$ $[M+H]^+$428.0945, found 428.0946.

2-(bicyclo[1.1.1]pentan-1-yl)-*N*-(p-tolyl)-3-(3-(trichloromethyl)bicyclo[1.1.1]pentan-1-yl)propenamide (4f)

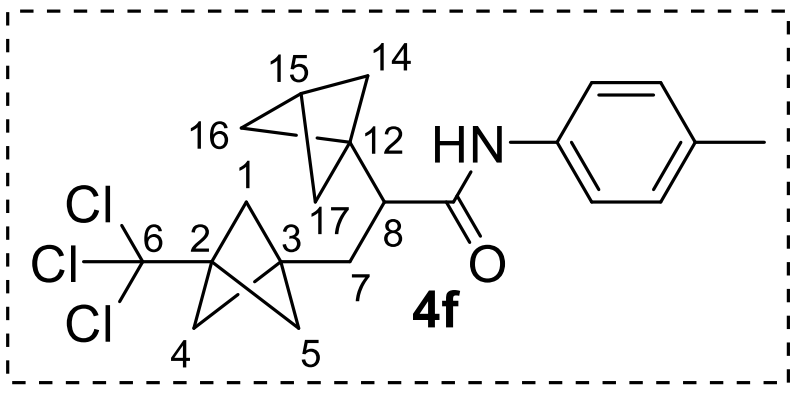

Synthesized according to *General Procedure B* using acrylamide **2c** (16.1 mg, 0.1 mmol, 1.0 equiv.) and **1** (0.2 mmol, 2.0 equiv.). Purification is through flash column chromatography (0-20% Et_2O/Hex) to give product **4f** (17.7 mg, 0.043 mmol, 43%) as a white solid.

R*f* 0.3 (20% Et_2O/hexane);**^{1}H NMR** (600 MHz, $CDCl_3$) δ 7.43–7.37 (m, 2H, ArH), 7.19–7.14 (m, 2H, ArH), 7.00 (s, 1H, exchangeable NH), 2.54 (s, 1H, H15), 2.37-2.33 (m, 4H, CH_3 and H8), 2.17 (dd, *J* = 14.4, 11.6 Hz, 1H, H7), 1.92–1.82 (m, 6H, BCP-CH_2), 1.80–1.74 (m, 6H, BCP-CH_2), 1.67 (dd, *J* = 14.4, 2.8 Hz, 1H, H7'); **^{13}C NMR** (151 MHz, $CDCl_3$) δ 170.3, 135.2, 134.3, 129.7, 120.3, 98.1, 51.8, 49.5, 48.4, 45.6, 35.7, 29.6, 26.8, 21.0; **HRMS** (ESI$^+$) calc. for $C_{21}H_{24}Cl_3NO$ $[M+H]^+$412.0996, found 412.0995.

2-(bicyclo[1.1.1]pentan-1-yl)-*N*-(4-phenoxyphenyl)-3-(3-(trichloromethyl)bicyclo[1.1.1]pentan-1-yl)propenamide (4g)

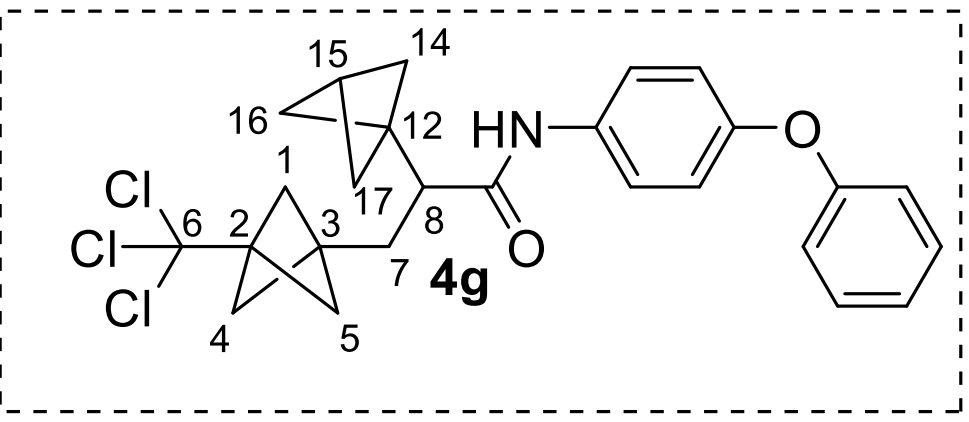

Synthesized according to *General Procedure B* using acrylamide **2g** (23.9 mg, 0.1 mmol, 1.0 equiv.) and **1** (0.2 mmol, 2.0 equiv.). Purification is through flash column chromatography (0-30% Et_2O/Hex) to give product **4g** (18.2 mg, 0.037 mmol, 37%) as a white solid.

$\mathbf{R_f}$ 0.23 (20% Et_2O/hexane);^{1}**H NMR** (600 MHz, $CDCl_3$) δ 7.48–7.42 (m, 2H, ArH), 7.35–7.30 (m, 2H, ArH), 7.09 (t, *J* = 7.4 Hz, 1H, ArH), 7.04–6.96 (m, 5H, ArH and exchangeable NH), 2.53 (s, 1H, H15), 2.34 (dd, *J* = 11.6, 2.8 Hz, 1H, H8), 2.15 (dd, *J* = 14.4, 11.5 Hz, 1H, H7), 1.90– 1.81 (m, 6H, BCP-CH_2), 1.79–1.72 (m, 6H, BCP-CH_2), 1.66 (dd, *J* = 14.4, 2.8 Hz, 1H, H7'); ^{13}**C NMR** (151 MHz, $CDCl_3$) δ 170.4, 157.6, 153.8, 133.2, 129.9, 123.3, 121.9, 119.8, 118.6, 98.1, 51.8, 49.5, 48.4, 45.6, 35.7, 29.6, 26.9; **HRMS** (ESI^+) calc. for $C_{26}H_{26}Cl_3NO_2$ $[M+H]^+$490.1102, found 490.1107.

2-(bicyclo[1.1.1]pentan-1-yl)-*N*-cyclopropyl-3-(3-(trichloromethyl)bicyclo[1.1.1]pentan-1-yl)propenamide (4h)

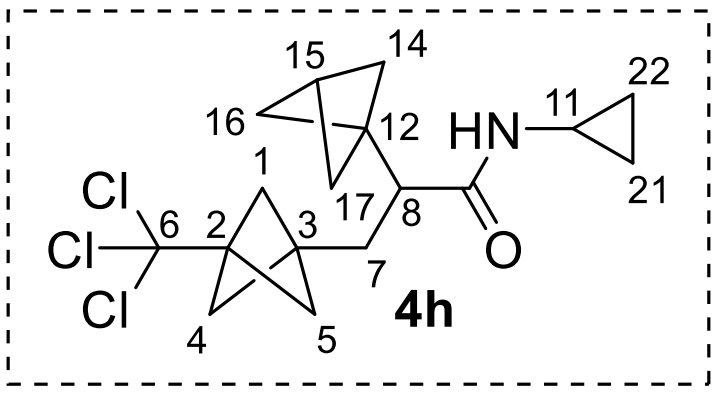

Synthesized according to *General Procedure B* using acrylamide **2l** (11.1 mg, 0.1 mmol, 1.0 equiv.) and **1** (0.2 mmol, 2.0 equiv.). Purification is through flash column chromatography (0-30% Et_2O/Hex) to give product **4g** (15.2 mg, 0.042 mmol, 42%) as a white solid.

$\mathbf{R_f}$ 0.3 (30% Et_2O/hexane);^{1}**H NMR** (600 MHz, $CDCl_3$) δ 5.44 (s, 1H, exchangeable NH), 2.70 (dddd, *J* = 10.9, 7.0, 3.8, 2.8 Hz, 1H, H11), 2.48 (s, 1H, H15), 2.10 (dd, *J* = 11.6, 2.5 Hz, 1H, H8), 2.03 (dd, *J* = 13.9, 11.5 Hz, 1H, H7), 1.85–1.73 (m, 6H, BCP-CH_2), 1.71-1.60 (m, 6H, BCP-CH_2), 1.55 (dd, *J* = 14.0, 2.6 Hz, 1H, H7'), 0.81-0.76 (m, 2H, H22), 0.50 – 0.46 (m, 2H, H21); ^{13}**C NMR** (151 MHz, $CDCl_3$) δ 173.4, 98.2, 51.7, 49.4, 49.4, 47.3, 45.6, 35.7, 29.4, 26.7, 22.7, 7.0, 7.0; **HRMS** (ESI^+) calc. for $C_{17}H_{22}Cl_3NO$ $[M+H]^+$362.0840, found 362.0839.

2-(bicyclo[1.1.1]pentan-1-yl)-*N*-cyclohexyl-3-(3-(trichloromethyl)bicyclo[1.1.1]pentan-1-yl)propenamide (4i)

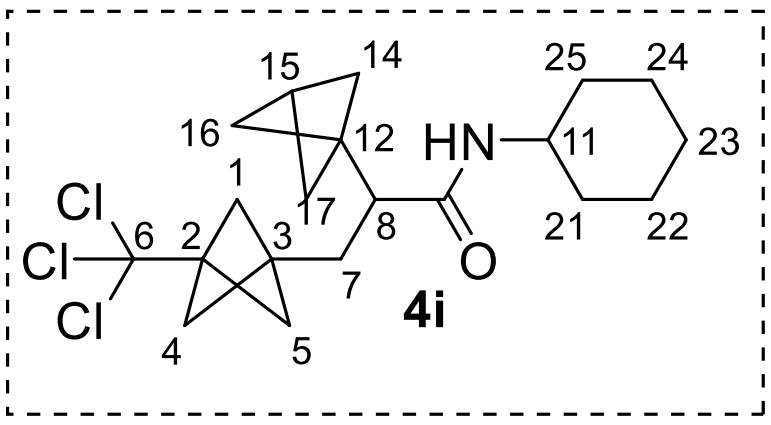

Synthesized according to *General Procedure B* using acrylamide **2m** (15.3 mg, 0.1 mmol, 1.0 equiv.) and **1** (0.2 mmol, 2.0 equiv.). Purification is through flash column chromatography (0-30% Et_2O/Hex) to give product **4i** (17.8 mg, 0.044 mmol, 44%) as a white solid.

R*f* 0.4 (28% Et_2O/hexane);**^{1}H NMR** (600 MHz, $CDCl_3$) δ 5.08 (d, *J* = 8.3 Hz, 1H, exchangeable NH), 3.78–3.69 (m, 1H, H11), 2.41 (s, 1H, H15), 2.04 (dd, *J* = 11.6, 2.7 Hz, 1H, H8), 1.95 (dd, *J* = 14.1, 11.6 Hz, 1H, H7), 1.88–1.82 (m, 2H, cyclohex-CH_2), 1.78–1.70 (m, 6H, BCP-CH_2), 1.67–1.52 (m, 10H, BCP-CH_2 and cyclohex-CH_2 overlap with water peak), 1.49 (dd, *J* = 14.1, 2.7 Hz, 1H, H7'), 1.31 (dtt, *J* = 13.6, 12.0, 3.6 Hz, 2H, cyclohex-CH_2), 1.14–1.01 (m, 2H, cyclohex-CH_2); **^{13}C NMR** (151 MHz, $CDCl_3$) δ 170.9, 98.2, 51.7, 49.5, 49.4, 47.7, 45.7, 35.8, 33.8, 33.5, 29.4, 26.6, 25.7, 25.1; **HRMS** (ESI^+) calc. for $C_{20}H_{28}Cl_3NO$ $[M+H]^+$404.1309, found 404.1308.

2-(bicyclo[1.1.1]pentan-1-yl)-*N*-(2-methyl-4-oxopentan-2-yl)-3-(3-(trichloromethyl)bicyclo[1.1.1]pentan-1-yl)propenamide (4j)

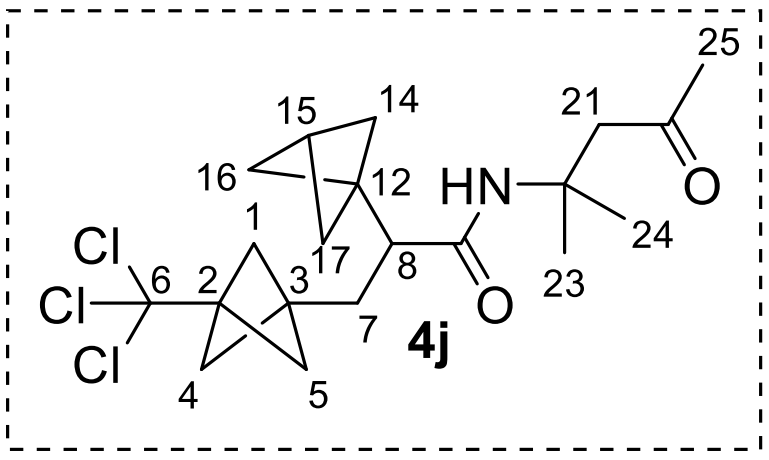

Synthesized according to *General Procedure B* using acrylamide **2n** (16.9 mg, 0.1 mmol, 1.0 equiv.) and **1** (0.2 mmol, 2.0 equiv.). Purification is through flash column chromatography (0-35% Et_2O/Hex) to give product **4i** (17.7 mg, 0.042 mmol, 42%) as a white solid.

R*f* 0.25 (28% Et_2O/hexane);**^{1}H NMR** (600 MHz, $CDCl_3$) δ 5.85 (s, 1H, exchangeable NH), 2.94–2.83 (m, 2H, H21), 2.46 (s, 1H, H15), 2.14 (s, 3H, H25), 2.08 (dd, *J* = 11.6, 2.6 Hz, 1H, H8), 1.99 (dd, *J* = 14.1, 11.6 Hz, 1H, H7), 1.84–1.62 (m, 12H, BCP-CH_2), 1.50 (dd, *J* = 14.1, 2.6 Hz, 1H, H7'), 1.42 (d, *J* = 10.5 Hz, 6H, H23 and H24); **^{13}C NMR** (151 MHz, $CDCl_3$) δ 208.3, 171.6, 98.3, 52.4, 51.7, 51.6, 49.4, 49.4, 47.9, 45.7, 35.7, 32.1, 29.3, 27.6, 27.4, 26.6; **HRMS** (ESI^+) calc. for $C_{20}H_{28}Cl_3NO_2$ $[M+H]^+$420.1258, found 420.1256.

2-(bicyclo[1.1.1]pentan-1-yl)-1-morpholino-3-(3-(trichloromethyl)bicyclo[1.1.1]pentan-1-yl)propan-1-one (4k)

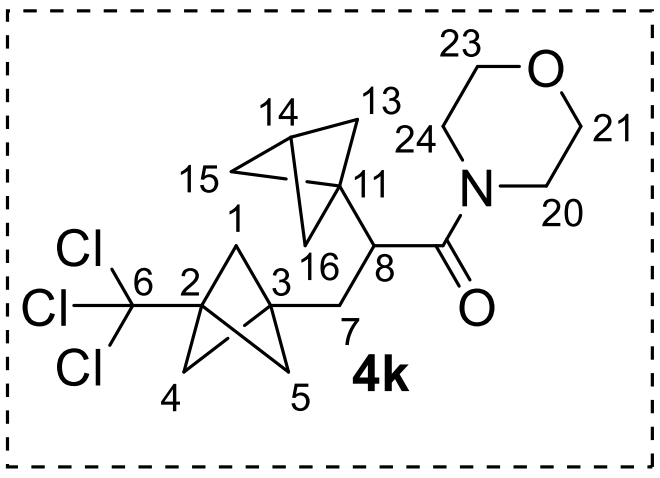

Synthesized according to *General Procedure B* using acrylamide **2p** (14.1 mg, 0.1 mmol, 1.0 equiv.) and **1** (0.2 mmol, 2.0 equiv.). Purification is through flash column chromatography (0-35% Et_2O/Hex) to give product **4i** (18.9 mg, 0.048 mmol, 48%) as a white solid.

R*f* 0.25 (30% Et_2O/hexane); **^{1}H NMR** (600 MHz, $CDCl_3$) δ 3.75–3.51 (m, 8H, morpholine CH_2), 2.74 (dd, *J* = 11.3, 2.8 Hz, 1H, H8), 2.49 (s, 1H, H14), 2.23 (dd, *J* = 13.9, 11.3 Hz, 1H, H7), 1.79–1.68 (m, 12H, BCP-CH_2), 1.56 (dd, *J* = 13.9, 2.8 Hz, 1H, H7'); **^{13}C NMR** (151 MHz, $CDCl_3$) δ 170.9, 98.1, 67.3, 66.9, 51.6, 49.6, 49.5, 46.3, 45.3, 42.2, 40.6, 35.8, 29.8, 27.2; **HRMS** (ESI$^+$) calc. for $C_{18}H_{24}Cl_3NO_2$ $[M+H]^+$392.0945, found 392.0946.

2-(bicyclo[3.1.1]heptan-1-yl)-*N*-phenyl-3-(5-(trichloromethyl)bicyclo[3.1.1]heptan-1-yl)propenamide (7a)

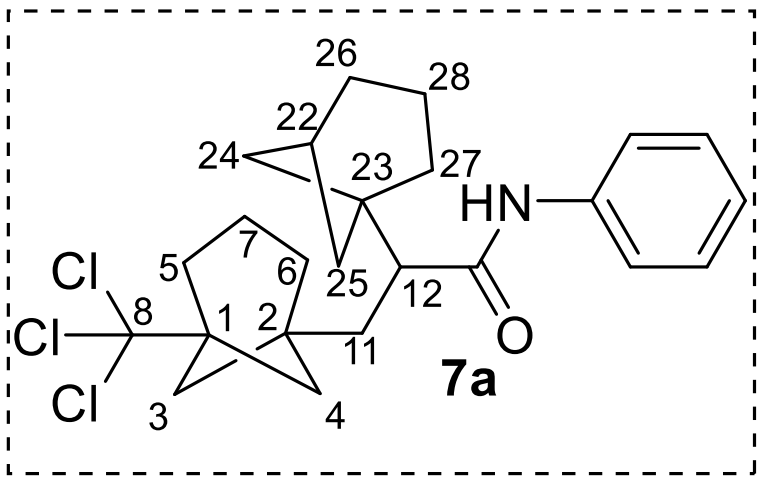

Synthesized according to *General Procedure B* using acrylamide **2a** (14.7 mg, 0.1 mmol, 1.0 equiv.) and **5** (0.2 mmol, 2.0 equiv.). Purification is through flash column chromatography (0-20% Et_2O/Hex) to give product **7a** (23.2 mg, 0.051 mmol, 51%) as a white solid.

R*f* 0.32 (20% Et_2O/hexane); **^{1}H NMR** (600 MHz, $CDCl_3$) δ 7.47 (dt, *J* = 8.8, 1.8 Hz, 2H, ArH), 7.31 (t, *J* = 7.8 Hz, 2H, ArH), 7.12–7.08 (m, 1H, ArH), 7.04 (s, 1H, exchangeable NH), 2.30 (tt, *J* = 6.4, 3.1 Hz, 1H, H22), 2.14 (dd, *J* = 9.6, 1.9 Hz, 1H, BCHep-CH_2), 2.08–2.02 (m, 4H, H12, H11 and, BCHep-CH_2), 1.94–1.65 (m, 11H, BCHep-CH_2), 1.64–1.47 (m, 6H, BCHep-CH_2), 1.31–1.28 (m, 1H, H11'); **^{13}C NMR** (151 MHz, $CDCl_3$) δ 171.5, 138.0, 129.2, 124.4, 120.2, 107.7, 55.0, 53.9, 45.0, 38.7, 38.4, 37.2, 36.7, 36.4, 35.7, 33.1, 31.2, 30.9, 29.9, 28.6, 17.8, 16.8; **HRMS** (ESI$^+$) calc. for $C_{24}H_{30}Cl_3NO$ $[M+H]^+$454.1466, found 454.1466.

2-(bicyclo[3.1.1]heptan-1-yl)-*N*-(4-methoxyphenyl)-3-(5-(trichloromethyl)bicyclo[3.1.1]heptan-1-yl)propenamide (7b)

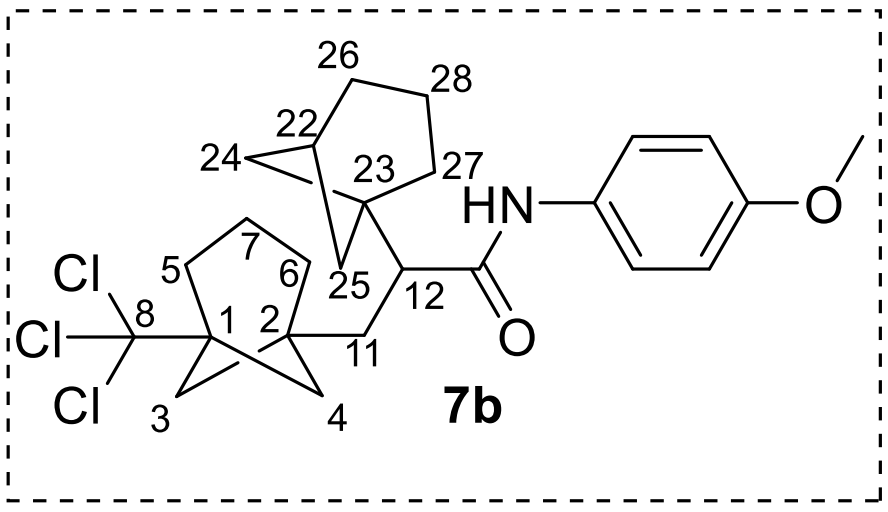

Synthesized according to *General Procedure B* using acrylamide **2e** (17.7 mg, 0.1 mmol, 1.0 equiv.) and **5** (0.2 mmol, 2.0 equiv.). Purification is through flash column chromatography (0-20% Et_2O/Hex) to give product **7b** (17.0 mg, 0.035 mmol, 35%) as a white solid.

R*f* 0.3 (18% Et_2O/hexane);**^{1}H NMR** (600 MHz, $CDCl_3$) δ 7.40–7.34 (m, 2H, ArH), 6.91–6.84 (m, 3H, ArH and exchangeable NH), 3.79 (s, 3H, OCH_3), 2.29 (tt, *J* = 6.3, 3.1 Hz, 1H, H22), 2.14 (dt, *J* = 9.2, 1.6 Hz, 1H, BCHep-CH_2), 2.09–2.02 (m, 4H, H12, H11 and, BCHep-CH_2), 1.92–1.64 (m, 11H, BCHep-CH_2), 1.62–1.48 (m, 6H, BCHep-CH_2 overlap with water peak), 1.30–1.27 (m, 1H, H11'); **^{13}C NMR** (151 MHz, $CDCl_3$) δ 171.4, 156.6, 131.1, 122.1, 114.3, 107.7, 55.7, 55.0, 53.7, 45.0, 38.6, 38.4, 37.2, 36.7, 36.3, 35.7, 33.1, 31.2, 30.9, 29.9, 28.7, 17.8, 16.8; **HRMS** (ESI^+) calc. for $C_{25}H_{32}Cl_3NO_2$ $[M+H]^+$484.1571, found 484.1571.

2-(bicyclo[3.1.1]heptan-1-yl)-*N*-(2-methyl-4-oxopentan-2-yl)-3-(5-(trichloromethyl)bicyclo[3.1.1]heptan-1-yl)propenamide (7c)

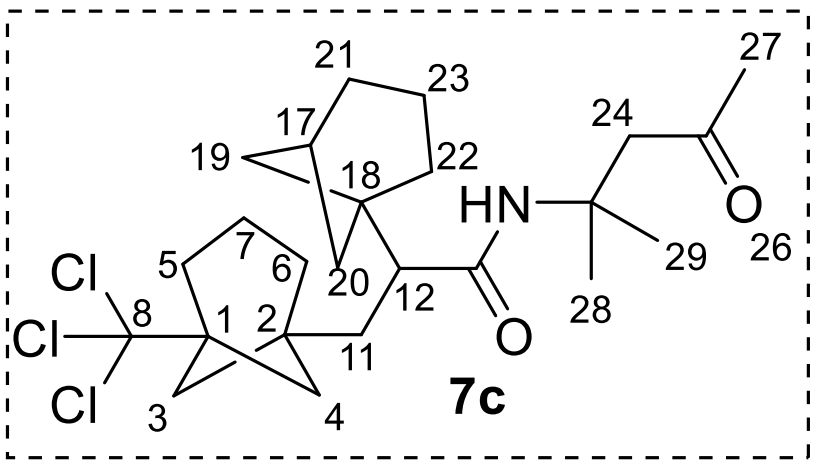

Synthesized according to *General Procedure B* using acrylamide **2n** (16.9 mg, 0.1 mmol, 1.0 equiv.) and **5** (0.2 mmol, 2.0 equiv.). Purification is through flash column chromatography (0-35% Et_2O/Hex) to give product **7c**(19.1 mg, 0.04 mmol, 40%) as a colorless oil.

R*f* 0.24 (28% Et_2O/hexane);**^{1}H NMR** (600 MHz, $CDCl_3$) δ 5.75 (s, 1H, exchangeable NH), 2.95 (d, *J* = 16.8 Hz, 1H, H17), 2.81 (d, *J* = 16.8 Hz, 1H, H17') 2.24 (tt, *J* = 6.4, 3.3 Hz, 1H, H18), 2.12 (s, 3H, H27), 2.08–2.05 (m, 2H, BCHep-CH_2), 1.98–1.42 (m, 20H, H12, H11 and BCHep-CH_2), 1.40 (s, 3H, H29), 1.39 (s, 3H, H28), 1.12 (dd, *J* = 13.7, 1.2 Hz, 1H, H11'); **^{13}C NMR** (151 MHz, $CDCl_3$) δ 208.2, 172.5, 107.8, 55.0, 53.5, 52.3, 51.6, 44.8, 38.8, 38.1, 37.2, 37.1, 36.3, 35.7, 33.0, 32.0, 30.9, 30.6, 29.8, 28.8, 27.5, 27.5, 17.8, 16.8; **HRMS** (ESI^+) calc. for $C_{24}H_{36}Cl_3NO_2$ $[M+H]^+$476.1884, found 484.1885.

2-(bicyclo[3.1.1]heptan-1-yl)-2-methyl-*N*-phenyl-3-(5-(trichloromethyl)bicyclo[3.1.1]heptan-1-yl)propenamide (7d)

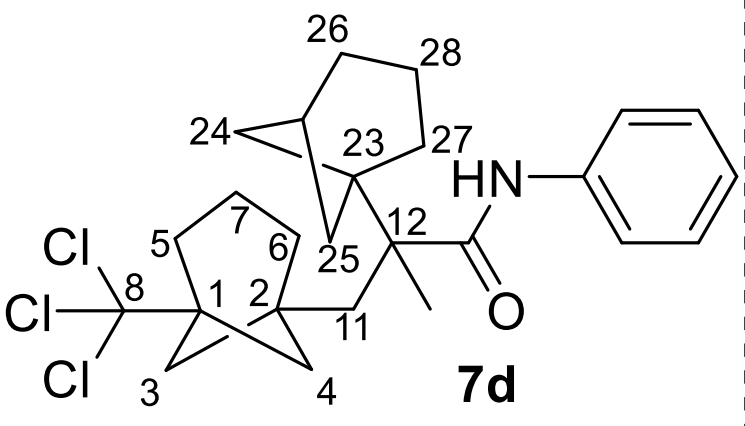

7d

Synthesized according to *General Procedure B* using acrylamide **2q** (16.1 mg, 0.1 mmol, 1.0 equiv.) and **5** (0.2 mmol, 2.0 equiv.). Purification is through flash column chromatography (0-20% Et_2O/Hex) to give product **7d** (17.8 mg, 0.038 mmol, 38%) as a white solid.

R_f 0.35 (20% Et_2O/hexane); **^{1}H NMR** (600 MHz, $CDCl_3$) δ 7.49–7.44 (m, 2H, ArH), 7.35–7.30 (m, 2H, ArH), 7.11 (tt, *J* = 7.4, 1.2 Hz, 1H, ArH), 7.06 (s, 1H, exchangeable NH), 2.26 (tt, *J* = 6.6, 3.0 Hz, 1H, H22), 2.22–2.01 (m, 7H, H11 and BCHep-CH_2), 1.85–1.54 (m, 13H, BCHep-CH_2), 1.36–1.31 (m, 2H, H11' and BCHep-CH_2), 1.07 (s, 3H, CH_3); **^{13}C** NMR (151 MHz, $CDCl_3$) δ 174.6, 137.9, 129.2, 124.4, 120.2, 107.5, 55.7, 49.8, 48.6, 45.2, 42.6, 40.2, 35.8, 33.9, 31.5, 31.0, 31.0, 30.4, 28.7, 28.6, 17.9, 17.2, 16.0; **HRMS** (ESI^+) calc. for $C_{25}H_{32}Cl_3NO$ $[M+H]^+$ 468.1622, found 468.1625.

2-(bicyclo[1.1.1]pentan-1-yl)-3-(3-(1,1,1,3,3,3-hexamethyl-2-(trimethylsilyl)trisilan-2-yl)bicyclo[1.1.1]pentan-1-yl)-*N*-phenylpropanamide (8a)

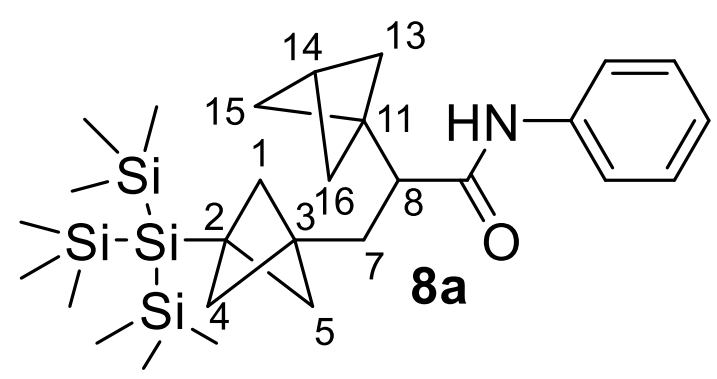

8a

Synthesized according to *General Procedure C* using acrylamide **2a** (14.7 mg, 0.1 mmol, 1.0 equiv.), TTMS (0.3 mmol, 3.0 equiv.) and **1** (0.2 mmol, 2.0 equiv.). Purification is through flash column chromatography (0-10% Et_2O/Hex) to give product **8a** (35.9 mg, 0.068 mmol, 68%) as a white solid.

R_f 0.6 (15% Et_2O/hexane); **^{1}H NMR** (600 MHz, $CDCl_3$) δ 7.51–7.44 (m, 2H, ArH), 7.36–7.29 (m, 2H, ArH), 7.11 (tt, *J* = 7.4, 1.2 Hz, 1H, ArH), 6.99 (s, 1H, exchangeable NH), 2.49 (s, 1H, H14), 2.28 (dd, *J* = 11.3, 3.0 Hz, 1H, H8), 1.86 (dd, *J* = 14.3, 11.3 Hz, 1H, H7), 1.77–1.67 (m, 12H, H1,H4,H5,H13,H15,H16), 1.41 (dd, *J* = 14.3, 3.0 Hz, 1H, H7'), 0.11 (s, 27H, TMS); ^{13}C NMR (151 MHz, $CDCl_3$) δ 171.2, 137.9, 129.2, 124.4, 120.3, 55.6, 49.5, 48.5, 47.9, 45.7, 32.7, 26.9, 25.9, 1.6; **HRMS** (ESI^+) calc. for $C_{28}H_{49}NOSi_4$ $[M+H]^+$ 528.2964, found 528.2967.

2-(bicyclo[1.1.1]pentan-1-yl)-3-(3-(1,1,1,3,3,3-hexamethyl-2-(trimethylsilyl)trisilan-2-yl)bicyclo[1.1.1]pentan-1-yl)-*N*-(4-(trifluoromethyl)phenyl)propenamide (8b)

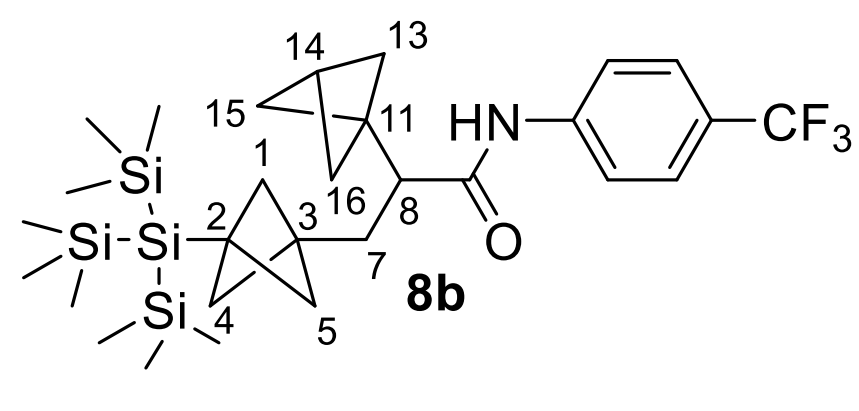

Synthesized according to *General Procedure C* using acrylamide **2b'** (21.5 mg, 0.1 mmol, 1.0 equiv.), TTMS (0.3 mmol, 3.0 equiv.) and **1** (0.2 mmol, 2.0 equiv.). Purification is through flash column chromatography (0-15% Et_2O/Hex) to give product **8b** (35.2 mg, 0.059 mmol, 59%) as a white solid.

$\mathbf{R_f}$ 0.5 (15% Et_2O/hexane); **^{1}H NMR** (600 MHz, $CDCl_3$) δ 7.65–7.55 (m, 4H, ArH), 7.14 (s, 1H, exchangeable NH), 2.50 (s, 1H, H14), 2.3–2.28 (m, 1H, H8), 1.90–1.84 (m, 1H, H7), 1.77–1.66 (m, 12H, H1, H1,H4,H5,H13,H15,H16), 1.43 (dd, *J* = 14.2, 2.3 Hz, 1H, H7'), 0.10 (s, 27H, TMS); **^{13}C NMR** (151 MHz, $CDCl_3$) δ 171.6, 141.0, 126.5 (q, *J* =3.8 Hz), 126.2 (q, *J* =32.7 Hz), 124.2 (q, *J* =271.5 Hz), 119.6, 55.5, 49.5, 48.6, 47.8, 45.5, 32.7, 27.0, 26.0, 1.6; **HRMS** (ESI$^+$) calc. for $C_{29}H_{47}FNOSi_4$ $[M+H]^+$594.2681, found 594.2694.

2-(bicyclo[1.1.1]pentan-1-yl)-3-(3-(2-(tert-butyl)-1,1,1,3,3,3-hexamethyltrisilan-2-yl)bicyclo[1.1.1]pentan-1-yl)-*N*-(4-fluorophenyl)propenamide (8c)

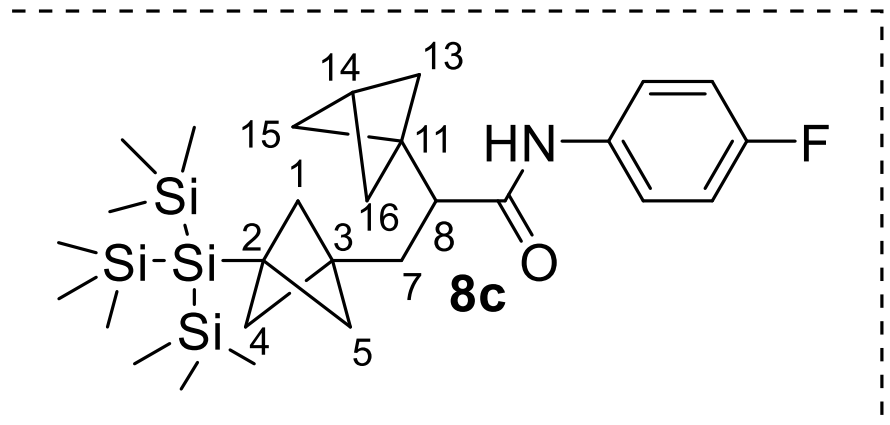

Synthesized according to *General Procedure C* using acrylamide **2b** (16.5 mg, 0.1 mmol, 1.0 equiv.), TTMS (0.3 mmol, 3.0 equiv.) and **1** (0.2 mmol, 2.0 equiv.). Purification is through flash column chromatography (0-10% Et_2O/Hex) to give product **8c** (28.6 mg, 0.054 mmol, 54%) as a white solid.

$\mathbf{R_f}$ 0.55 (15% Et_2O/hexane); **^{1}H NMR** (600 MHz, $CDCl_3$) δ 7.46–7.33 (m, 2H, ArH), 7.04–6.97 (m, 3H, ArH and exchangeable NH), 2.49 (s, 1H, H14), 2.28 (dd, *J* = 11.4, 3.0 Hz, 1H, H8), 1.85 (dd, *J* = 14.2, 11.4 Hz, 1H, H7), 1.77–1.66 (m, 12H, H1,H4,H5,H13,H15,H16), 1.41 (dd, *J* = 14.3, 3.0 Hz, 1H, H7'), 0.11 (s, 27H); **^{13}C NMR** (151 MHz, $CDCl_3$) δ 171.2, 159.6 (d, *J* = 243.5 Hz), 133.9 (d, *J* = 2.8 Hz), 122.2 (d, *J* = 7.8 Hz), 115.8 (d, *J* = 22.5 Hz), 55.6, 49.4, 48.4, 47.9, 45.7, 32.7, 26.9, 26.0, 1.6; **HRMS** (ESI$^+$) calc. for $C_{28}H_{48}FNOSi_4$ $[M+H]^+$546.2870, found 546.2875.

2-(bicyclo[1.1.1]pentan-1-yl)-3-(3-(1,1,1,3,3,3-hexamethyl-2-(trimethylsilyl)trisilan-2-yl)bicyclo[1.1.1]pentan-1-yl)-*N*-(p-tolyl)propenamide (8d)

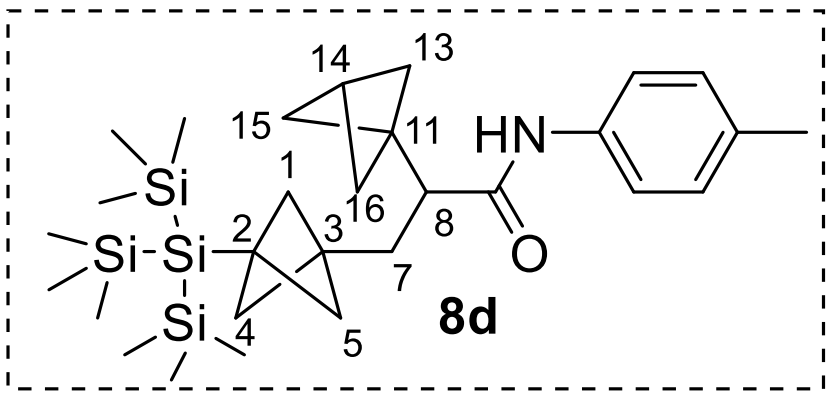

Synthesized according to *General Procedure C* using acrylamide **2c** (16.1 mg, 0.1 mmol, 1.0 equiv.), TTMS (0.3 mmol, 3.0 equiv.) and **1** (0.2 mmol, 2.0 equiv.). Purification is through flash column chromatography (0-10% Et_2O/Hex) to give product **8d** (32.5 mg, 0.06 mmol, 60%) as a white solid.
$\mathbf{R_f}$ 0.65 (18% Et_2O/hexane);**^{1}H NMR** (600 MHz, $CDCl_3$) δ 7.39–7.30 (m, 2H, ArH), 7.12 (d, *J* = 8.0 Hz, 2H, ArH), 6.96 (s, 1H, exchangeable NH), 2.48 (s, 1H, H14), 2.31 (s, 3H, CH_3), 2.27 (dd, *J* = 11.3, 3.0 Hz, 1H, H8), 1.86 (dd, *J* = 14.2, 11.3 Hz, 1H, H7), 1.78–1.66 (m, 12H H1,H4,H5,H13,H15,H16), 1.40 (dd, *J* = 14.2, 3.0 Hz, 1H, H7'), 0.11 (s, 27H, TMS); **^{13}C NMR** (151 MHz, $CDCl_3$) δ 171.0, 135.3, 134.0, 129.6, 120.5, 55.6, 49.4, 48.4, 47.9, 45.8, 32.6, 26.8, 25.9, 21.0, 1.6; **HRMS** (ESI^+) calc. for $C_{29}H_{51}NOSi_4$ $[M+H]^+$542.3120, found 542.3118.

2-(bicyclo[1.1.1]pentan-1-yl)-*N*-(3,5-dimethylphenyl)-3-(3-(1,1,1,3,3,3-hexamethyl-2-(trimethylsilyl)trisilan-2-yl)bicyclo[1.1.1]pentan-1-yl)propenamide (8e)

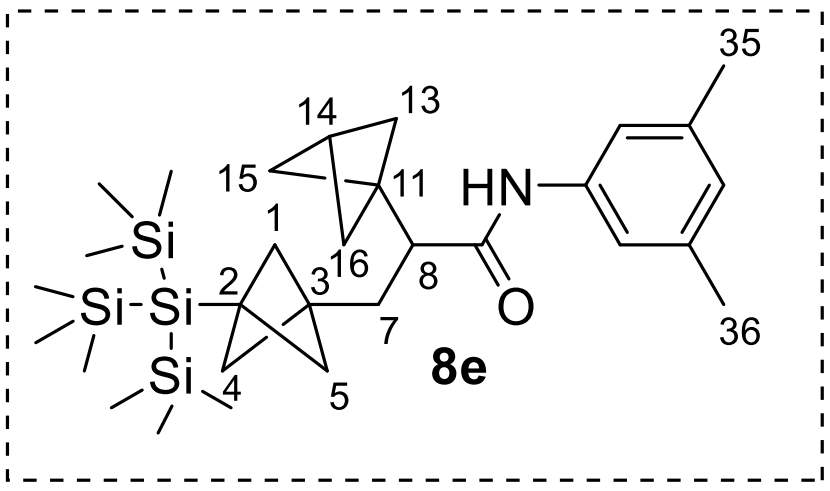

Synthesized according to *General Procedure C* using acrylamide **2d'** (17.5 mg, 0.1 mmol, 1.0 equiv.), TTMS (0.3 mmol, 3.0 equiv.) and **1** (0.2 mmol, 2.0 equiv.). Purification is through flash column chromatography (0-10% Et_2O/Hex) to give product **8e** (27.8 mg, 0.05 mmol, 50%) as a white solid.
$\mathbf{R_f}$ 0.62 (15% Et_2O/hexane);**^{1}H NMR** (600 MHz, $CDCl_3$) δ 7.10 (s, 2H, ArH), 6.96 (s, 1H, exchangeable NH), 6.75 (s, 1H, ArH), 2.48 (s, 1H, H14), 2.30-2.24 (m, 7H, CH_3 and H8), 1.85 (dd, *J* = 14.3, 11.3 Hz, 1H, H7), 1.78–1.66 (m, 12H, H1,H4,H5,H13,H15,H16), 1.40 (dd, *J* = 14.3, 2.9 Hz, 1H, H7'), 0.12 (s, 27H, TMS); **^{13}C NMR** (151 MHz, $CDCl_3$) δ 171.1, 138.8, 137.7, 126.2, 118.2, 55.6, 49.4, 48.4, 47.9, 45.8, 32.7, 26.9, 25.9, 21.5, 1.6; **HRMS** (ESI^+) calc. for $C_{30}H_{53}NOSi_4$ $[M+H]^+$556.3277, found 556.3278.

2-(bicyclo[1.1.1]pentan-1-yl)-*N*-(4-(tert-butyl)phenyl)-3-(3-(1,1,1,3,3,3-hexamethyl-2-(trimethylsilyl)trisilan-2-yl)bicyclo[1.1.1]pentan-1-yl)propenamide (8f)

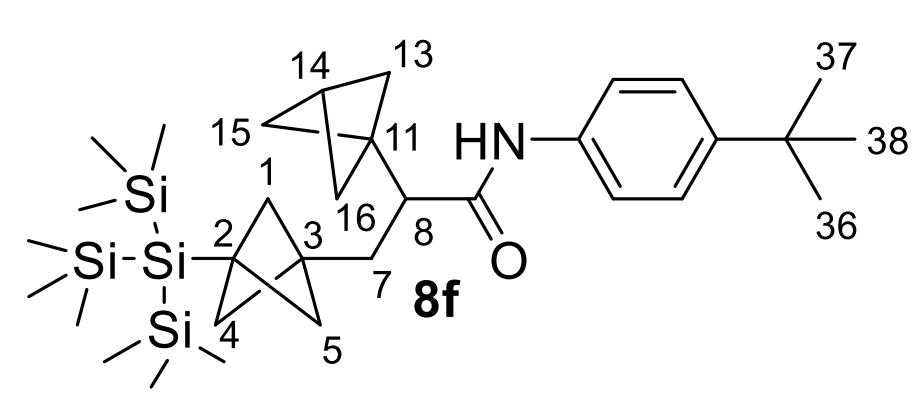

Synthesized according to *General Procedure C* using acrylamide **2d** (20.3 mg, 0.1 mmol, 1.0 equiv.), TTMS (0.3 mmol, 3.0 equiv.) and **1** (0.2 mmol, 2.0 equiv.). Purification is through flash column chromatography (0-10% Et_2O/Hex) to give product **8f** (29.8 mg, 0.051 mmol, 51%) as a white solid.

R_f 0.68 (18% Et_2O/hexane);**^{1}H NMR** (600 MHz, $CDCl_3$) δ 7.41–7.31 (m, 4H, ArH), 6.96 (s, 1H, exchangeable NH), 2.48 (s, 1H, H14), 2.27 (dd, *J* = 11.3, 3.0 Hz, 1H, H8), 1.86 (dd, *J* = 14.3, 11.3 Hz, 1H, H7), 1.79–1.67 (m, 12H, H1,H4,H5,H13,H15,H16), 1.40 (dd, *J* = 14.2, 3.0 Hz, 1H, H7'), 1.31 (s, 9H, CH_3), 0.11 (s, 27H, TMS); **^{13}C NMR** (151 MHz, $CDCl_3$) δ 171.1, 147.3, 135.3, 126.0, 120.1, 55.6, 49.5, 48.4, 47.9, 45.8, 34.5, 32.6, 31.5, 26.8, 25.9, 1.6; **HRMS** (ESI$^+$) calc. for $C_{32}H_{57}NOSi_4$ $[M+H]^+$584.3590, found 584.3592.

2-(bicyclo[1.1.1]pentan-1-yl)-3-(3-(1,1,1,3,3,3-hexamethyl-2-(trimethylsilyl)trisilan-2-yl)bicyclo[1.1.1]pentan-1-yl)-*N*-(4-methoxyphenyl)propenamide (8g)

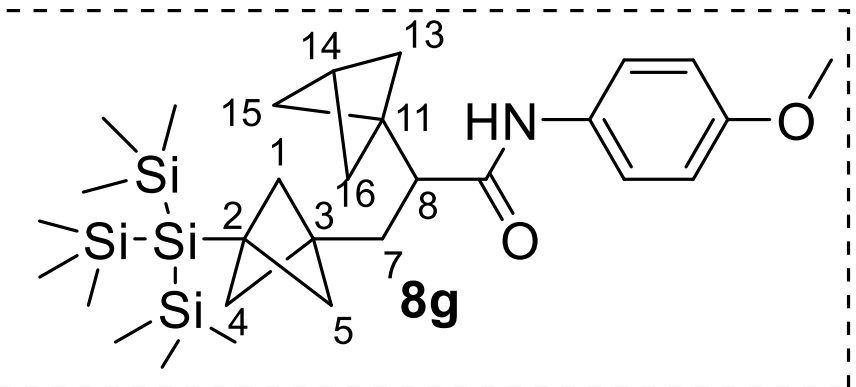

Synthesized according to *General Procedure C* using acrylamide **2e** (17.7 mg, 0.1 mmol, 1.0 equiv.), TTMS (0.3 mmol, 3.0 equiv.) and **1** (0.2 mmol, 2.0 equiv.). Purification is through flash column chromatography (0-15% Et_2O/Hex) to give product **8g** (35.2 mg, 0.063 mmol, 63%) as a white solid.

R_f 0.3 (15% Et_2O/hexane);**^{1}H NMR** (600 MHz, $CDCl_3$) δ 7.37–7.33 (m, 2H, ArH), 6.97 (s, 1H, exchangeable NH), 6.88–6.82 (m, 2H. ArH), 3.79 (s, 3H, OCH_3), 2.27 (dd, *J* = 11.3, 3.0 Hz, 1H, H8), 1.85 (dd, *J* = 14.2, 11.4 Hz, 1H, H7), 1.78–1.65 (m, 12H, H1,H4,H5,H13,H15,H16), 1.40 (dd, *J* = 14.2, 3.0 Hz, 1H, H7'), 0.12 (s, 27H, TMS); **^{13}C NMR** (151 MHz, $CDCl_3$) δ 171.0, 156.6, 131.0, 122.3, 114.3, 55.7, 55.6, 49.4, 48.2, 48.0, 45.8, 32.6, 26.8, 25.9, 1.6; **HRMS** (ESI$^+$) calc. for $C_{29}H_{51}NO_2Si_4$ $[M+H]^+$558.3070, found 558.3070.

2-(bicyclo[1.1.1]pentan-1-yl)-*N*-butyl-3-(3-(1,1,1,3,3,3-hexamethyl-2-(trimethylsilyl)trisilan-2-yl)bicyclo[1.1.1]pentan-1-yl)propenamide (8h)

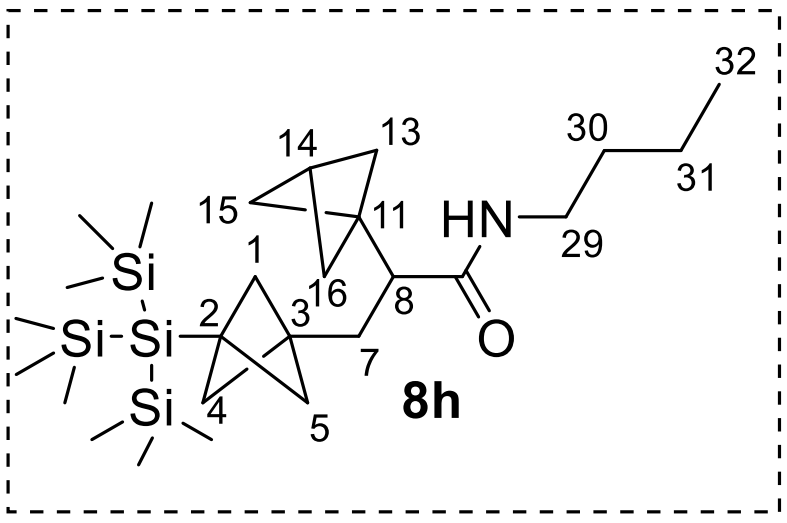

Synthesized according to *General Procedure C* using acrylamide **2k** (12.7 mg, 0.1 mmol, 1.0 equiv.), TTMS (0.3 mmol, 3.0 equiv.) and **1** (0.2 mmol, 2.0 equiv.). Purification is through flash column chromatography (0-25% Et_2O/Hex) to give product **8h** (26.4 mg, 0.052 mmol, 52%) as a white solid.

R*f* 0.25 (15% Et_2O/hexane);**^{1}H NMR** (600 MHz, $CDCl_3$) δ 5.29 (t, *J* = 5.8 Hz, 1H, exchangeable NH), 3.34–3.26 (m, 1H, H29), 3.23 – 3.14 (m, 1H, H29'), 2.44 (s, 1H, H14), 2.08 (dd, *J* = 11.5, 2.8 Hz, 1H, H8), 1.79–1.58 (m, 13H, H1,H4,H5,H13,H15,H16 and H7), 1.52–1.45 (m, 2H, H30), 1.39-1.32 (m, 2H, H31), 1.30 (dd, *J* = 14.2, 2.8 Hz, 1H, H7'), 0.92 (t, *J* = 7.3 Hz, 3H, H32), 0.13 (s, 27H, TMS); **^{13}C NMR** (151 MHz, $CDCl_3$) δ 172.5, 55.6, 49.4, 48.1, 47.5, 45.9, 39.2, 32.5, 32.2, 26.6, 25.8, 20.3, 13.9, 1.6; **HRMS** (ESI$^+$) calc. for $C_{26}H_{53}NOSi_4$ $[M+H]^+$508.3277, found 508.3270.

2-(bicyclo[1.1.1]pentan-1-yl)-*N*-cyclopropyl-3-(3-(1,1,1,3,3,3-hexamethyl-2-(trimethylsilyl)trisilan-2-yl)bicyclo[1.1.1]pentan-1-yl)propenamide (8i)

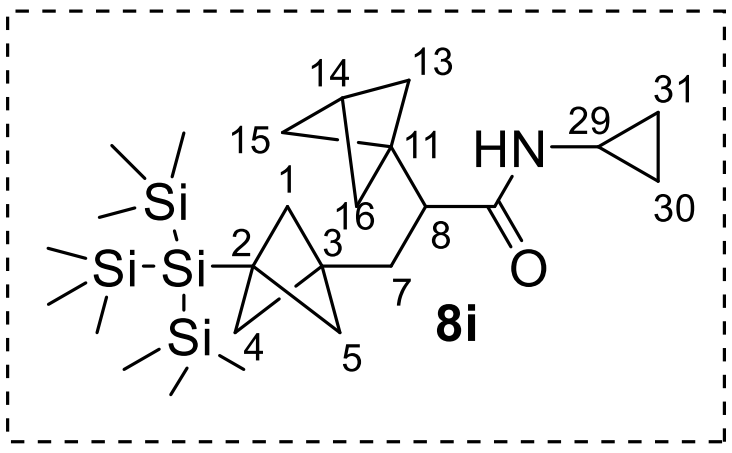

Synthesized according to *General Procedure C* using acrylamide **2l** (11.1 mg, 0.1 mmol, 1.0 equiv.), TTMS (0.3 mmol, 3.0 equiv.) and **1** (0.2 mmol, 2.0 equiv.). Purification is through flash column chromatography (0-30% Et_2O/Hex) to give product **8i** (22.1 mg, 0.045 mmol, 45%) as a white solid.

R*f* 0.2 (22% Et_2O/hexane);**^{1}H NMR** (600 MHz, $CDCl_3$) δ 5.52 (s, 1H, exchangeable NH), 2.67 (tp, *J* = 6.9, 3.3 Hz, 1H, H29), 2.43 (s, 1H, H14), 2.04 (dd, *J* = 11.5, 2.8 Hz, 1H, H8), 1.77–1.57 (m, 13H, H1,H4,H5,H13,H15,H16 and H7), 1.28 (dd, *J* = 14.2, 2.9 Hz, 1H, H7'), 0.80–0.71 (m, 2H, H31), 0.52 – 0.41 (m, 2H, H30), 0.13 (s, 27H, TMS); **^{13}C NMR** (151 MHz, $CDCl_3$) δ 174.0, 55.5, 49.3, 48.1, 47.2, 45.8, 32.4, 26.6, 25.8, 22.5, 6.9, 6.7, 1.6; **HRMS** (ESI$^+$) calc. for $C_{25}H_{49}NOSi_4$ $[M+H]^+$492.2964, found 492.2566.

2-(bicyclo[1.1.1]pentan-1-yl)-*N*-cyclohexyl-3-(3-(1,1,1,3,3,3-hexamethyl-2-(trimethylsilyl)trisilan-2-yl)bicyclo[1.1.1]pentan-1-yl)propenamide (8j)

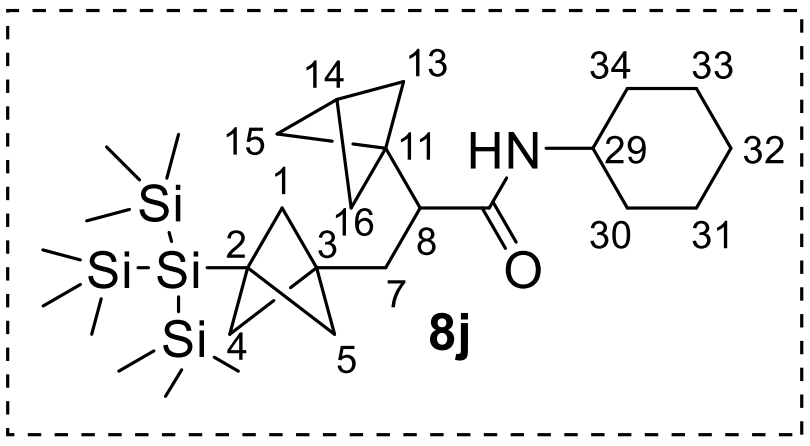

Synthesized according to *General Procedure C* using acrylamide **2m** (15.3 mg, 0.1 mmol, 1.0 equiv.), TTMS (0.3 mmol, 3.0 equiv.) and **1** (0.2 mmol, 2.0 equiv.). Purification is through flash column chromatography (0-20% Et_2O/Hex) to give product **8j** (22.4 mg, 0.042 mmol, 42%) as a white solid.

R*f* 0.25 (18% Et_2O/hexane);**^{1}H NMR** (600 MHz, $CDCl_3$) δ 5.16 (d, *J* = 8.4 Hz, 1H, exchangeable NH), 3.84–3.76 (m, 1H, H29), 2.43 (s, 1H, H14), 2.04 (dd, *J* = 11.5, 2.8 Hz, 1H, H8), 1.94–1.84 (m, 2H, cyclohexyl-CH_2), 1.78–1.58 (m, 17H, H1,H4,H5,H13,H15,H16, H7 and cyclohexyl-CH_2), 1.41–1.32 (m, 2H, cyclohexyl-CH_2), 1.28 (dd, *J* = 14.1, 2.8 Hz, 1H, H7'), 1.20-1.06 (m, 2H, cyclohexyl-CH_2), 0.13 (s, 27H, TMS); **^{13}C NMR** (151 MHz, $CDCl_3$) δ 171.4, 55.6, 49.3, 48.2, 47.8, 47.7, 45.9, 33.8, 33.6, 32.3, 26.6, 25.7, 25.7, 25.0, 25.0, 1.6; **HRMS** (ESI^+) calc. for $C_{28}H_{55}NOSi_4$ $[M+H]^+$534.3433, found 534.3434.

2-(bicyclo[1.1.1]pentan-1-yl)-3-(3-(1,1,1,3,3,3-hexamethyl-2-(trimethylsilyl)trisilan-2-yl)bicyclo[1.1.1]pentan-1-yl)-*N*-(2-methyl-4-oxopentan-2-yl)propenamide (8k)

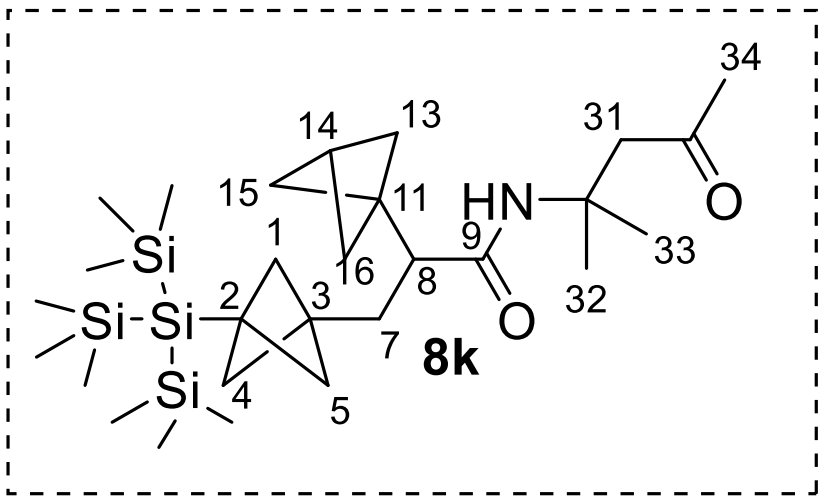

Synthesized according to *General Procedure C* using acrylamide **2n** (16.9 mg, 0.1 mmol, 1.0 equiv.), TTMS (0.3 mmol, 3.0 equiv.) and **1** (0.2 mmol, 2.0 equiv.). Purification is through flash column chromatography (0-25% Et_2O/Hex) to give product **8k** (35.2 mg, 0.064 mmol, 64%) as a white solid.

R*f* 0.2 (18% Et_2O/hexane);^{1}H NMR (600 MHz, $CDCl_3$) δ 5.76 (s, 1H, exchangeable NH), 2.98 (d, *J* = 16.7 Hz, 1H, H31), 2.78 (d, *J* = 16.7 Hz, 1H, H31'), 2.42 (s, 1H, H14), 2.13 (s, 3H, H34), 2.02 (dd, *J* = 11.5, 2.7 Hz, 1H, H8), 1.74–1.57 (m, 13H, H1,H4,H5,H13,H15,H16 and H7), 1.41 (s, 6H, H32 and H33), 1.24 (dd, *J* = 14.1, 2.7 Hz, 1H, H7'), 0.13 (s, 27H, TMS); **^{13}C NMR** (151 MHz, $CDCl_3$) δ 208.0, 172.2, 55.6, 52.4, 51.7, 49.4, 48.2, 47.8, 45.9, 32.3, 32.1, 27.4, 27.3, 26.6, 25.6, 1.6; **HRMS** (ESI^+) calc. for $C_{28}H_{55}NO_2Si_4$ $[M+H]^+$550.3383, found 550.3380.

2-(bicyclo[1.1.1]pentan-1-yl)-3-(3-(1,1,1,3,3,3-hexamethyl-2-(trimethylsilyl)trisilan-2-yl)bicyclo[1.1.1]pentan-1-yl)-1-morpholinopropan-1-one (8l)

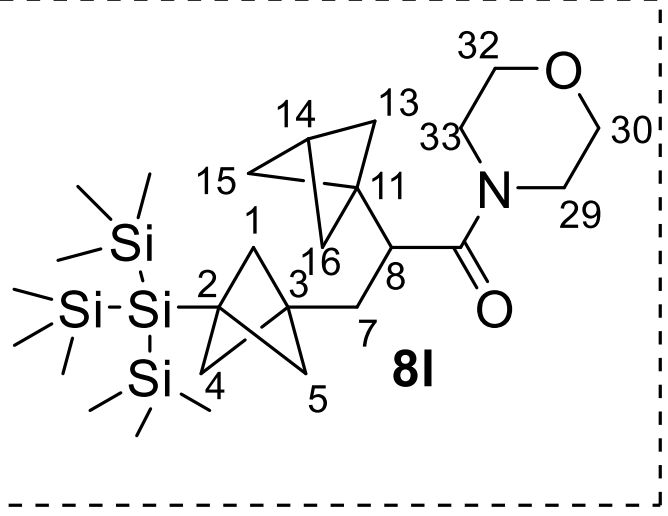

Synthesized according to *General Procedure C* using acrylamide **2p** (14.1 mg, 0.1 mmol, 1.0 equiv.), TTMS (0.3 mmol, 3.0 equiv.) and **1** (0.2 mmol, 2.0 equiv.). Purification is through flash column chromatography (0-30% Et_2O/Hex) to give product **8l** (29.8 mg, 0.057 mmol, 57%) as a white solid.

$\mathbf{R}_f$ 0.2 (22% Et_2O/hexane); ^{1}**H NMR** (600 MHz, $CDCl_3$) δ 3.72–3.48 (m, 8H, H29,H30,H32 and H33), 2.70 (dd, *J* = 11.3, 2.6 Hz, 1H, H8), 2.44 (s, 1H, H14), 1.95 (dd, *J* = 13.7, 11.3 Hz, 1H, H7), 1.70 – 1.59 (m, 12H, H1,H4,H5,H13,H15,H16), 1.28 (dd, *J* = 13.8, 2.6 Hz, 1H, H7'), 0.13 (s, 27H, TMS); ^{13}**C NMR** (151 MHz, $CDCl_3$) δ 171.5, 67.3, 66.9, 55.6, 49.6, 48.3, 46.4, 45.4, 42.2, 40.4, 32.8, 27.2, 25.7, 1.6; **HRMS** (ESI^+) calc. for $C_{26}H_{51}NO_2Si_4$ $[M+H]^+$522.3070, found 522.3068.

2-(bicyclo[1.1.1]pentan-1-yl)-3-(3-(1,1,1,3,3,3-hexamethyl-2-(trimethylsilyl)trisilan-2-yl)bicyclo[1.1.1]pentan-1-yl)-2-methyl-*N*-phenylpropanamide (8m)

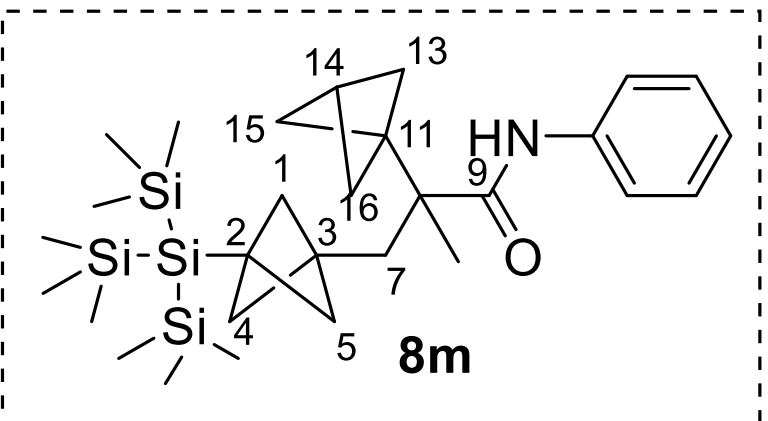

Synthesized according to *General Procedure C* using acrylamide **2q** (16.1 mg, 0.1 mmol, 1.0 equiv.), TTMS (0.3 mmol, 3.0 equiv.) and **1** (0.2 mmol, 2.0 equiv.). Purification is through flash column chromatography (0-10% Et_2O/Hex) to give product **8m** (35.8 mg, 0.066 mmol, 66%) as a white solid.

$\mathbf{R}_f$ 0.62 (15% Et_2O/hexane); ^{1}**H NMR** (600 MHz, $CDCl_3$) δ 7.46 (d, *J* = 7.9 Hz, 2H, ArH), 7.32 (t, *J* = 7.7 Hz, 2H, ArH), 7.12–7.07 (m, 2H, ArH and exchangeable NH), 2.49 (s, 1H, H14), 1.95 (d, *J* = 14.8 Hz, 1H, H7), 1.81 (s, 6H, BCP-CH_2), 1.73–1.66 (m, 6H, BCP-CH_2), 1.36 (d, *J* = 14.9 Hz, 1H, H7'), 1.22 (s, 3H, CH_3), 0.10 (s, 27H, TMS); ^{13}**C NMR** (151 MHz, $CDCl_3$) δ 172.3, 138.0, 129.1, 124.3, 120.3, 57.2, 50.9, 47.5, 47.0, 46.5, 39.6, 27.1, 25.2, 18.4, 1.6; **HRMS** (ESI^+) calc. for $C_{29}H_{51}NOSi_4$ $[M+H]^+$542.3120, found 542.3122.

2-(bicyclo[1.1.1]pentan-1-yl)-3-(3-(1,1,1,3,3,3-hexamethyl-2-(trimethylsilyl)trisilan-2-yl)bicyclo[1.1.1]pentan-1-yl)-*N*-(4-methoxyphenyl)-2-methylpropanamide (8n)

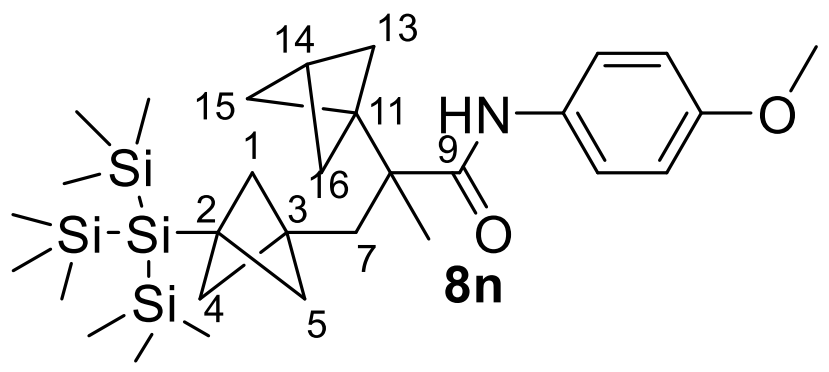

Synthesized according to *General Procedure C* using acrylamide **2r** (19.1 mg, 0.1 mmol, 1.0 equiv.), TTMS (0.3 mmol, 3.0 equiv.) and **1** (0.2 mmol, 2.0 equiv.). Purification is through flash column chromatography (0-15% Et_2O/Hex) to give product **8n** (27.5 mg, 0.048 mmol, 48%) as a white solid.

$\mathbf{R}_f$ 0.3 (14% Et_2O/hexane); **^{1}H NMR** (600 MHz, $CDCl_3$) δ 7.37–7.33 (m, 2H, ArH), 7.00 (s, 1H, exchangeable NH), 6.88–6.83 (m, 2H, ArH), 3.79 (s, 3H, OCH_3), 2.48 (s, 1H, H14), 1.94 (d, J = 14.8 Hz, 1H, H7), 1.81 (s, 6H, BCP-CH_2), 1.68 (qd, J = 9.5, 1.7 Hz, 6H, BCP-CH_2), 1.34 (d, J = 14.8 Hz, 1H, H7'), 1.21 (s, 3H, CH_3), 0.11 (s, 27H, TMS); **^{13}C NMR** (151 MHz, $CDCl_3$) δ 172.1, 156.5, 131.1, 122.3, 114.3, 57.3, 55.7, 51.0, 47.5, 47.1, 46.3, 39.5, 27.1, 25.1, 18.4, 1.6; **HRMS** (ESI$^+$) calc. for $C_{30}H_{53}NO_2Si_4$ $[M+H]^+$ 572.3226, found 572.3223.

methyl (2-(bicyclo[1.1.1]pentan-1-yl)-3-(3-(1,1,1,3,3,3-hexamethyl-2-(trimethylsilyl)trisilan-2-yl)bicyclo[1.1.1]pentan-1-yl)propanoyl)-*L*-phenylalanyl-*L*-leucinate (8o) (inseparable mixture of 1:1 diastereomers)

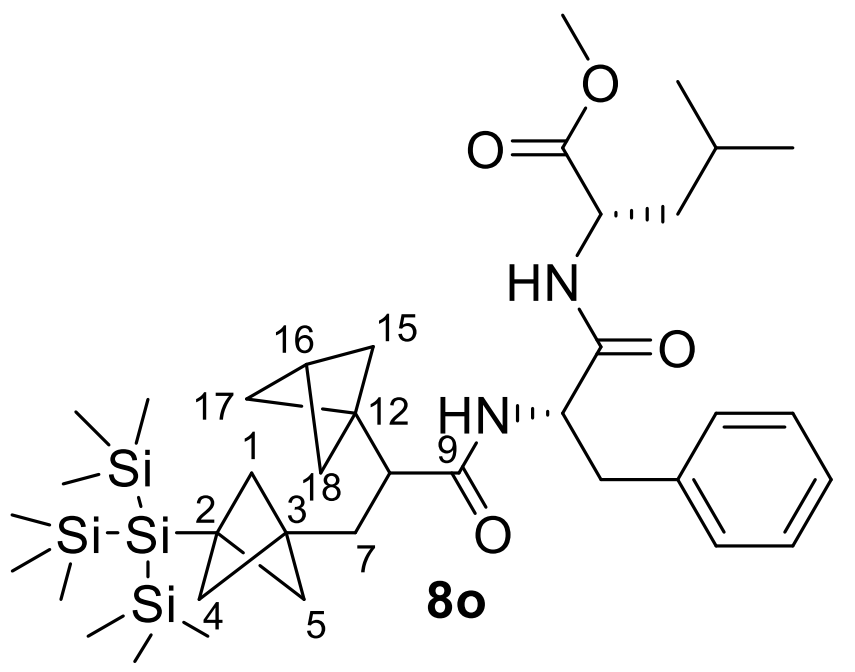

8o

Synthesized according to *General Procedure C* using acrylamide **2v** (17.3 mg, 0.05 mmol, 1.0 equiv.), TTMS (0.15 mmol, 3.0 equiv.) and **1** (0.1 mmol, 2.0 equiv.). Purification is through flash column chromatography (0-40% Et_2O/Hex) to give product **8o** (17.5 mg, 0.024mmol, 49%) as a white solid.

R_f 0.3 (40% Et_2O/Hex);**^{1}H NMR** (600 MHz, $CDCl_3$) δ 7.32–7.24 (m, 10H, ArH overlap with $CDCl_3$), 6.08 (t, *J* = 7.6 Hz, 2H, exchangeable NH), 5.86 (d, *J* = 7.5 Hz, 1H, exchangeable NH), 5.81 (d, *J* = 7.9 Hz, 1H, exchangeable NH), 4.68–4.56 (m, 2H, H11), 4.48-4.41 (m, 2H, H28), 3.69 (s, 3H, OCH_3), 3.65 (s, 3H, OCH_3), 3.15 (ddd, *J* = 13.6, 11.4, 5.5 Hz, 2H, H19), 2.93 (ddd, *J* = 22.8, 13.6, 8.9 Hz, 2H, H19'), 2.43 (s, 1H, H16), 2.41 (s, 1H, H16), 2.17 (ddd, *J* = 13.9, 10.8, 3.1 Hz, 2H, H8), 1.81–1.28 (m, 34H, H1,H4,H5,H15,H17,H18, H7,H29,H34 overlap with water peak), 0.89-0.85 (m, 12H, H35 and H36), 0.14 (s, 27H, TMS), 0.13 (s, 27H, TMS); **^{13}C NMR** (151 MHz, $CDCl_3$) δ 172.9, 172.7, 172.6, 172.5, 170.7, 170.7, 136.9, 136.7, 129.5, 129.5, 128.9, 128.9, 127.3, 127.2, 55.6, 55.4, 54.7, 54.5, 52.4, 52.4, 51.2, 51.1, 49.3, 49.3, 48.0, 47.8, 47.1, 47.1, 45.7, 45.6, 41.6, 41.5, 39.1, 38.9, 32.7, 32.3, 30.5, 30.5, 26.7, 26.7, 25.9, 25.9, 24.9, 24.8, 22.8, 22.7, 22.3, 22.0, 1.7, 1.7; **HRMS** (ESI^+) calc. for $C_{38}H_{66}N_2O_4Si_4$ $[M+H]^+$749.3992, found 749.3981.

(*Z*)-5-(bicyclo[1.1.1]pentan-1-yl)-2-((3-(dichloromethyl)bicyclo[1.1.1]pentan-1-yl)methyl)-*N*-phenylpent-2-enamide (3af)

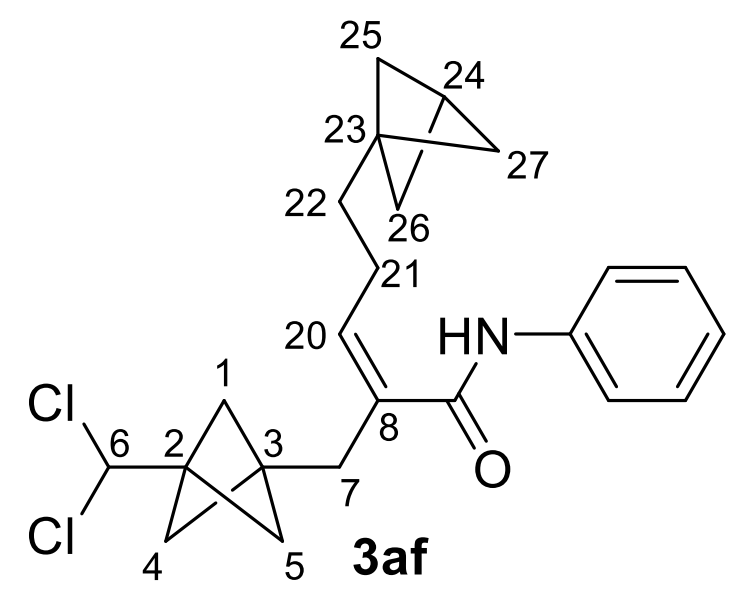

Synthesized according to *General Procedure A* using acrylamide **2ad** (9.4 mg, 0.05 mmol, 1.0 equiv.) and **1** (0.1 mmol, 2.0 equiv.). Purification is through flash column chromatography (0-20% Et_2O/Hex) to give product **3af** (2.0 mg, 0.005mmol, 10%) as a colorless oil.

R*f* 0.38 (20% Et_2O/hexane);**^{1}H NMR** (600 MHz, $CDCl_3$) δ 7.54 (d, *J* = 8.0 Hz, 2H, ArH), 7.39–7.31 (m, 3H, ArH and exchangeable NH), 7.12 (t, *J* = 7.4 Hz, 1H, ArH), 6.38 (t, *J* = 7.2 Hz, 1H, H20), 5.73 (s, 1H, H6), 2.71 (s, 2H, H7), 2.50 (s, 1H, H24), 2.13 (q, *J* = 7.5 Hz, 2H, H21), 1.74 (s, 6H, H1,H4,H5), 1.69 (s, 6H, H25,H26,H27), 1.58-1.53 (m, 2H, H22 overlap with water peak); **^{13}C NMR** (151 MHz, $CDCl_3$) δ 167.3, 138.1, 137.1, 134.2, 129.2, 124.5, 120.0, 72.0, 50.5, 49.3, 45.3, 44.7, 37.8, 32.1, 29.3, 27.7, 25.9; **HRMS** (ESI^+) calc. for $C_{23}H_{27}Cl_2NO$ $[M+H]^+$404.1542, found 404.1542.

(*Z*)-5-(bicyclo[1.1.1]pentan-1-yl)-*N*-phenyl-2-((3-(trichloromethyl)bicyclo[1.1.1]pentan-1-yl)methyl)pent-2-enamide (4aa)

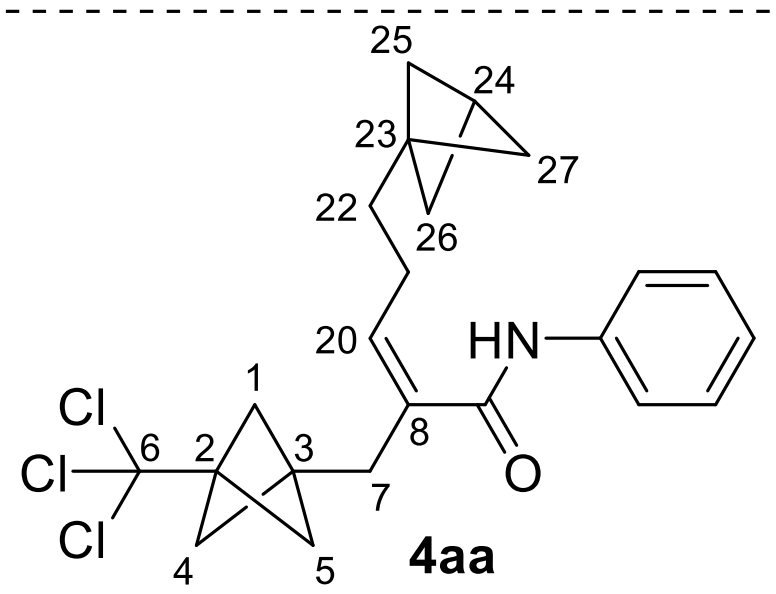

Synthesized according to *General Procedure B* using acrylamide **2ad** (9.4 mg, 0.05 mmol, 1.0 equiv.) and **1** (0.1 mmol, 2.0 equiv.). Purification is through flash column chromatography (0-20% Et_2O/Hex) to give product **4aa** (1.3 mg, 0.003mmol, 6%) as a colorless oil.

R*f* 0.41 (20% Et_2O/hexane);**^{1}H NMR** (600 MHz, $CDCl_3$) δ 7.56–7.52 (m, 2H, ArH), 7.39 (s, 1H, exchangeable NH), 7.34 (dd, *J* = 8.5, 7.3 Hz, 2H, ArH), 7.12 (t, *J* = 7.4 Hz, 1H, ArH), 6.38 (t, *J* = 7.2 Hz, 1H, H20), 2.76 (s, 2H, H7), 2.50 (s, 1H, H24), 2.14 (q, *J* = 7.5 Hz, 2H, H21), 1.88 (s, 6H, H1,H4,H5), 1.70 (s, 6H, H25,H26,H27), 1.59-1.53 (m, 2H, H22 overlap with water peak); **^{13}C NMR** (151 MHz, $CDCl_3$) δ 167.2, 138.1, 137.1, 134.2, 129.2, 124.5, 120.1, 98.0, 52.1, 50.5, 49.7, 45.3, 35.9, 32.1, 28.8, 27.7, 26.0. **HRMS** (ESI^+) calc. for $C_{23}H_{26}Cl_3NO$ $[M+H]^+$438.1153, found 438.1151.

2-(bicyclo[1.1.1]pentan-1-yl-3-d)-*N*-phenyl-3-(3-(trichloromethyl)bicyclo[1.1.1]pentan-1-yl)propenamide (4ab)

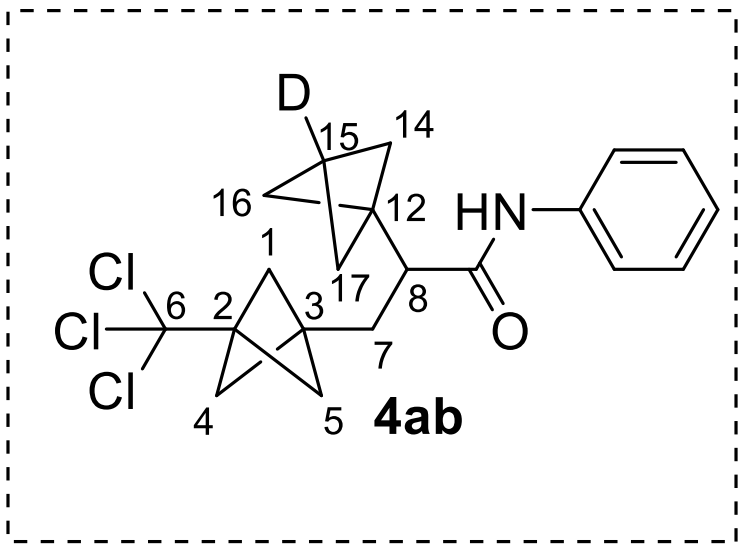

Synthesized according to *General Procedure B* using acrylamide **2a** (7.4 mg, 0.05 mmol, 1.0 equiv.), **1** (0.1 mmol, 2.0 equiv.) and $CDCl_3$(0.1 M, 0.5 mL) as solvent. Purification is through flash column chromatography (0-20% Et_2O/Hex) to give product **4ab** (7.4 mg, 0.019mmol, 37%) as a white solid.

R*f* 0.28 (20% Et_2O/hexane);**^{1}H NMR** (600 MHz, $CDCl_3$) δ 7.53–7.45 (m, 2H, ArH), 7.40–7.29 (m, 2H, ArH), 7.13 (tt, *J* = 7.3, 1.2 Hz, 1H, ArH), 6.98 (s, 1H, exchangeable NH), 2.34 (dd, *J* = 11.5, 2.8 Hz, 1H, H8), 2.15 (dd, *J* = 14.3, 11.6 Hz, 1H, H7), 1.92–1.80 (m, 6H, H1,H4,H5), 1.78–1.71 (m, 6H, H14,H16,H17), 1.66 (dd, *J* = 14.4, 2.8 Hz, 1H, H7'); **^{13}C NMR** (151 MHz, $CDCl_3$) δ 170.4, 137.8, 129.3, 124.6, 120.1, 98.1, 51.8, 49.5, 49.3, 48.5, 45.5, 35.7, 29.9, 29.6; **HRMS** (ESI^+) calc. for $C_{20}H_{21}DCl_3NO$ $[M+H]^+$399.0903, found 399.0903.

***N*-(2-(bicyclo[1.1.1]pentan-1-yl)-3-(3-(dichloromethyl)bicyclo[1.1.1]pentan-1-yl)propyl)aniline (3ad)**

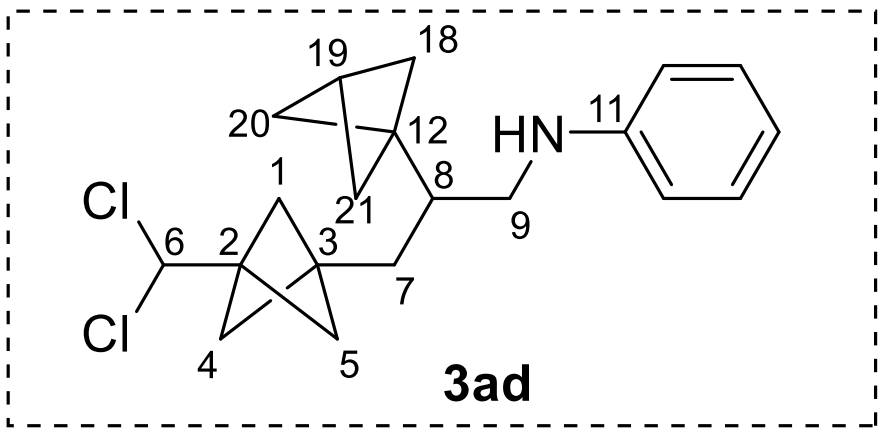

To a solution of **3a** (20 mg, 0.055 mmol, 1.0 equiv.) in anhydrous THF (0.6 mL) at 0 °C was added $LiAlH_4$ (0.08 mL, 0.16 mmol, 2.0 M in THF, 3.0 equiv.) slowly. The reaction mixture was warmed to rt and then refluxed overnight. The reaction mixture was cooled to 0 °C and sat. aq. Rochelle salt (5.0 mL) was added slowly. The layers were separated and the aqueous layer was extracted with EtOAc (3 x 5 mL). The combined organic layers were washed with brine (10 mL), dried over Na_2SO_4, filtered and concentrated under reduced pressure. The crude residue was purified by flash column chromatography (0-10% Et_2O/Hex) to give amine **3ad** (8 mg, 0.023, 42%) as colorless oil.

R*f* 0.60 (20% Et_2O/hexane);**^{1}H NMR** (600 MHz, $CDCl_3$) δ 7.21 – 7.13 (m, 2H, ArH), 6.69 (t, *J* = 7.3 Hz, 1H, ArH), 6.57 (d, *J* = 8.0 Hz, 2H), 5.74 (s, 1H, H6), 3.55 (s, 1H, exchangeable NH), 3.01 (m, 3.05-2.96, 2H, H9), 2.50 (s, 1H, H19), 1.75 – 1.67 (m, 13H, H1, H4, H5, H18, H20, H21 and H8), 1.63 (dd, *J* = 14.6, 4.4 Hz, 1H, H7), 1.46 (dd, *J* = 14.7, 7.5 Hz, 1H, H7'); **^{13}C NMR** (151 MHz, CDCl3) δ 148.5, 129.4, 117.3, 112.8, 72.1, 49.4, 49.3, 48.0, 46.2, 44.6, 38.3, 37.5, 31.0, 27.5, 0.1; **HRMS** (ESI^+) calc. for $C_{20}H_{25}Cl_2N$ $[M+H]^+$350.1437, found 350.1451.

3-(3-methylbicyclo[1.1.1]pentan-1-yl)-2-(3-methylenecyclobutyl)-*N*-phenylpropanamide (3ae)

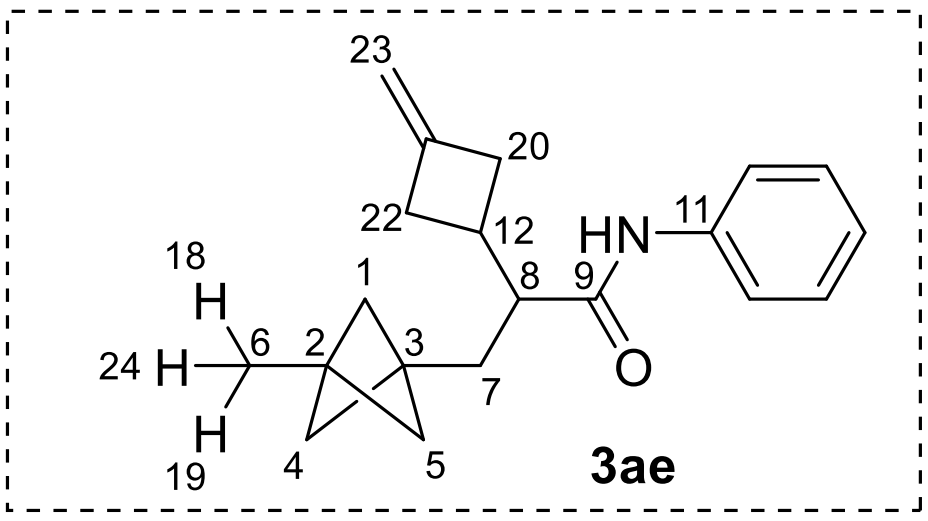

To a solution of **3a** (20 mg, 0.055 mmol, 1.0 equiv.) in anhydrous toluene (1.0 mL) was added AIBN (1.4 mg, 0.008 mmol, 0.15 equiv.) and Bu_3SnH (44 μL, 0.16 mmol, 3.0 equiv.). After stirring at 80 °C for 3 h, the reaction mixture was concentrated under reduced pressure. The residue was filtered through celite and washed with EtOAc, and concentrated under reduced pressure. The crude residue was purified by flash column chromatography (0-20% Et_2O/Hex) to give **3ae** (6.5 mg, 0.022, 40%) as colorless oil.
Rf 0.49 (20% Et_2O/hexane);^{1}**H NMR** (600 MHz, $CDCl_3$) δ 7.49 (d, *J* = 7.6 Hz, 2H, ArH), 7.35 – 7.29 (m, 2H, ArH), 7.11 (t, *J* = 7.5 Hz, 1H, ArH), 7.02 (s, 1H, exchangeable NH), 4.78 (h, *J* = 2.3 Hz, 2H), 2.56 – 2.49 (m, 2H, H20 and H22), 2.46-2.41 (m, 1H, H12), 2.35 – 2.26 (m, 3H, H20', H22' and H8), 2.10 (dd, *J* = 14.0, 10.6 Hz, 1H, H7), 1.79 – 1.70 (m, 6H, H1, H4 and H5), 1.55 (dd, *J* = 14.1, 2.0 Hz, 1H, H7'), 1.14 (s, 3H, H18, H19 and H24); ^{13}**C NMR** (151 MHz, CDCl3) δ 171.6, 145.3, 138.0, 129.2, 124.4, 120.1, 107.2, 49.3, 47.8, 46.2, 43.8, 43.7, 39.8, 33.8, 27.1, 26.0; **HRMS** (ESI$^+$) calc. for $C_{20}H_{25}NO$ $[M+H]^+$296.2009, found 296.2012.

(*Z*)-5-(bicyclo[1.1.1]pentan-1-yl)-2-((3-(1,1,1,3,3,3-hexamethyl-2-(trimethylsilyl)trisilan-2-yl)bicyclo[1.1.1]pentan-1-yl)methyl)-*N*-phenylpent-2-enamide (8p)

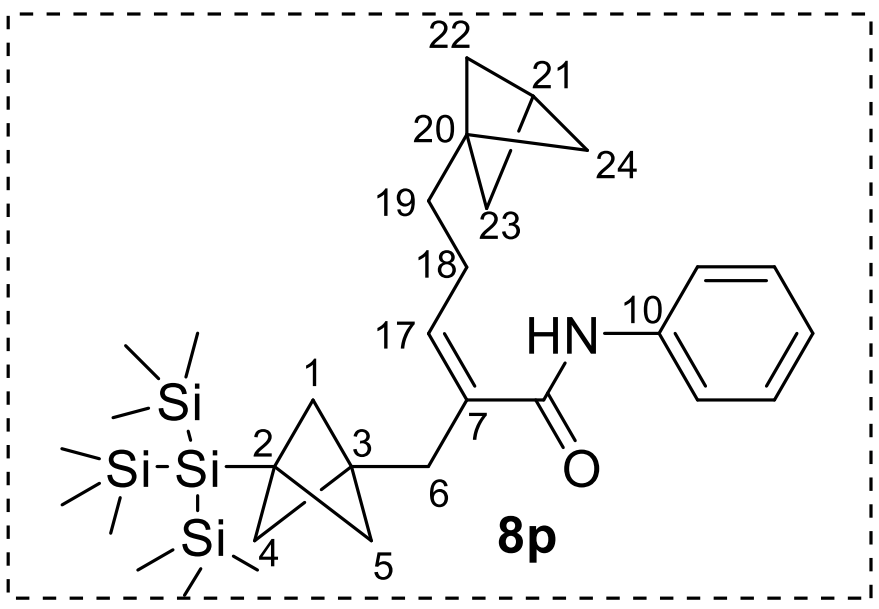

Synthesized according to *General Procedure C* using acrylamide **2ad** (9.4 mg, 0.05 mmol, 1.0 equiv.), TTMS (0.15 mmol, 3.0 equiv.) and **1** (0.1 mmol, 2.0 equiv.). Purification is through flash column chromatography (0-10% Et_2O/Hex) to give product **8p** (4.0 mg, 0.007 mmol, 14%) as a white solid.
$\mathbf{R_f}$ 0.42 (15% Et_2O/Hex);^{1}**H NMR** (600 MHz, $CDCl_3$) δ7.54 – 7.51 (m, 2H, ArH), 7.36 – 7.32 (m, 3H, ArH and exchangeable NH), 7.13 – 7.10 (m, 1H, ArH), 6.40 (t, *J* = 7.2 Hz, 1H, H17), 2.51 (s, 2H, H6), 2.49 (s, 1H, H21), 2.14 – 2.10 (m, 2H, H18), 1.77 (s, 6H, H1, H4 and H5), 1.68 (s, 6H, H22, H23 and H24), 1.57-1.54 (m, 2H, overlap with water peak), 0.14 (s, 27H, TMS); ^{13}**C NMR** (151 MHz, $CDCl_3$) δ 167.5, 138.3, 137.3, 133.9, 129.2, 124.3, 120.1, 55.8, 55.5, 50.5, 47.4, 45.4, 32.1, 27.7, 26.4, 26.0, 1.6; **HRMS** (ESI$^+$) calc. for $C_{31}H_{53}NOSi_4$ $[M+H]^+$568.3277, found 568.3274.

(*Z*)-2-((3-(1,1,1,3,3,3-hexamethyl-2-(trimethylsilyl)trisilan-2-yl)bicyclo[1.1.1]pentan-1-yl)methyl)-*N*-phenylpent-2-enamide (8q)

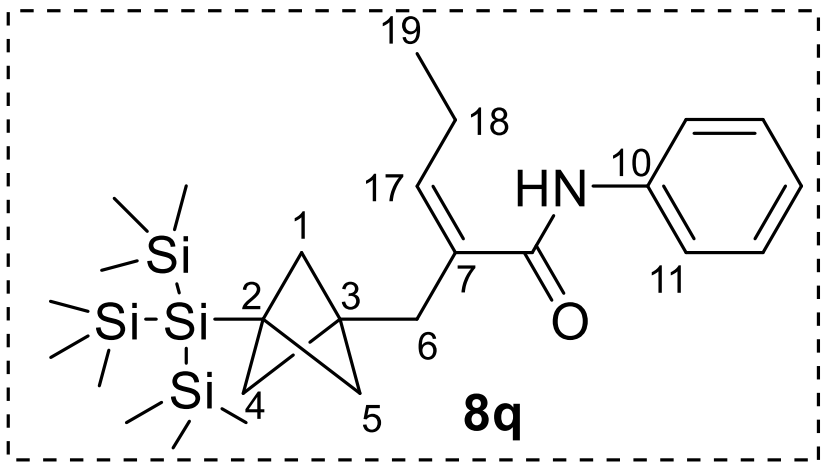

Synthesized according to *General Procedure C* using acrylamide **2ad** (9.4 mg, 0.05 mmol, 1.0 equiv.), TTMS (0.15 mmol, 3.0 equiv.) and **1** (0.1 mmol, 2.0 equiv.). Purification is through flash column chromatography (0-15% Et_2O/Hex) to give product **8q** (9.5 mg, 0.019 mmol, 38%) as a white solid.

R*f* 0.32 (15% Et_2O/Hex);**^{1}H NMR** (600 MHz, $CDCl_3$) δ 7.53 (d, *J* = 7.3 Hz, 2H, ArH), 7.39 (s, 1H, exchangeable NH), 7.34 (td, *J* = 7.4, 1.5 Hz, 2H, ArH), 7.11 (tt, *J* = 7.3, 1.2 Hz, 1H, ArH), 6.41 (t, *J* = 7.3 Hz, 1H, H17), 2.51 (s, 2H, H6), 2.18 (p, *J* = 7.5 Hz, 2H, H18), 1.77 (s, 6H, H1, H4 and H5), 1.07 (t, *J* = 7.5 Hz, 3H, H19), 0.14 (s, 27H, TMS); **^{13}C NMR** (151 MHz, $CDCl_3$) δ 167.6, 138.7, 138.3, 133.6, 129.2, 124.3, 120.2, 55.7, 47.4, 32.0, 26.4, 22.0, 13.7, 1.6; **HRMS** (ESI^+) calc. for $C_{26}H_{47}NOSi_4$ $[M+H]^+$502.2807, found 502.2808.

2-(bicyclo[1.1.1]pentan-1-yl-3-d)-3-(3-(1,1,1,3,3,3-hexamethyl-2-(trimethylsilyl)trisilan-2-yl)bicyclo[1.1.1]pentan-1-yl)-*N*-phenylpropanamide (8r)

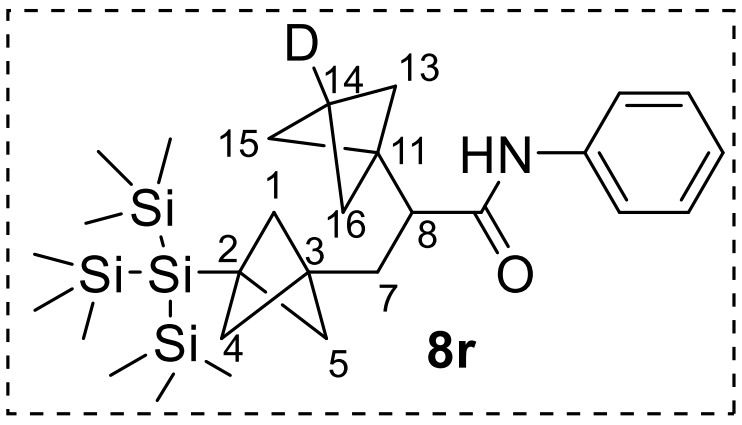

Synthesized according to *General Procedure C* using acrylamide **2a** (14.7 mg, 0.1 mmol, 1.0 equiv.), $(CH_3Si)_3Si$-D (0.3 mmol, 3.0 equiv.) and **1** (0.2 mmol, 2.0 equiv.). Purification is through flash column chromatography (0-10% Et_2O/Hex) to give product **8r** (6.9 mg, 0.013 mmol, 13%) as a white solid. The reaction occurred with 65% deuterium incorporation (as calculated by integration of the singlet at 2.49 ppm), confirming the role of tris(trimethylsilyl)silane as hydrogen atom donor.

R*f* 0.6 (15% Et_2O/hexane); **^{1}H NMR** (600 MHz, $CDCl_3$) δ 7.47 (d, *J* = 7.9 Hz, 2H, ArH), 7.32 (t, *J* = 7.7 Hz, 2H, ArH), 7.11 (t, *J* = 7.4 Hz, 1H, ArH), 6.97 (s, 1H, exchangeable NH), 2.28 (dd, *J* = 11.3, 3.0 Hz, 1H, H8), 1.86 (dd, *J* = 14.3, 11.3 Hz, 1H, H7), 1.78 – 1.67 (m, 12H, H1, H4, H5, H13, H15 and H16), 1.41 (dd, *J* = 14.3, 3.0 Hz, 1H, H7'), 0.11 (s, 27H, TMS); ^{13}C NMR (151 MHz, $CDCl_3$) δ 171.1, 137.9, 129.2, 124.4, 120.3, 55.6, 49.3, 48.5, 47.9, 45.7, 32.7, 26.9, 25.9, 1.6; **HRMS** (ESI^+) calc. for $C_{28}H_{48}DNOSi_4$ $[M+H]^+$529.3027, found 529.3022.

Incorporation of TTMS into [3.1.1]propellane was unsuccessful and direct addition of TTMS to acrylamide (the product shown below) was observed

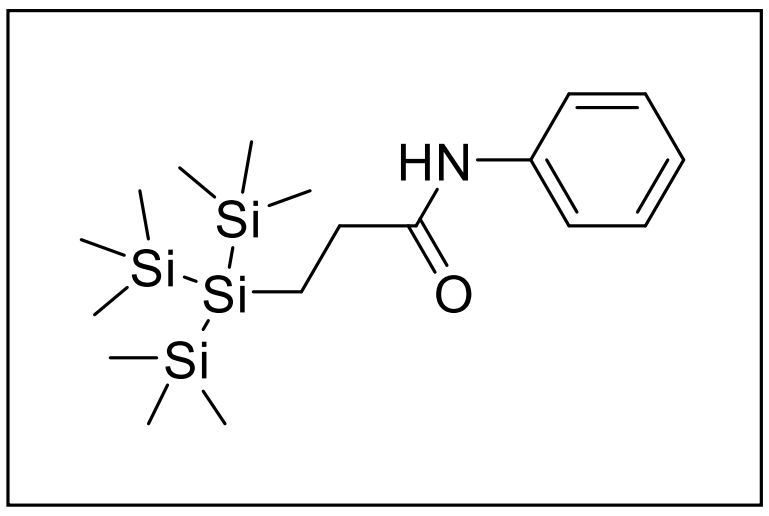

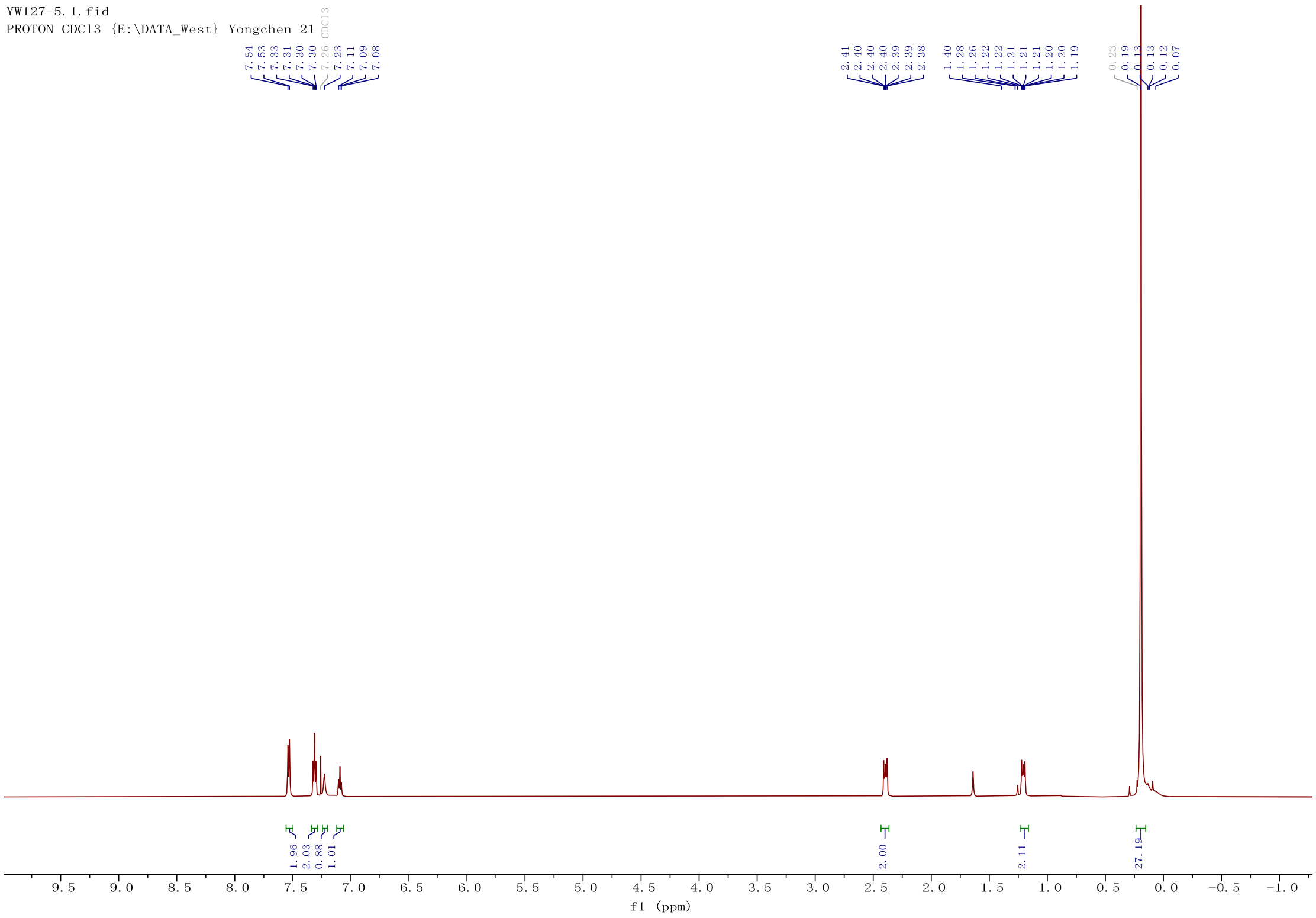

4. Mechanistic Study

Radical scavenger experiments

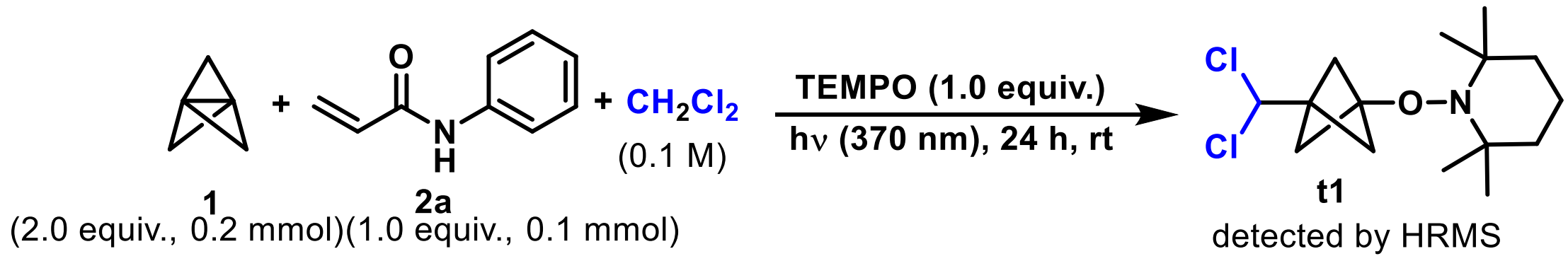

Target Compound Screening Report

Results Acquired by The University of Texas at Austin Mass Spectrometry Facility

**Data File**	MSF25-0056(YW261-1)_hrESIposLC1.d	**Sample Name**	0056(YW261-1)	**Comment**	0056(YW261-1)
**Position**	P1-A6	**Instrument Name**	6530	**User Name**	
**Acq Method**	LC_C18_pos_jl.m	**Acquired Time**	12/5/2025 11:38:42 AM	**DA Method**	MSF.m

MS Zoomed Spectrum

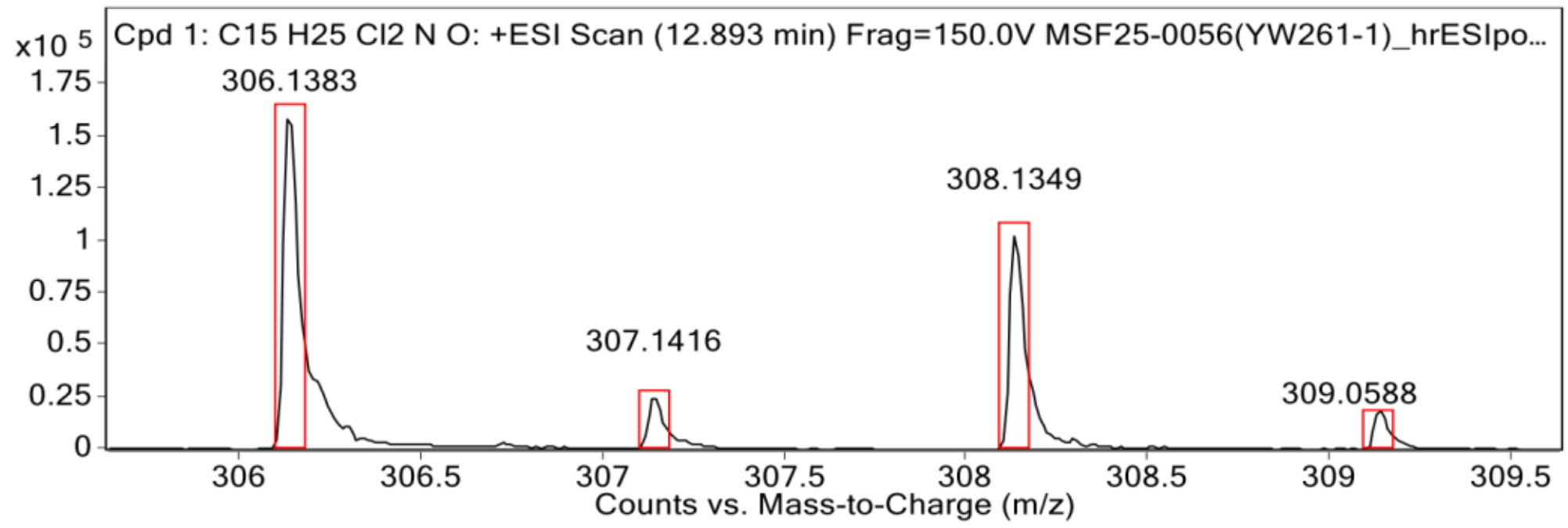

MS Spectrum Peak List

Obs. m/z	Calc. m/z	Charge	Abundance	Formula	Ion Species	Tgt Mass Error (ppm)
306.1383	306.1386	1	164782	C15H25Cl2NO	(M+H)+	0.91
307.1416	307.1419	1	25836	C15H25Cl2NO	(M+H)+	0.88
308.1349	308.1359	1	103536	C15H25Cl2NO	(M+H)+	3.07
309.0588	309.1390	1	606	C15H25Cl2NO	(M+H)+	259.41
368.3153			493038			

--- End Of Report ---

HRMS (ESI$^+$) calc. for $C_{15}H_{25}Cl_2NO$ $[M+H]^+$306.1386, found 306.1383.

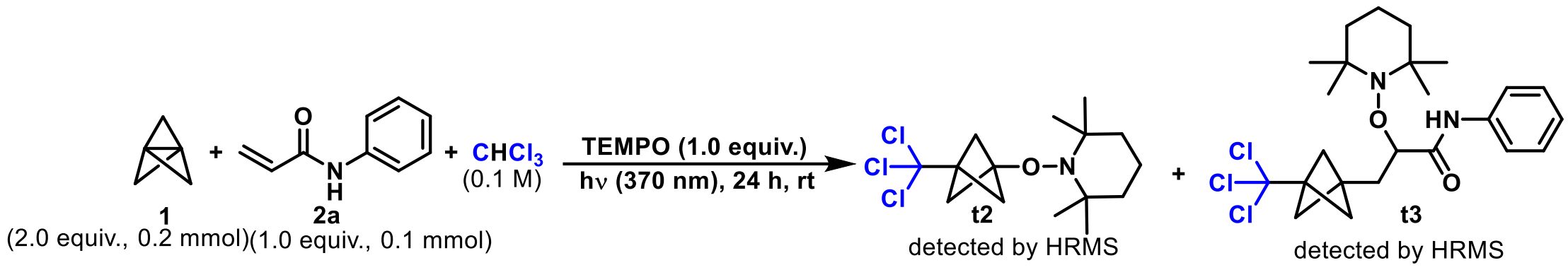

Target Compound Screening Report

Results Acquired by The University of Texas at Austin Mass Spectrometry Facility

**Data File**	MSF25-0056(YW261-2)_hrESIposLC1.d	**Sample Name**	0056(YW261-2)	**Comment**	0056(YW261-2)
**Position**	P1-A7	**Instrument Name**	6530	**User Name**	
**Acq Method**	LC_C18_pos_jl.m	**Acquired Time**	12/5/2025 12:01:43 PM	**DA Method**	MSF.m

MS Zoomed Spectrum

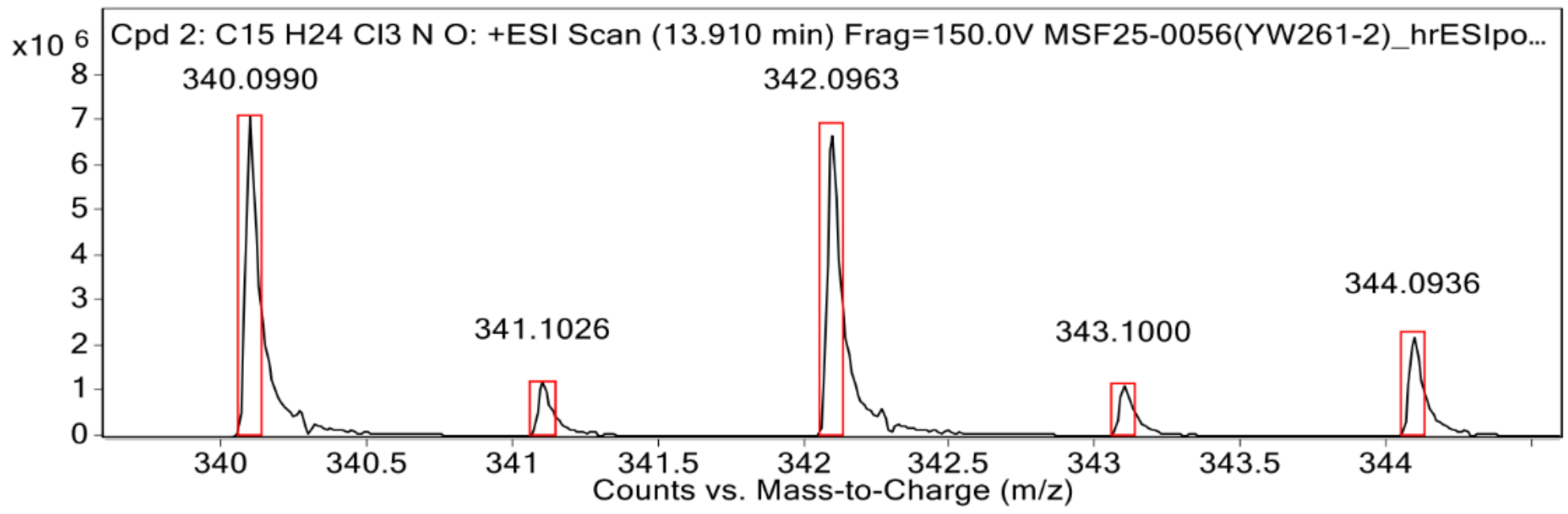

MS Spectrum Peak List

Obs. m/z	Calc. m/z	Charge	Abundance	Formula	Ion Species	Tgt Mass Error (ppm)
340.0990	340.0996	1	7111736	C15H24Cl3NO	(M+H)+	1.7
341.1026	341.1029	1	1212738	C15H24Cl3NO	(M+H)+	0.78
342.0963	342.0968	1	6753024	C15H24Cl3NO	(M+H)+	1.58
343.1000	343.1000	1	1154120	C15H24Cl3NO	(M+H)+	0.06
344.0936	344.0942	1	2221385	C15H24Cl3NO	(M+H)+	1.53

--- End Of Report ---

HRMS (ESI$^+$) calc. for **t2**, $C_{15}H_{24}Cl_3NO$ $[M+H]^+$340.0996, found 340.0990.

Target Compound Screening Report

Results Acquired by The University of Texas at Austin Mass Spectrometry Facility

**Data File**	MSF25-0056(YW261-2)_hrESIposLC1.d	**Sample Name**	0056(YW261-2)	**Comment**	0056(YW261-2)
**Position**	P1-A7	**Instrument Name**	6530	**User Name**	
**Acq Method**	LC_C18_pos_jl.m	**Acquired Time**	12/5/2025 12:01:43 PM	**DA Method**	MSF.m

MS Zoomed Spectrum

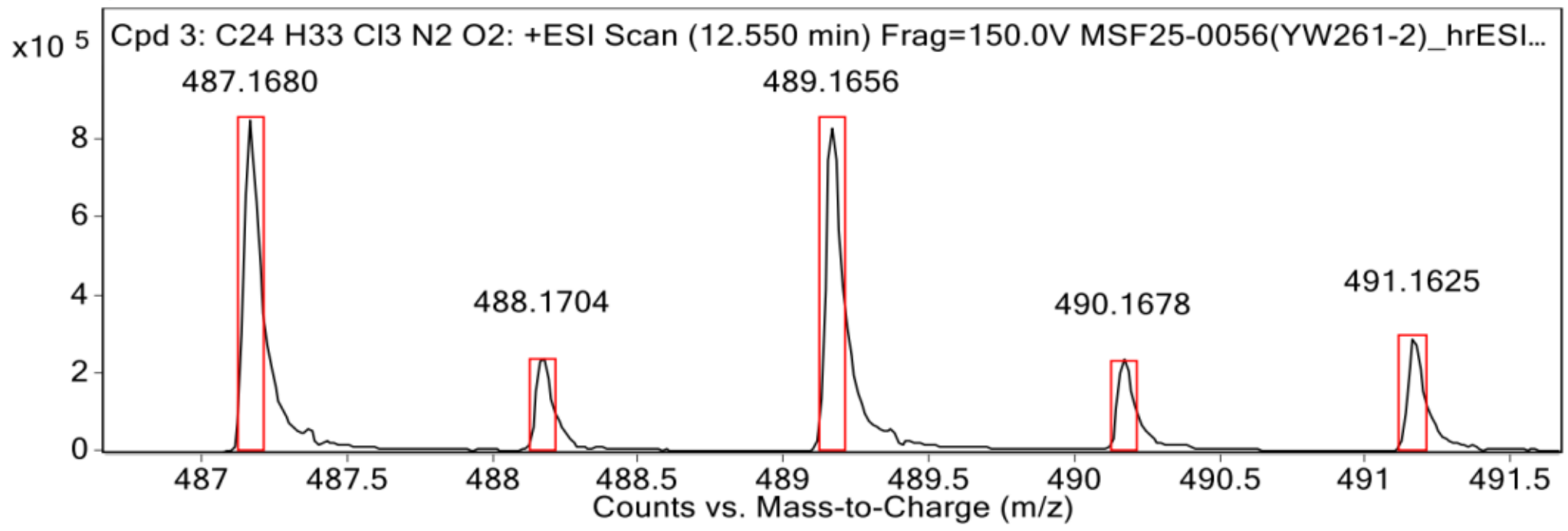

MS Spectrum Peak List

Obs. m/z	Calc. m/z	Charge	Abundance	Formula	Ion Species	Tgt Mass Error (ppm)
487.1680	487.1680	1	859296	C24H33Cl3N2O2	(M+H)+	-0.02
488.1704	488.1713	1	244633	C24H33Cl3N2O2	(M+H)+	1.73
489.1656	489.1655	1	830578	C24H33Cl3N2O2	(M+H)+	-0.36
490.1678	490.1685	1	238616	C24H33Cl3N2O2	(M+H)+	1.38
491.1625	491.1632	1	297823	C24H33Cl3N2O2	(M+H)+	1.35

--- End Of Report ---

HRMS (ESI$^+$) calc. for **t3**, $C_{24}H_{33}Cl_3N_2O_2$ $[M+H]^+$487.1680, found 487.1680.

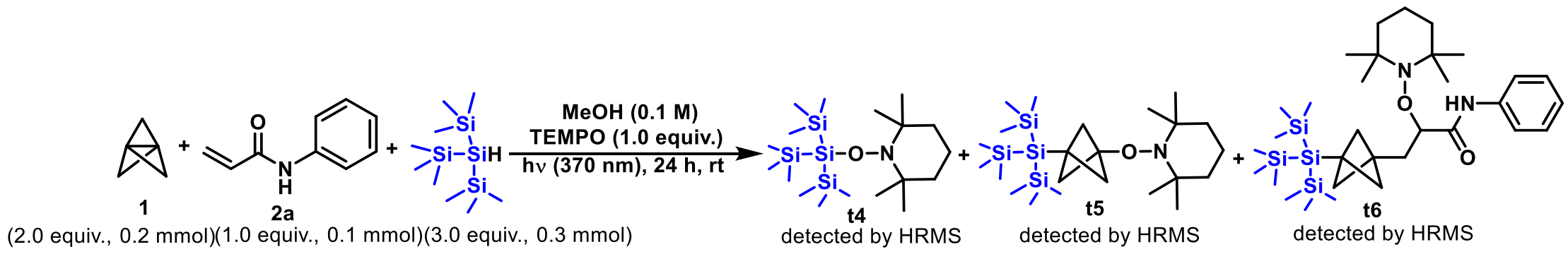

Target Compound Screening Report

Results Acquired by The University of Texas at Austin Mass Spectrometry Facility

**Data File**	MSF25-0056(YW261-3)_hrESIposLC2.d	**Sample Name**	0056(YW261-3)	**Comment**	0056(YW261-3)
**Position**	P1-A8	**Instrument Name**	6530	**User Name**	
**Acq Method**	LC_C18_pos_jl.m	**Acquired Time**	12/5/2025 3:06:27 PM	**DA Method**	MSF.m

MS Zoomed Spectrum

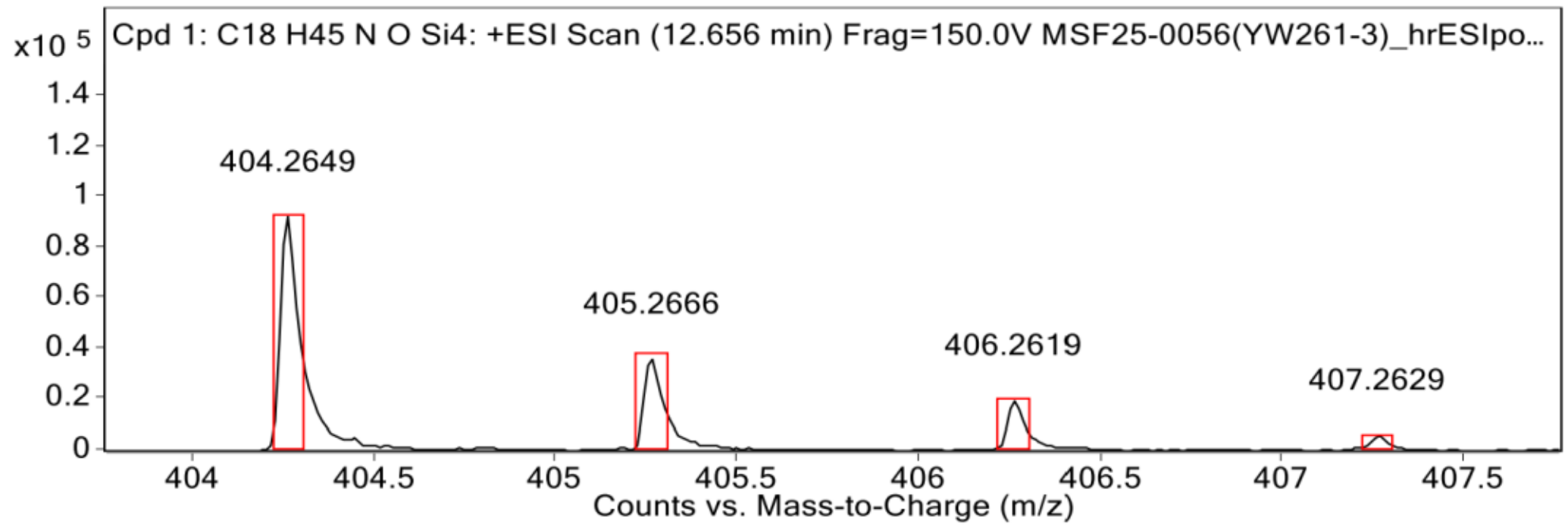

MS Spectrum Peak List

Obs. m/z	Calc. m/z	Charge	Abundance	Formula	Ion Species	Tgt Mass Error (ppm)
404.2649	404.2651	1	92698	C18H45NOSi4	(M+H)+	0.58
405.2666	405.2665	1	36313	C18H45NOSi4	(M+H)+	-0.1
406.2619	406.2643	1	19856	C18H45NOSi4	(M+H)+	5.82
407.2629	407.2647	1	6058	C18H45NOSi4	(M+H)+	4.64
470.3107			1663366			

--- End Of Report ---

HRMS (ESI^+) calc. for **t4**, $C_{18}H_{45}NOSi_4$ $[M+H]^+$404.2651, found 404.2649.

Target Compound Screening Report

Results Acquired by The University of Texas at Austin Mass Spectrometry Facility

**Data File**	MSF25-0056(YW261-3)_hrESIposLC2.d	**Sample Name**	0056(YW261-3)	**Comment**	0056(YW261-3)
**Position**	P1-A8	**Instrument Name**	6530	**User Name**	
**Acq Method**	LC_C18_pos_jl.m	**Acquired Time**	12/5/2025 3:06:27 PM	**DA Method**	MSF.m

MS Zoomed Spectrum

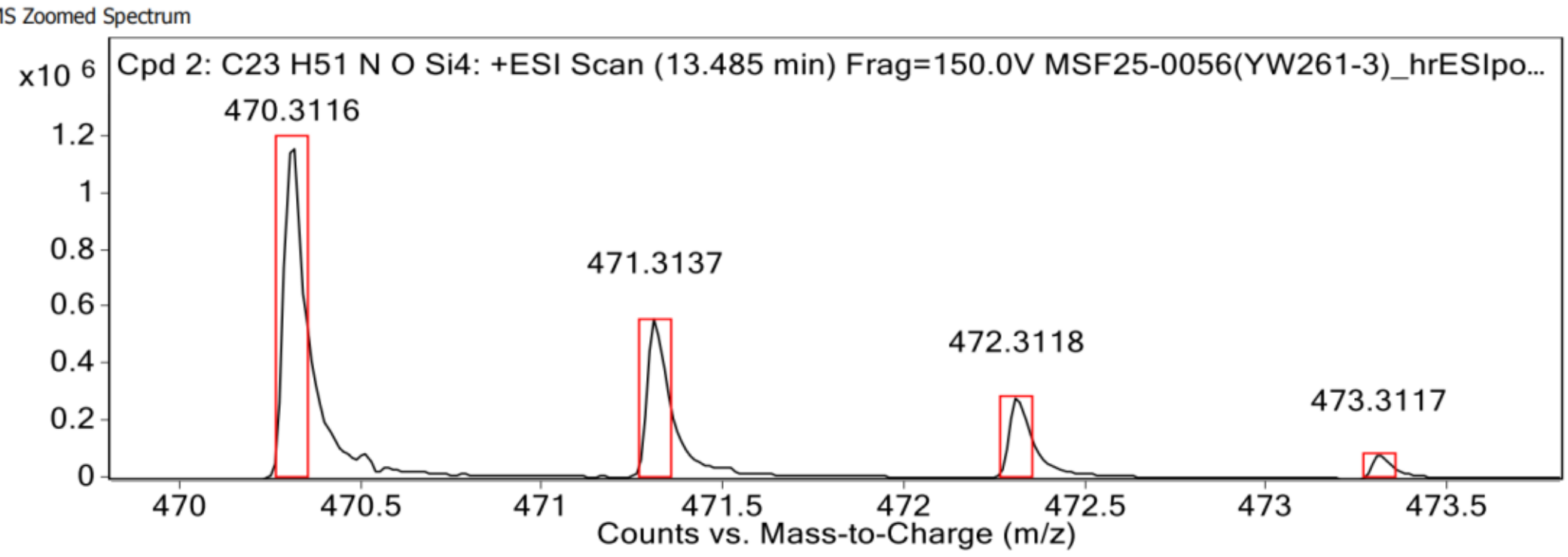

MS Spectrum Peak List

Obs. m/z	Calc. m/z	Charge	Abundance	Formula	Ion Species	Tgt Mass Error (ppm)
124.0861			1750776			
470.3116	470.3120	1	1183674	C23H51NOSi4	(M+H)+	1.04
471.3137	471.3137	1	565276	C23H51NOSi4	(M+H)+	0.03
472.3118	472.3118	1	288530	C23H51NOSi4	(M+H)+	-0.07
473.3117	473.3122	1	82170	C23H51NOSi4	(M+H)+	1.15

--- End Of Report ---

HRMS (ESI^+) calc. for **t5**, $C_{23}H_{51}NOSi_4$ $[M+H]^+$470.3120, found 470.3116.

Target Compound Screening Report

Results Acquired by The University of Texas at Austin Mass Spectrometry Facility

**Data File**	MSF25-0056(YW261-3)_hrESIposLC2.d	**Sample Name**	0056(YW261-3)	**Comment**	0056(YW261-3)
**Position**	P1-A8	**Instrument Name**	6530	**User Name**	
**Acq Method**	LC_C18_pos_jl.m	**Acquired Time**	12/5/2025 3:06:27 PM	**DA Method**	MSF.m

MS Zoomed Spectrum

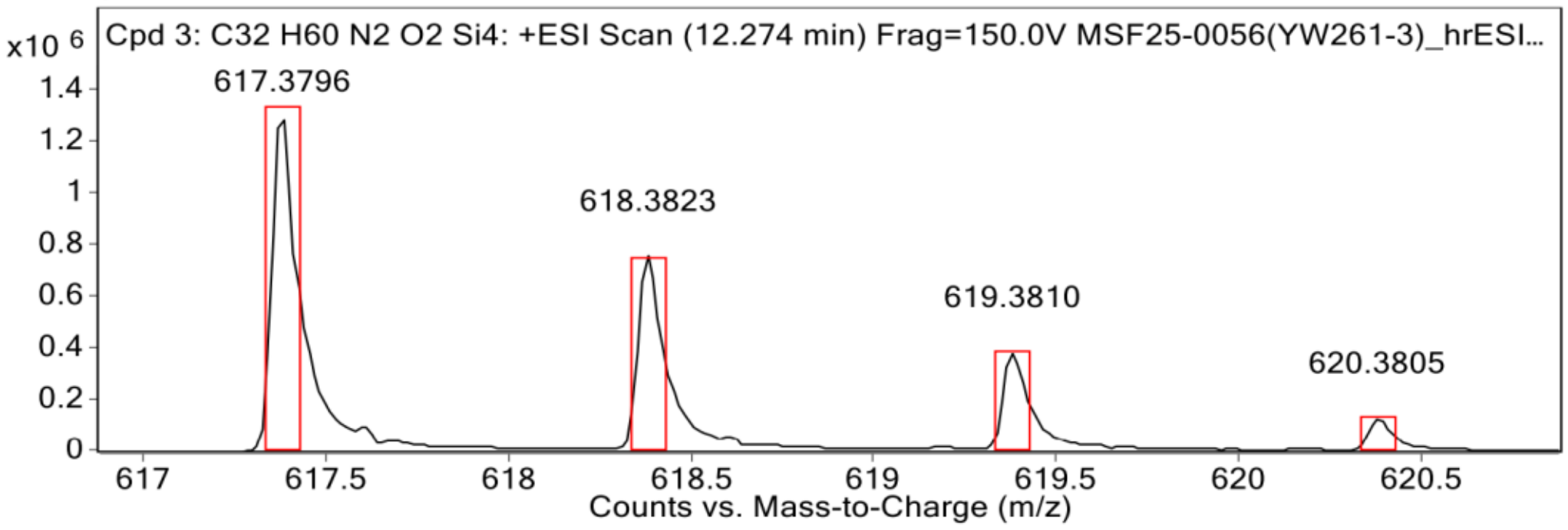

MS Spectrum Peak List

Obs. m/z	Calc. m/z	Charge	Abundance	Formula	Ion Species	Tgt Mass Error (ppm)
551.3322			4986938			
617.3796	617.3805	1	1303790	C32H60N2O2Si4	(M+H)+	1.46
618.3823	618.3824	1	758969	C32H60N2O2Si4	(M+H)+	0.19
619.3810	619.3812	1	387841	C32H60N2O2Si4	(M+H)+	0.32
620.3805	620.3816	1	133016	C32H60N2O2Si4	(M+H)+	1.83

--- End Of Report ---

HRMS (ESI^+) calc. for **t6**, $C_{32}H_{60}N_2O_2Si_4$ $[M+H]^+$617.3805, found 617.3796.

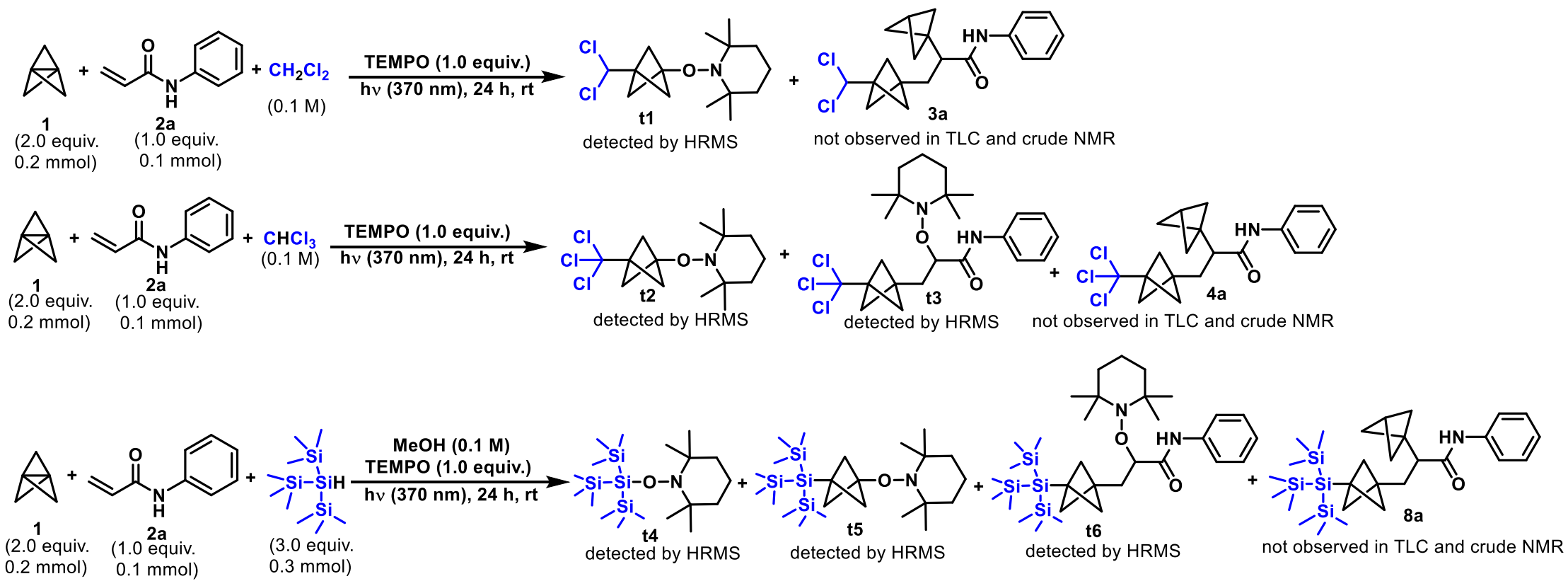

The three MCRs were completely suppressed in the presence of TEMPO as radical scavenger, indicating that the formation of **3a**, **4a**, and **8a** proceeds through a radical pathway. The detection of TEMPO adducts **t1**-**t6** and radical clock products **3af**, **4aa**, **8p** and **8q** provide direct evidence for the generation of dichloromethyl, trichloromethyl, and silyl radicals, as well as their subsequent addition to propellane.

Radical Clock experiments

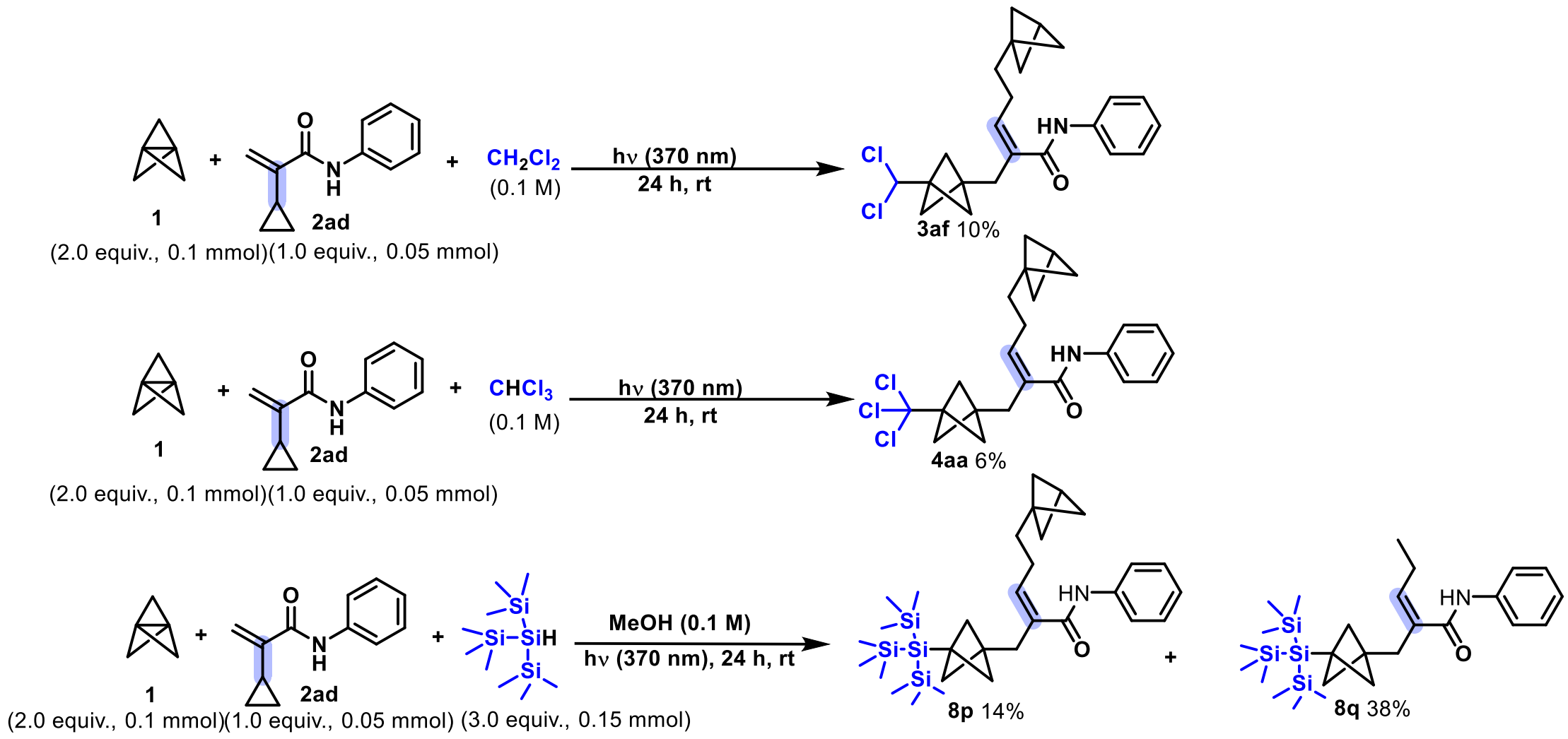

Deuterium labelling experiments

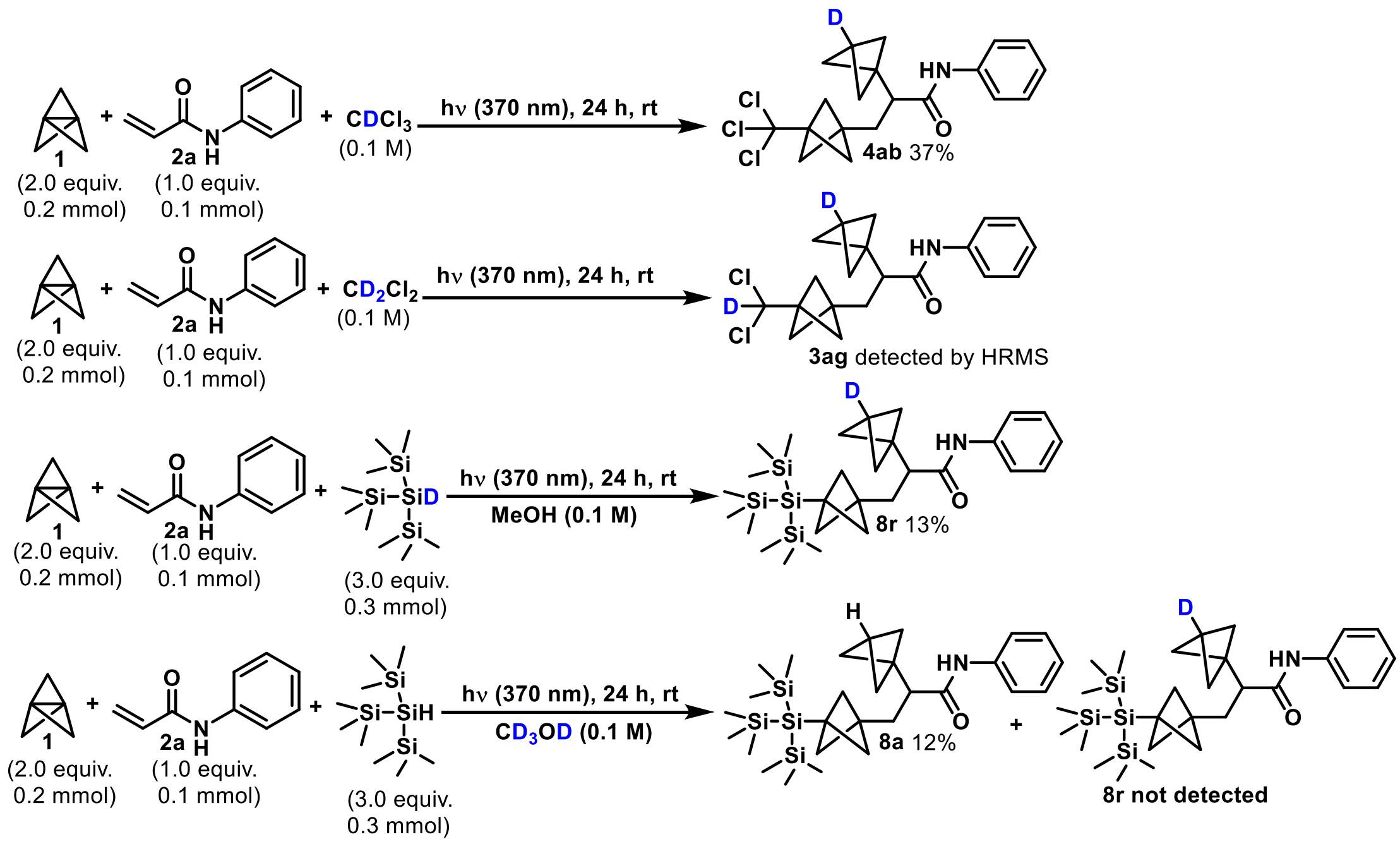

Replacing $CHCl_3$ with $CDCl_3$ resulted in complete deuterium incorporation in product **4ab** with 37% yield (see section 5: NMR spectra of novel compounds), demonstrating that the hydrogen atom in the second BCP core originates from $CHCl_3$. By contrast, substituting CH_2Cl_2 with CD_2Cl_2 resulted in only trace levels of deuterium incorporation in the corresponding product, as determined by HRMS analysis. This outcome indicates that the C–D bonds in CD_2Cl_2 are substantially stronger than the corresponding C–H bonds (C-H BDE of CH_2Cl_2 is approximately 95 to 97 kcal/mol),[18] making them markedly less prone to radical-mediated deuterium atom abstraction. The silylation reaction proceeded with approximately 65% deuterium incorporation, as determined by integration of the singlet at 2.49 ppm, thereby corroborating the role of tris(trimethylsilyl)silane as the hydrogen atom donor. Notably, employing $(CH_3Si)_3SiD$

led to the formation of **8r** in a substantially diminished yield of 13%, indicating that the deuterated silane functions as a less effective hydrogen atom donor than non-deuterated $(CH_3Si)_3SiH$ (Si-H BDE is about 83.7 Kcal/mol).[19]

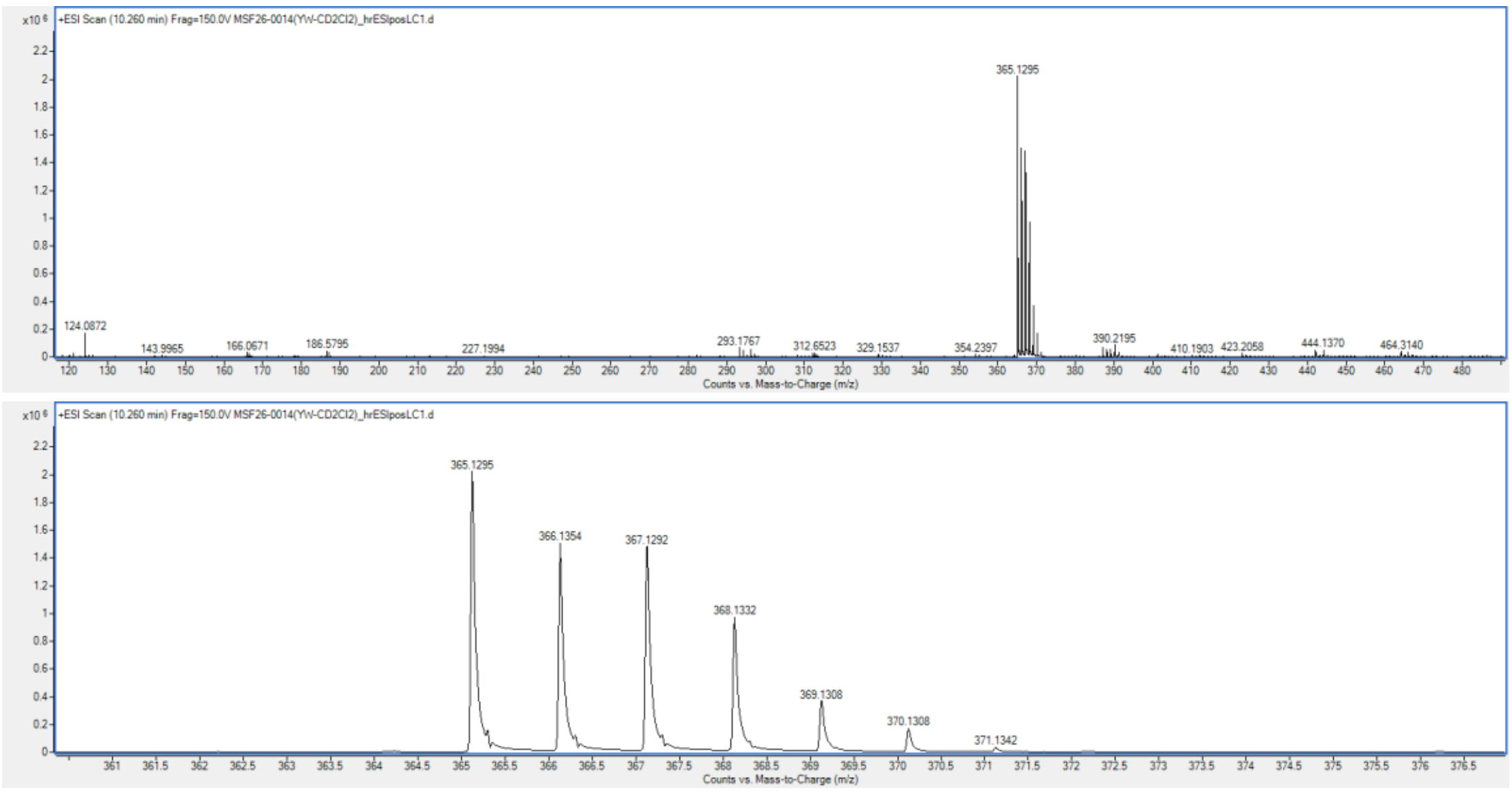

HRMS (ESI$^+$) calc. for **3ag** $C_{20}H_{21}D_2Cl_2NO$ $[M+H]^+$366.1355, found 366.1354. Note: The CD_2Cl_2 reaction mixture also contains $C_{20}H_{22}DCl_2NO$, calc. for $C_{20}H_{22}DCl_2NO$ $[M+H]^+$365.1292, found 365.1295.

^{1}H NMR (600 MHz, CDCl$_3$, 65% D incorporation)

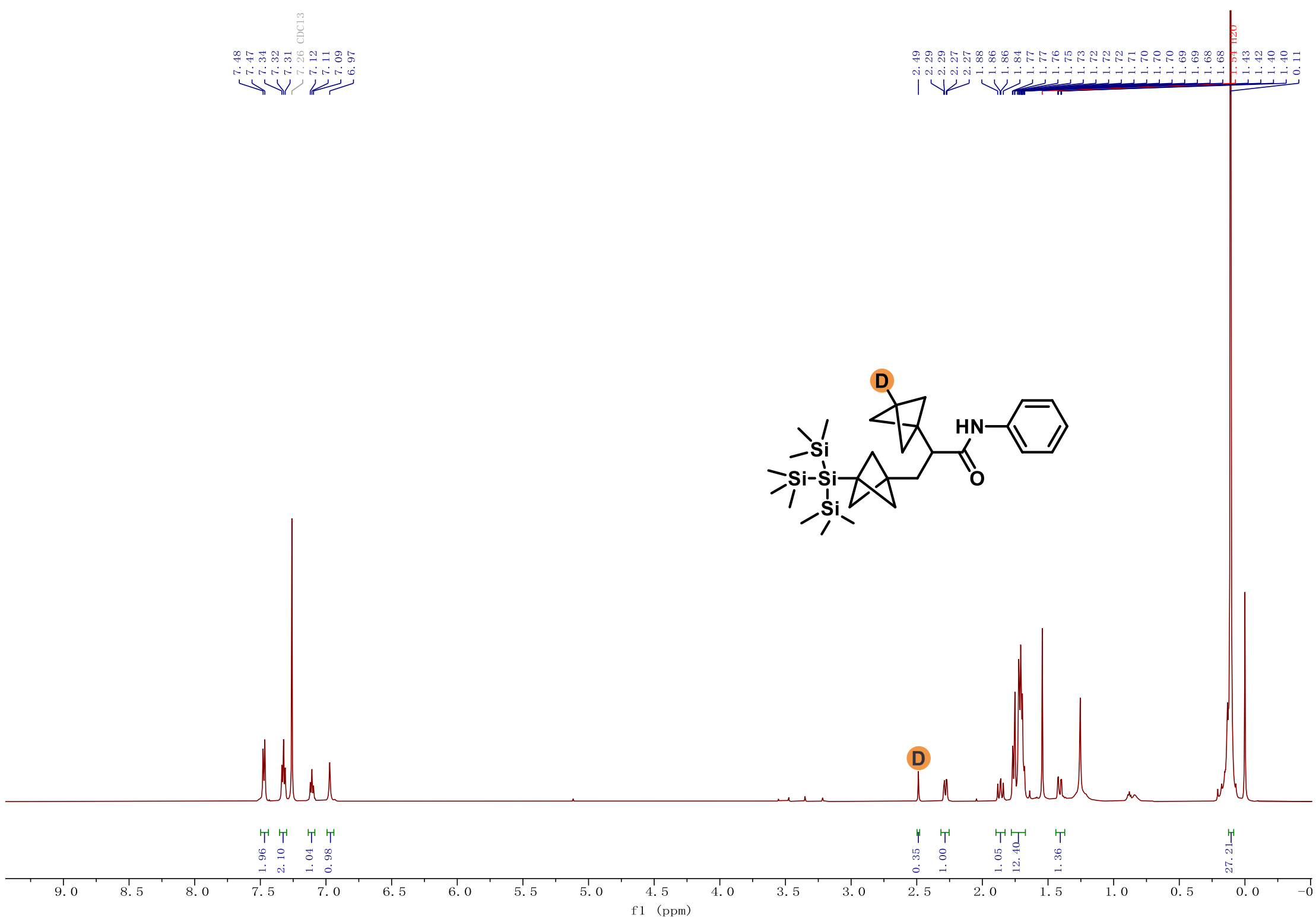

UV-Vis study

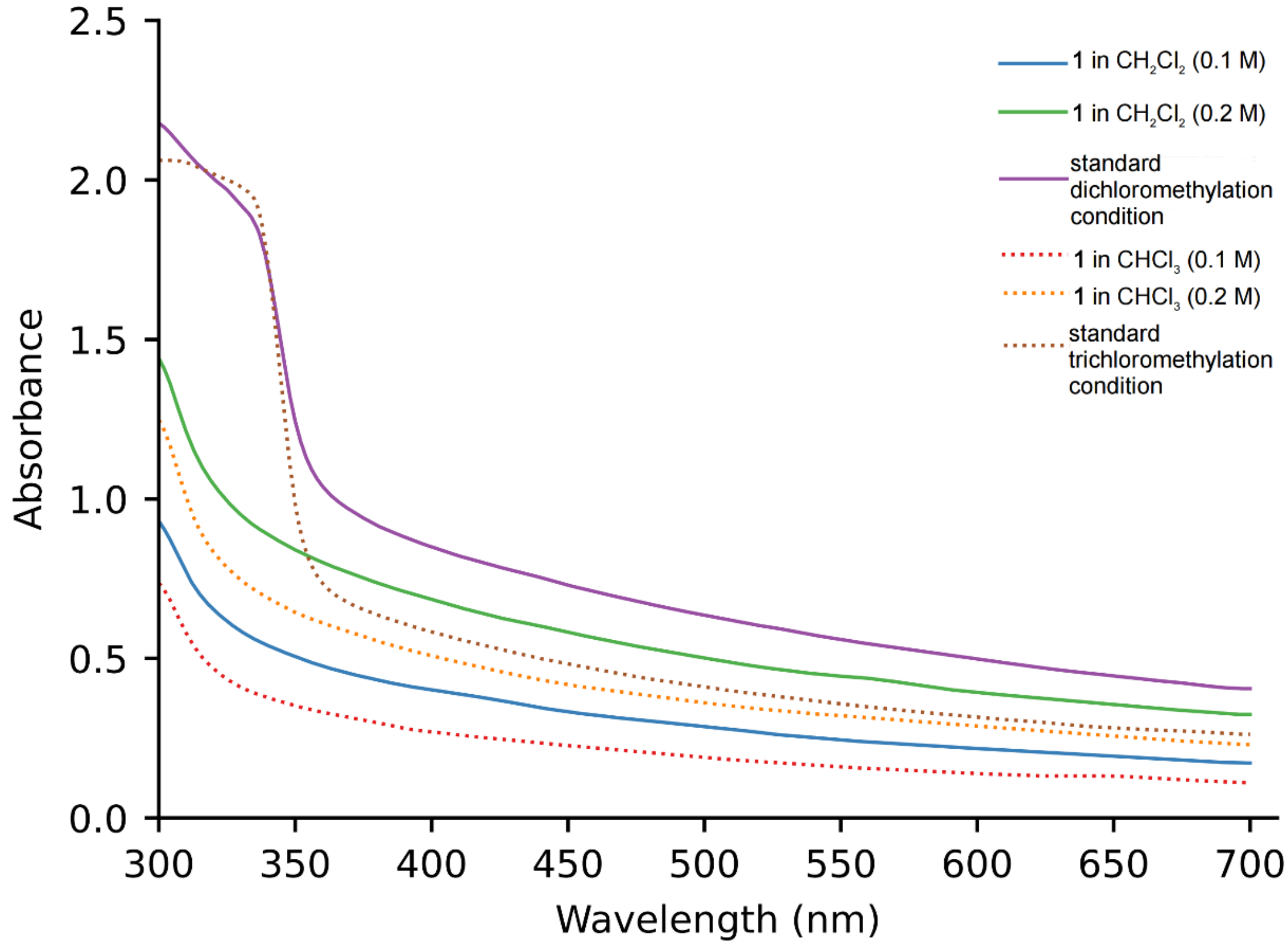

Standard dichloromethylation condition sample preparation: mix acrylamide **2a** (0.1 mmol, 1.0 equiv.) + [1.1.1]propellane **1** (0.2 mmol, 2.0 equiv.) in CH_2Cl_2 solution (0.1 M) in a reaction vial for UV measurement

Standard trichloromethylation condition sample preparation: mix acrylamide **2a** (0.1 mmol, 1.0 equiv.) + [1.1.1]propellane **1** (0.2 mmol, 2.0 equiv.) in $CHCl_3$ solution (0.1 M) in a reaction vial for UV measurement

These observations of TEMPO adduct, together with the results of the radical clock, deuterium-labelling experiments, UV spectra and DFT calculation, support a radical chain mechanism for all three transformations. On the basis of these experimental findings, a plausible sequence of initiation and propagation steps is proposed, as outlined below.

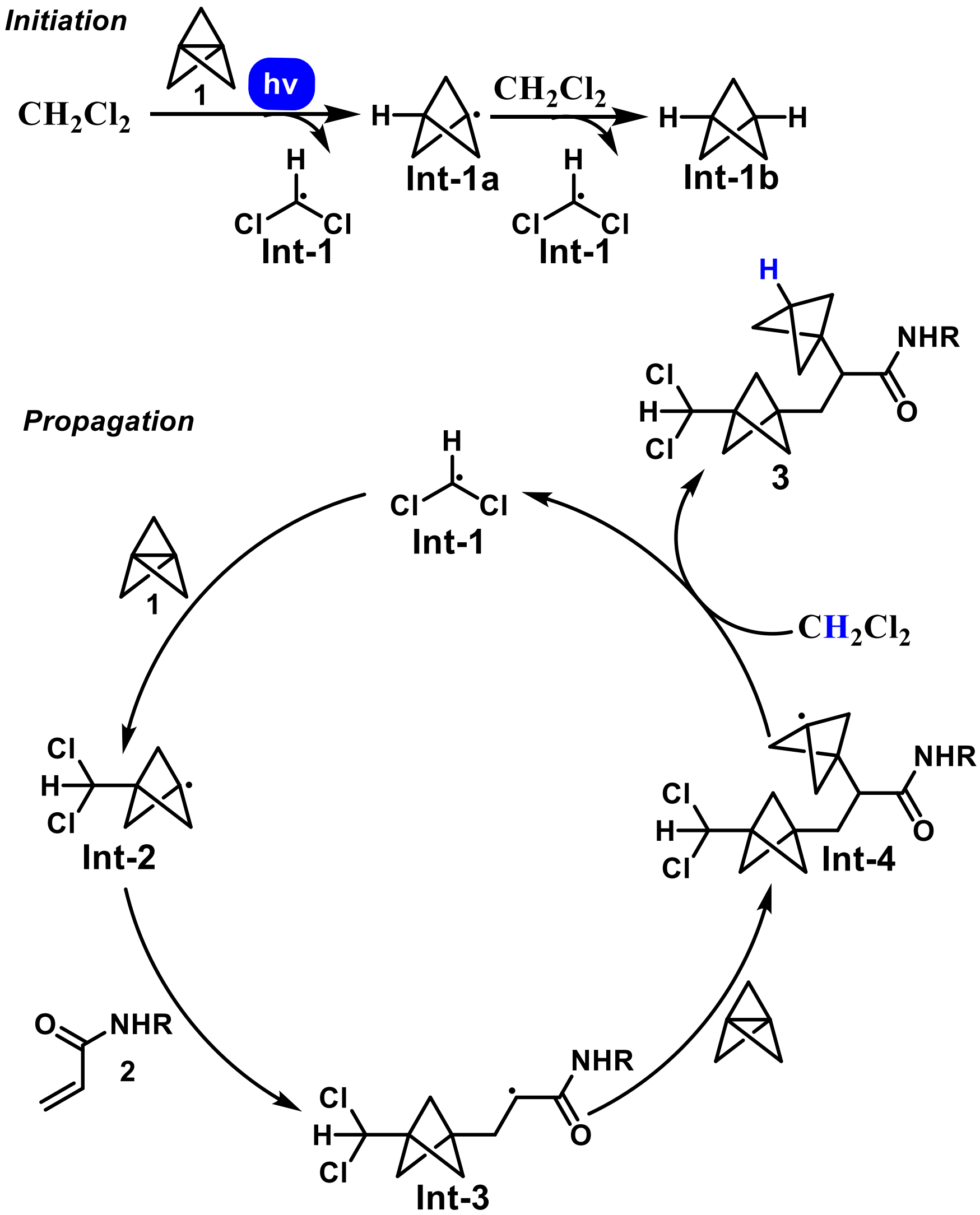

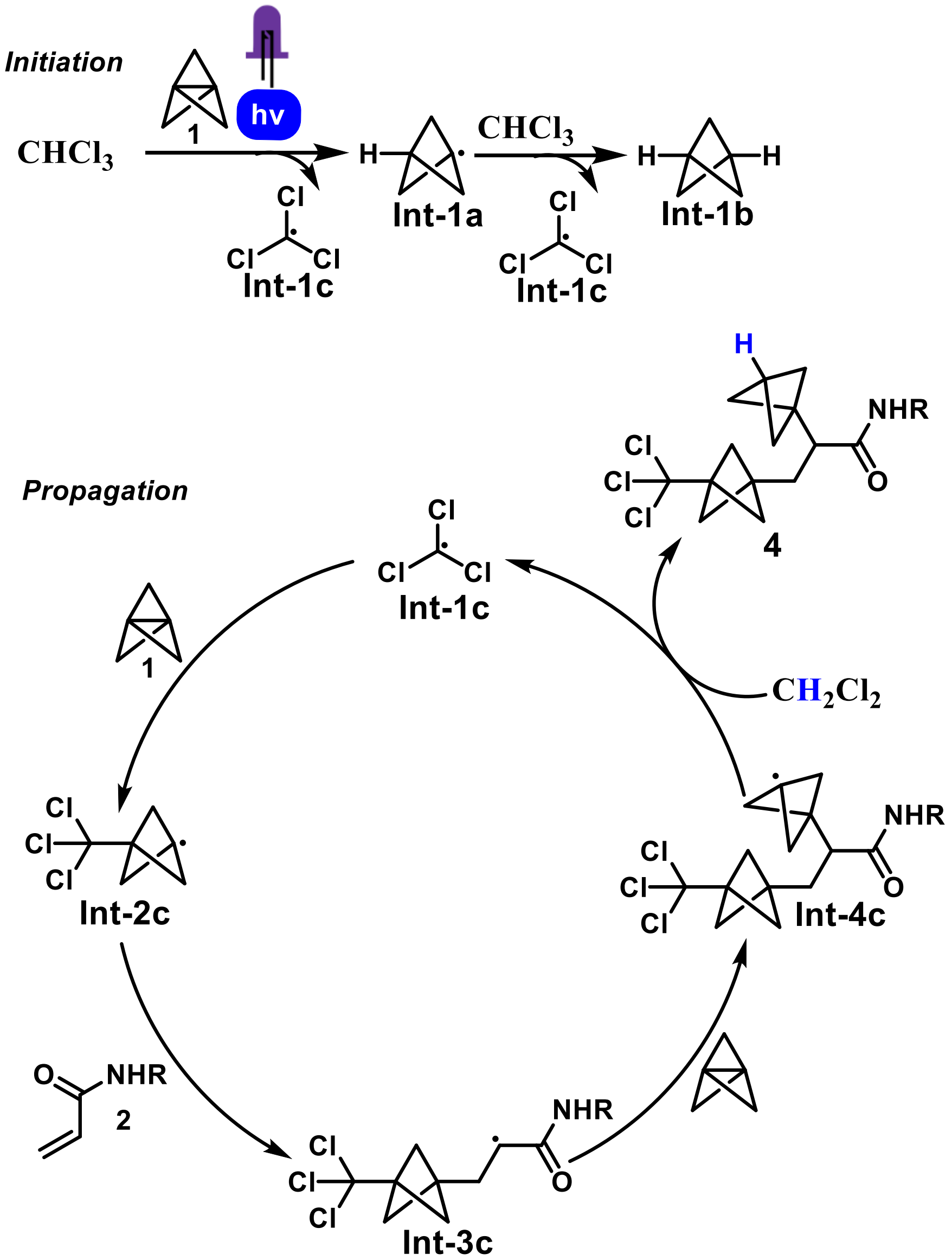
Initiation
CHCl3
1
hv
H
Int-1a
CHCl3
H
H
Int-1b
Cl
Cl
Cl
Int-1c
Cl
Cl
Cl
Int-1c
Propagation
Cl
Cl
Cl
Int-1c
1
Cl
Cl
Cl
Int-2c
O
NHR
2
Cl
Cl
Cl
NHR
O
Int-3c
Cl
Cl
Cl
NHR
O
Int-4c
CH2Cl2
H
Cl
Cl
Cl
NHR
O
4

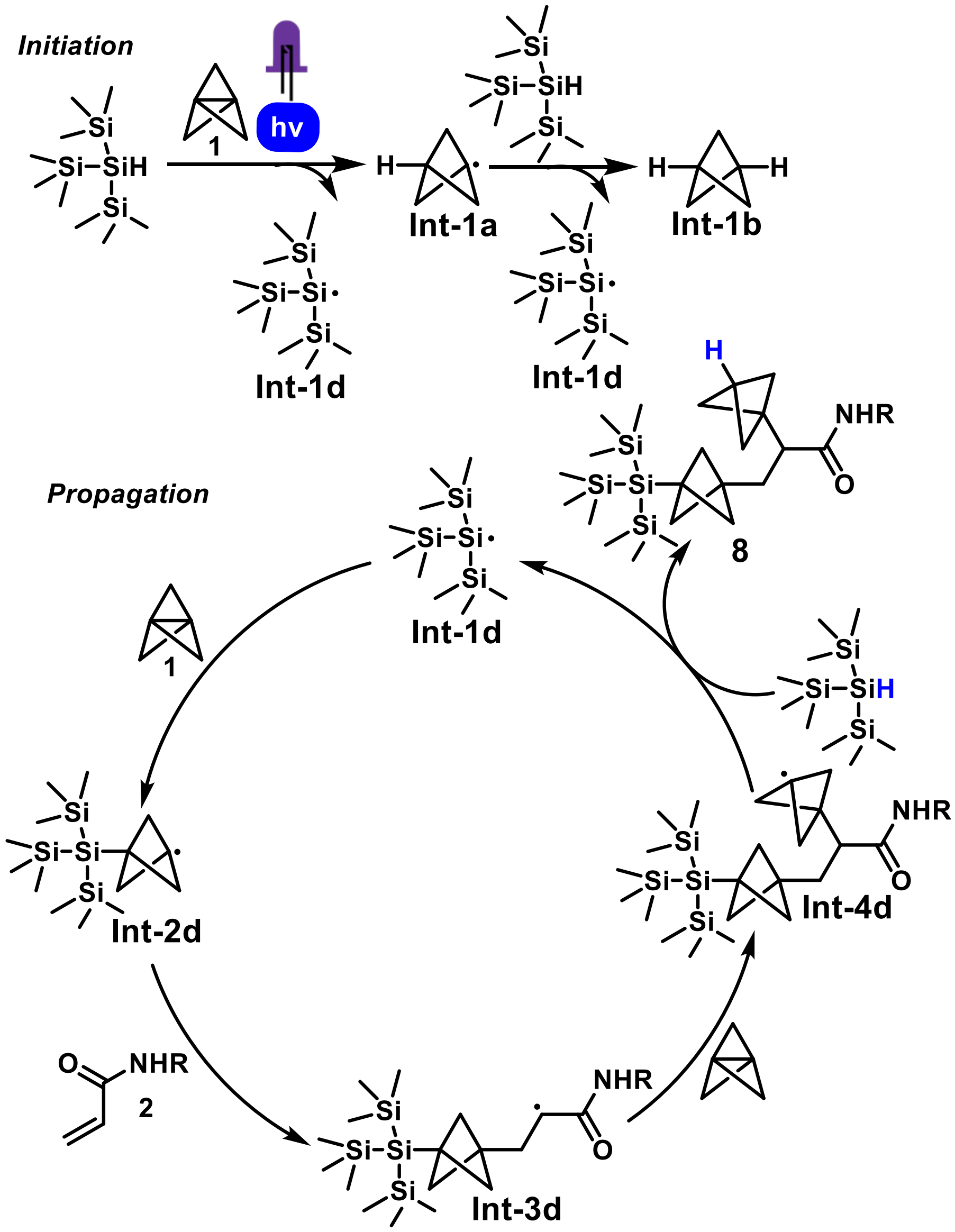

Initiation
hν
1
Int-1a
Int-1b
Int-1d
Int-1d
Propagation
Int-1d
8
NHR
1
Int-2d
Int-4d
2
Int-3d

5. NMR spectra of novel compounds.

(*S*)-3-acryloyl-5-isopropyloxazolidin-2-one (2u)

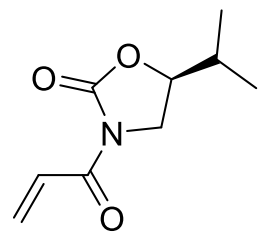

^{1}H NMR (600 MHz, $CDCl_3$)

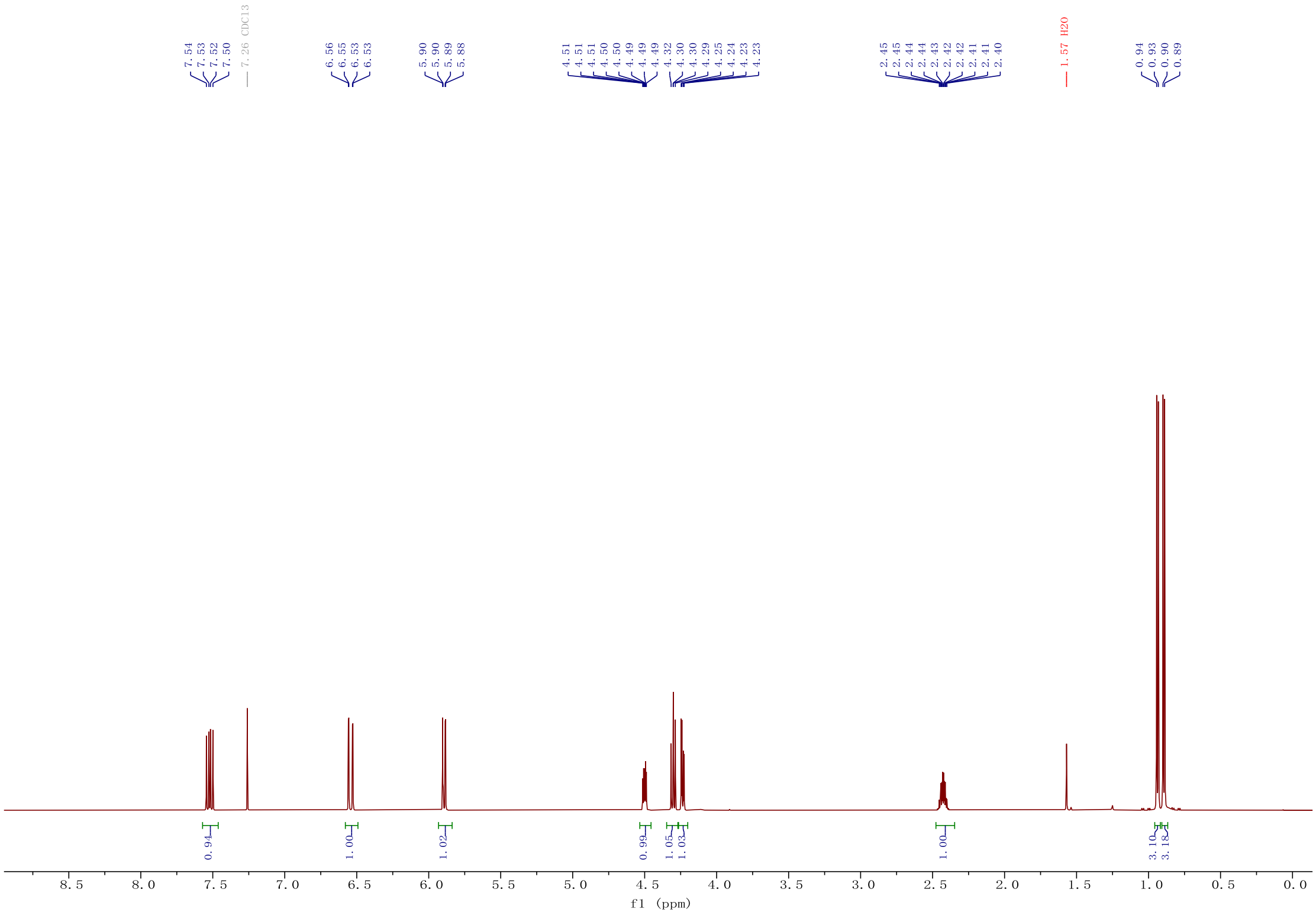

^{13}C NMR (151 MHz, $CDCl_3$)

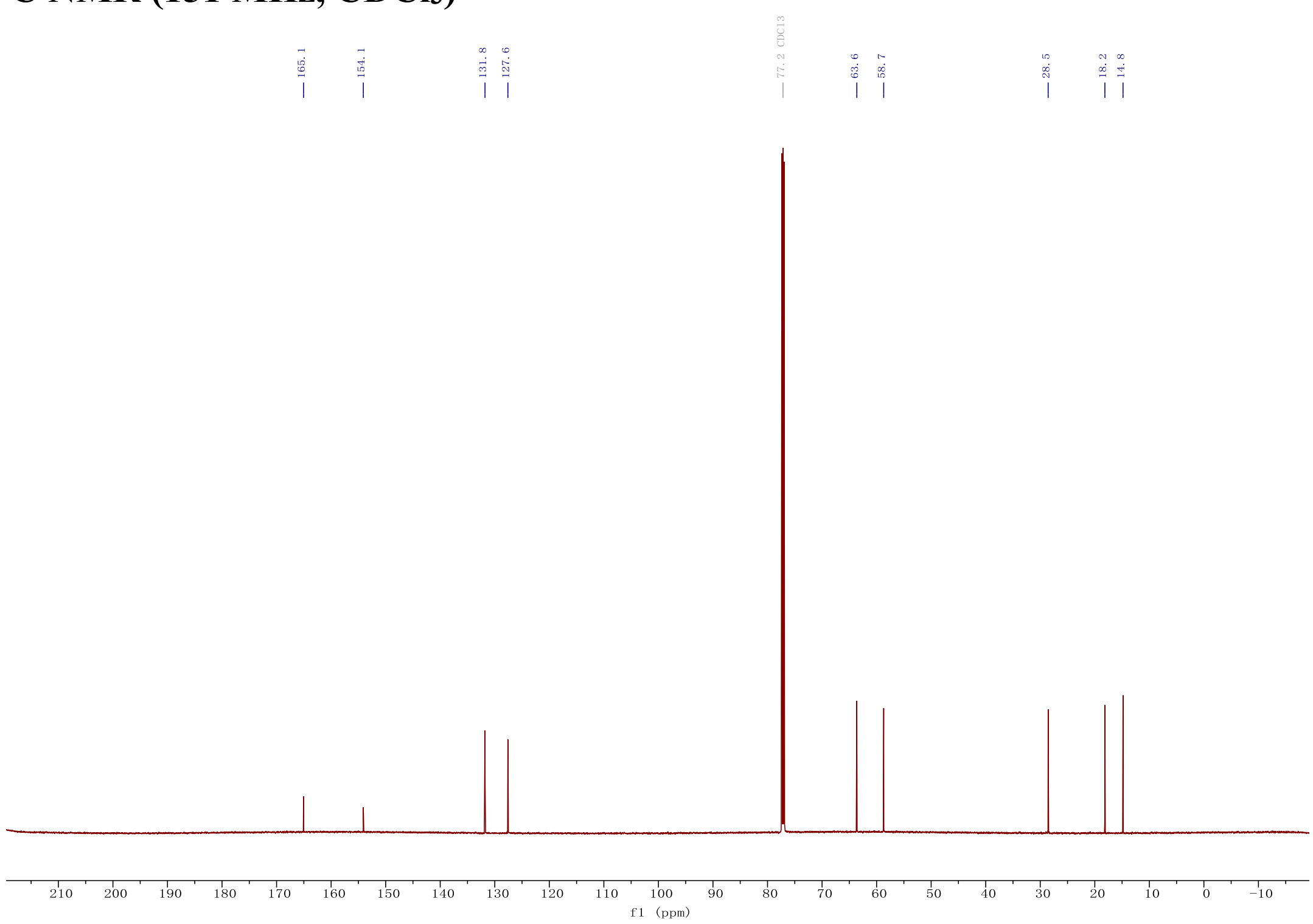

methyl acryloyl-*L*-phenylalanyl-*L*-leucinate (2v)

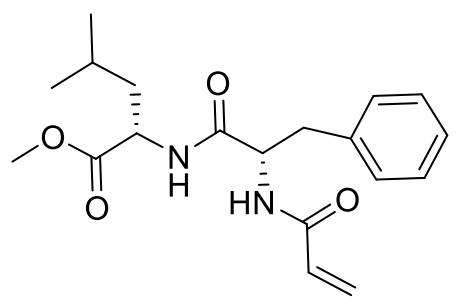

^{1}H NMR (600 MHz, CDCl$_3$)

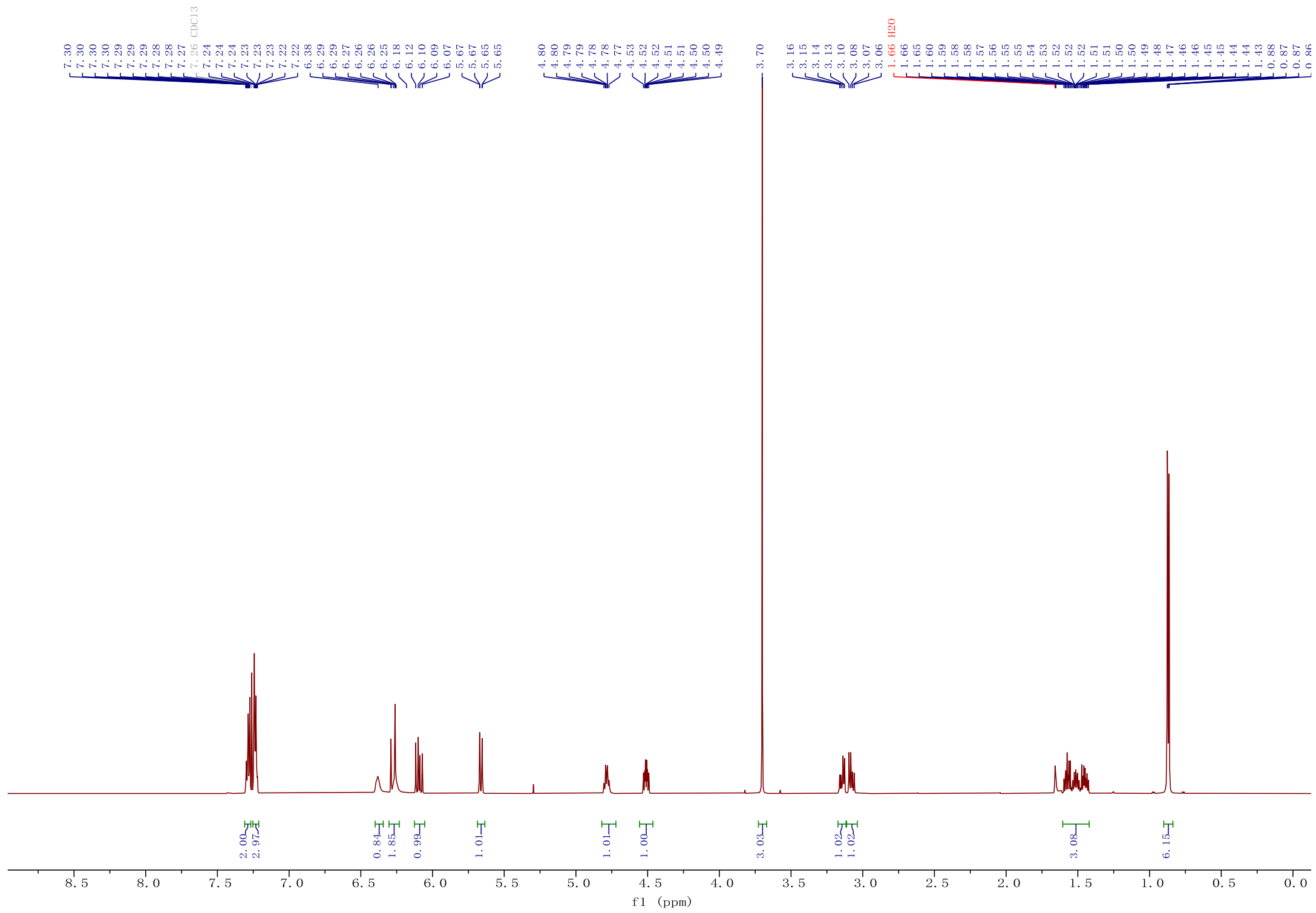

^{13}C NMR (151 MHz, CDCl$_3$)

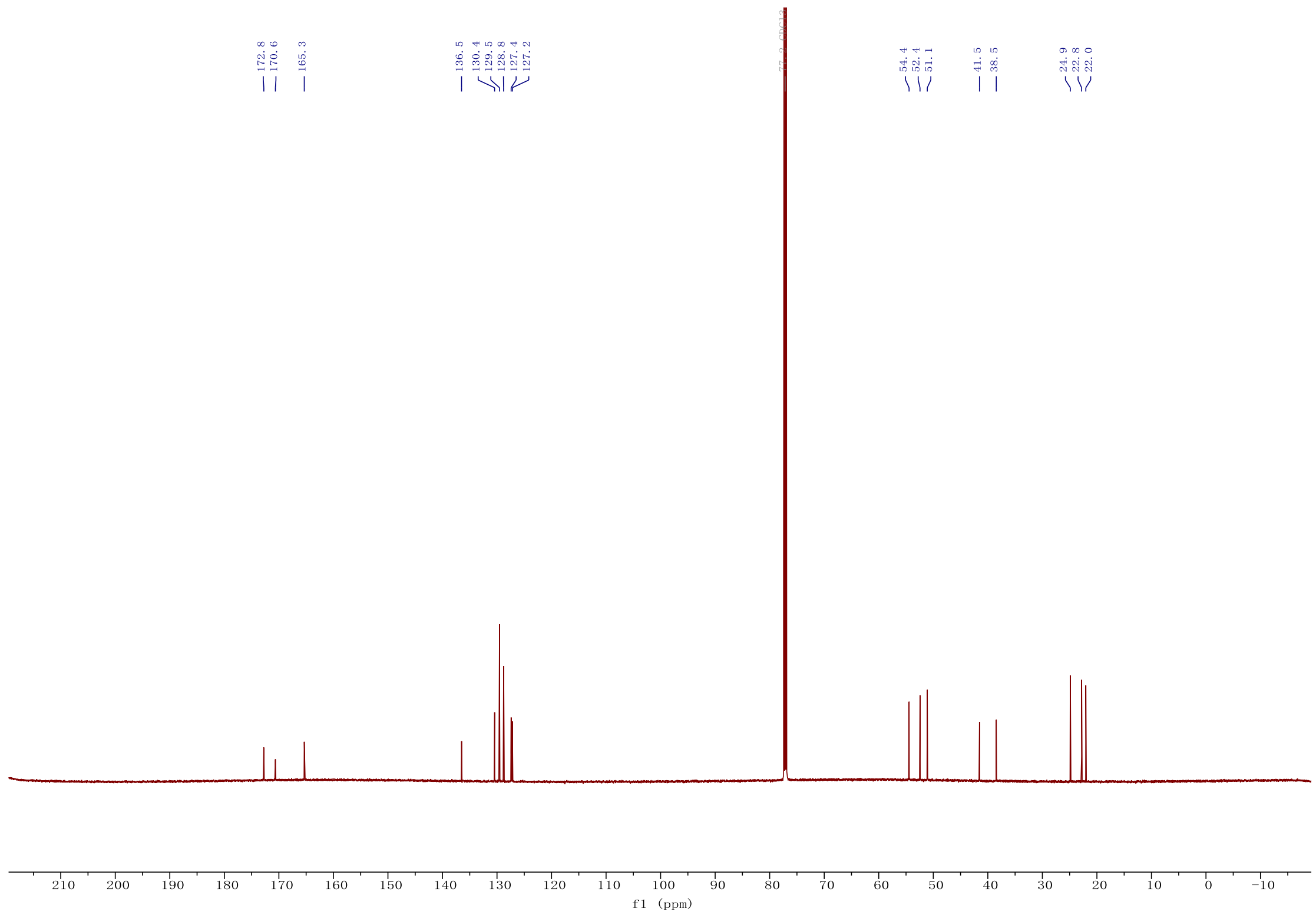

2-(bicyclo[1.1.1]pentan-1-yl)-3-(3-(dichloromethyl)bicyclo[1.1.1]pentan-1-yl)-*N*-phenylpropanamide (3a)

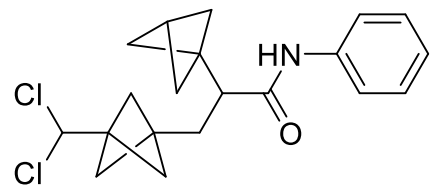

^{1}H NMR (600 MHz, CDCl3)

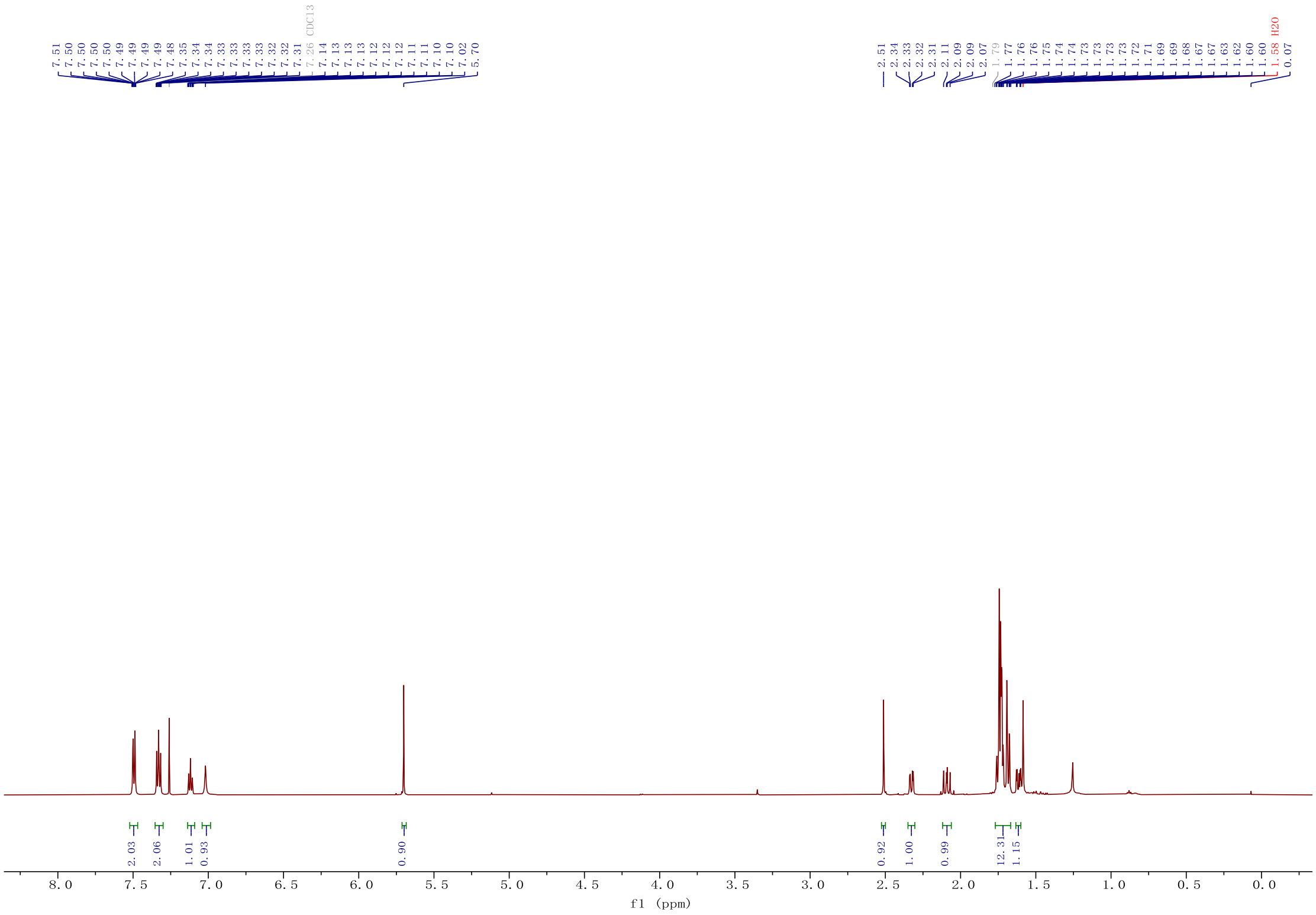

^{13}C NMR (151 MHz, CDCl3)

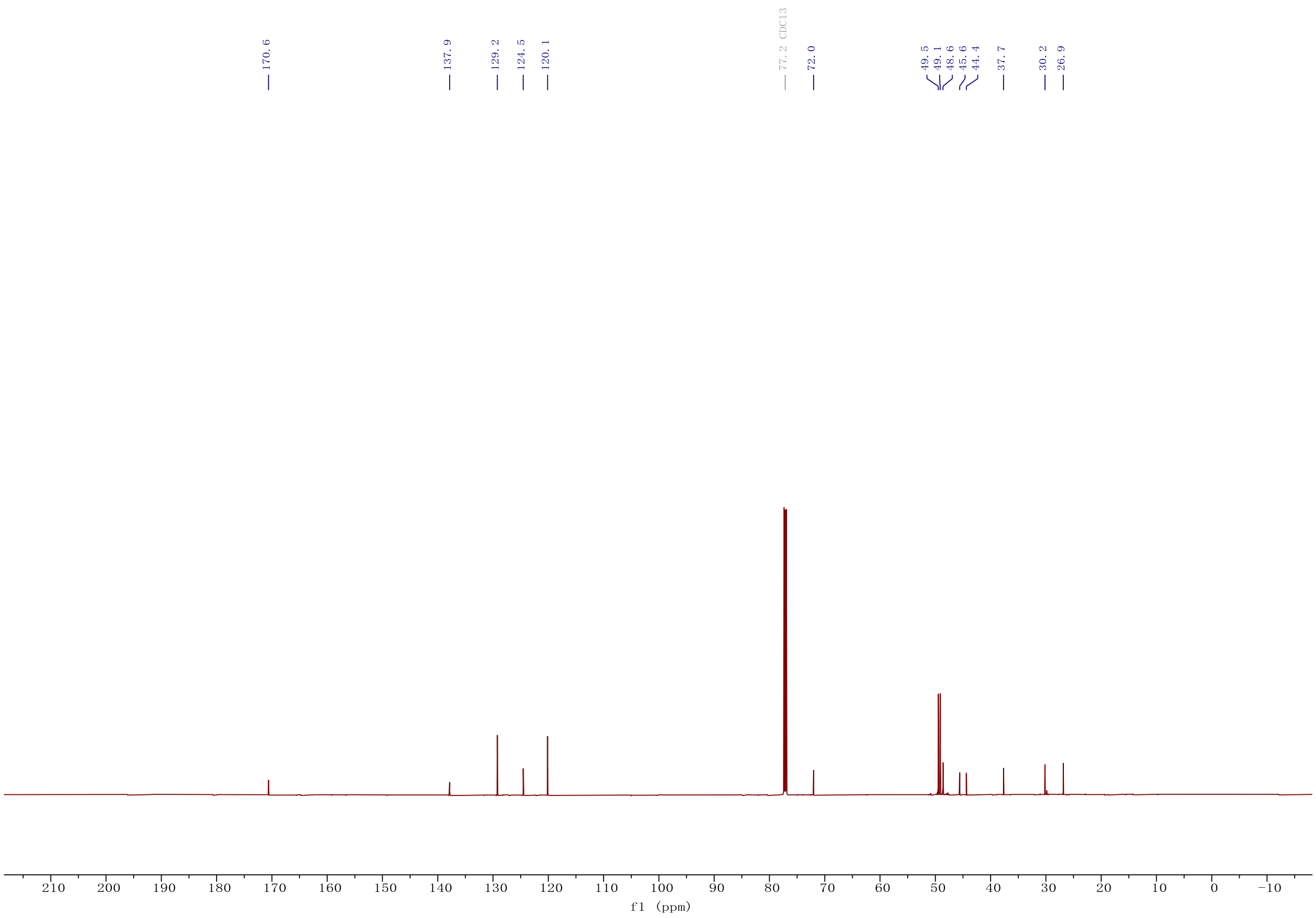

2-(bicyclo[1.1.1]pentan-1-yl)-3-(3-(dichloromethyl)bicyclo[1.1.1]pentan-1-yl)-*N*-(4-fluorophenyl)propenamide (3b)

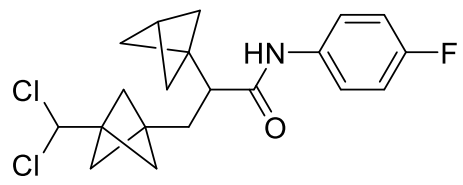

^{1}H NMR (600 MHz, CDCl3)

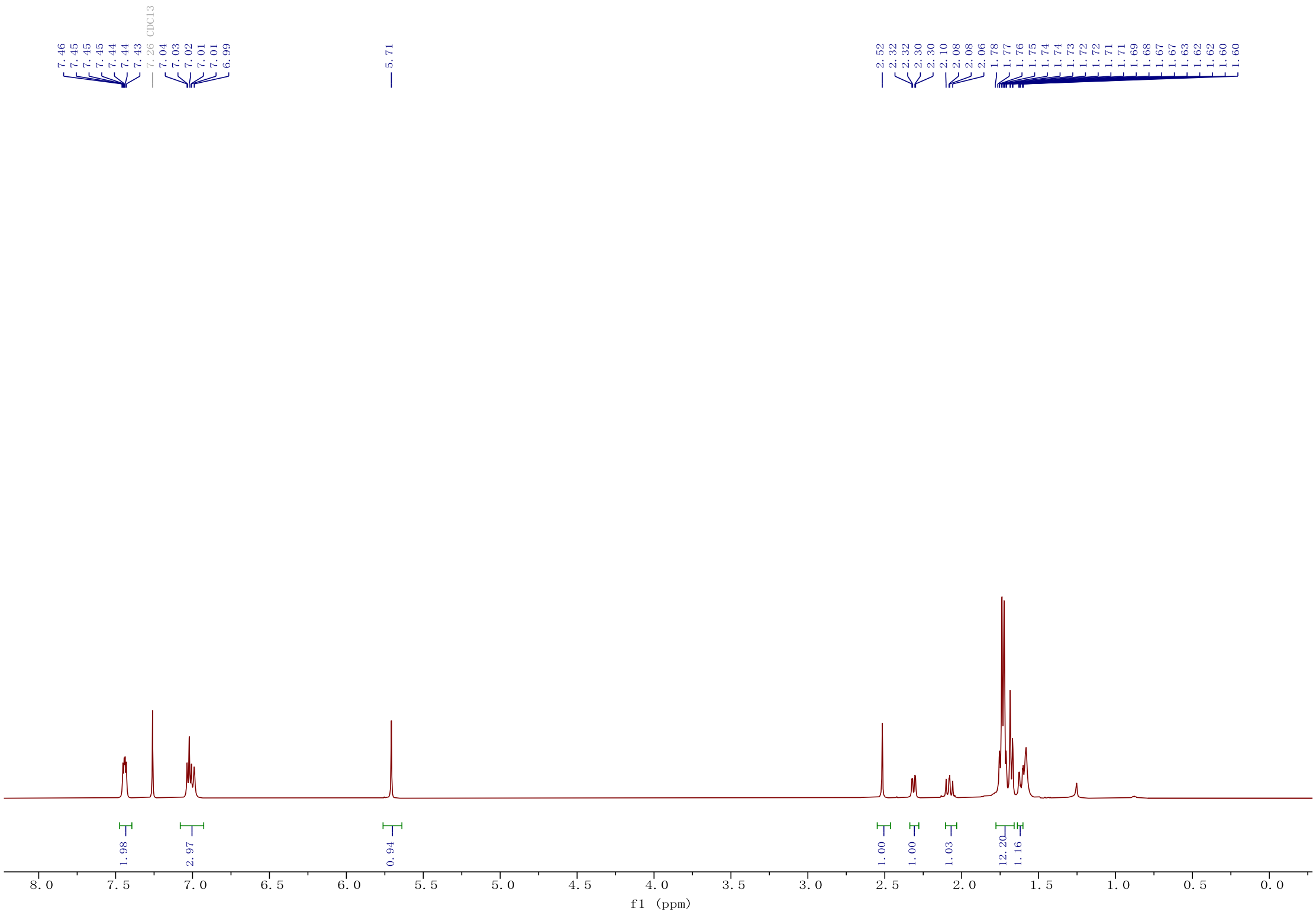

^{13}C NMR (151 MHz, CDCl3)

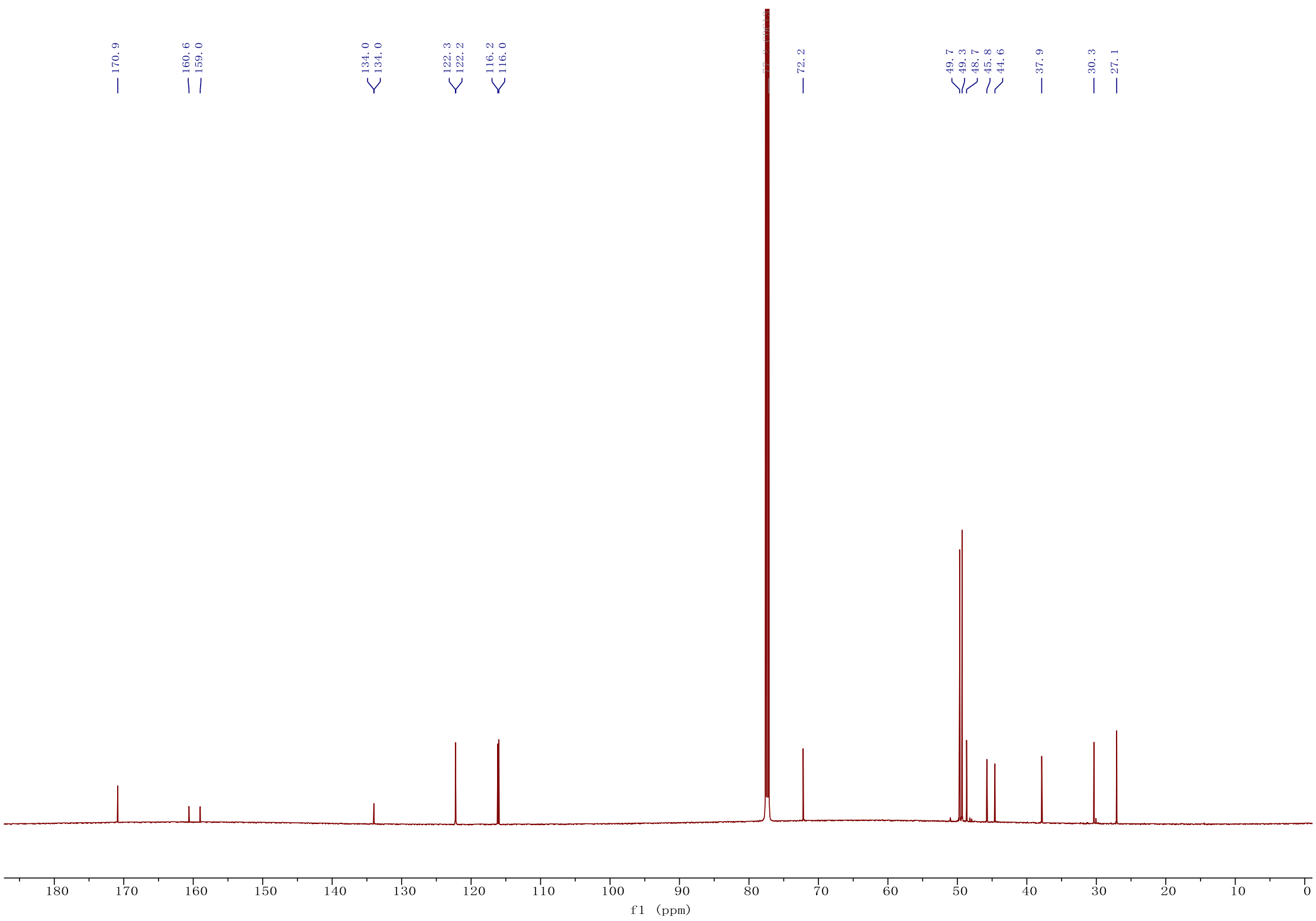

2-(bicyclo[1.1.1]pentan-1-yl)-3-(3-(dichloromethyl)bicyclo[1.1.1]pentan-1-yl)-*N*-(p-tolyl)propenamide (3c)

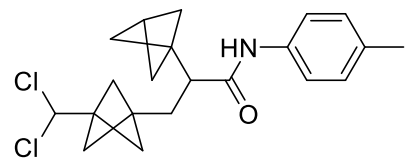

^{1}H NMR (600 MHz, CDCl$_3$)

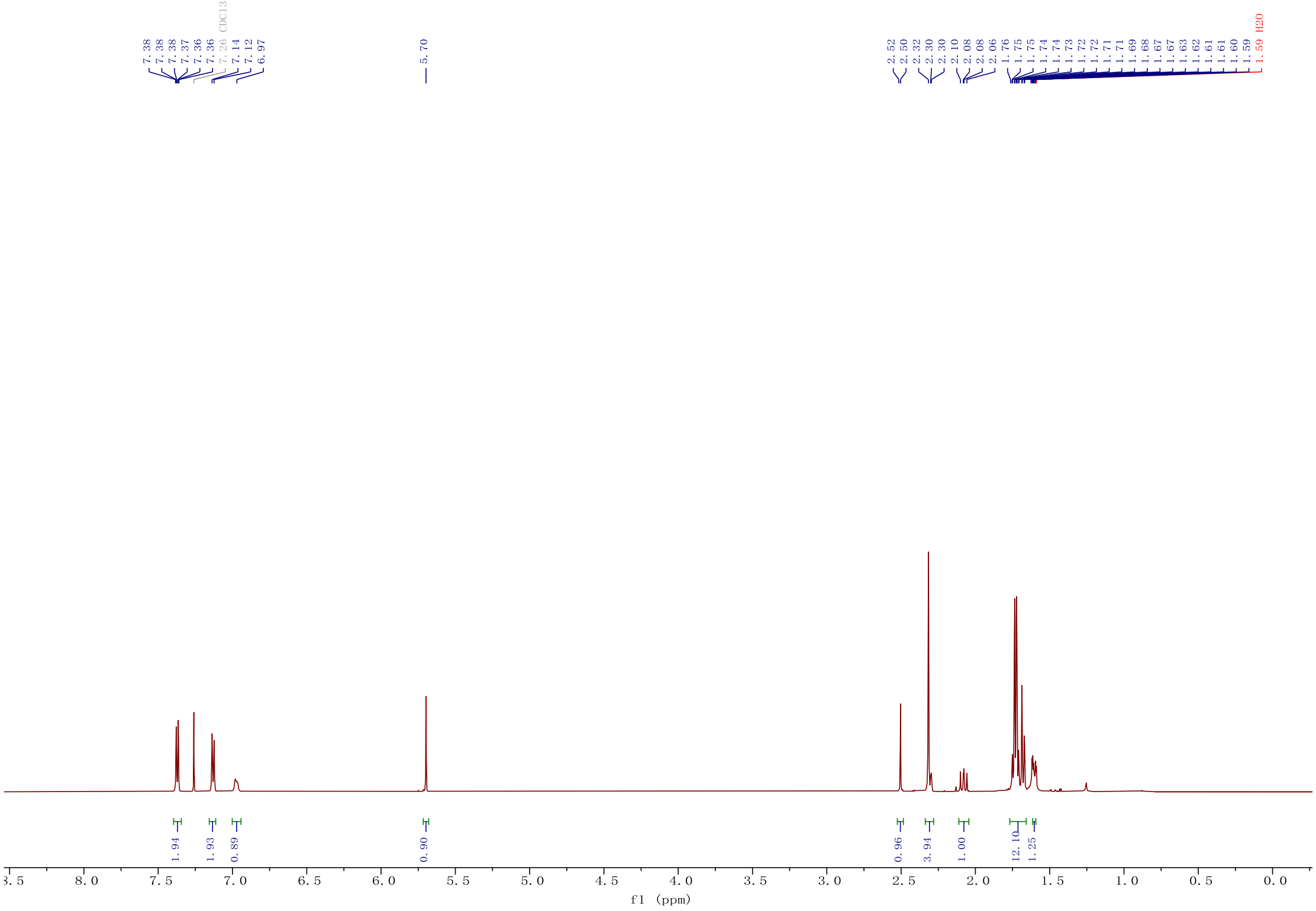

^{13}C NMR (151 MHz, CDCl$_3$)

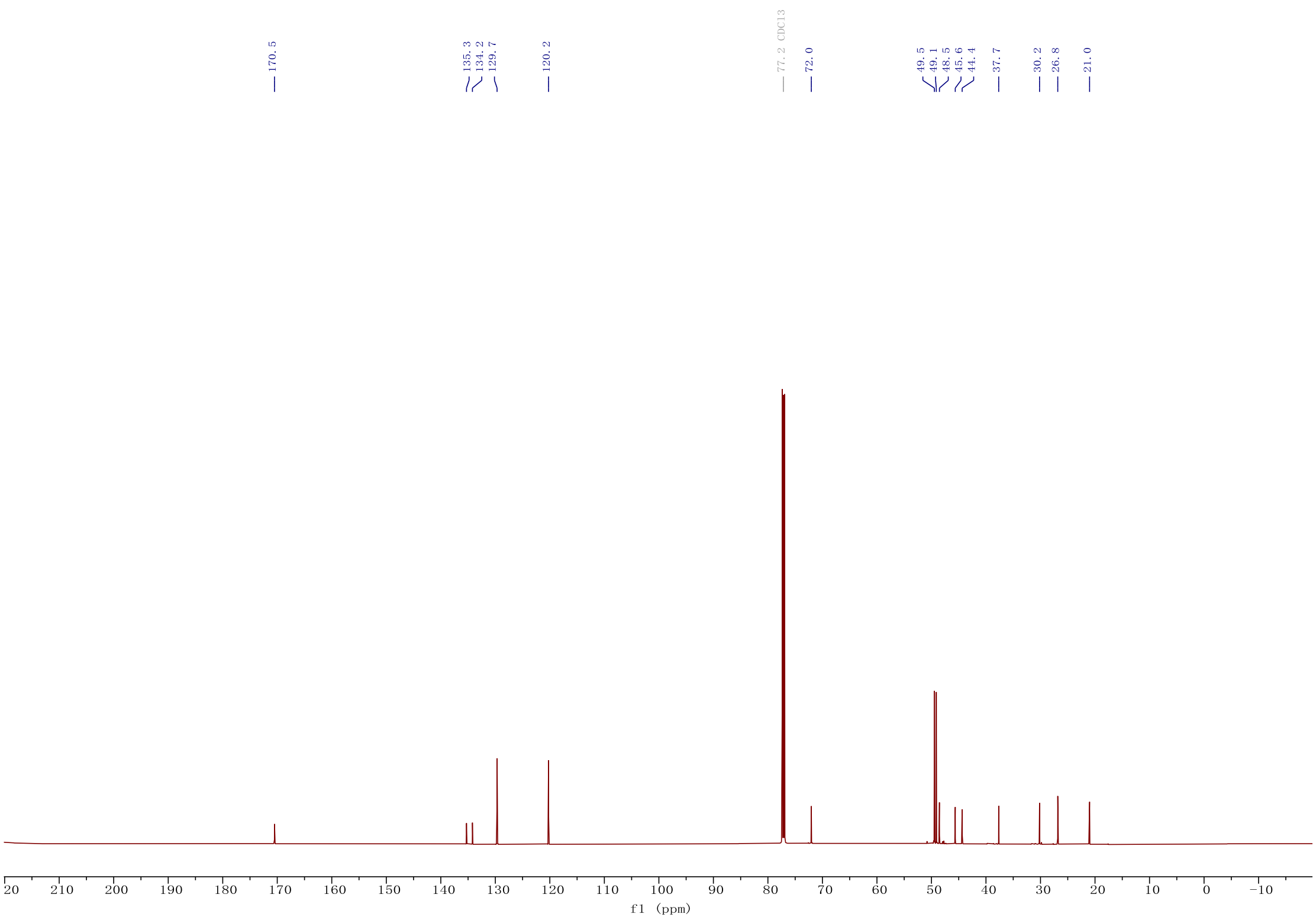

2-(bicyclo[1.1.1]pentan-1-yl)-*N*-(4-(tert-butyl)phenyl)-3-(3-(dichloromethyl)bicyclo[1.1.1]pentan-1-yl)propenamide (3d)

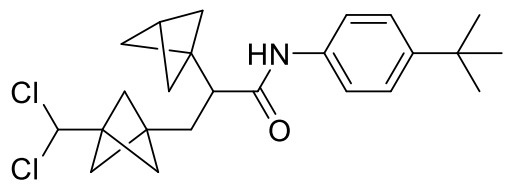

^{1}H NMR (600 MHz, $CDCl_3$)

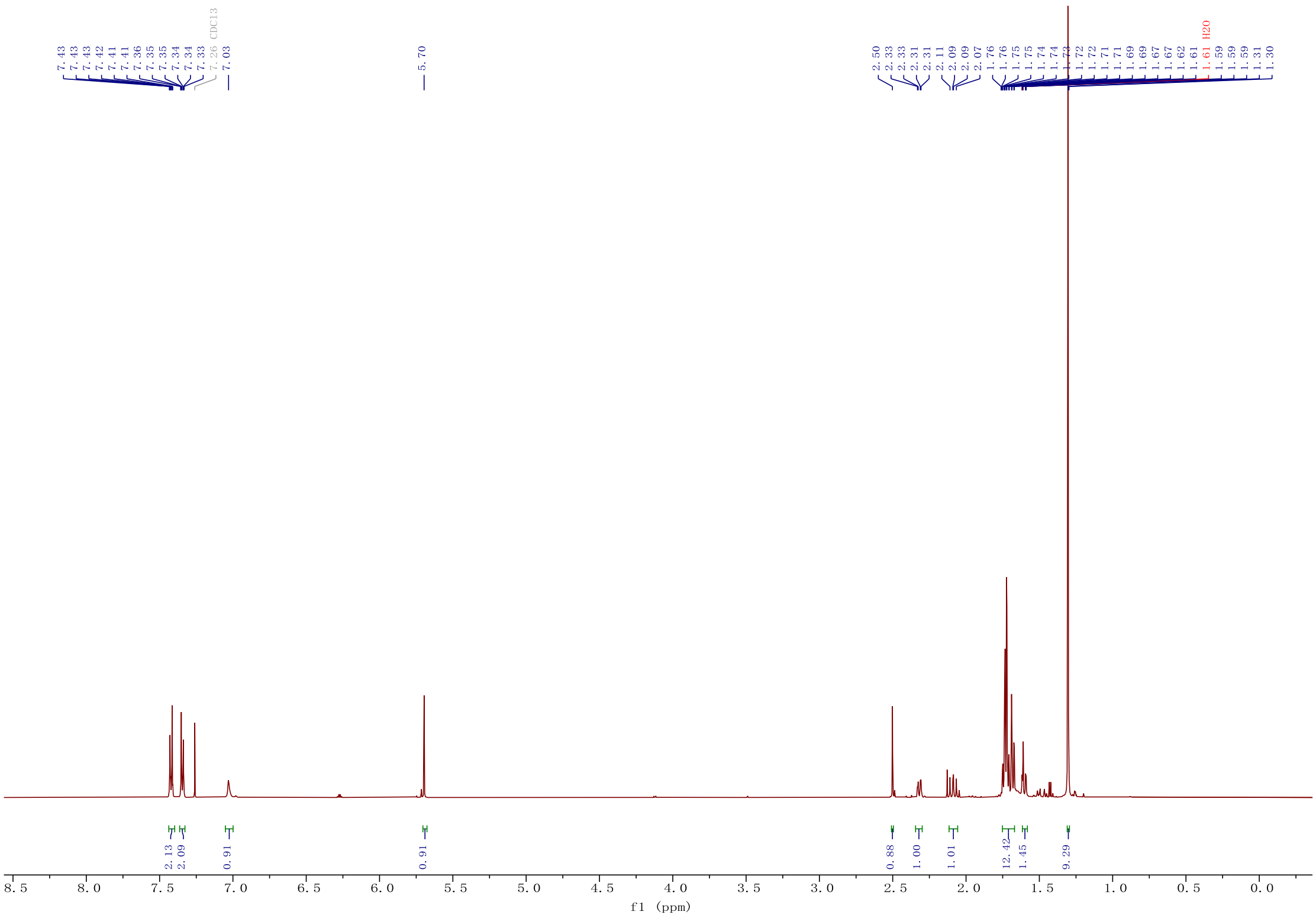

^{13}C NMR (151 MHz, $CDCl_3$)

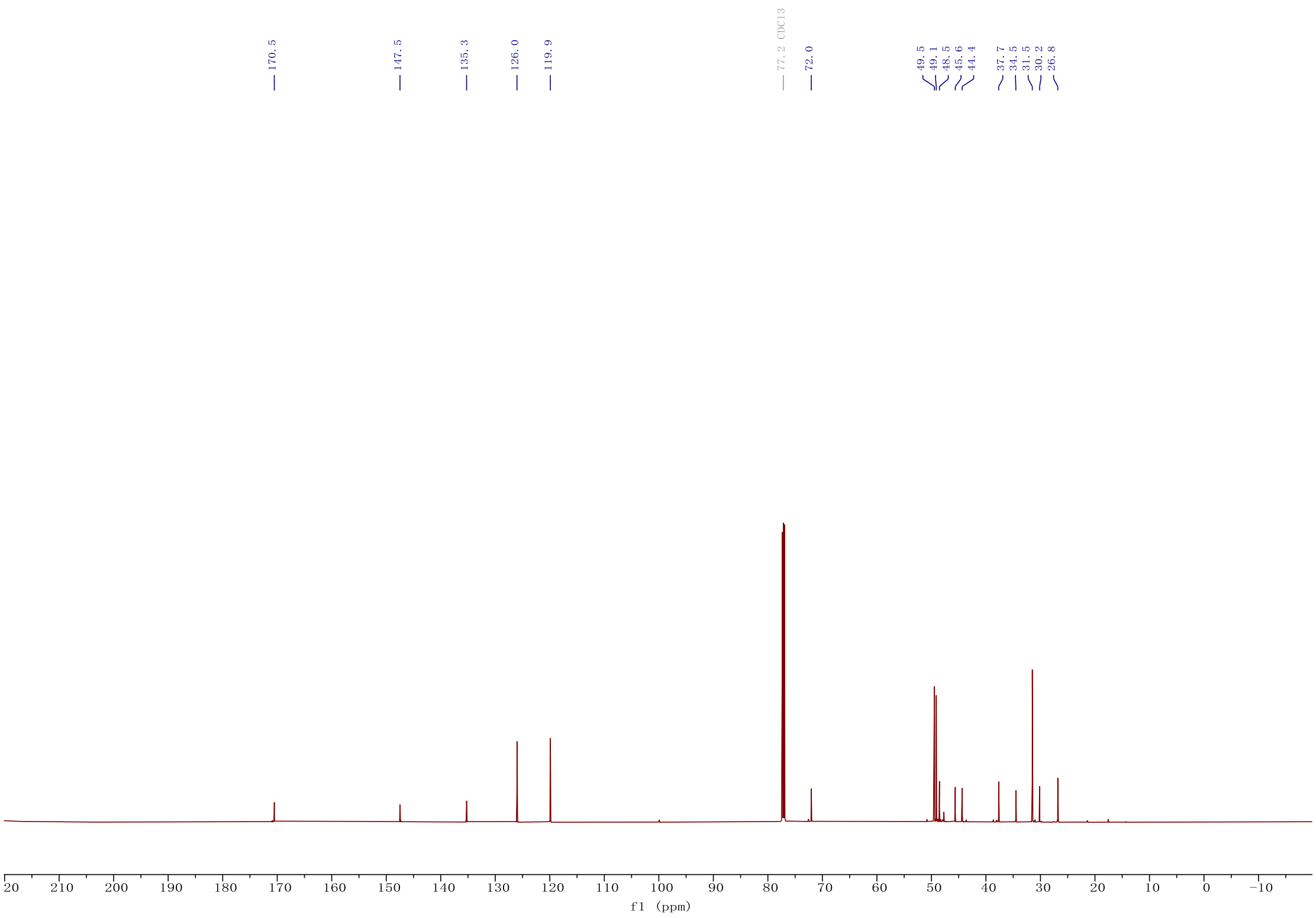

2-(bicyclo[1.1.1]pentan-1-yl)-3-(3-(dichloromethyl)bicyclo[1.1.1]pentan-1-yl)-*N*-(4-methoxyphenyl)propenamide (3e)

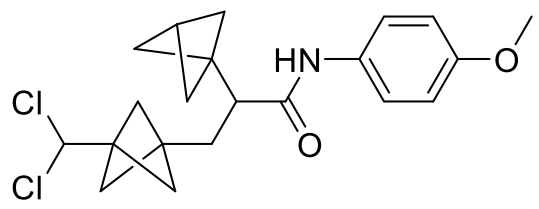

^{1}H NMR (600 MHz, $CDCl_3$)

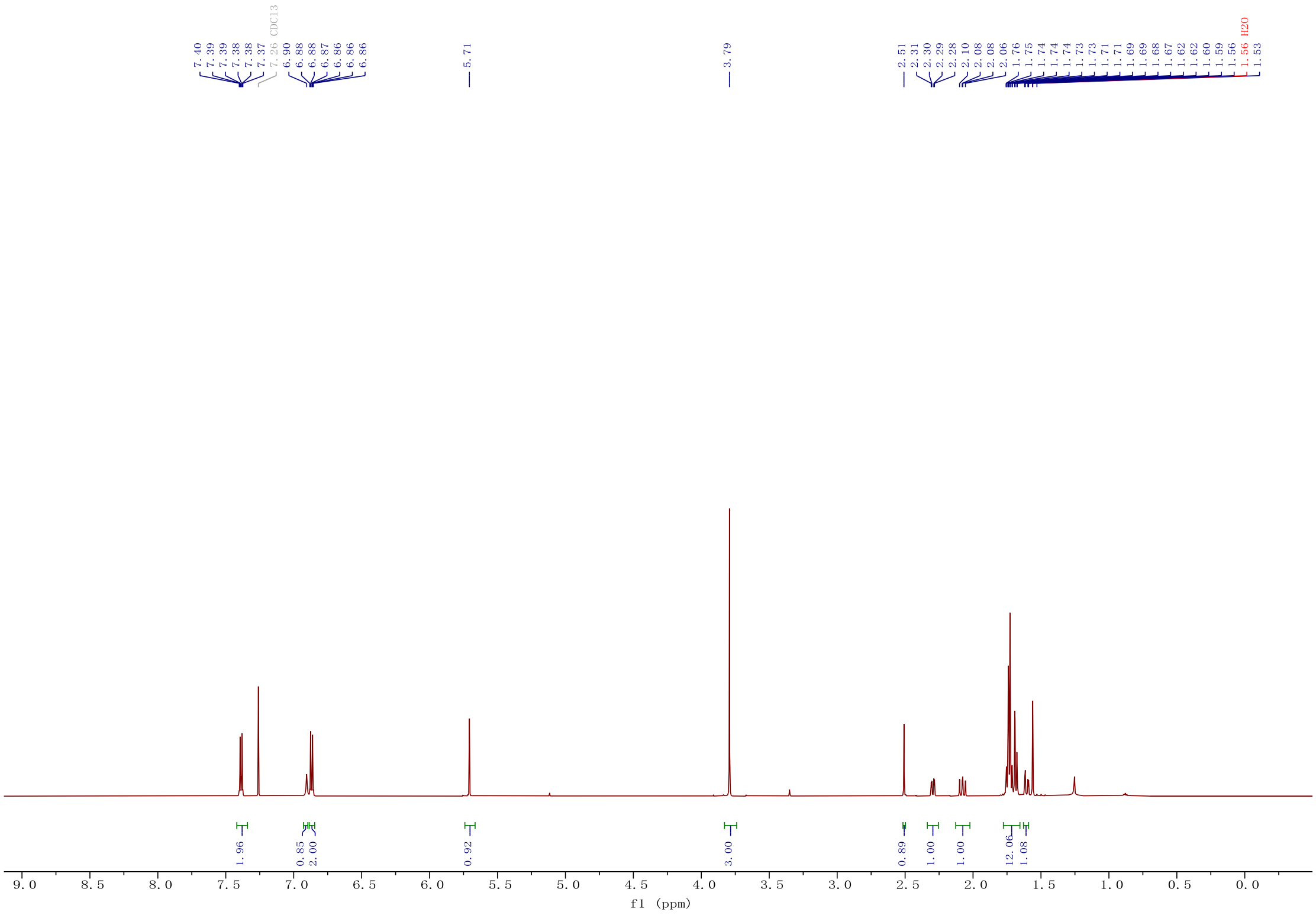

^{13}C NMR (151 MHz, $CDCl_3$)

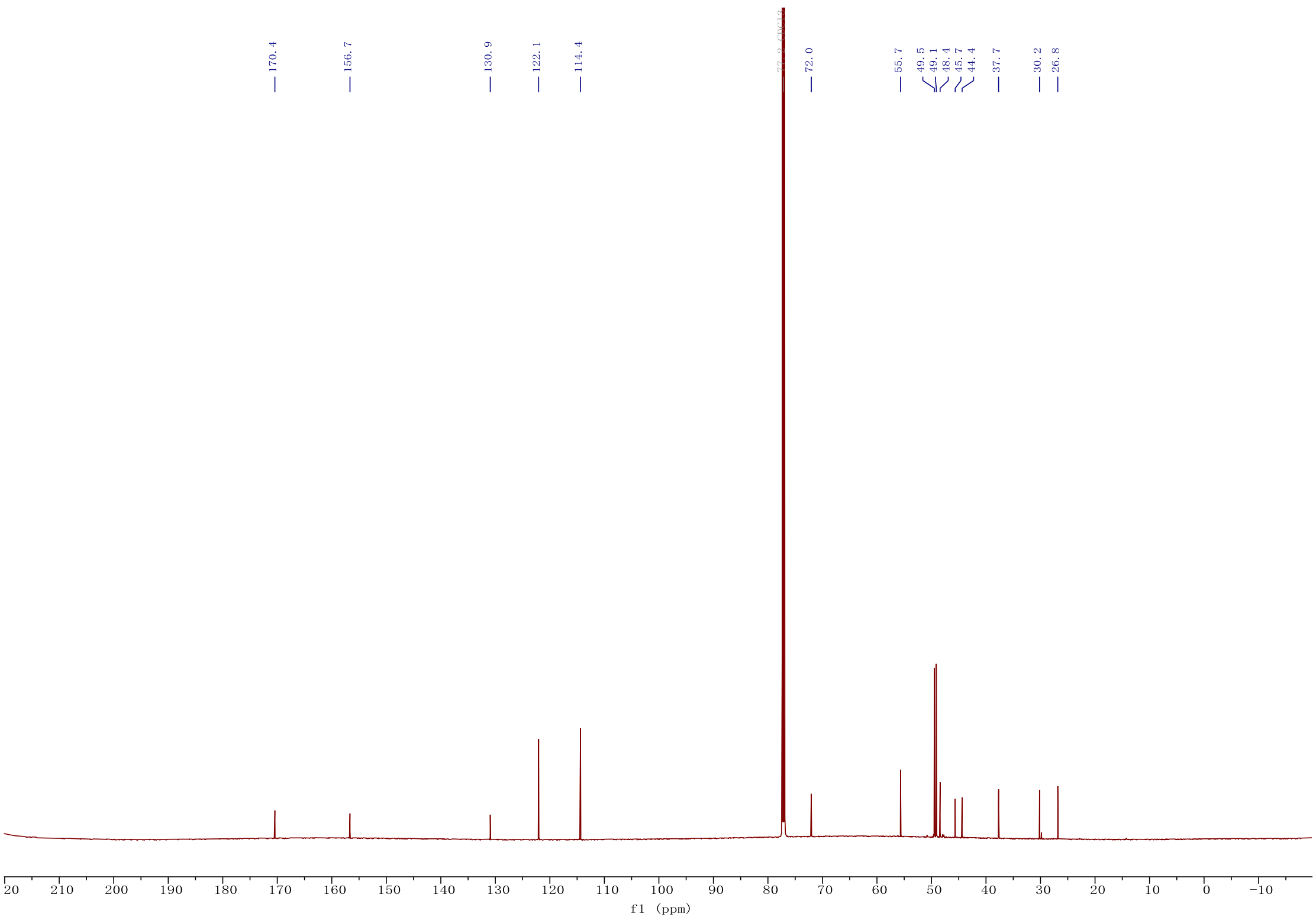

2-(bicyclo[1.1.1]pentan-1-yl)-*N*-(2-bromophenyl)-3-(3-(dichloromethyl)bicyclo[1.1.1]pentan-1-yl)propenamide (3f)

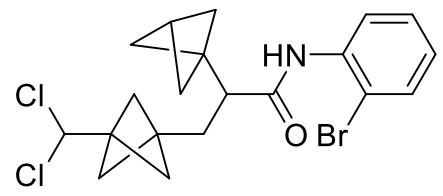

^{1}H NMR (600 MHz, $CDCl_3$)

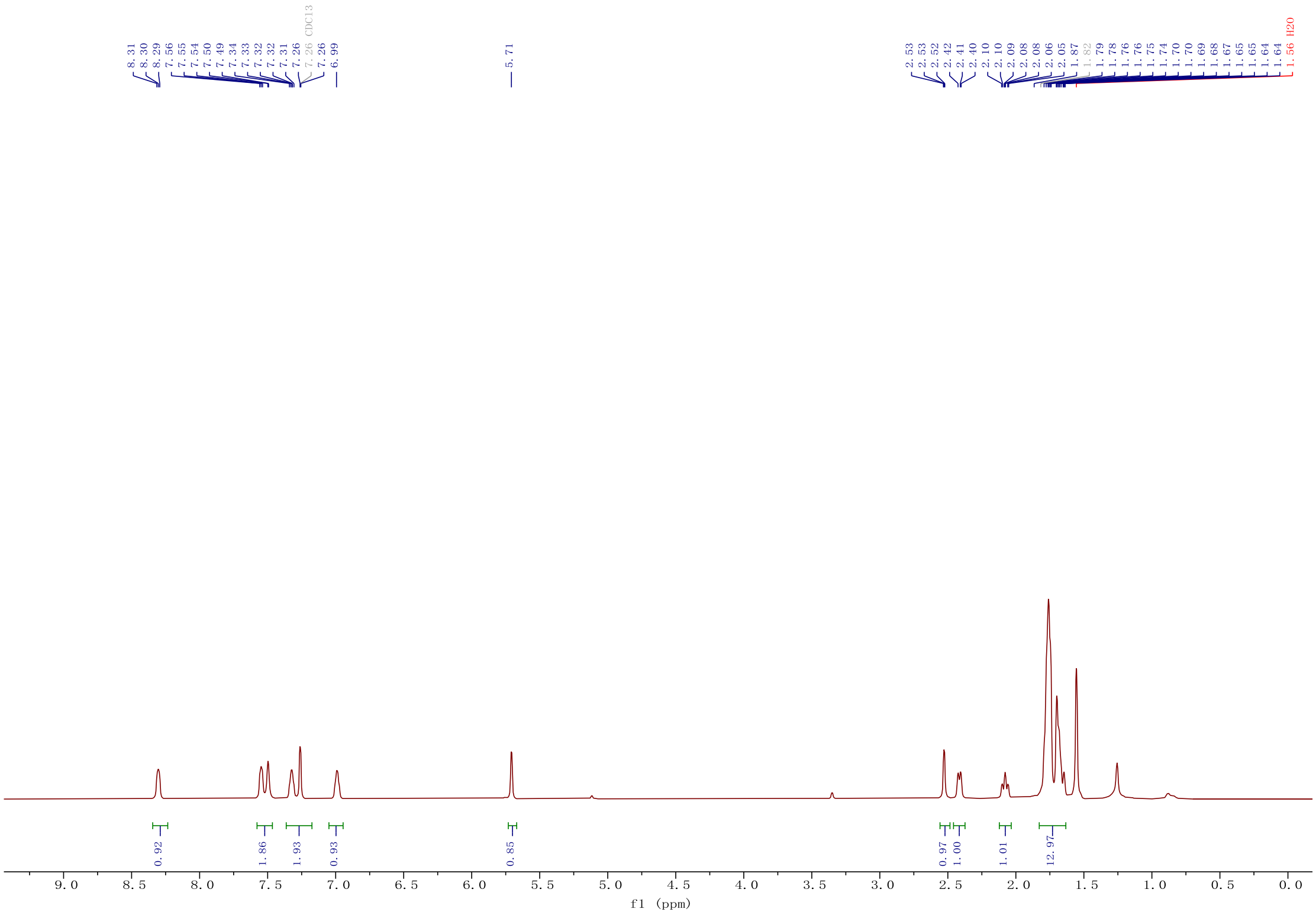

^{13}C NMR (151 MHz, $CDCl_3$)

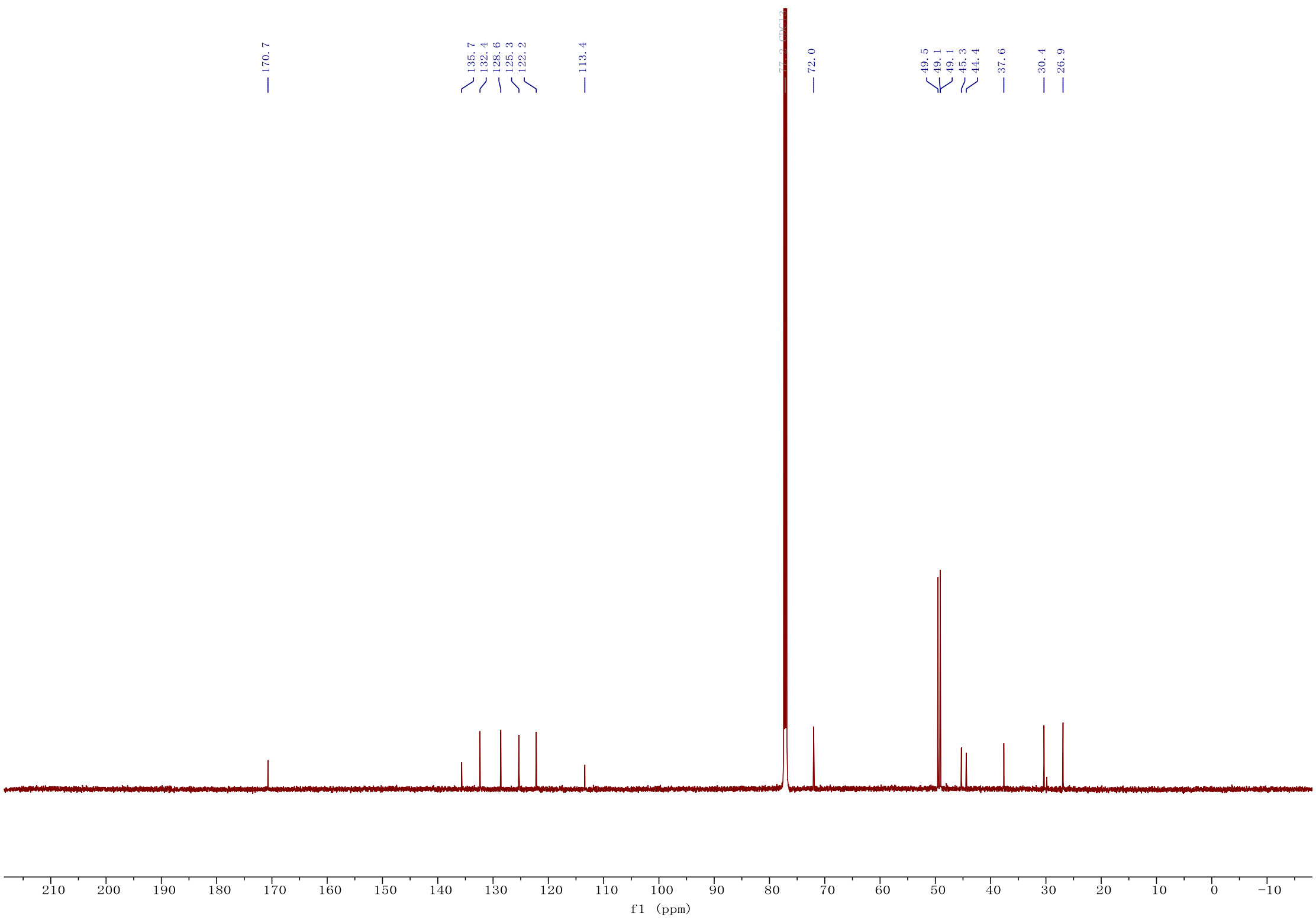

2-(bicyclo[1.1.1]pentan-1-yl)-3-(3-(dichloromethyl)bicyclo[1.1.1]pentan-1-yl)-*N*-(4-phenoxyphenyl)propenamide (3g)

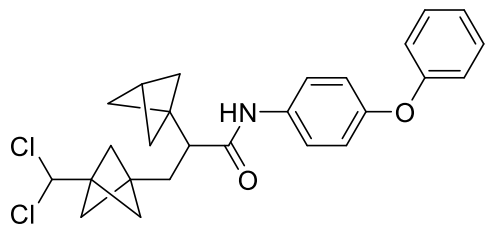

^{1}H NMR (600 MHz, $CDCl_3$)

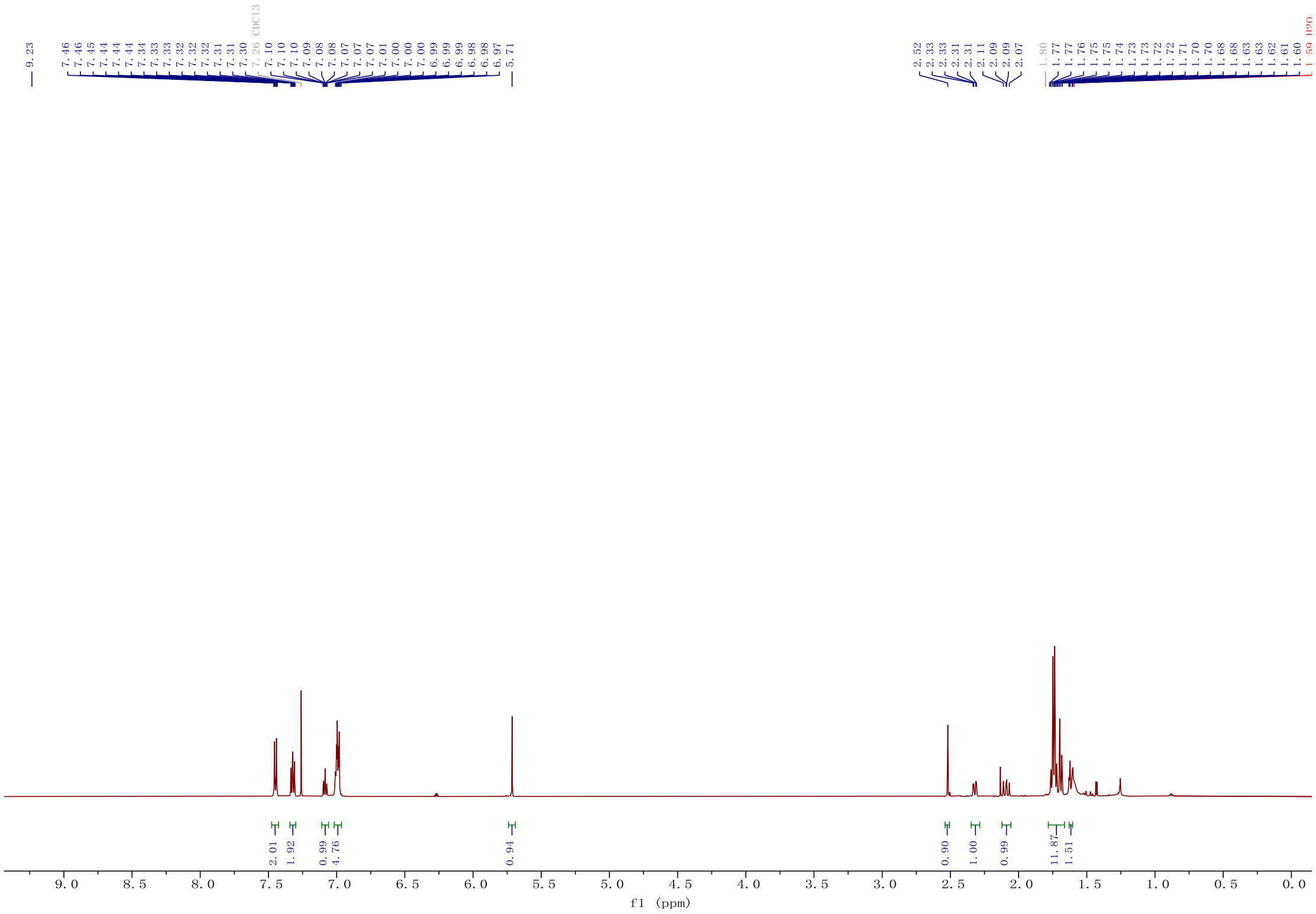

^{13}C NMR (151 MHz, $CDCl_3$)

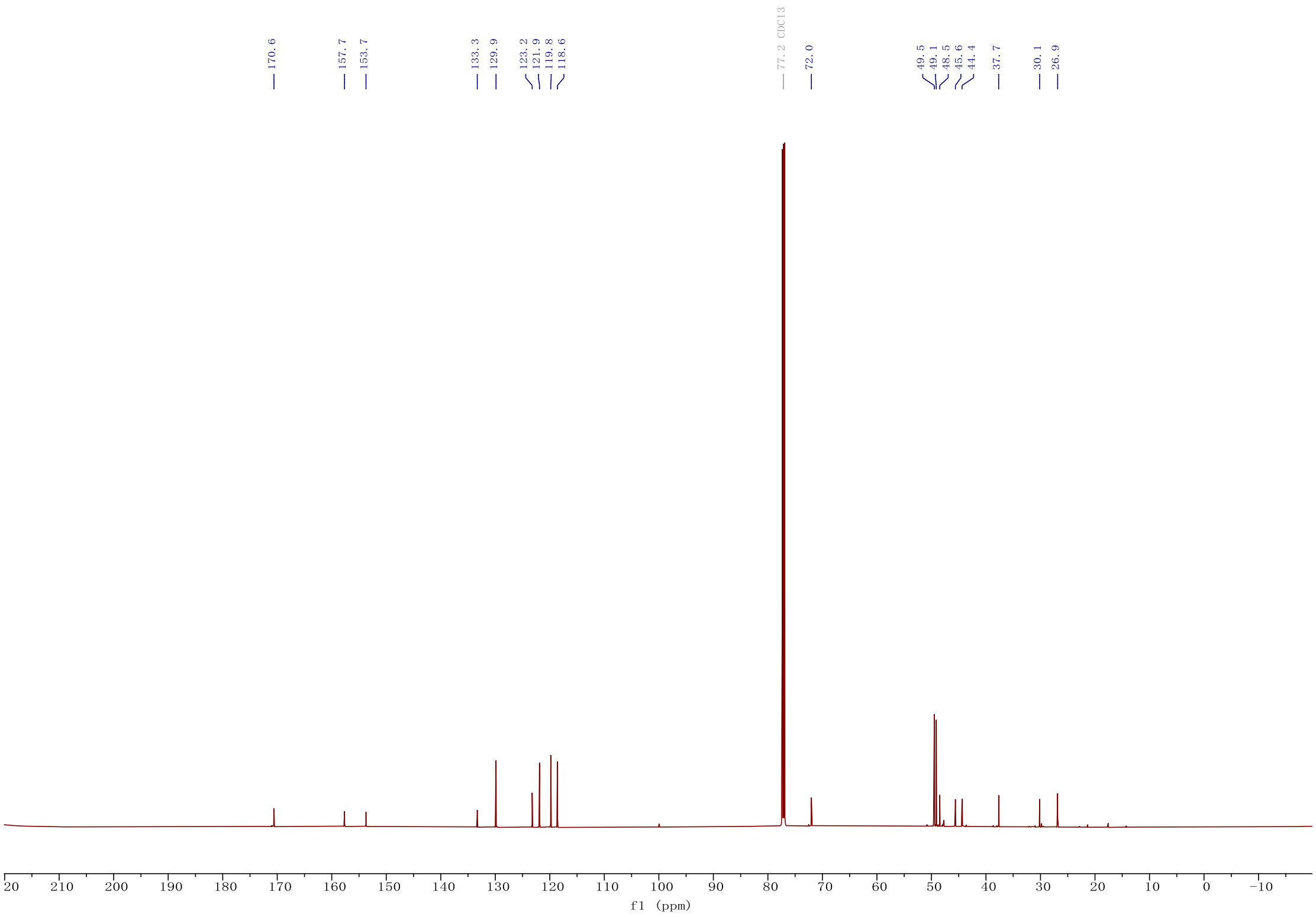

2-(bicyclo[1.1.1]pentan-1-yl)-3-(3-(dichloromethyl)bicyclo[1.1.1]pentan-1-yl)-*N*-(4-(4,4,5,5-tetramethyl-1,3,2-dioxaborolan-2-yl)phenyl)propenamide (3h)

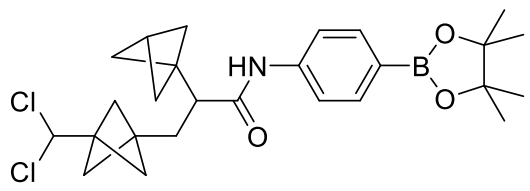

^{1}H NMR (600 MHz, CDCl$_3$)

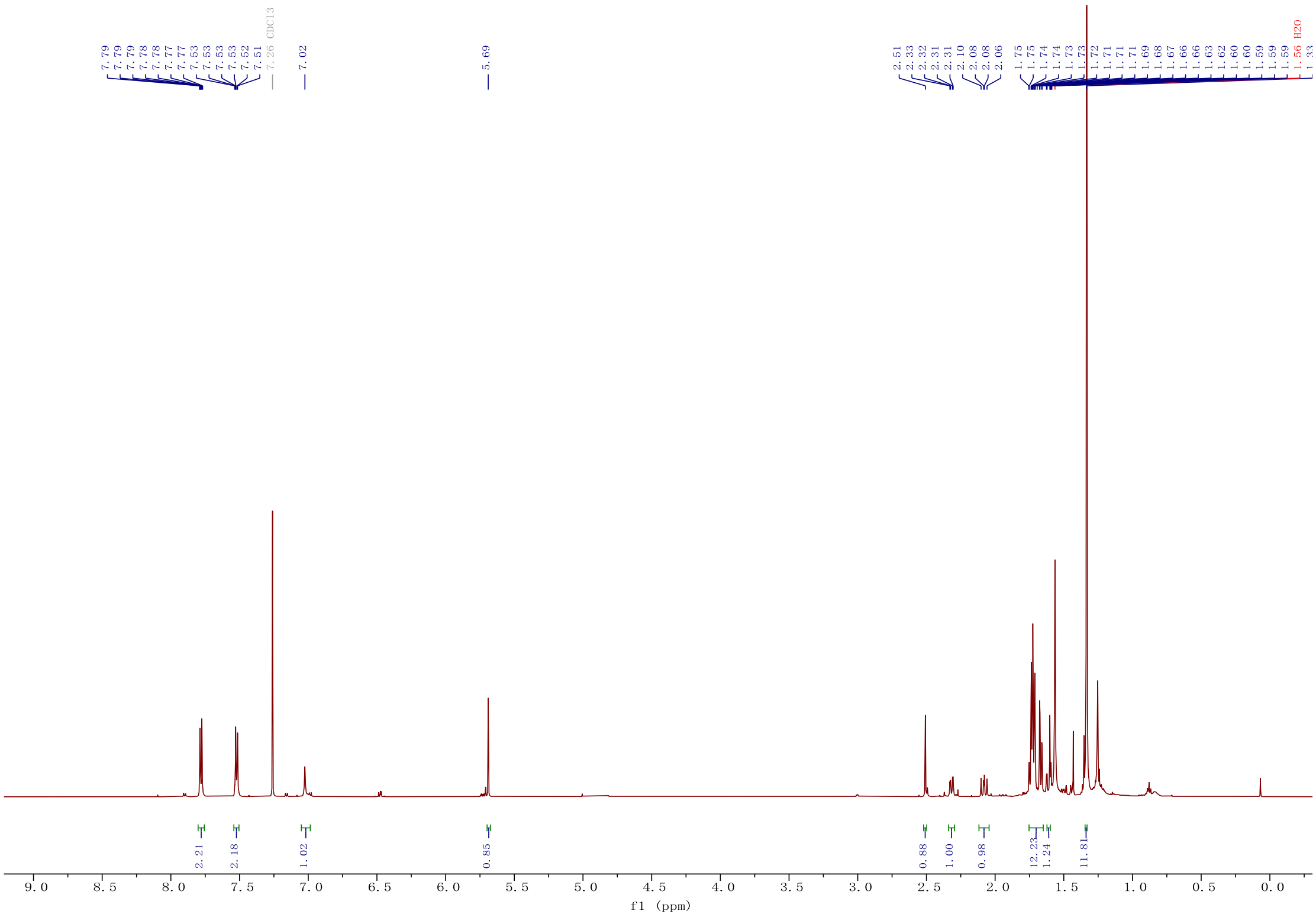

^{13}C NMR (151 MHz, CDCl$_3$)

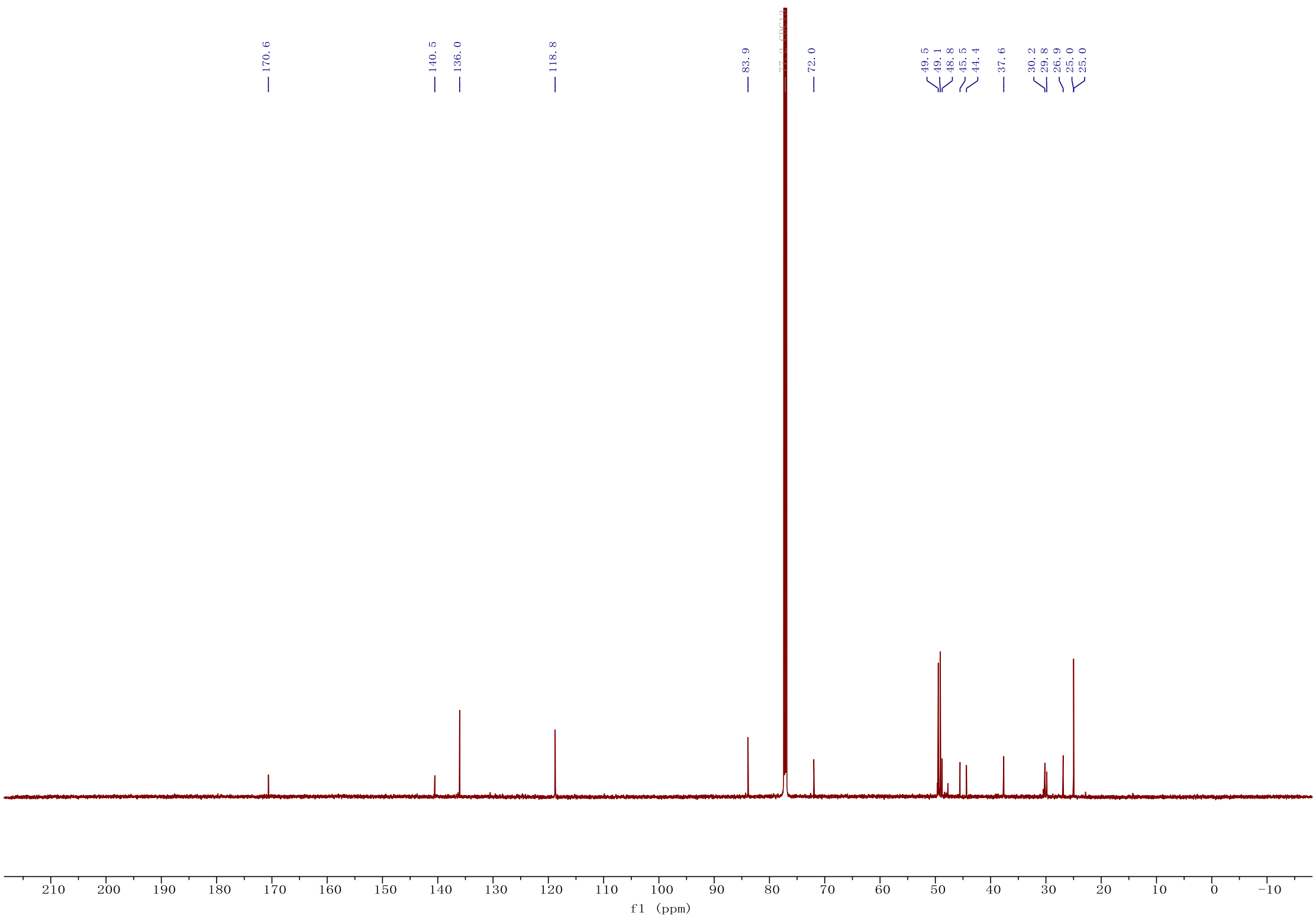

N-benzyl-2-(bicyclo[1.1.1]pentan-1-yl)-3-(3-(dichloromethyl)bicyclo[1.1.1]pentan-1-yl)propenamide (3i)

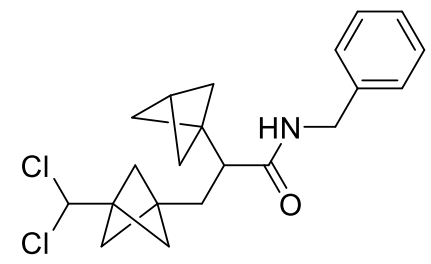

^{1}H NMR (600 MHz, $CDCl_3$)

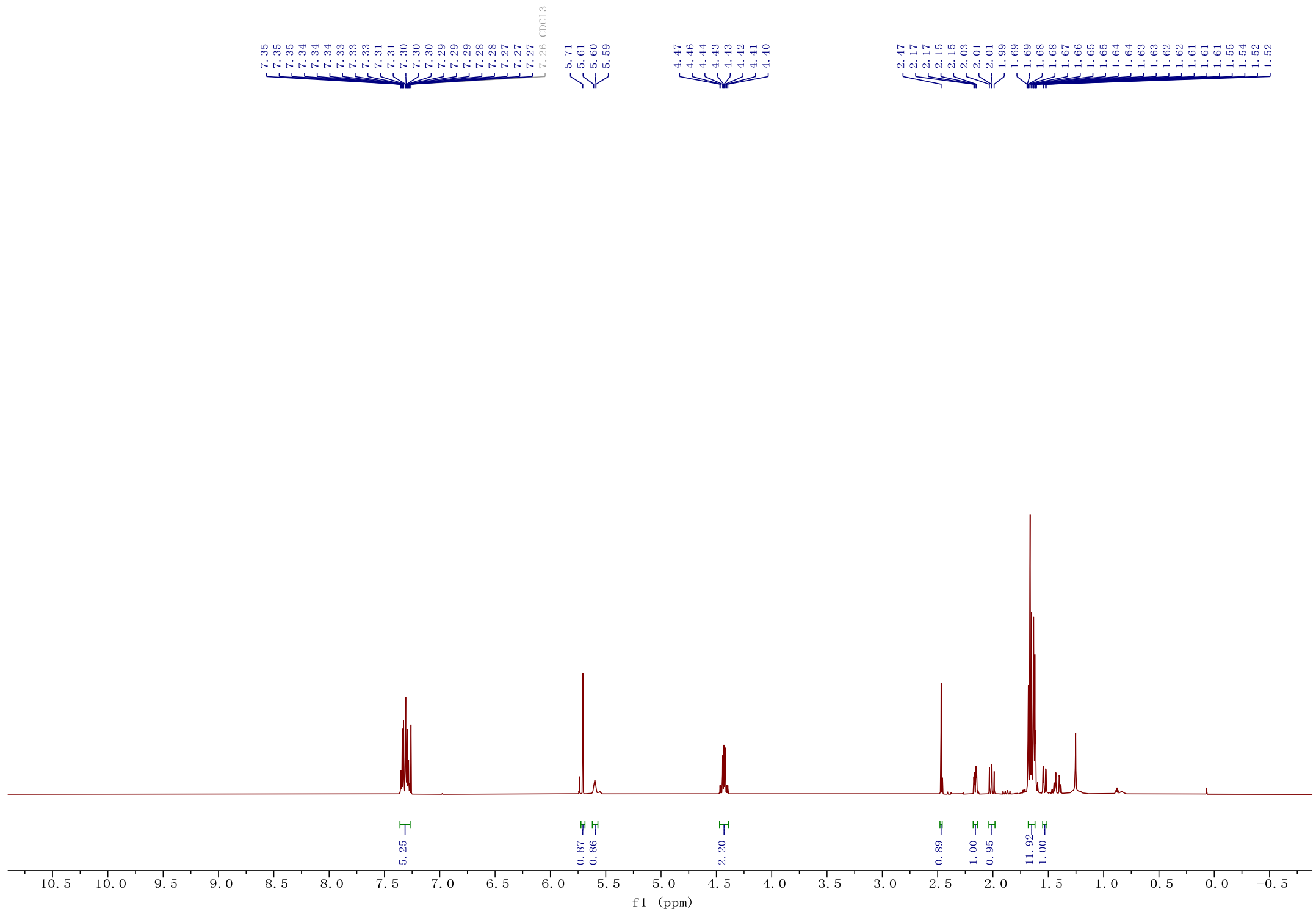

^{13}C NMR (151 MHz, $CDCl_3$)

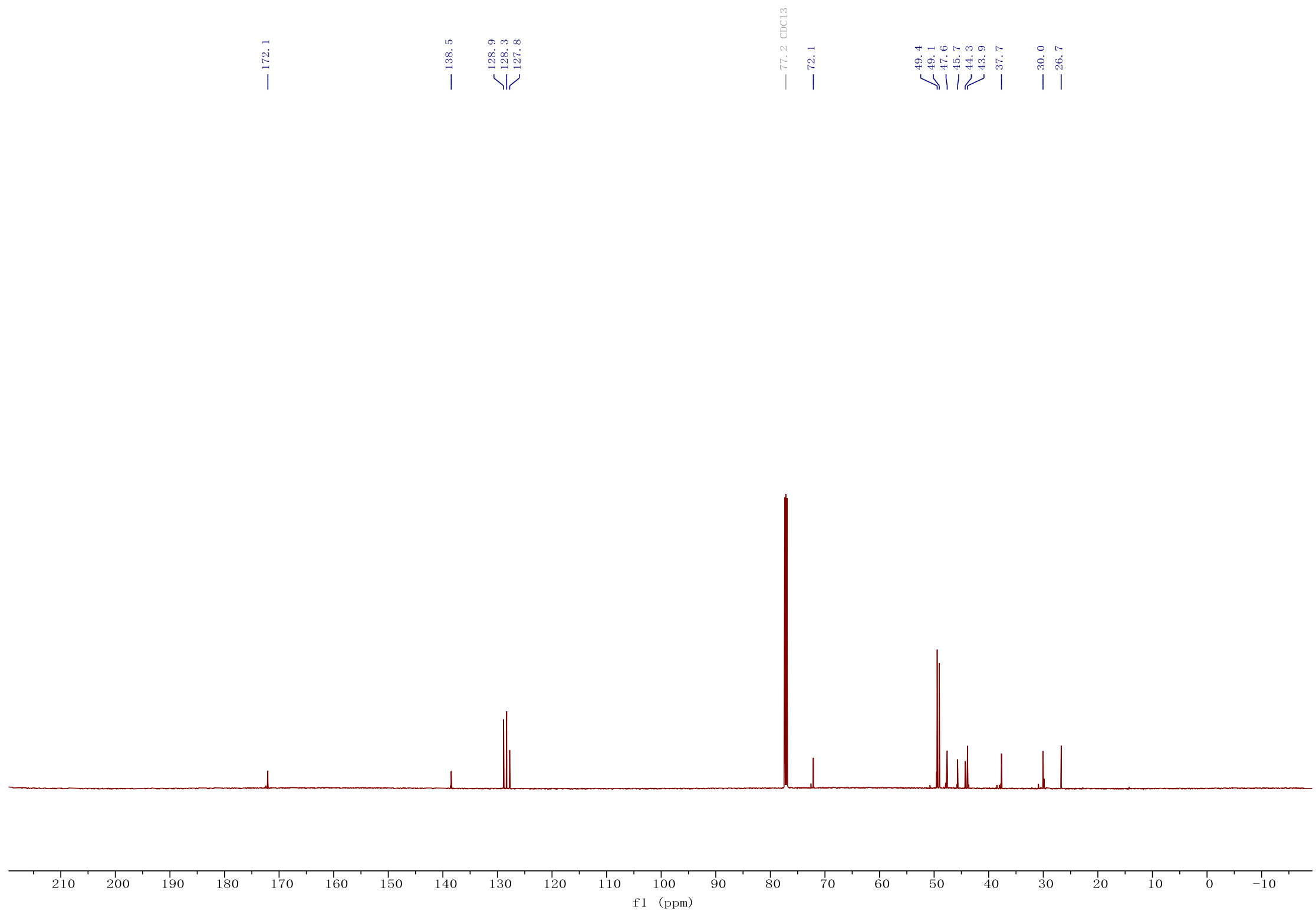

2-(bicyclo[1.1.1]pentan-1-yl)-3-(3-(dichloromethyl)bicyclo[1.1.1]pentan-1-yl)-*N*-(2-methylbenzyl)propenamide (3j)

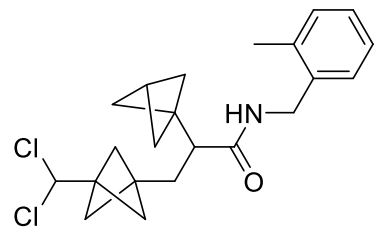

^{1}H NMR (600 MHz, CDCl$_3$)

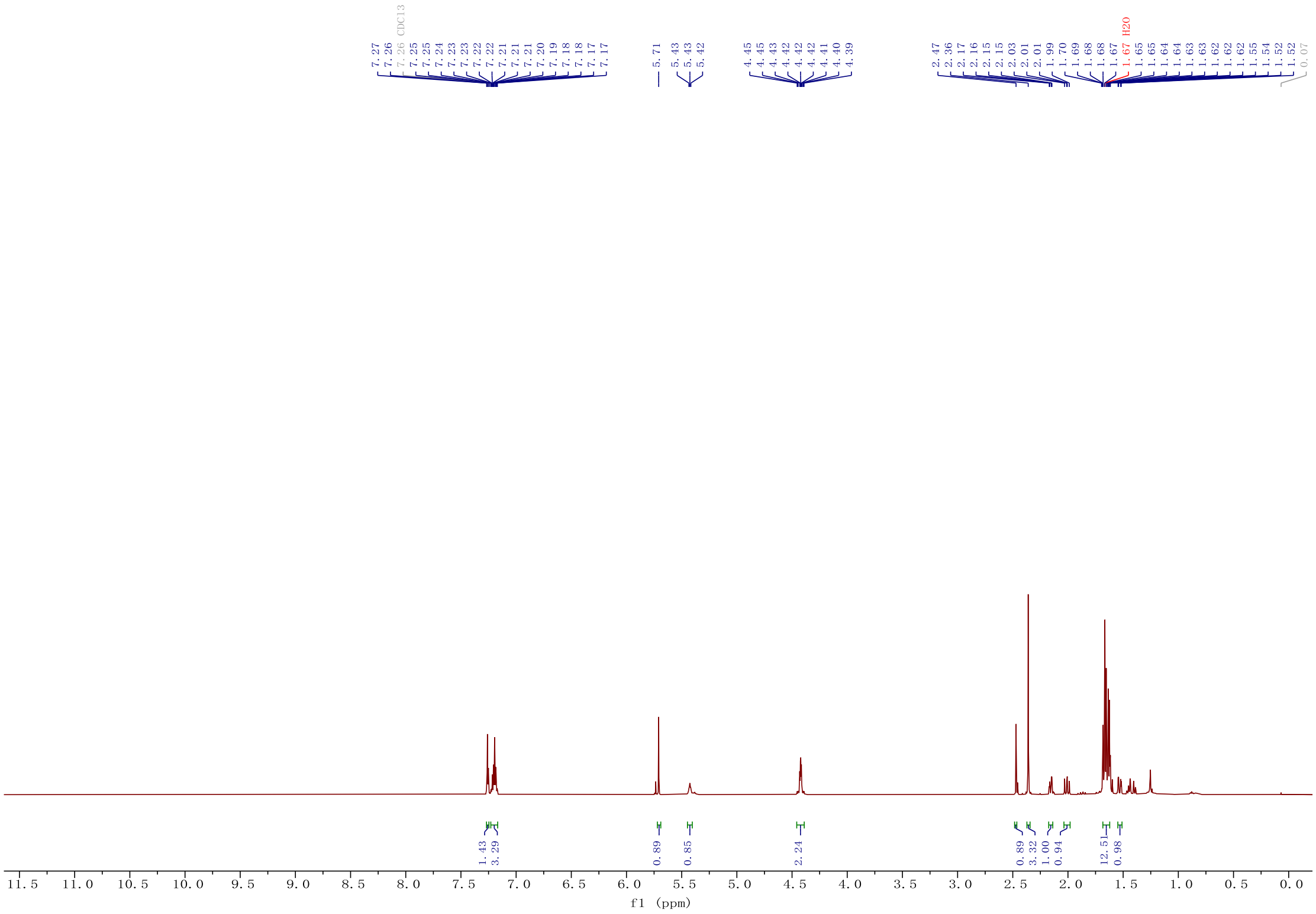

^{13}C NMR (151 MHz, CDCl$_3$)

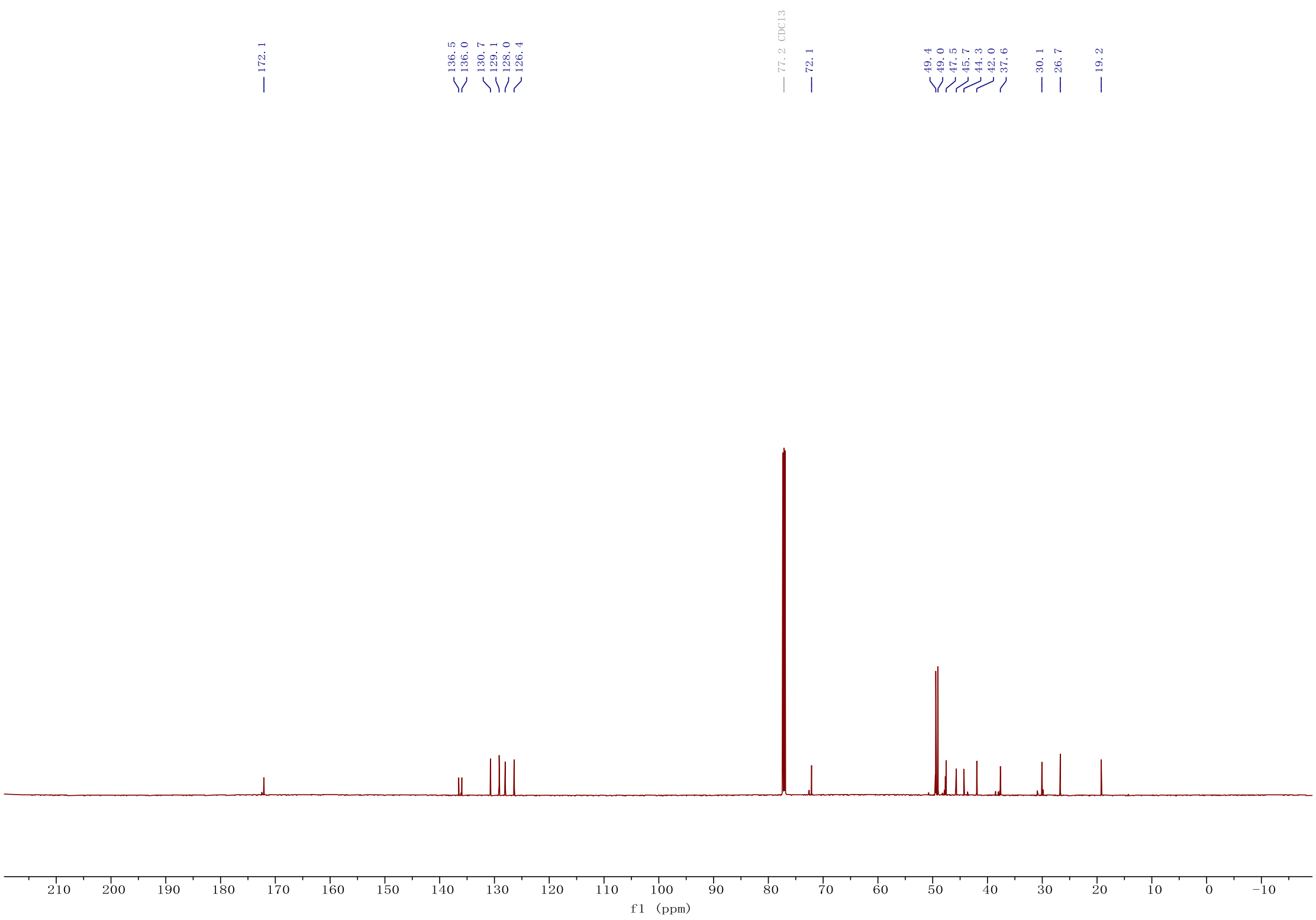

2-(bicyclo[1.1.1]pentan-1-yl)-*N*-butyl-3-(3-(dichloromethyl)bicyclo[1.1.1]pentan-1-yl)propenamide (3k)

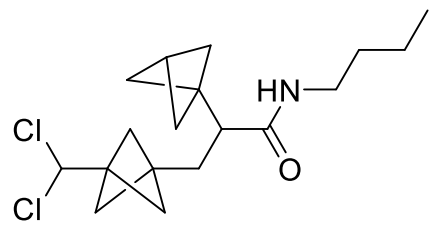

^{1}H NMR (600 MHz, $CDCl_3$)

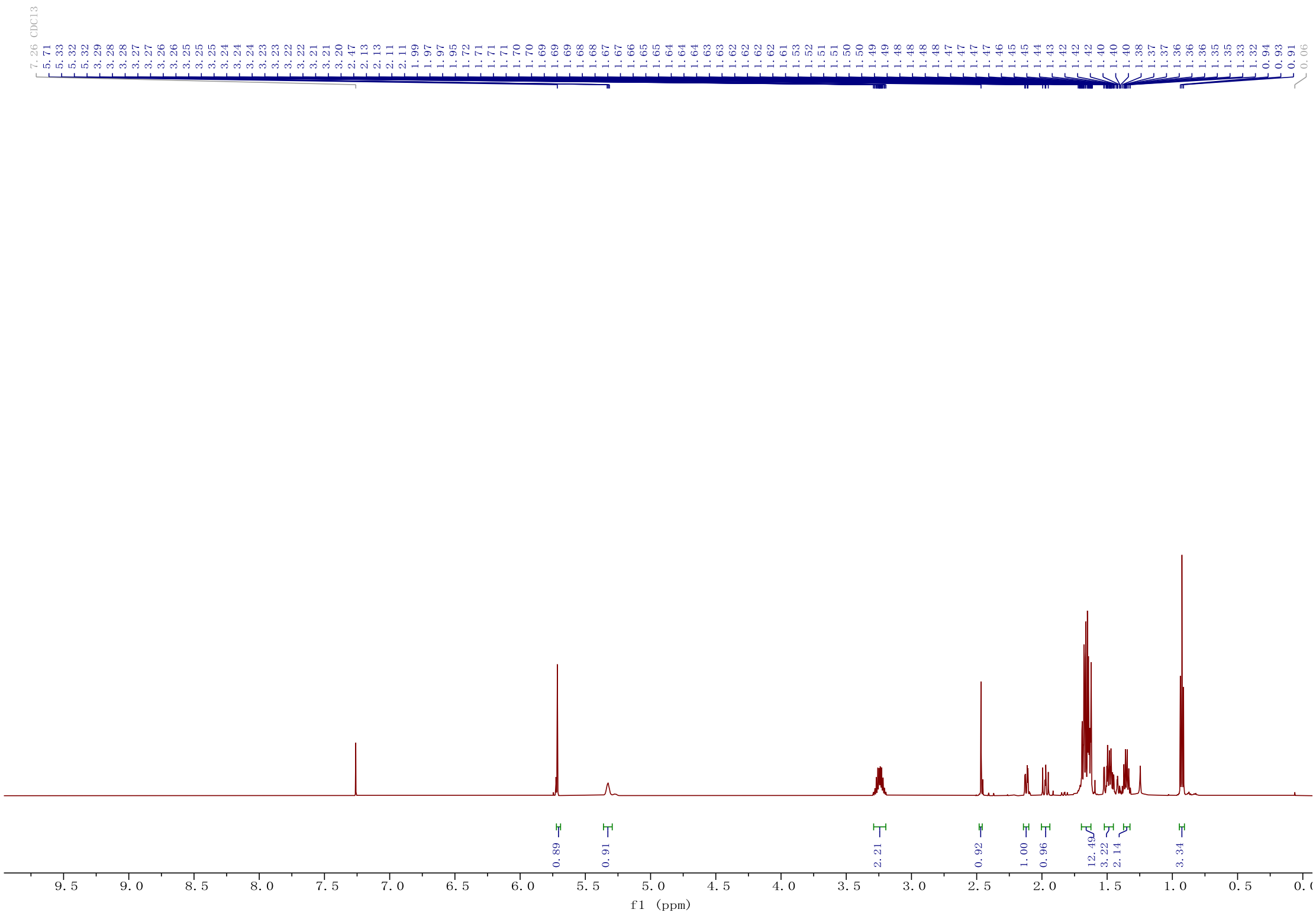

^{13}C NMR (151 MHz, $CDCl_3$)

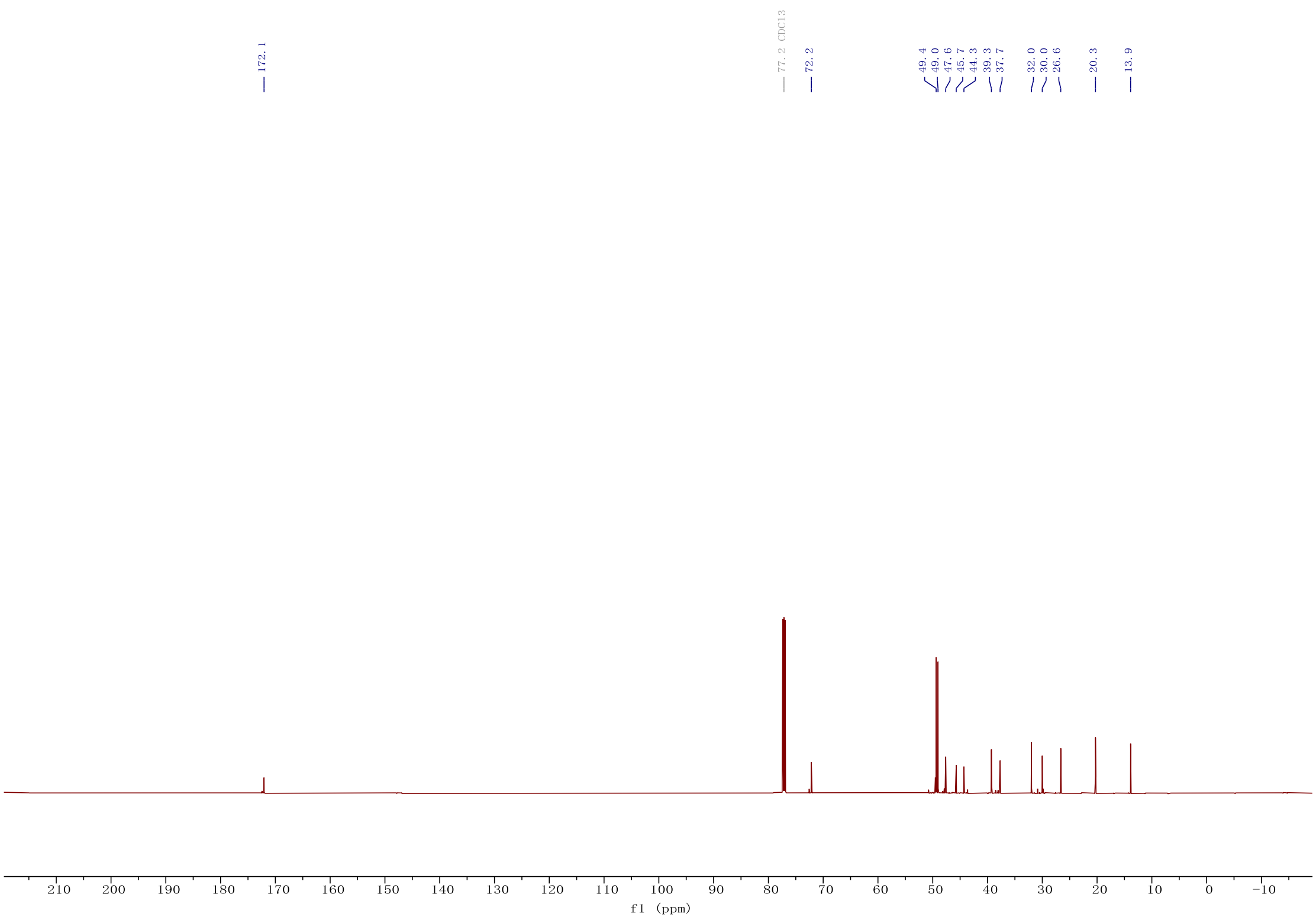

2-(bicyclo[1.1.1]pentan-1-yl)-*N*-cyclopropyl-3-(3-(dichloromethyl)bicyclo[1.1.1]pentan-1-yl)propenamide (3l)

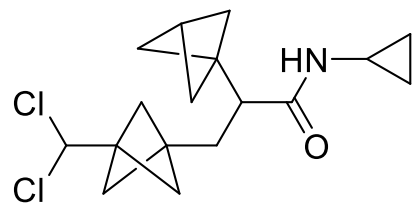

^{1}H NMR (600 MHz, $CDCl_3$)

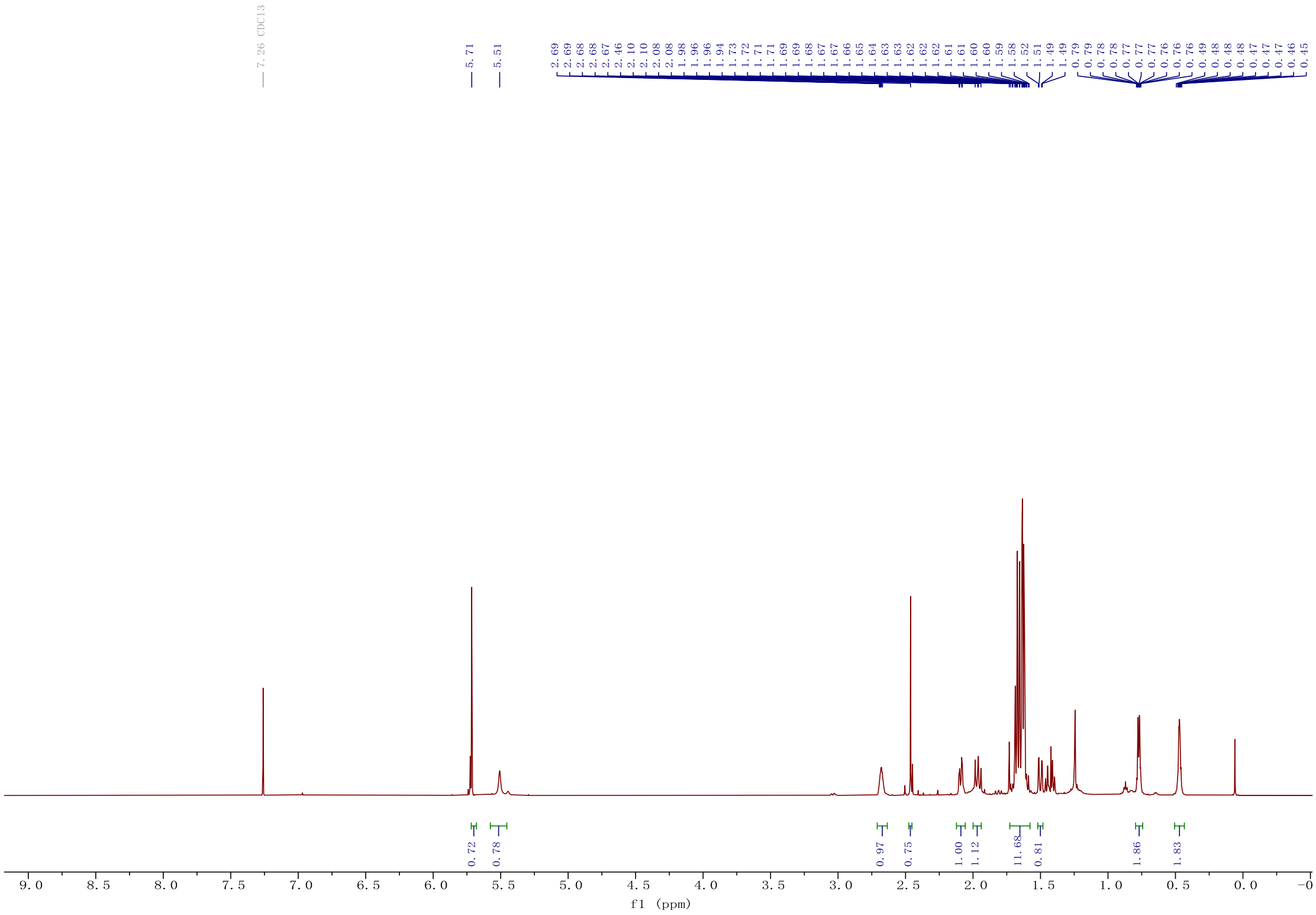

^{13}C NMR (151 MHz, $CDCl_3$)

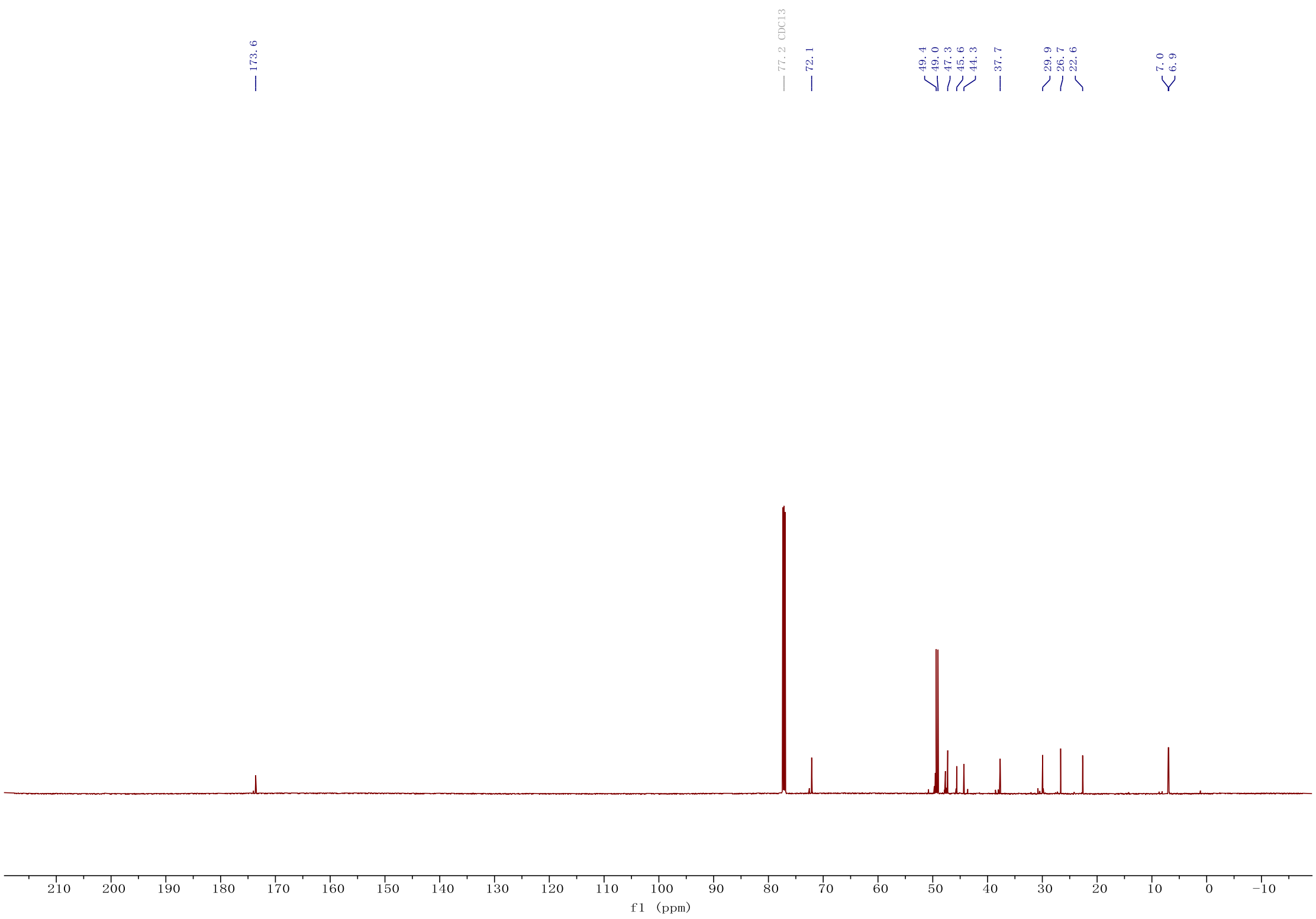

2-(bicyclo[1.1.1]pentan-1-yl)-*N*-cyclohexyl-3-(3-(dichloromethyl)bicyclo[1.1.1]pentan-1-yl)propenamide (3m)

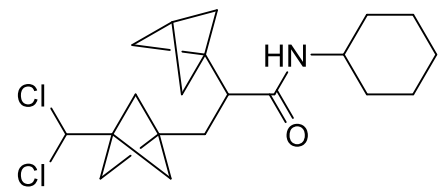

^{1}H NMR (600 MHz, $CDCl_3$)

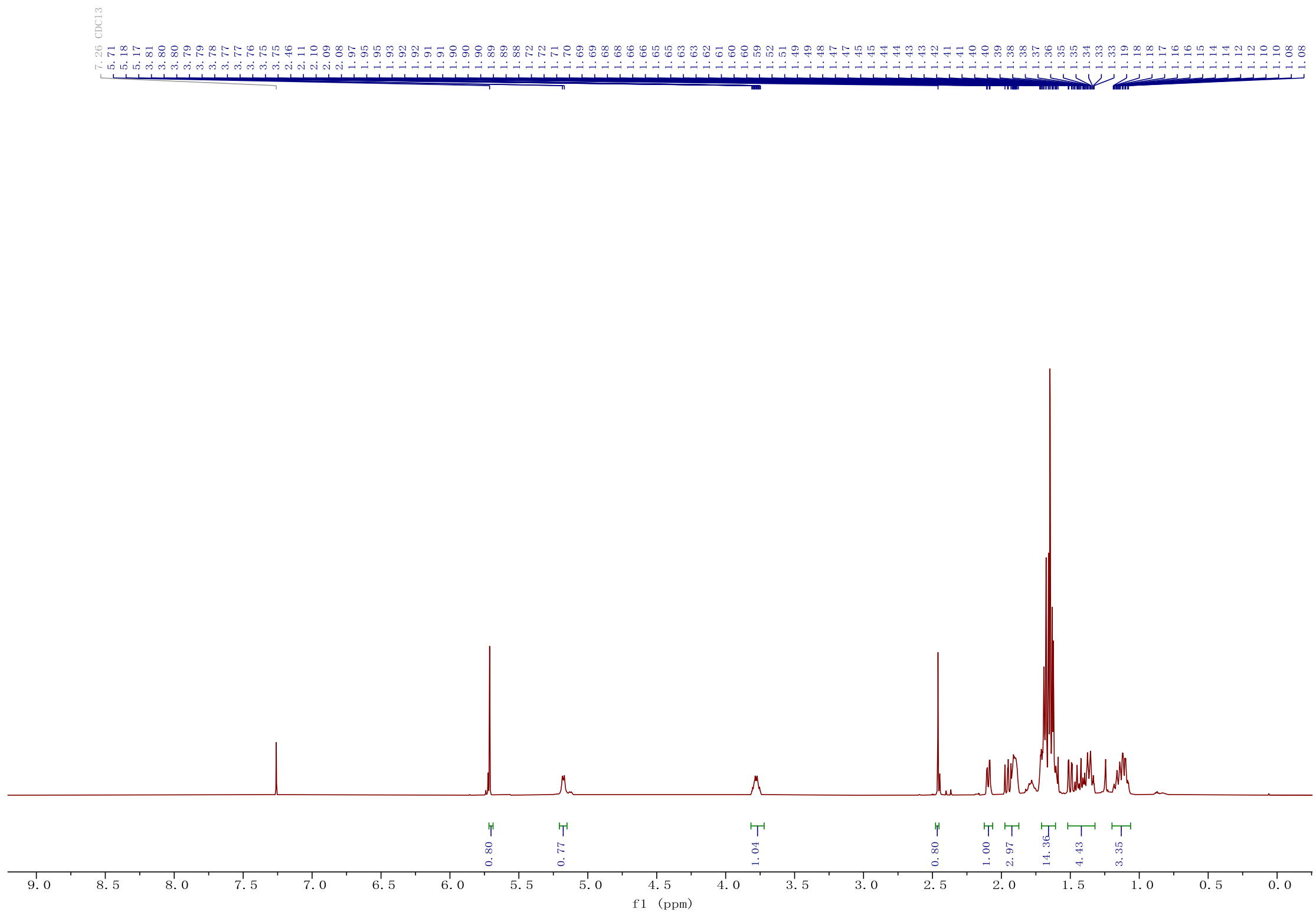

^{13}C NMR (151 MHz, $CDCl_3$)

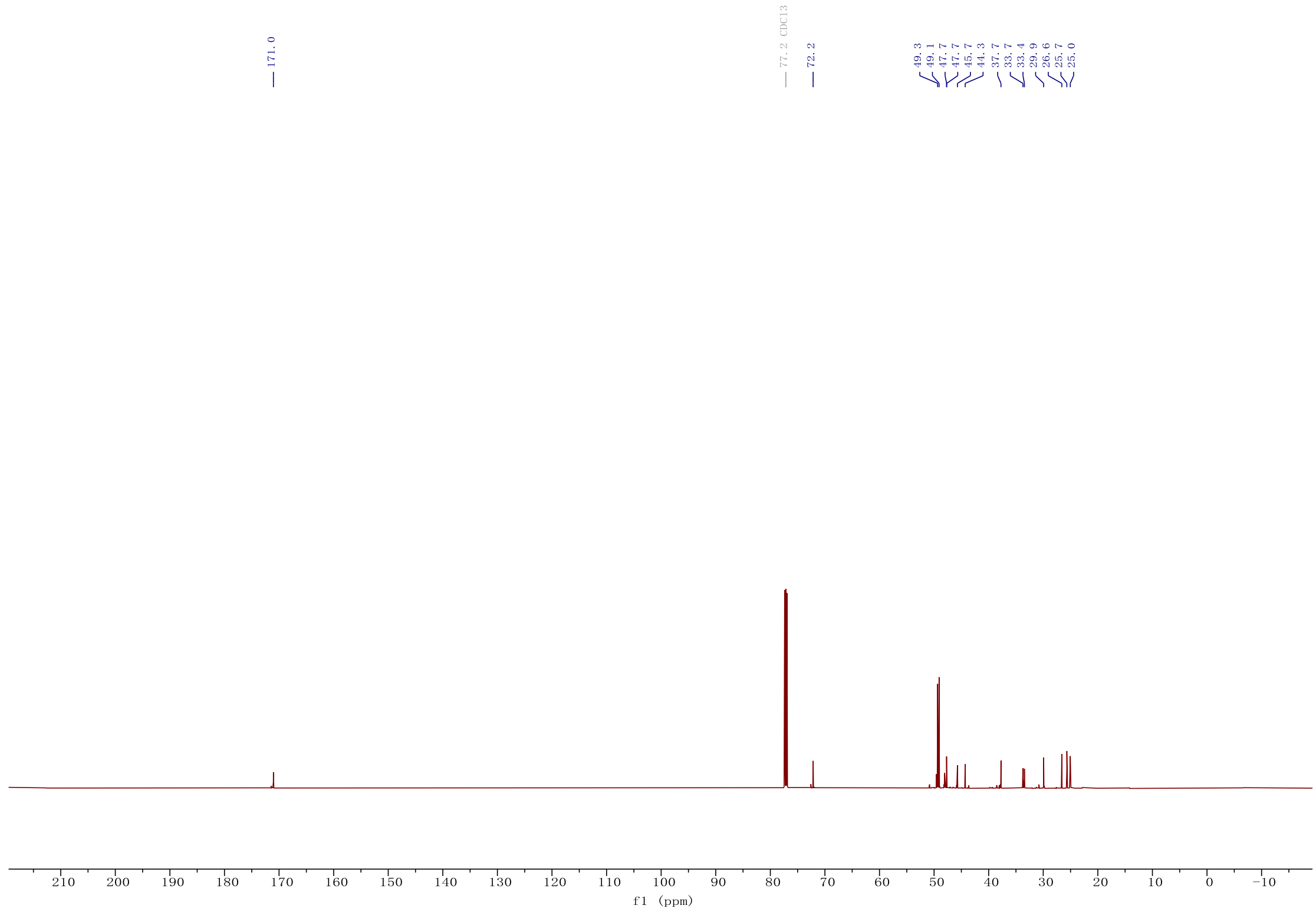

2-(bicyclo[1.1.1]pentan-1-yl)-3-(3-(dichloromethyl)bicyclo[1.1.1]pentan-1-yl)-*N*-(2-methyl-4-oxopentan-2-yl)propenamide (3n)

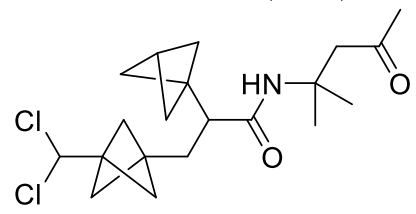

^{1}H NMR (600 MHz, CDCl₃)

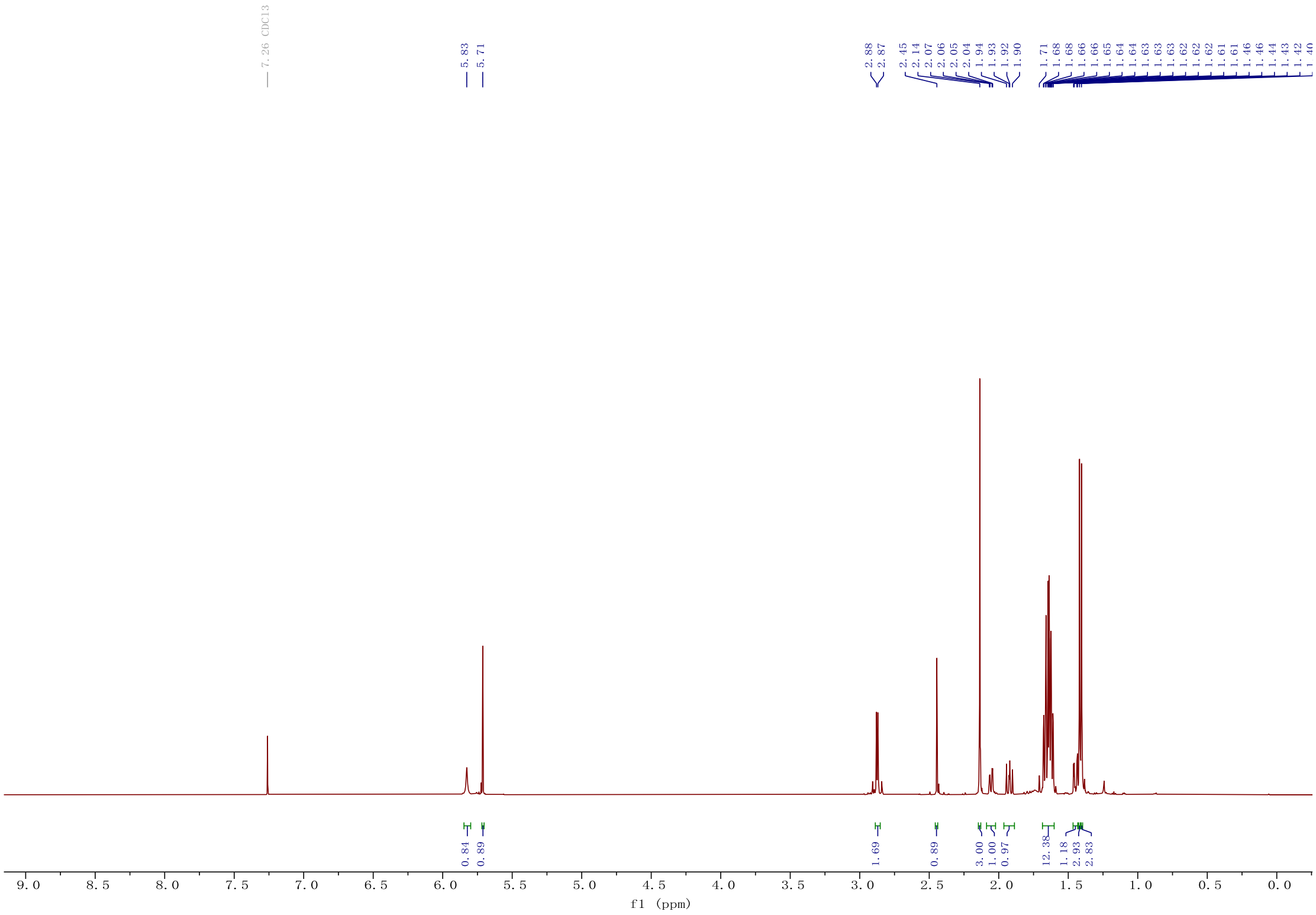

^{13}C NMR (151 MHz, CDCl₃)

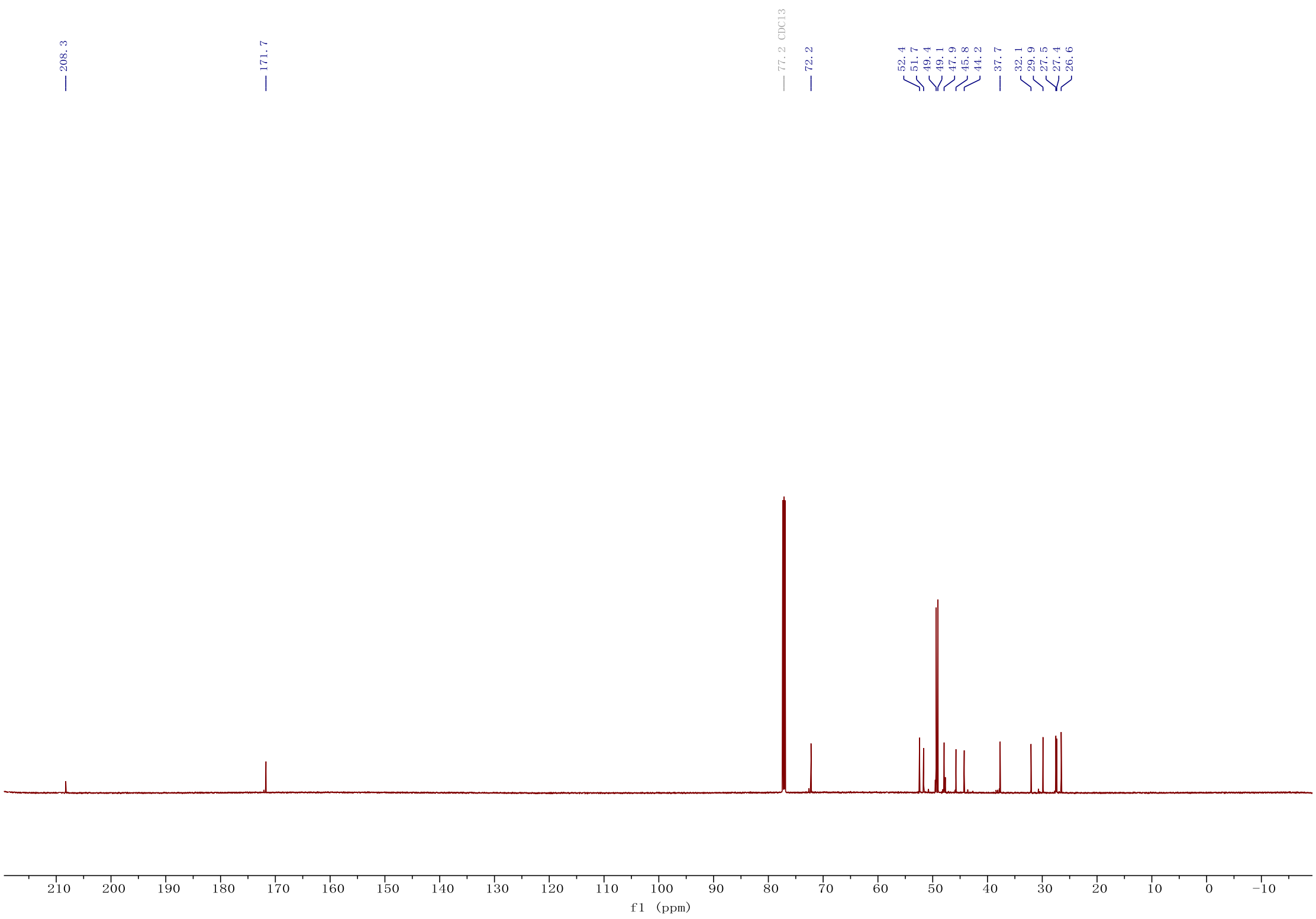

2-(bicyclo[1.1.1]pentan-1-yl)-3-(3-(dichloromethyl)bicyclo[1.1.1]pentan-1-yl)-*N*,*N*-dimethylpropanamide (3o)

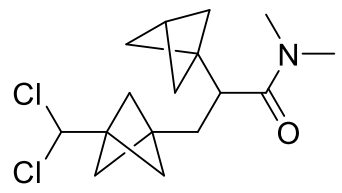

^{1}H NMR (600 MHz, CDCl3)

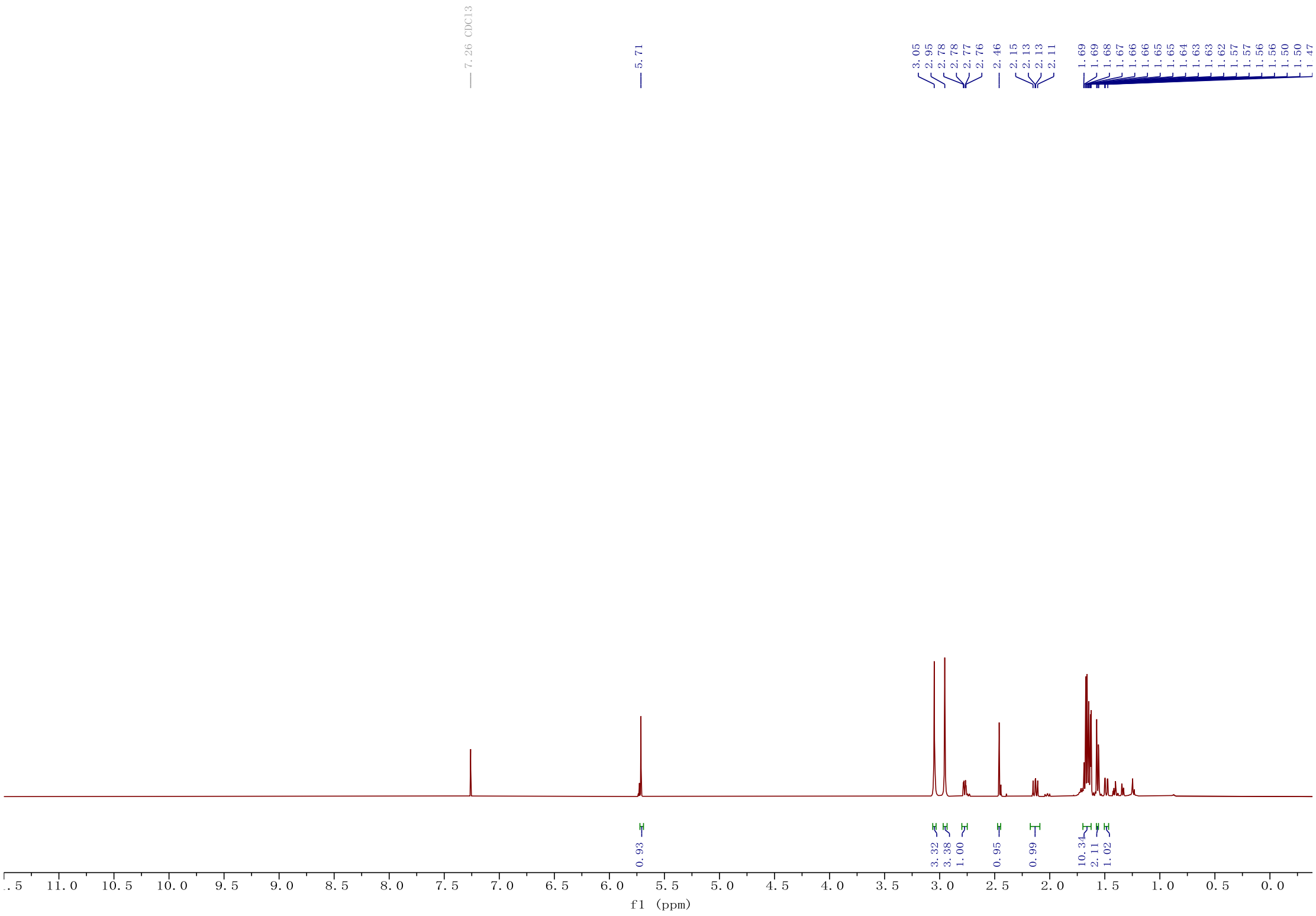

^{13}C NMR (151 MHz, CDCl3)

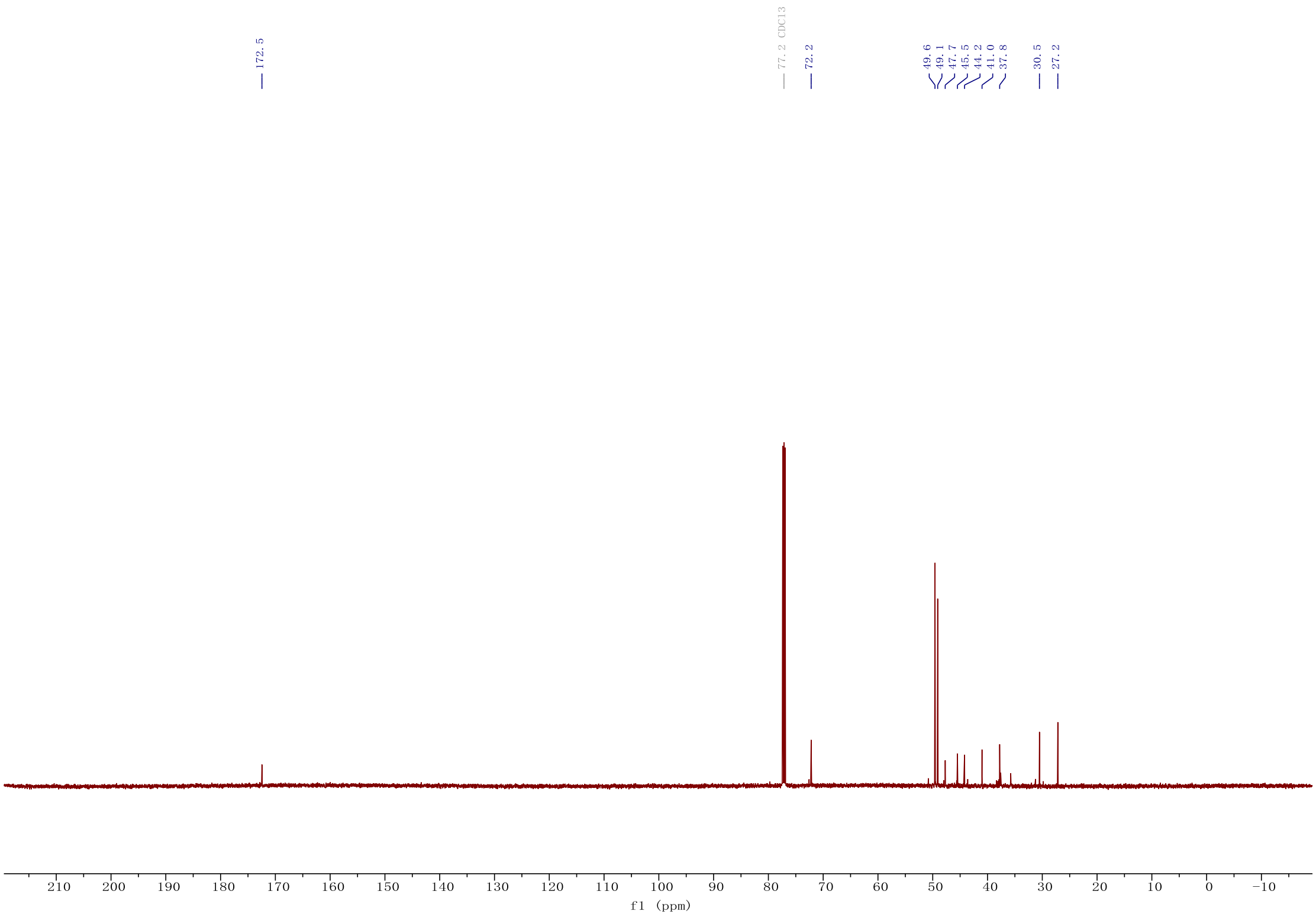

2-(bicyclo[1.1.1]pentan-1-yl)-3-(3-(dichloromethyl)bicyclo[1.1.1]pentan-1-yl)-1-morpholinopropan-1-one (3p)

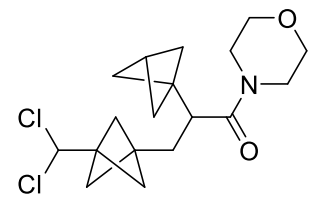

^{1}H NMR (600 MHz, $CDCl_3$)

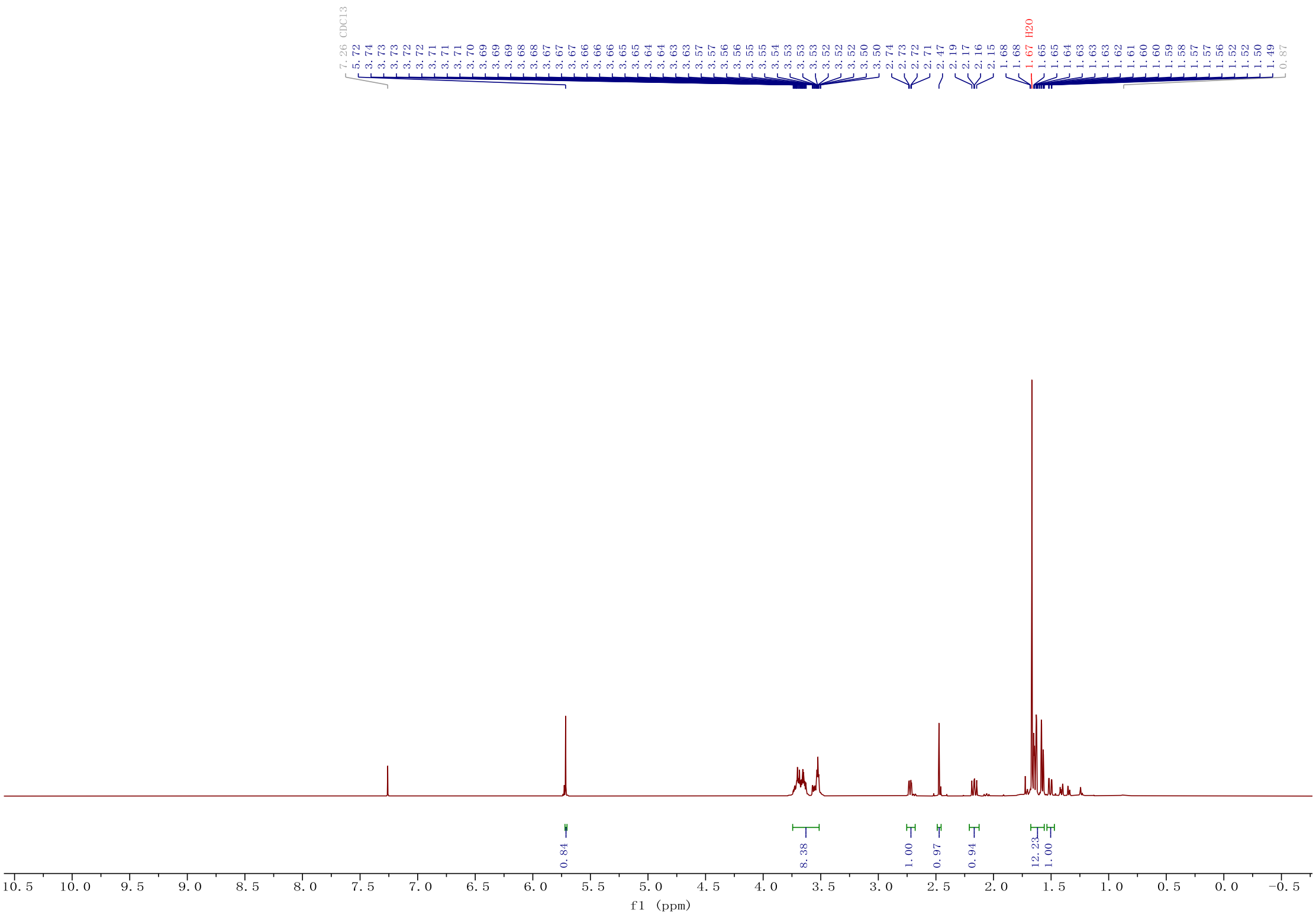

^{13}C NMR (151 MHz, $CDCl_3$)

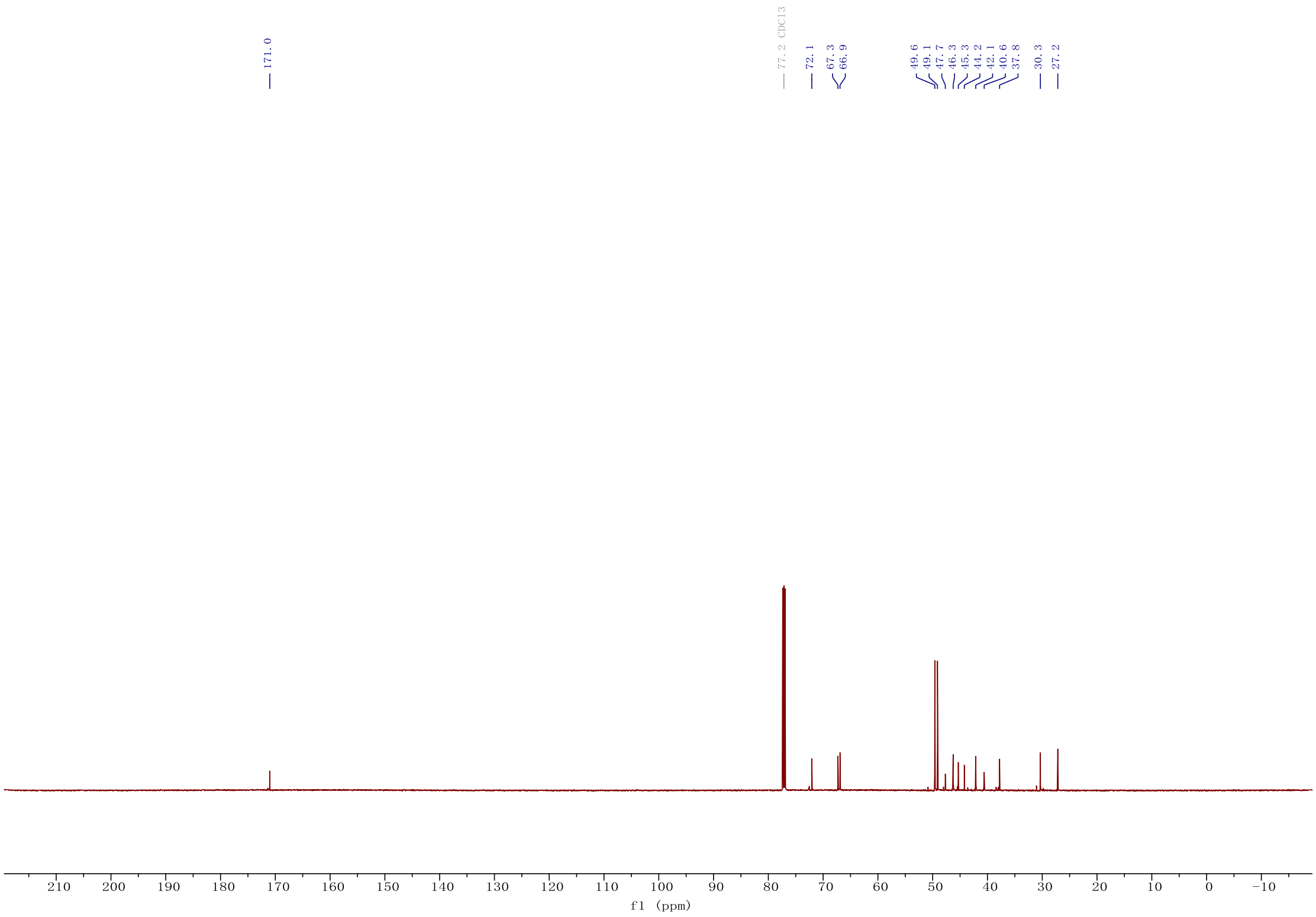

2-(bicyclo[1.1.1]pentan-1-yl)-3-(3-(dichloromethyl)bicyclo[1.1.1]pentan-1-yl)-2-methyl-*N*-phenylpropanamide (3q)

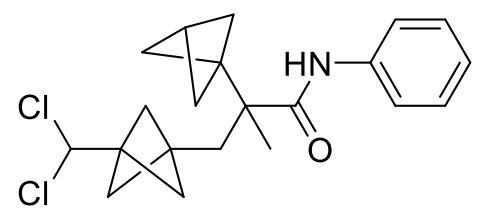

^{1}H NMR (600 MHz, $CDCl_3$)

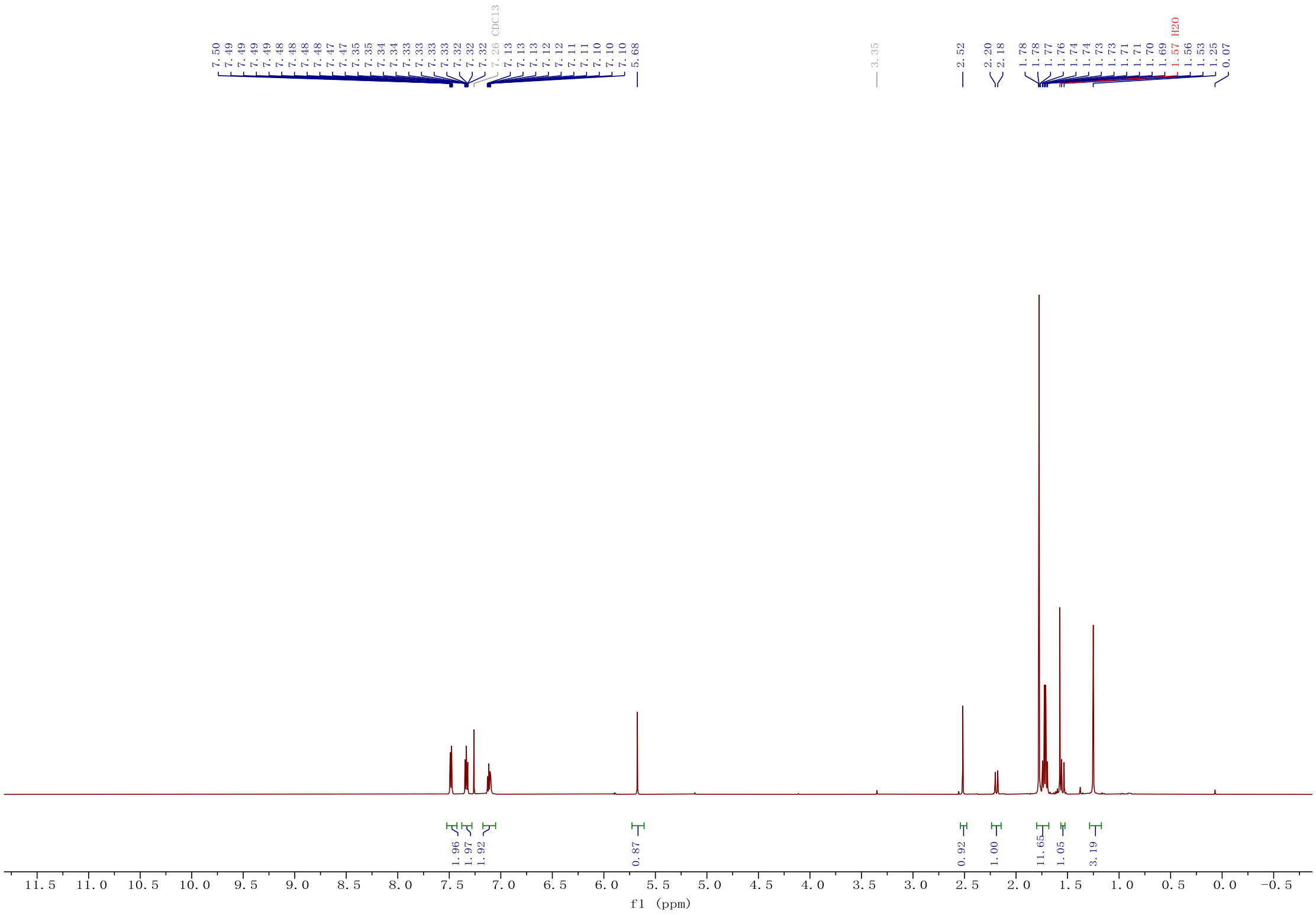

^{13}C NMR (151 MHz, $CDCl_3$)

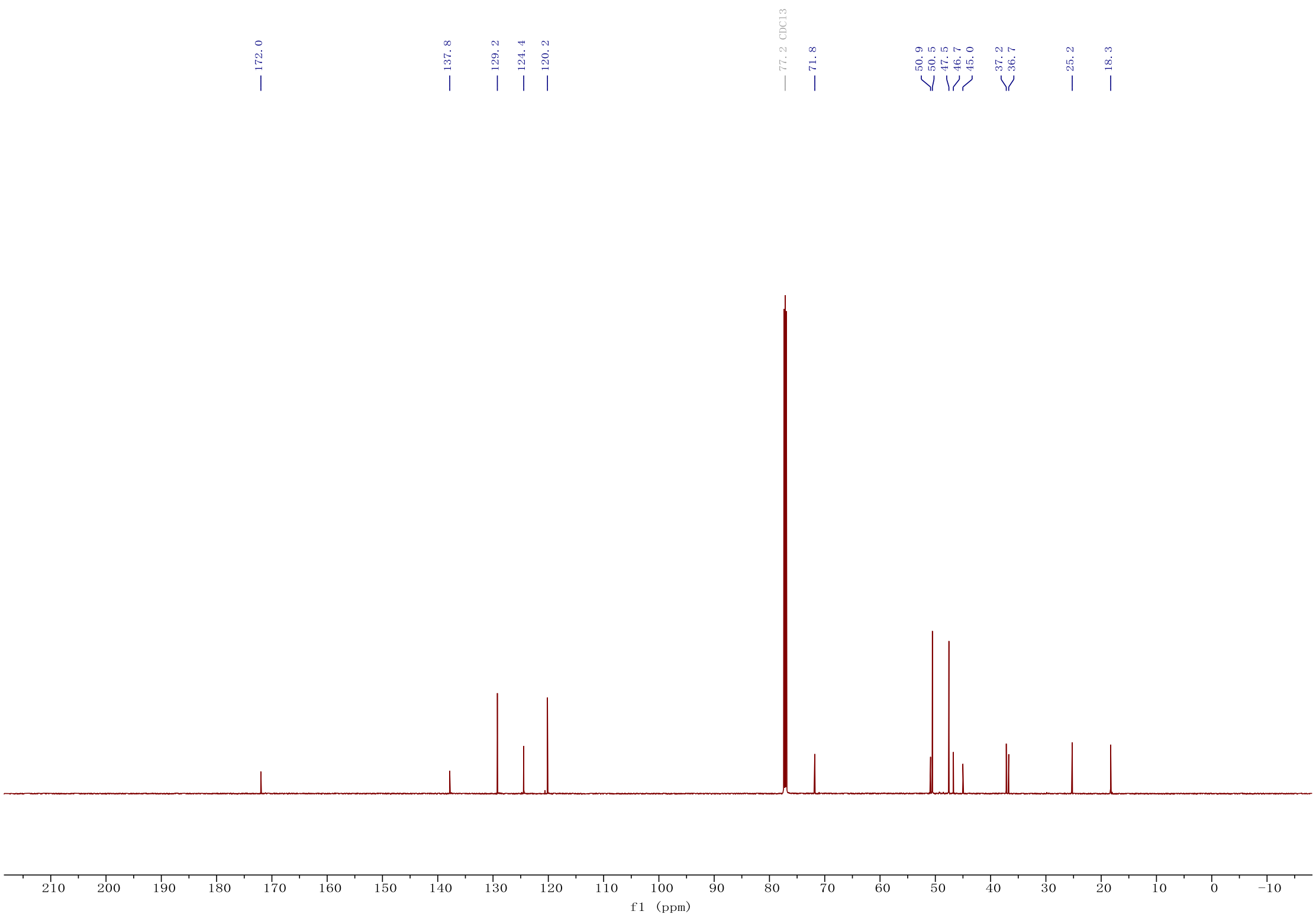

2-(bicyclo[1.1.1]pentan-1-yl)-3-(3-(dichloromethyl)bicyclo[1.1.1]pentan-1-yl)-*N*-(4-methoxyphenyl)-2-methylpropanamide (3r)

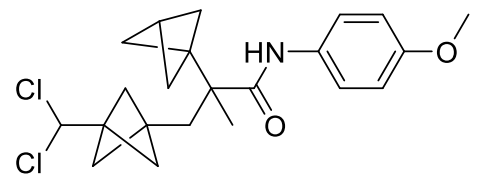

^{1}H NMR (600 MHz, CDCl$_3$)

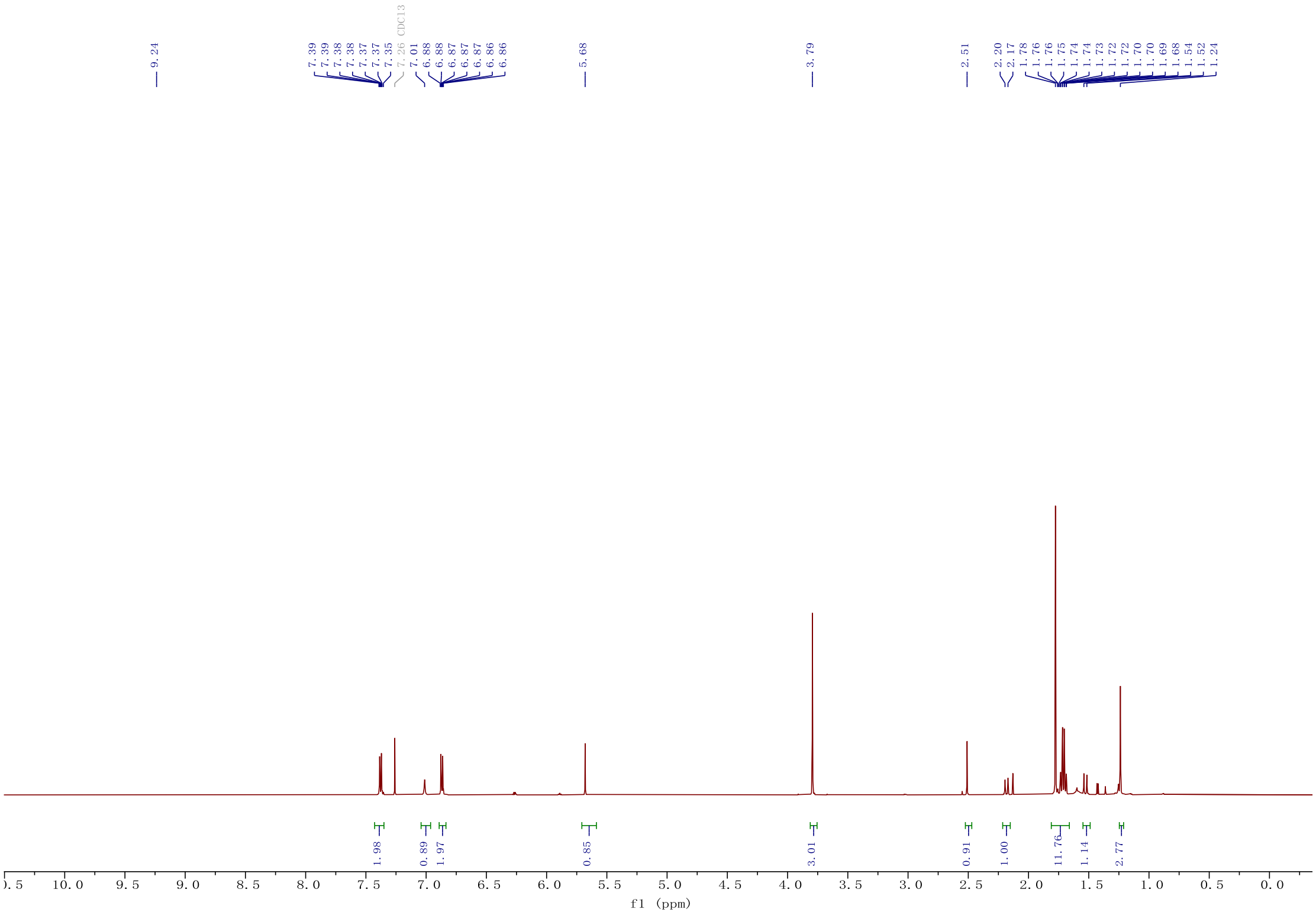

^{13}C NMR (151 MHz, CDCl$_3$)

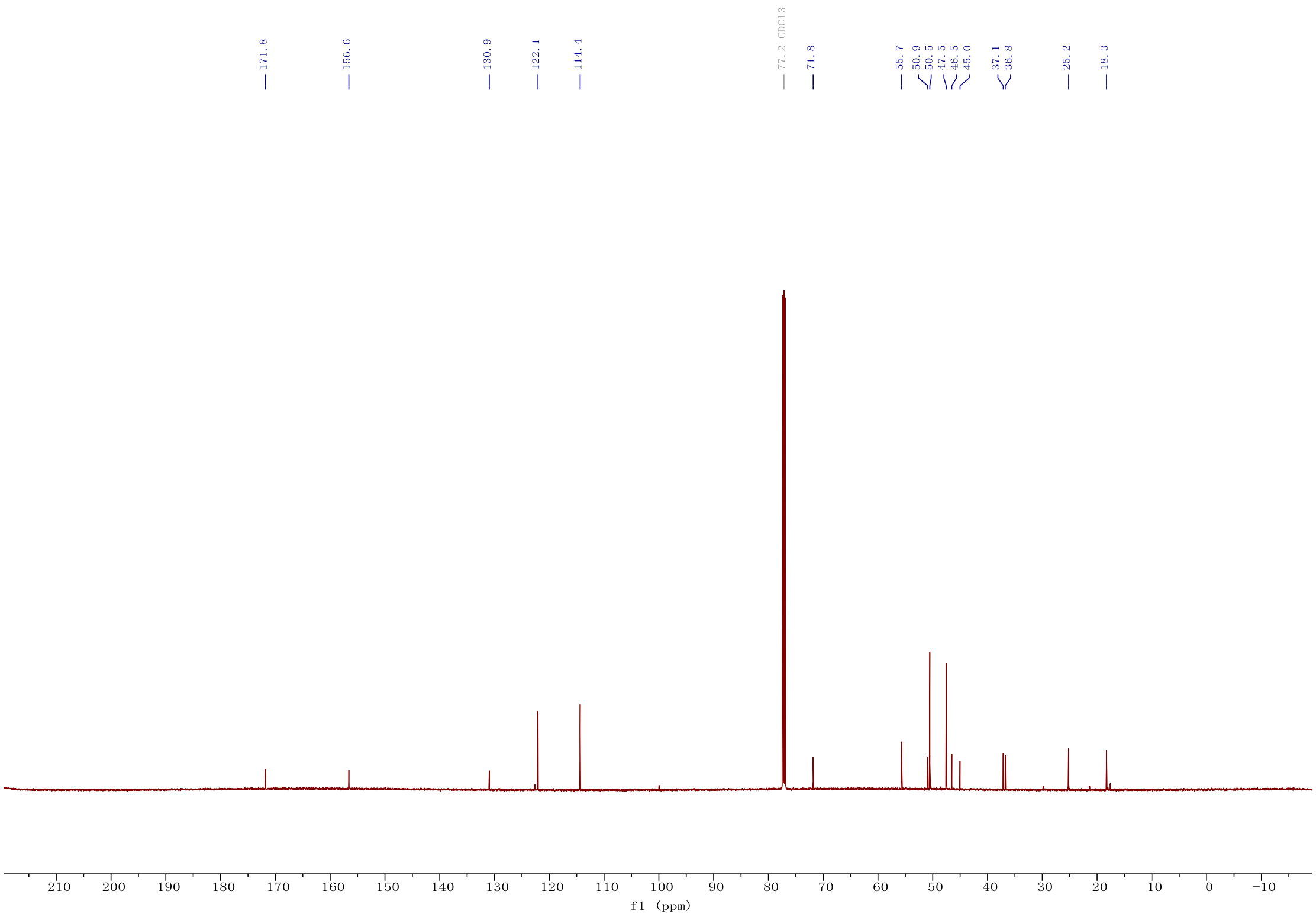

2-(bicyclo[1.1.1]pentan-1-yl)-3-(3-(dichloromethyl)bicyclo[1.1.1]pentan-1-yl)-2-methyl-*N*-(4-(trifluoromethyl)phenyl)propenamide (3s)

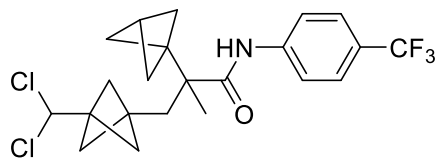

^{1}H NMR (600 MHz, CDCl3)

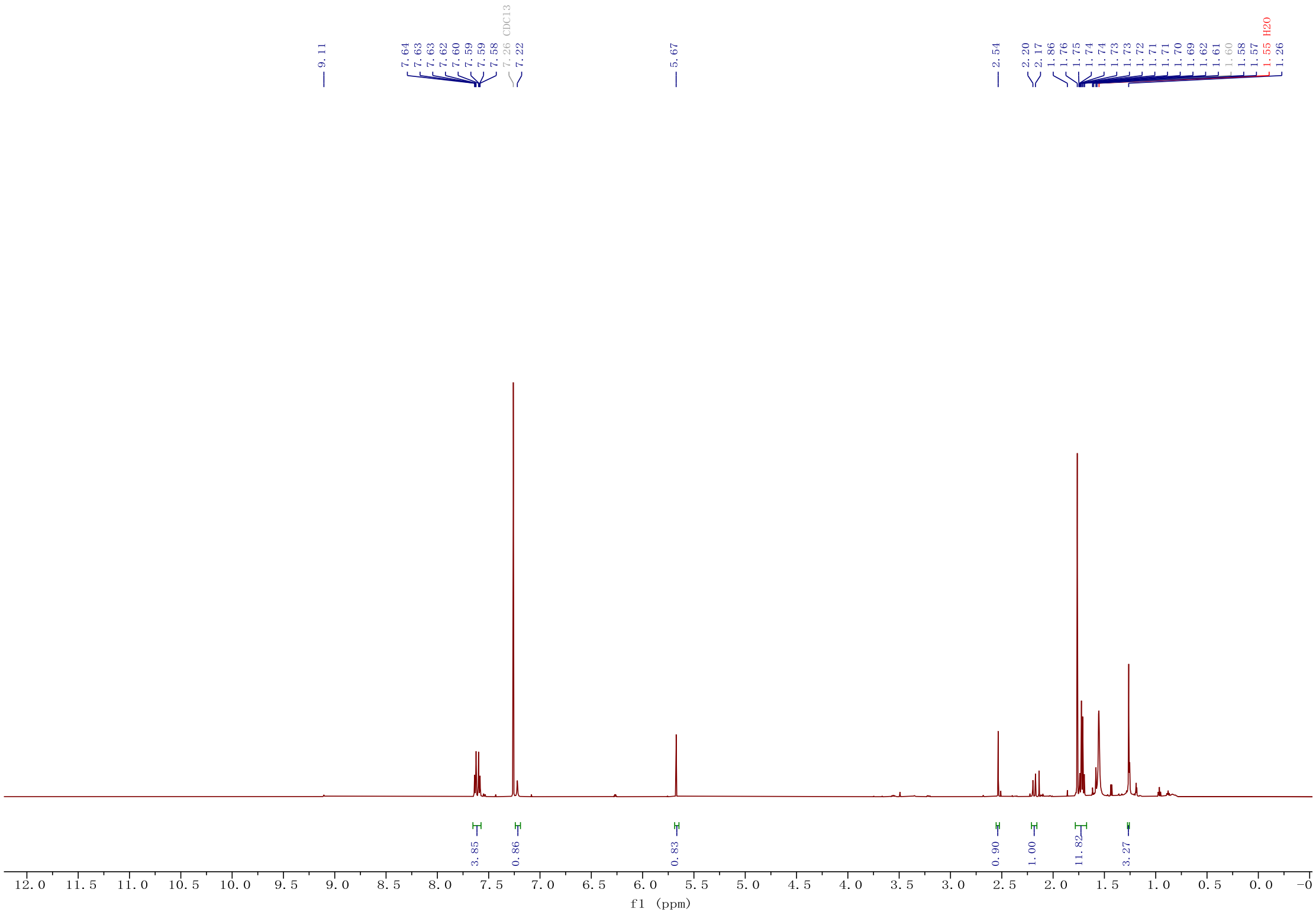

^{13}C NMR (151 MHz, CDCl3)

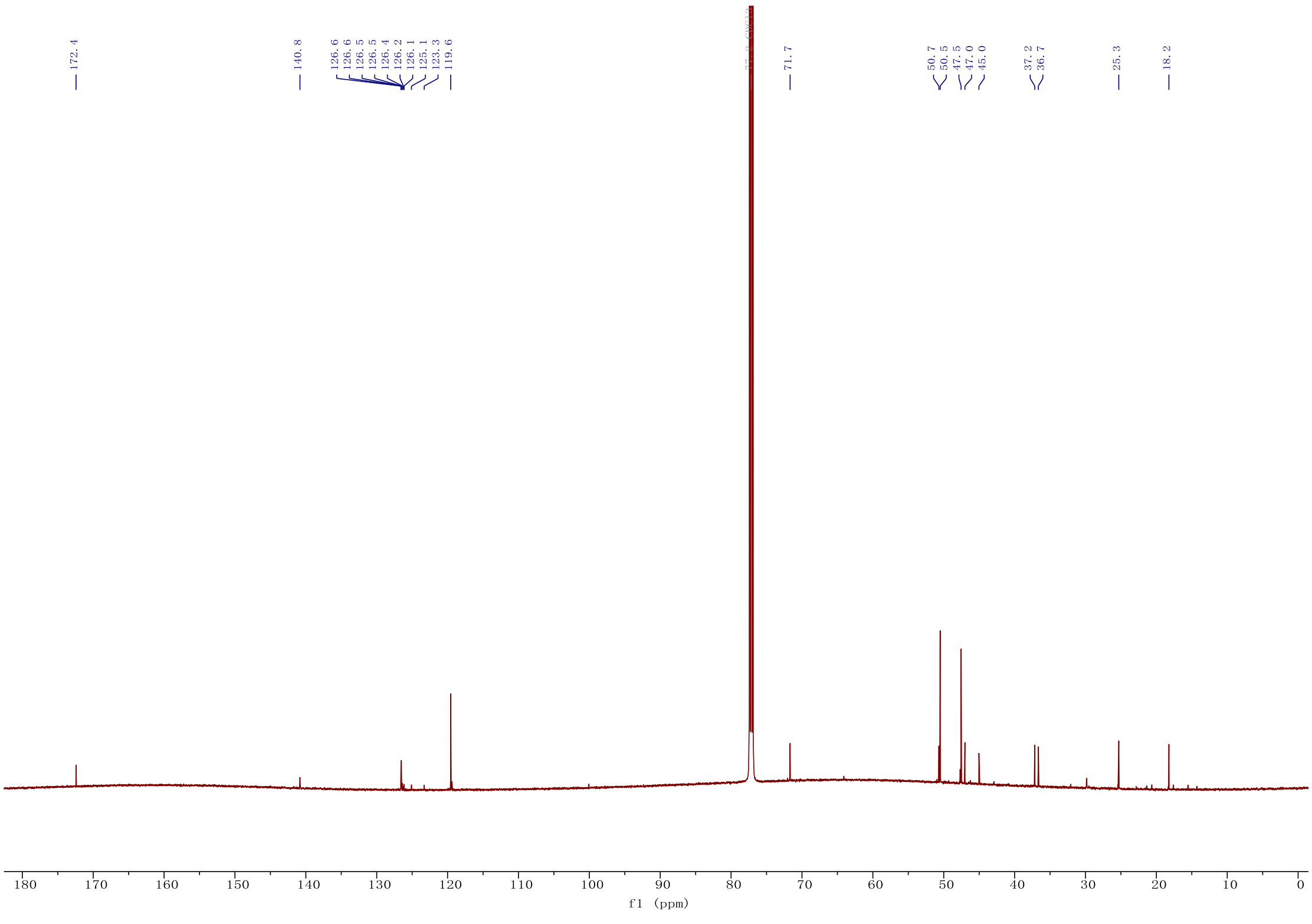

2-(bicyclo[1.1.1]pentan-1-yl)-3-(3-(dichloromethyl)bicyclo[1.1.1]pentan-1-yl)-2-methyl-*N*-phenylbutanamide (3t)

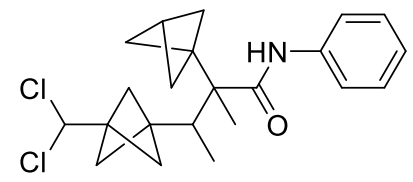

^{1}H NMR (600 MHz, CDCl$_3$)

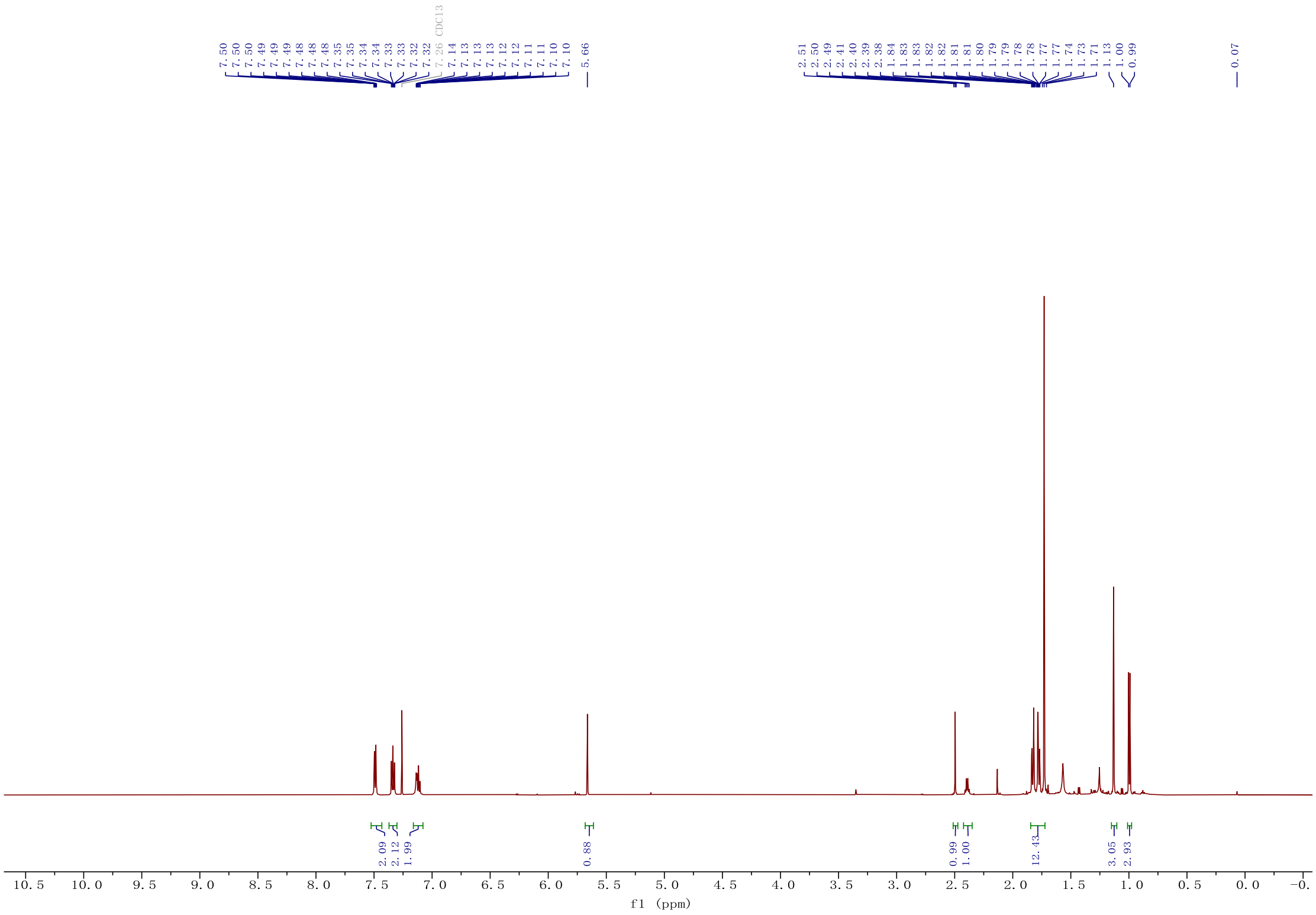

^{13}C NMR (151 MHz, CDCl$_3$)

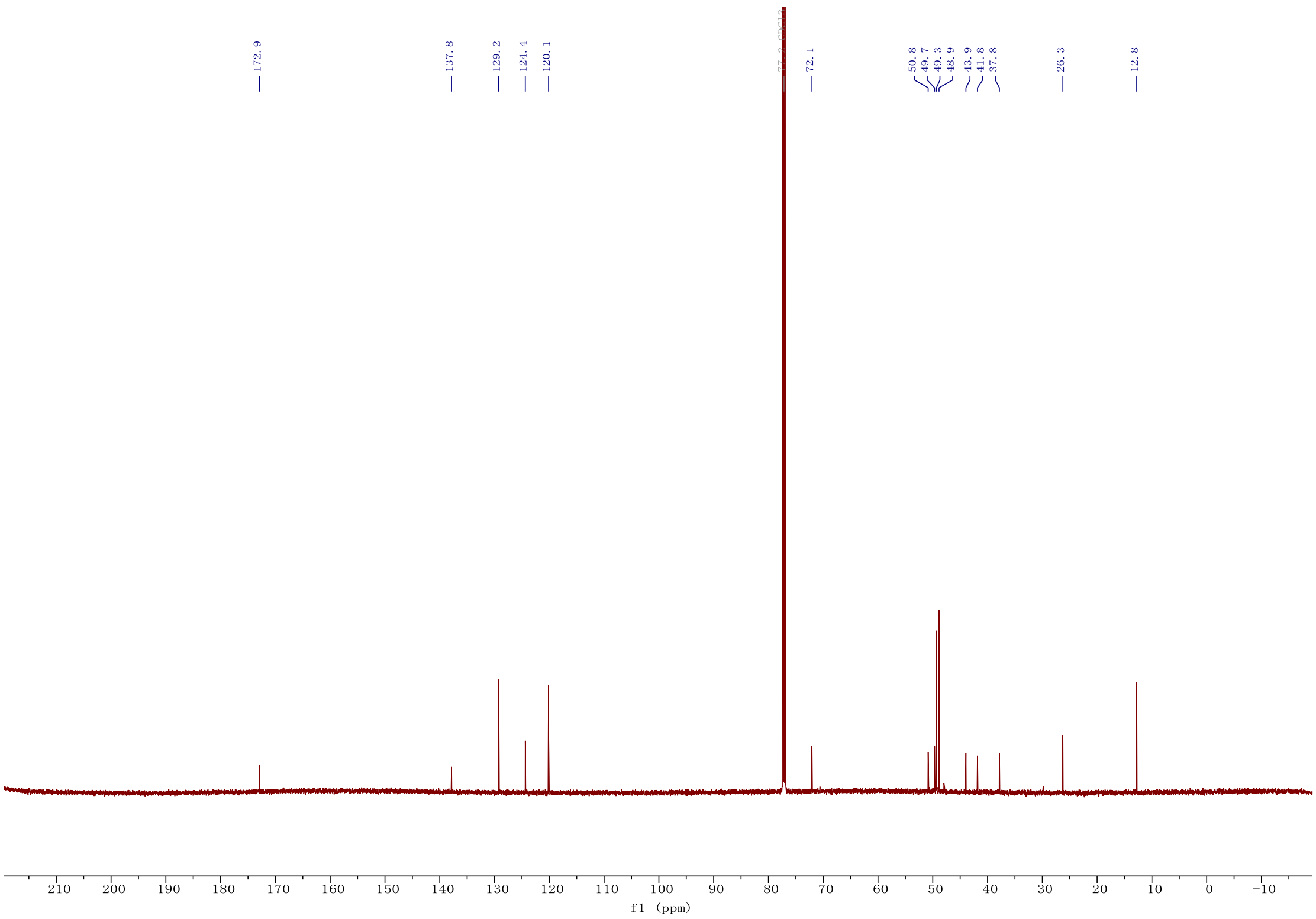

(5*S*)-3-(2-(bicyclo[1.1.1]pentan-1-yl)-3-(3-(dichloromethyl)bicyclo[1.1.1]pentan-1-yl)propanoyl)-5-isopropyloxazolidin-2-one (3ut, top isomer)

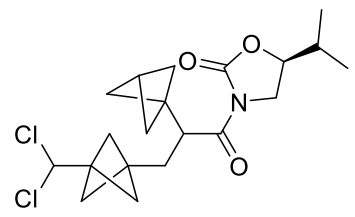

^{1}H NMR (600 MHz, CDCl$_3$)

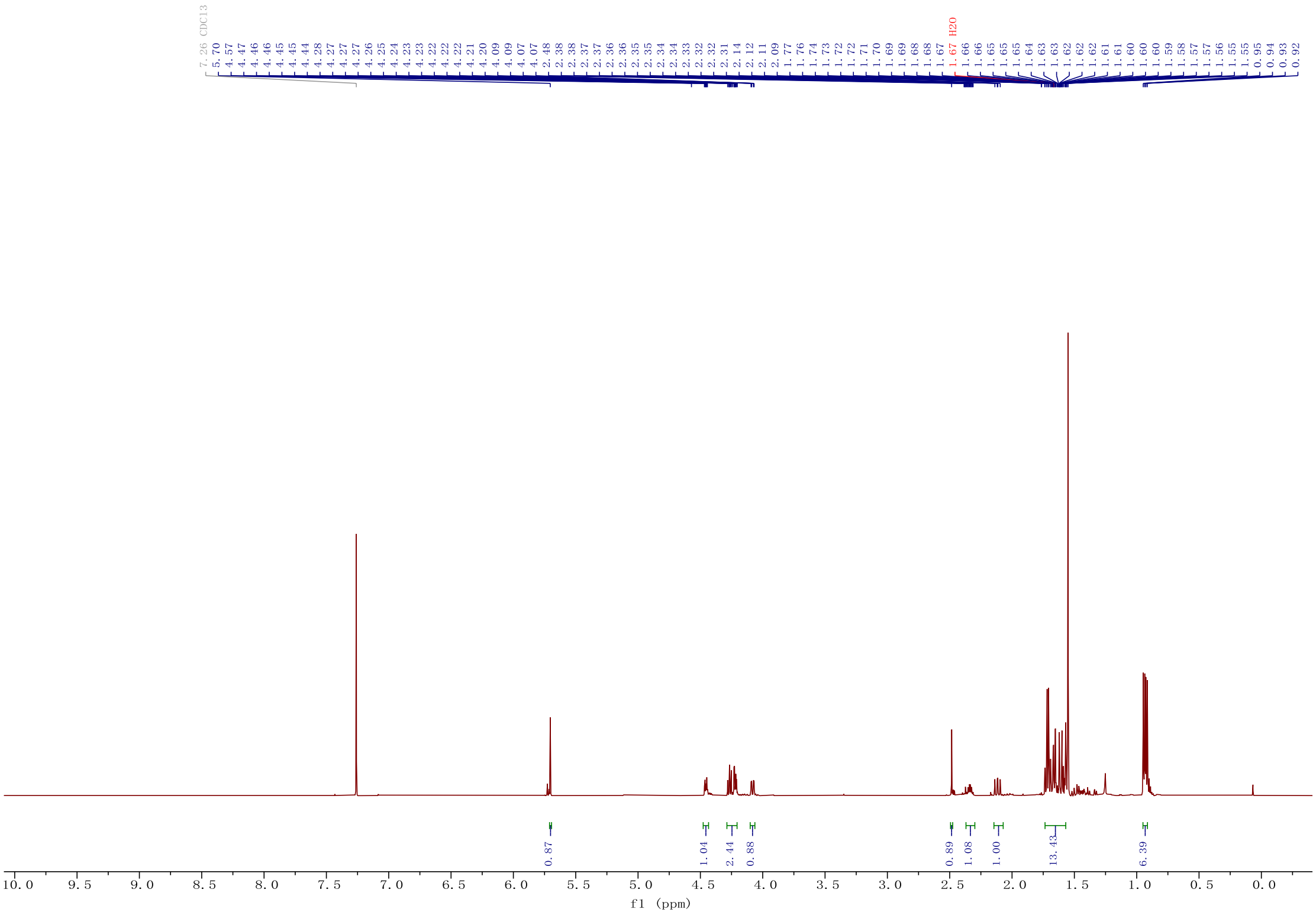

^{13}C NMR (151 MHz, CDCl$_3$)

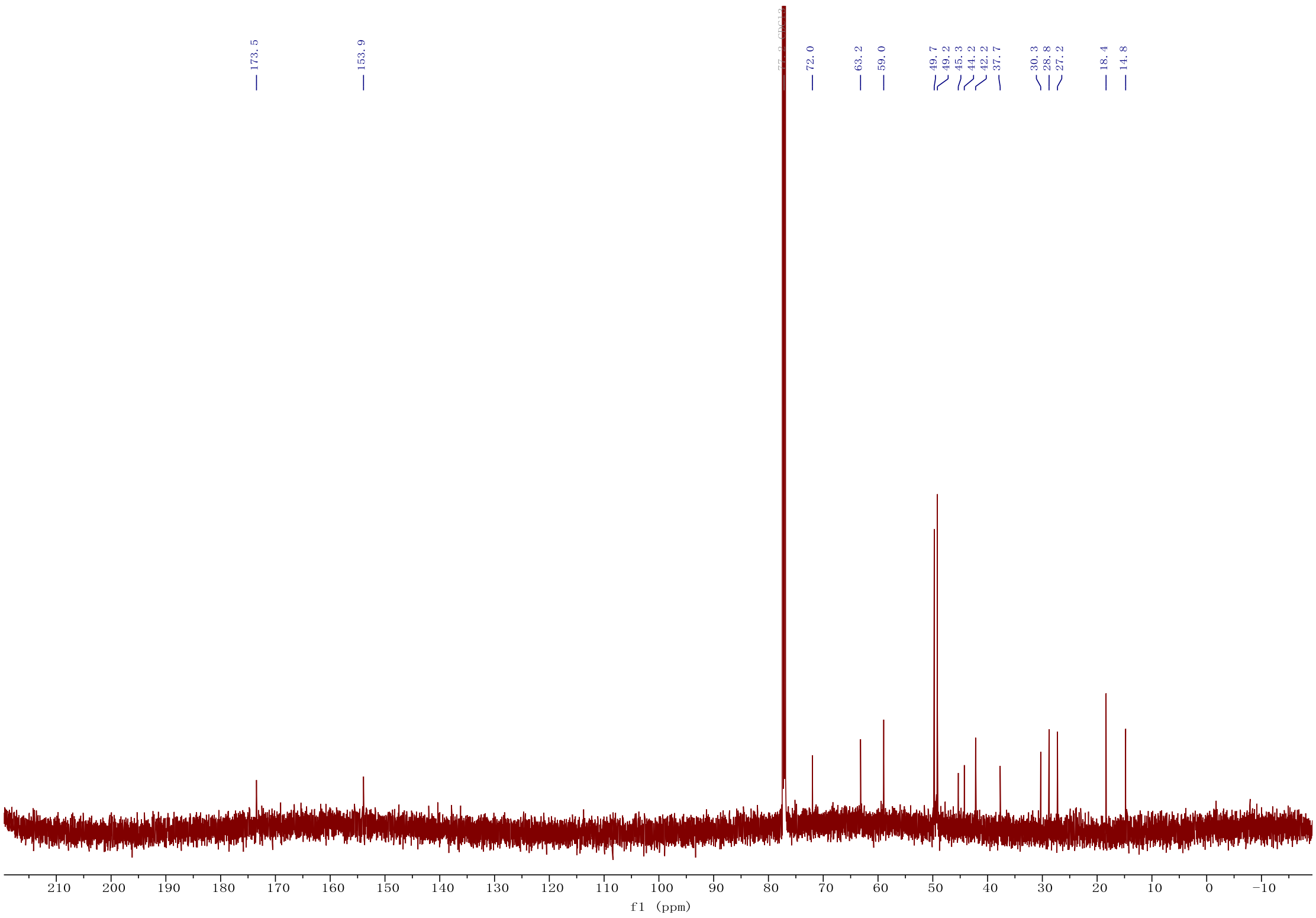

(5*S*)-3-(2-(bicyclo[1.1.1]pentan-1-yl)-3-(3-(dichloromethyl)bicyclo[1.1.1]pentan-1-yl)propanoyl)-5-isopropyloxazolidin-2-one (3ub, bottom isomer)

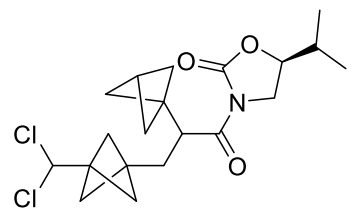

^{1}H NMR (600 MHz, CDCl3)

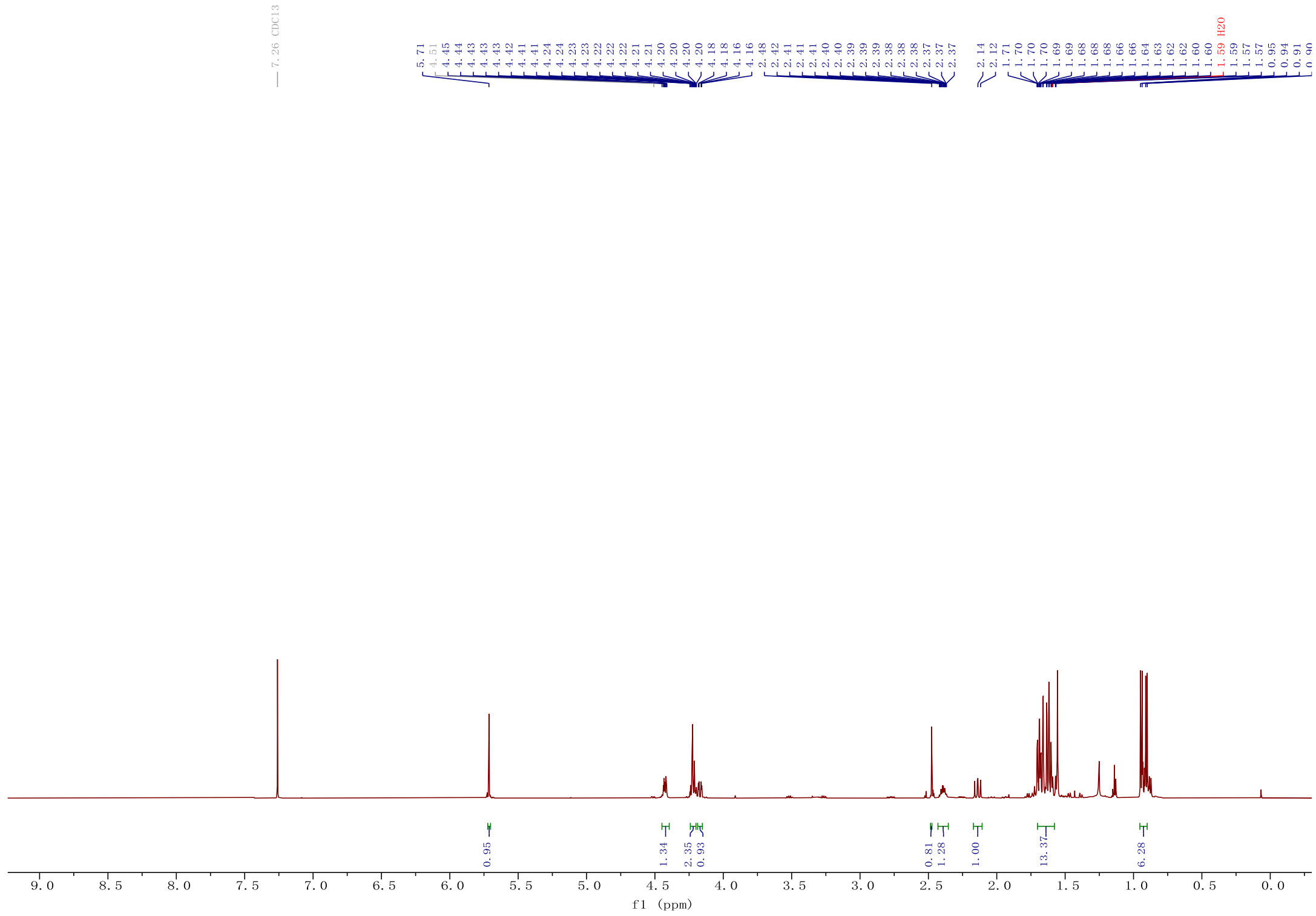

^{13}C NMR (151 MHz, CDCl3)

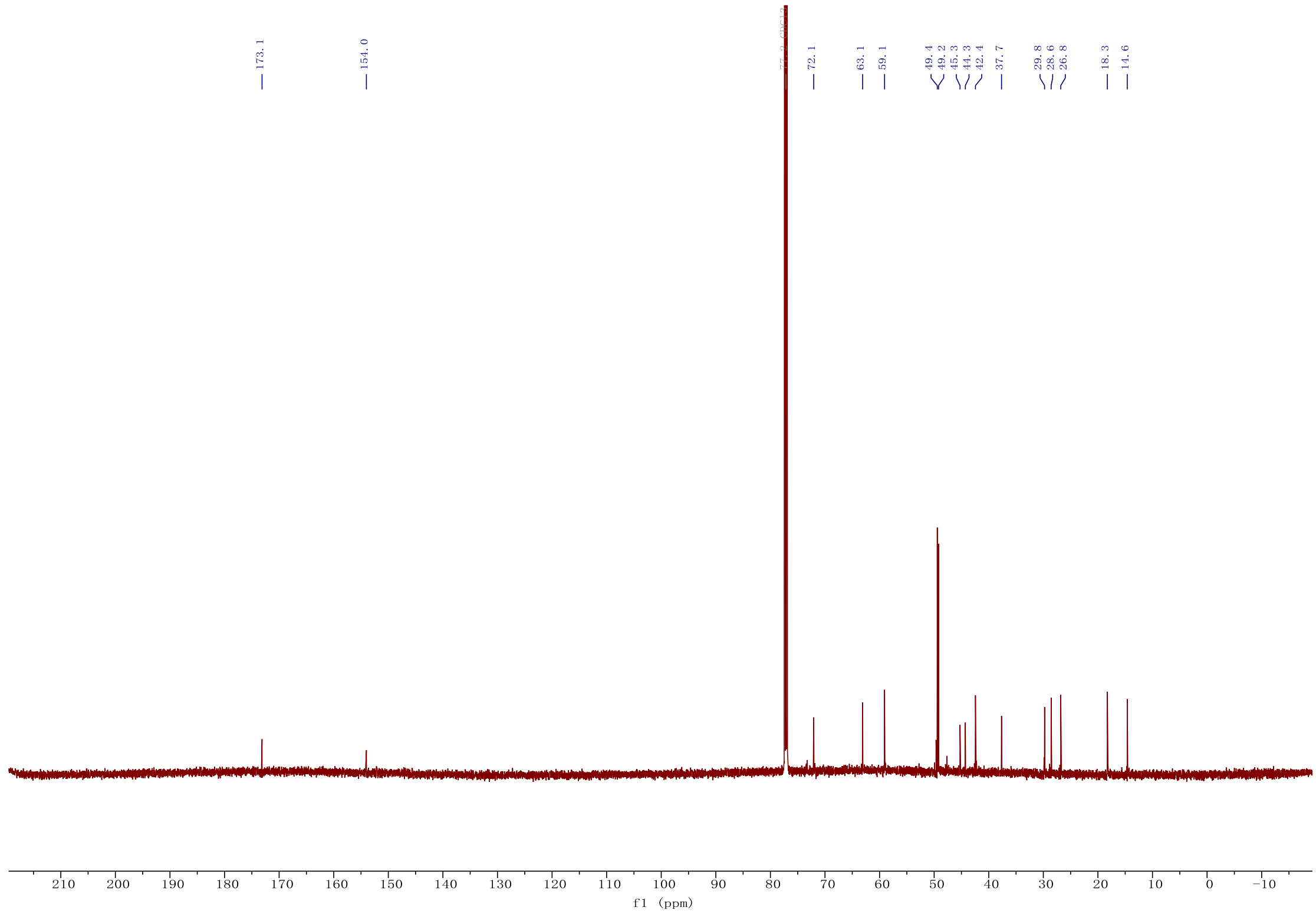

methyl (2-(bicyclo[1.1.1]pentan-1-yl)-3-(3-(dichloromethyl)bicyclo[1.1.1]pentan-1-yl)propanoyl)-*L*-phenylalanyl-*L*-leucinate (3v)

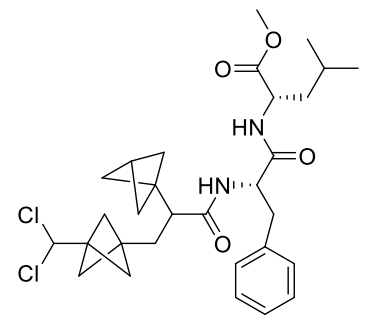

^{1}H NMR (600 MHz, $CDCl_3$)

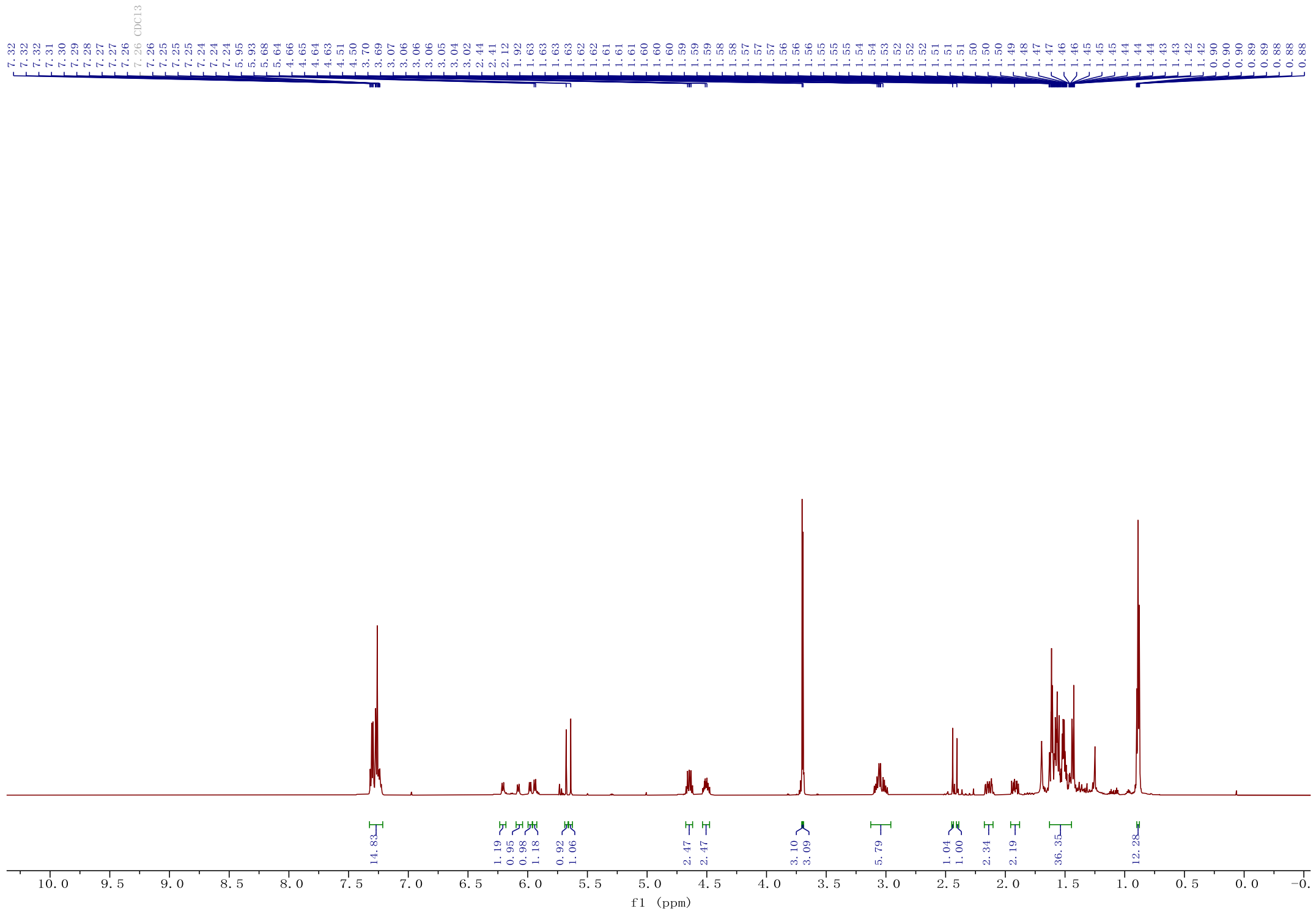

^{13}C NMR (151 MHz, $CDCl_3$)

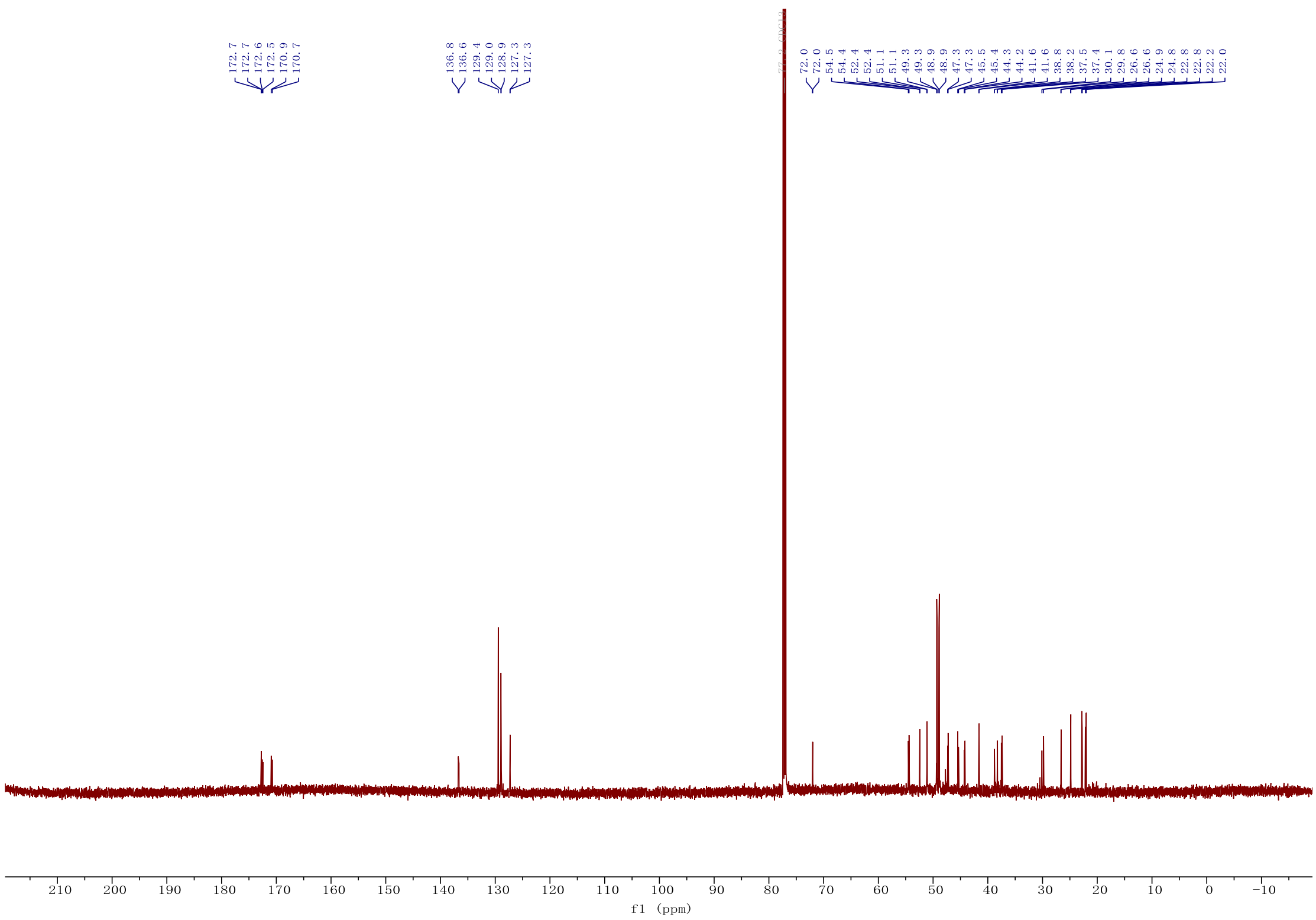

2-(bicyclo[3.1.1]heptan-1-yl)-3-(5-(dichloromethyl)bicyclo[3.1.1]heptan-1-yl)-*N*-phenylpropanamide (6a)

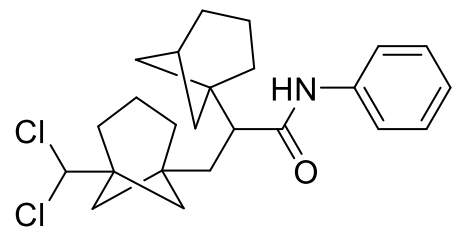

^{1}H NMR (600 MHz, CDCl$_3$)

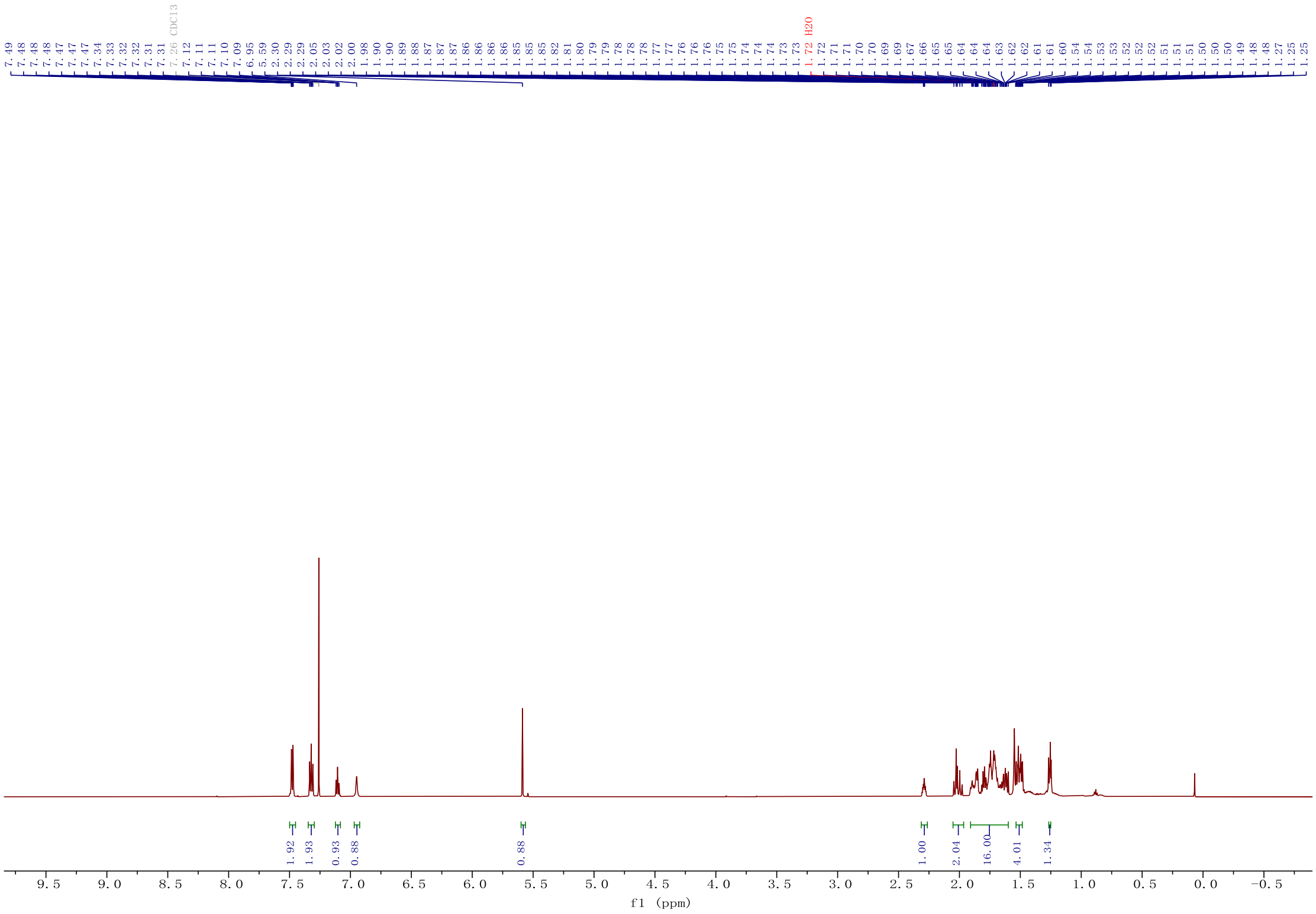

^{13}C NMR (151 MHz, CDCl$_3$)

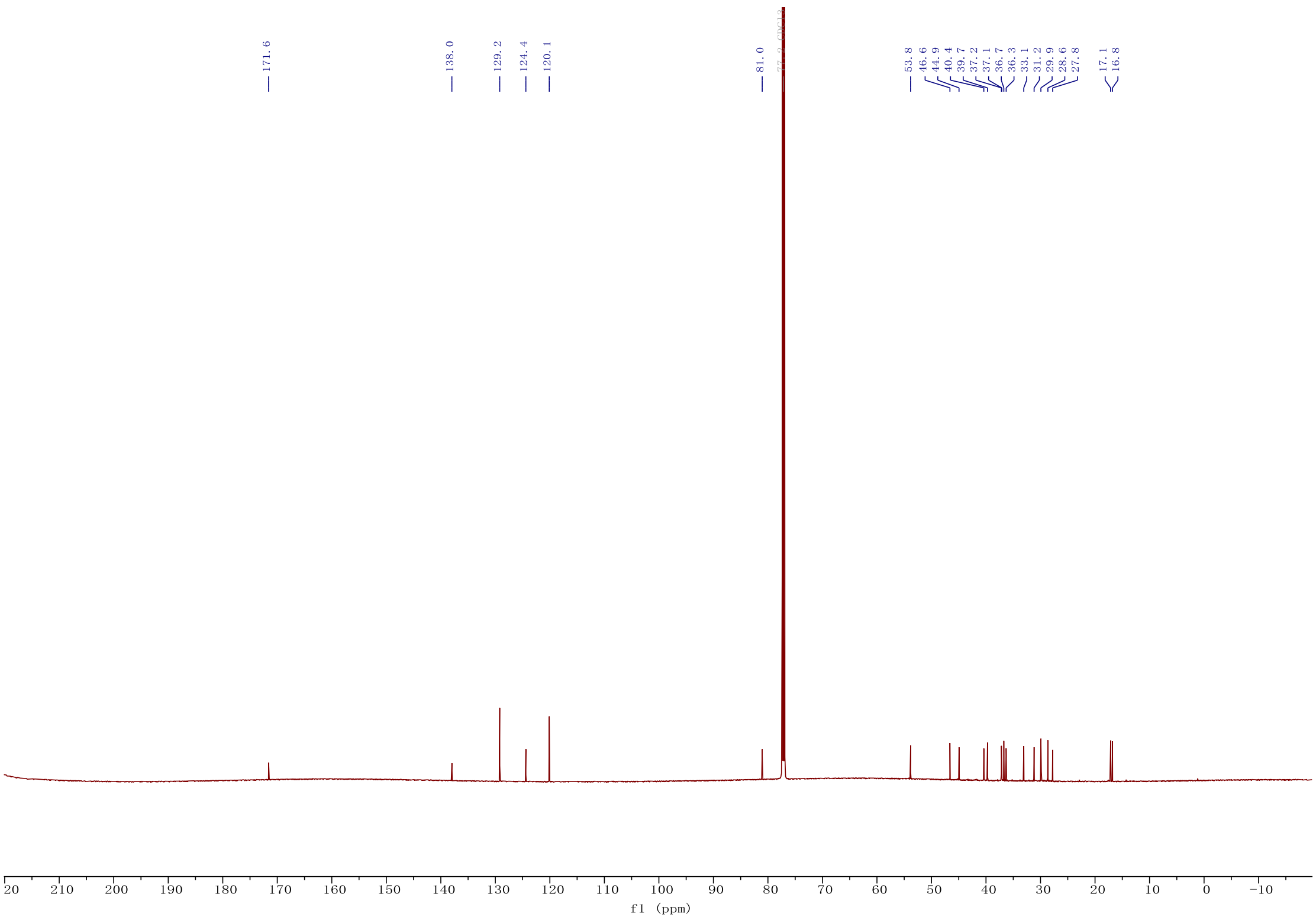

2-(bicyclo[3.1.1]heptan-1-yl)-3-(5-(dichloromethyl)bicyclo[3.1.1]heptan-1-yl)-*N*-(4-methoxyphenyl)propenamide (6b)

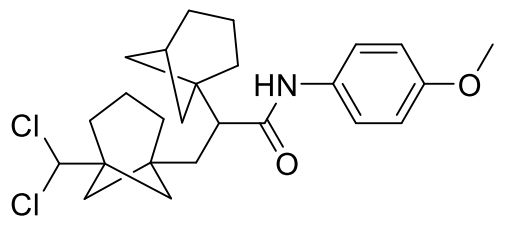

^{1}H NMR (600 MHz, $CDCl_3$)

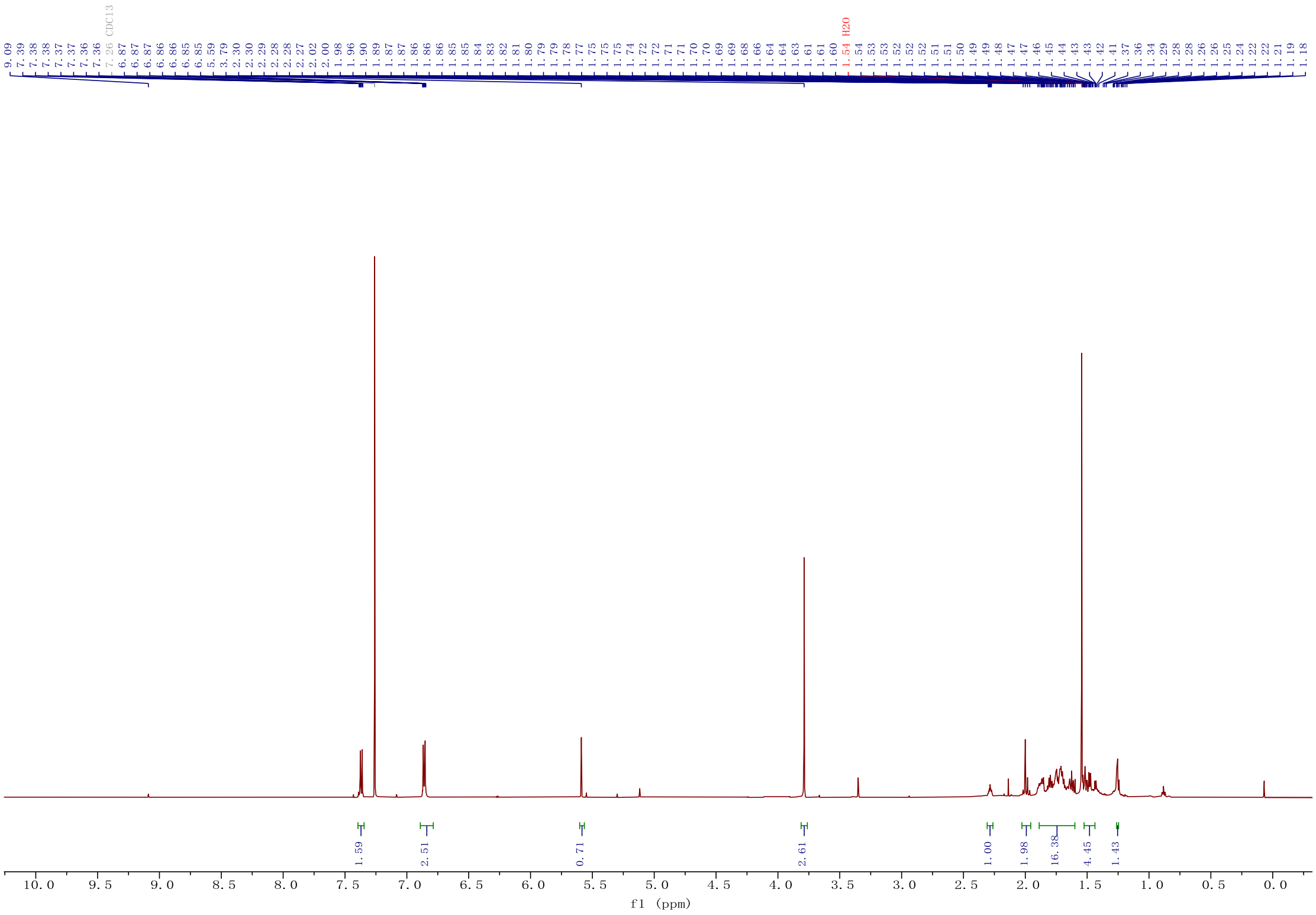

^{13}C NMR (151 MHz, $CDCl_3$)

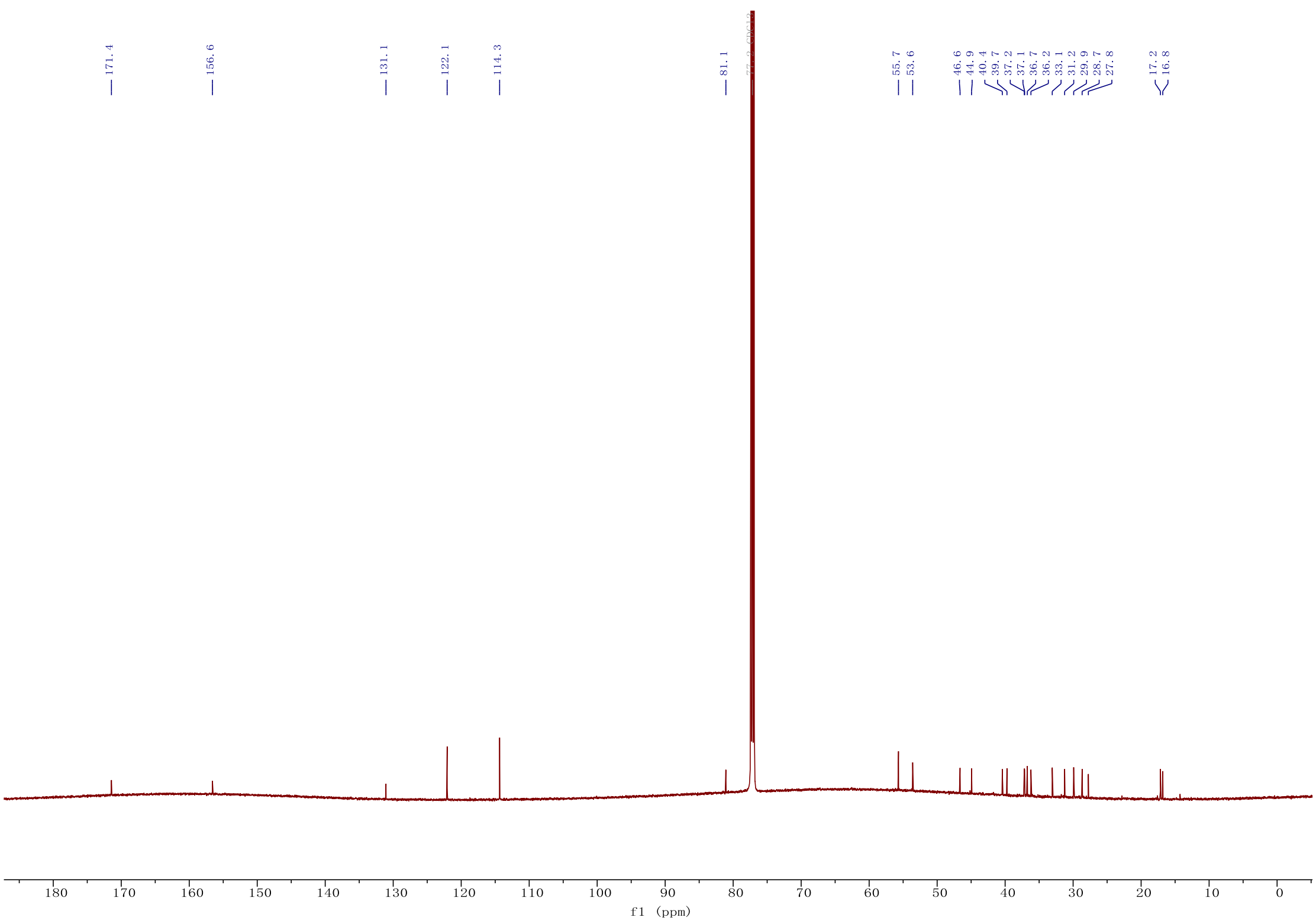

2-(bicyclo[3.1.1]heptan-1-yl)-3-(5-(dichloromethyl)bicyclo[3.1.1]heptan-1-yl)-*N*-(2-methyl-4-oxopentan-2-yl)propenamide (6c)

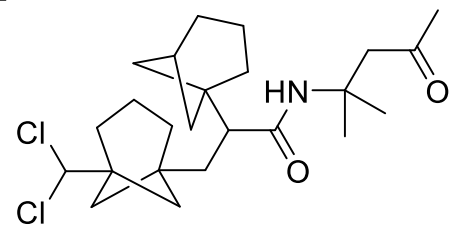

^{1}H NMR (600 MHz, $CDCl_3$)

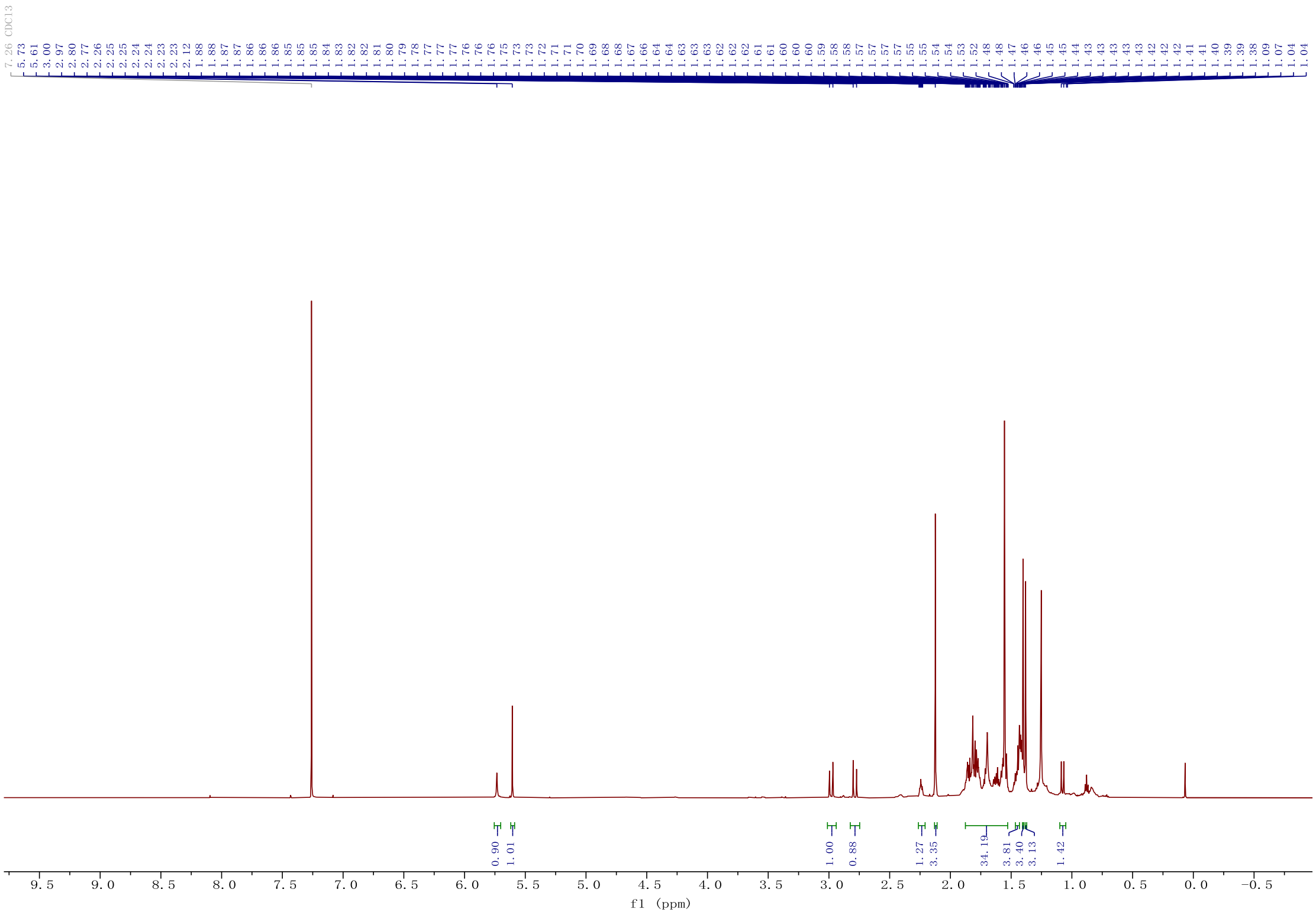

^{13}C NMR (151 MHz, $CDCl_3$)

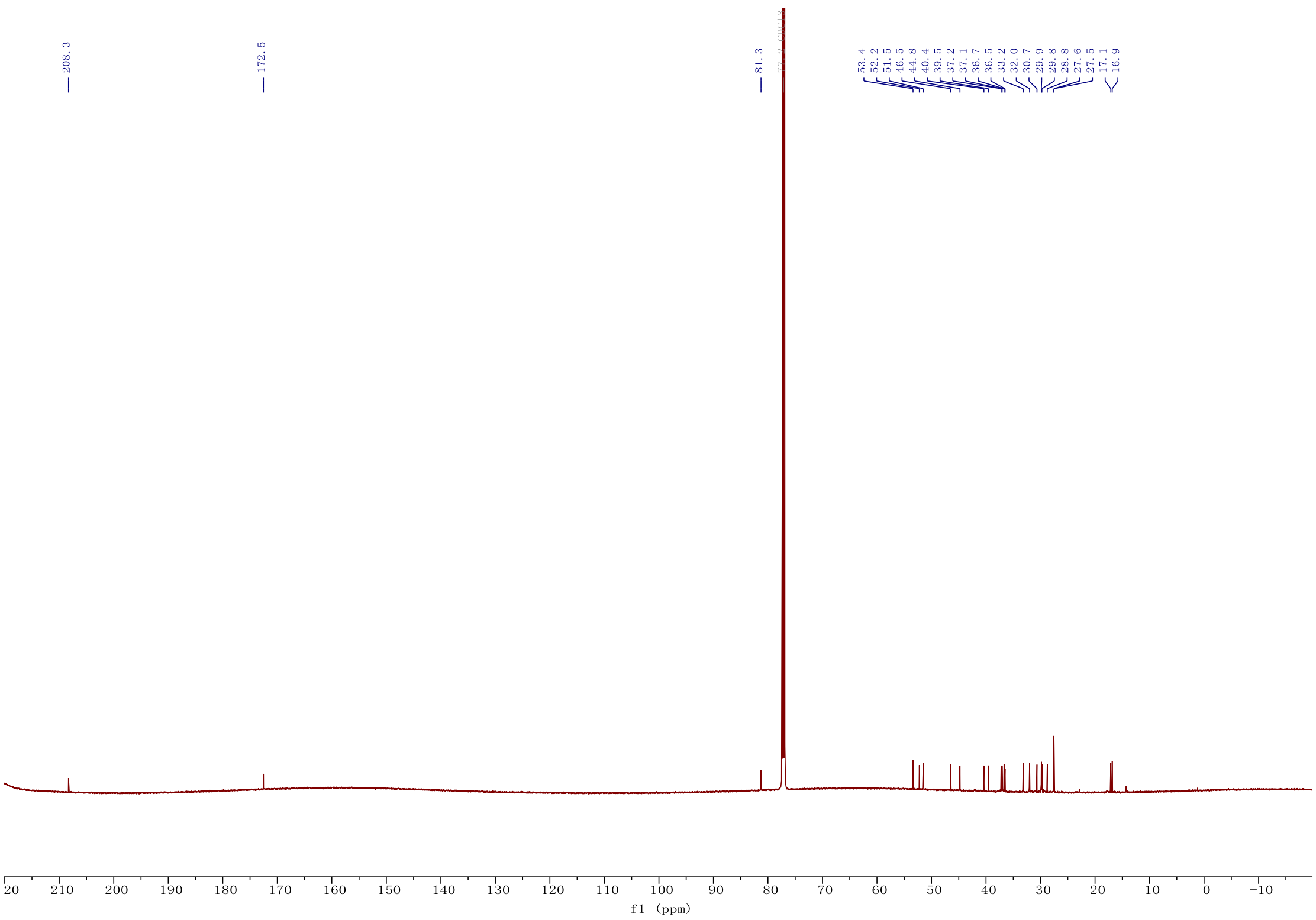

2-(bicyclo[3.1.1]heptan-1-yl)-3-(5-(dichloromethyl)bicyclo[3.1.1]heptan-1-yl)-1-morpholinopropan-1-one (6d)

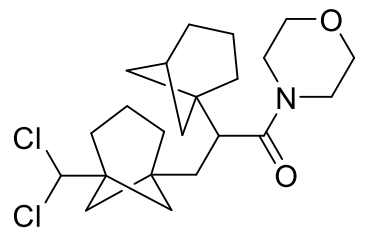

^{1}H NMR (600 MHz, CDCl$_3$)

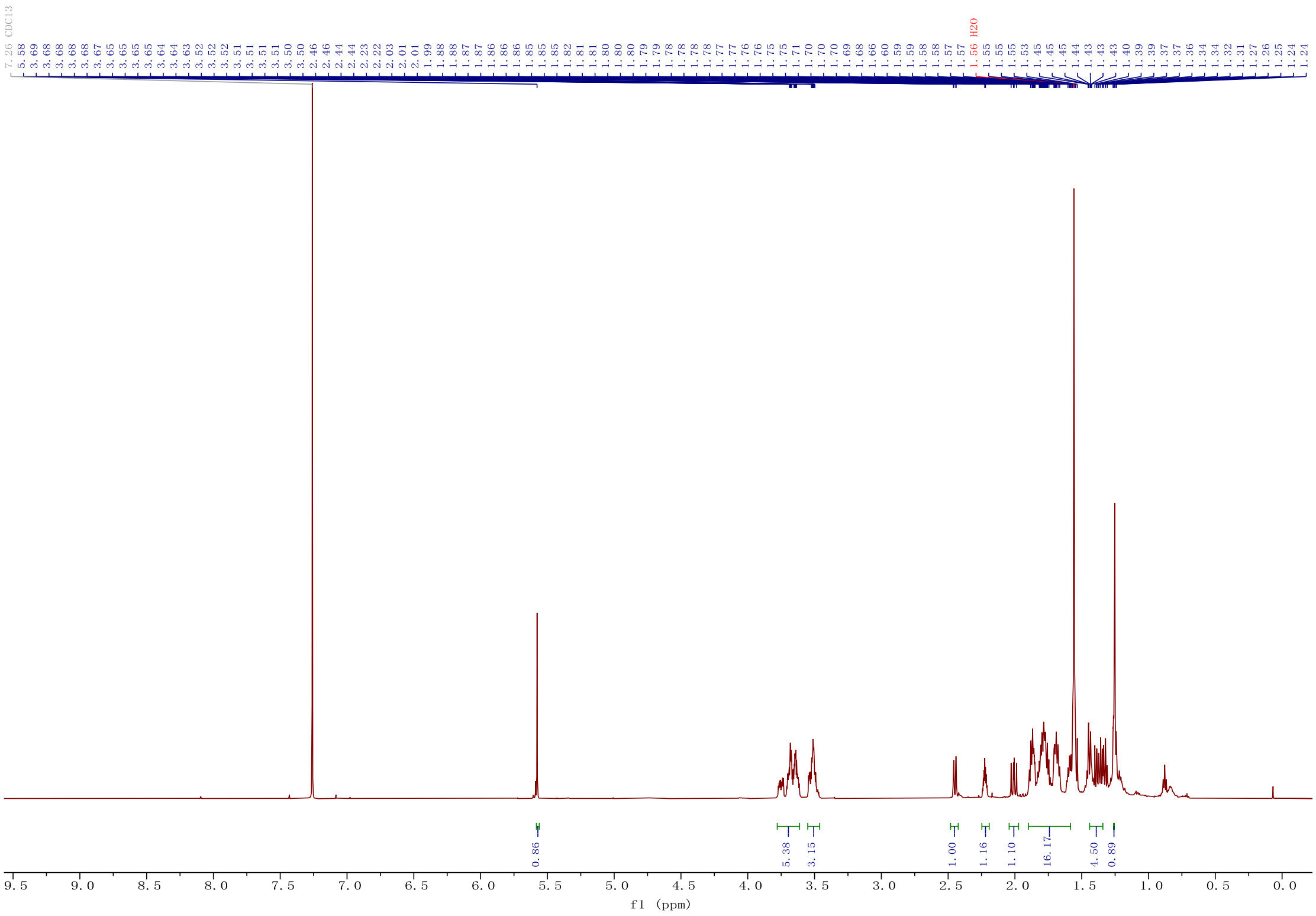

^{13}C NMR (151 MHz, CDCl$_3$)

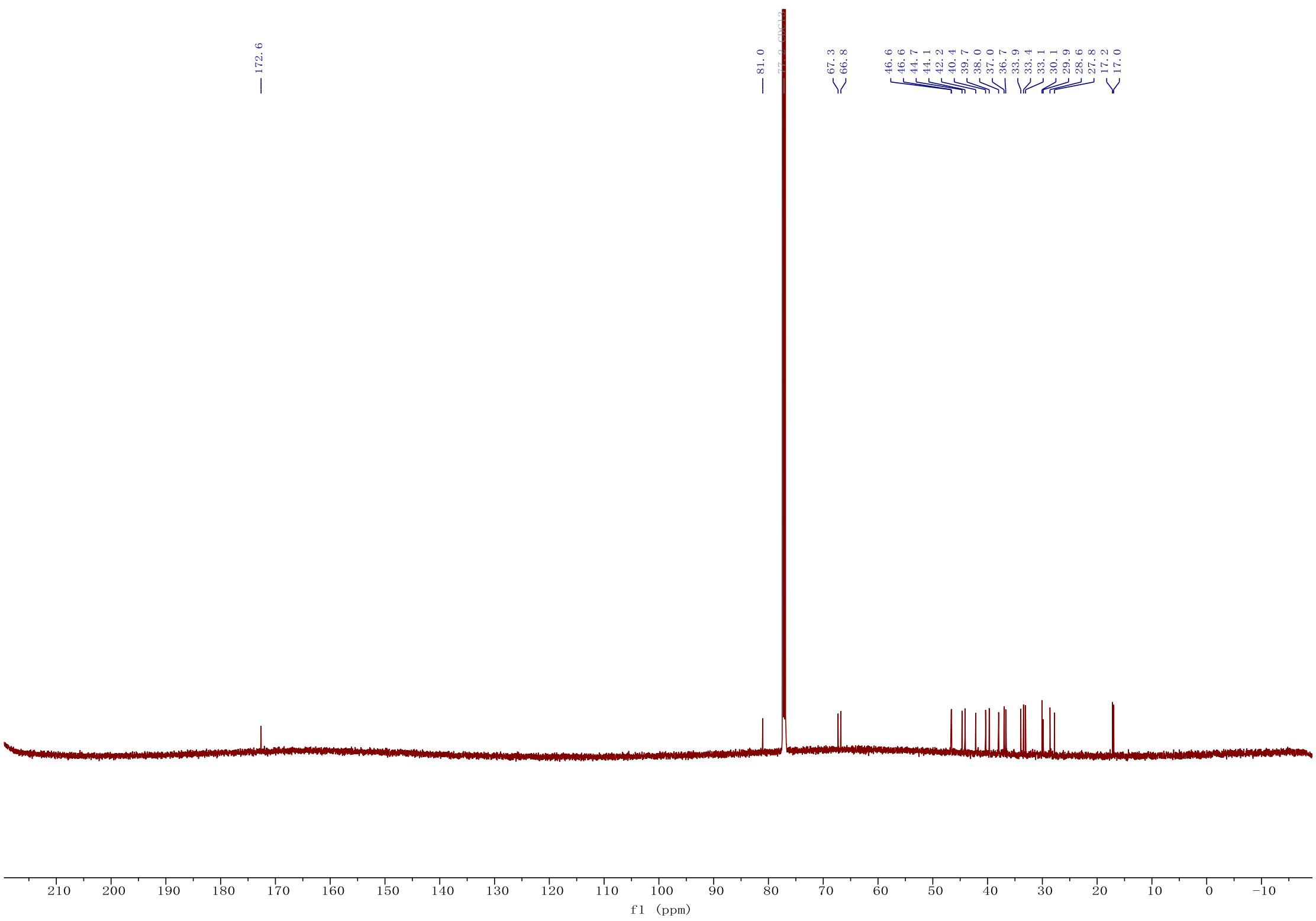

2-(bicyclo[3.1.1]heptan-1-yl)-3-(5-(dichloromethyl)bicyclo[3.1.1]heptan-1-yl)-2-methyl-*N*-phenylpropanamide (6e)

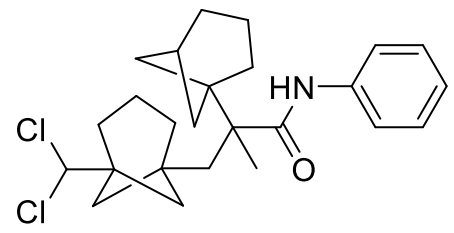

^{1}H NMR (600 MHz, CDCl$_3$)

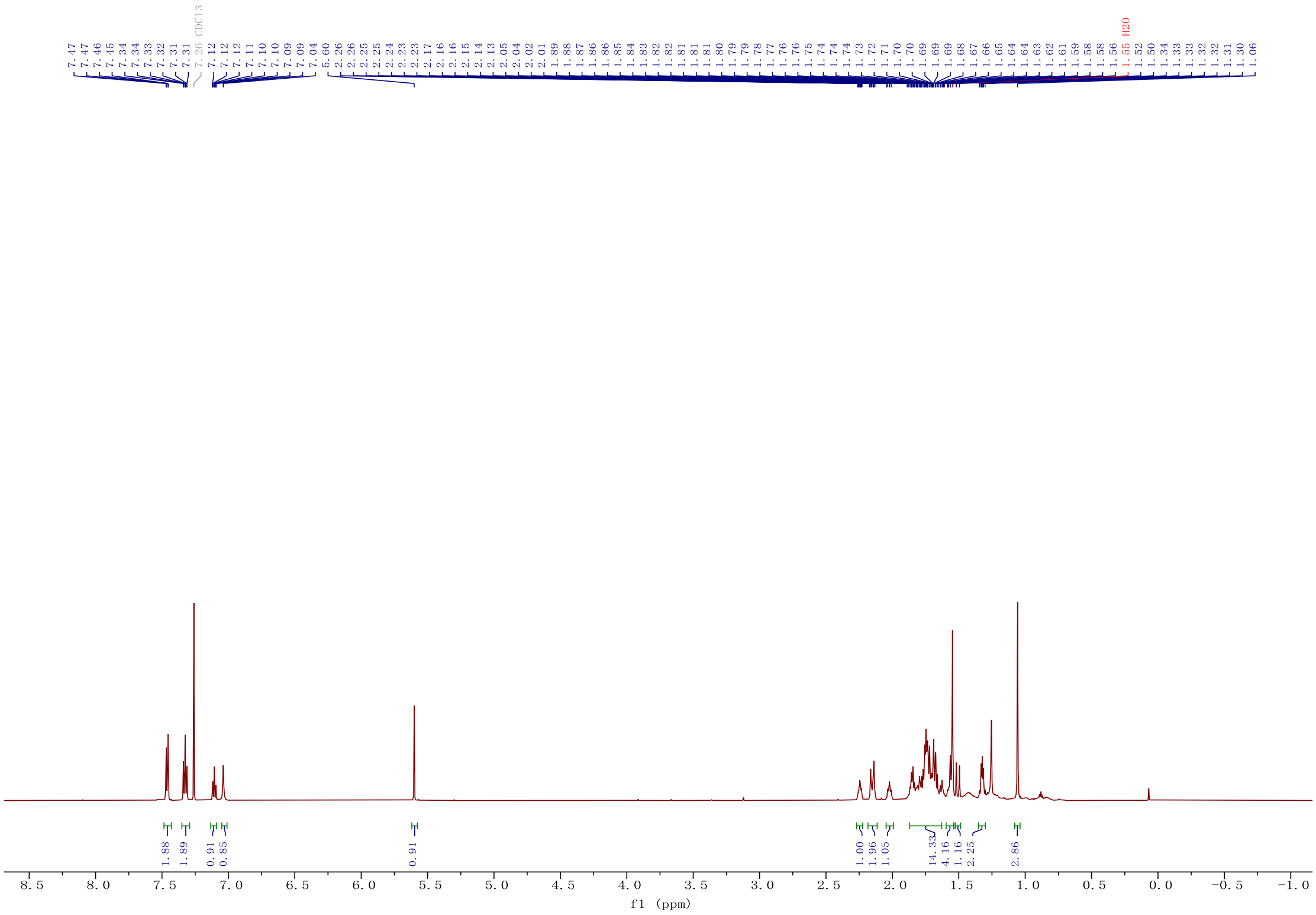

^{13}C NMR (151 MHz, CDCl$_3$)

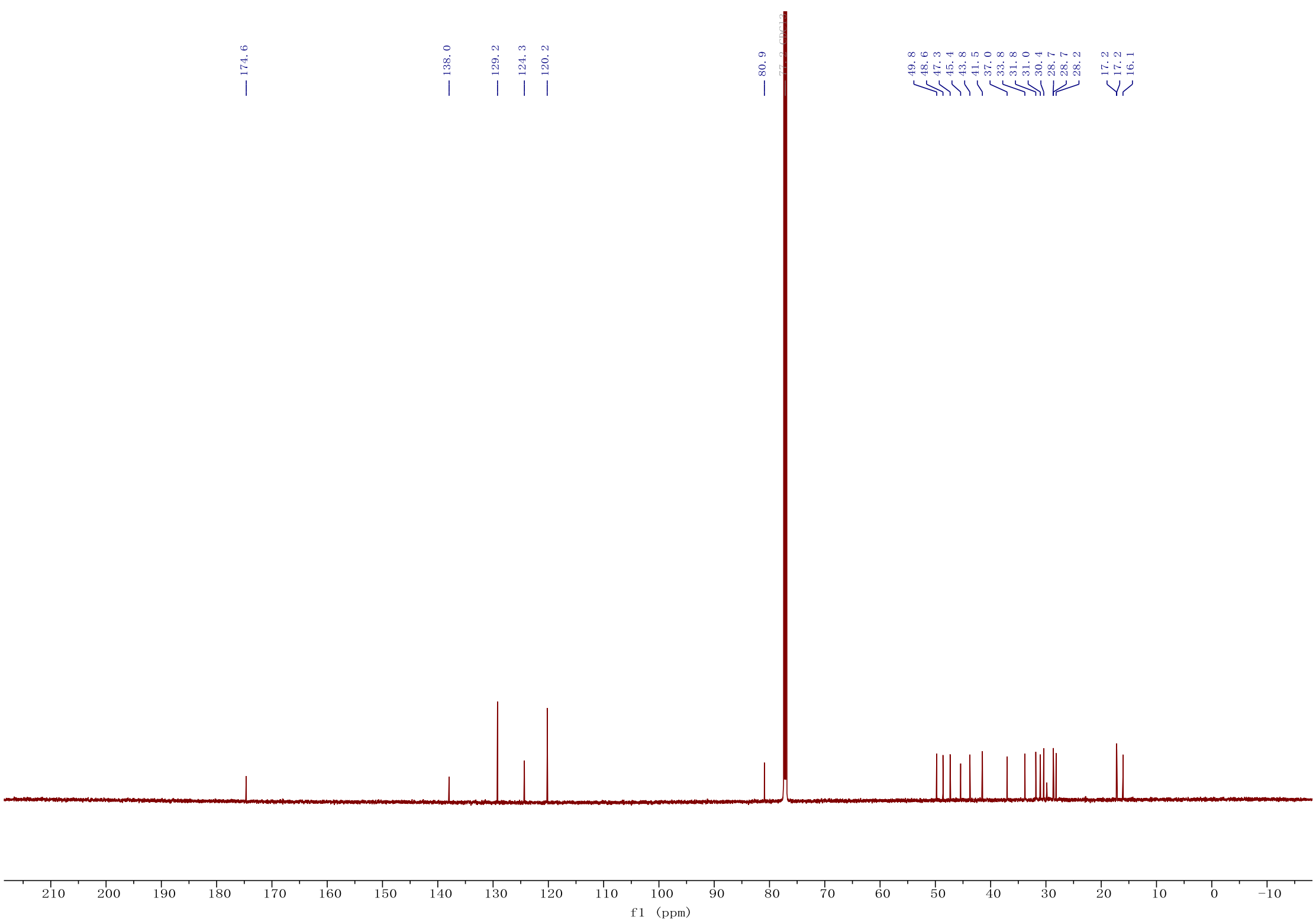

benzyl 2-(bicyclo[1.1.1]pentan-1-yl)-3-(3-(dichloromethyl)bicyclo[1.1.1]pentan-1-yl)propanoate (3aa)

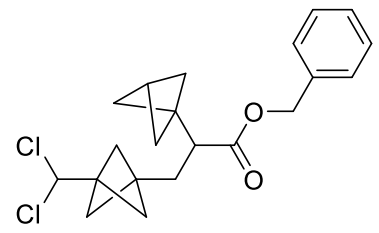

^{1}H NMR (600 MHz, CDCl$_3$)

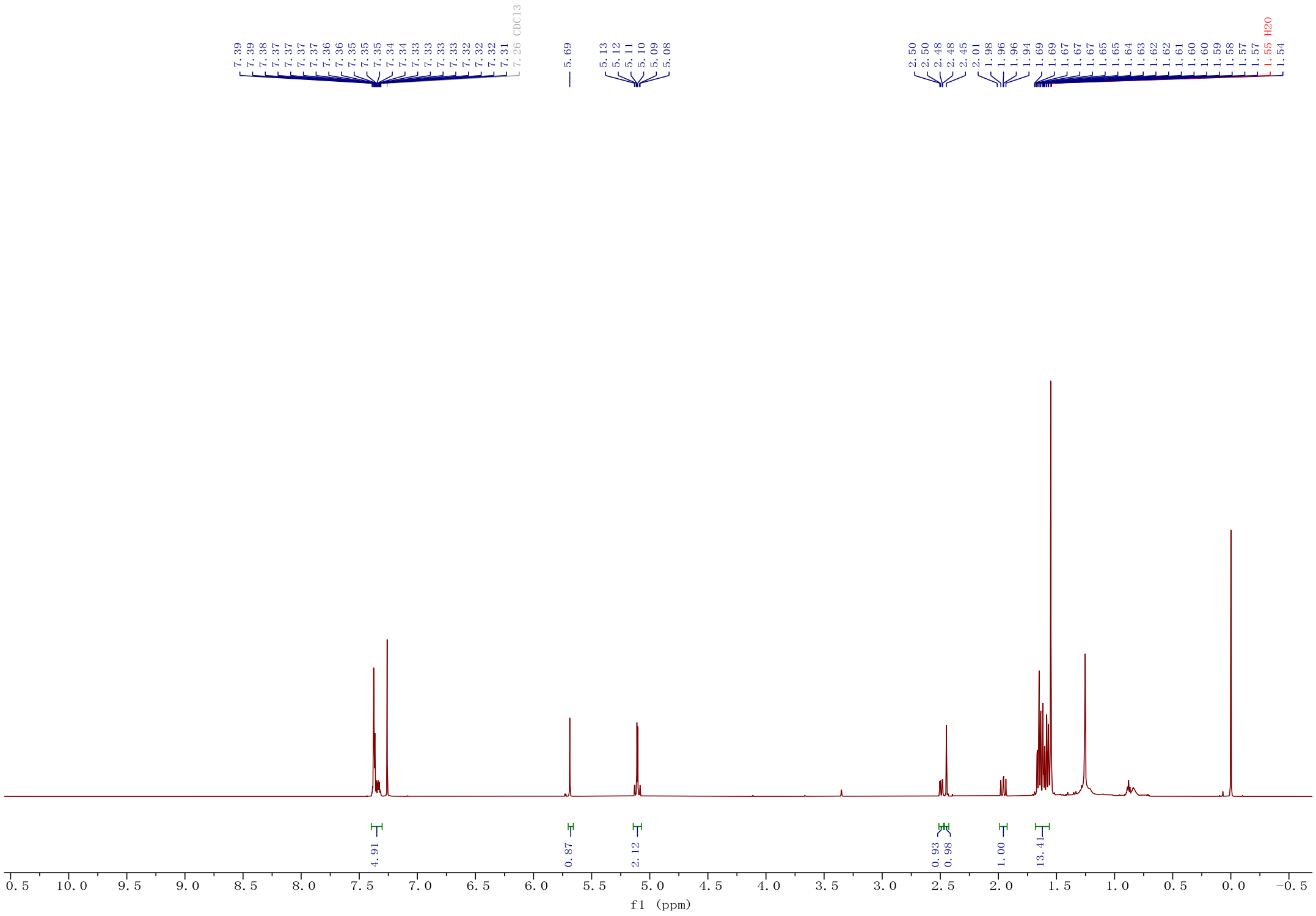

^{13}C NMR (151 MHz, CDCl$_3$)

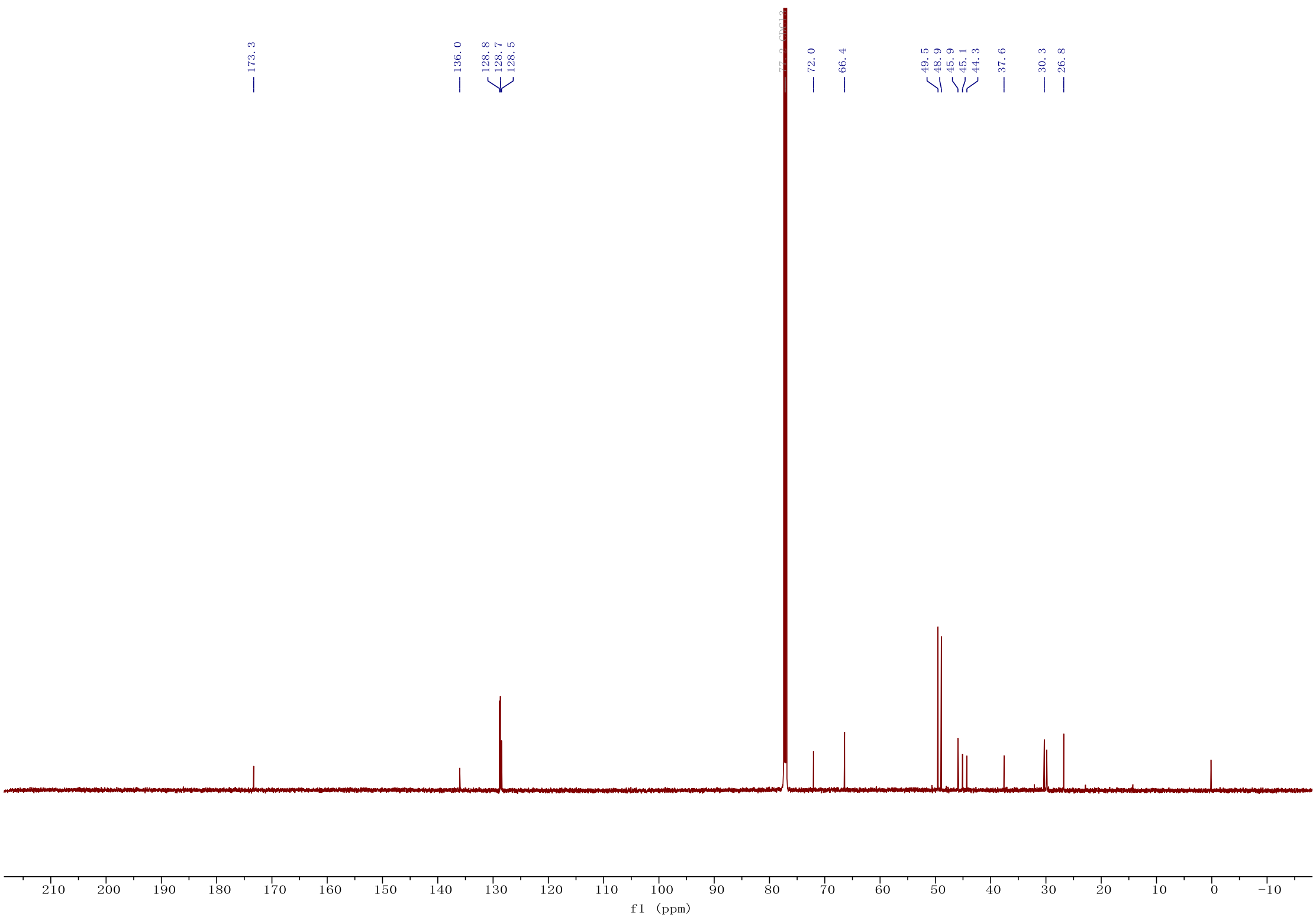

2-(bicyclo[1.1.1]pentan-1-yl)-3-(3-(dichloromethyl)bicyclo[1.1.1]pentan-1-yl)propanenitrile (3ab)

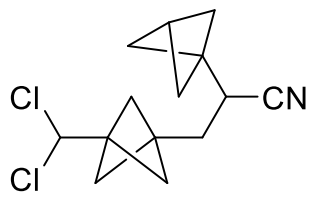

^{1}H NMR (600 MHz, CDCl$_3$)

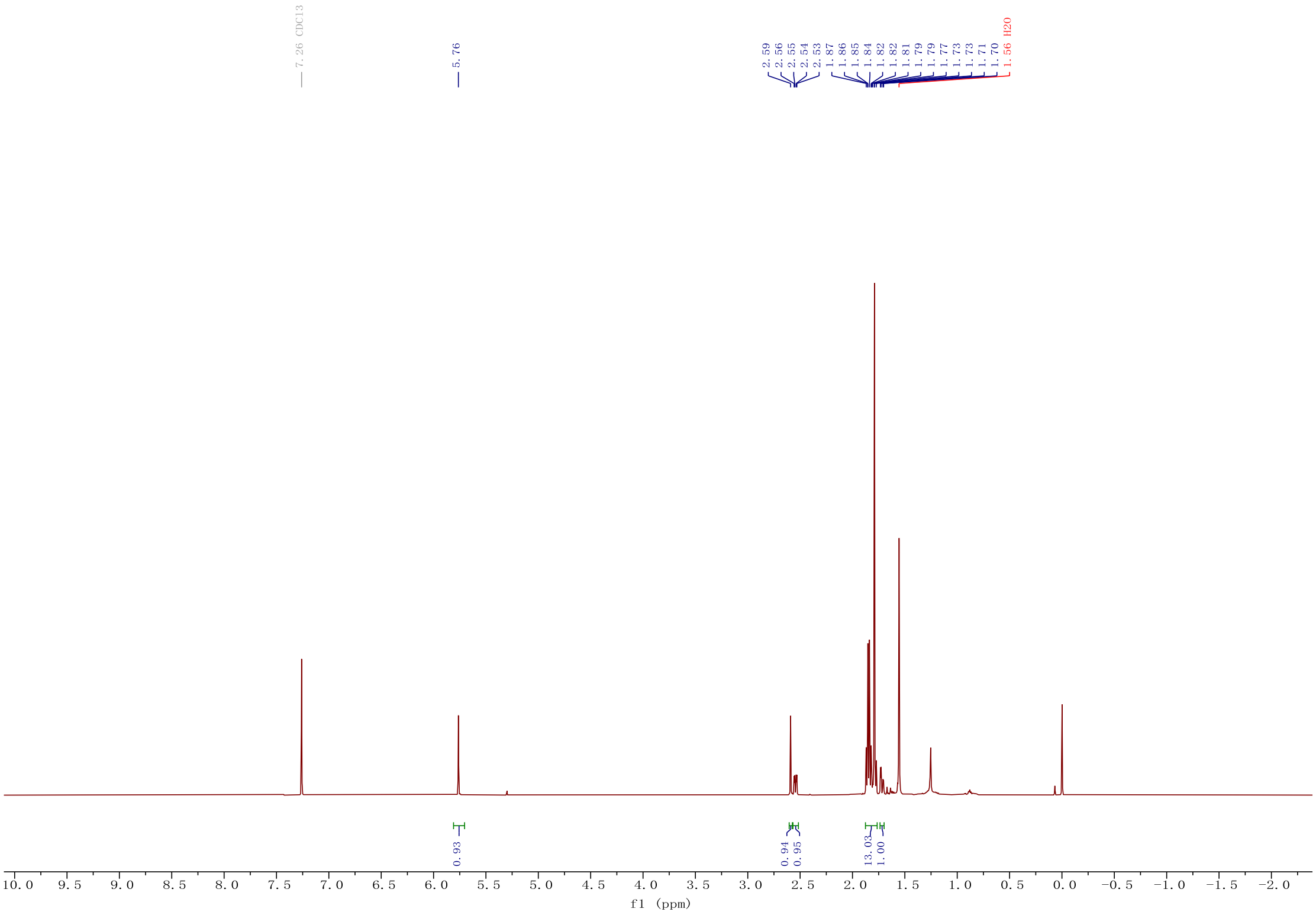

^{13}C NMR (151 MHz, CDCl$_3$)

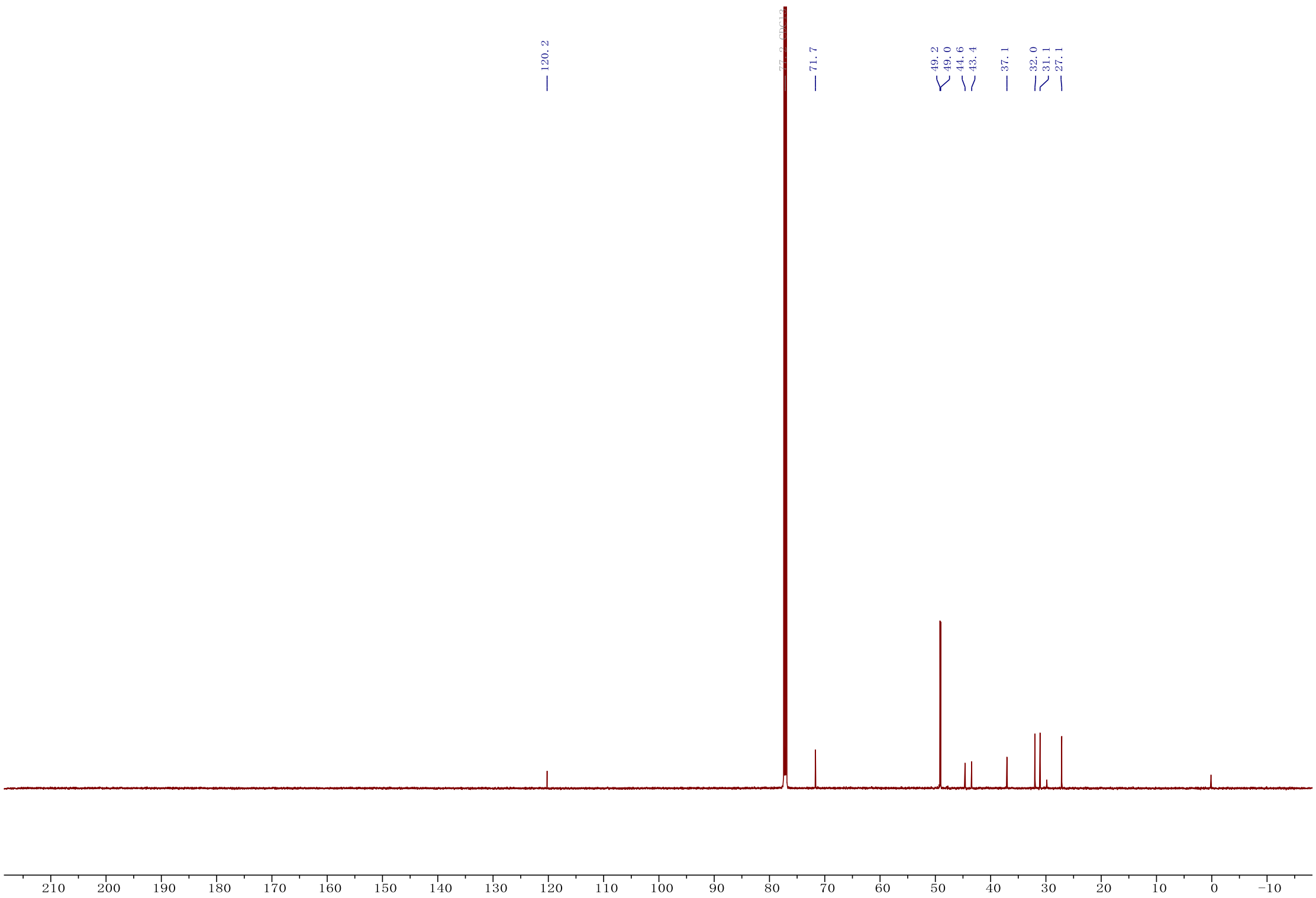

1-(2-(bicyclo[1.1.1]pentan-1-yl)-2-(phenylsulfonyl)ethyl)-3-(dichloromethyl)bicyclo[1.1.1]pentane (3ac)

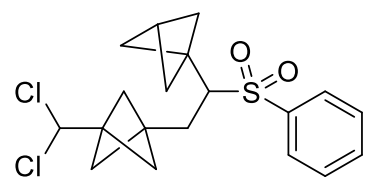

^{1}H NMR (600 MHz, CDCl$_3$)

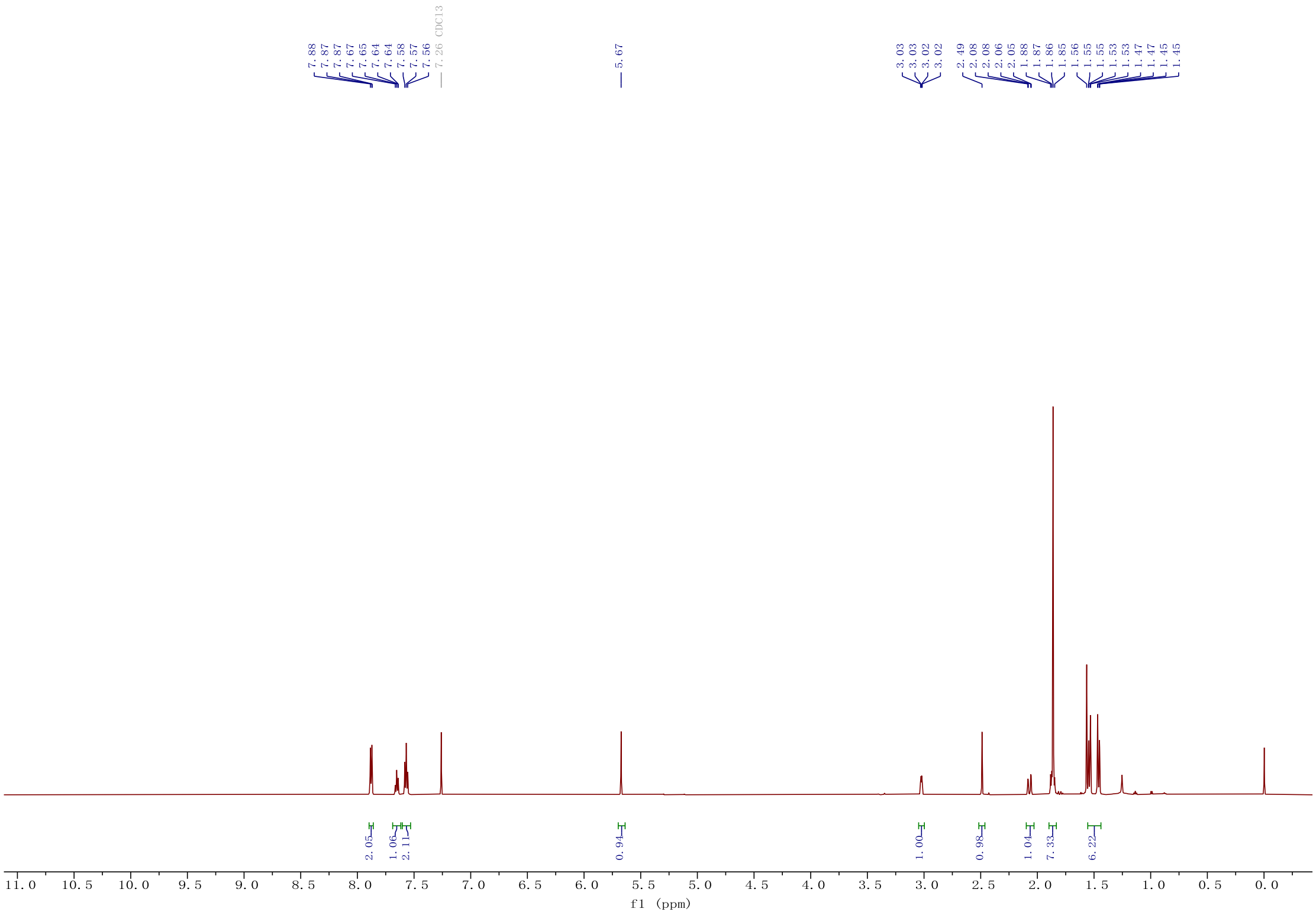

^{13}C NMR (151 MHz, CDCl$_3$)

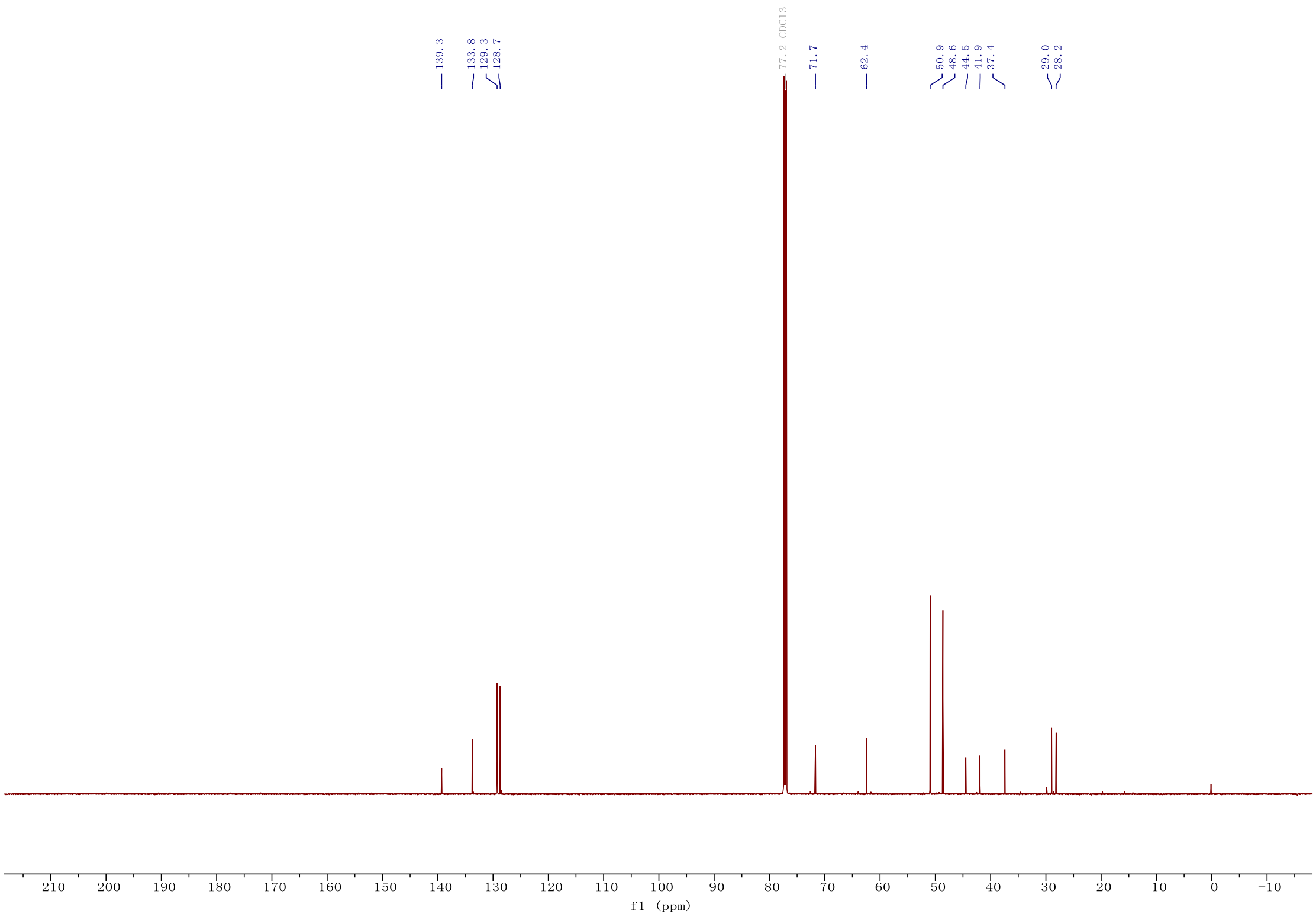

2-(bicyclo[1.1.1]pentan-1-yl)-*N*-phenyl-3-(3-(trichloromethyl)bicyclo[1.1.1]pentan-1-yl)propenamide (4a)

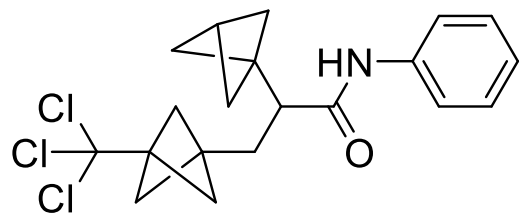

^{1}H NMR (600 MHz, $CDCl_3$)

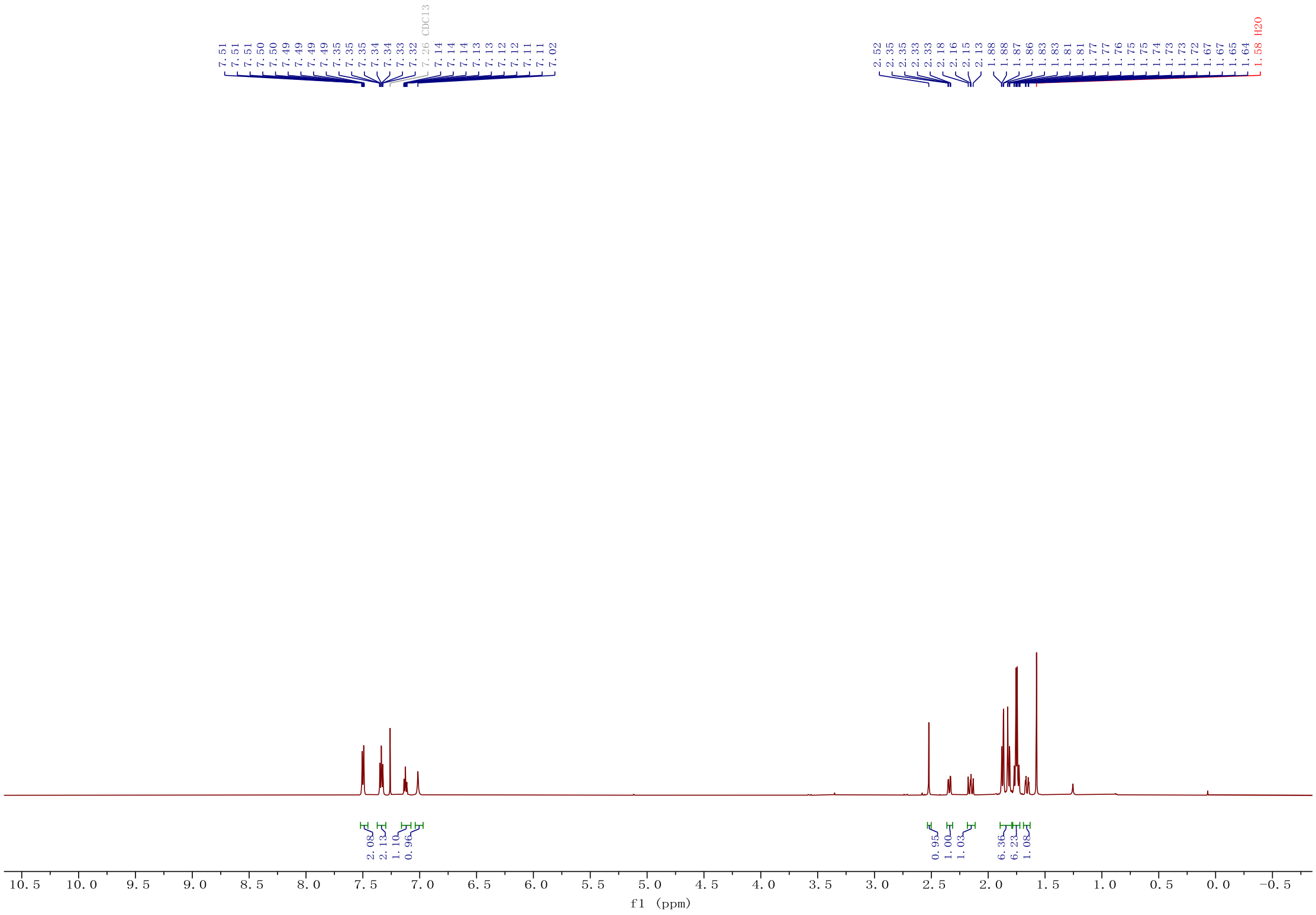

^{13}C NMR (151 MHz, $CDCl_3$)

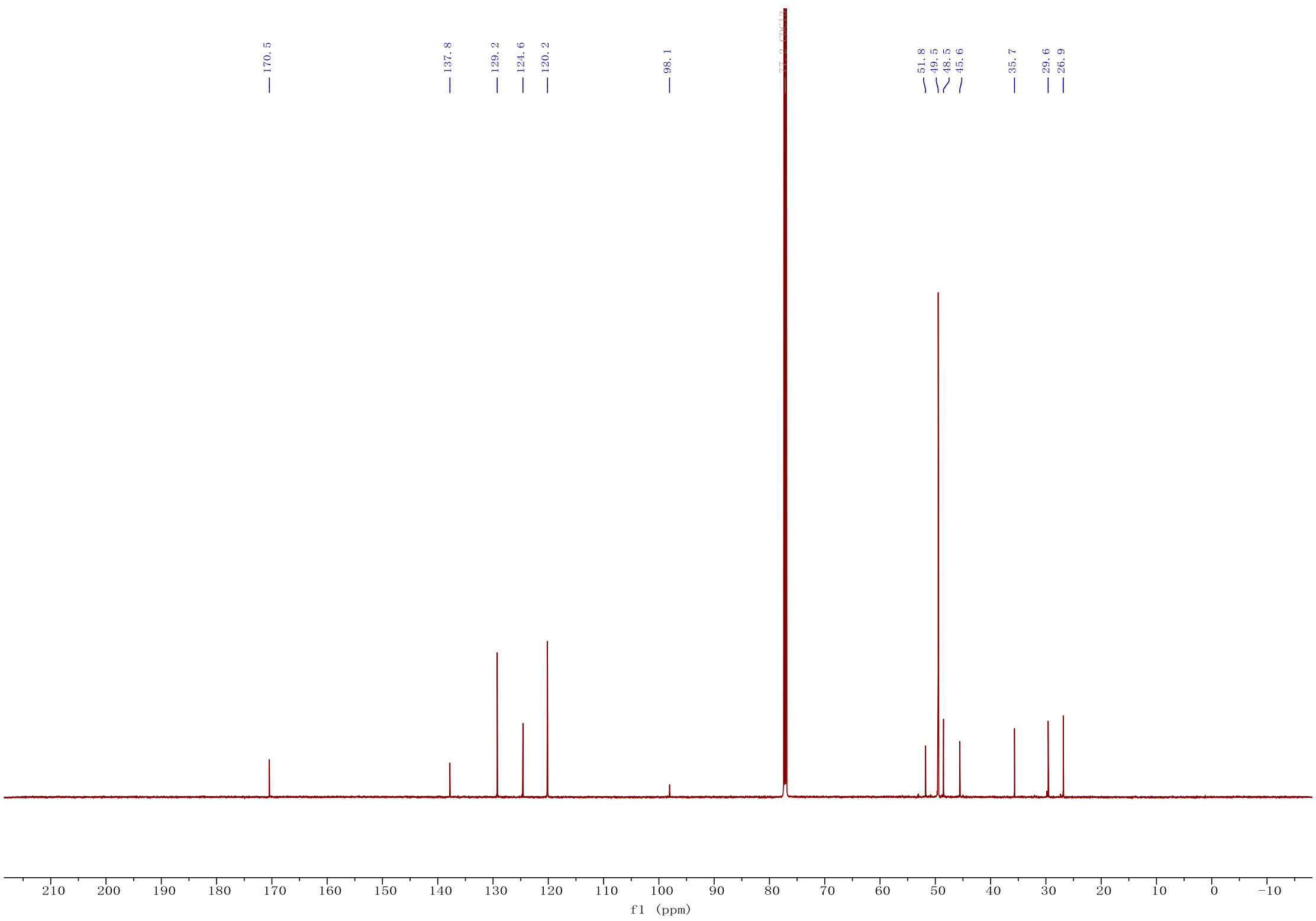

2-(bicyclo[1.1.1]pentan-1-yl)-*N*-(4-fluorophenyl)-3-(3-(trichloromethyl)bicyclo[1.1.1]pentan-1-yl)propenamide (4b)

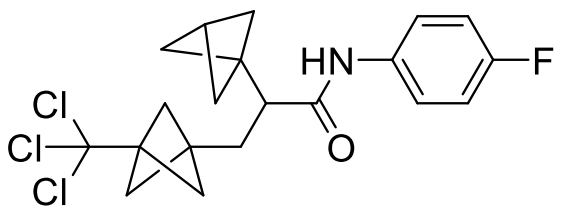

^{1}H NMR (600 MHz, CDCl3)

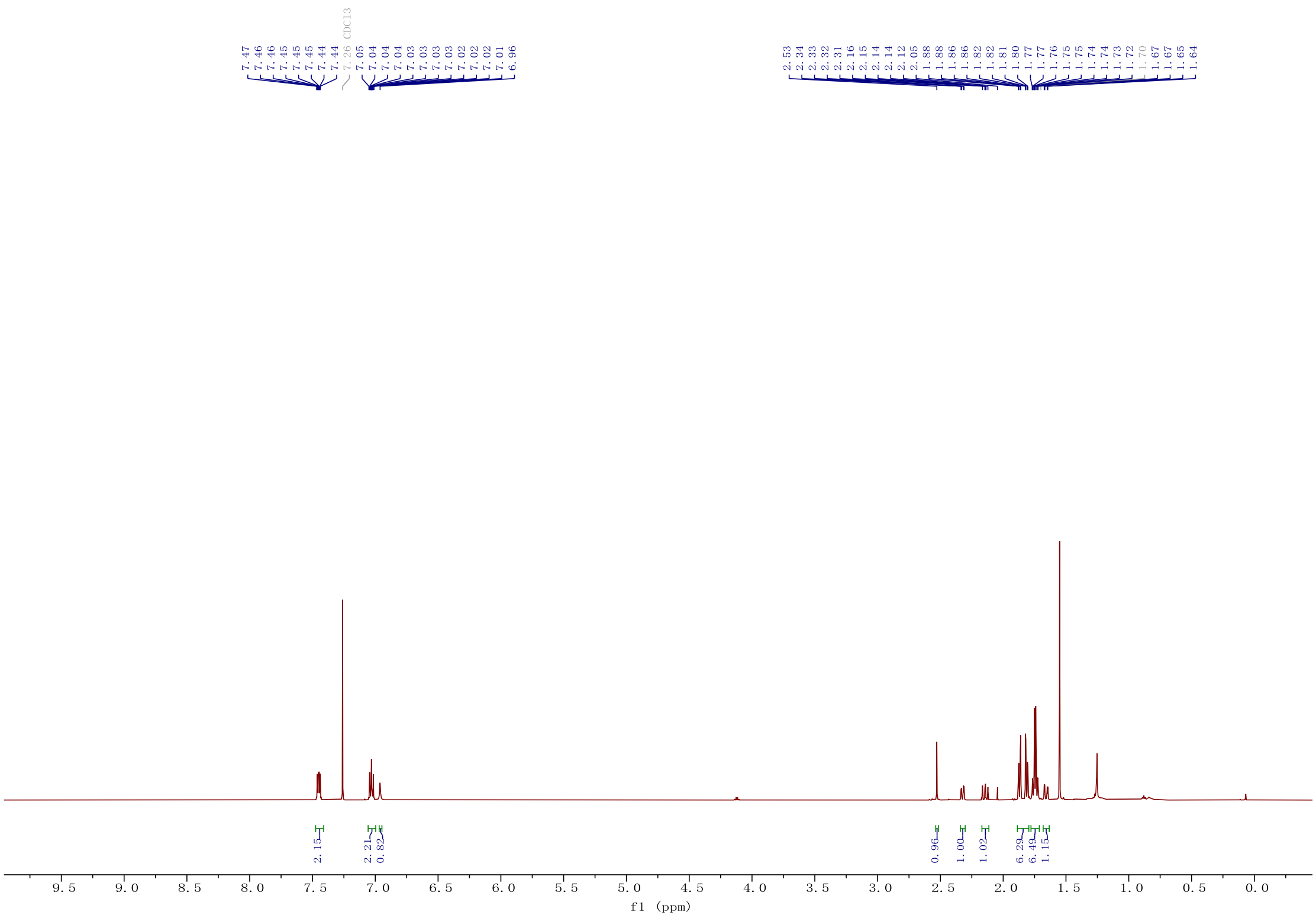

^{13}C NMR (151 MHz, CDCl3)

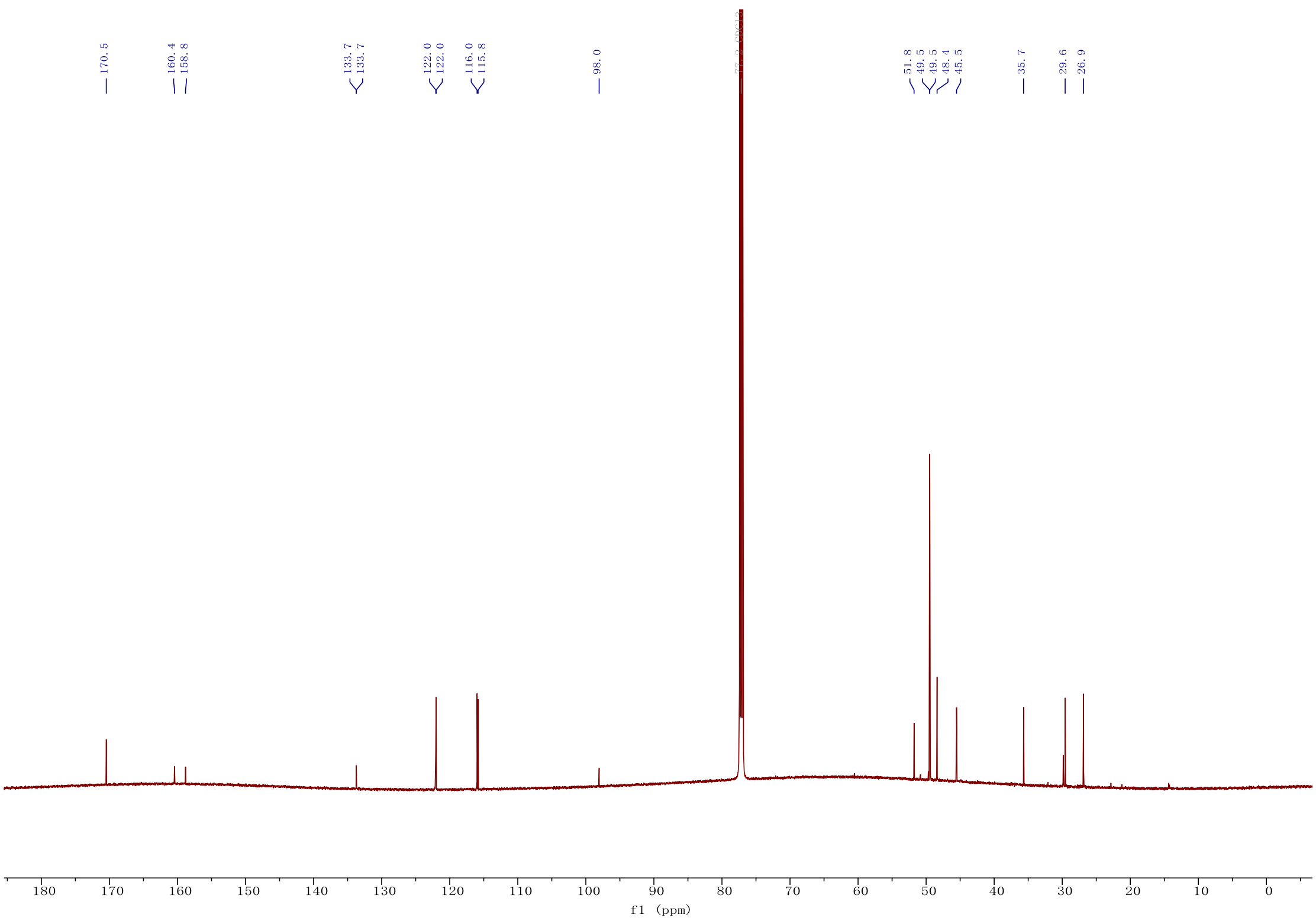

2-(bicyclo[1.1.1]pentan-1-yl)-*N*-(2-bromophenyl)-3-(3-(trichloromethyl)bicyclo[1.1.1]pentan-1-yl)propenamide (4c)

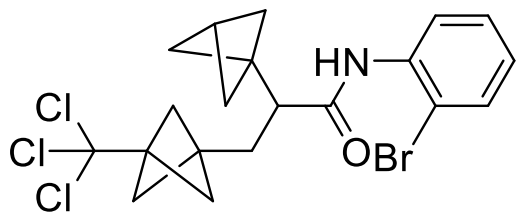

^{1}H NMR (600 MHz, CDCl$_3$)

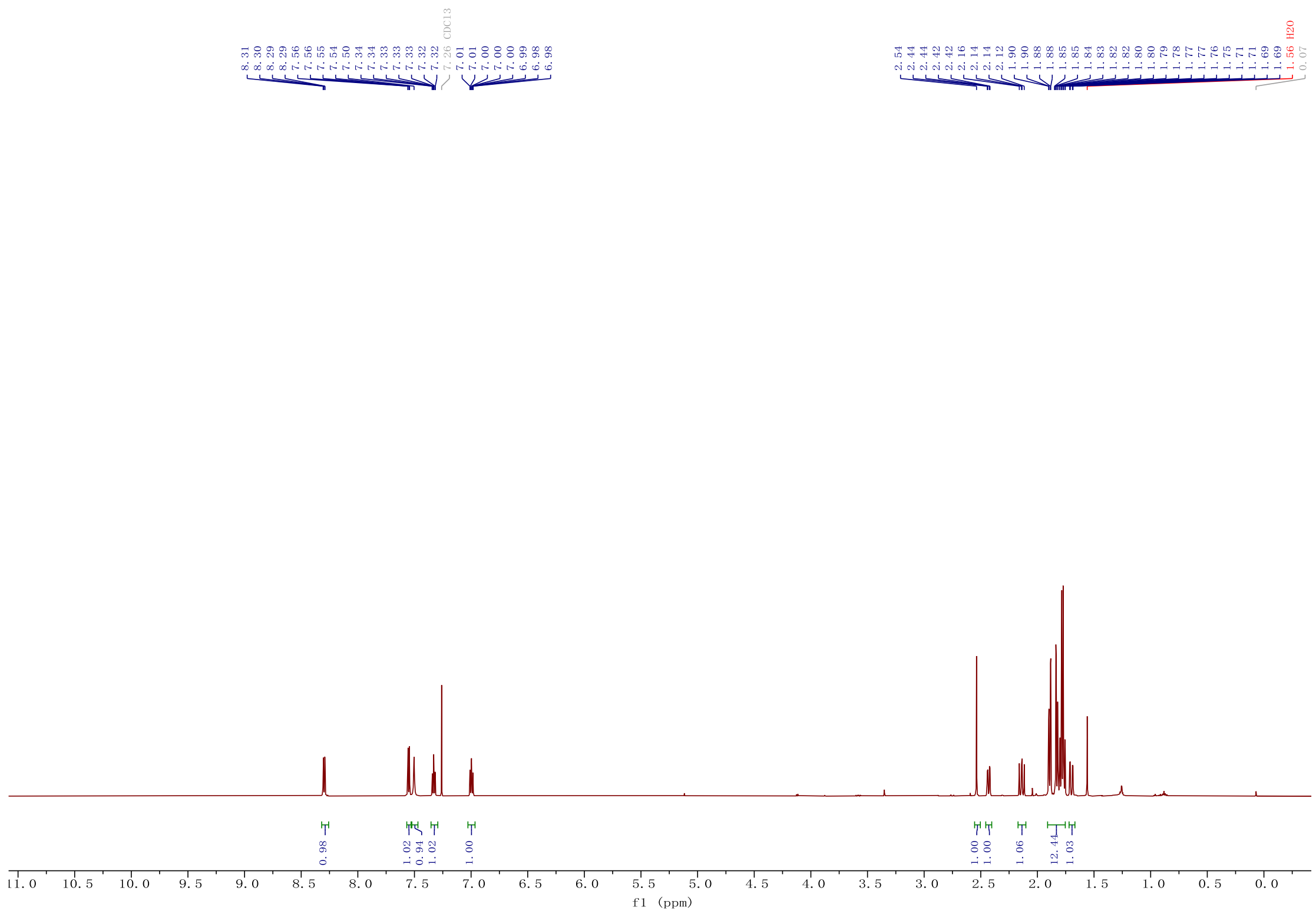

^{13}C NMR (151 MHz, CDCl$_3$)

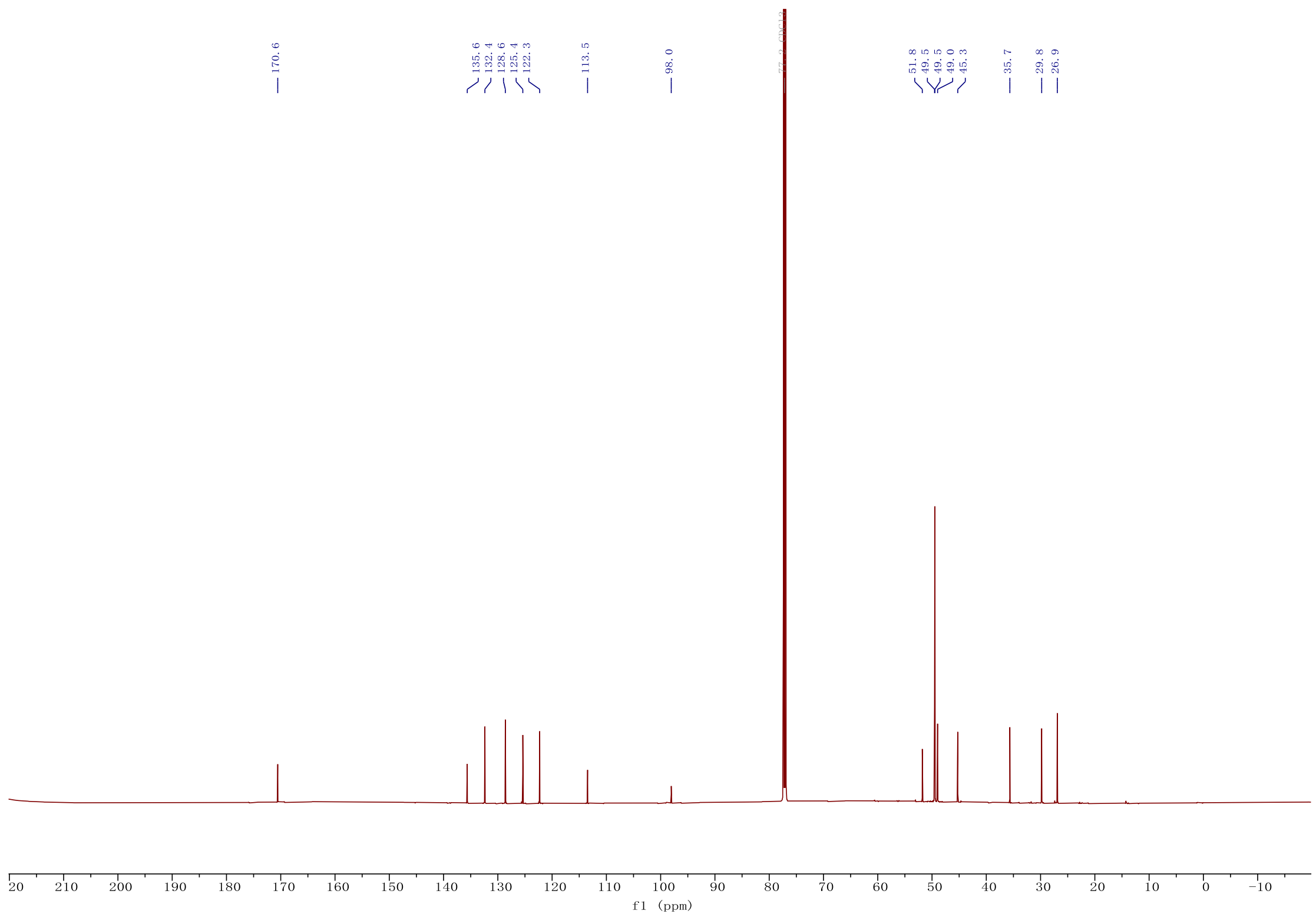

2-(bicyclo[1.1.1]pentan-1-yl)-*N*-(3,5-dimethylphenyl)-3-(3-(trichloromethyl)bicyclo[1.1.1]pentan-1-yl)propenamide (4d)

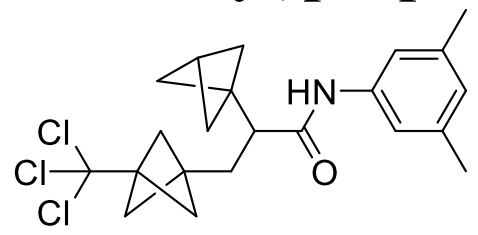

^{1}H NMR (600 MHz, CDCl$_3$)

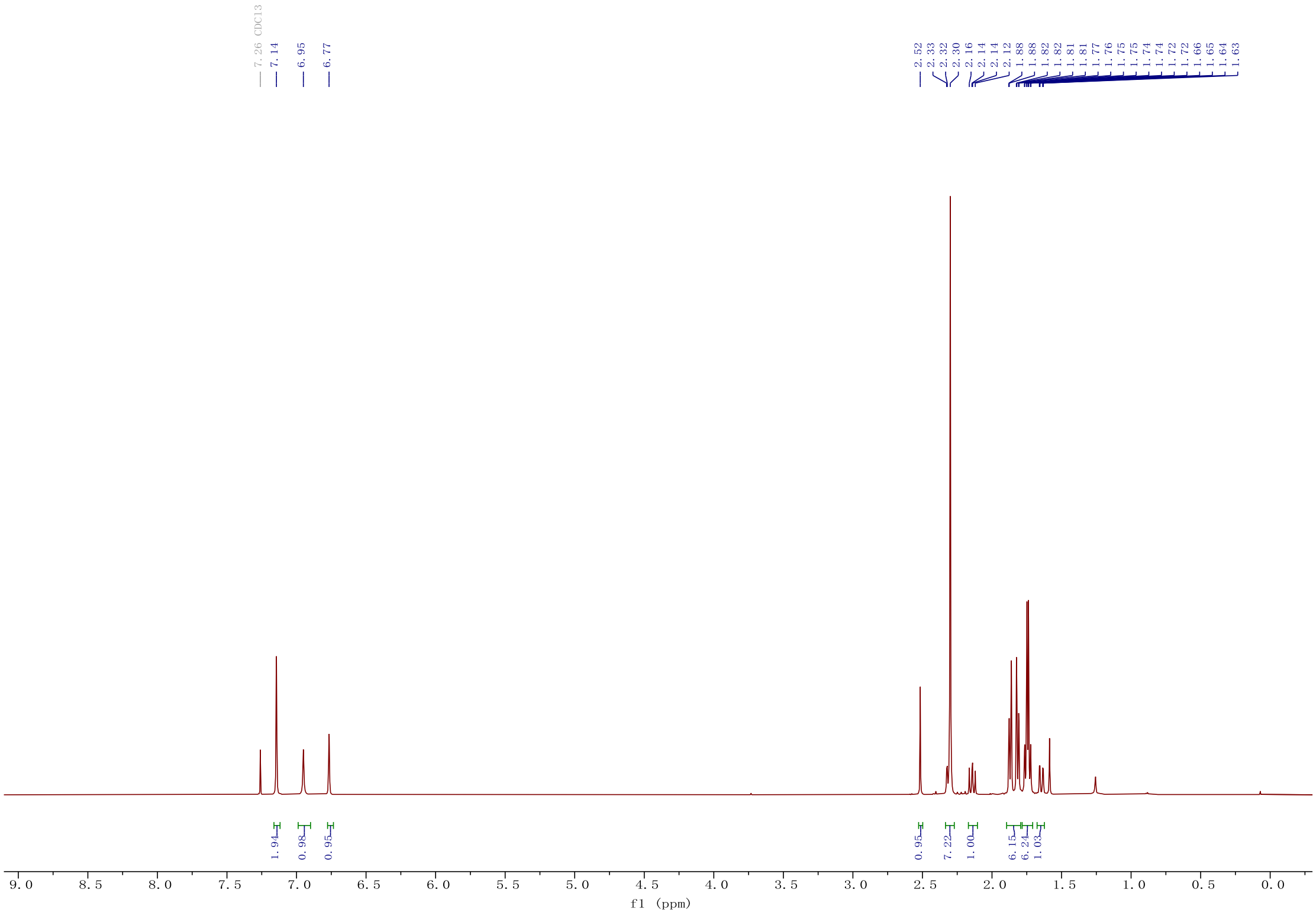

^{13}C NMR (151 MHz, CDCl$_3$)

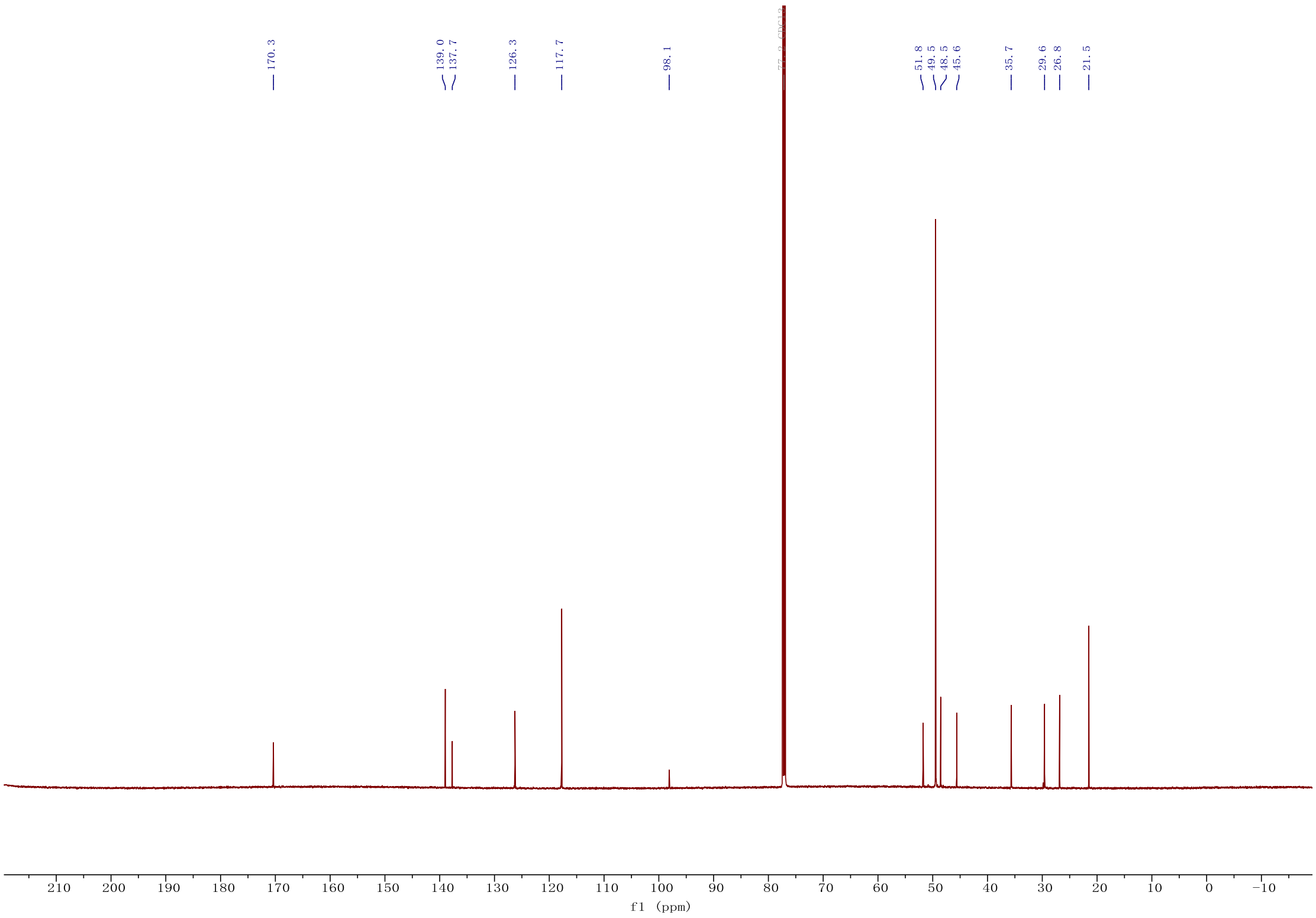

2-(bicyclo[1.1.1]pentan-1-yl)-*N*-(4-methoxyphenyl)-3-(3-(trichloromethyl)bicyclo[1.1.1]pentan-1-yl)propenamide (4e)

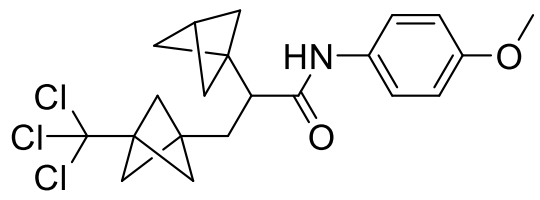

^{1}H NMR (600 MHz, $CDCl_3$)

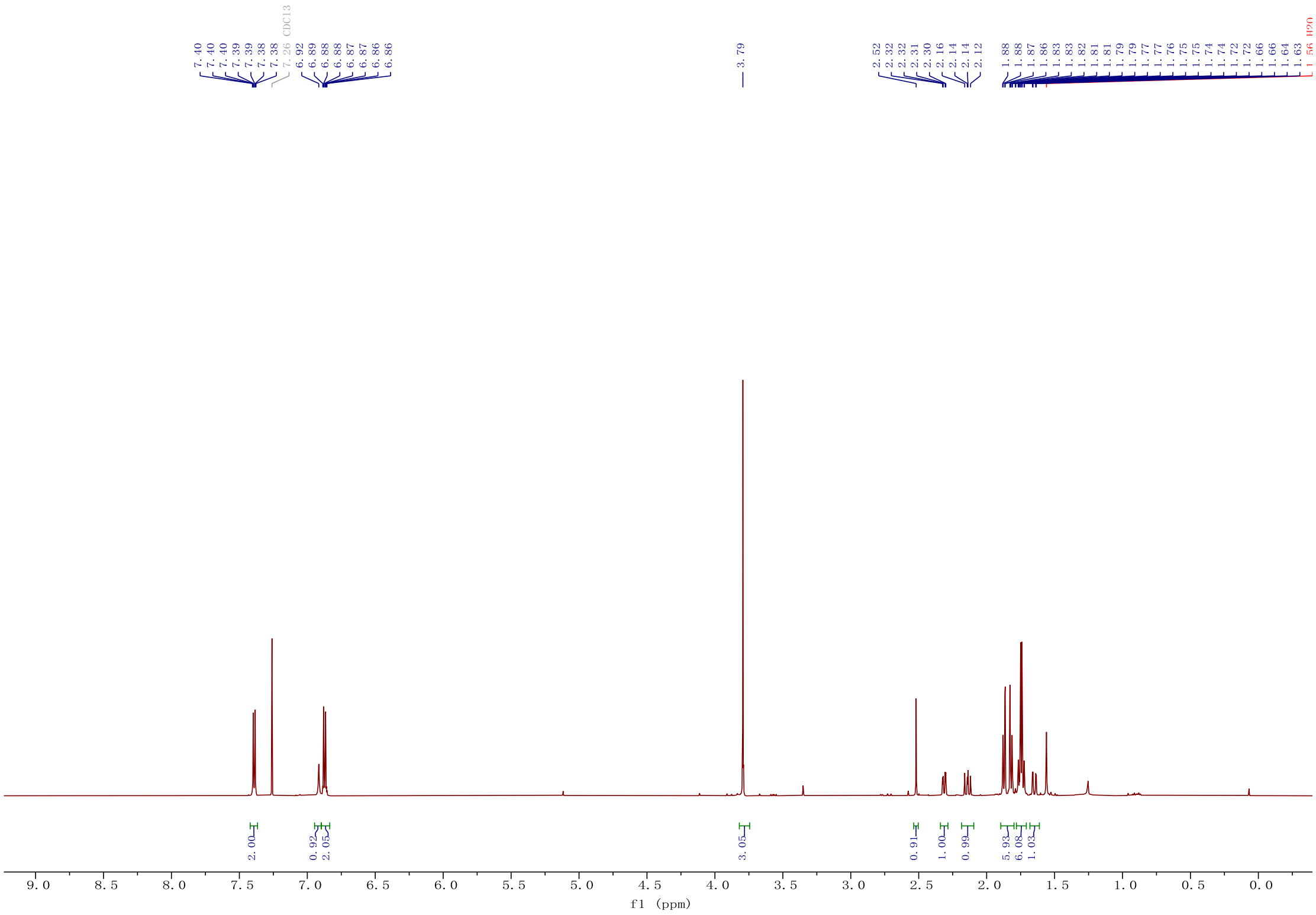

^{13}C NMR (151 MHz, $CDCl_3$)

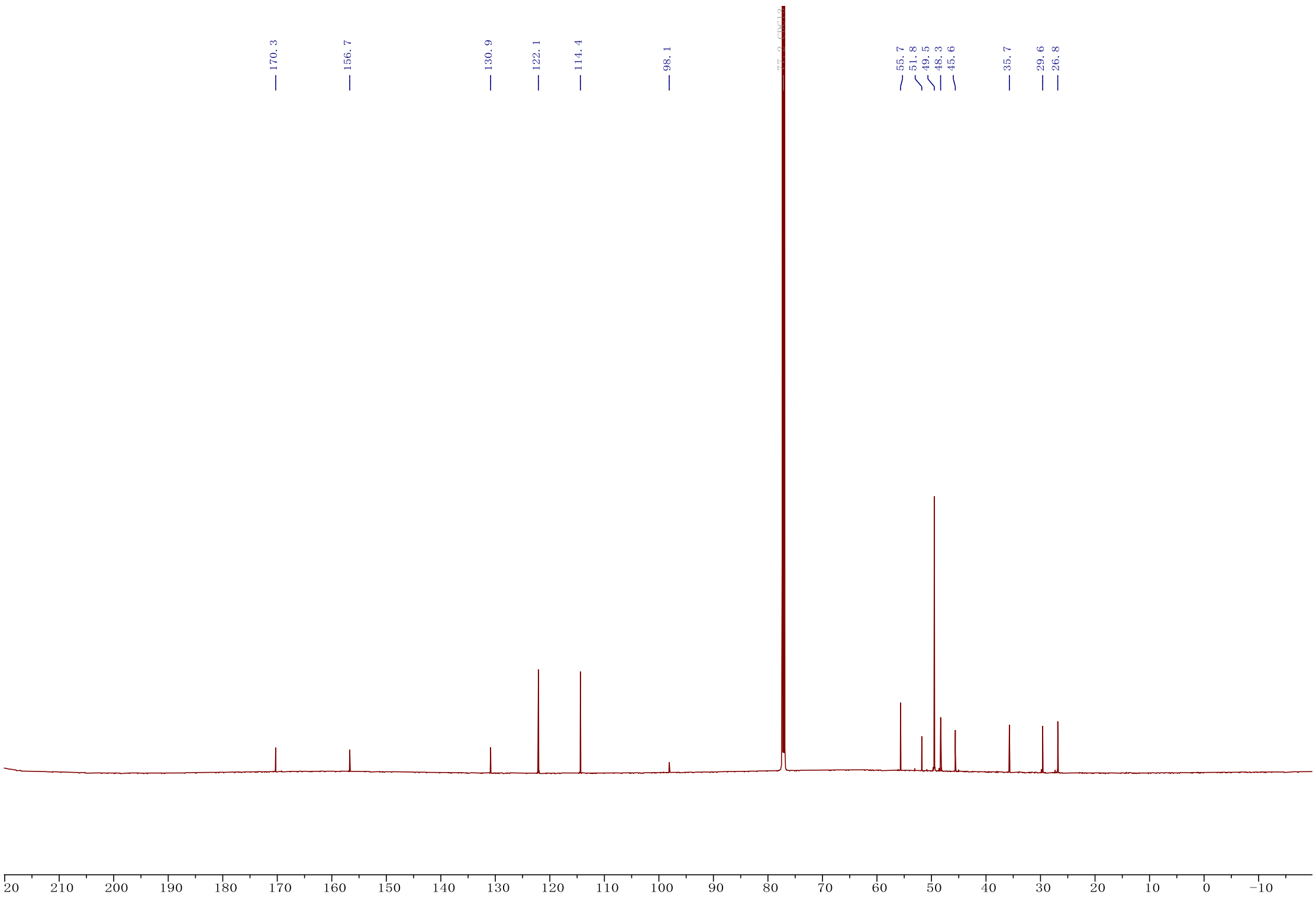

2-(bicyclo[1.1.1]pentan-1-yl)-*N*-(p-tolyl)-3-(3-(trichloromethyl)bicyclo[1.1.1]pentan-1-yl)propenamide (4f)

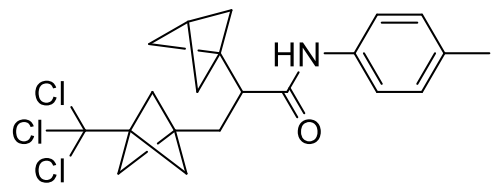

^{1}H NMR (600 MHz, $CDCl_3$)

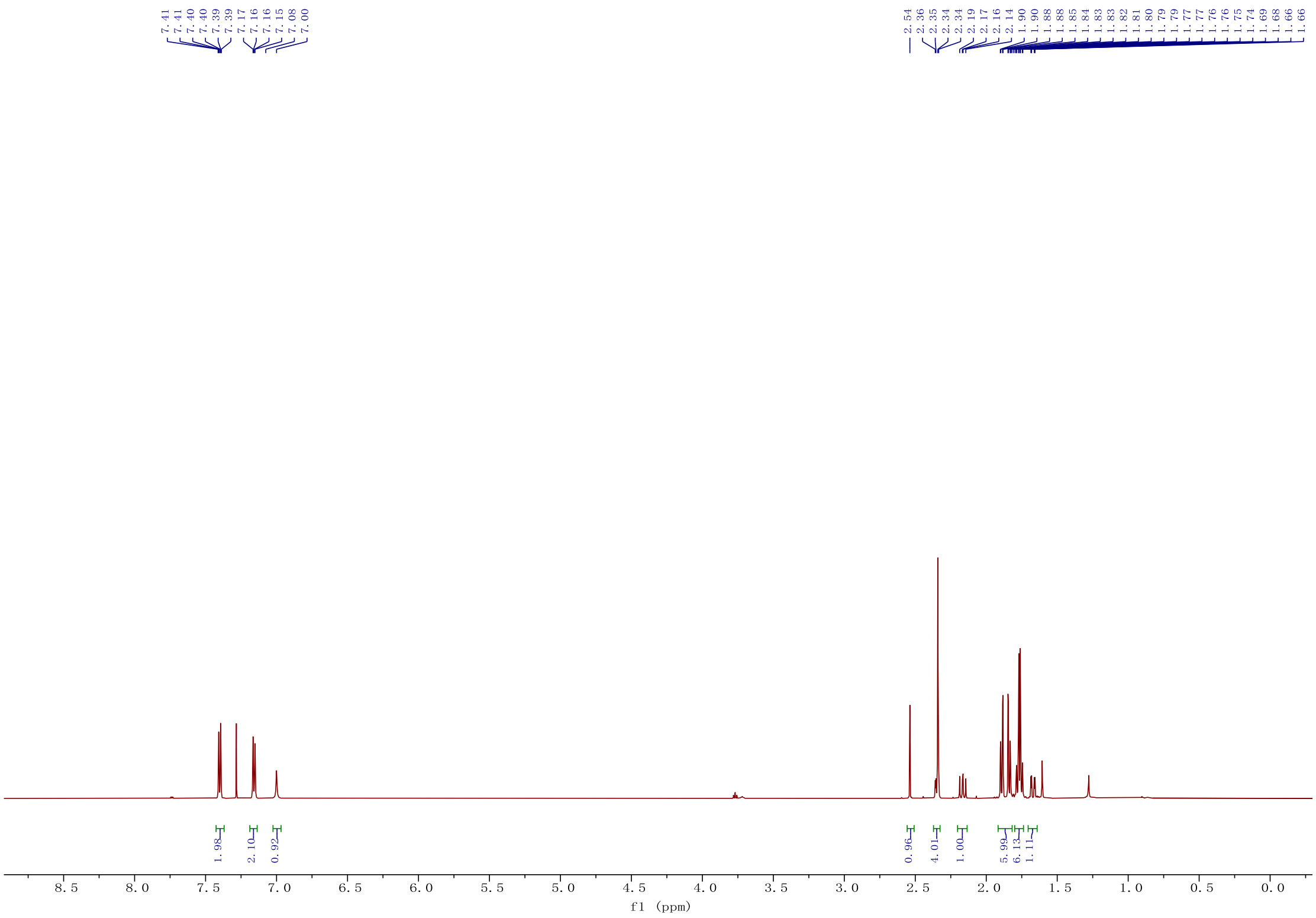

^{13}C NMR (151 MHz, $CDCl_3$)

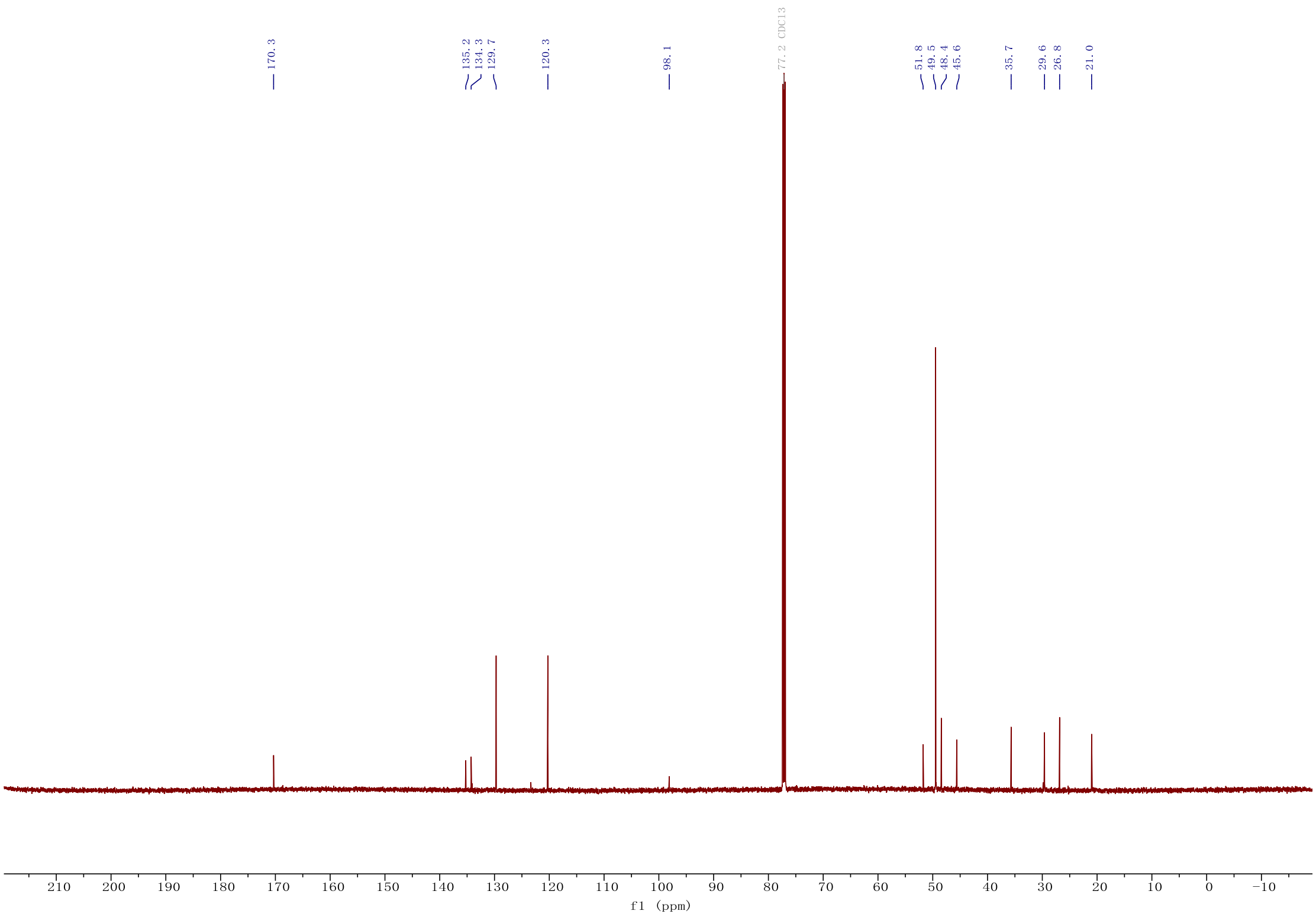

2-(bicyclo[1.1.1]pentan-1-yl)-*N*-(4-phenoxyphenyl)-3-(3-(trichloromethyl)bicyclo[1.1.1]pentan-1-yl)propenamide (4g)

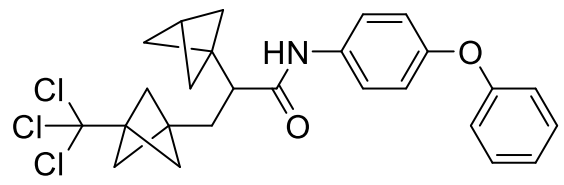

^{1}H NMR (600 MHz, $CDCl_3$)

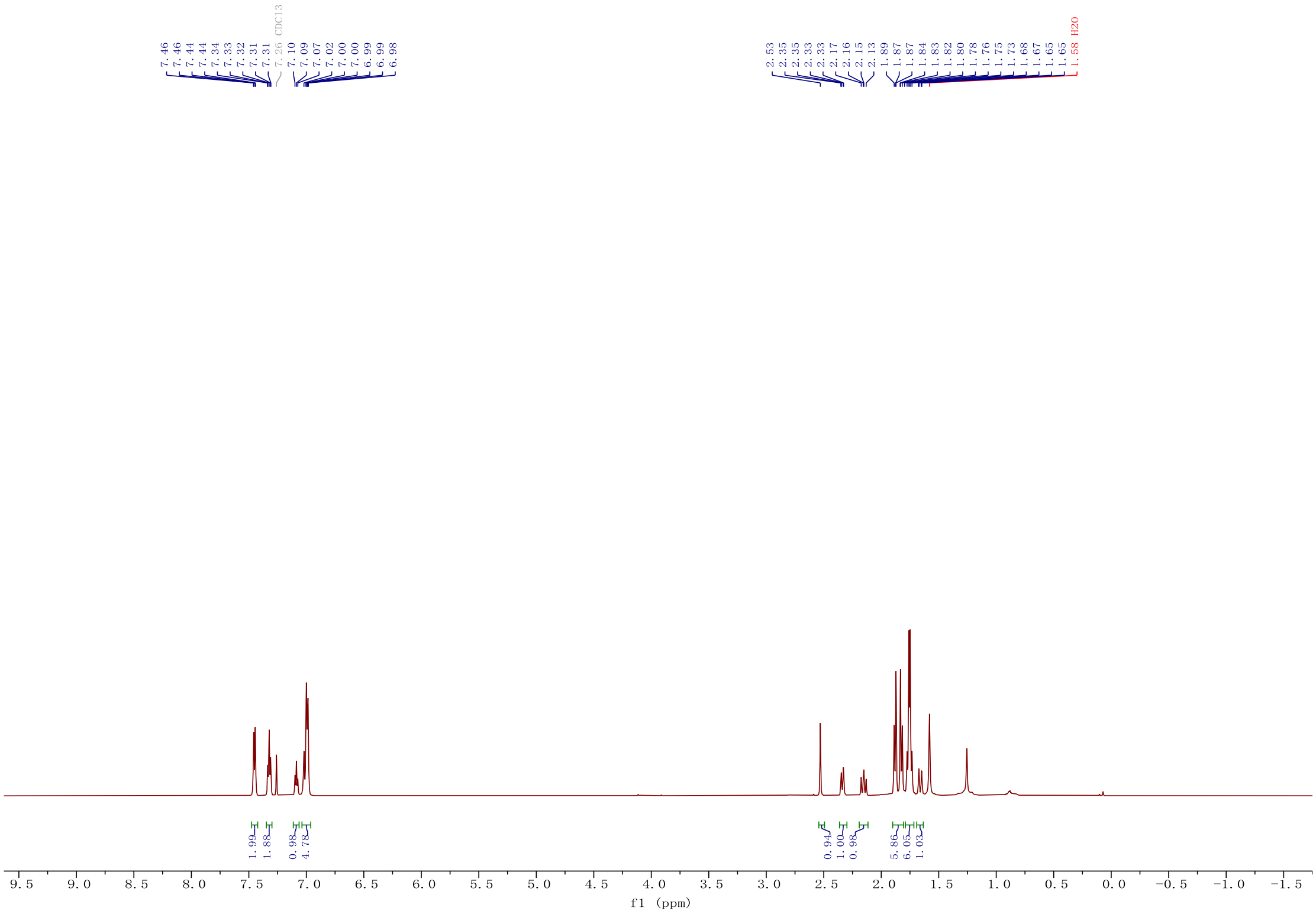

^{13}C NMR (151 MHz, $CDCl_3$)

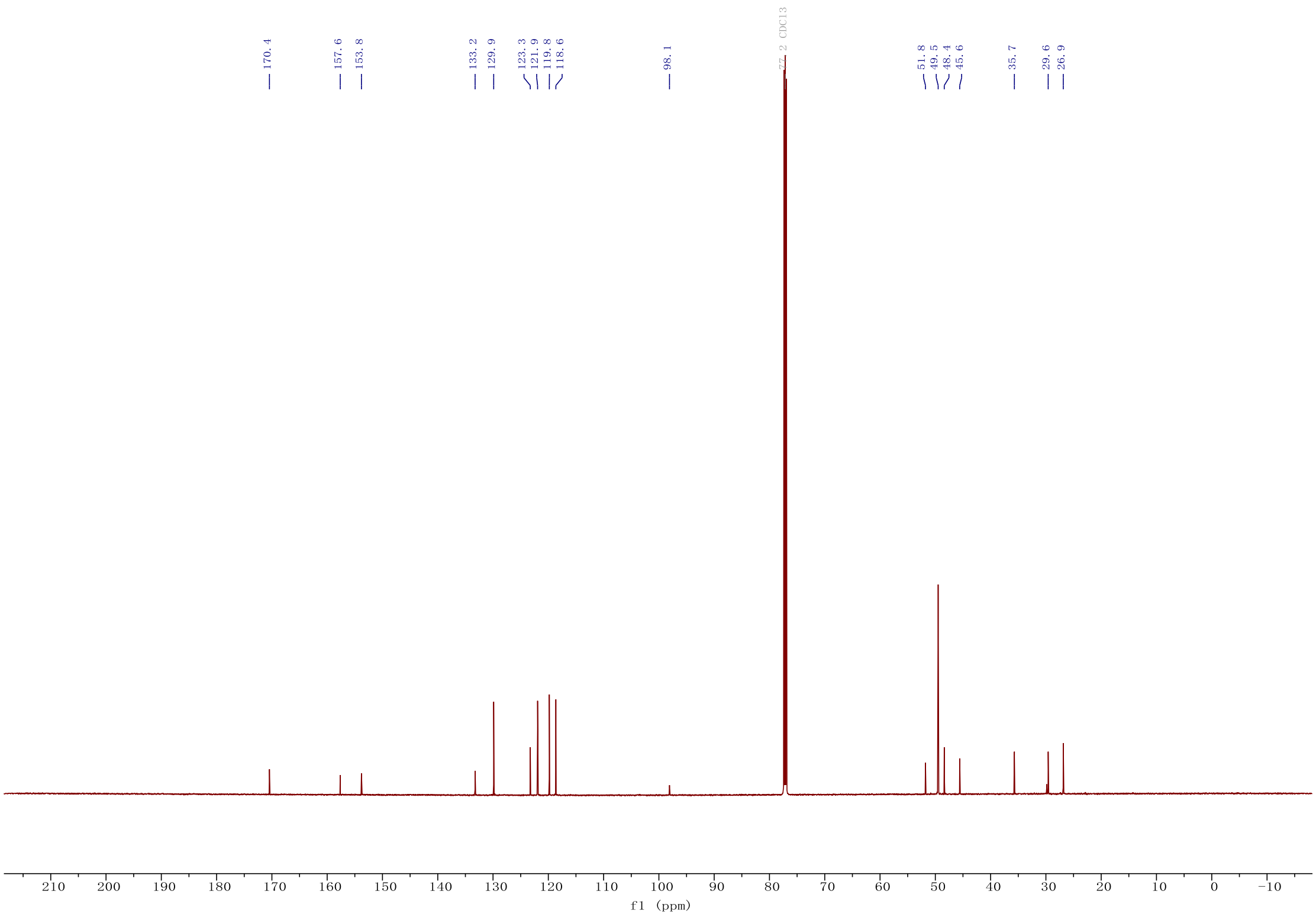

2-(bicyclo[1.1.1]pentan-1-yl)-*N*-cyclopropyl-3-(3-(trichloromethyl)bicyclo[1.1.1]pentan-1-yl)propenamide (4h)

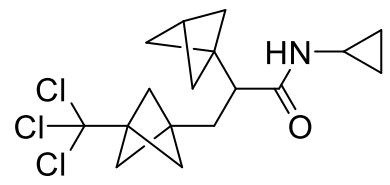

^{1}H NMR (600 MHz, CDCl$_3$)

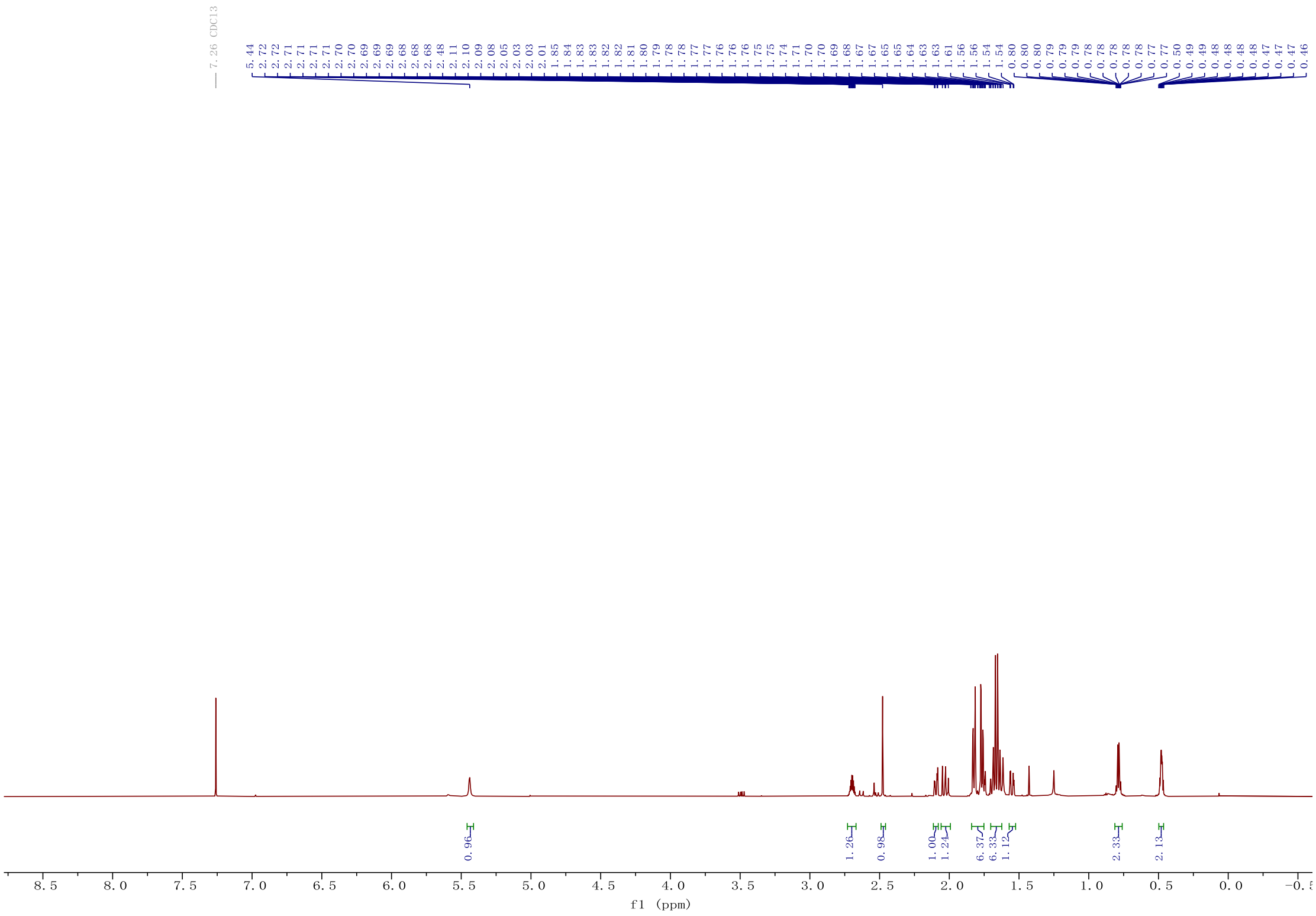

^{13}C NMR (151 MHz, CDCl$_3$)

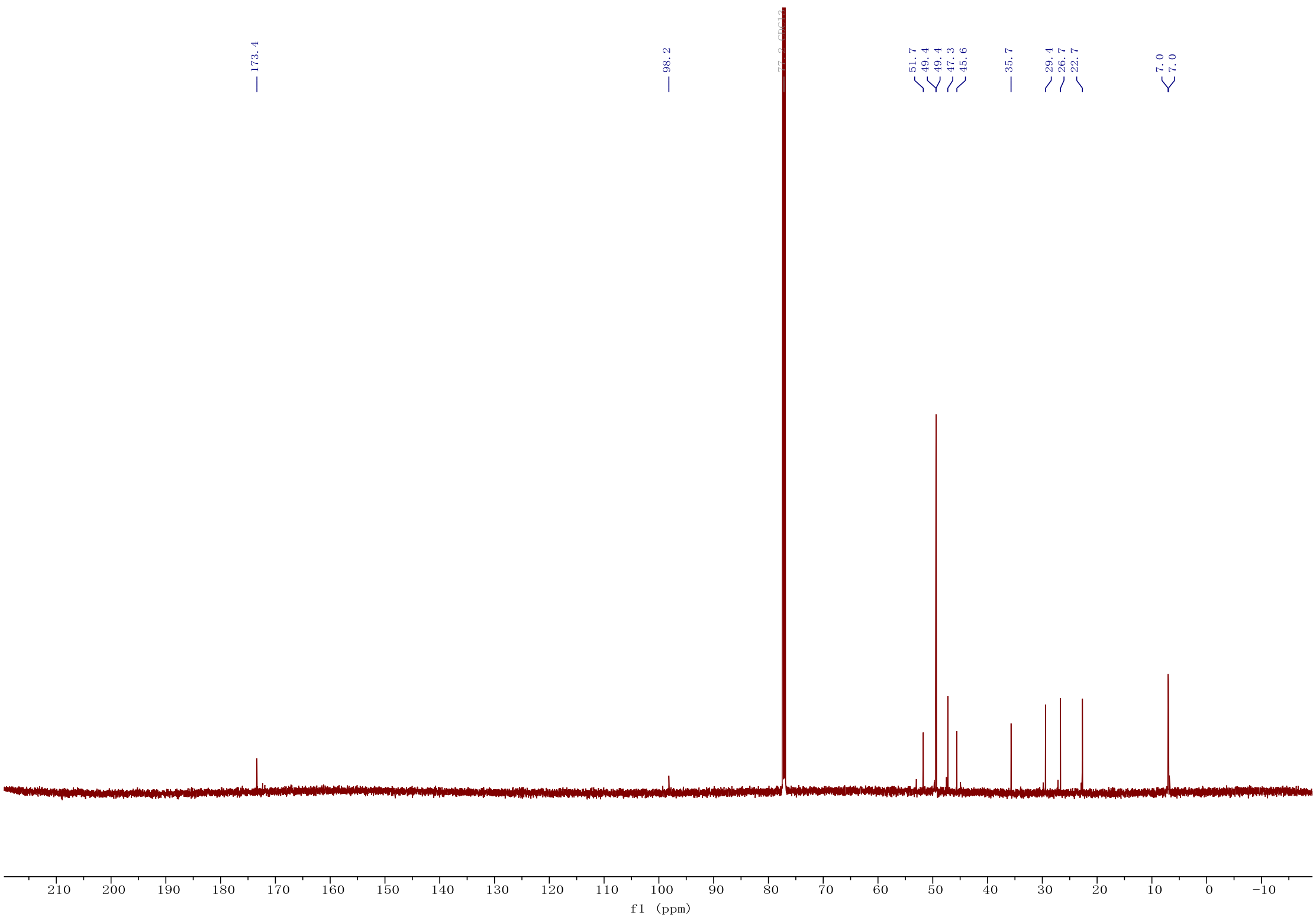

2-(bicyclo[1.1.1]pentan-1-yl)-*N*-cyclohexyl-3-(3-(trichloromethyl)bicyclo[1.1.1]pentan-1-yl)propenamide (4i)

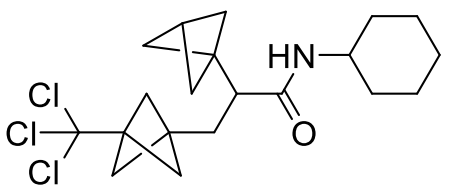

^{1}H NMR (600 MHz, CDCl$_3$)

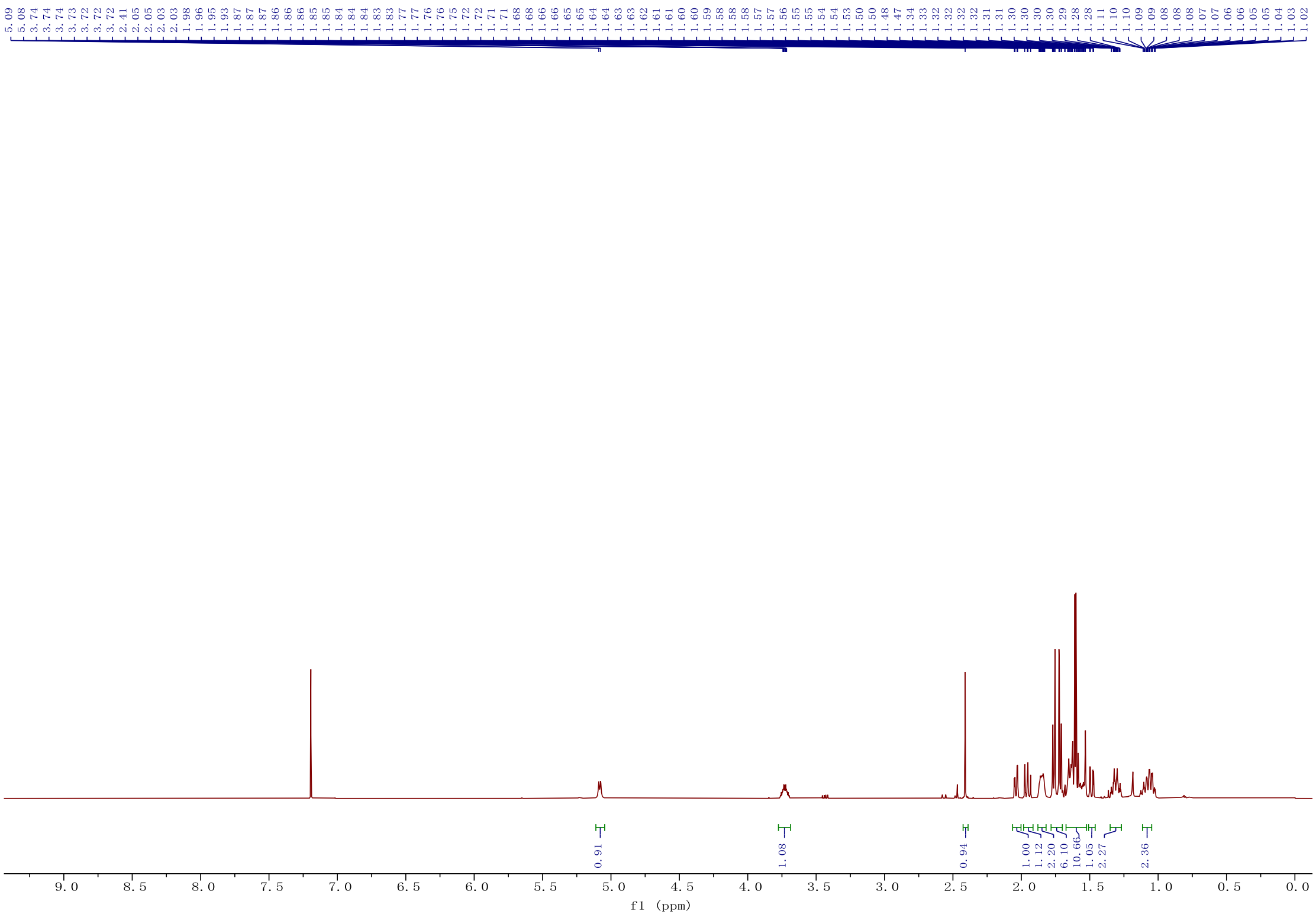

^{13}C NMR (151 MHz, CDCl$_3$)

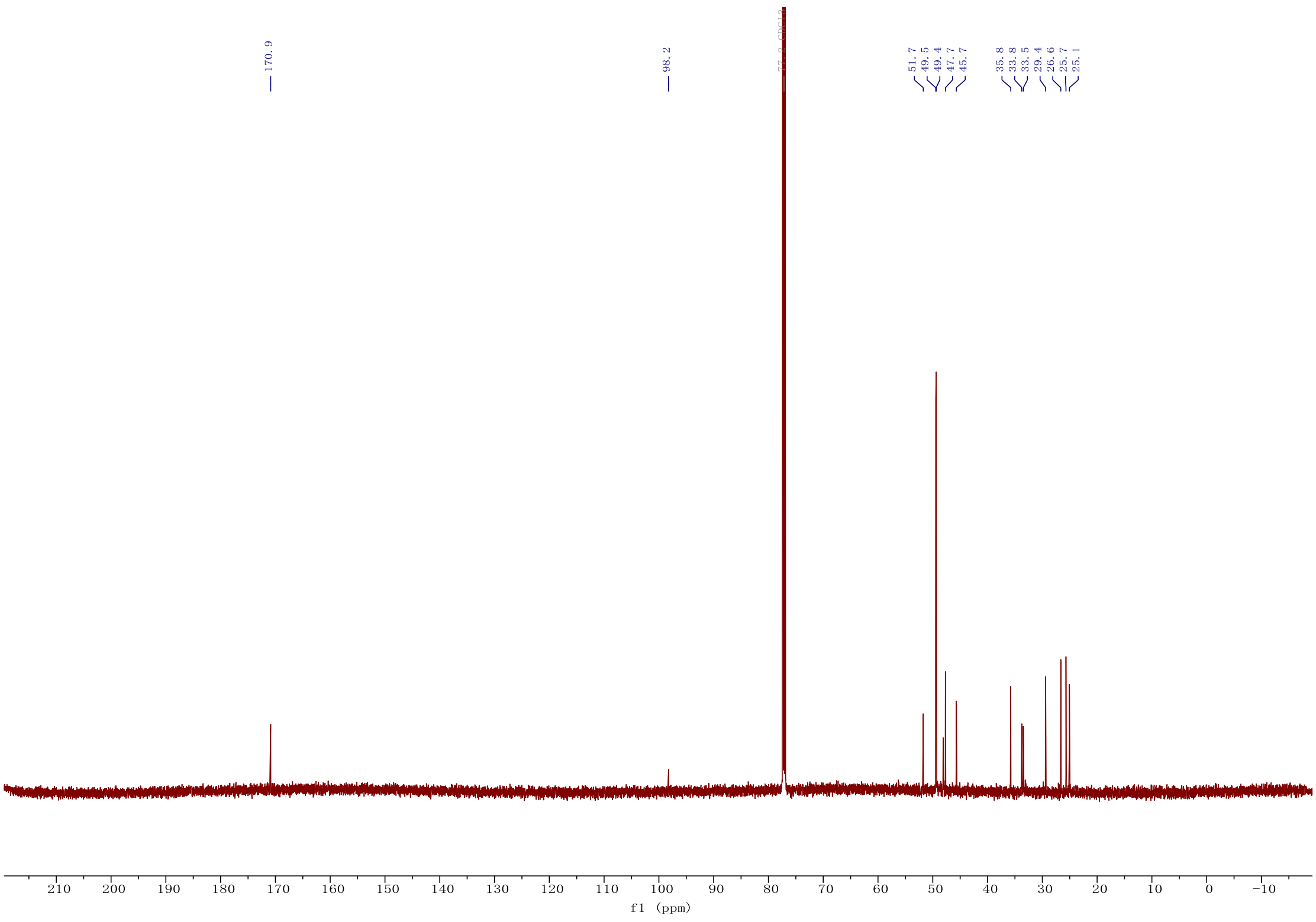

2-(bicyclo[1.1.1]pentan-1-yl)-*N*-(2-methyl-4-oxopentan-2-yl)-3-(3-(trichloromethyl)bicyclo[1.1.1]pentan-1-yl)propenamide (4j)

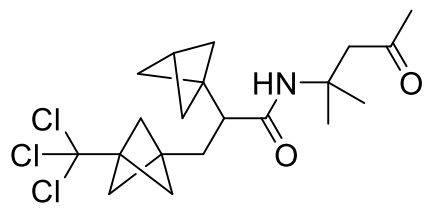

^{1}H NMR (600 MHz, $CDCl_3$)

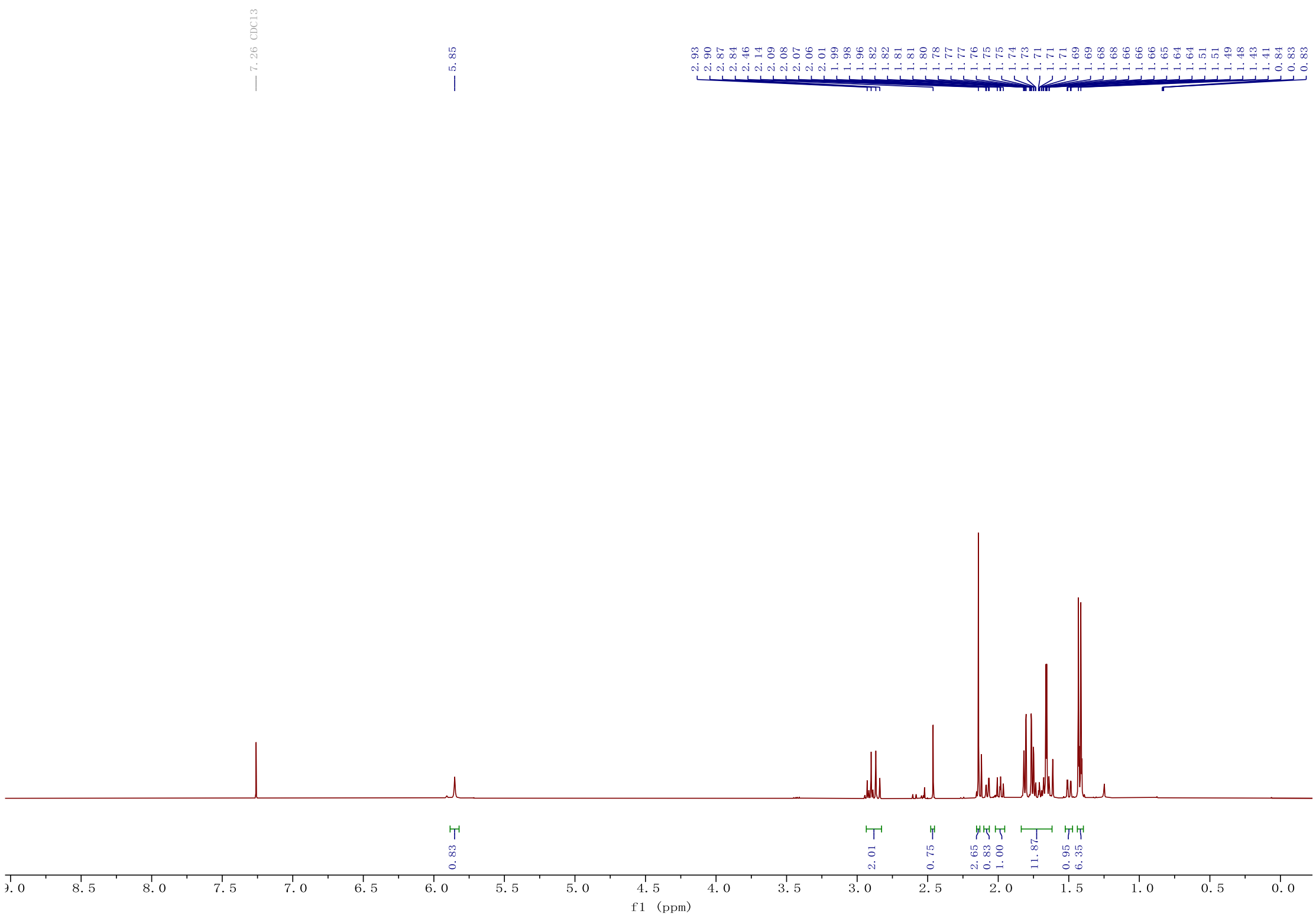

^{13}C NMR (151 MHz, $CDCl_3$)

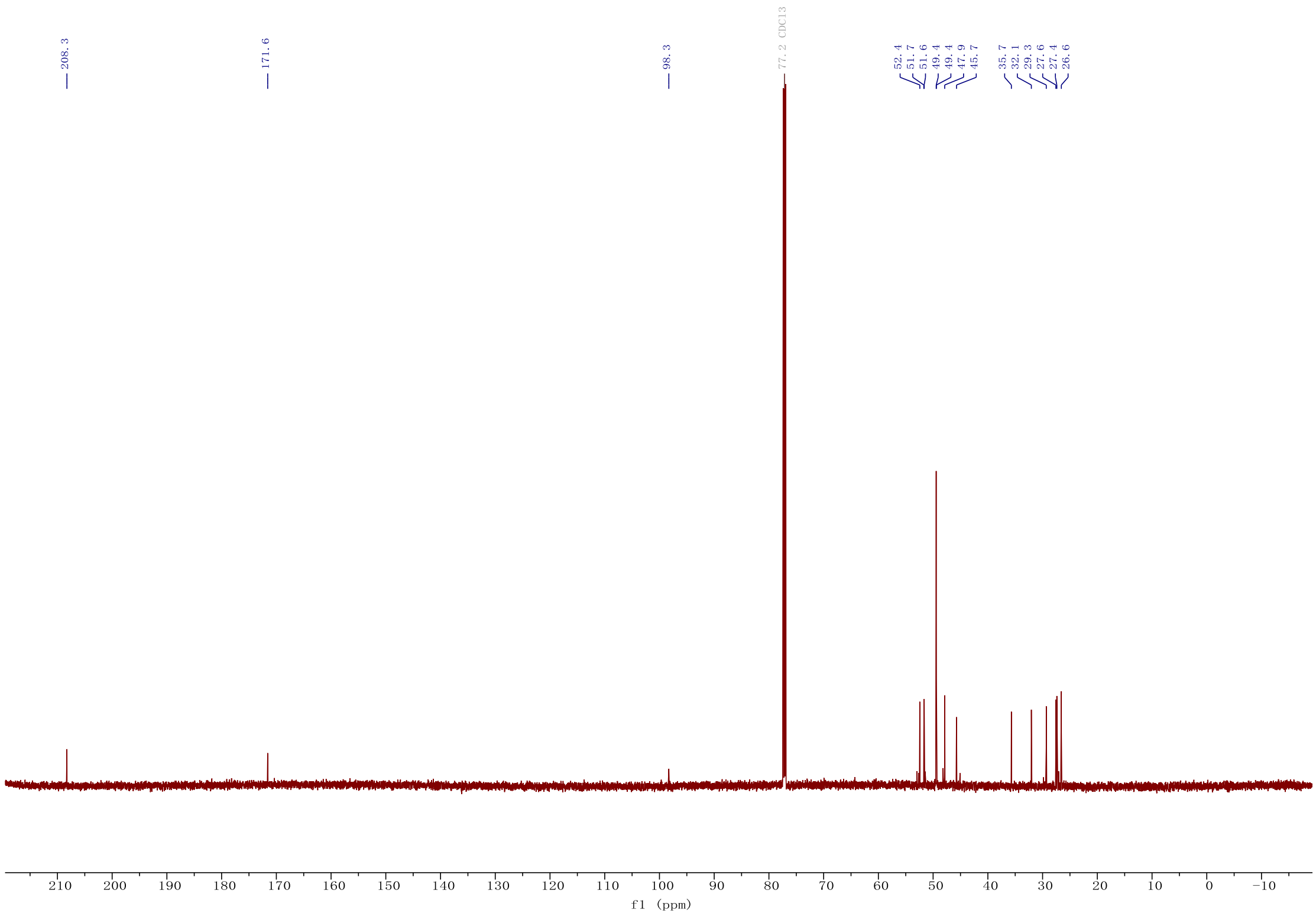

2-(bicyclo[1.1.1]pentan-1-yl)-1-morpholino-3-(3-(trichloromethyl)bicyclo[1.1.1]pentan-1-yl)propan-1-one (4k)

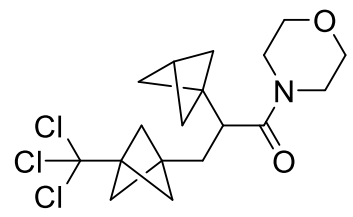

^{1}H NMR (600 MHz, CDCl$_3$)

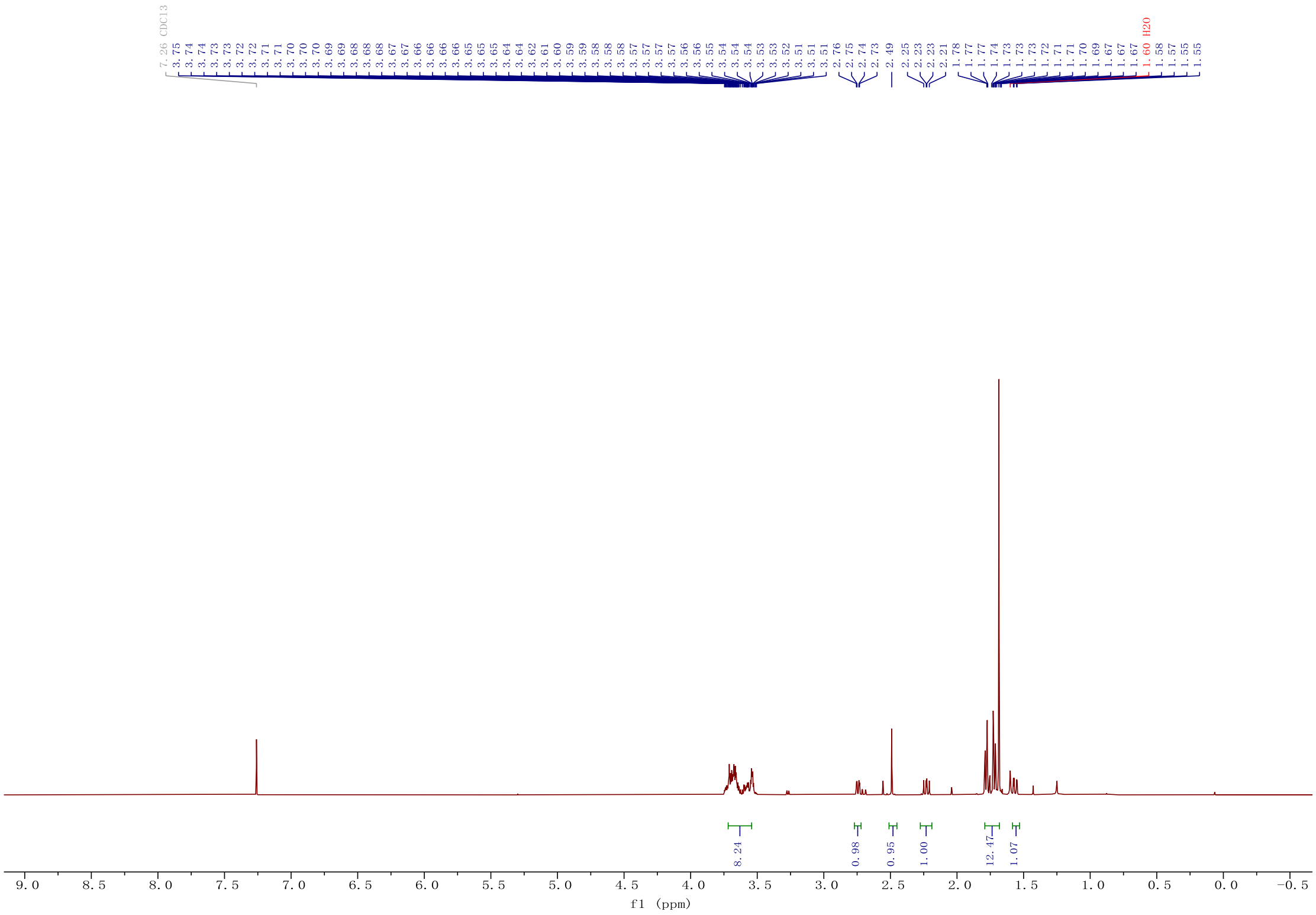

^{13}C NMR (151 MHz, CDCl$_3$)

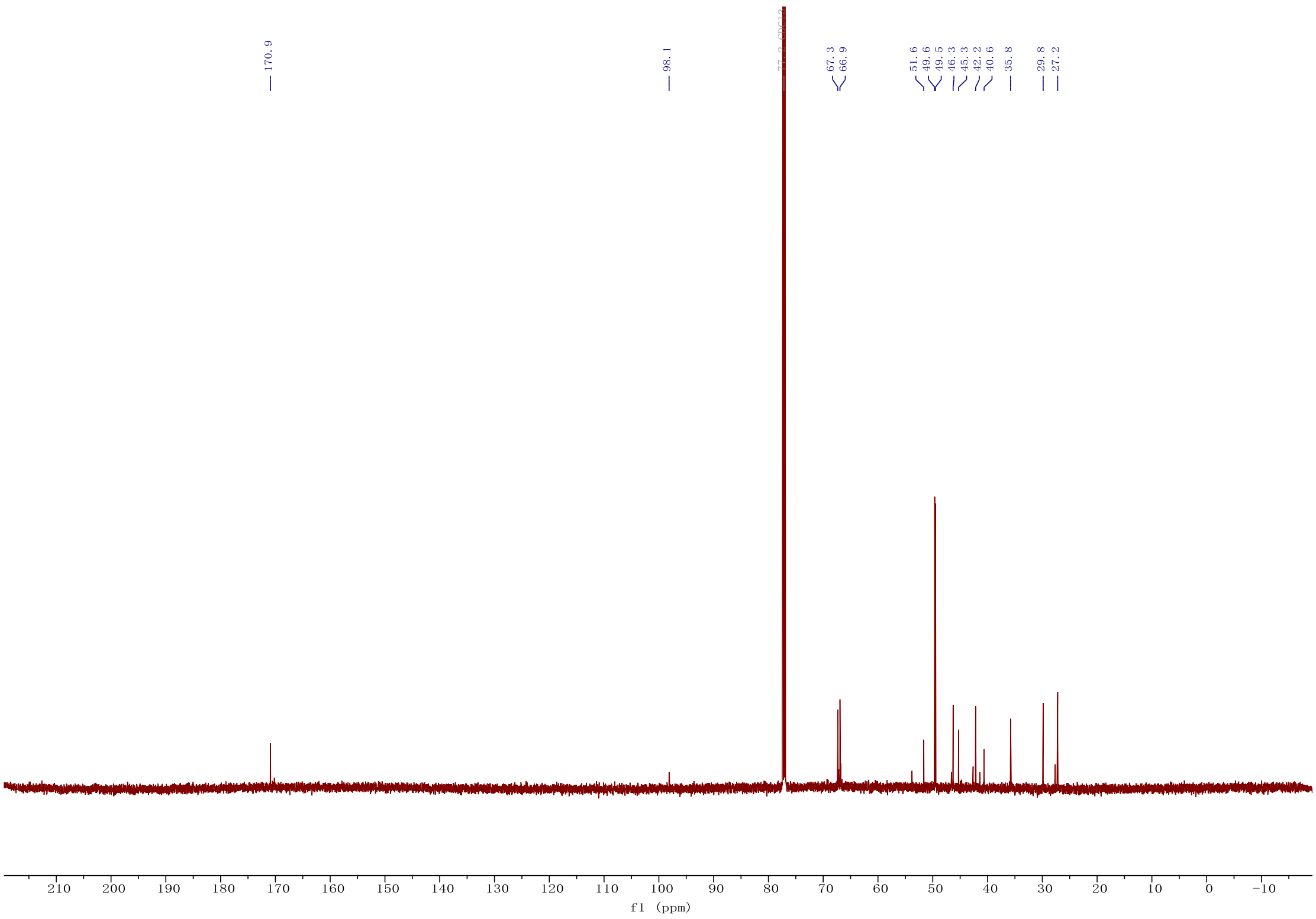

2-(bicyclo[3.1.1]heptan-1-yl)-*N*-phenyl-3-(5-(trichloromethyl)bicyclo[3.1.1]heptan-1-yl)propenamide (7a)

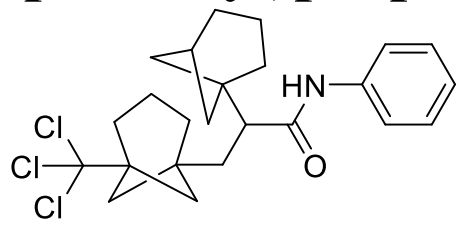

^{1}H NMR (600 MHz, $CDCl_3$)

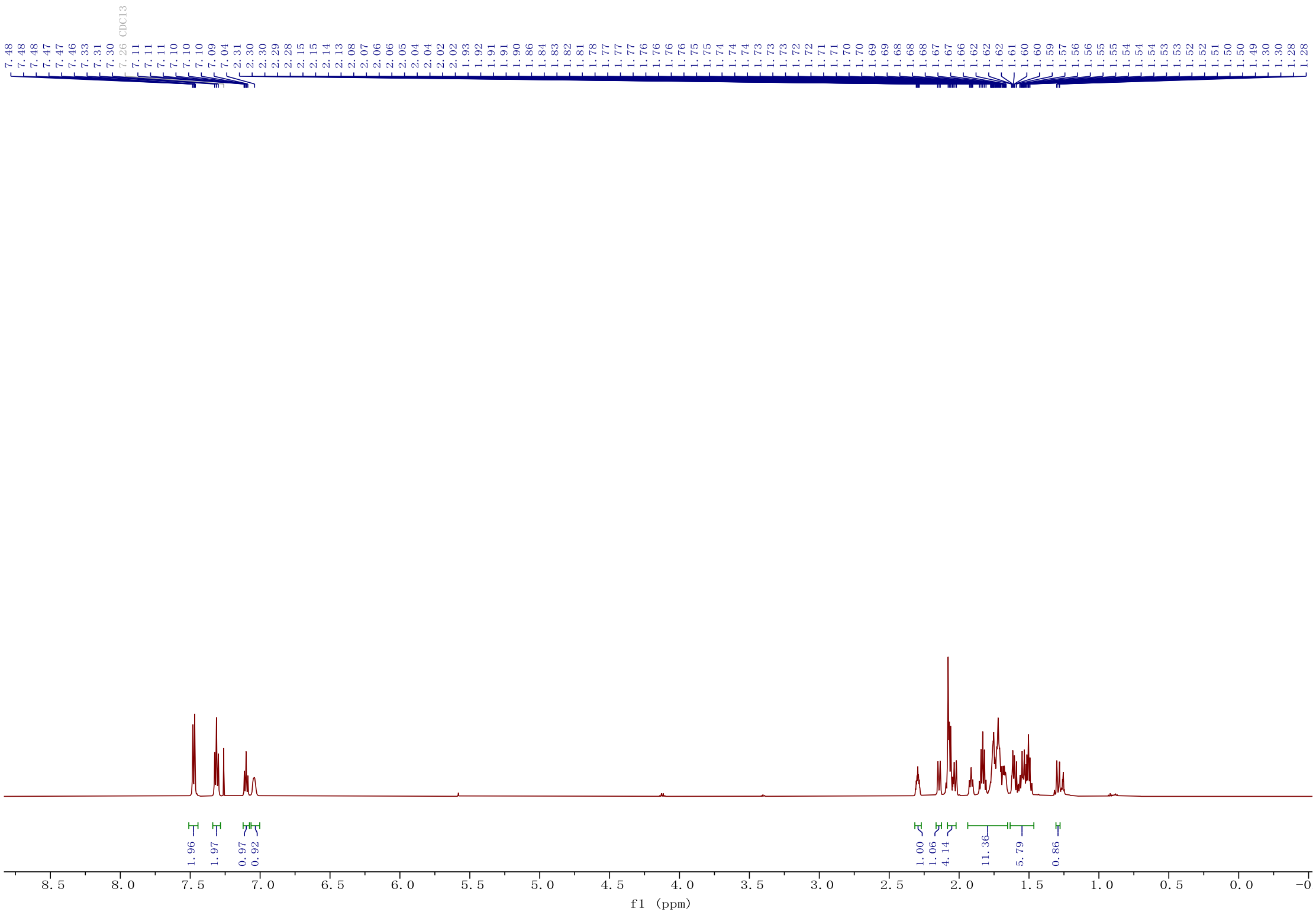

^{13}C NMR (151 MHz, $CDCl_3$)

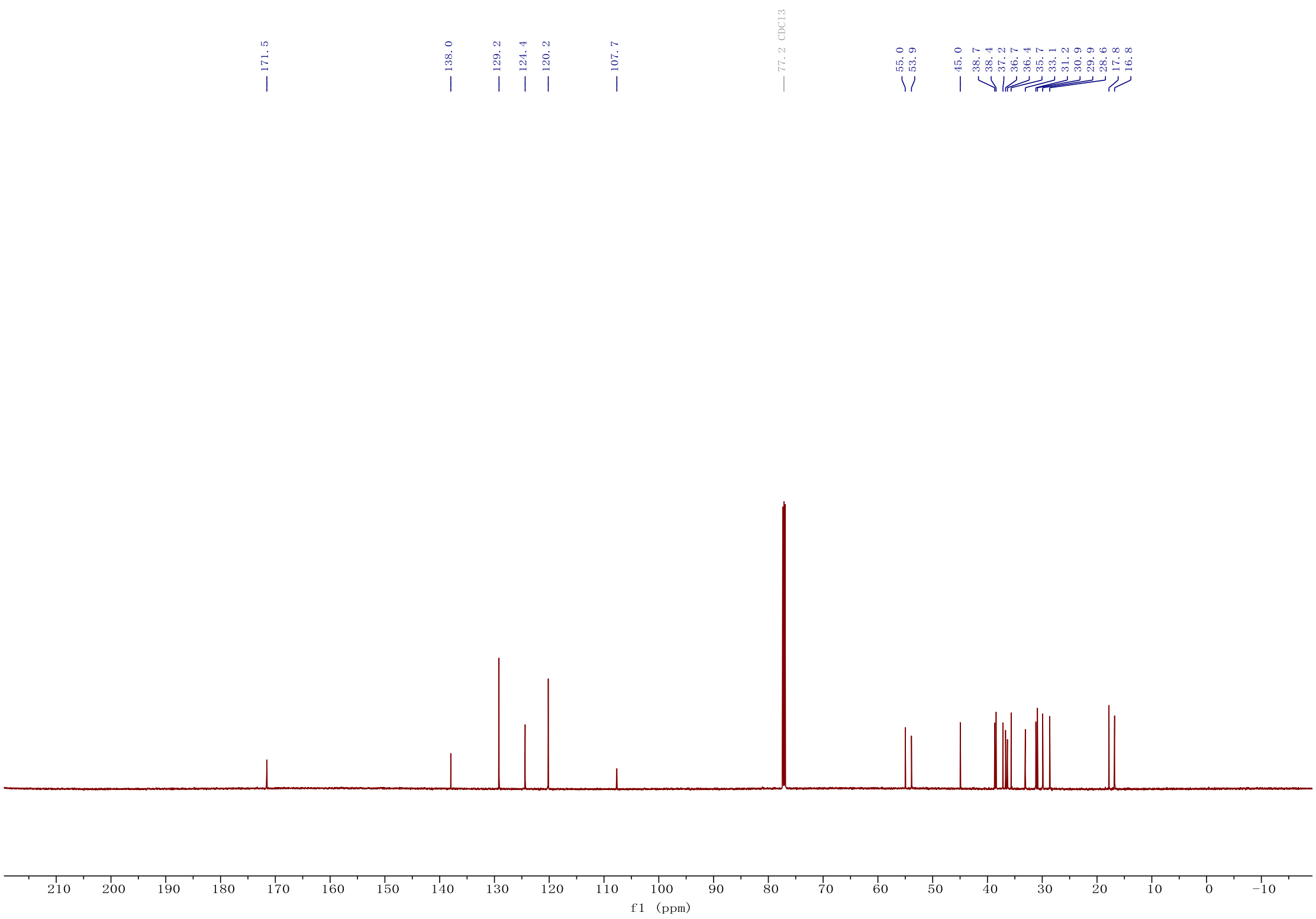

2-(bicyclo[3.1.1]heptan-1-yl)-*N*-(4-methoxyphenyl)-3-(5-(trichloromethyl)bicyclo[3.1.1]heptan-1-yl)propenamide (7b)

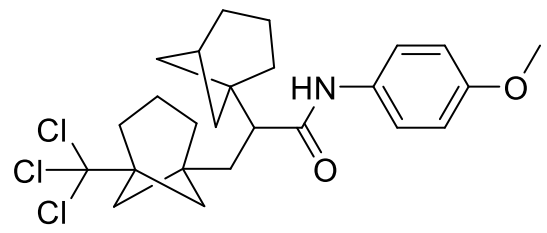

^{1}H NMR (600 MHz, CDCl$_3$)

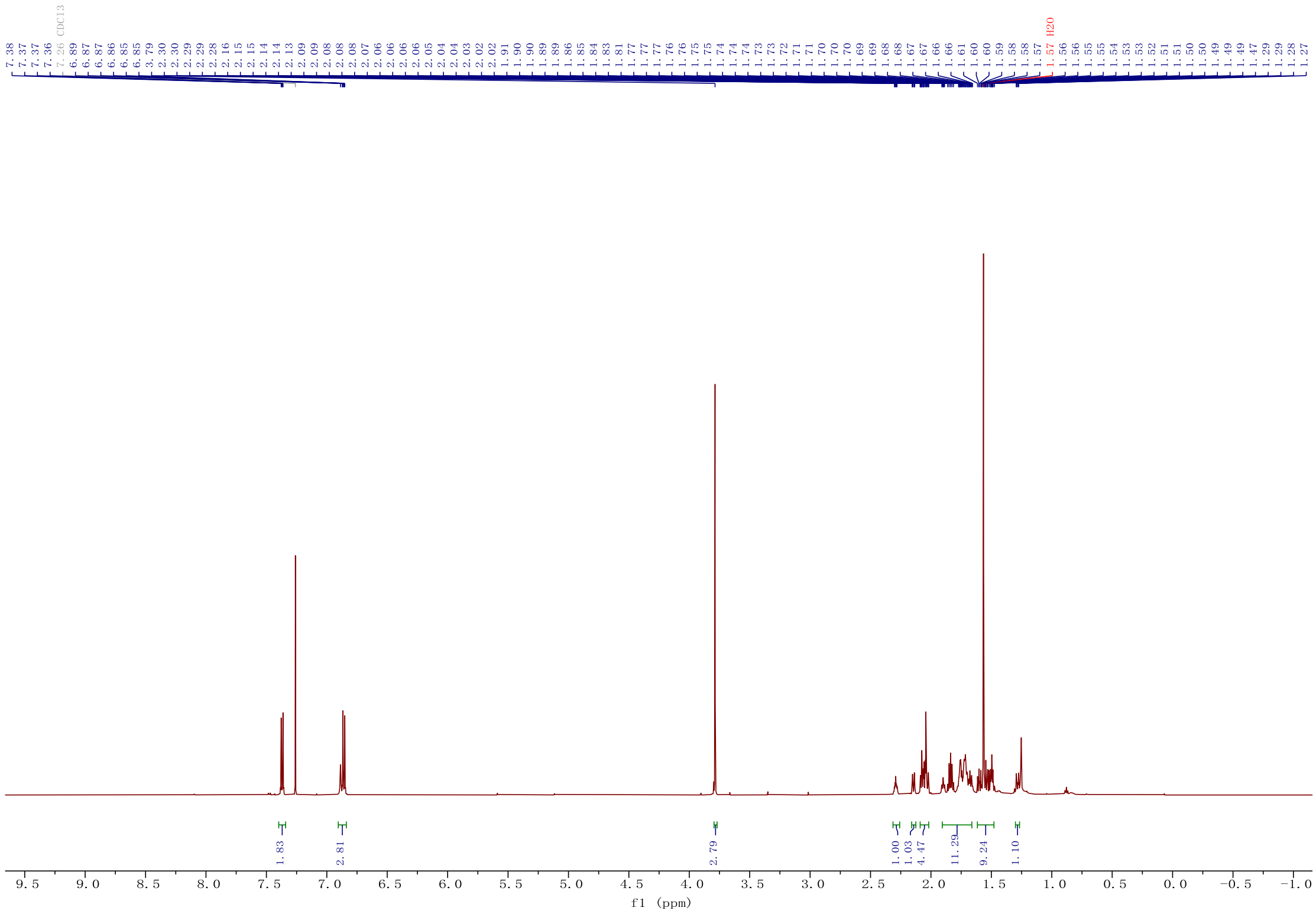

^{13}C NMR (151 MHz, CDCl$_3$)

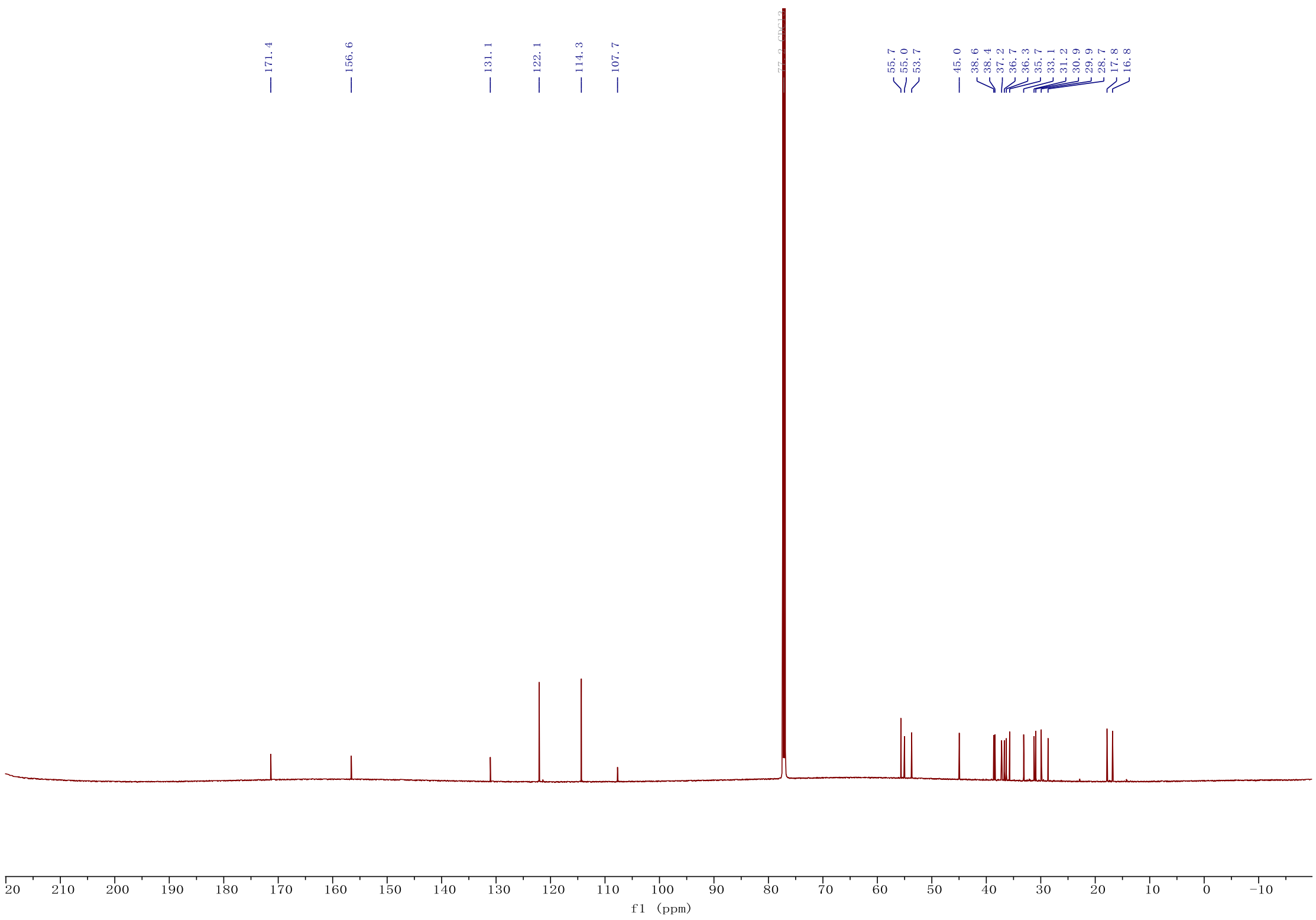

2-(bicyclo[3.1.1]heptan-1-yl)-*N*-(2-methyl-4-oxopentan-2-yl)-3-(5-(trichloromethyl)bicyclo[3.1.1]heptan-1-yl)propenamide (7c)

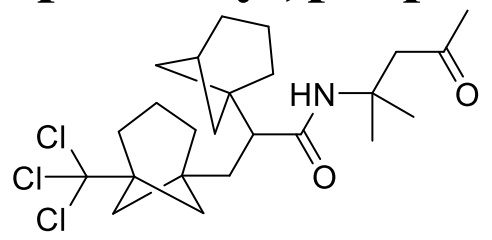

^{1}H NMR (600 MHz, CDCl$_3$)

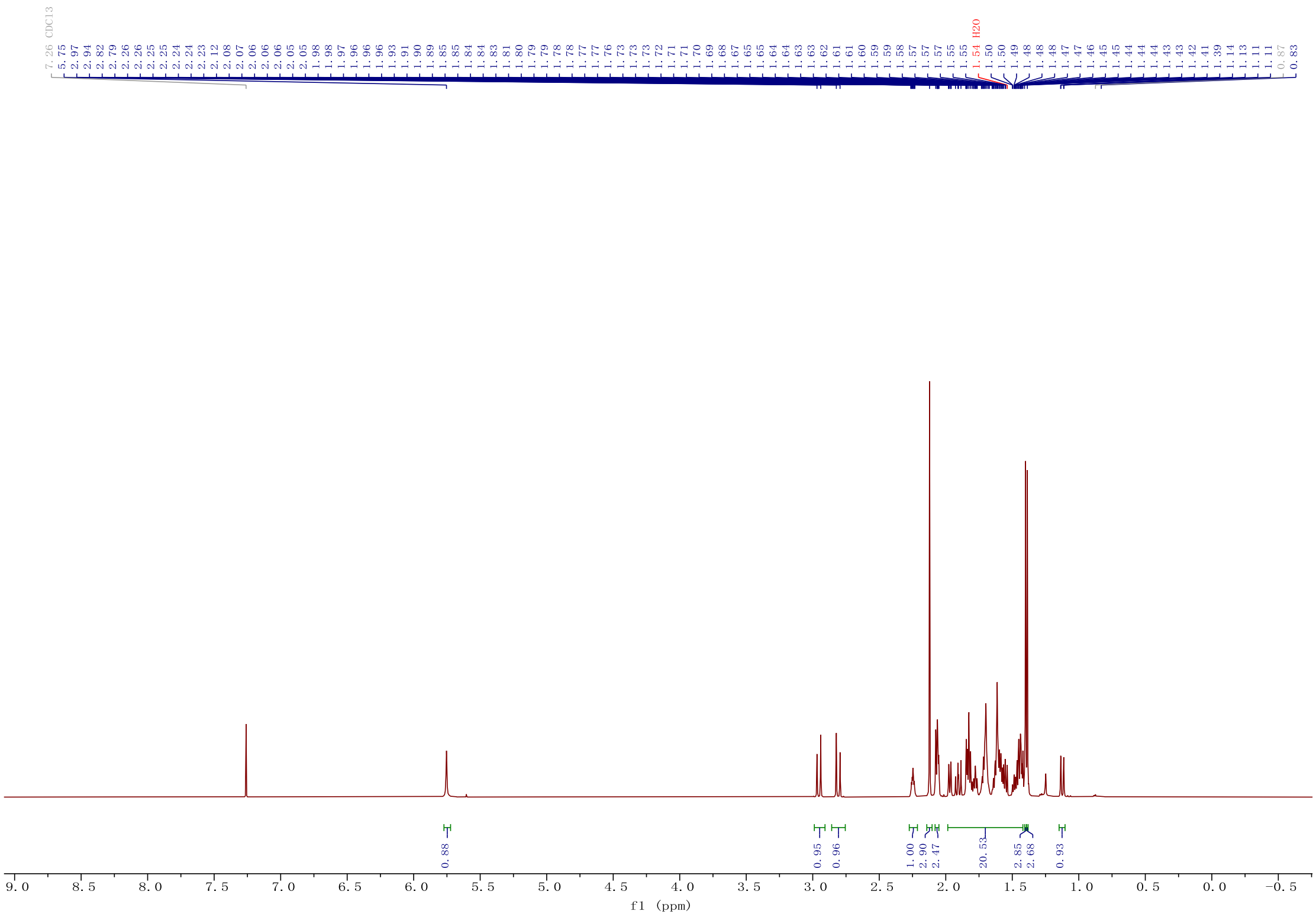

^{13}C NMR (151 MHz, CDCl$_3$)

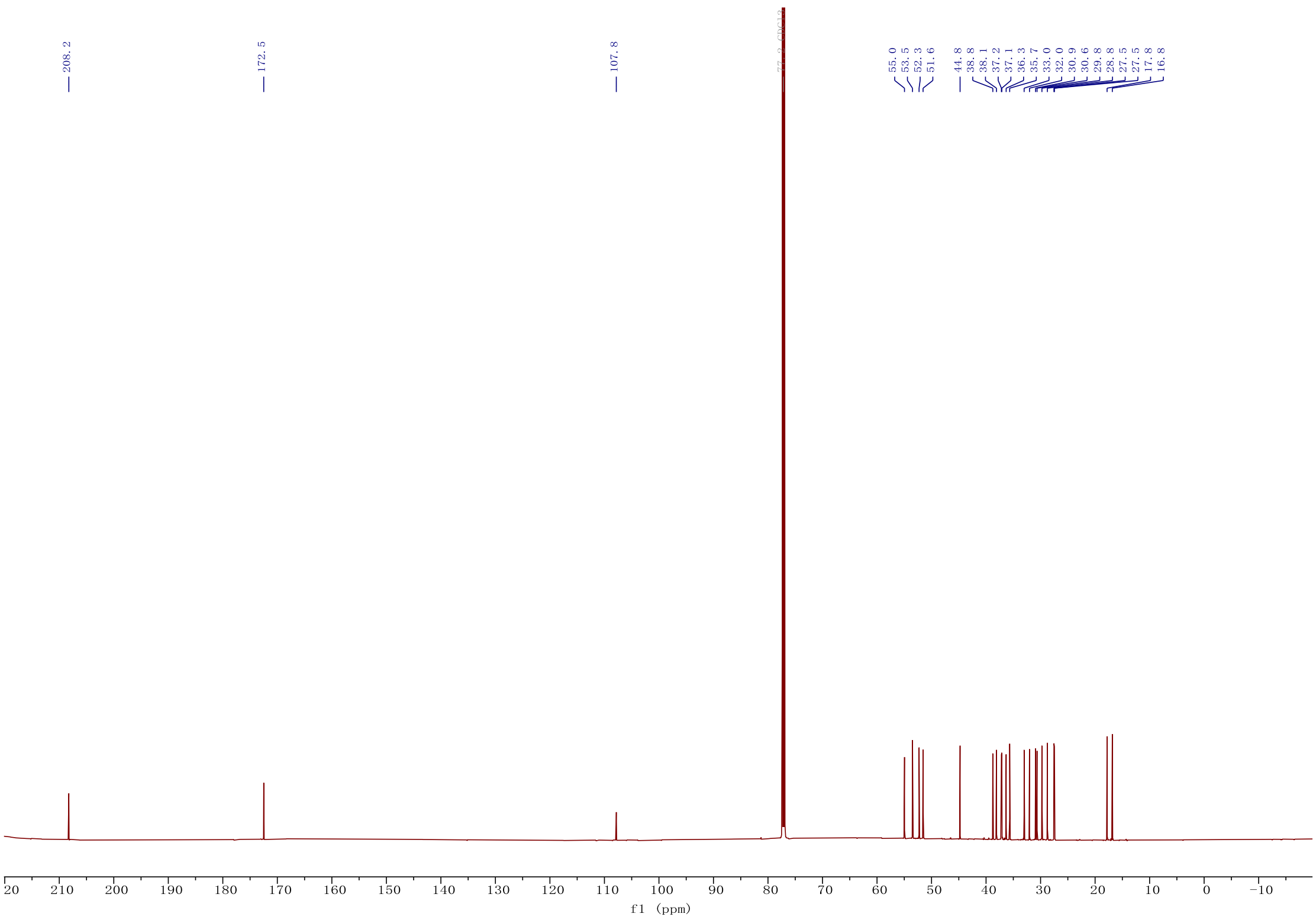

2-(bicyclo[3.1.1]heptan-1-yl)-2-methyl-*N*-phenyl-3-(5-(trichloromethyl)bicyclo[3.1.1]heptan-1-yl)propenamide (7d)

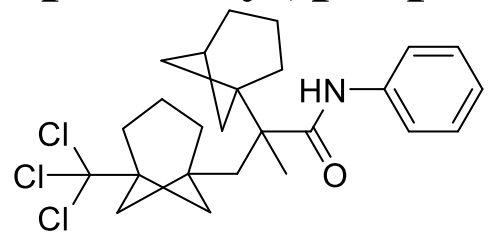

^{1}H NMR (600 MHz, $CDCl_3$)

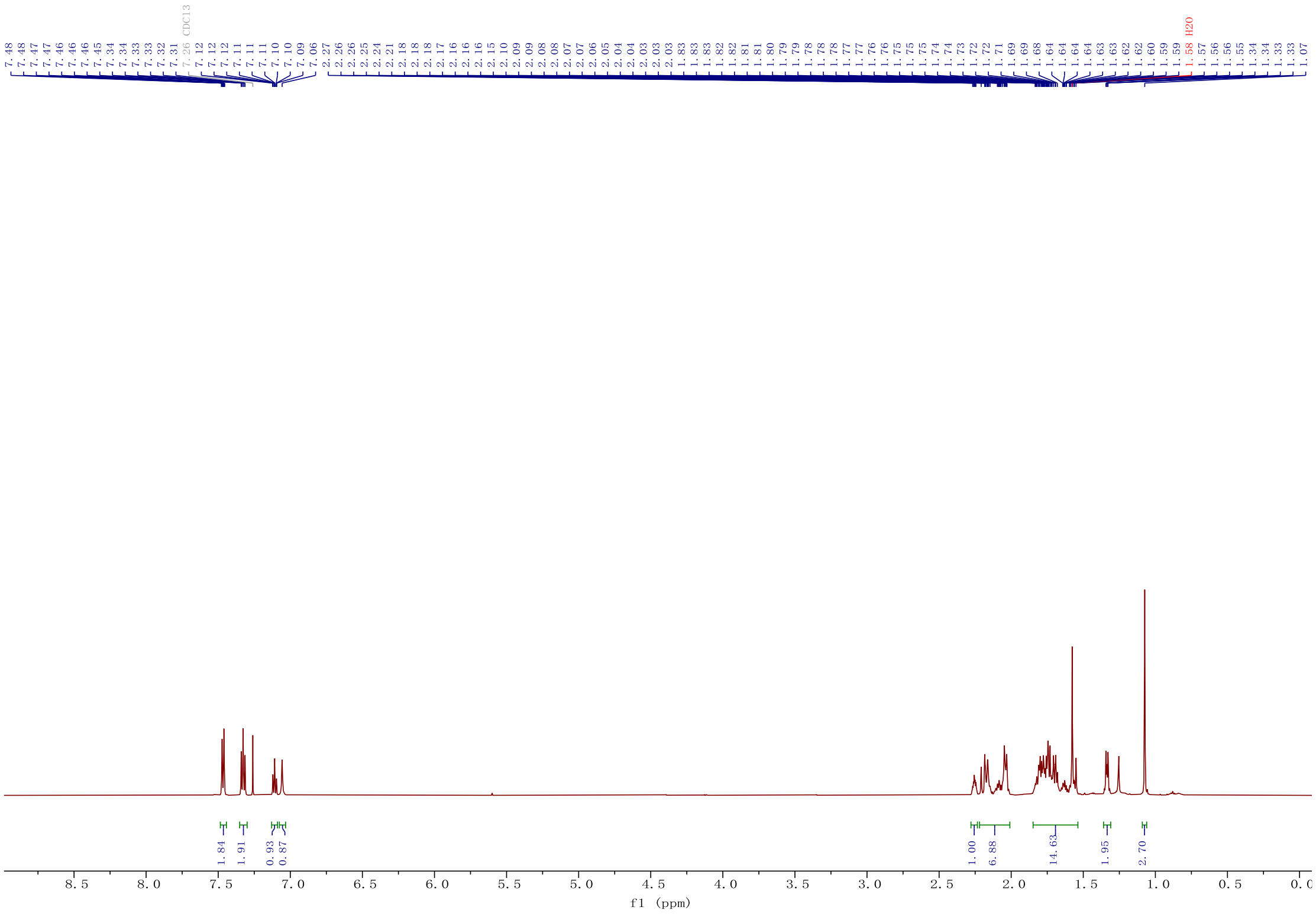

^{13}C NMR (151 MHz, $CDCl_3$)

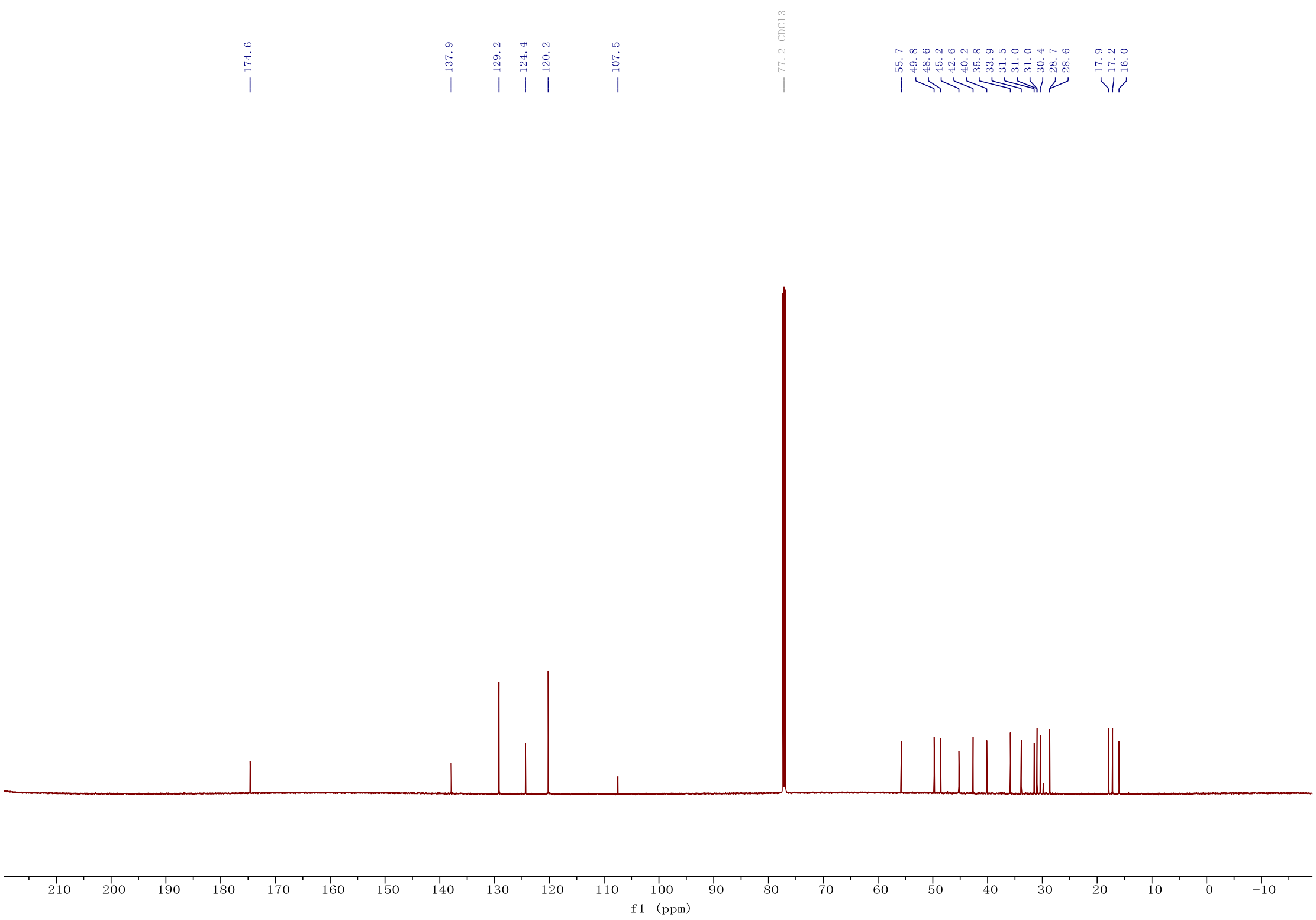

2-(bicyclo[1.1.1]pentan-1-yl)-3-(3-(1,1,1,3,3,3-hexamethyl-2-(trimethylsilyl)trisilan-2-yl)bicyclo[1.1.1]pentan-1-yl)-*N*-phenylpropanamide (8a)

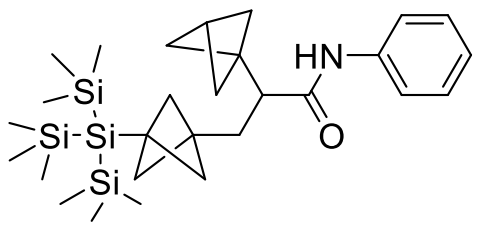

^{1}H NMR (600 MHz, $CDCl_3$)

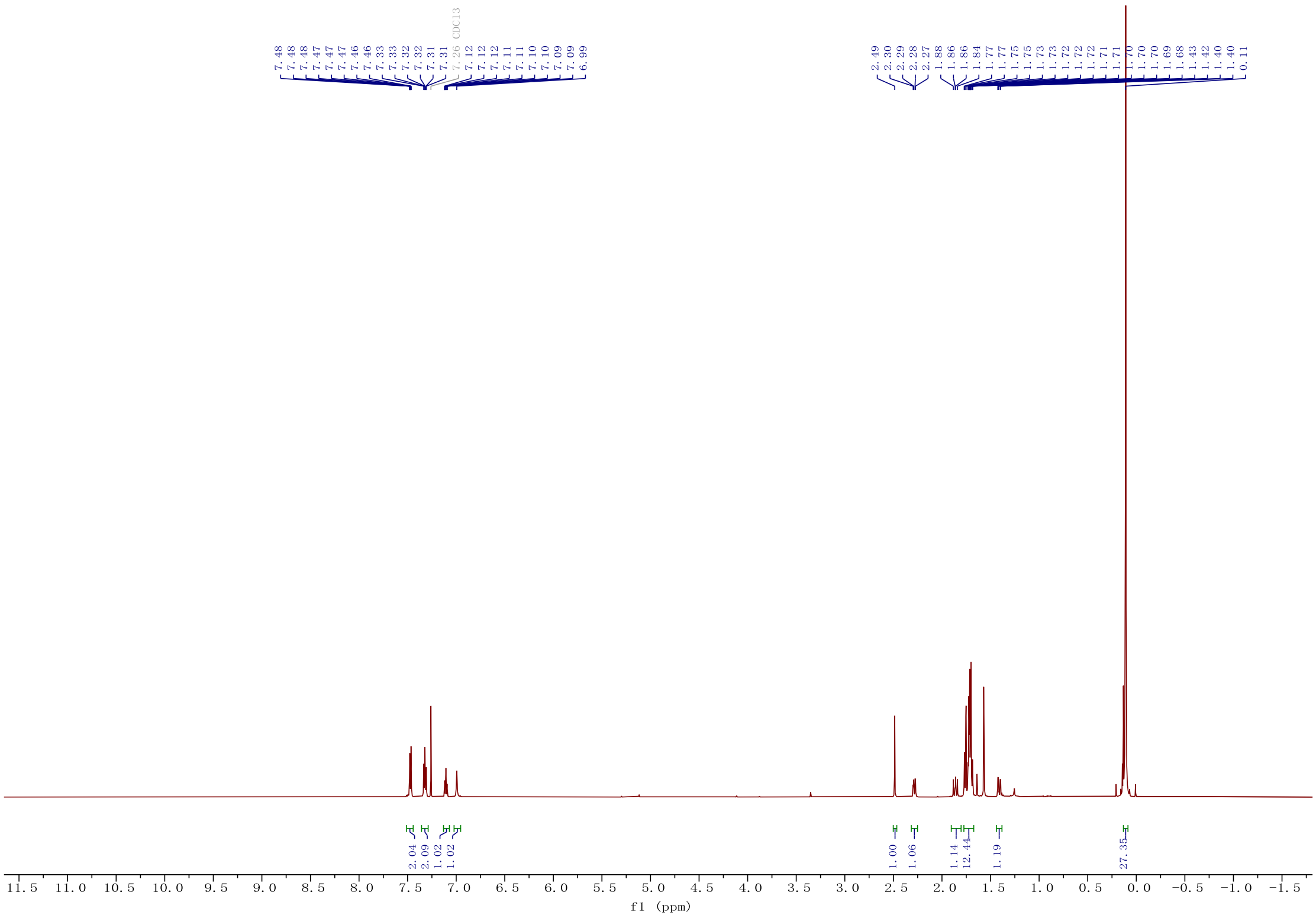

^{13}C NMR (151 MHz, $CDCl_3$)

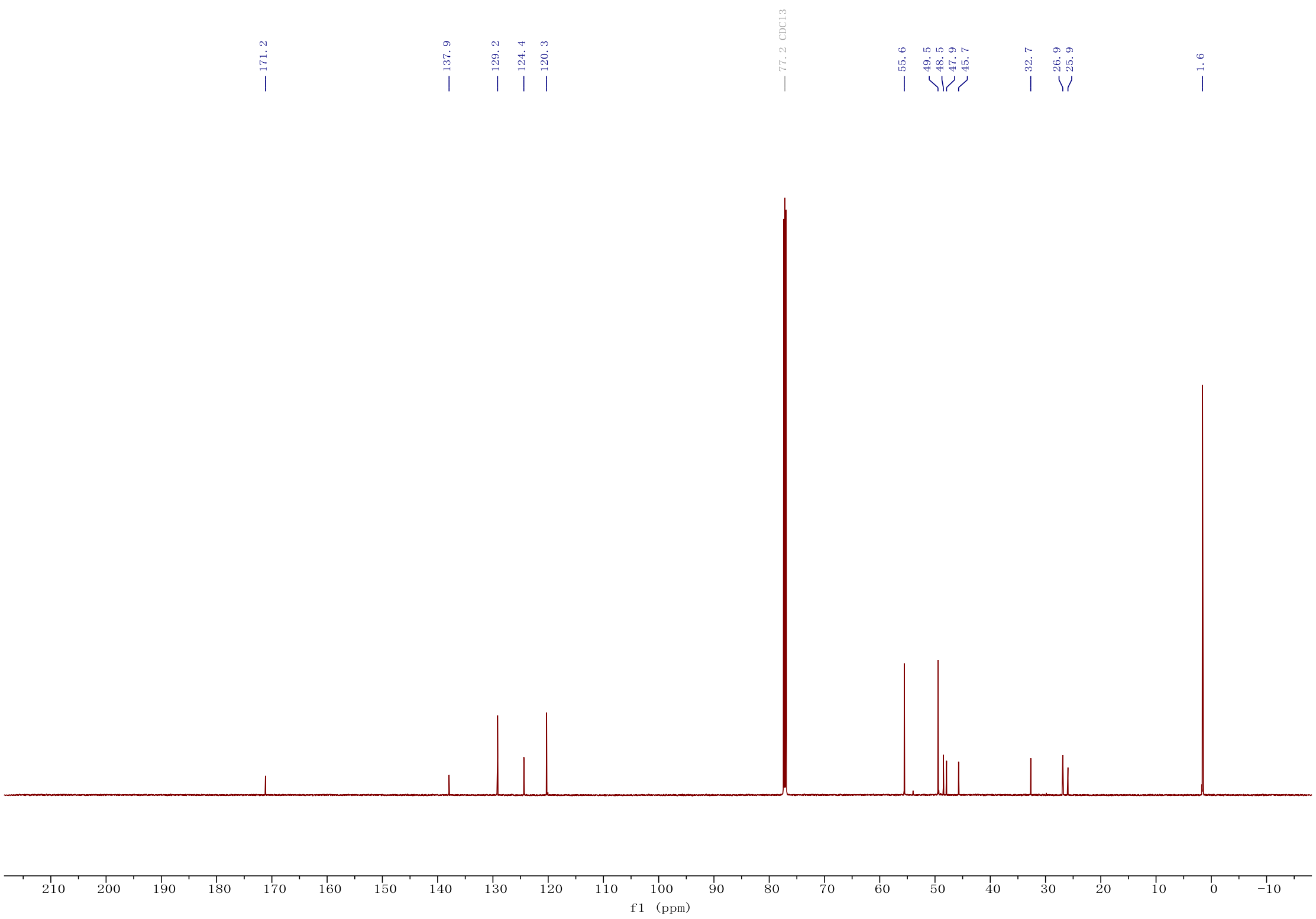

2-(bicyclo[1.1.1]pentan-1-yl)-3-(3-(1,1,1,3,3,3-hexamethyl-2-(trimethylsilyl)trisilan-2-yl)bicyclo[1.1.1]pentan-1-yl)-*N*-(4-(trifluoromethyl)phenyl)propenamide (8b)

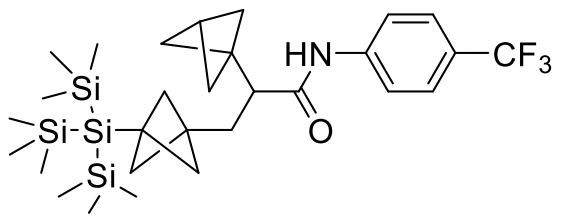

^{1}H NMR (600 MHz, CDCl$_3$)

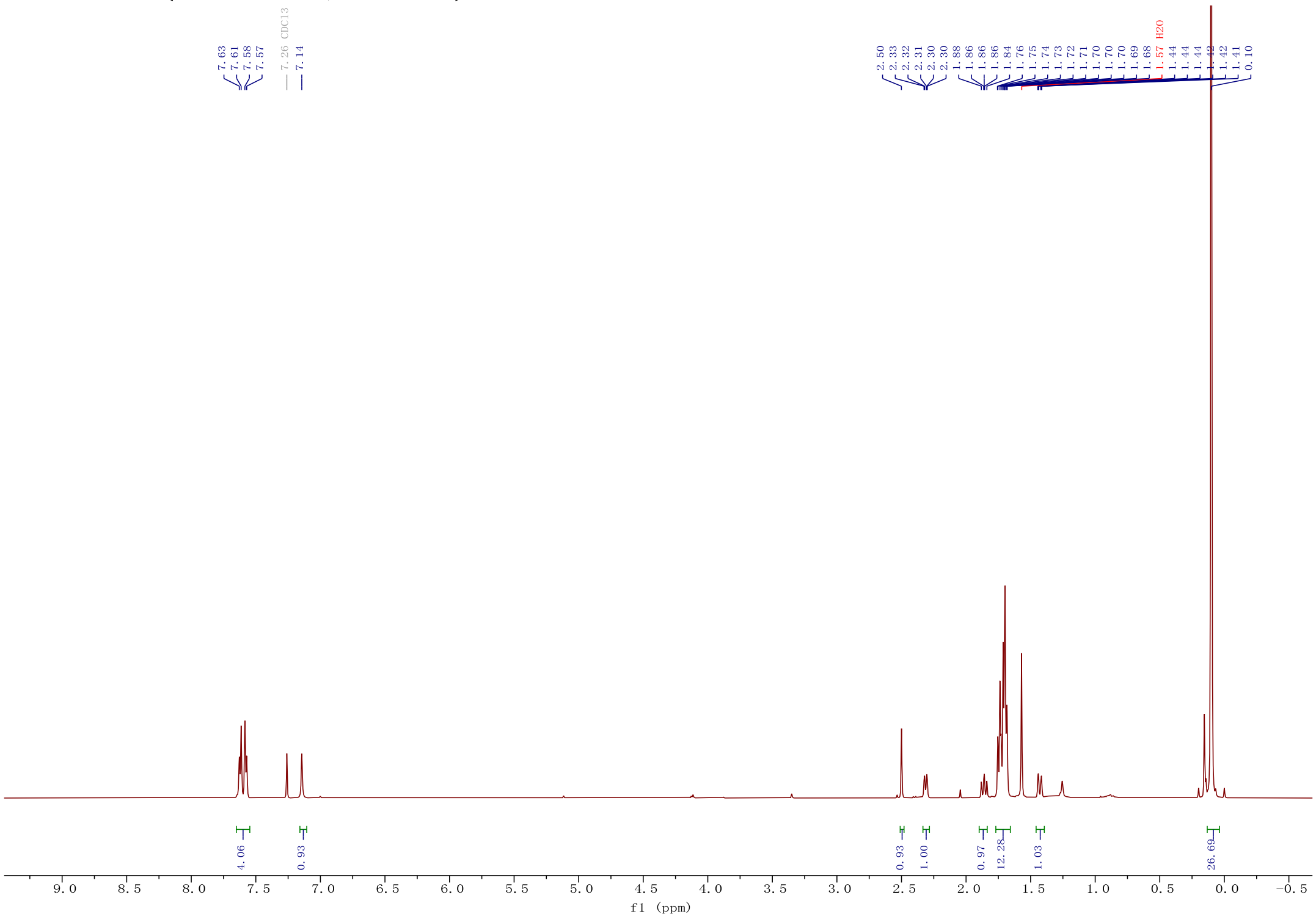

^{13}C NMR (151 MHz, CDCl$_3$)

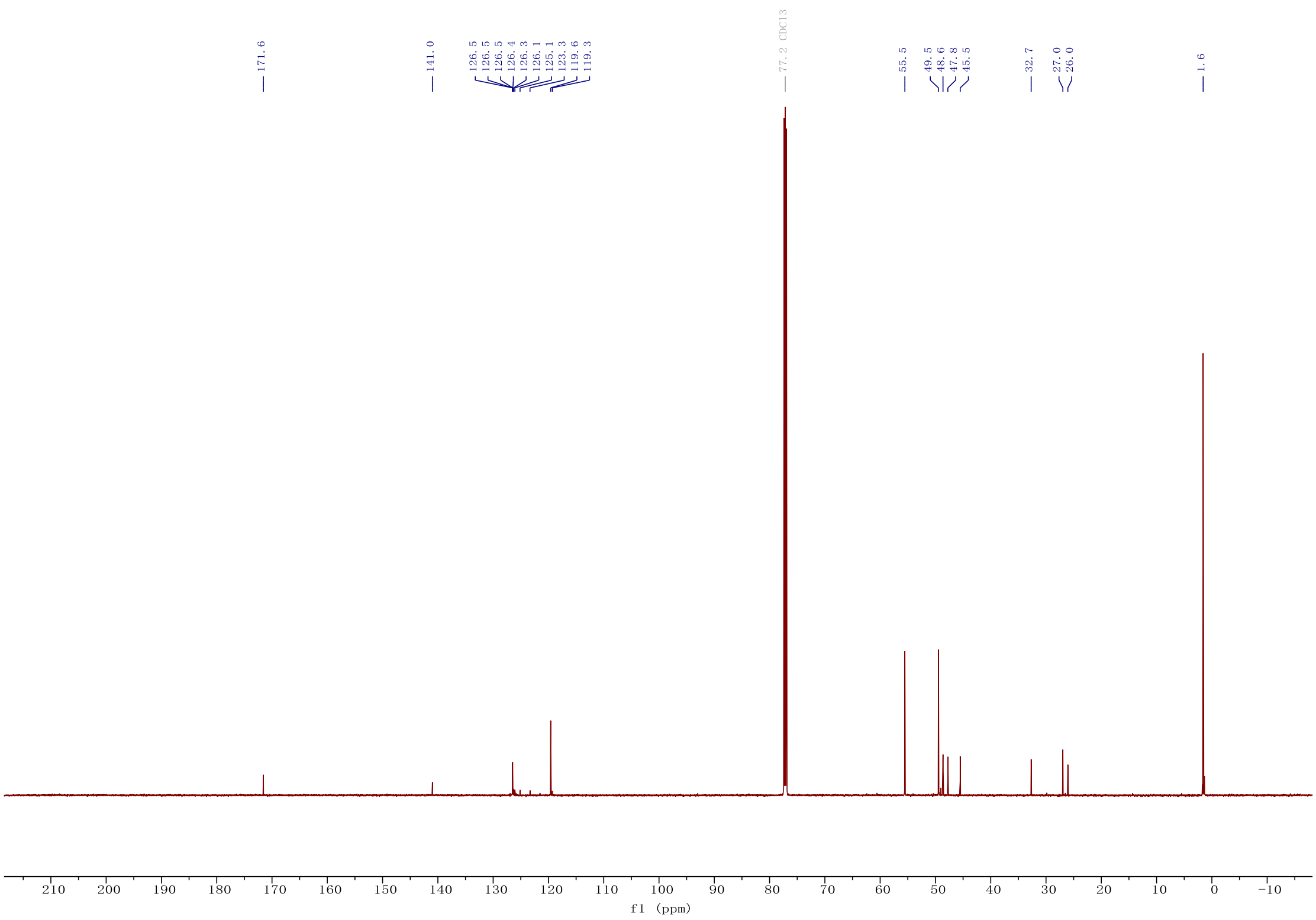

2-(bicyclo[1.1.1]pentan-1-yl)-*N*-(4-fluorophenyl)-3-(3-(1,1,1,3,3,3-hexamethyl-2-(trimethylsilyl)trisilan-2-yl)bicyclo[1.1.1]pentan-1-yl)propenamide (8c)

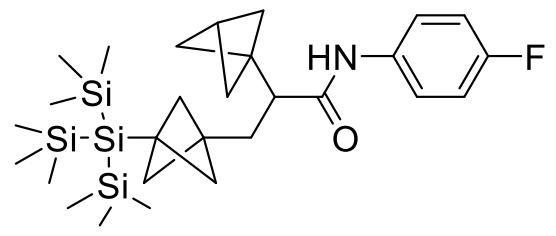

^{1}H NMR (600 MHz, CDCl$_3$)

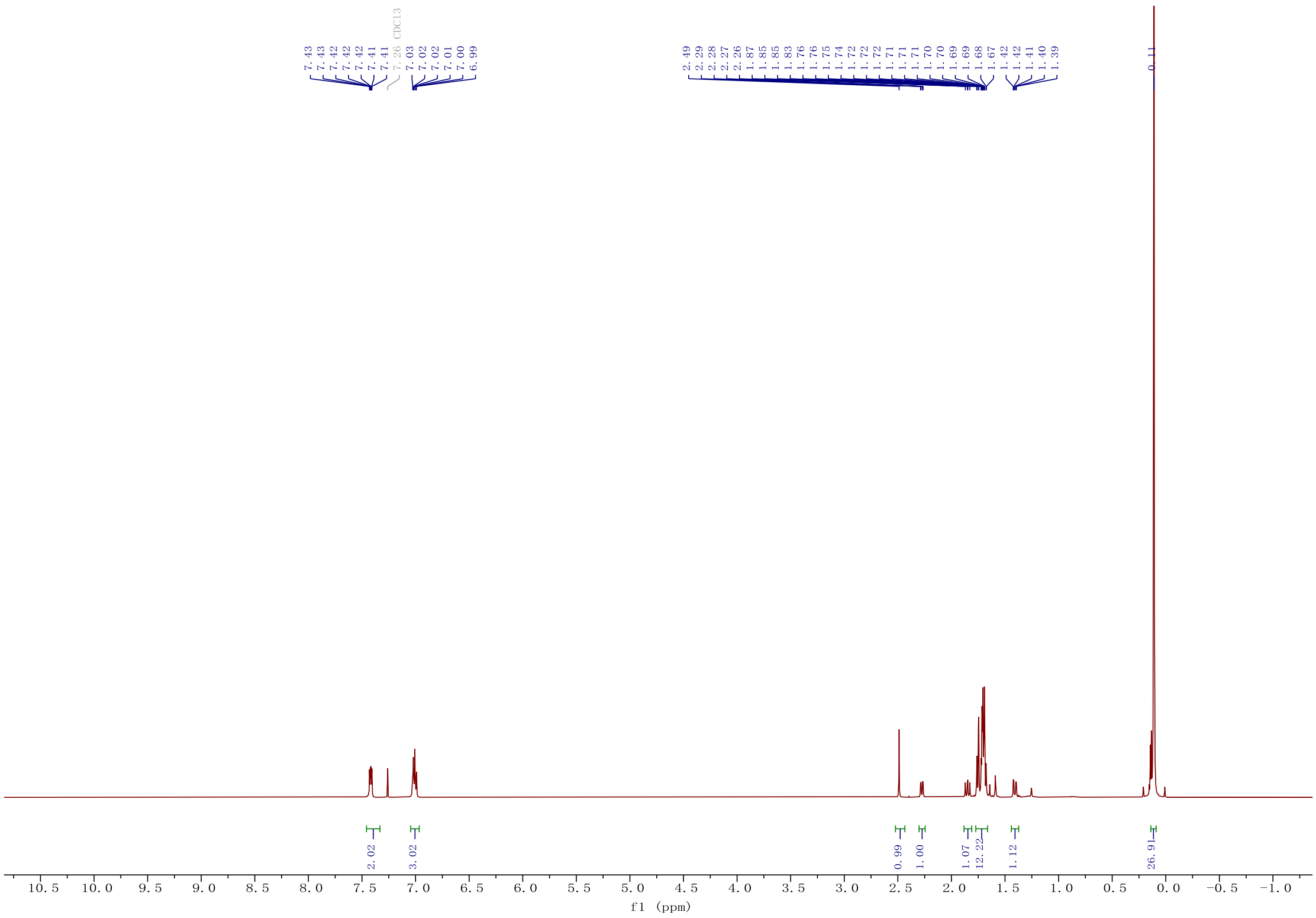

^{13}C NMR (151 MHz, CDCl$_3$)

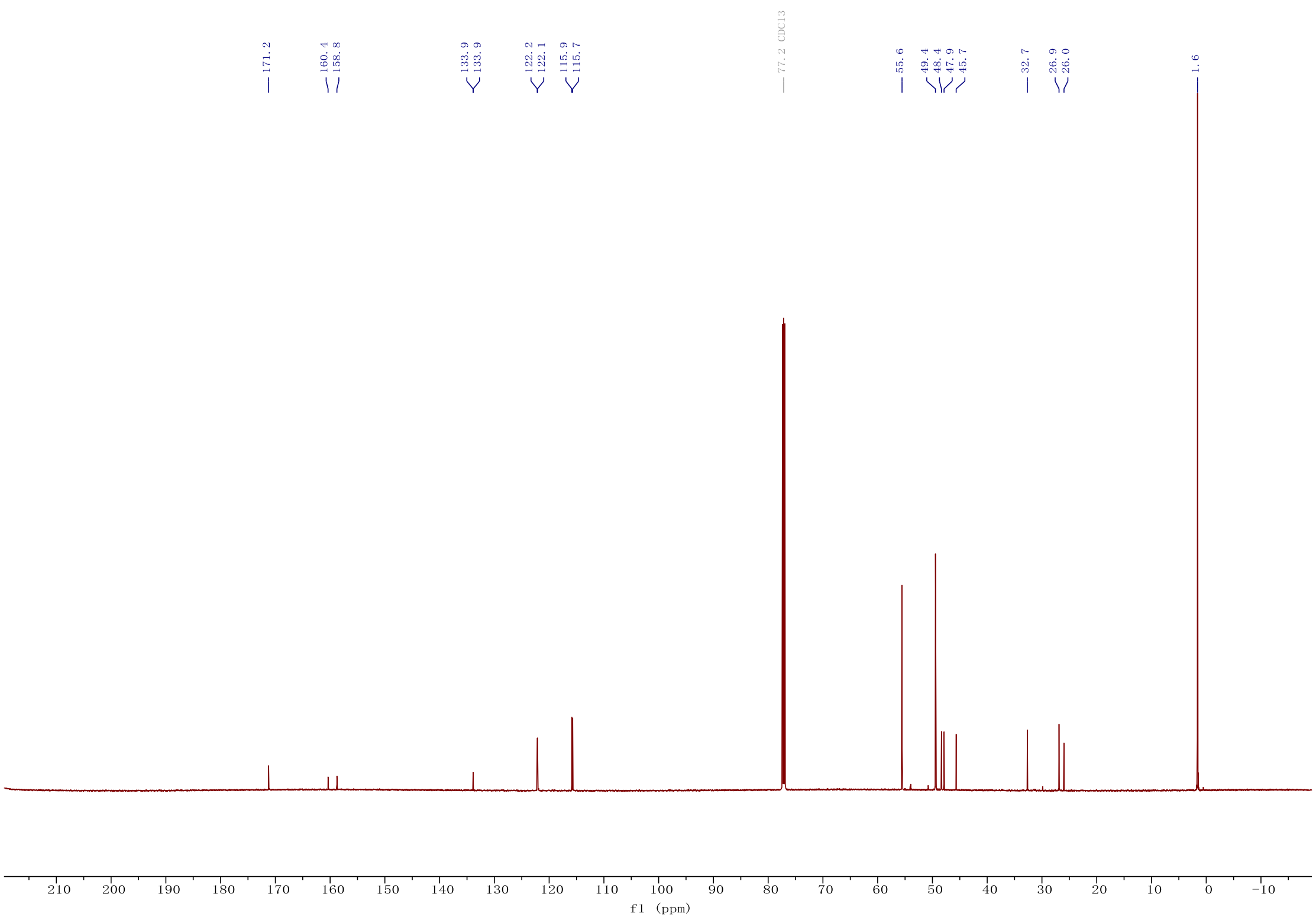

2-(bicyclo[1.1.1]pentan-1-yl)-3-(3-(1,1,1,3,3,3-hexamethyl-2-(trimethylsilyl)trisilan-2-yl)bicyclo[1.1.1]pentan-1-yl)-*N*-(p-tolyl)propenamide (8d)

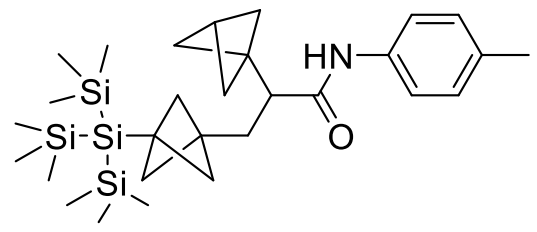

^{1}H NMR (600 MHz, CDCl$_3$)

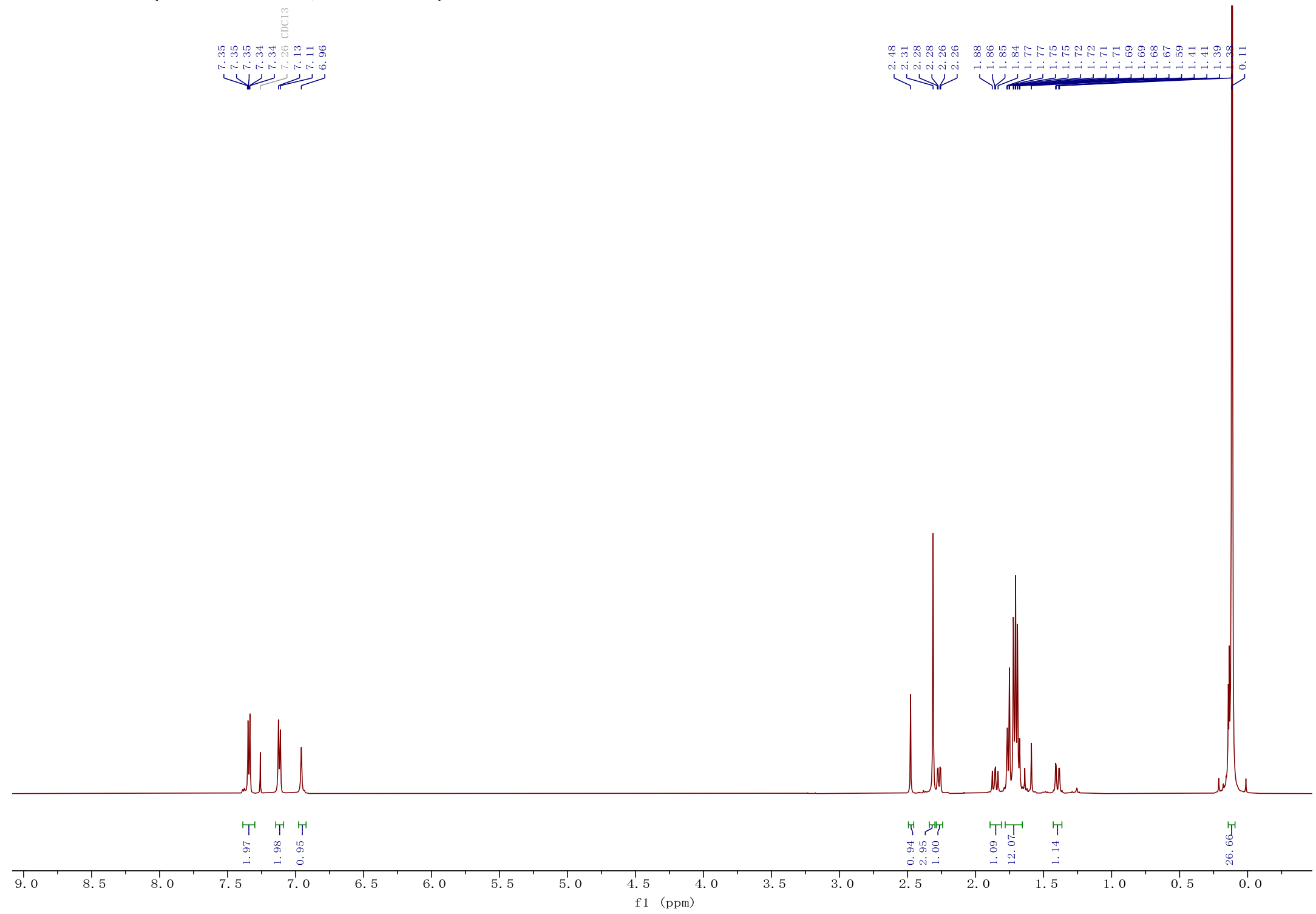

^{13}C NMR (151 MHz, CDCl$_3$)

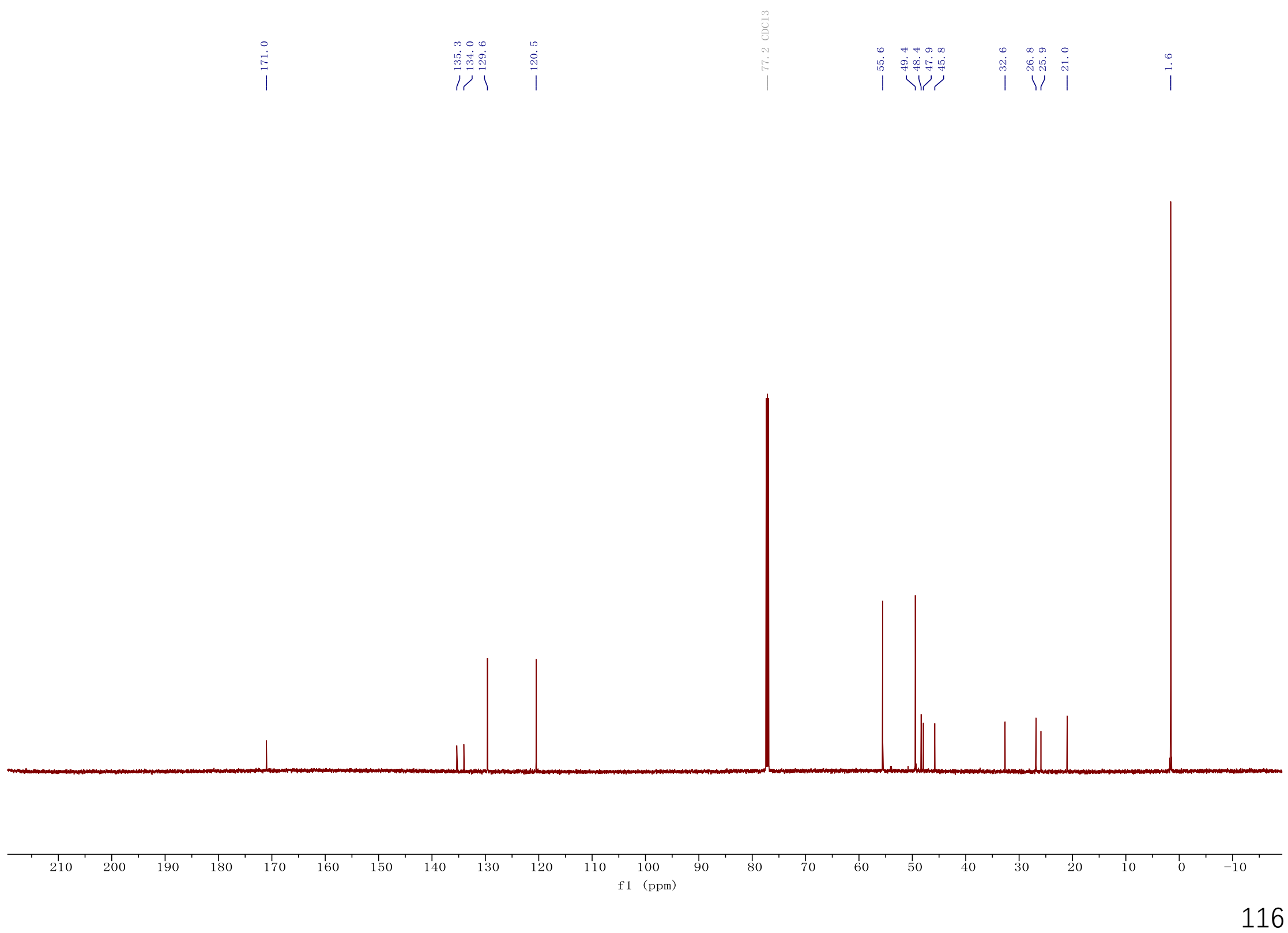

2-(bicyclo[1.1.1]pentan-1-yl)-*N*-(3,5-dimethylphenyl)-3-(3-(1,1,1,3,3,3-hexamethyl-2-(trimethylsilyl)trisilan-2-yl)bicyclo[1.1.1]pentan-1-yl)propenamide (8e)

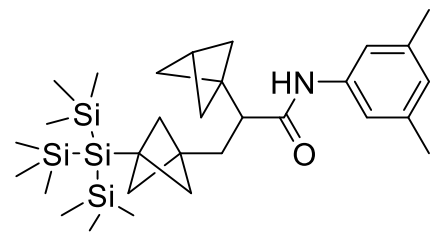

^{1}H NMR (600 MHz, CDCl$_3$)

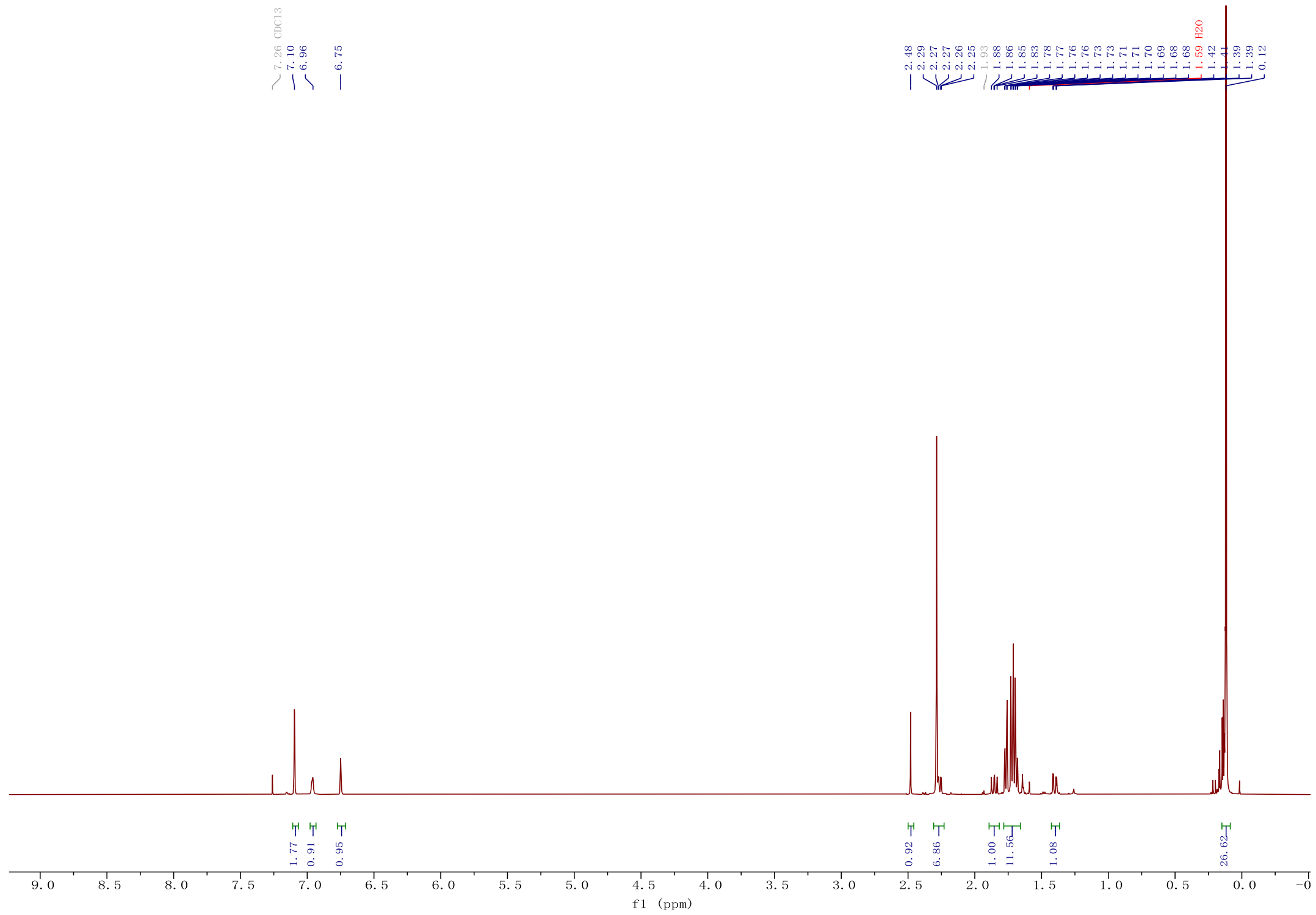

^{13}C NMR (151 MHz, CDCl$_3$)

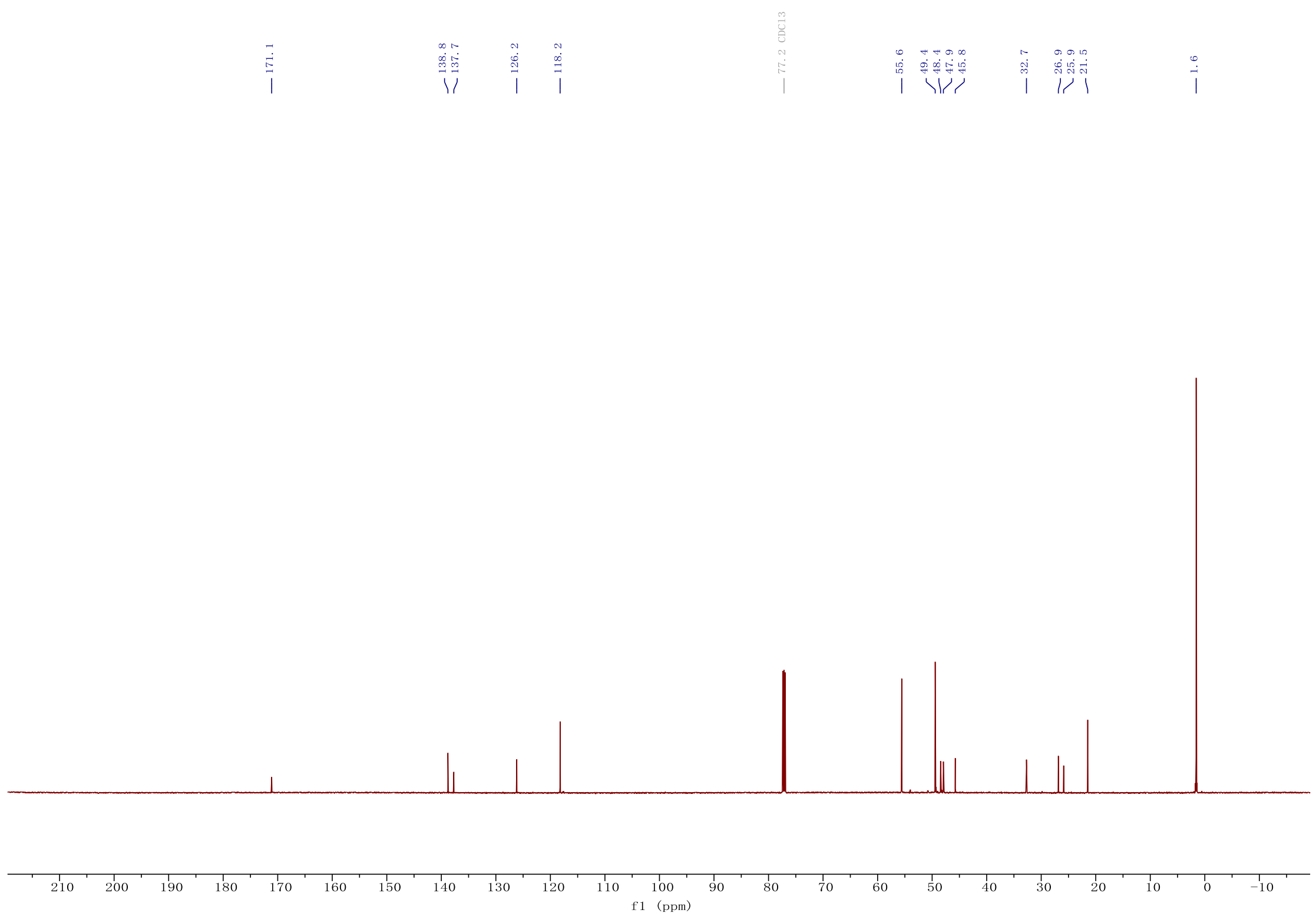

2-(bicyclo[1.1.1]pentan-1-yl)-*N*-(4-(tert-butyl)phenyl)-3-(3-(1,1,1,3,3,3-hexamethyl-2-(trimethylsilyl)trisilan-2-yl)bicyclo[1.1.1]pentan-1-yl)propenamide (8f)

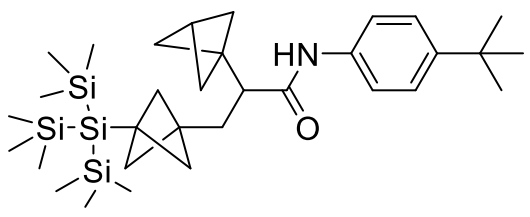

^{1}H NMR (600 MHz, CDCl$_3$)

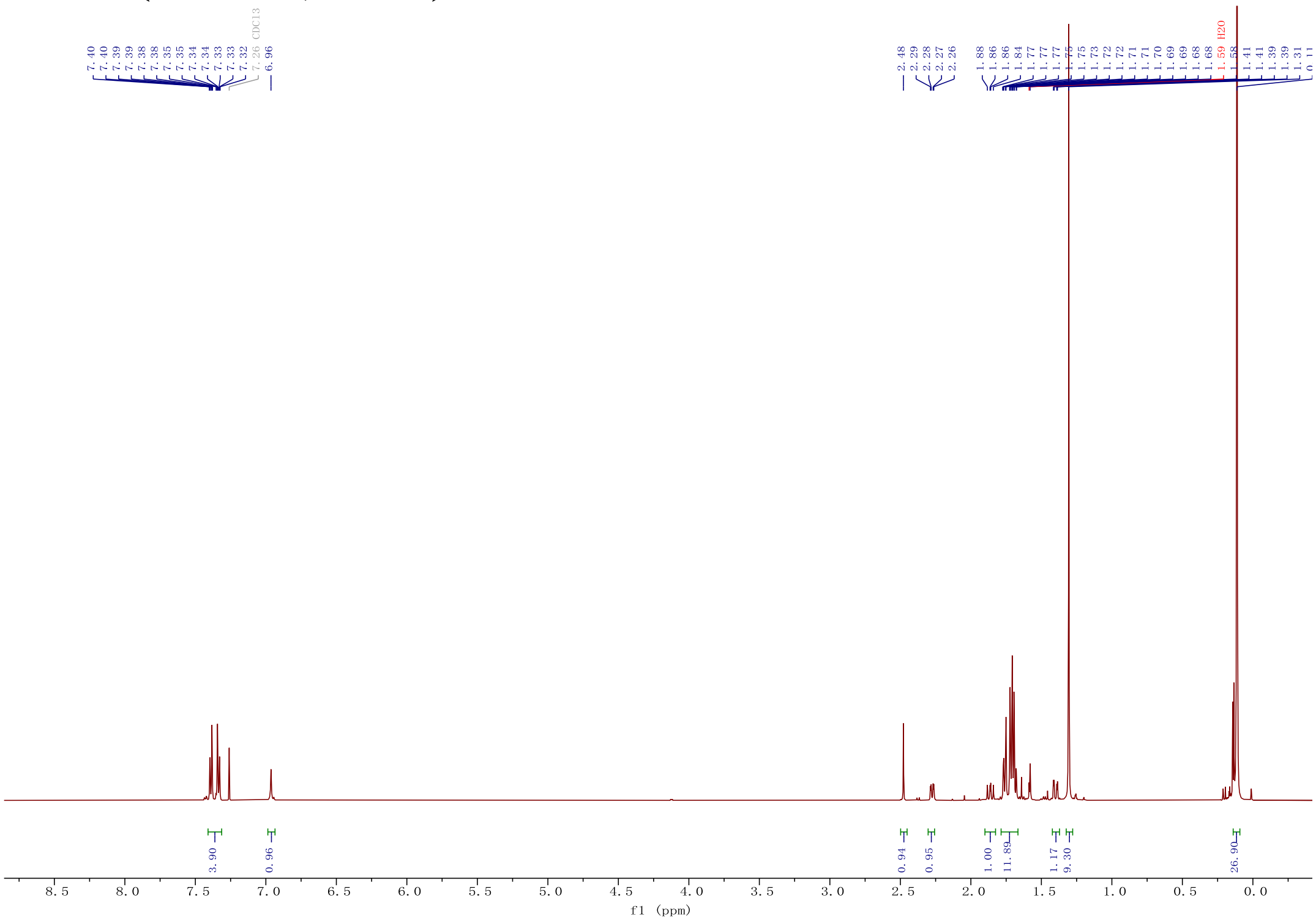

^{13}C NMR (151 MHz, CDCl$_3$)

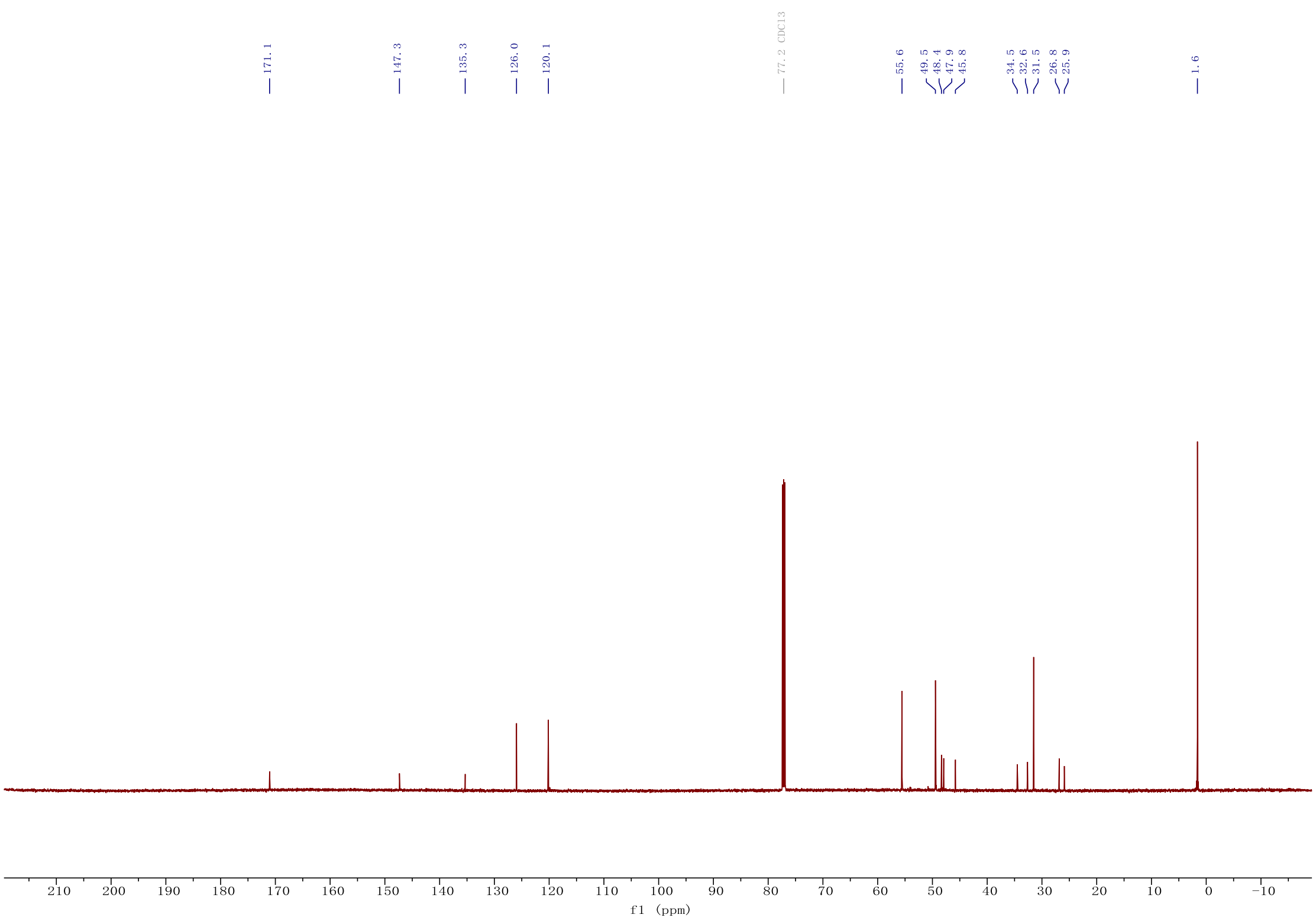

2-(bicyclo[1.1.1]pentan-1-yl)-3-(3-(1,1,1,3,3,3-hexamethyl-2-(trimethylsilyl)trisilan-2-yl)bicyclo[1.1.1]pentan-1-yl)-*N*-(4-methoxyphenyl)propenamide (8g)

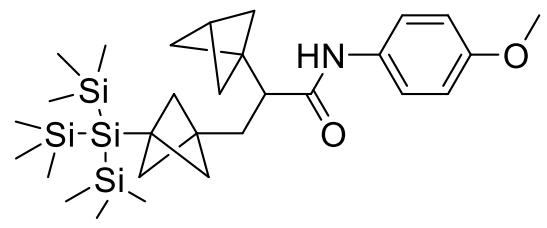

^{1}H NMR (600 MHz, $CDCl_3$)

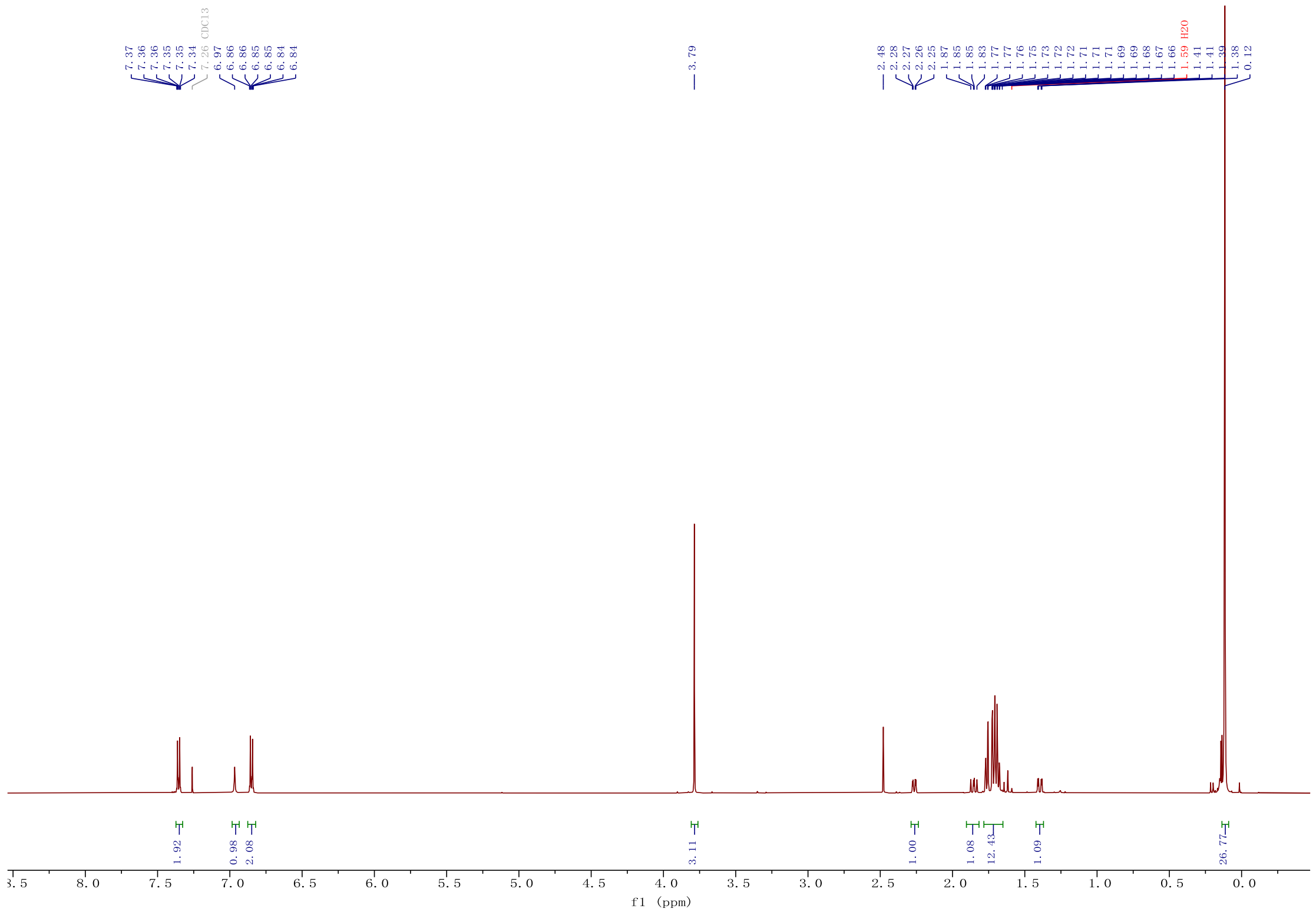

^{13}C NMR (151 MHz, $CDCl_3$)

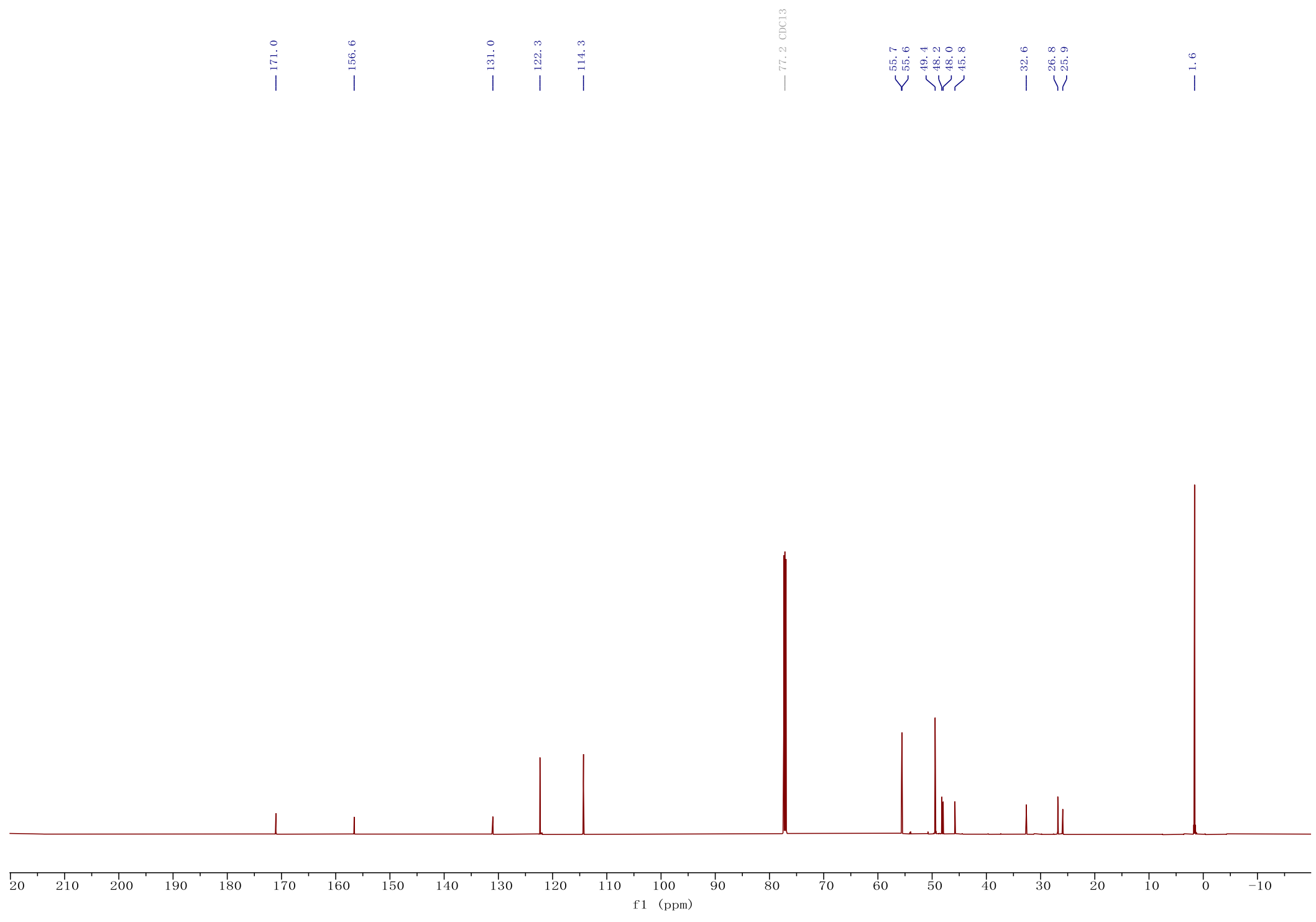

2-(bicyclo[1.1.1]pentan-1-yl)-*N*-butyl-3-(3-(1,1,1,3,3,3-hexamethyl-2-(trimethylsilyl)trisilan-2-yl)bicyclo[1.1.1]pentan-1-yl)propenamide (8h)

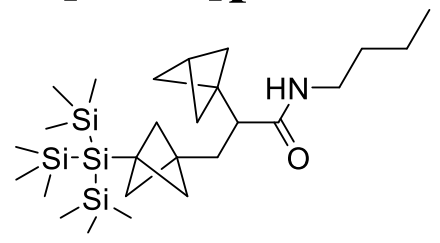

^{1}H NMR (600 MHz, CDCl$_3$)

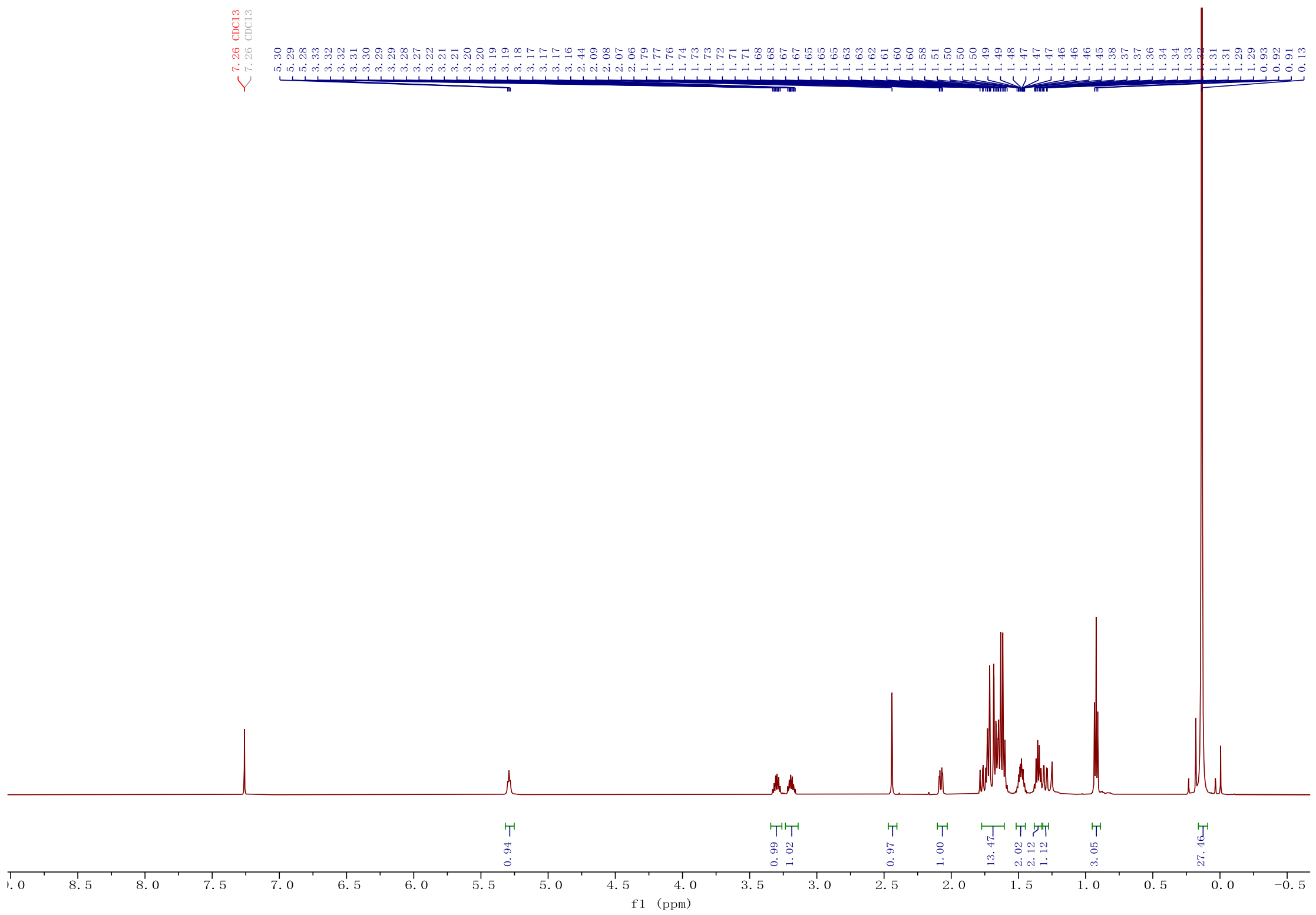

^{13}C NMR (151 MHz, CDCl$_3$)

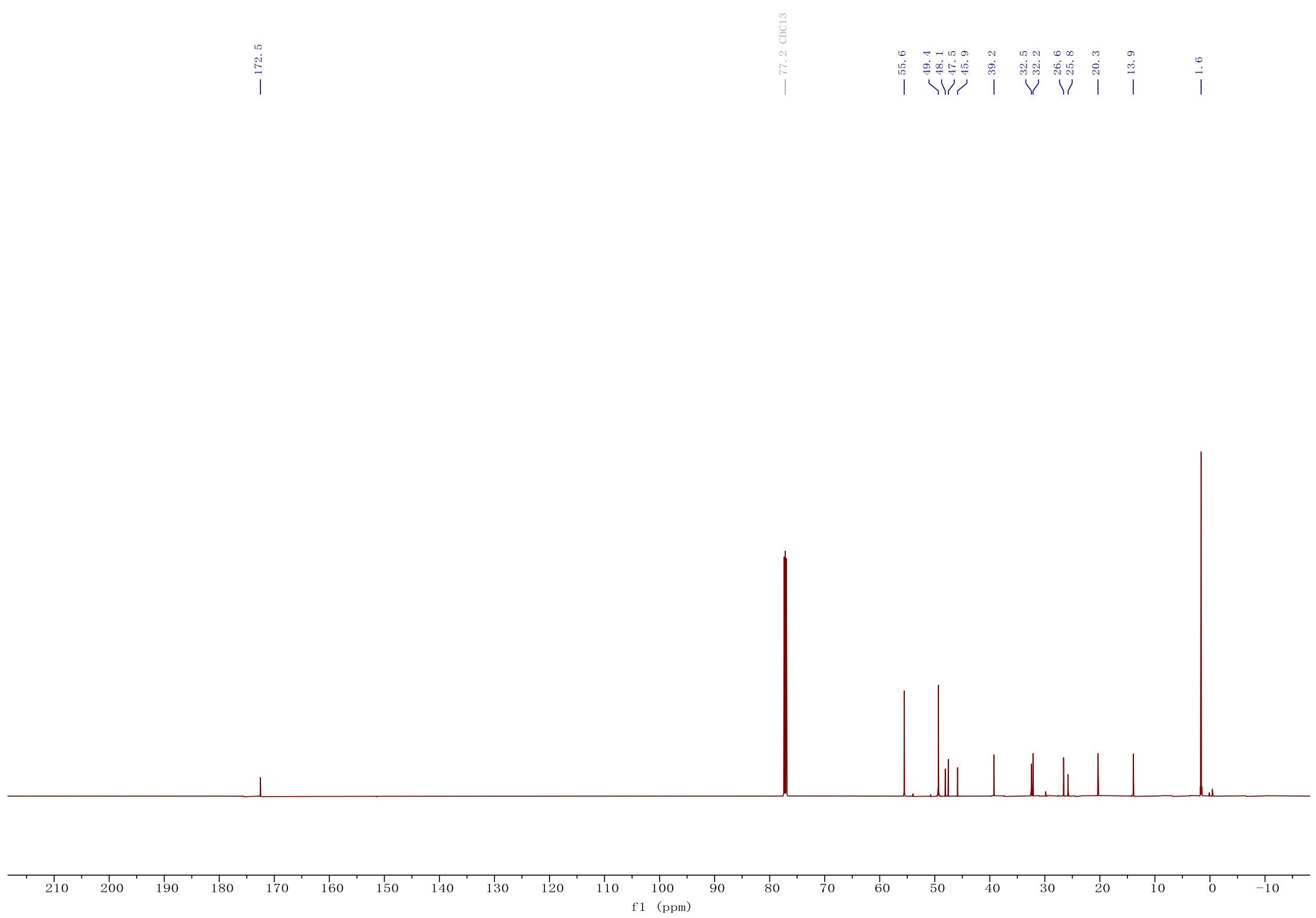

2-(bicyclo[1.1.1]pentan-1-yl)-*N*-cyclopropyl-3-(3-(1,1,1,3,3,3-hexamethyl-2-(trimethylsilyl)trisilan-2-yl)bicyclo[1.1.1]pentan-1-yl)propenamide (8i)

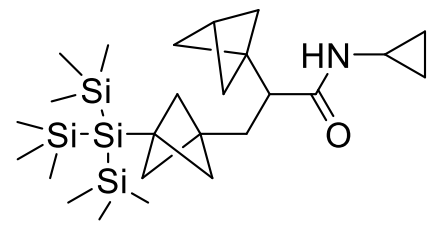

^{1}H NMR (600 MHz, CDCl$_3$)

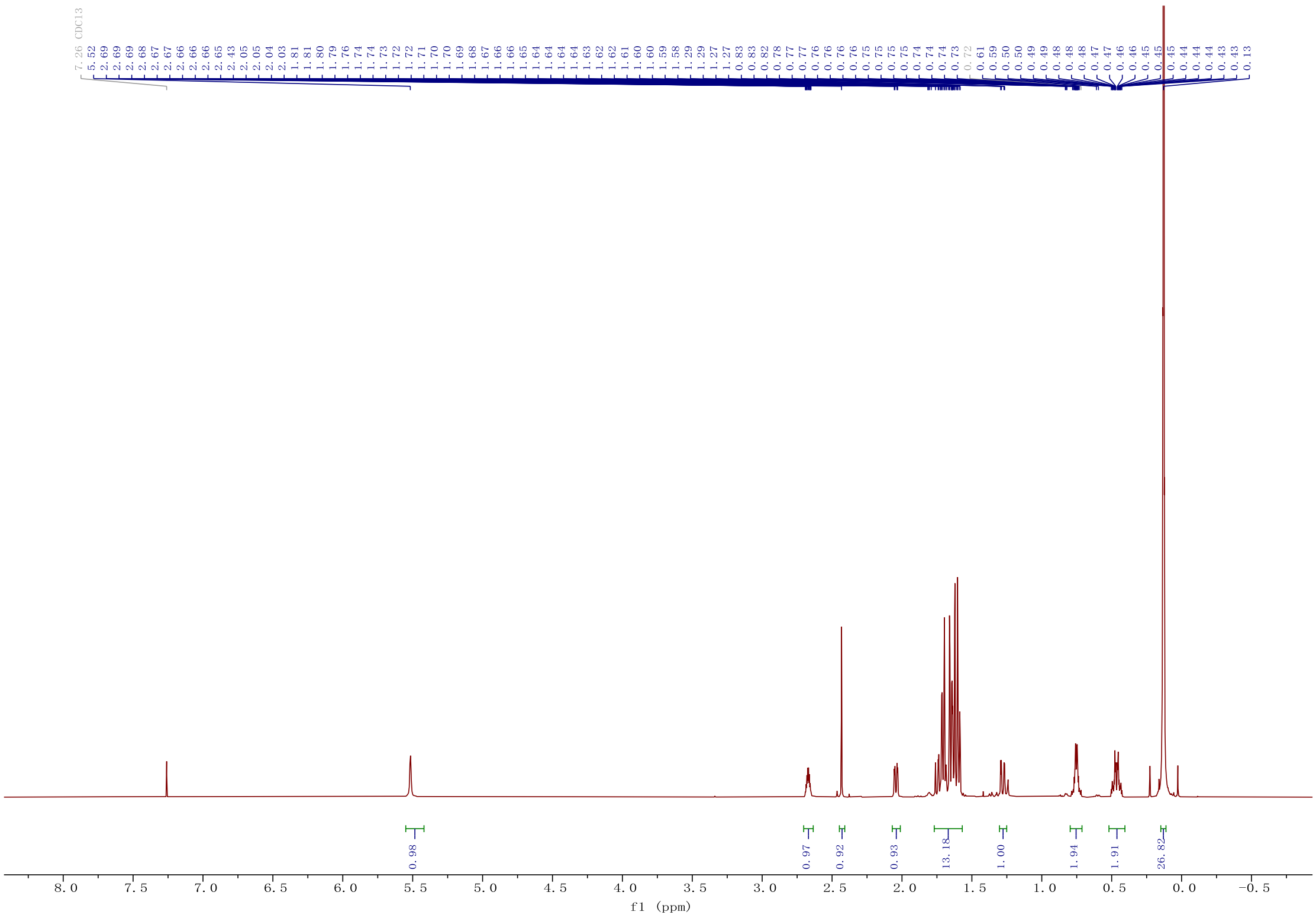

^{13}C NMR (151 MHz, CDCl$_3$)

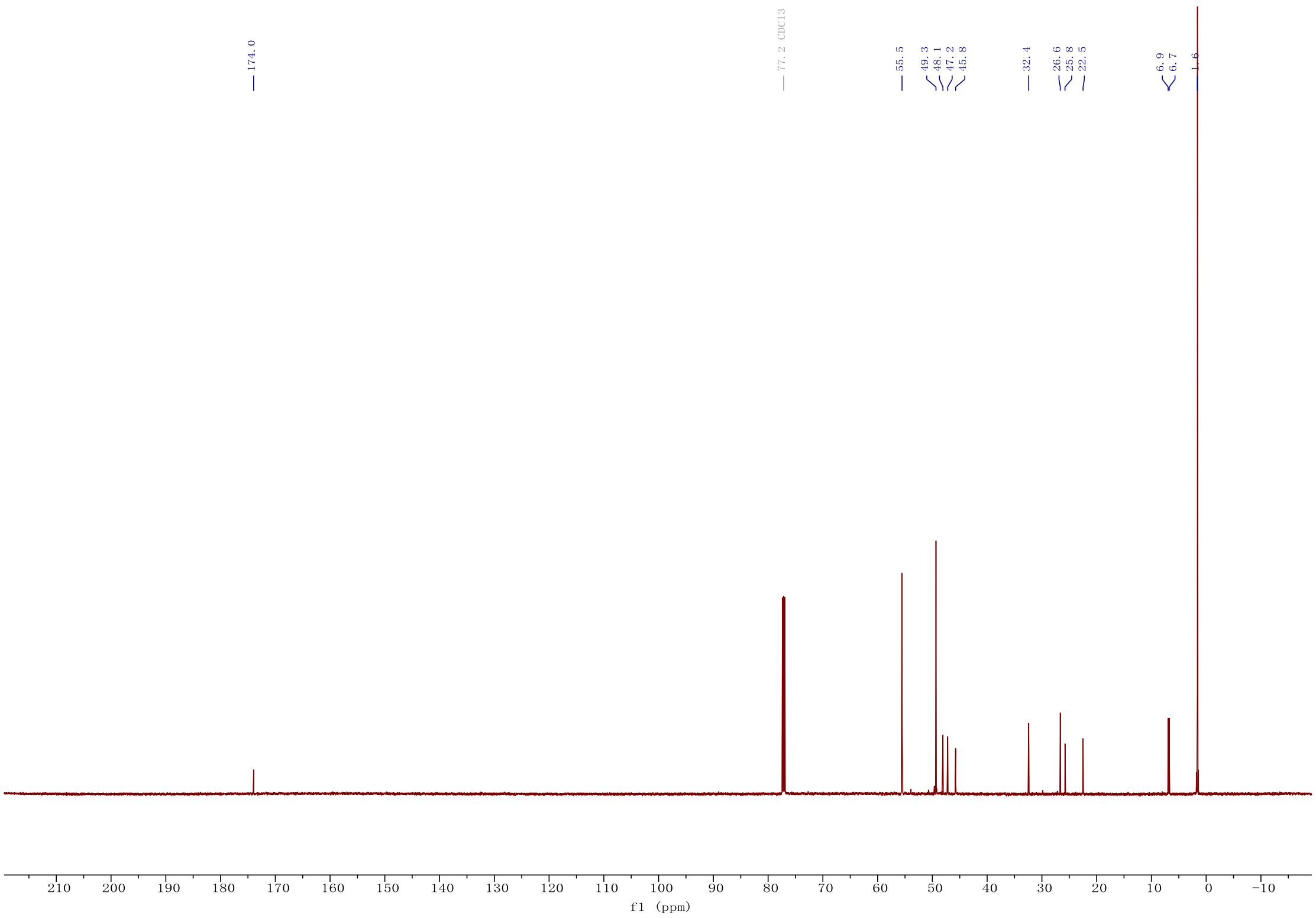

2-(bicyclo[1.1.1]pentan-1-yl)-*N*-cyclohexyl-3-(3-(1,1,1,3,3,3-hexamethyl-2-(trimethylsilyl)trisilan-2-yl)bicyclo[1.1.1]pentan-1-yl)propenamide (8j)

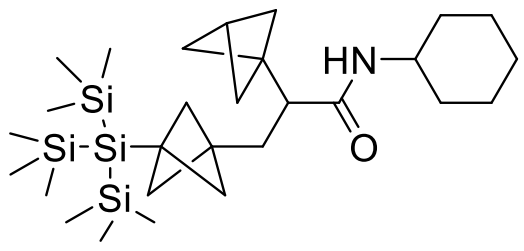

^{1}H NMR (600 MHz, CDCl3)

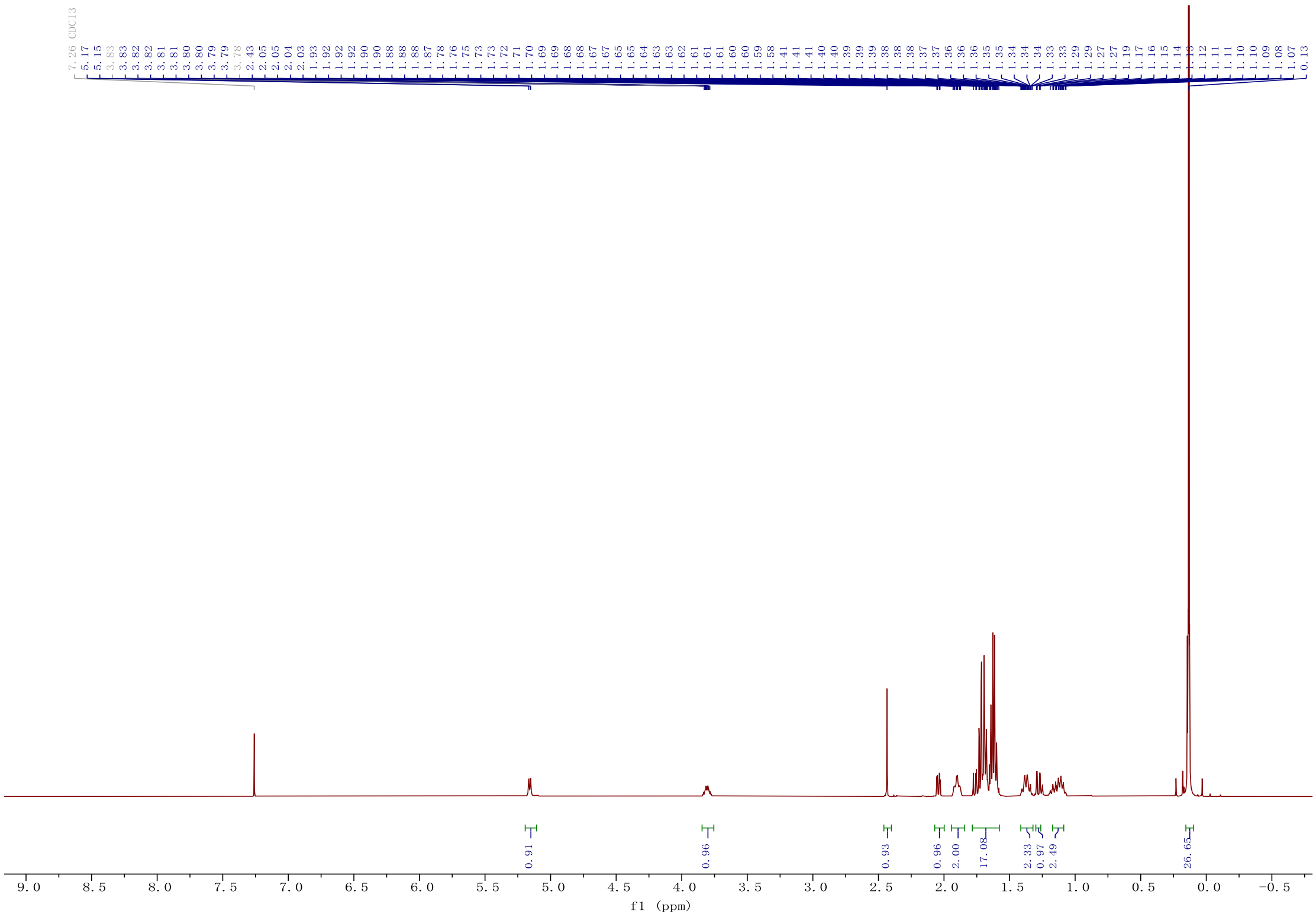

^{13}C NMR (151 MHz, CDCl3)

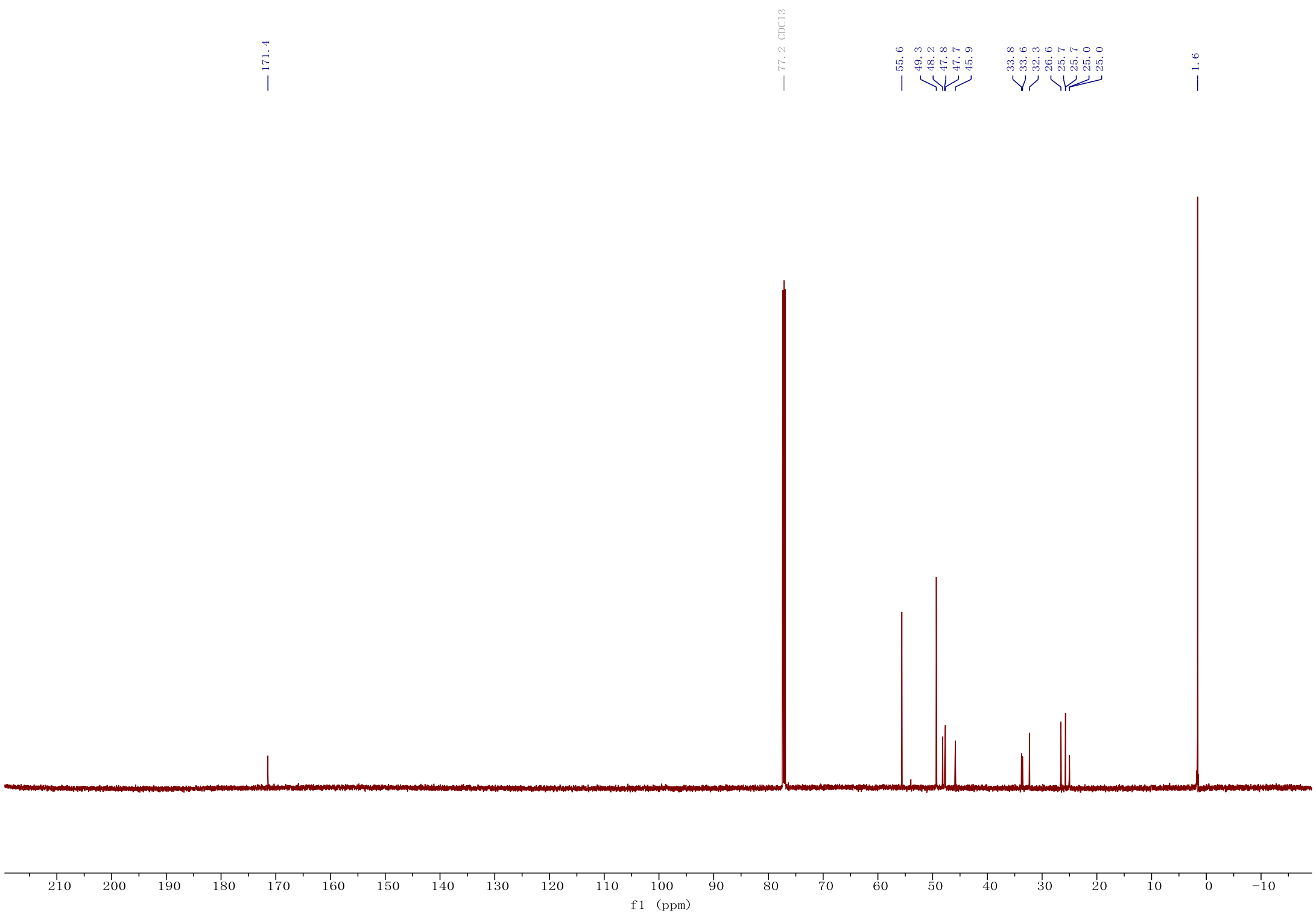

2-(bicyclo[1.1.1]pentan-1-yl)-3-(3-(1,1,1,3,3,3-hexamethyl-2-(trimethylsilyl)trisilan-2-yl)bicyclo[1.1.1]pentan-1-yl)-*N*-(2-methyl-4-oxopentan-2-yl)propenamide (8k)

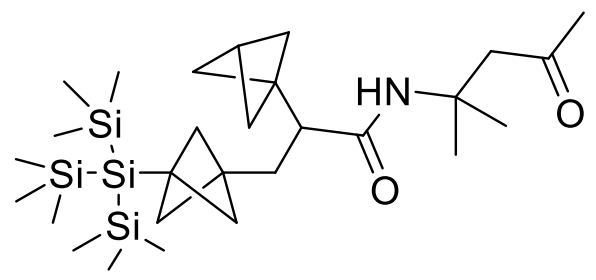

^{1}H NMR (600 MHz, CDCl$_3$)

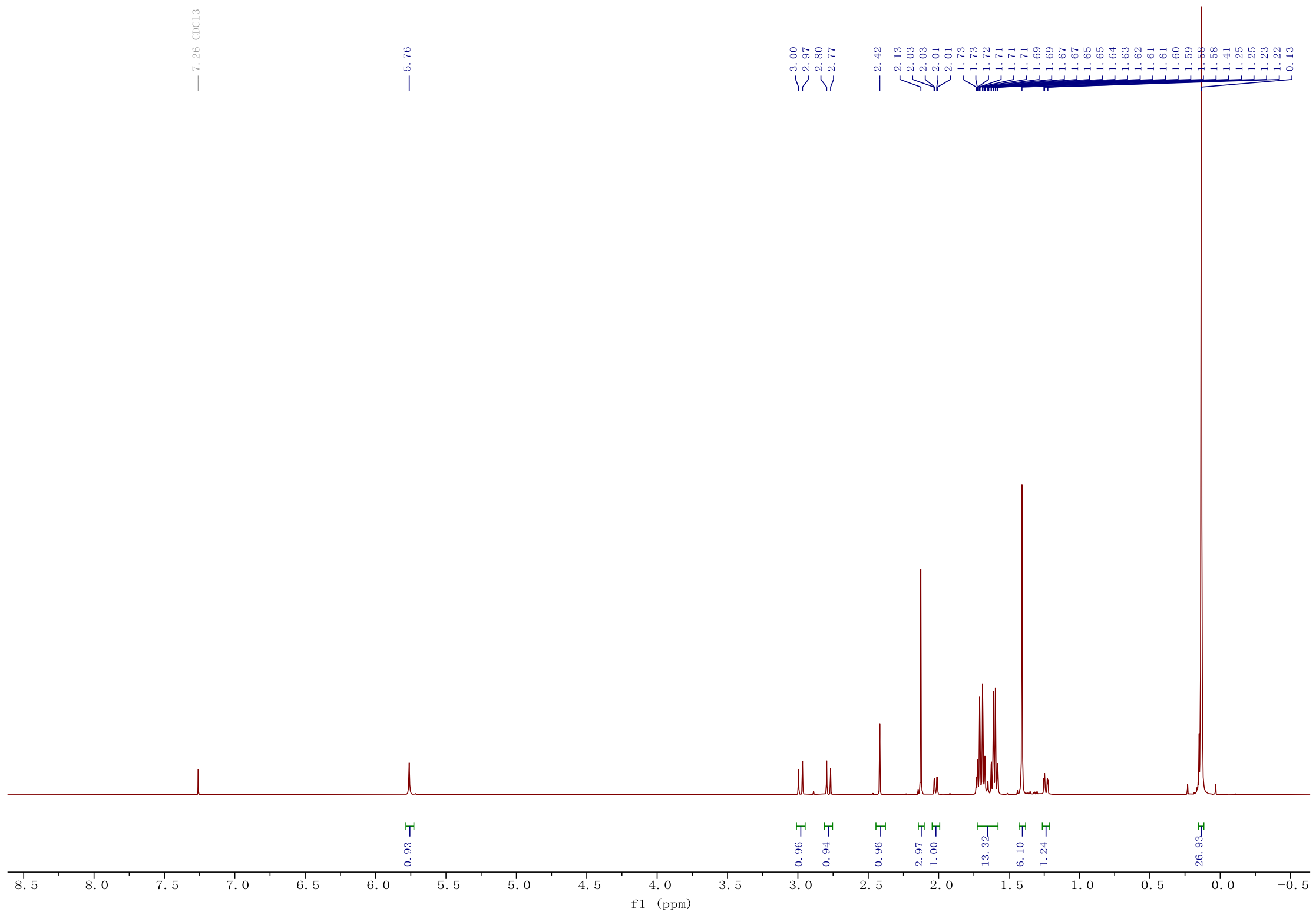

^{13}C NMR (151 MHz, CDCl$_3$)

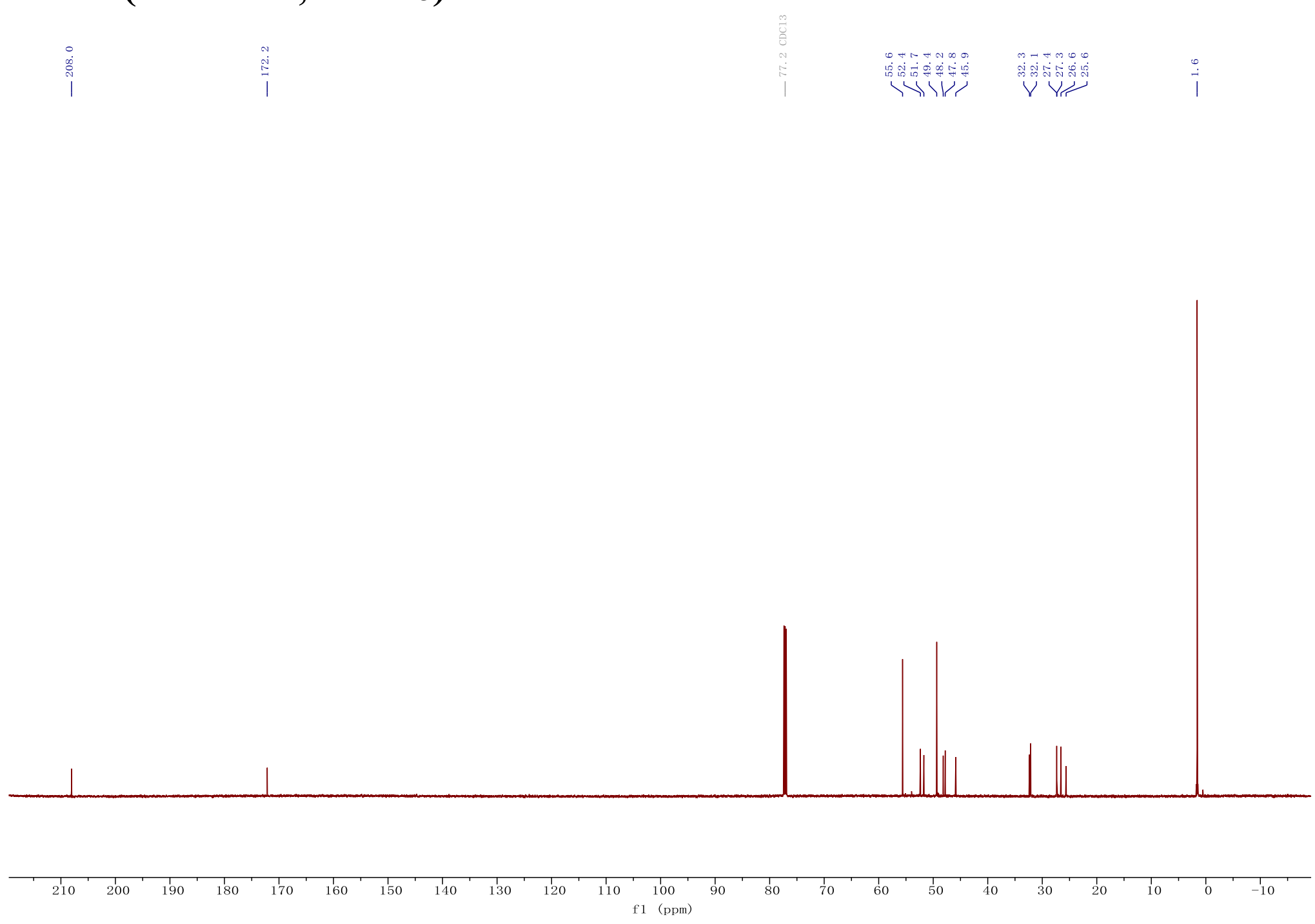

2-(bicyclo[1.1.1]pentan-1-yl)-3-(3-(1,1,1,3,3,3-hexamethyl-2-(trimethylsilyl)trisilan-2-yl)bicyclo[1.1.1]pentan-1-yl)-1-morpholinopropan-1-one (8l)

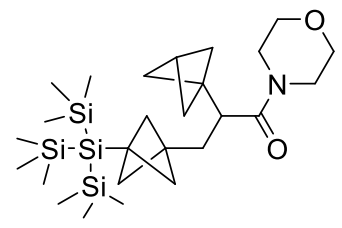

^{1}H NMR (600 MHz, CDCl$_3$)

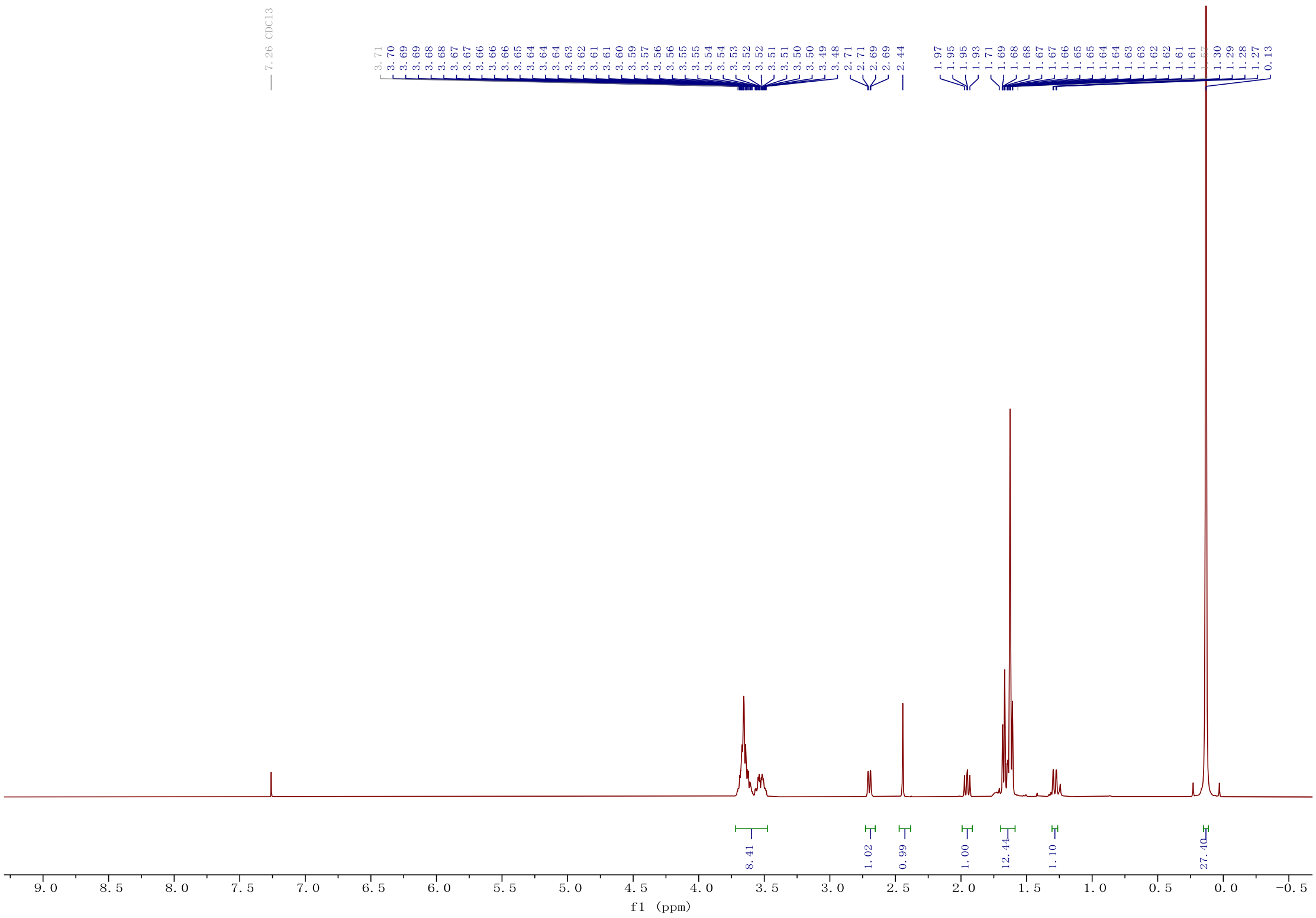

^{13}C NMR (151 MHz, CDCl$_3$)

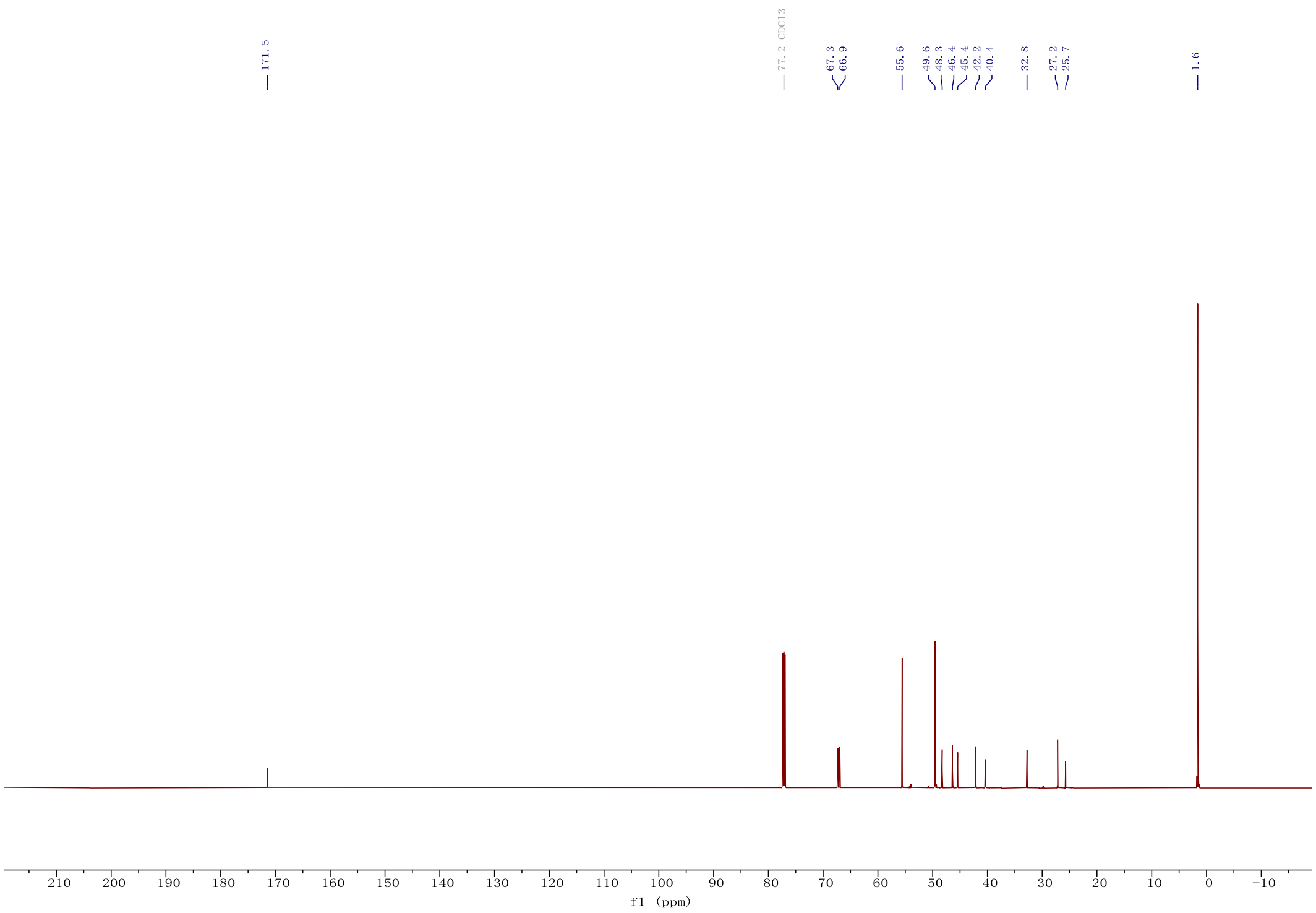

2-(bicyclo[1.1.1]pentan-1-yl)-3-(3-(1,1,1,3,3,3-hexamethyl-2-(trimethylsilyl)trisilan-2-yl)bicyclo[1.1.1]pentan-1-yl)-2-methyl-*N*-phenylpropanamide (8m)

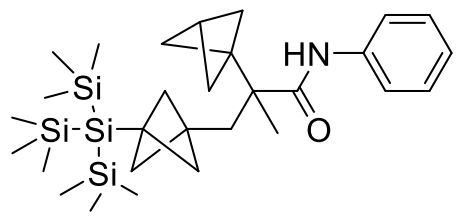

^{1}H NMR (600 MHz, $CDCl_3$)

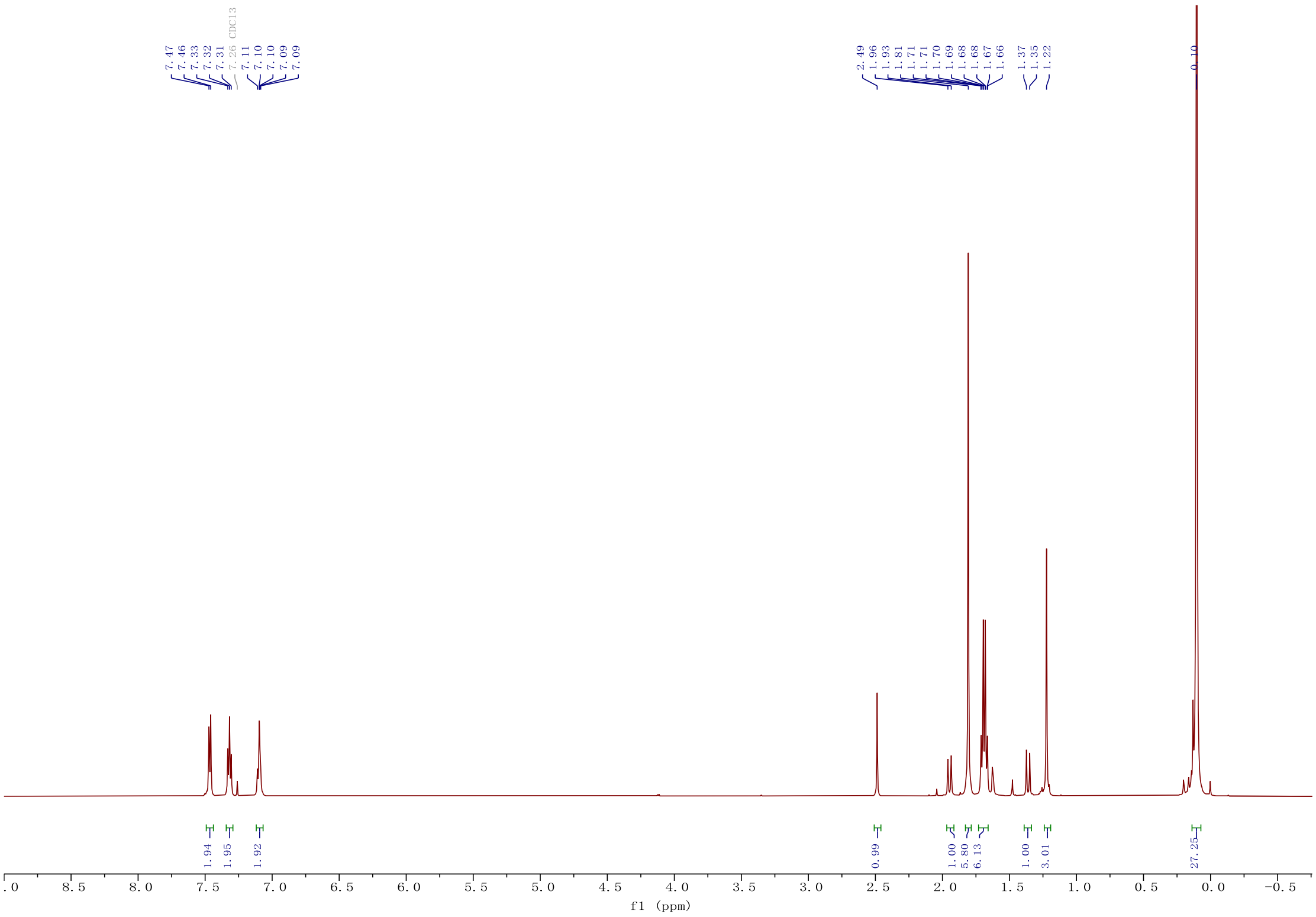

^{13}C NMR (151 MHz, $CDCl_3$)

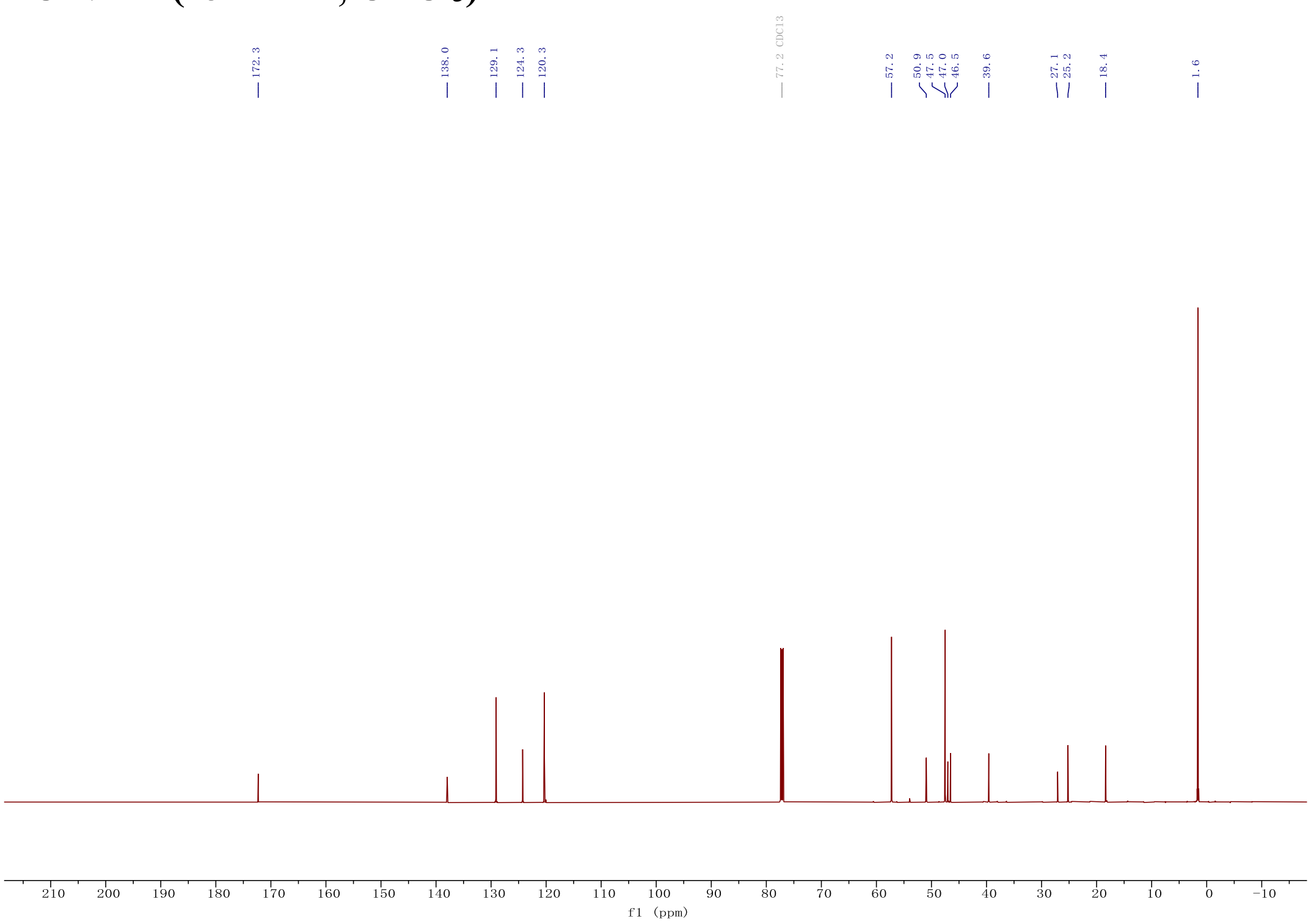

2-(bicyclo[1.1.1]pentan-1-yl)-3-(3-(1,1,1,3,3,3-hexamethyl-2-(trimethylsilyl)trisilan-2-yl)bicyclo[1.1.1]pentan-1-yl)-*N*-(4-methoxyphenyl)-2-methylpropanamide (8n)

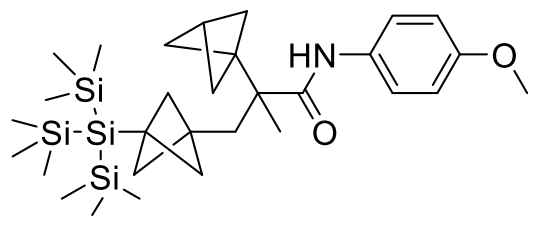

^{1}H NMR (600 MHz, CDCl3)

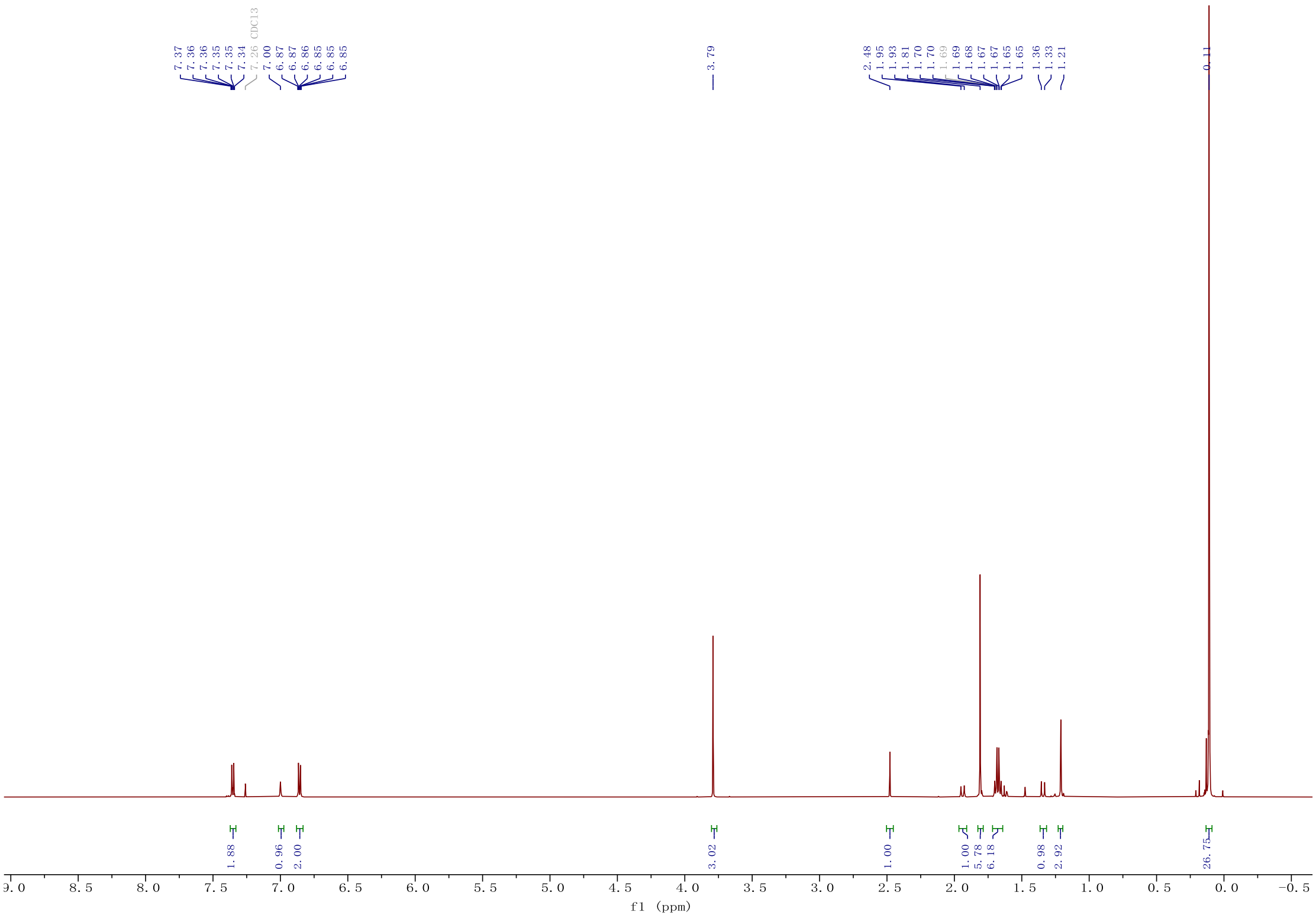

^{13}C NMR (151 MHz, CDCl3)

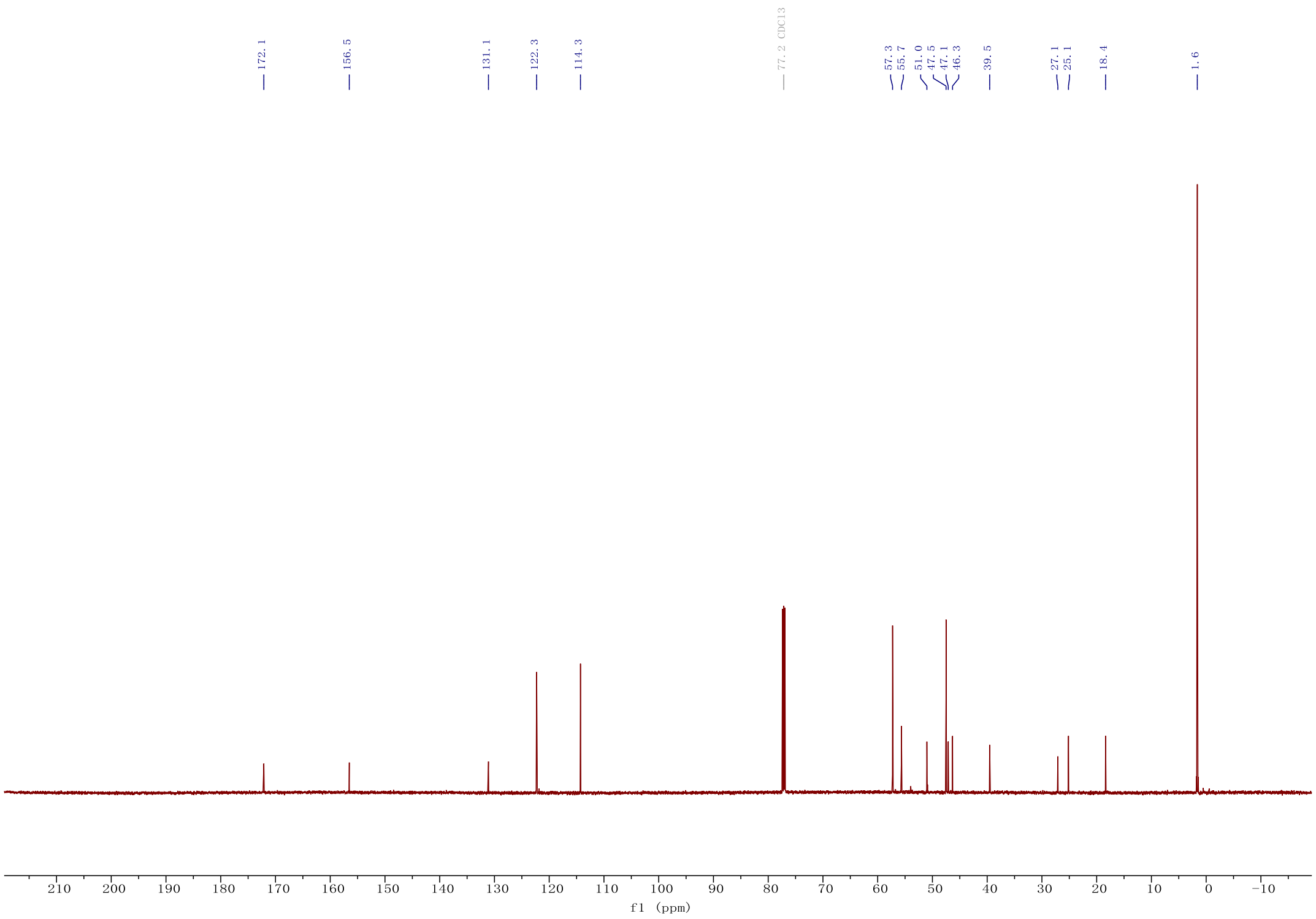

methyl (2-(bicyclo[1.1.1]pentan-1-yl)-3-(3-(1,1,1,3,3,3-hexamethyl-2-(trimethylsilyl)trisilan-2-yl)bicyclo[1.1.1]pentan-1-yl)propanoyl)-*L*-phenylalanyl-*L*-leucinate (8o)

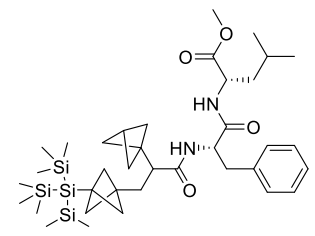

^{1}H NMR (600 MHz, CDCl$_3$)

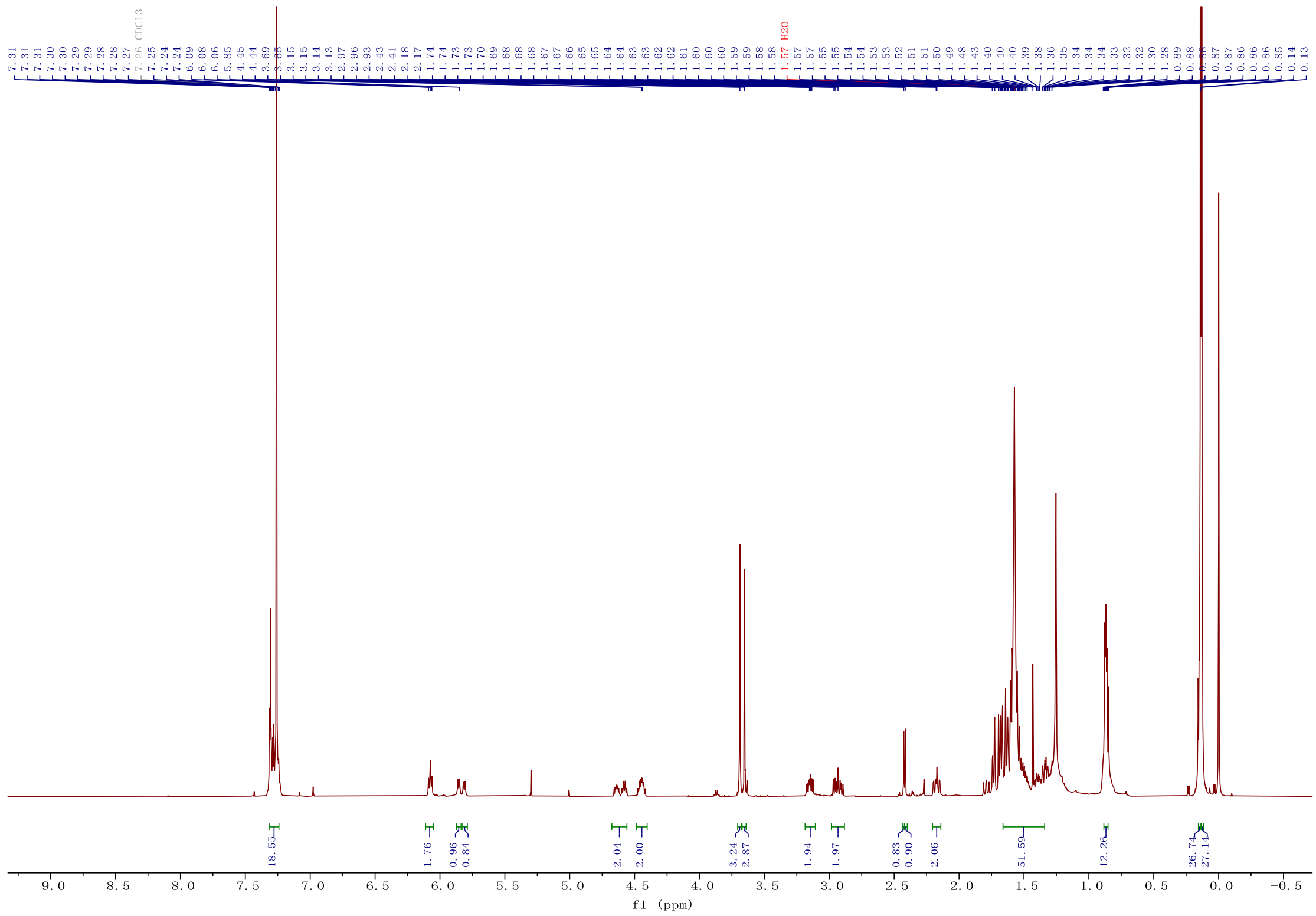

^{13}C NMR (151 MHz, CDCl$_3$)

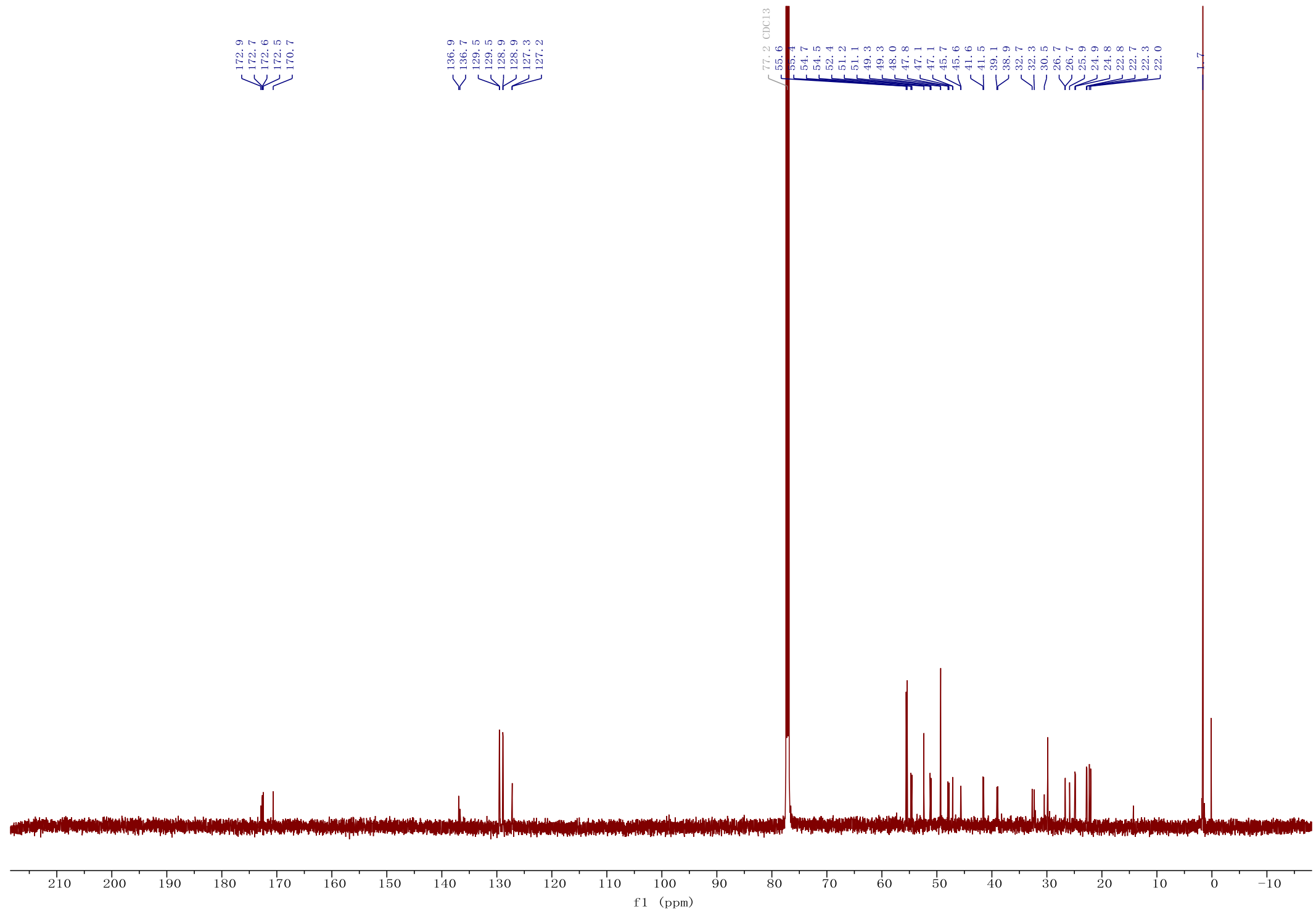

(*Z*)-5-(bicyclo[1.1.1]pentan-1-yl)-2-((3-(dichloromethyl)bicyclo[1.1.1]pentan-1-yl)methyl)-*N*-phenylpent-2-enamide (3ad)

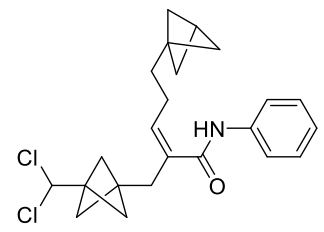

^{1}H NMR (600 MHz, $CDCl_3$)

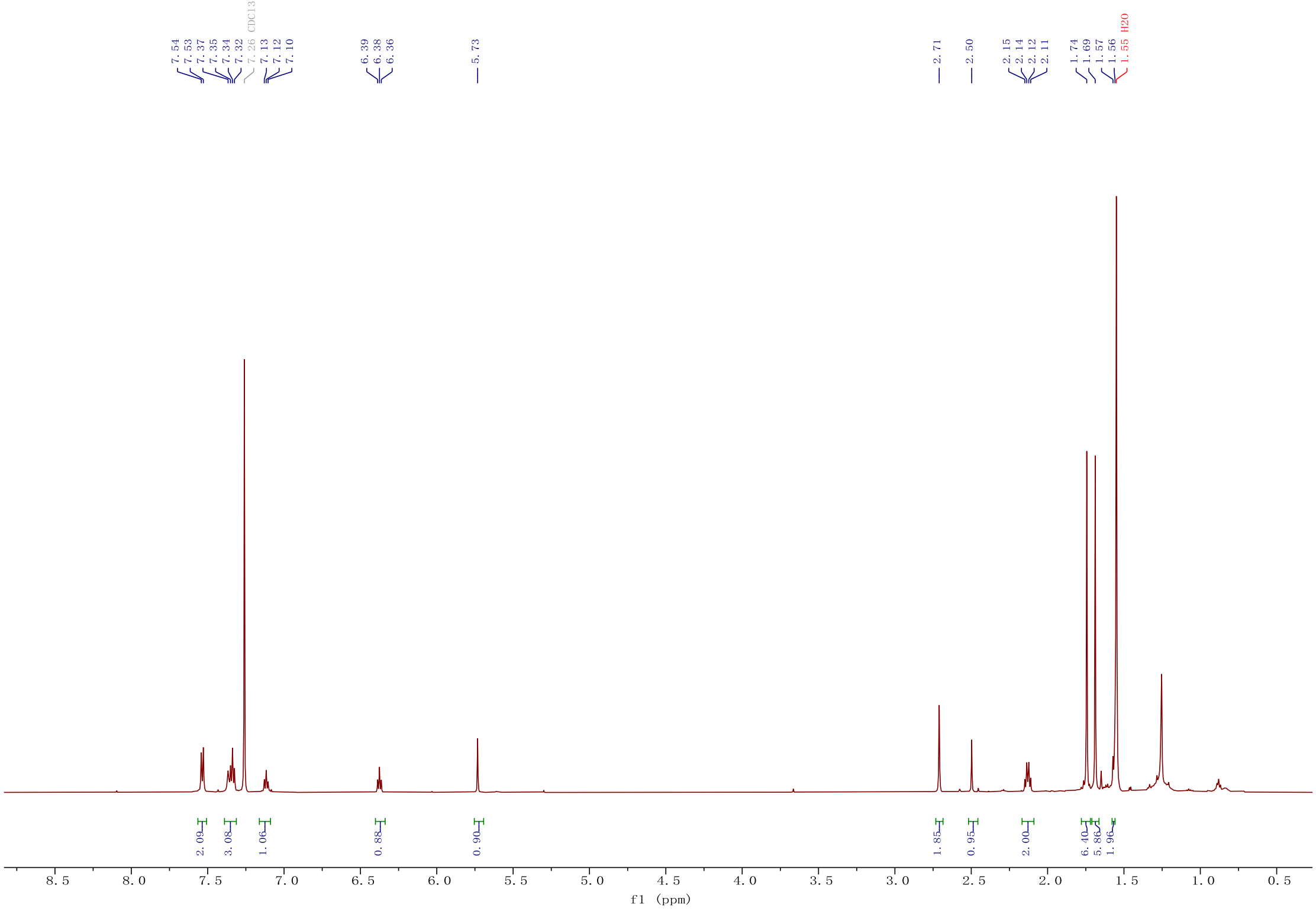

^{13}C NMR (151 MHz, $CDCl_3$)

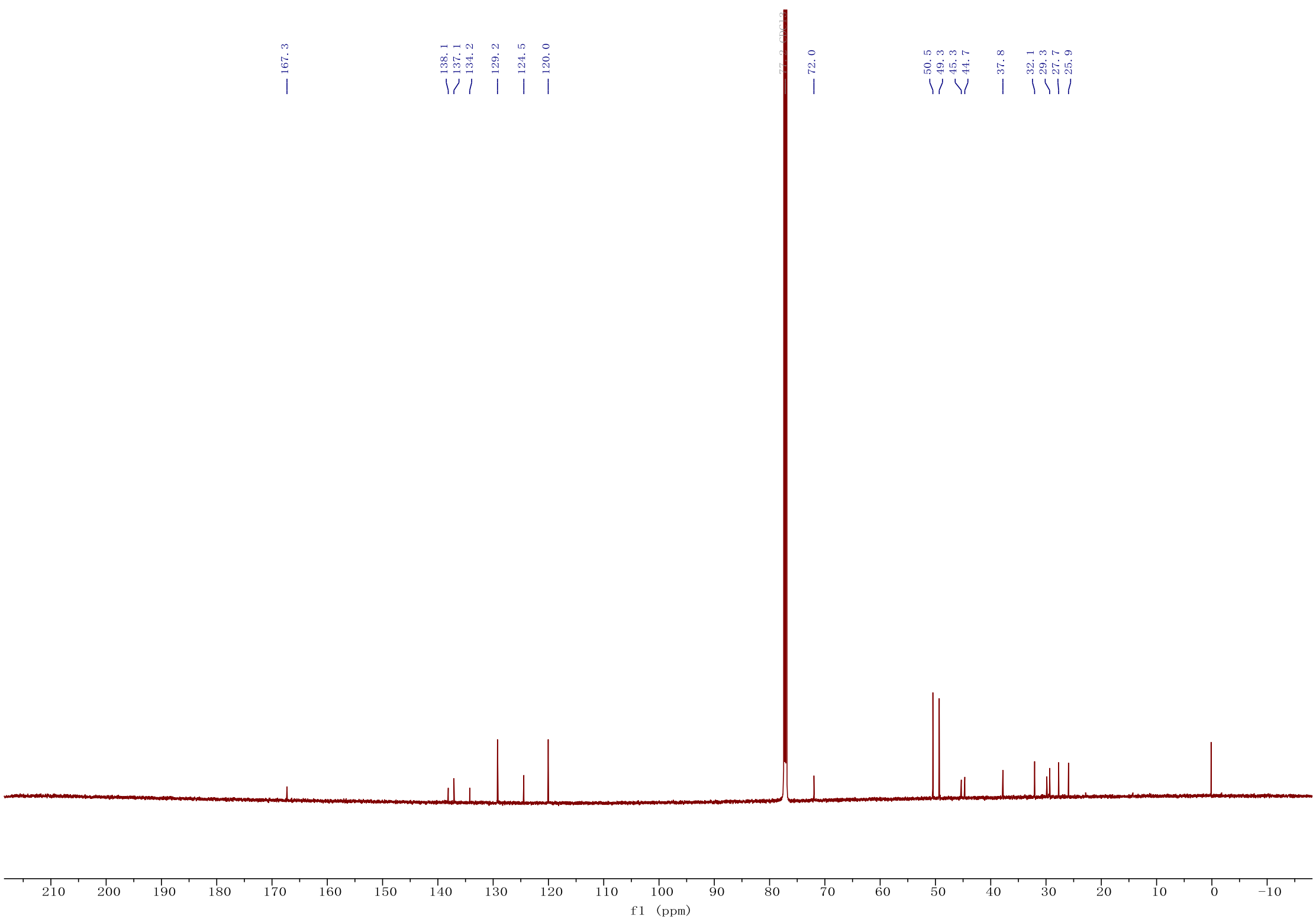

2-([1,1'-bi(bicyclo[1.1.1]pentan)]-3-yl)-3-(3-(dichloromethyl)bicyclo[1.1.1]pentan-1-yl)-*N*-phenylpropanamide (3a’)

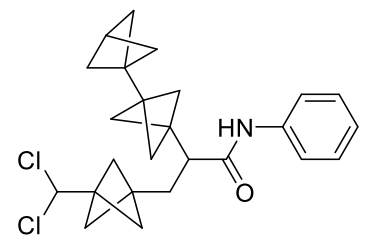

^{1}H NMR (600 MHz, CDCl$_3$)

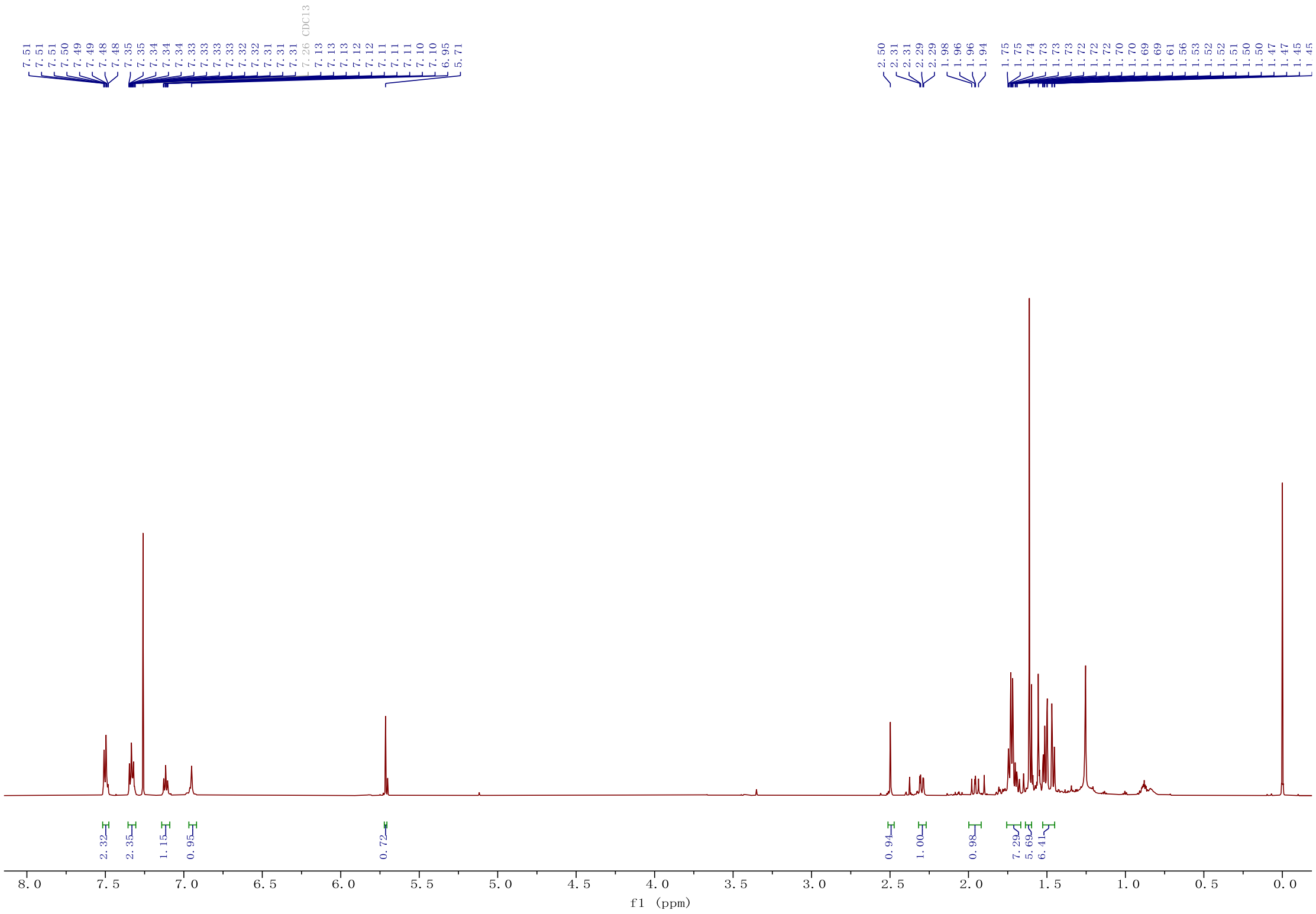

^{13}C NMR (151 MHz, CDCl$_3$)

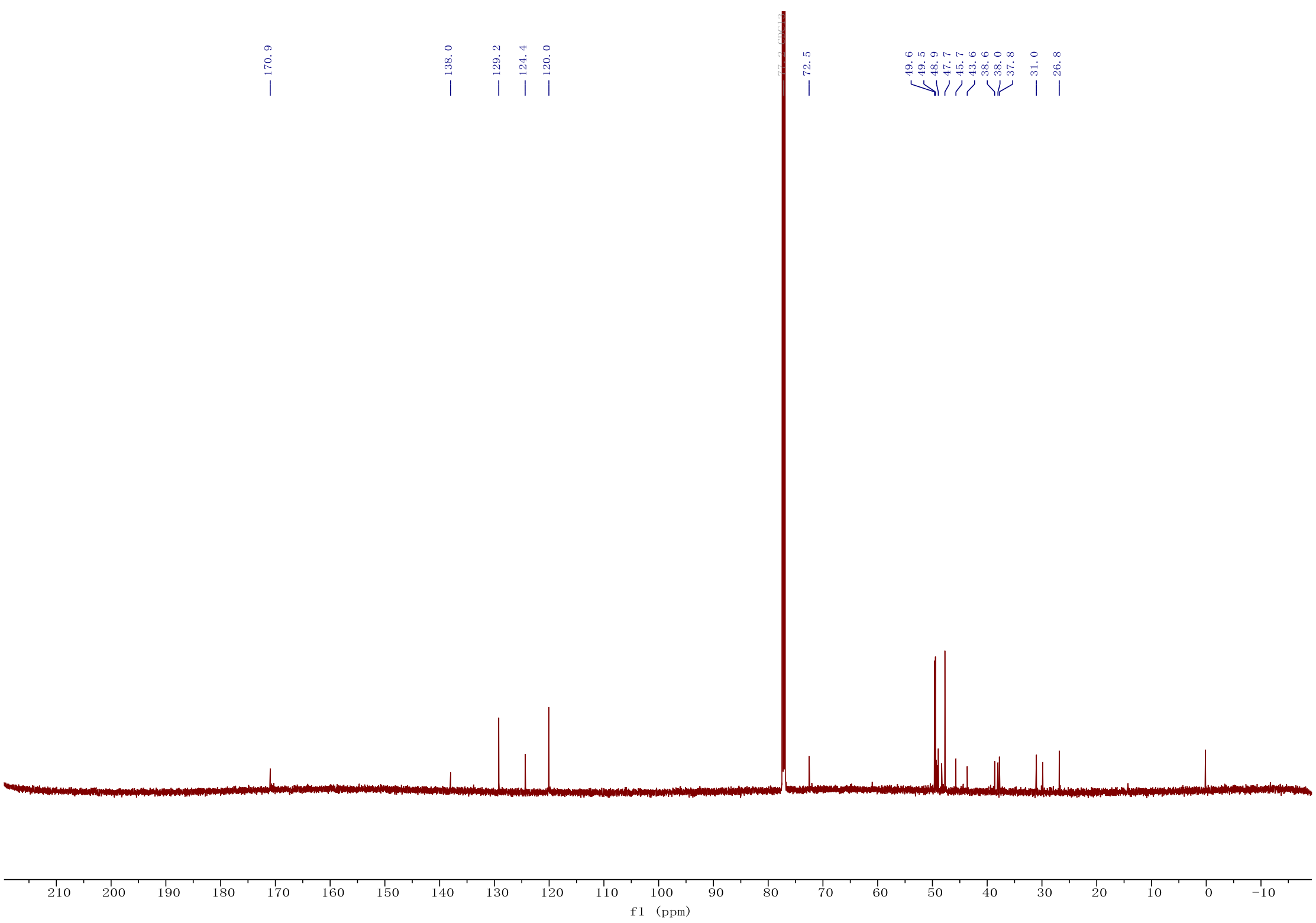

(*Z*)-5-(bicyclo[1.1.1]pentan-1-yl)-*N*-phenyl-2-((3-(trichloromethyl)bicyclo[1.1.1]pentan-1-yl)methyl)pent-2-enamide (4ab)

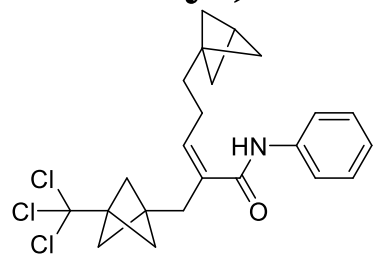

^{1}H NMR (600 MHz, $CDCl_3$)

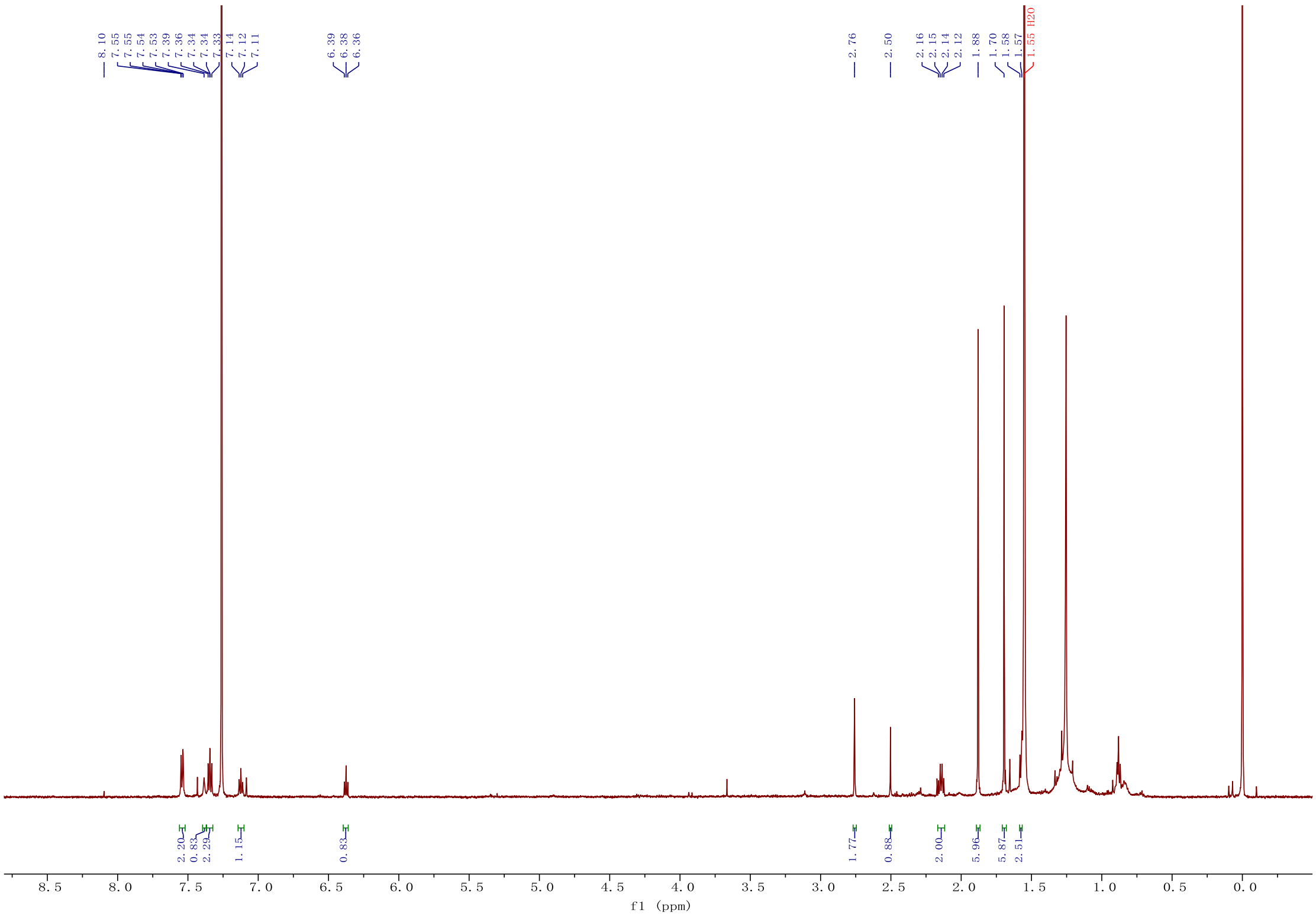

^{13}C NMR (151 MHz, $CDCl_3$)

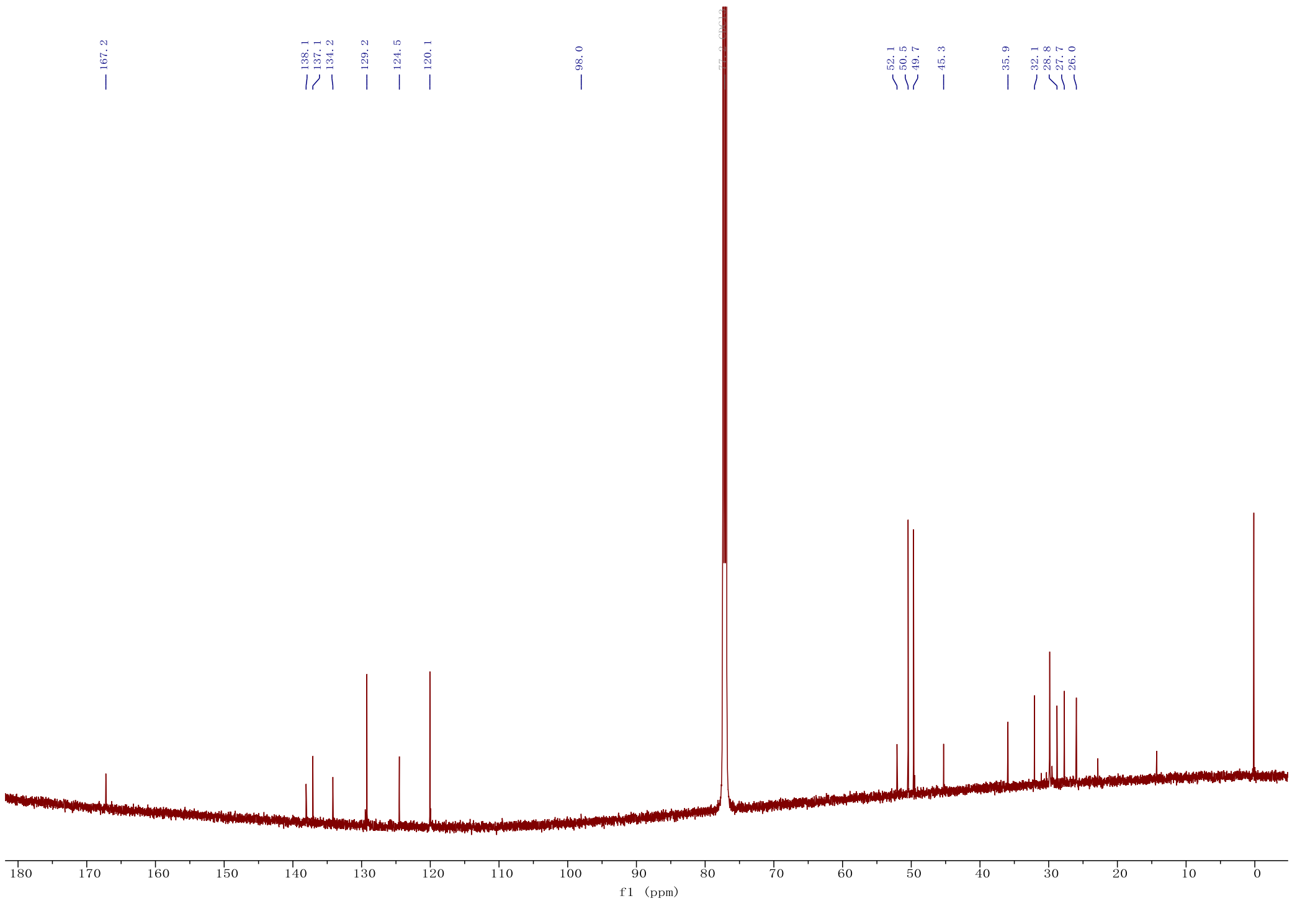

2-(bicyclo[1.1.1]pentan-1-yl-3-d)-*N*-phenyl-3-(3-(trichloromethyl)bicyclo[1.1.1]pentan-1-yl)propenamide (4ab)

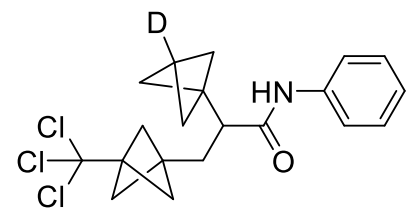

^{1}H NMR (600 MHz, $CDCl_3$)

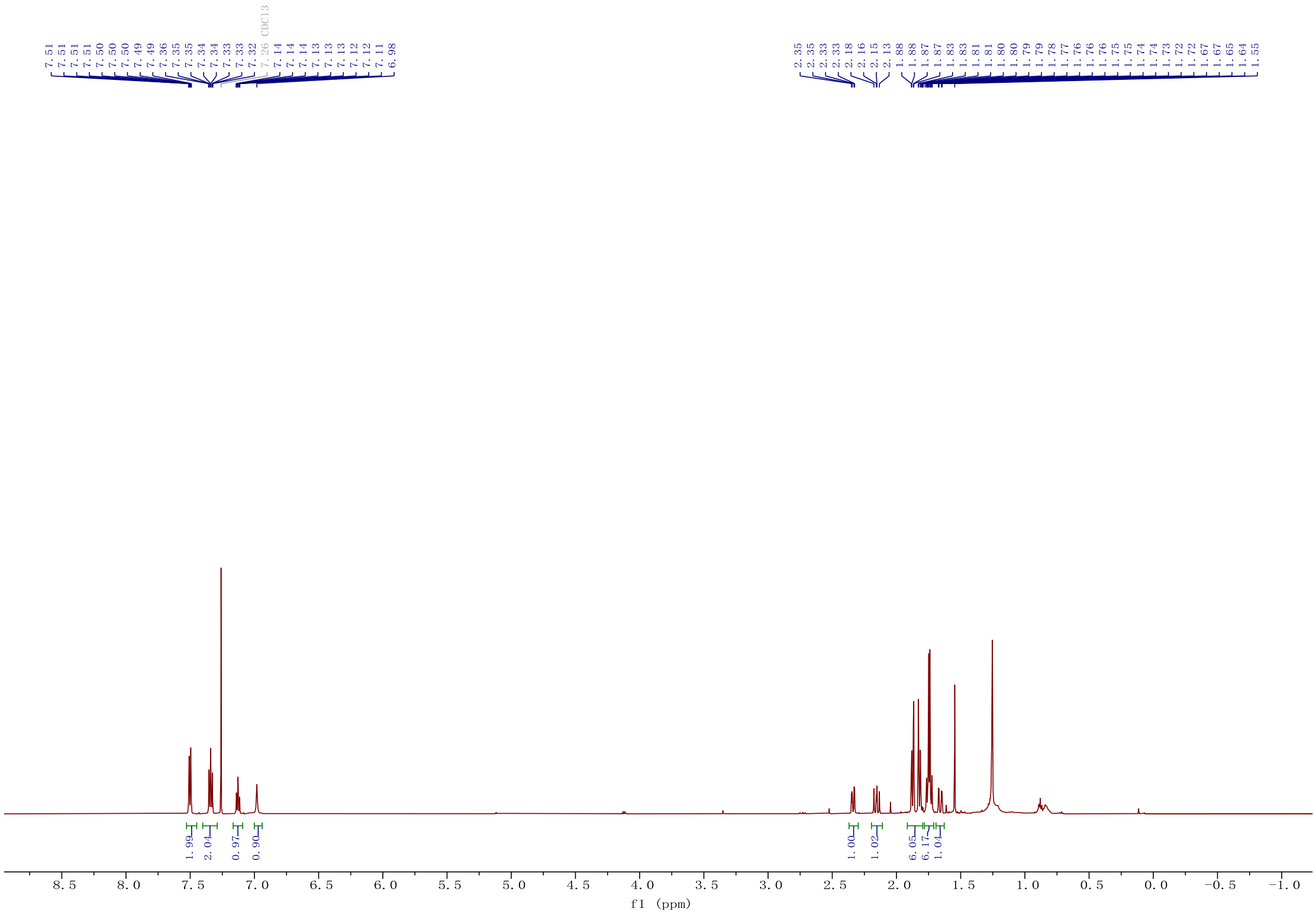

^{13}C NMR (151 MHz, $CDCl_3$)

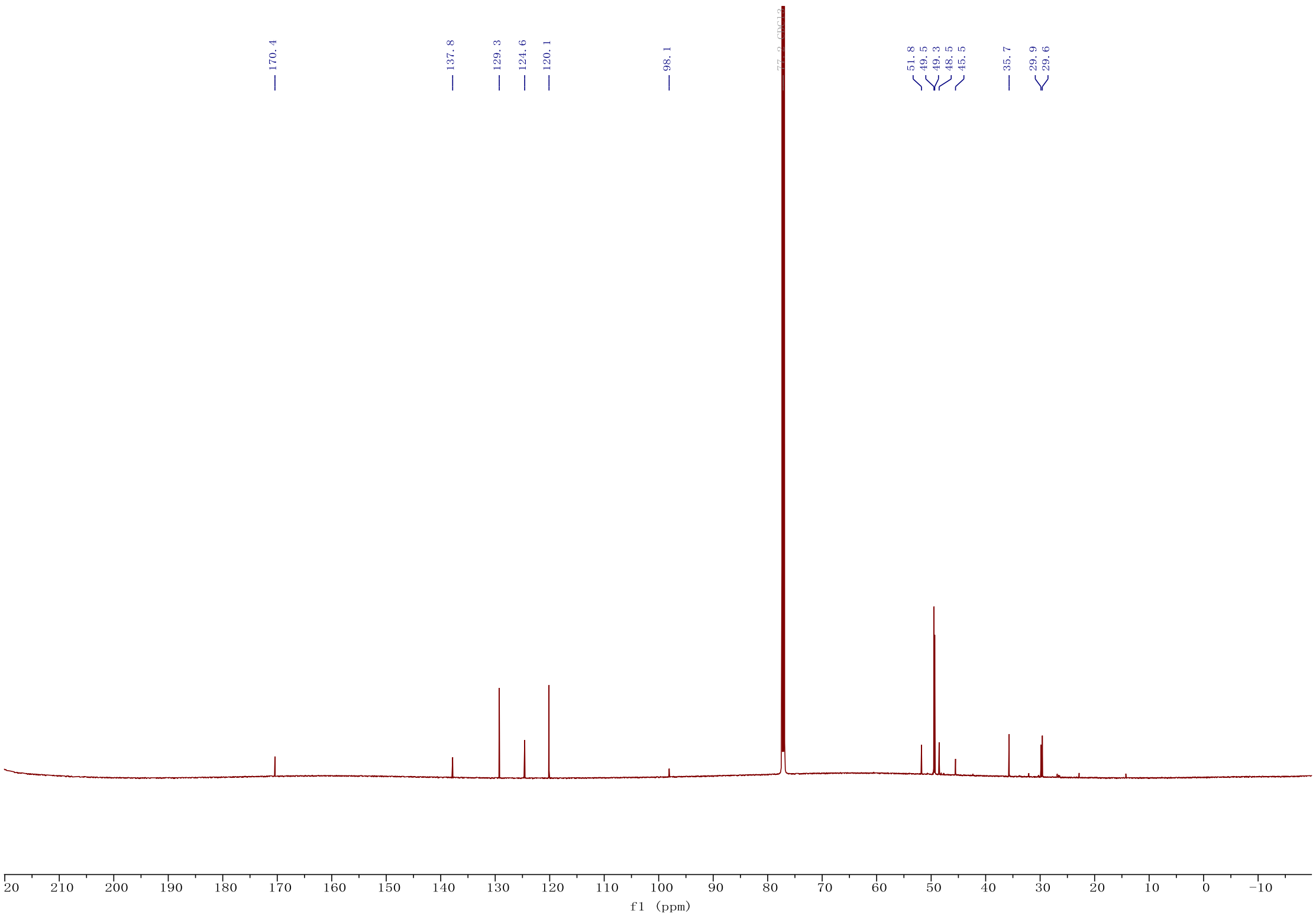

N-(2-(bicyclo[1.1.1]pentan-1-yl)-3-(3-(dichloromethyl)bicyclo[1.1.1]pentan-1-yl)propyl)aniline (3ad)

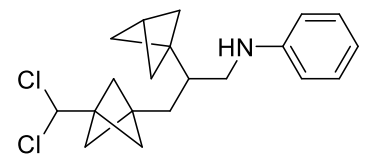

^{1}H NMR (600 MHz, $CDCl_3$)

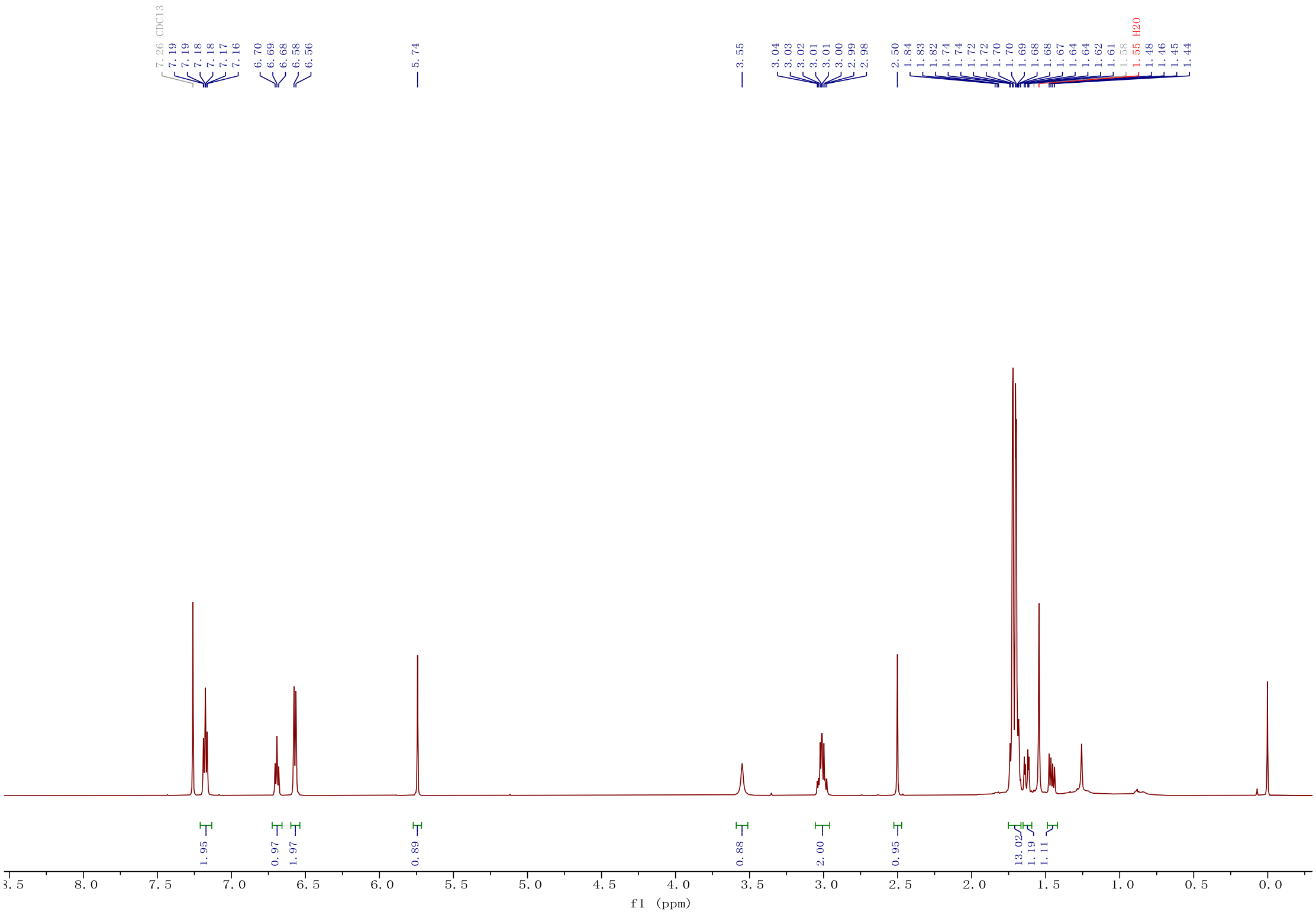

^{13}C NMR (151 MHz, $CDCl_3$)

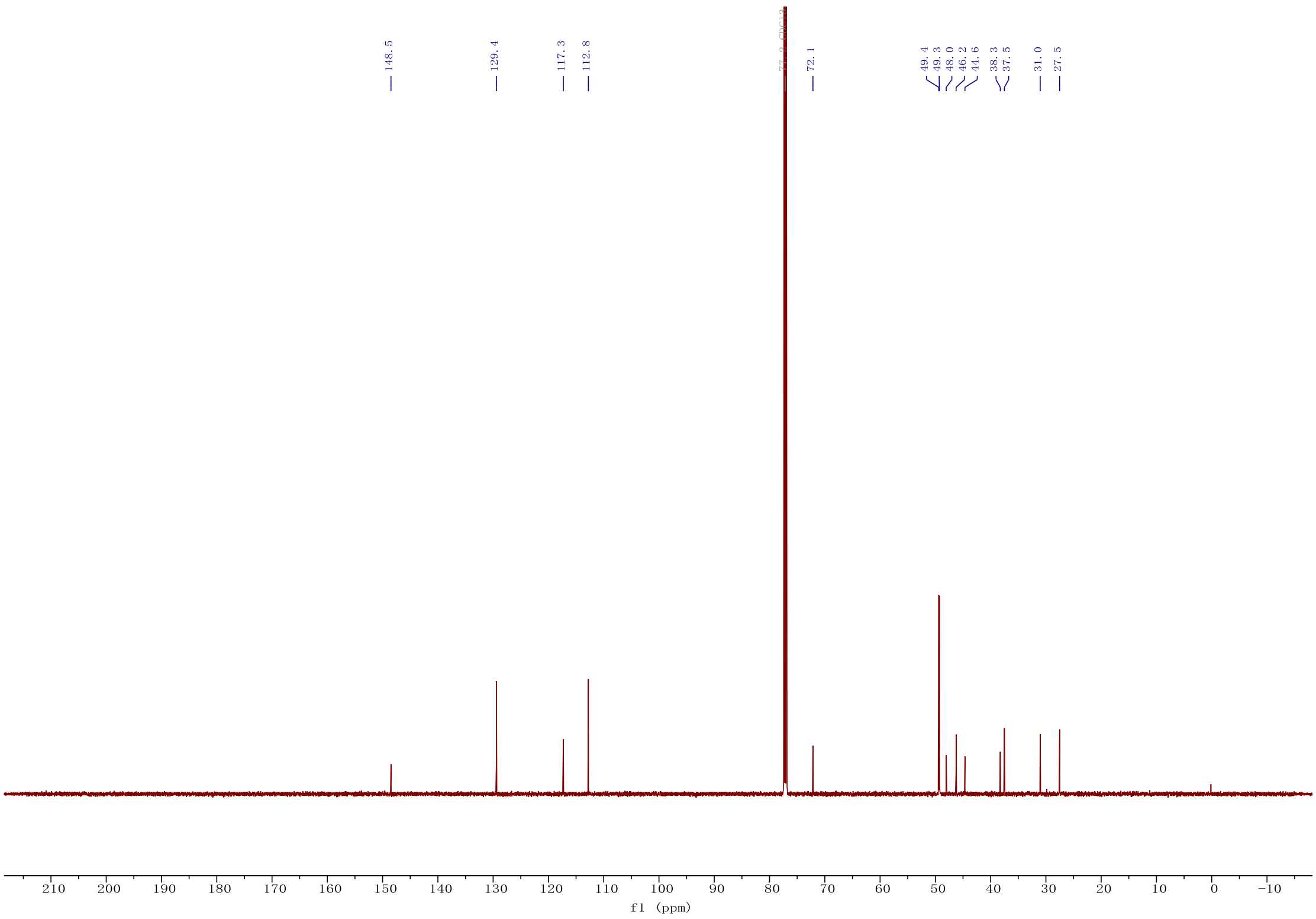

3-(3-methylbicyclo[1.1.1]pentan-1-yl)-2-(3-methylenecyclobutyl)-*N*-phenylpropanamide (3ae)

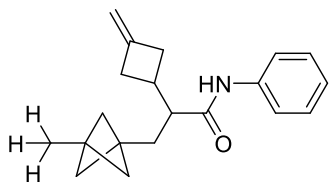

^{1}H NMR (600 MHz, CDCl$_3$)

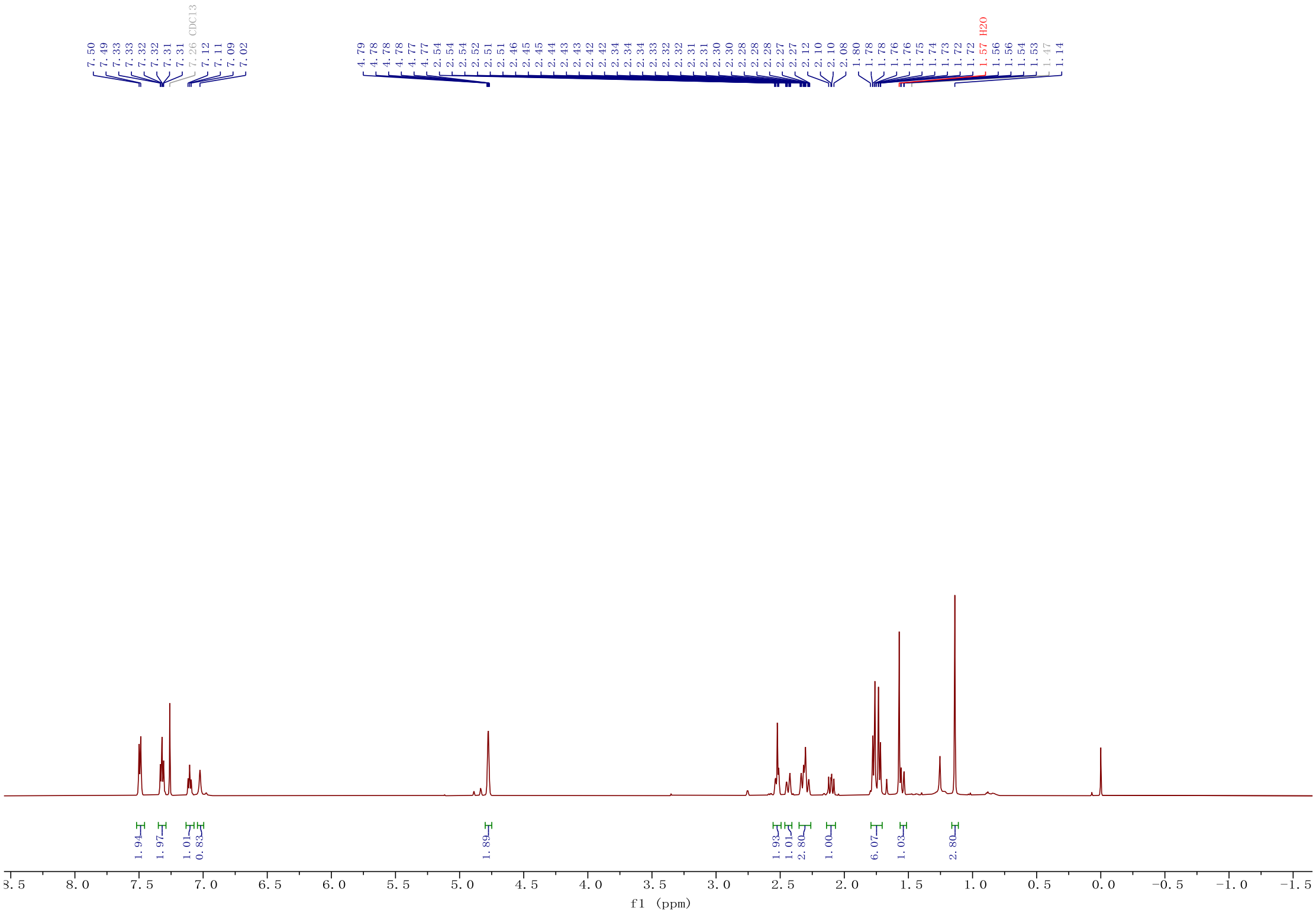

^{13}C NMR (151 MHz, CDCl$_3$)

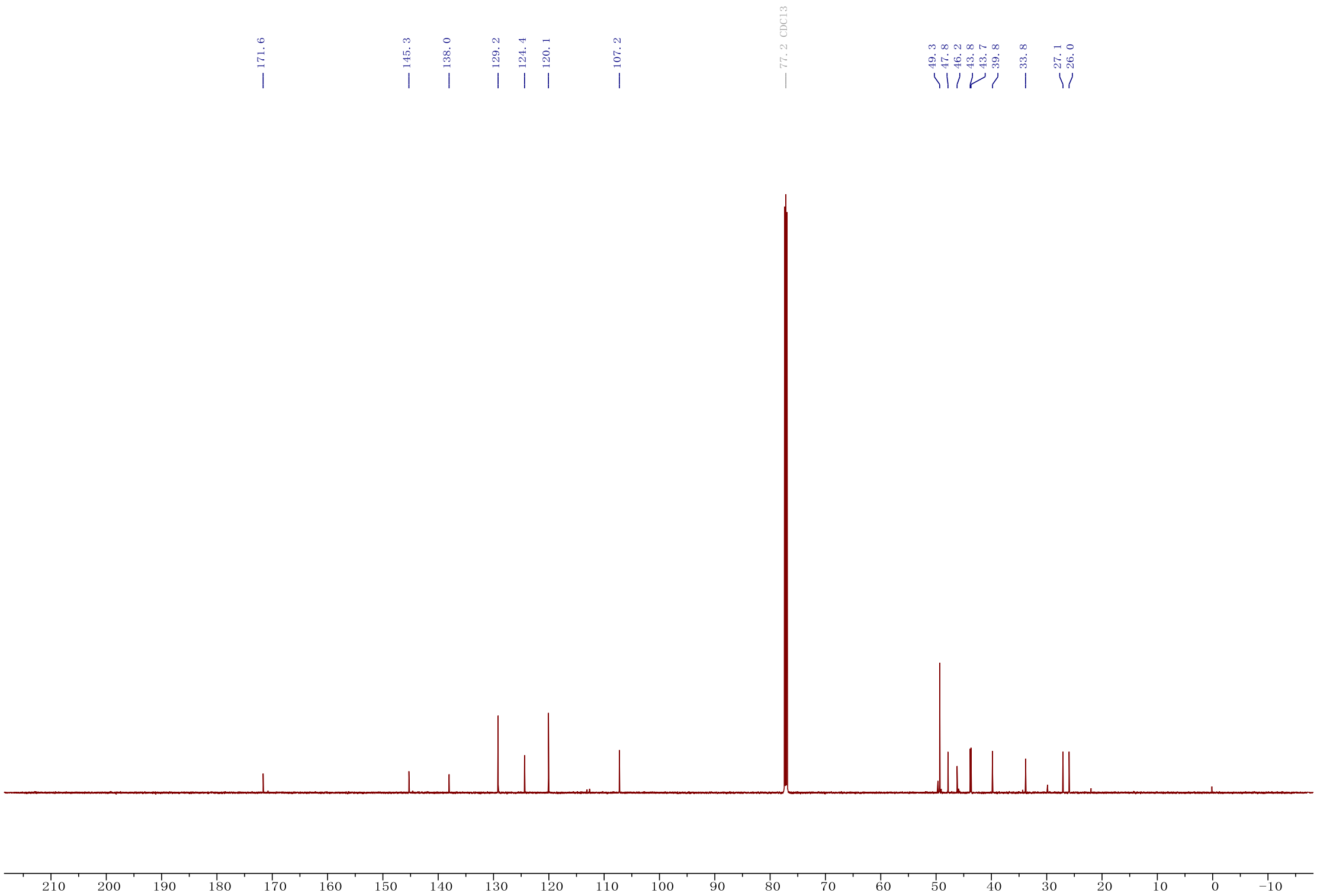

(*Z*)-5-(bicyclo[1.1.1]pentan-1-yl)-2-((3-(1,1,1,3,3,3-hexamethyl-2-(trimethylsilyl)trisilan-2-yl)bicyclo[1.1.1]pentan-1-yl)methyl)-*N*-phenylpent-2-enamide (8p)

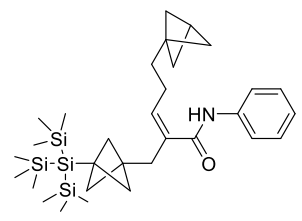

^{1}H NMR (600 MHz, CDCl$_3$)

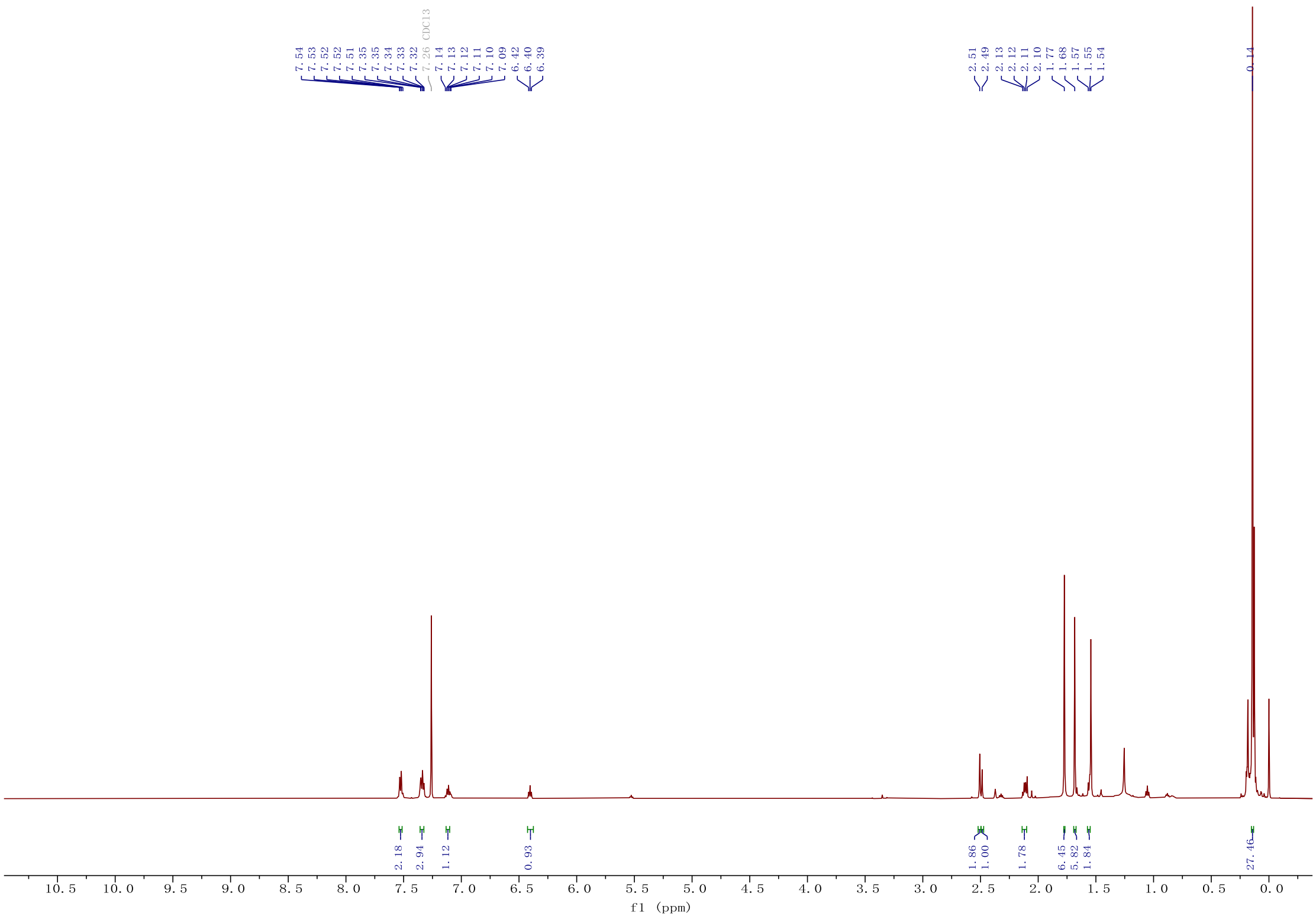

^{13}C NMR (151 MHz, CDCl$_3$)

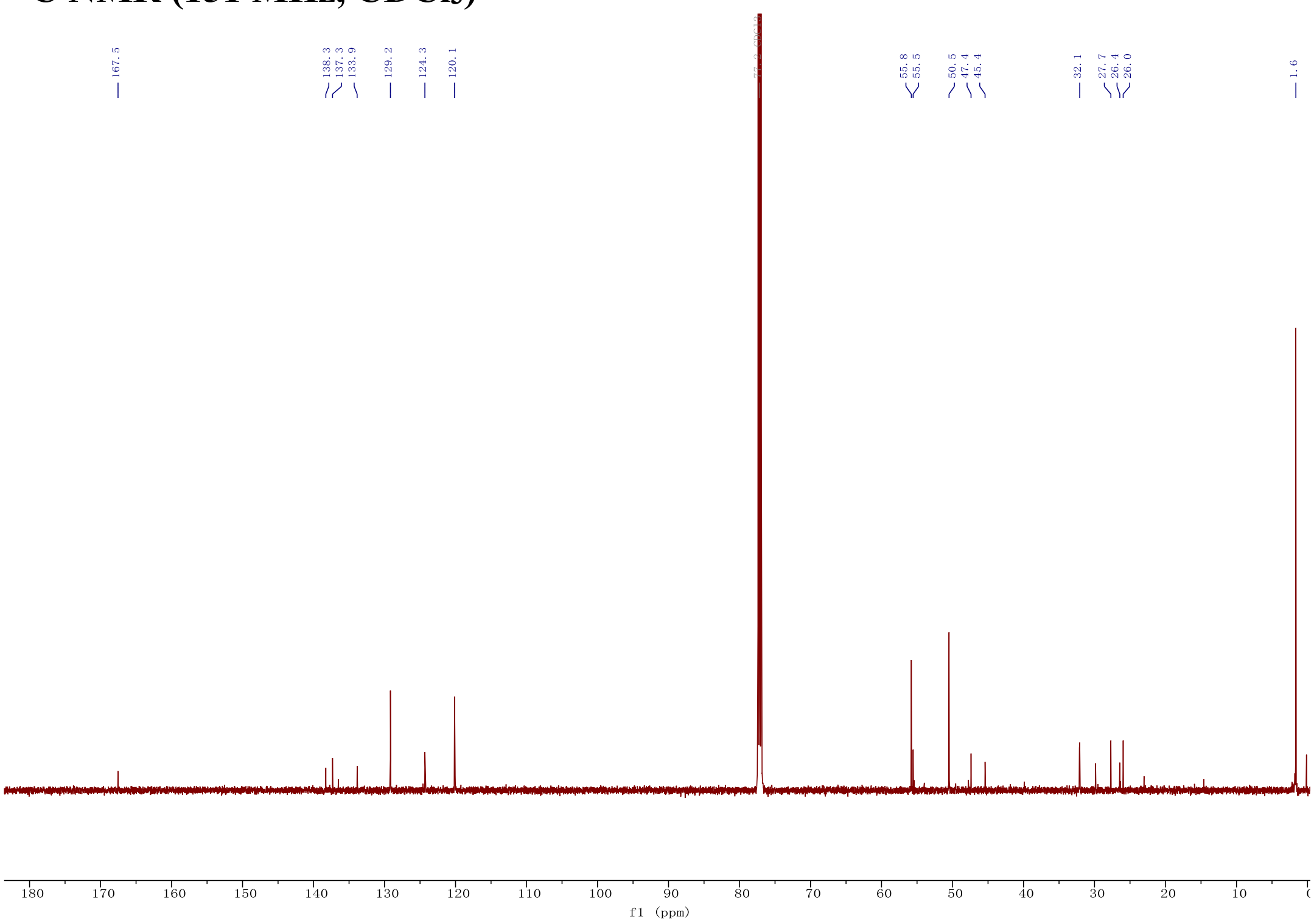

(*Z*)-2-((3-(1,1,1,3,3,3-hexamethyl-2-(trimethylsilyl)trisilan-2-yl)bicyclo[1.1.1]pentan-1-yl)methyl)-*N*-phenylpent-2-enamide (8q)

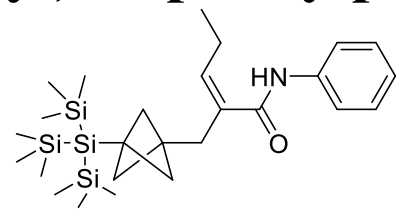

^{1}H NMR (600 MHz, CDCl$_3$)

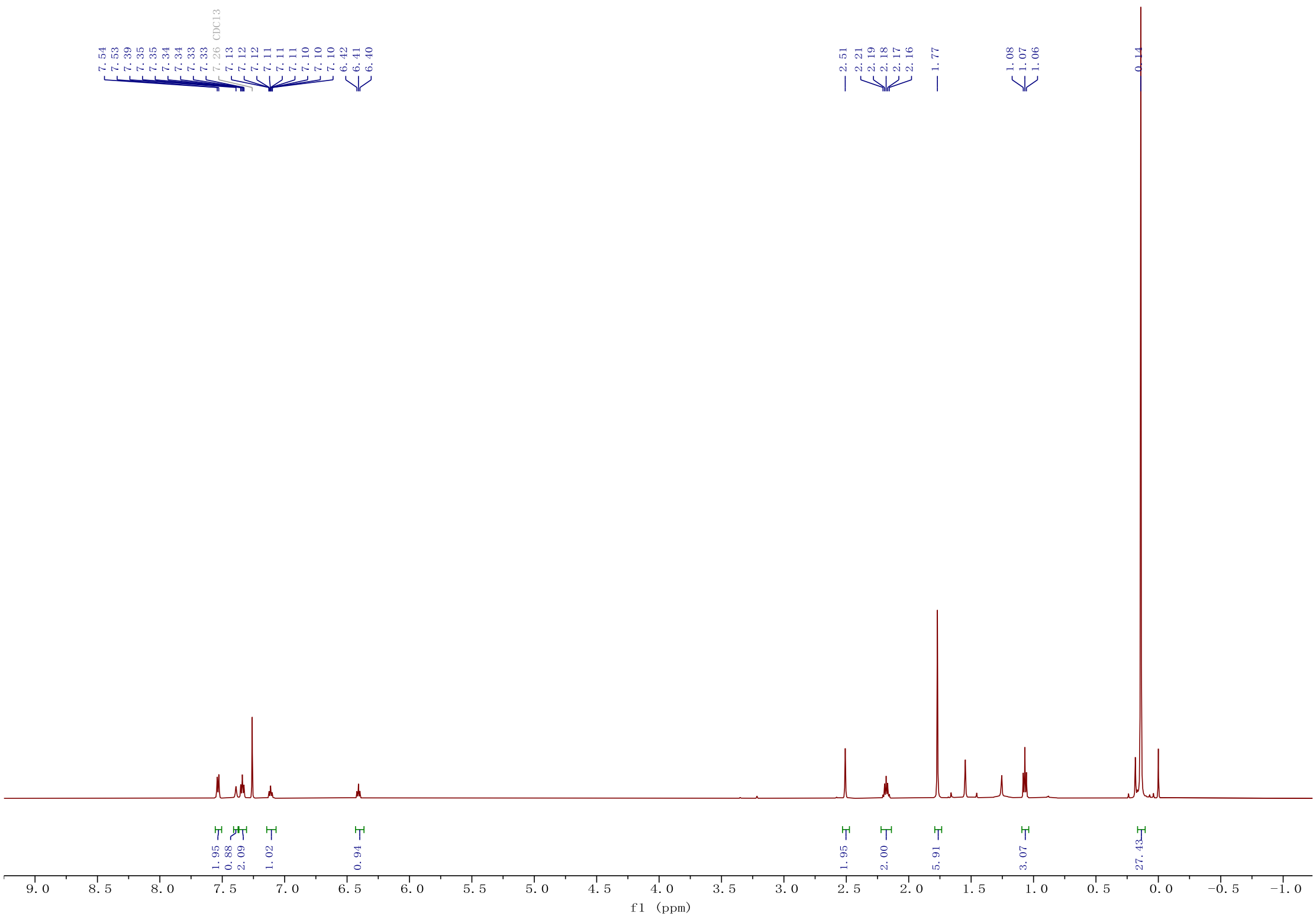

^{13}C NMR (151 MHz, CDCl$_3$)

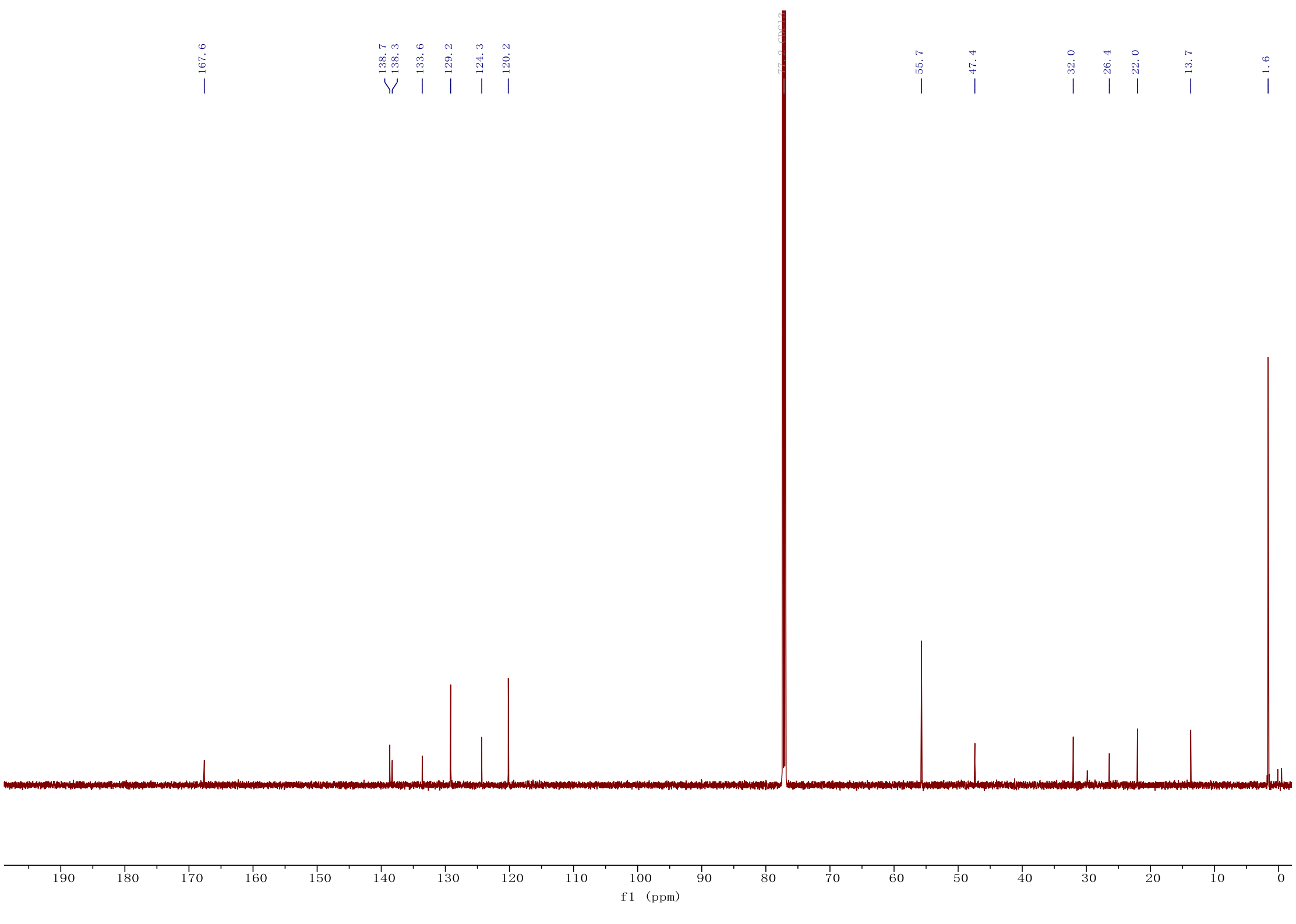

2-(bicyclo[1.1.1]pentan-1-yl-3-d)-3-(3-(1,1,1,3,3,3-hexamethyl-2-(trimethylsilyl)trisilan-2-yl)bicyclo[1.1.1]pentan-1-yl)-*N*-phenylpropanamide (8r)

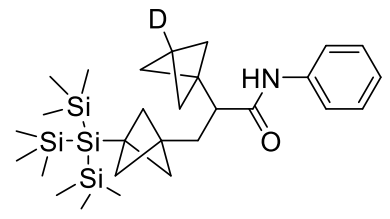

^{1}H NMR (600 MHz, $CDCl_3$)

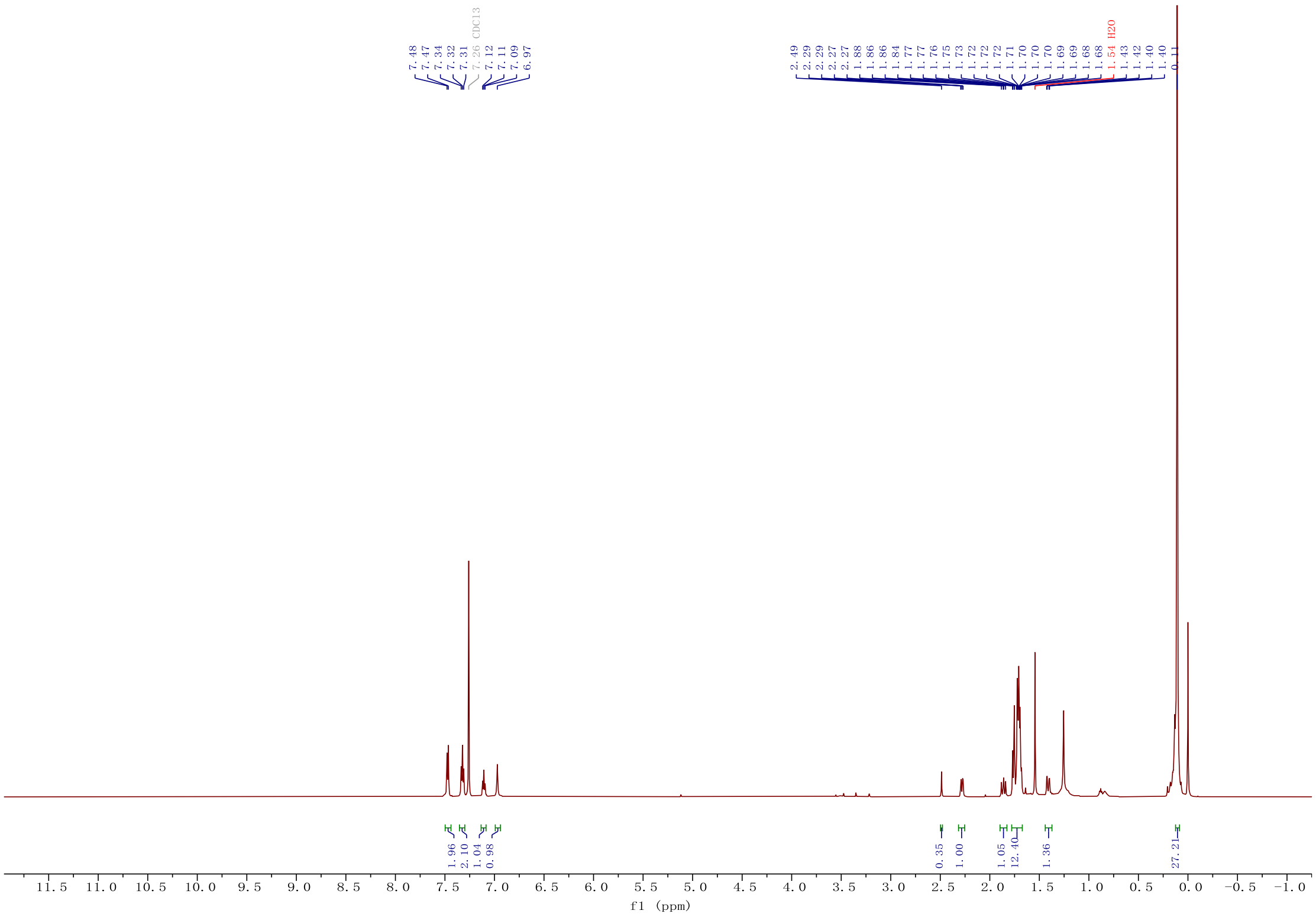

^{13}C NMR (151 MHz, $CDCl_3$)

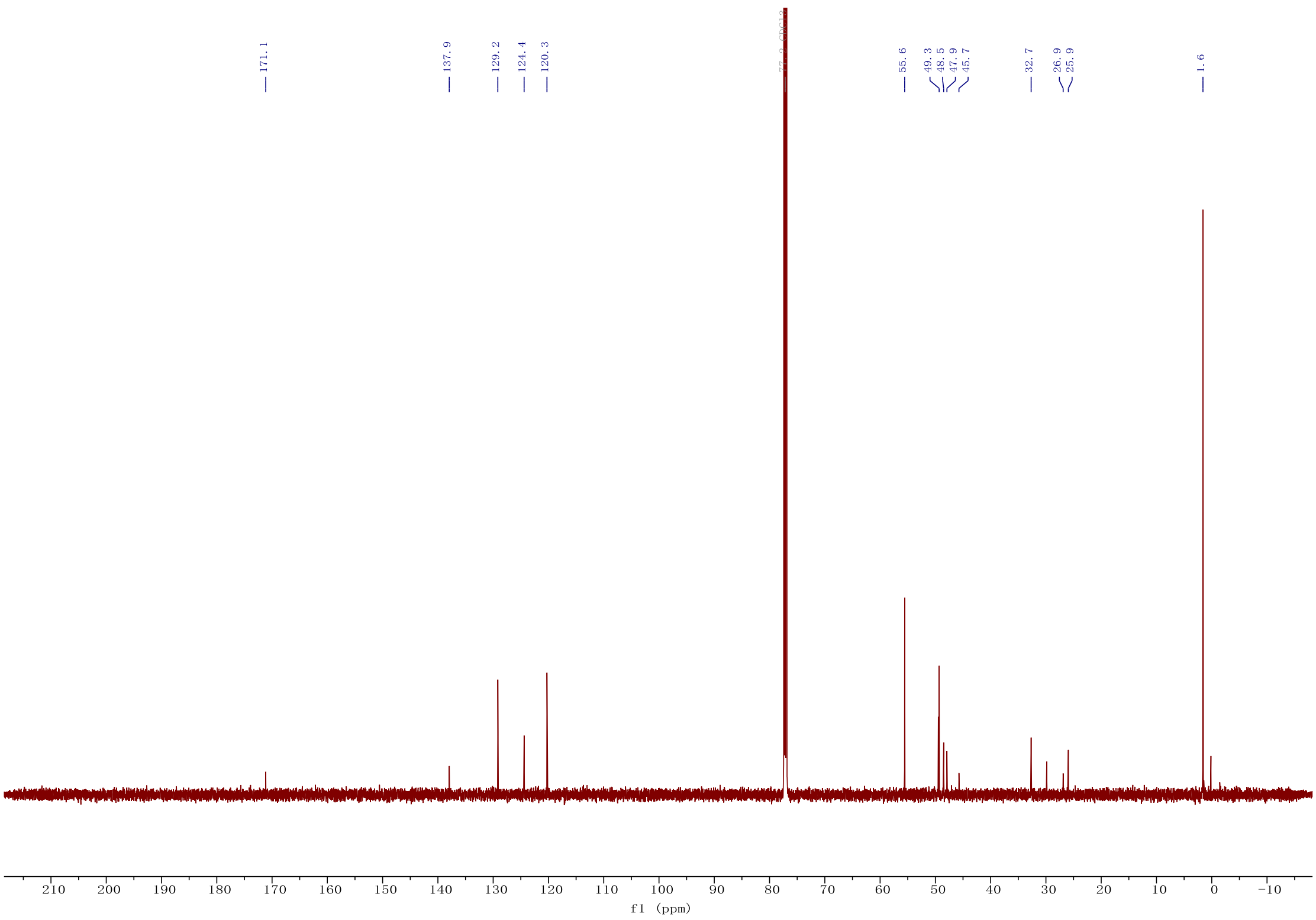

6. References.

1 J. Shen, L. Li, Q. Pang, L.-F. Yang, K. Ge, C. Shen and P. Zhang, *J. Org. Chem.*, 2025, **90**, 9223–9235.

2 N. Frank, J. Nugent, B. R. Shire, H. D. Pickford, P. Rabe, A. J. Sterling, T. Zarganes-Tzitzikas, T. Grimes, A. L. Thompson, R. C. Smith, C. J. Schofield, P. E. Brennan, F. Duarte and E. A. Anderson, *Nature*, 2022, **611**, 721–726.

3 C. Y. Legault, *CYLview20*, Université de Sherbrooke, 2020, http://www.cylview.org.

4 L. A. Bateman, T. B. Nguyen, A. M. Roberts, D. K. Miyamoto, W. M. Ku, T. R. Huffman, Y. Petri, M. J. Heslin, C. M. Contreras, C. F. Skibola, J. A. Olzmann and D. K. Nomura, *Chem. Commun.*, 2017, **53**, 7234–7237.

5 A. G. Németh, R. Szabó, K. Németh, G. M. Keserű and P. Ábrányi-Balogh, *Org. Biomol. Chem.*, 2022, **20**, 4361–4368.

6 C. E. Allen, P. R. Curran, A. S. Brearley, V. Boissel, L. Sviridenko, N. J. Press, J. P. Stonehouse and A. Armstrong, *Org. Lett.*, 2015, **17**, 458–460.

7 H. Li, R. Ke, Y. Zhou, S. Chang, J. Wang, C. Su, P. Wu, B. Yang, Z. Wang, K. Ding and D. Ma, *Eur. J. Med. Chem.*, 2024, **272**, 116473.

8 F. Pertusati, S. Ferla, M. Bassett, A. Brancale, S. Khandil, A. D. Westwell and C. McGuigan, *Eur. J. Med. Chem.*, 2019, **180**, 1–14.

9 L. Ackerman, A. V. Lygin and N. Hofmann, *Org. Lett.*, 2011, **13**, 3278–3281.

10 A. S. K. Lahdenperä, P. D. Bacoş and R. J. Phipps, *J. Am. Chem. Soc.*, 2022, **144**, 22451–22457.

11 M. Abuaf and Y. Mastai, *Macromol. Chem. Phys.*, 2022, **221**, 200328.

12 S. Liu, L. Zhang, L. Xu, P. Gao, X. Duan and L. Guo, *Org. Lett.*, 2023, **25**, 1336–1341.

13 D. Wu, W. Fan, L. Wu, P. Chen and G. Liu, *ACS Catal.*, 2022, **12**, 5284–5291.

14 X. Zhao, W. Li, L. Zhou, X. Zhao, Y. Zhang, B. Li, R. Li and L. Zhu, *J. Org. Chem.*, 2024, **89**, 8334–8341.

15 Q. Tang, S. Cai, C. Wang, G. Lin and X. Sun, *Org. Lett.*, 2020, 22, 3351–3355.

16 Y. Fang, Z. Yang and H. Park, *Synth. Commun.*, 2014, **44**, 1212–1217.

17 (*a*) J. Nugent, C. Arroniz, B. R. Shire, A. J. Sterling, H. D. Pickford, M. L. J. Wong, S. J. Mansfield, D. F. J. Caputo, B. Owen, J. J. Mousseau, F. Duarte and E. A. Anderson, *ACS Catal.*, 2019, **9**, 9568–9574; (*b*) J. Nugent, B. R. Shire, D. F. J. Caputo, H. D. Pickford, F. Nightingale, I. T. T. Houlsby, J. J. Mousseau and E. A. Anderson, *Angew. Chem., Int. Ed.*, 2020, **59**, 11866–11870; (*c*) H. D. Pickford, J. Nugent, B. Owen, J. J. Mousseau, R. C. Smith and E. A. Anderson, *J. Am. Chem. Soc.*, 2021, **143**, 9729–9736.

18 A. K. Chandra and T. Uchimaru, *J. Phys. Chem. A*, 2000, **104**, 9244–9249.

19 H.-J. Wang and Y. Fu, *J. Mol. Struct.: THEOCHEM*, 2009, **893**, 67–72.

7. 3D Coordinates of optimized structures, (thermal correction) and energy associated (Hartree) from DLPNO-CCSD(T), charge and multiplicity.

DCM: (0.003614) -958.7680886

0 1

C	0.000000	-0.000000	0.774774
H	0.898211	-0.000000	1.380751
H	-0.898211	0.000000	1.380751
Cl	0.000000	1.466681	-0.217946
Cl	-0.000000	-1.466681	-0.217946

[1.1.1]propellane: (0.068814) -193.719234

0 1

C	0.767836	-0.000000	0.000209
C	-0.000000	-1.122799	-0.648741
C	-0.767836	0.000000	0.000209
C	0.000000	0.000000	1.297121
H	-0.000000	-2.077889	-0.138859
H	-0.000000	-1.159437	-1.730860
H	0.000000	-0.919007	1.869553
H	0.000000	0.919007	1.869553
C	0.000000	1.122799	-0.648741
H	0.000000	1.159437	-1.730860
H	0.000000	2.077889	-0.138859

Int-1: (-0.011396) -958.0995351

0 2

C	0.011165	0.694353	-0.000000
H	-0.446596	1.671721	-0.000000
Cl	0.011165	-0.171701	1.460385
Cl	0.011165	-0.171701	-1.460385

TS-1: (0.075064) -1151.812651

0 2

C	-1.102707	-0.007530	-0.143551
C	-2.608623	-0.005315	0.283768
C	-1.972376	1.189439	-0.407136
H	-1.796061	2.068994	0.200419
H	-2.263509	1.372275	-1.434510
C	-2.056386	-1.031010	-0.692378
H	-1.952056	-2.045888	-0.327729

H	-2.350637	-0.926636	-1.729808
C	-1.489161	-0.177946	1.297838
H	-1.365972	-1.165009	1.727366
H	-1.296847	0.656942	1.961048
C	1.121913	-0.033464	-0.611461
H	1.115030	0.123560	-1.681676
Cl	1.655936	-1.600654	-0.137517
Cl	1.779324	1.283039	0.283594

Int-2: (0.081226) -1151.860642

0 2

C	0.738332	-0.000261	-0.138134
C	1.752153	-1.095009	-0.541526
C	2.444914	-0.000152	0.279110
C	1.752107	1.094766	-0.541254
H	2.000147	-1.150476	-1.598425
H	1.622725	-2.061774	-0.062542
H	2.000074	1.150332	-1.598140
H	1.622513	2.061423	-0.062085
C	1.295130	0.000078	1.298800
H	1.145948	-0.912426	1.869837
H	1.145878	0.912762	1.869570
C	-0.719140	-0.000037	-0.541718
H	-0.830911	0.000061	-1.622076
Cl	-1.538262	1.459187	0.067785
Cl	-1.538439	-1.459599	0.067278

Cyano acrylate: (0.025426) -170.5809775

0 1

C	0.015420	-0.020767	-0.140341
N	1.158117	0.072271	-0.123670
C	-1.519339	-0.145722	-0.162729
H	-2.128014	0.684433	0.129260
C	-2.099015	-1.307106	-0.552253
H	-3.165373	-1.393925	-0.567810
H	-1.490340	-2.137261	-0.844243

TS-2: (0.123237) -1322.445403

0 2

C	-3.217963	-1.432046	-0.243222
H	-3.249321	-1.669067	0.812099

H	-2.918479	-2.216033	-0.925438
C	-3.756381	-0.294465	-0.718733
H	-3.806475	-0.073390	-1.776823
C	-4.235089	0.717627	0.160176
N	-4.621568	1.541653	0.865009
C	-0.864452	-0.943495	0.117363
C	0.251091	-1.601393	0.937694
H	0.677264	-2.519316	0.542600
H	0.141929	-1.585778	2.018915
C	-0.552893	0.427082	0.717062
H	-0.833545	1.296064	0.127506
H	-0.703834	0.544547	1.787248
C	0.094580	-0.688678	-1.051093
H	0.510017	-1.562639	-1.545872
H	-0.158703	0.128431	-1.721688
C	0.751566	-0.288503	0.286856
C	2.137833	0.278322	0.481248
H	2.340091	0.473902	1.530289
Cl	3.372042	-0.875358	-0.082593
Cl	2.308289	1.825709	-0.382311

Int-3: (0.12731) -1322.512347

0 2

C	-2.477104	-0.749628	0.969835
H	-2.760687	-0.016901	1.726752
H	-2.481077	-1.741257	1.430054
C	-3.444823	-0.733839	-0.162090
H	-3.488562	-1.557977	-0.861674
C	-4.265639	0.362585	-0.416064
N	-4.948865	1.276808	-0.613559
C	-1.072942	-0.447212	0.485633
C	0.212449	-0.416547	1.350142
H	0.554465	-1.374826	1.739925
H	0.284271	0.390630	2.078374
C	-0.587753	0.861315	-0.183749
H	-0.964934	1.062759	-1.186584
H	-0.563885	1.748314	0.448432
C	-0.267701	-1.225028	-0.583768
H	0.048096	-2.232558	-0.314705
H	-0.624104	-1.149670	-1.611264
C	0.637042	-0.074791	-0.091390
C	2.011026	0.233730	-0.595090
H	2.014564	0.455214	-1.658311

Cl	2.675962	1.668040	0.231186
Cl	3.097955	-1.161269	-0.356882

TS-3: (0.218175) -1516.222993

0 2

C	1.200064	-1.856475	0.449810
H	1.659812	-2.217854	-0.474907
H	0.956285	-2.748871	1.039063
C	2.225567	-1.047536	1.191320
H	3.130506	-1.562624	1.489130
C	1.837292	-0.015226	2.069359
N	1.553531	0.882725	2.739856
C	-0.079507	-1.134625	0.123634
C	-1.173926	-1.657895	-0.837575
H	-1.728369	-2.536586	-0.508931
H	-0.909769	-1.693641	-1.895019
C	-0.253996	0.259601	-0.535406
H	0.024048	1.121704	0.070243
H	0.062424	0.350366	-1.575103
C	-1.222546	-0.738843	1.102039
H	-1.775284	-1.565519	1.548470
H	-1.007201	0.060080	1.809376
C	-1.674039	-0.300494	-0.302813
C	-2.951113	0.375482	-0.688419
H	-3.002495	0.574713	-1.754970
Cl	-4.353232	-0.655552	-0.292016
Cl	-3.103620	1.950127	0.136175
C	4.051270	0.915652	-1.434973
C	3.219405	-0.307625	-1.779236
H	3.760986	-1.214051	-2.023719
H	2.304599	-0.136956	-2.334842
C	2.962695	1.586826	-0.616068
H	3.282013	2.296383	0.138148
H	2.042433	1.827290	-1.133613
C	4.733999	0.278145	-0.241681
H	5.325518	-0.610159	-0.429985
H	5.111284	0.947038	0.522836
C	3.233995	0.129987	-0.335971

Int-4: (0.221331) -1516.262676

0 2

C	-1.195414	-0.339040	0.705611

H	-1.405919	0.233204	1.612877
H	-1.394424	-1.391160	0.924513
C	-2.165819	0.090902	-0.399476
H	-1.905835	-0.406619	-1.338254
C	-2.094203	1.534353	-0.634622
N	-2.071665	2.670216	-0.794528
C	0.250423	-0.183463	0.324890
C	1.463845	-0.561488	1.212740
H	1.603753	-1.624439	1.409220
H	1.627287	0.058866	2.093612
C	1.011480	1.118343	-0.047394
H	0.754774	1.582781	-0.998866
H	1.148168	1.844247	0.753730
C	0.981471	-0.870634	-0.857867
H	1.094913	-1.952637	-0.793088
H	0.719182	-0.530192	-1.859907
C	2.040106	-0.030184	-0.111713
C	3.477618	0.108309	-0.500077
H	3.593520	0.520057	-1.498410
Cl	4.325322	1.214214	0.614091
Cl	4.285346	-1.483431	-0.505147
C	-5.278611	-0.706136	0.340447
C	-4.136708	-1.712532	0.153292
H	-4.172666	-2.339436	-0.734461
H	-3.787276	-2.227094	1.044783
C	-4.383620	0.187579	1.208280
H	-4.639194	1.243575	1.255207
H	-4.044931	-0.228579	2.153886
C	-4.855342	0.050970	-0.922953
H	-4.929864	-0.484556	-1.866314
H	-5.134045	1.100141	-0.985727
C	-3.619716	-0.271629	-0.054408

TS-4: (0.235469) -2475.012599

0 2

C	0.586862	0.137839	0.560867
H	0.392003	0.810535	1.400112
H	0.214128	-0.851645	0.837325
C	-0.215319	0.606591	-0.658620
H	0.053362	0.005935	-1.532739
C	0.085424	2.003451	-0.981730
N	0.288495	3.108472	-1.213851
C	2.065509	0.049752	0.306309

C	3.039333	1.187437	-0.105904
H	3.217451	1.964653	0.636903
H	2.931043	1.584405	-1.114760
C	2.778681	-0.849046	-0.737366
H	2.724186	-1.924039	-0.566701
H	2.651220	-0.578124	-1.785738
C	3.133525	-0.404475	1.334059
H	3.315934	0.271592	2.169043
H	3.096484	-1.451456	1.634892
C	3.887278	-0.095243	0.028268
C	5.355640	-0.202260	-0.234752
H	5.611975	0.095672	-1.247434
l	6.271072	0.865583	0.862907
l	5.908322	-1.888369	-0.040284
C	-3.484739	0.302615	-0.155866
C	-2.835050	0.901889	-1.414431
H	-2.956391	1.972684	-1.565155
H	-2.913779	0.319596	-2.330161
C	-2.471362	-0.854763	-0.184317
H	-2.276561	-1.352473	0.763152
H	-2.529230	-1.535243	-1.031450
C	-2.524166	1.118931	0.726623
H	-2.631265	2.201468	0.693424
H	-2.330276	0.729421	1.723931
C	-1.722501	0.475852	-0.429901
H	-4.801136	0.172644	0.102124
C	-6.068255	0.047201	0.495858
H	-6.714503	0.106275	-0.373544
Cl	-6.197214	-1.524350	1.256443
Cl	-6.375836	1.380479	1.587531

Int-5: (0.235015) -1516.943552

0 1			
C	-1.149732	-0.327934	0.690795
H	-1.360456	0.237630	1.602345
H	-1.355173	-1.380567	0.901955
C	-2.118612	0.113532	-0.413727
H	-1.846401	-0.373712	-1.355587
C	-2.033063	1.559674	-0.638598
N	-1.997660	2.696333	-0.791331
C	0.298437	-0.178016	0.318420
C	1.506425	-0.573860	1.206107
H	1.638637	-1.639872	1.391269

H	1.670923	0.035398	2.094550
C	1.069752	1.122710	-0.036842
H	0.819503	1.599409	-0.983963
H	1.208343	1.838880	0.772718
C	1.029607	-0.856739	-0.869352
H	1.135734	-1.940160	-0.816379
H	0.773200	-0.503515	-1.868486
C	2.090999	-0.031742	-0.110178
C	3.530742	0.101305	-0.492145
H	3.653100	0.522537	-1.485723
Cl	4.382091	1.190269	0.636152
Cl	4.328160	-1.495589	-0.510983
C	-5.304565	-0.690129	0.334239
C	-4.815282	0.073537	-0.917601
H	-5.096336	1.124513	-0.988294
H	-4.900478	-0.448097	-1.871141
C	-4.120126	-1.663357	0.130661
H	-3.774226	-2.196844	1.015915
H	-4.157925	-2.299878	-0.753690
C	-4.353405	0.196638	1.172267
H	-4.605352	1.255442	1.237445
H	-4.021137	-0.214466	2.125660
C	-3.558250	-0.239749	-0.078782
H	-6.330035	-0.955682	0.577516

TS-5: (0.09436) -2110.610935

0 2

C	1.255212	0.005837	0.453725
C	-0.526937	0.003590	0.596065
C	0.409323	-1.081798	1.156057
H	0.379831	-2.052202	0.666305
H	0.503566	-1.137651	2.238256
C	0.405932	1.096320	1.147693
H	0.373829	2.063106	0.651004
H	0.500065	1.160057	2.229516
C	0.263926	-0.000867	-0.727815
H	0.220256	0.907742	-1.323918
H	0.223492	-0.915173	-1.315432
C	2.759943	0.007872	0.380368
H	3.205959	0.013505	1.370658
Cl	3.344667	1.464945	-0.461654
Cl	3.348711	-1.454838	-0.448715
H	-1.878191	-0.004846	0.642953

C	-3.195835	-0.029571	0.514283
H	-3.651104	0.011934	1.498312
Cl	-3.575543	-1.541215	-0.281920
Cl	-3.619229	1.384374	-0.426169

Int-6: (0.094588) -1152.541219

0 1

C	0.033685	0.687109	-0.000000
C	-0.720596	2.371622	-0.000000
C	0.223548	1.780843	1.073597
H	-0.215733	1.574271	2.048758
H	1.219060	2.218663	1.142717
C	0.223548	1.780843	-1.073597
H	-0.215733	1.574271	-2.048758
H	1.219060	2.218663	-1.142717
C	-1.471425	1.016464	-0.000000
H	-2.018781	0.764249	-0.907549
H	-2.018781	0.764249	0.907549
C	0.681533	-0.662273	-0.000000
H	1.765705	-0.595248	0.000000
Cl	0.224425	-1.580453	-1.460503
Cl	0.224425	-1.580453	1.460503
H	-1.166096	3.362426	-0.000000

TS-6: (0.147954) -2281.248422

0 2

C	1.110215	1.476219	1.513168
H	1.441852	0.768444	2.277230
H	0.850033	2.401860	2.037480
C	2.284809	1.741240	0.593549
H	3.150007	2.187245	1.080053
C	2.016269	2.359822	-0.670213
N	1.813804	2.817344	-1.706919
C	-0.115618	0.936840	0.823124
C	-0.188097	-0.151846	-0.282859
H	0.091740	-1.164290	0.007025
H	0.189667	0.128172	-1.266517
C	-1.173332	1.734549	0.013148
H	-1.781956	2.436218	0.583223
H	-0.859164	2.129422	-0.951809
C	-1.292379	0.213621	1.525010
H	-1.070271	-0.771027	1.937142

H	-1.908803	0.822510	2.186079
C	-1.642380	0.264855	0.027121
C	-2.863896	-0.269241	-0.650069
H	-2.851965	-0.087129	-1.720962
Cl	-2.983010	-2.036338	-0.429399
Cl	-4.331093	0.520593	-0.010679
H	2.774591	0.549677	0.269145
C	3.368110	-0.660137	0.051738
H	4.428266	-0.456861	0.164888
Cl	2.792939	-1.738720	1.288205
Cl	2.980937	-1.135552	-1.572350

Int-7: (0.143221) -1323.175401

0 1

C	-2.495629	-0.937399	0.939798
H	-2.764518	-0.349710	1.820325
H	-2.484944	-1.988003	1.237230
C	-3.571119	-0.770010	-0.138879
H	-4.518663	-1.197133	0.191993
C	-3.816154	0.633308	-0.462683
N	-4.005641	1.739497	-0.699202
C	-1.122402	-0.548539	0.469105
C	0.175634	-0.598757	1.314874
H	0.529192	-1.590761	1.595985
H	0.249124	0.123976	2.127226
C	-0.646667	0.832238	-0.060615
H	-1.027686	1.142364	-1.033120
H	-0.623910	1.646674	0.663096
C	-0.317821	-1.194638	-0.689261
H	0.008217	-2.223367	-0.536714
H	-0.680998	-1.009924	-1.700527
C	0.583981	-0.098668	-0.082112
C	1.951200	0.271001	-0.562495
H	1.948191	0.592488	-1.599960
Cl	2.608952	1.626198	0.393677
Cl	3.052218	-1.130462	-0.463011
H	-3.280542	-1.281814	-1.058832

TS-7: (0.164822) -1345.571535

0 2

C	-1.073316	-0.001804	0.292654
C	0.687980	-0.045943	0.080007

C	-0.106758	1.166152	0.591968
H	-0.176679	2.024425	-0.072078
H	0.017565	1.428343	1.640486
C	-0.110733	-0.975739	1.008549
H	-0.184088	-2.019973	0.714380
H	0.013246	-0.826482	2.078980
C	-0.348108	-0.262186	-1.041233
H	-0.430127	-1.269050	-1.443899
H	-0.427435	0.520776	-1.791784
C	-2.565216	0.046257	0.522786
H	-2.799456	0.250630	1.563530
Cl	-3.317422	-1.518040	0.118415
Cl	-3.310044	1.346630	-0.441986
C	3.046104	-0.022757	-0.083494
C	4.615641	0.023446	-0.139287
C	3.850450	0.098887	1.175946
H	3.839654	1.058065	1.679983
H	3.898388	-0.771911	1.818968
C	3.822143	-1.164354	-0.668818
H	3.787162	-1.287306	-1.744724
H	3.869367	-2.079289	-0.090353
C	3.750351	1.064620	-0.838169
H	3.712993	1.021393	-1.920166
H	3.736295	2.057581	-0.404673

Int-8: (0.172207) -1345.635147

0 1

C	-0.870639	-0.000019	0.306951
C	0.960577	-0.000035	0.117921
C	0.106008	1.075954	0.831150
H	0.051580	2.050098	0.346420
H	0.215853	1.142549	1.913397
C	0.105986	-1.076014	0.831140
H	0.051539	-2.050153	0.346402
H	0.215830	-1.142622	1.913386
C	-0.090242	-0.000019	-1.022156
H	-0.153224	-0.909137	-1.619485
H	-0.153206	0.909106	-1.619475
C	-2.353967	-0.000005	0.500718
H	-2.627728	-0.000008	1.551870
Cl	-3.082924	-1.460024	-0.220376
Cl	-3.082893	1.460038	-0.220360
C	2.449294	-0.000020	-0.038261

C	4.207577	0.000043	-0.223885
C	3.474049	0.000005	1.122883
H	3.527762	0.911504	1.713847
H	3.527827	-0.911500	1.713831
C	3.275461	-1.093205	-0.760038
H	3.152350	-1.151551	-1.839068
H	3.318813	-2.062708	-0.269050
C	3.275389	1.093235	-0.760021
H	3.152272	1.151588	-1.839051
H	3.318671	2.062733	-0.269017
